# Metagenomic analysis of captive Amur tiger faecal microbiome

**DOI:** 10.1186/s12917-018-1696-5

**Published:** 2018-12-04

**Authors:** Fengping He, Dan Liu, Le Zhang, Jiancheng Zhai, Yue Ma, Yanchun Xu, Guangshun Jiang, Ke Rong, Jianzhang Ma

**Affiliations:** 1 College of Wildlife Resources, Northeast Forestry University, Harbin, 150040 China; 2 Heilongjiang Siberian Tiger Park, Harbin, 150040 China; 3 State Forestry Administration Detecting Center of Wildlife, Harbin, 150040 China; 4 China Feline Research Center of Chinese State Forestry Administration, Harbin, 150040 China

**Keywords:** Gastrointestinal microbiota, Amur tiger, *Panthera tigris altaica*, Metagenomics, Pyrosequencing

## Abstract

**Background:**

The gastrointestinal tracts of animals are home to large, complex communities of microbes. The compositions of these communities ultimately reflect the coevolution of microorganisms with their animal host and are influenced by the living environment, diet and immune status of the host. Gut microbes have been shown to be important for human disease and health, but little research exists in the gut microbiome of the Amur tiger, which is one of the most endangered species in the world.

**Results:**

In this study, we present the use of whole-metagenome shotgun sequencing to analyze the composition and functional structures of the gut microbiota in captive Amur tigers. Our results showed a high abundance of four major phyla in captive Amur tigers, including Proteobacteria, Firmicutes, Actinobacteria and Fusobacteria. Moreover, at the genus level, *Escherichia*, *Collinsella* and *Fusobacterium* were most abundant in the captive Amur tiger fecal metagenome. At the species level, *Escherichia coli*, *Fusobacterium ulcerans* and *Fusobacterium varium* were the species with highest abundances in the captive Amur tiger gut microbiota. The primary functional categories of the Amur tiger faecal metagenome were associated mainly with Carbohydrate metabolism, Membrane transport and Amino acid metabolism based on the KEGG pathway database. The comparative metagenomic analyses showed that the captive Amur tiger fecal metagenome had a lower abundance of Spirochaetes, Cyanobacteria and Ascomycota than other animals, and the primary functional categories were primarily associated with carbohydrate metabolism subsystems, clustering-based subsystems and protein metabolism.

**Conclusions:**

We presented here for the first time the use of the shotgun metagenomic sequencing approach to study the composition and functional structures of the gut microbiota in captive Amur tiger.

**Electronic supplementary material:**

The online version of this article (10.1186/s12917-018-1696-5) contains supplementary material, which is available to authorized users.

## Background

The Amur tiger (*Panthera tigris altaica*), also called the Siberian tiger, is the largest extant tiger subspecies in the genus *Panthera* (Mammalia, Carnivora, Felidae) [1]. Amur tigers, which are found in the Russian Far East [2] and Northeast China [3], are listed as Endangered on the IUCN Red List of Threatened Species™ and are included in CITES Appendix I. The population of wild Amur tiger continues to decline due to habitat loss and degradation, poaching and prey depletion [4].

Fortunately, the Amur tiger has been successfully bred in China, and the population of the Amur tiger has increased from 27 to approximately 1000 after more than 30 years of hard work. However, with the growing population of the Amur tiger, veterinary antibiotics and antiparasitics have been widely used to maintain the healthy development of the population in the Heilongjiang Siberian Tiger Park.

Many studies have shown that the gastrointestinal tracts of humans and animals contain large, complex microbial communities [5–7]. The gut microbiota is now recognized as a coevolutionary partner that facilitates host nutritional acquisition, immune modulation, and homeostasis in response to profound lifestyle changes [8–12]. The diversity and function of the gut microbiota may indicate host genotypic characteristics and reflect an adaptive ecosystem response to the host diet and living habits.

In recent years, metagenomic sequencing based on next-generation sequencing technologies [13] has been used to describe the microbial diversity and functional capacity of microbial communities in the gastrointestinal tracts of humans [14, 15] as well as some animal species [16–22]. In this study, for the first time, we used whole metagenome shotgun sequencing to profile the microbial flora inhabiting the digestive system of captive Amur tiger and identify the functional attributes encoded in the gut microbiome.

## Results

We sequenced the faecal metagenomes of 3 captive Amur tigers using the Illumina platform and obtained 32.39 GB of high-quality bases that were free of adaptor and tiger DNA contaminants (Additional file 1). The unique sequence reads that passed the quality control filtering step were then subjected to further analyses focused on biodiversity and functional annotation. The metagenomic sequence data from this study have been submitted to the National Center for Biotechnology Information (NCBI) Sequence Read Archive (SRA) under accession numbers SRP119699.

### Phylogenetic analysis of Amur tiger faecal bacteria, eukaryotes, archaea, and viruses

**Fig. 1:**
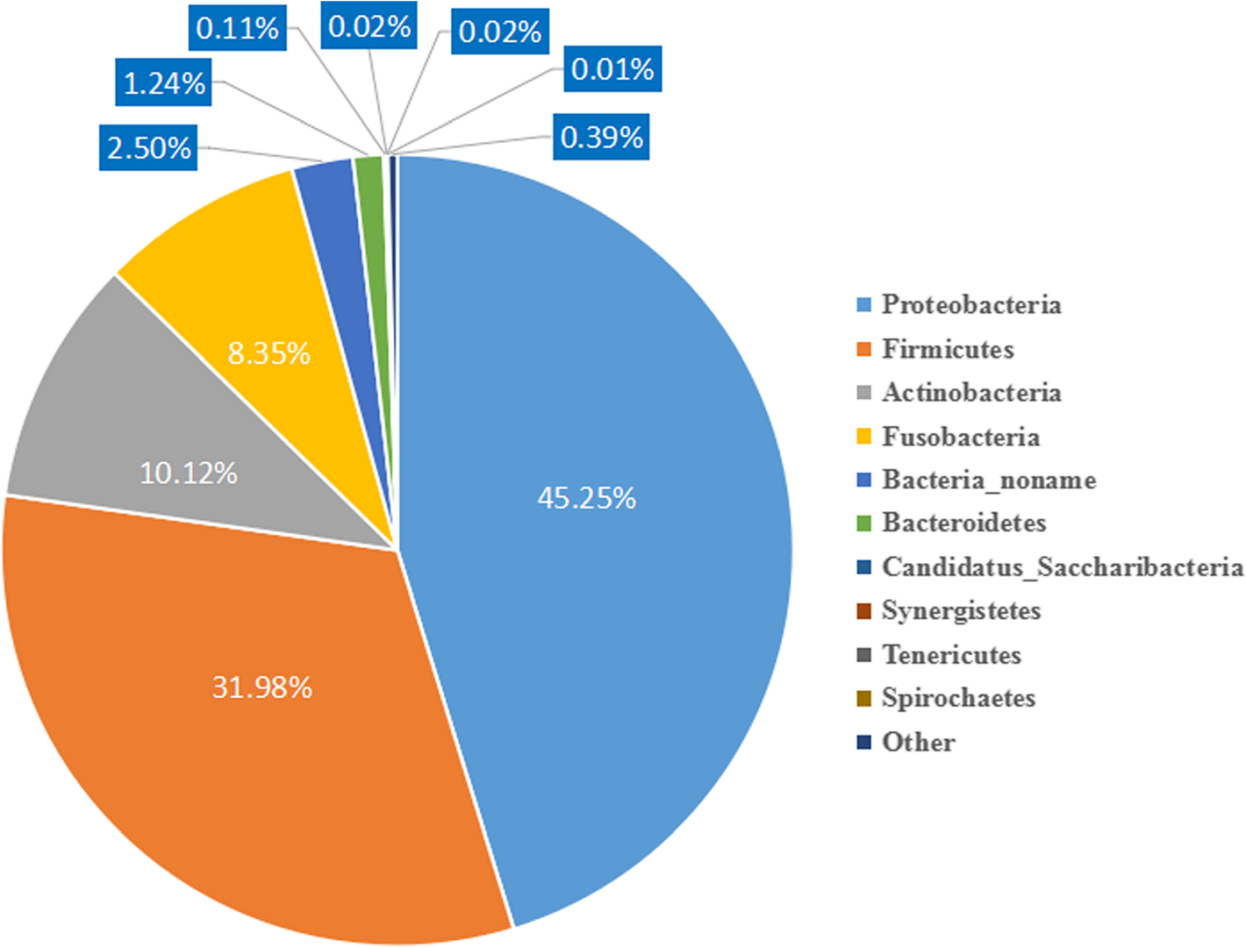
Bacterial phylum profiles of the Amur tiger microbiome

The phylogenetic computation revealed a profile comprising 98.10% bacteria, 1.89% viruses, 0.01% eukaryotes and 0.003% archaea. In the Amur tiger intestinal metagenome, Proteobacteria was the most predominant phylum (44.39%), followed by Firmicutes (31.38%), Actinobacteria (9.92%), and Fusobacteria (8.20%) (Fig. 1).

Of the Proteobacteria, most belonged to Enterobacteriaceae, and a very small amount were Betaproteobacteria or Pseudomonadales (Additional file 2). The dominant family of intestinal microbes in the Amur tiger is Enterobacteriaceae, and most of the Enterobacteriaceae were *Escherichia coli*. Firmicutes was the second most predominant phylum in captive Amur tiger gastrointestinal tract, with Clostridiales as the primary contributor to the Firmicutes population, followed by Erysipelotrichales and Selenomonadales (Additional file 2). Among Clostridiales, Lachnospiraceae, Peptostreptococcaceae and Clostridiaceae were the families with the highest abundance. A relatively high proportion of the Clostridiales content was attributable to *Ruminococcus gnavus*, *Clostridium hiranonis* and *Clostridium perfringens*. Erysipelotrichales contains a single family, Erysipelotrichaceae, and this family was represented mainly of *Erysipelotrichaceae bacterium nk3d112*, *Solobacterium moorei* and *Holdemanella biformis*. Selenomonadales was mostly represented by Veillonellaceae, including *Megamonas funiformis*, *M. rupellensis* and *M. hypermegale.*

Similarly, Coriobacteriia was the primary contributor to the population belonging to the phylum Actinobacteria, followed by the class Actinobacteria. The major genus in the Actinobacteria phylum was *Collinsella*. *Collinsella stercoris* and *C. intestinalis* were the predominant species among *Collinsella* in the Amur tiger metagenome. Actinobacteria was also represented by *Bifidobacterium*, *Actinomycetes* and *Corynebacterium*, with each genus represented by a variety of microbial species, such as *Actinomyces cardiffensis*, *Actinomyces turicensis, Corynebacterium diphtheriae* and *Bifidobacterium breve*. The major genus in the Fusobacteria phylum was *Fusobacterium*. *Fusobacterium ulcerans*, *F. varium, F. mortiferum* and *F. perfoetens* were the main species among the Fusobacteria in the Amur tiger metagenome. Fusobacteria are a phylum of bacteria that were significantly more abundant in Carnivora (e.g., Felidae and Canidae) [23]. A high proportion of Fusobacteria were also discovered in the gut microbiomes of some carnivorous vertebrates, such as vultures and alligators [24, 25].

Eukaryota was a minor constituent (0.01%) of the Amur tiger metagenome. Most of the Eukaryota were Ascomycota, with a small fraction of Basidiomycota (Additional file 3). Ascomycota is the largest Eukaryota phylum. *Trichophyton tonsurans* was the primary contributor to the Ascomycota population, followed by *Pseudogymnoascus pannorum*. Archaea sequences had very low abundance (0.003%) in the Amur tiger metagenome, with Euryarchaeota the primary contributor (Additional file 4). In the Amur tiger faecal metagenome, Methanosarcinaceae was the major Archaea component and included *Methanosarcina sp. WH1* and *Methanomethylovorans hollandica*. Only 1.89% of the Amur tiger metagenome sequences were viruses, of which Caudovirales was the only order identified (Additional file 5). The major family in Caudovirales was Podoviridae, followed by Siphoviridae and Myoviridae.

### Metabolic profiles of the Amur tiger metagenome

**Fig. 2:**
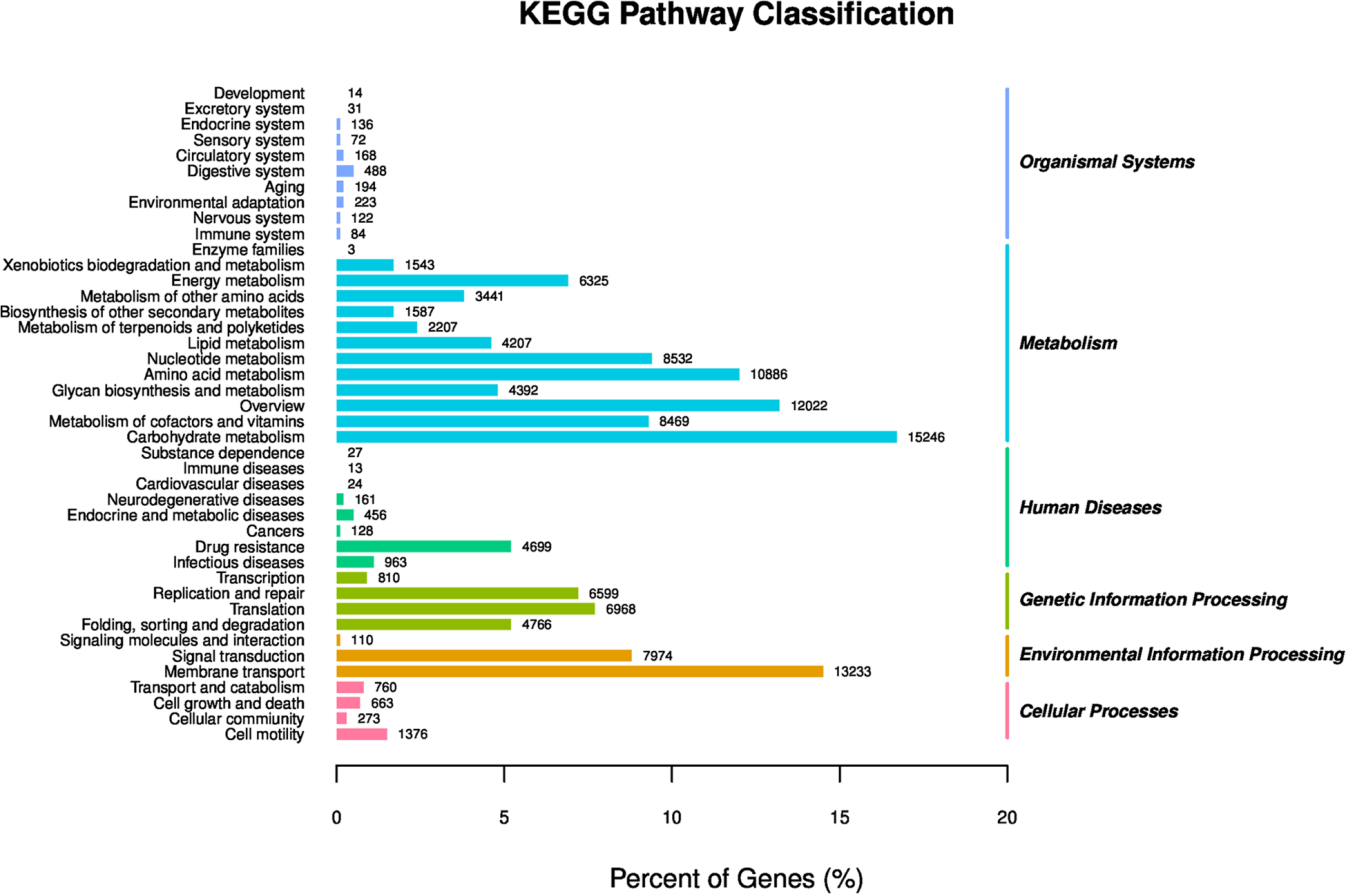
Functional composition of the Amur tiger microbiome

In addition to providing novel insights on the taxonomic diversity of the characteristic microbial consortium, our metagenomic research enabled the identification of the gene functions involved in the symbiotic functions with the host. Based on the KEGG PATHWAY database, hits were classified into 6 groups comprising 42 functional categories (Fig. 2).

Metabolism was the largest group, with higher proportions of Carbohydrate metabolism, Amino acid metabolism, Nucleotide metabolism, Metabolism of cofactors and vitamins and Energy metabolism. Collectively, the 12 functional categories in the metabolism group accounted for 86.62% of the hits. Carbohydrate metabolism and Amino acid metabolism were the most abundant functional categories, representing 16.75 and 11.96% of the Amur tiger faecal metagenome, respectively. Environmental information processing was the second most prominent functional group, Membrane transport was the most abundant functional categories, followed by Signal transduction and Signalling molecules and interaction. Approximately 14.54% of the annotated reads from the Amur tiger faecal metagenomes were categorized within Membrane transport.

The third most prominent functional group was Genetic information processing, which included the categories of Translation, Replication and repair, Folding, sorting and degradation. The fourth most prominent group, Human diseases, was dominated by the categories Drug resistance, Endocrine and metabolic diseases and Neurodegenerative diseases. The remaining two groups of captive Amur tiger faecal metagenomes were Cellular processes and Organismal systems. Cell motility, Transport and catabolism and Cell growth and death were important categories within Cellular processes. Most hits of the Organismal system group were distributed among the Digestive system, Environmental adaptation and Aging.

To fully describe the attributes of the genes and gene products identified by faecal metagenome sequencing of the Amur tiger, we performed GO annotations of the unigenes obtained from sequencing. A total of 401,606 unigenes were annotated with 7961 GO functions belonging to molecular function, biological process and cellular components (Additional file 6). The biological processes group contained 157,188 (39.14%) unigenes that were annotated with 4506 GO functions. The most abundant in the biological process group was biological processes, followed by transport and regulation of transcription, DNA-templated (Additional file 7). In the cellular components group, a total of 76,428 (19.03%) unigenes were annotated with 851 GO functions, mostly belonging to the cytosol, plasma membrane and cellular components. A total of 167,942 (41.82%) unigenes were annotated with 3051 GO functions in the molecular function group, in which molecular function was the largest category, followed by protein binding and ATP binding.

To further understand the carbohydrate enzymes present in the Amur tiger gut microbiome, we submitted the samples to the Carbohydrate-Active enZYmes database (CAZy). A total of 21,404 annotation results were obtained for the faeces metagenome sequences of the Amur tiger (Additional file 8). Among the 6 large functional CAZy classes, glycosyl transferase (GT) families were the most abundant, followed by glycoside hydrolase (GH) families (Additional file 9). In the further functional subclasses, GT4 and GT2 belonging to the GT family were the most abundant, followed by CBM50 of the CBM family and GH23 and GH13 of the GH family.

## Discussion

In this study, whole-metagenome shotgun sequencing was performed to assess the composition and functional structure of the gut microbiota of the captive Amur tiger. In our study, we observed a high abundance of four major phyla, Proteobacteria, Firmicutes, Actinobacteria and Fusobacteria. Moreover, at the genus level, *Escherichia*, *Collinsella* and *Fusobacterium* were most abundant in the captive Amur tiger fecal metagenome. Importantly, at the species level, *Escherichia coli*, *Fusobacterium ulcerans* and *Fusobacterium varium* were the species with the highest abundances in the captive Amur tiger gut microbiota.

Despite extensive variation among individuals, the gut microbiota of members of the same species are often more similar to one another compared with those of other species. Thus, it is important to provide a comparison between the gut microbiota of the Amur tiger and those of other animals. The results of this study were compared with data sets from different animals, including humans, in the MG-RAST database. Paired data from other studies were chosen, including three wild Amur tigers (WT1, WT2, and WT3), two cats (cat 1 and cat 2), two chickens (chicken 1 and chicken 2), two humans (human 1 and human 2), two mice (mouse 1 and mouse 2), two pandas (panda 1 and panda 2), two pigs (pig 1 and pig 2) and two wolves (wolf 1 and wolf 2), which were compared with Amur tigers in captivity (ptg_z1, ptg_z2, and ptg_z3).

**Fig. 3:**
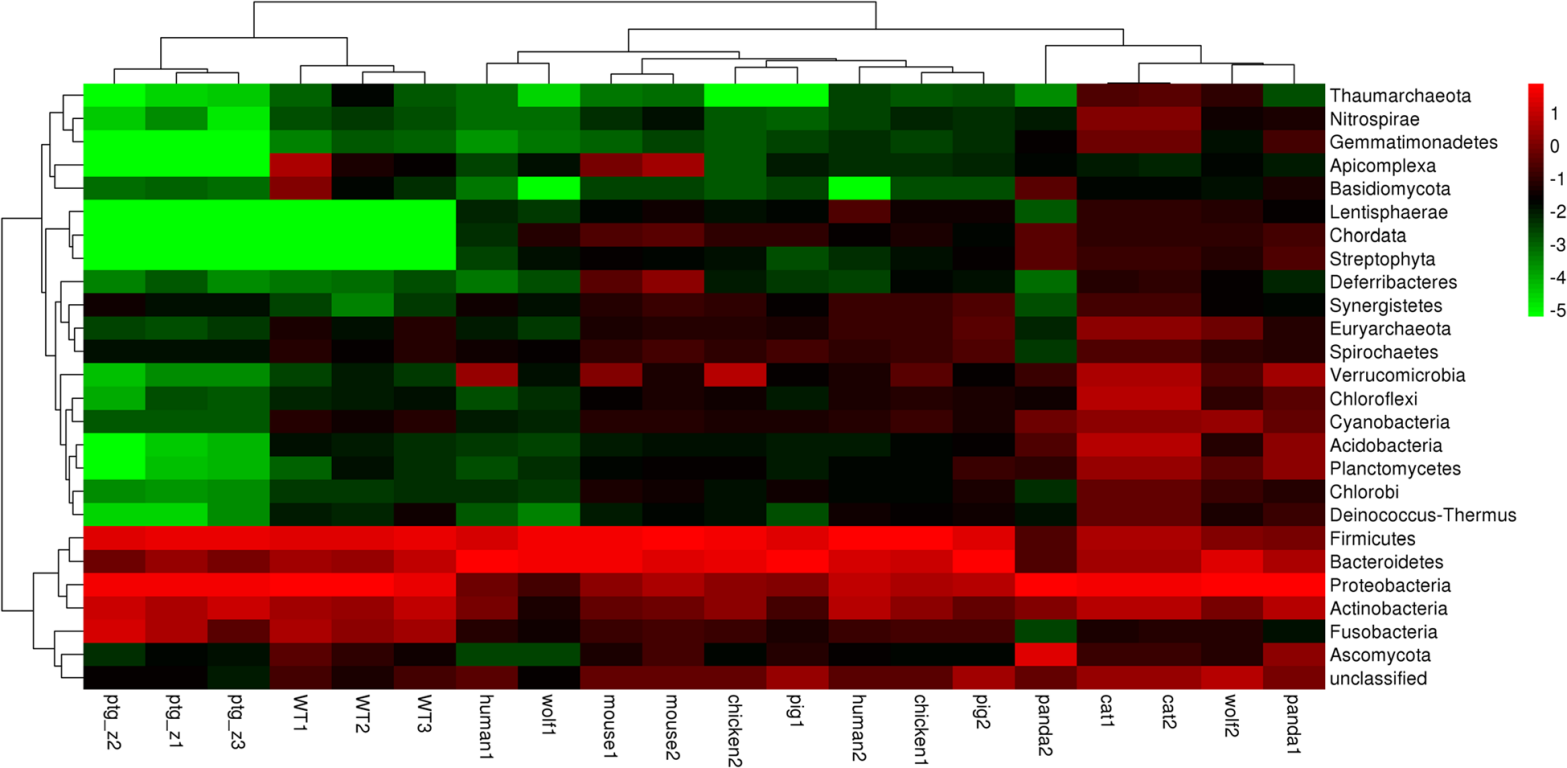
Heat map of phylogenetic clustering of the wild Amur tiger gut metagenome compared to those of other animals

**Fig. 4:**
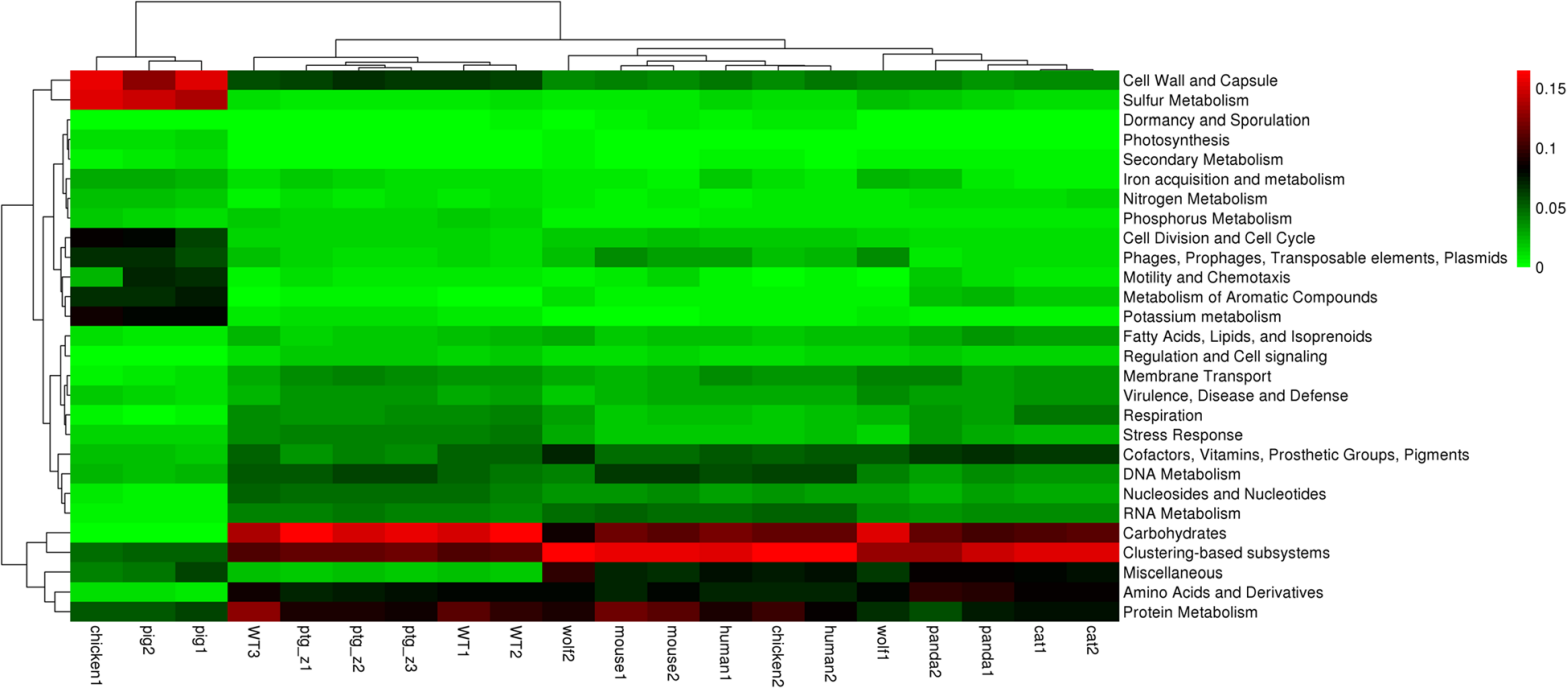
Heat map of metabolic clustering of the wild Amur tiger gut metagenome compared to those of other animals

The comparisons were performed at the phylogenetic (Fig. 3) and the metabolic levels (Fig. 4). In the phylogenetic comparison, Actinobacteria, Bacteroidetes, Firmicutes and Proteobacteria were the most abundant in all the samples, and the captive Amur tiger samples clustered with the wild Amur tiger samples. Interestingly, the abundance of Apicomplexa and Euryarchaeota were lower in the captive Amur tiger samples than in those from the wild Amur tigers. In addition, the heat map shows that the captive Amur tiger fecal metagenome had a lower abundance of Spirochaetes, Cyanobacteria and Ascomycota than other animals. These findings suggest that the characteristics of gut microbial diversity may adapt to the habits and diet of the Amur tiger in captivity. The metabolic comparison showed that the captive Amur tiger, wild Amur tiger, cat, panda, wolf and human samples clustered together and separated from those of pigs. As expected, all the gut metagenomes were dominated by carbohydrate metabolism subsystems, and the Protein Metabolism and Clustering-based subsystems were represented in relatively high abundance as well.

Intestinal microbial community structure is the result of the coevolution of the microbes and host animal and its environment, and intestinal microorganisms influence physiological function. The intestinal microbial community is also affected by the survival environment and feeding habit of the host animal [26]. Wild felids are susceptible to changes in metabolism and behaviour when kept in captivity; these changes are linked to differences from their natural habitat and feeding patterns [27–31].

## Conclusions

In this study, we present the use of whole-metagenome shotgun sequencing to analyze the composition and functional structures of the gut microbiota in captive Amur tigers. Our results showed a high abundance of four major phyla in captive Amur tigers, including Proteobacteria, Firmicutes, Actinobacteria and Fusobacteria. Moreover, at the genus level, *Escherichia*, *Collinsella* and *Fusobacterium* were most abundant in the captive Amur tiger fecal metagenome. At the species level, *Escherichia coli*, *Fusobacterium ulcerans* and *Fusobacterium varium* were the species with highest abundances in the captive Amur tiger gut microbiota. The primary functional categories of the Amur tiger faecal metagenome were associated mainly with Carbohydrate metabolism, Membrane transport and Amino acid metabolism based on the KEGG pathway database. The comparative metagenomic analyses showed that the captive Amur tiger fecal metagenome had a lower abundance of Spirochaetes, Cyanobacteria and Ascomycota than other animals, and the primary functional categories were primarily associated with carbohydrate metabolism subsystems, clustering-based subsystems and protein metabolism. Taken together, these findings reveal the composition and functional structures of the gut microbiota in captive Amur tiger.

## Methods

### Faecal sample collection

Fresh faecal samples from 3 Amur tigers were collected at a single time point from the Heilongjiang Siberian Tiger Park in Heilongjiang Province, China, with permission form the authorities of the Heilongjiang Siberian Tiger Park. The age, sex and diet fed in the 10 days prior to fecal sampling are shown in Table 1 and 2. We randomly selected healthy Amur tigers in the Siberian Tiger Park, and faecal samples were collected aseptically immediately after defecation. The fresh faecal samples were transported to the laboratory on dry ice within 24 h of collection and stored at − 80 °C until DNA extraction. We brought no toxic substance that would interfere with the animal habitats. The research complied with the protocols established by the China Wildlife Conservation Association and the legal requirements of China.

**Table 1:** Sex, Age and Physical condition of captive Amur tiger in Siberian Tiger Park

Sample	Sex	Age (year)	Physical condition
ptg_z1	Male	7	Healthy
ptg_z2	Male	7	Healthy
ptg_z3	Female	12	Healthy

**Table 2:** The diet of captive Amur tiger in the 10 days before the sampling in Siberian Tiger Park

Date	ptg_z1		ptg_z2		ptg_z3	
	Diet	Quantity (kg)	Diet	Quantity (kg)	Diet	Quantity (kg)
2017-6-10	Fasting	–	Fasting	–	Fasting	–
2017-6-11	Duck	3.5	Duck	3.5	Duck	3.5
2017-6-12	Duck	3.5	Duck	3.5	Duck	3.5
2017-6-13	Duck	3.5	Duck	3.5	Duck	3.5
2017-6-14	Fasting	–	Fasting	–	Fasting	–
2017-6-15	Duck	3.5	Duck	3.5	Duck	3.5
2017-6-16	Duck	3.5	Duck	3.5	Duck	3.5
2017-6-17	Fasting	–	Fasting	–	Fasting	–
2017-6-18	Duck	3.5	Duck	3.5	Duck	3.5
2017-6-19	Duck	3.5	Duck	3.5	Duck	3.5

### DNA extraction

DNA was extracted from the faecal samples using the E.Z.N.A.® Stool DNA Kit (D4015–02, Omega, Inc., USA) according to the manufacturer’s instructions. This reagent, which is designed to recover DNA from trace amounts of sample, has been shown to be effective for the preparation of DNA from most bacteria. Sample blanks consisted of unused swabs processed by DNA extraction and verified to contain no DNA amplicons. The total DNA was eluted in 50 μl of Elution buffer by modification of the procedure described by the manufacturer (QIAGEN) and stored at − 80 °C until measurement by PCR by LC-BIO TECHNOLOGIES (HANGZHOU) CO., LTD., Hang Zhou, Zhejiang Province, China.

### DNA library construction

The DNA library was constructed using a TruSeq Nano DNA LT Library Preparation Kit (FC-121-4001). DNA was fragmented by dsDNA Fragmentase (NEB, M0348S) with incubation at 37 °C for 30 min. Library construction began with fragmented cDNA. Blunt-end DNA fragments were generated using a combination of fill-in reactions and exonuclease activity, and size selection was performed with the provided sample purification beads. An A-base was then added to the blunt ends of each strand to prepare them for ligation to the indexed adapters. Each adapter contained a T-base overhang to ligate the adapter to the A-tailed fragmented DNA. These adapters contained the full complement of the sequencing primer hybridization sites for the single, paired-end, indexed reads. Single- or dual-index adapters were ligated to the fragments, and the ligated products were amplified by PCR under the following conditions: initial denaturation at 95 °C for 3 min; 8 cycles of denaturation at 98 °C for 15 s, annealing at 60 °C for 15 s, and extension at 72 °C for 30 s; and final extension at 37 °C for 5 min.

### Data analysis

Raw sequencing reads were processed to obtain valid reads for further analysis. First, sequencing adapters were removed from the sequencing reads using cutadapt v1.9. Second, low-quality reads were trimmed by fqtrim v0.94 using a sliding-window algorithm. Third, the reads were aligned to the host genome using bowtie2 v2.2.0 to remove host contamination. The quality-filtered reads were then de novo assembled to construct the metagenome for each sample by SPAdes v3.10.0. All coding regions (CDS) of the metagenomic contigs were predicted by MetaGeneMark v3.26.

The CDS sequences of all samples were clustered by CD-HIT v4.6.1 to obtain unigenes. The unigene abundance for a certain sample was estimated by TPM based on the number of aligned reads by bowtie2 v2.2.0. The lowest common ancestor taxonomy of the unigenes was obtained by aligning them against the NCBI NR database using DIAMOND v 0.7.12. Similarly, the functional annotation (GO, KEGG, CAZy) of the unigenes was obtained. Based on the taxonomic and functional annotations and abundance profiles of the unigenes, differential analysis was performed at the taxonomic, functional or gene level using Fisher’s exact test (non-replicated groups) or the Kruskal-Wallis test (replicated groups).

### Comparative metagenomic analysis

Comparative metagenomic analysis was performed using the MG-RAST pipelines. The metagenomic runs from the captive Amur tiger data were compared with the current publicly available gut metagenomes. In the MG-RAST metagenomic annotation pipeline, the captive Amur tiger fecal metagenomic datasets were compared with those of wild Amur tigers (WT1, WT2, and WT3) and fourteen public datasets from other animals, including cat 1 (mgm 4,626,753.3), cat 2 (mgm4626754.3), chicken 1 (mgm4743765.3), chicken 2 (mgm4440284.3), human 1 (mgm4472483.3), human 2 (mgm4440941.3), mouse 1 (mgm4537490.3), mouse 2 (mgm4540251.3), panda 1 (mgm4694746.3), panda 2 (mgm4683980.3), pig 1 (mgm4745745.3), pig 2 (mgm4745743.3), wolf 1 (mgm4535626.3) and wolf 2 (mgm4441601.3).

Integrate phylum level abundance information of captive Amur tiger and other species, standardize as a percentage, take the 25 phylum with the highest percentage, others are unclassified, take the log10 for heat map drawing (the value with a percentage of 0 is replaced by 1/2 of the minimum value in all data), heat map drawing using the pheatmap package. Download the subsystems database on the SEED website (http://www.theseed.org) and comparison to unigenes of captive Amur tiger. Next, calculate the abundance of each category of subsystems, integrate abundance information of Amur tigers and other species subsystems, standardized as a percentage, using the pheatmap package to draw the heat map.

## Additional files

Additional file 1: Information regarding the sequence data. (PDF 11 kb)

Additional file 2: Phylogenetic classification of the bacteria in the Amur tiger metagenome. (DOCX 537 kb)

Additional file 3: Phylogenetic classification of Eukaryota in the Amur tiger metagenome. (DOCX 22 kb)

Additional file 4: Phylogenetic classification of Archaea in the Amur tiger metagenome. (DOCX 21 kb)

Additional file 5: Phylogenetic classification of the viruses in the Amur tiger metagenome. (DOCX 43 kb)

Additional file 6: GO annotations of the Amur tiger faecal metagenome. (DOCX 683 kb)

Additional file 7: GO annotations of the Amur tiger faecal metagenome. (TIF 9899 kb)

Additional file 8: CAZy annotations of the Amur tiger metagenome. (DOCX 48 kb)

Additional file 9: CAZy classification of the Amur tiger faecal metagenome. (TIF 9289 kb)

## Abbreviations

CAZy: Carbohydrate-active enzymes
CBM: Carbohydratebinding modules
CITES: Convention on International Trade in Endangered Species of Wild Fauna and Flora
DNA: Deoxyribonucleic acid
GH: Glycoside hydrolase
GO: Gene ontology
GT: Glycosyl transferase
IUCN: International union for conservation of nature
KEGG: Kyoto encyclopedia of genes and genomes
NCBI: National center for biotechnology information
SRA: Short read archive
