# Supplementary material for: Metagenomic analysis of captive Amur tiger faecal microbiome
Source: BMC Vet Res. 2018 Dec 4;14:379. doi: 10.1186/s12917-018-1696-5 (PMC6278063; doi:10.1186/s12917-018-1696-5)
Supplement: Supplementary file 1 — Information regarding the sequence data. (PDF 11 kb) [file 12917_2018_1696_MOESM1_ESM.pdf]

Additional file 1 Information regarding the sequence data.

	Amur tiger metagenome
**Raw data**	
Total of reads	248,805,914
Total bp	37.32G
**Valid Data**	
Total of reads	229,366,156
Total bp	32.39G
Valid%	92.19%
Q20%	97.99
Q30%	94.06
GC%	43.84

Valid Data: cutadapt 1.9 remove sequencing connector; fqtrim 0.94 remove low-quality bases; FastQC 0.10.1 data quality control statistics; bowtie2 2.2.0 remove host pollution.
