# Supplementary material for: Metagenomic analysis of captive Amur tiger faecal microbiome
Source: BMC Vet Res. 2018 Dec 4;14:379. doi: 10.1186/s12917-018-1696-5 (PMC6278063; doi:10.1186/s12917-018-1696-5)
Supplement: Supplementary file 2 — Phylogenetic classification of the bacteria in the Amur tiger metagenome. (DOCX 537 kb) [file 12917_2018_1696_MOESM2_ESM.docx]

**Additional file 2: Phylogenetic classification of bacteria in the Amur tiger metagenome.**

| Phylum | Order | Genus | Species | ptg*  (%) |
| --- | --- | --- | --- | --- |
| Acidobacteria | Acidobacteria noname | Chloracidobacterium | Chloracidobacterium thermophilum | 0.00002 |
| Acidobacteria | Acidobacteriales | Acidobacteriaceae noname | Acidobacteriaceae bacterium TAA166 | 0.00001 |
| Acidobacteria | Acidobacteriales | Granulicella | Granulicella mallensis | 0.00001 |
| Actinobacteria | Acidimicrobiales | Acidithrix | Acidithrix ferrooxidans | 0.00003 |
| Actinobacteria | Acidimicrobiales | Ilumatobacter | Ilumatobacter nonamiensis | 0.00001 |
| Actinobacteria | Actinobacteria noname | Actinobacteria noname | actinobacterium acAMD-5 | 0.00006 |
| Actinobacteria | Actinobacteria noname | Actinobacteria noname | actinobacterium acMicro-1 | 0.00006 |
| Actinobacteria | Actinobacteria noname | Actinobacteria noname | actinobacterium LLX17 | 0.00004 |
| Actinobacteria | Actinobacteria noname | Actinobacteria noname | actinobacterium SCGC AAA027-M14 | 0.00002 |
| Actinobacteria | Actinobacteria noname | Actinobacteria noname | actinobacterium SCGC AAA028-A23 | 0.00006 |
| Actinobacteria | Actinobacteria noname | Actinobacteria noname | actinobacterium SCGC AAA041-L13 | 0.00201 |
| Actinobacteria | Actinobacteria noname | Actinobacteria noname | actinobacterium SCGC AAA044-D11 | 0.00005 |
| Actinobacteria | Actinobacteria noname | Actinobacteria noname | marine actinobacterium PHSC20C1 | 0.00006 |
| Actinobacteria | Actinobacteria noname | Candidatus Microthrix | Candidatus Microthrix parvicella | 0.00006 |
| Actinobacteria | Actinobacteria unclassified | Actinobacteria unclassified | Actinobacteria unclassified | 0.03669 |
| Actinobacteria | Actinomycetales | Actinobaculum | Actinobaculum massiliense | 0.00035 |
| Actinobacteria | Actinomycetales | Actinobaculum | Actinobaculum schaalii | 0.00082 |
| Actinobacteria | Actinomycetales | Actinobaculum | Actinobaculum sp. oral taxon 183 | 0.00053 |
| Actinobacteria | Actinomycetales | Actinobaculum | Actinobaculum urinale | 0.00035 |
| Actinobacteria | Actinomycetales | Actinomyces | Actinomyces cardiffensis | 0.05459 |
| Actinobacteria | Actinomycetales | Actinomyces | Actinomyces coleocanis | 0.00258 |
| Actinobacteria | Actinomycetales | Actinomyces | Actinomyces dentalis | 0.00091 |
| Actinobacteria | Actinomycetales | Actinomyces | Actinomyces europaeus | 0.00136 |
| Actinobacteria | Actinomycetales | Actinomyces | Actinomyces georgiae | 0.00207 |
| Actinobacteria | Actinomycetales | Actinomyces | Actinomyces gerencseriae | 0.00088 |
| Actinobacteria | Actinomycetales | Actinomyces | Actinomyces graevenitzii | 0.00133 |
| Actinobacteria | Actinomycetales | Actinomyces | Actinomyces israelii | 0.00064 |
| Actinobacteria | Actinomycetales | Actinomyces | Actinomyces johnsonii | 0.00113 |
| Actinobacteria | Actinomycetales | Actinomyces | Actinomyces massiliensis | 0.00107 |
| Actinobacteria | Actinomycetales | Actinomyces | Actinomyces naeslundii | 0.00024 |
| Actinobacteria | Actinomycetales | Actinomyces | Actinomyces neuii | 0.00258 |
| Actinobacteria | Actinomycetales | Actinomyces | Actinomyces odontolyticus | 0.00059 |
| Actinobacteria | Actinomycetales | Actinomyces | Actinomyces oris | 0.00021 |
| Actinobacteria | Actinomycetales | Actinomyces | Actinomyces slackii | 0.00071 |
| Actinobacteria | Actinomycetales | Actinomyces | Actinomyces sp. HPA0247 | 0.00150 |
| Actinobacteria | Actinomycetales | Actinomyces | Actinomyces sp. ICM39 | 0.00063 |
| Actinobacteria | Actinomycetales | Actinomyces | Actinomyces sp. ICM47 | 0.00067 |
| Actinobacteria | Actinomycetales | Actinomyces | Actinomyces sp. ICM54 | 0.00057 |
| Actinobacteria | Actinomycetales | Actinomyces | Actinomyces sp. MS2 | 0.00412 |
| Actinobacteria | Actinomycetales | Actinomyces | Actinomyces sp. oral taxon 170 | 0.00037 |
| Actinobacteria | Actinomycetales | Actinomyces | Actinomyces sp. oral taxon 171 | 0.00036 |
| Actinobacteria | Actinomycetales | Actinomyces | Actinomyces sp. oral taxon 172 | 0.00064 |
| Actinobacteria | Actinomycetales | Actinomyces | Actinomyces sp. oral taxon 175 | 0.00040 |
| Actinobacteria | Actinomycetales | Actinomyces | Actinomyces sp. oral taxon 178 | 0.00128 |
| Actinobacteria | Actinomycetales | Actinomyces | Actinomyces sp. oral taxon 180 | 0.00093 |
| Actinobacteria | Actinomycetales | Actinomyces | Actinomyces sp. oral taxon 181 | 0.00132 |
| Actinobacteria | Actinomycetales | Actinomyces | Actinomyces sp. oral taxon 448 | 0.00037 |
| Actinobacteria | Actinomycetales | Actinomyces | Actinomyces sp. oral taxon 848 | 0.00048 |
| Actinobacteria | Actinomycetales | Actinomyces | Actinomyces sp. oral taxon 849 | 0.00029 |
| Actinobacteria | Actinomycetales | Actinomyces | Actinomyces sp. oral taxon 877 | 0.00118 |
| Actinobacteria | Actinomycetales | Actinomyces | Actinomyces sp. ph3 | 0.00052 |
| Actinobacteria | Actinomycetales | Actinomyces | Actinomyces sp. S4-C9 | 0.00120 |
| Actinobacteria | Actinomycetales | Actinomyces | Actinomyces sp. S6-Spd3 | 0.00174 |
| Actinobacteria | Actinomycetales | Actinomyces | Actinomyces suimastitidis | 0.00564 |
| Actinobacteria | Actinomycetales | Actinomyces | Actinomyces timonensis | 0.00104 |
| Actinobacteria | Actinomycetales | Actinomyces | Actinomyces turicensis | 0.02842 |
| Actinobacteria | Actinomycetales | Actinomyces | Actinomyces unclassified | 0.00198 |
| Actinobacteria | Actinomycetales | Actinomyces | Actinomyces urogenitalis | 0.00347 |
| Actinobacteria | Actinomycetales | Actinomyces | Actinomyces vaccimaxillae | 0.01450 |
| Actinobacteria | Actinomycetales | Actinomyces | Actinomyces viscosus | 0.00042 |
| Actinobacteria | Actinomycetales | Actinomycetaceae unclassified | Actinomycetaceae unclassified | 0.00007 |
| Actinobacteria | Actinomycetales | Actinomycetales unclassified | Actinomycetales unclassified | 0.00004 |
| Actinobacteria | Actinomycetales | Arcanobacterium | Arcanobacterium haemolyticum | 0.00046 |
| Actinobacteria | Actinomycetales | Arcanobacterium | Arcanobacterium sp. S3PF19 | 0.00140 |
| Actinobacteria | Actinomycetales | Mobiluncus | Mobiluncus curtisii | 0.00657 |
| Actinobacteria | Actinomycetales | Mobiluncus | Mobiluncus mulieris | 0.00108 |
| Actinobacteria | Actinomycetales | Trueperella | Trueperella pyogenes | 0.00279 |
| Actinobacteria | Actinomycetales | Varibaculum | Varibaculum cambriense | 0.00865 |
| Actinobacteria | Bifidobacteriales | Alloscardovia | Alloscardovia criceti | 0.00115 |
| Actinobacteria | Bifidobacteriales | Alloscardovia | Alloscardovia omnicolens | 0.00271 |
| Actinobacteria | Bifidobacteriales | Bifidobacterium | Bifidobacterium actinocoloniiforme | 0.00081 |
| Actinobacteria | Bifidobacteriales | Bifidobacterium | Bifidobacterium adolescentis | 0.01418 |
| Actinobacteria | Bifidobacteriales | Bifidobacterium | Bifidobacterium adolescentis CAG:119 | 0.00011 |
| Actinobacteria | Bifidobacteriales | Bifidobacterium | Bifidobacterium angulatum | 0.00752 |
| Actinobacteria | Bifidobacteriales | Bifidobacterium | Bifidobacterium animalis | 0.00095 |
| Actinobacteria | Bifidobacteriales | Bifidobacterium | Bifidobacterium asteroides | 0.00107 |
| Actinobacteria | Bifidobacteriales | Bifidobacterium | Bifidobacterium biavatii | 0.00079 |
| Actinobacteria | Bifidobacteriales | Bifidobacterium | Bifidobacterium bifidum | 0.01022 |
| Actinobacteria | Bifidobacteriales | Bifidobacterium | Bifidobacterium bifidum CAG:234 | 0.00128 |
| Actinobacteria | Bifidobacteriales | Bifidobacterium | Bifidobacterium bohemicum | 0.00065 |
| Actinobacteria | Bifidobacteriales | Bifidobacterium | Bifidobacterium bombi | 0.00002 |
| Actinobacteria | Bifidobacteriales | Bifidobacterium | Bifidobacterium boum | 0.00009 |
| Actinobacteria | Bifidobacteriales | Bifidobacterium | Bifidobacterium breve | 0.02999 |
| Actinobacteria | Bifidobacteriales | Bifidobacterium | Bifidobacterium callitrichos | 0.00138 |
| Actinobacteria | Bifidobacteriales | Bifidobacterium | Bifidobacterium catenulatum | 0.00004 |
| Actinobacteria | Bifidobacteriales | Bifidobacterium | Bifidobacterium choerinum | 0.00004 |
| Actinobacteria | Bifidobacteriales | Bifidobacterium | Bifidobacterium coryneforme | 0.00097 |
| Actinobacteria | Bifidobacteriales | Bifidobacterium | Bifidobacterium crudilactis | 0.00005 |
| Actinobacteria | Bifidobacteriales | Bifidobacterium | Bifidobacterium cuniculi | 0.01673 |
| Actinobacteria | Bifidobacteriales | Bifidobacterium | Bifidobacterium dentium | 0.00239 |
| Actinobacteria | Bifidobacteriales | Bifidobacterium | Bifidobacterium gallicum | 0.00137 |
| Actinobacteria | Bifidobacteriales | Bifidobacterium | Bifidobacterium gallinarum | 0.00016 |
| Actinobacteria | Bifidobacteriales | Bifidobacterium | Bifidobacterium indicum | 0.00165 |
| Actinobacteria | Bifidobacteriales | Bifidobacterium | Bifidobacterium kashiwanohense | 0.00544 |
| Actinobacteria | Bifidobacteriales | Bifidobacterium | Bifidobacterium longum | 0.02040 |
| Actinobacteria | Bifidobacteriales | Bifidobacterium | Bifidobacterium magnum | 0.00010 |
| Actinobacteria | Bifidobacteriales | Bifidobacterium | Bifidobacterium merycicum | 0.00013 |
| Actinobacteria | Bifidobacteriales | Bifidobacterium | Bifidobacterium mongoliense | 0.00024 |
| Actinobacteria | Bifidobacteriales | Bifidobacterium | Bifidobacterium pseudocatenulatum | 0.00397 |
| Actinobacteria | Bifidobacteriales | Bifidobacterium | Bifidobacterium pseudolongum | 0.00492 |
| Actinobacteria | Bifidobacteriales | Bifidobacterium | Bifidobacterium psychraerophilum | 0.00005 |
| Actinobacteria | Bifidobacteriales | Bifidobacterium | Bifidobacterium pullorum | 0.00163 |
| Actinobacteria | Bifidobacteriales | Bifidobacterium | Bifidobacterium reuteri | 0.00447 |
| Actinobacteria | Bifidobacteriales | Bifidobacterium | Bifidobacterium ruminantium | 0.00198 |
| Actinobacteria | Bifidobacteriales | Bifidobacterium | Bifidobacterium saeculare | 0.01046 |
| Actinobacteria | Bifidobacteriales | Bifidobacterium | Bifidobacterium saguini | 0.00004 |
| Actinobacteria | Bifidobacteriales | Bifidobacterium | Bifidobacterium scardovii | 0.01577 |
| Actinobacteria | Bifidobacteriales | Bifidobacterium | Bifidobacterium sp. 12 1 47BFAA | 0.00075 |
| Actinobacteria | Bifidobacteriales | Bifidobacterium | Bifidobacterium sp. 7101 | 0.00006 |
| Actinobacteria | Bifidobacteriales | Bifidobacterium | Bifidobacterium sp. A11 | 0.00160 |
| Actinobacteria | Bifidobacteriales | Bifidobacterium | Bifidobacterium sp. MSTE12 | 0.00359 |
| Actinobacteria | Bifidobacteriales | Bifidobacterium | Bifidobacterium stellenboschense | 0.00138 |
| Actinobacteria | Bifidobacteriales | Bifidobacterium | Bifidobacterium stercoris | 0.00126 |
| Actinobacteria | Bifidobacteriales | Bifidobacterium | Bifidobacterium subtile | 0.00236 |
| Actinobacteria | Bifidobacteriales | Bifidobacterium | Bifidobacterium thermacidophilum | 0.00028 |
| Actinobacteria | Bifidobacteriales | Bifidobacterium | Bifidobacterium thermophilum | 0.00363 |
| Actinobacteria | Bifidobacteriales | Bifidobacterium | Bifidobacterium tsurumiense | 0.00195 |
| Actinobacteria | Bifidobacteriales | Bifidobacterium | Bifidobacterium unclassified | 0.00829 |
| Actinobacteria | Bifidobacteriales | Gardnerella | Gardnerella vaginalis | 0.00386 |
| Actinobacteria | Bifidobacteriales | Parascardovia | Parascardovia denticolens | 0.00003 |
| Actinobacteria | Bifidobacteriales | Scardovia | Scardovia inopinata | 0.00043 |
| Actinobacteria | Bifidobacteriales | Scardovia | Scardovia wiggsiae | 0.00003 |
| Actinobacteria | Catenulisporales | Actinospica | Actinospica robiniae | 0.00002 |
| Actinobacteria | Catenulisporales | Catenulispora | Catenulispora acidiphila | 0.00004 |
| Actinobacteria | Coriobacteriales | Atopobium | Atopobium fossor | 0.01727 |
| Actinobacteria | Coriobacteriales | Atopobium | Atopobium parvulum | 0.02598 |
| Actinobacteria | Coriobacteriales | Atopobium | Atopobium rimae | 0.02962 |
| Actinobacteria | Coriobacteriales | Atopobium | Atopobium sp. BS2 | 0.00125 |
| Actinobacteria | Coriobacteriales | Atopobium | Atopobium sp. BV3Ac4 | 0.00196 |
| Actinobacteria | Coriobacteriales | Atopobium | Atopobium sp. ICM42b | 0.00719 |
| Actinobacteria | Coriobacteriales | Atopobium | Atopobium sp. ICM58 | 0.00094 |
| Actinobacteria | Coriobacteriales | Atopobium | Atopobium sp. oral taxon 199 | 0.00653 |
| Actinobacteria | Coriobacteriales | Atopobium | Atopobium sp. oral taxon 810 | 0.01028 |
| Actinobacteria | Coriobacteriales | Atopobium | Atopobium unclassified | 0.00257 |
| Actinobacteria | Coriobacteriales | Atopobium | Atopobium vaginae | 0.00171 |
| Actinobacteria | Coriobacteriales | Collinsella | Collinsella aerofaciens | 0.47357 |
| Actinobacteria | Coriobacteriales | Collinsella | Collinsella intestinalis | 2.23062 |
| Actinobacteria | Coriobacteriales | Collinsella | Collinsella sp. 4 8 47FAA | 0.21142 |
| Actinobacteria | Coriobacteriales | Collinsella | Collinsella sp. CAG:166 | 0.12900 |
| Actinobacteria | Coriobacteriales | Collinsella | Collinsella sp. CAG:289 | 0.17631 |
| Actinobacteria | Coriobacteriales | Collinsella | Collinsella sp. CAG:398 | 0.11625 |
| Actinobacteria | Coriobacteriales | Collinsella | Collinsella sp. GD3 | 0.23498 |
| Actinobacteria | Coriobacteriales | Collinsella | Collinsella sp. MS5 | 0.16841 |
| Actinobacteria | Coriobacteriales | Collinsella | Collinsella stercoris | 2.50028 |
| Actinobacteria | Coriobacteriales | Collinsella | Collinsella tanakaei | 1.97113 |
| Actinobacteria | Coriobacteriales | Collinsella | Collinsella unclassified | 0.14955 |
| Actinobacteria | Coriobacteriales | Coriobacteriaceae noname | Coriobacteriaceae bacterium 68-1-3 | 0.03050 |
| Actinobacteria | Coriobacteriales | Coriobacteriaceae noname | Coriobacteriaceae bacterium BV3Ac1 | 0.00416 |
| Actinobacteria | Coriobacteriales | Coriobacteriaceae noname | Coriobacteriaceae bacterium GD5 | 0.00141 |
| Actinobacteria | Coriobacteriales | Coriobacteriaceae unclassified | Coriobacteriaceae unclassified | 0.10271 |
| Actinobacteria | Coriobacteriales | Coriobacterium | Coriobacterium glomerans | 0.08112 |
| Actinobacteria | Coriobacteriales | Enorma | Enorma massiliensis | 0.12472 |
| Actinobacteria | Coriobacteriales | Olsenella | Olsenella profusa | 0.09076 |
| Actinobacteria | Coriobacteriales | Olsenella | Olsenella sp. oral taxon 809 | 0.01292 |
| Actinobacteria | Coriobacteriales | Olsenella | Olsenella uli | 0.06003 |
| Actinobacteria | Coriobacteriales | Olsenella | Olsenella unclassified | 0.00054 |
| Actinobacteria | Coriobacteriales | Senegalimassilia | Senegalimassilia anaerobia | 0.02142 |
| Actinobacteria | Corynebacteriales | Corynebacteriaceae noname | Corynebacterium-like bacterium B27 | 0.00012 |
| Actinobacteria | Corynebacteriales | Corynebacterium | Corynebacterium accolens | 0.00015 |
| Actinobacteria | Corynebacteriales | Corynebacterium | Corynebacterium ammoniagenes | 0.00009 |
| Actinobacteria | Corynebacteriales | Corynebacterium | Corynebacterium amycolatum | 0.00016 |
| Actinobacteria | Corynebacteriales | Corynebacterium | Corynebacterium argentoratense | 0.00056 |
| Actinobacteria | Corynebacteriales | Corynebacterium | Corynebacterium atypicum | 0.00045 |
| Actinobacteria | Corynebacteriales | Corynebacterium | Corynebacterium aurimucosum | 0.00023 |
| Actinobacteria | Corynebacteriales | Corynebacterium | Corynebacterium auriscanis | 0.00010 |
| Actinobacteria | Corynebacteriales | Corynebacterium | Corynebacterium bovis | 0.00011 |
| Actinobacteria | Corynebacteriales | Corynebacterium | Corynebacterium callunae | 0.00044 |
| Actinobacteria | Corynebacteriales | Corynebacterium | Corynebacterium camporealensis | 0.00017 |
| Actinobacteria | Corynebacteriales | Corynebacterium | Corynebacterium capitovis | 0.00010 |
| Actinobacteria | Corynebacteriales | Corynebacterium | Corynebacterium casei | 0.00062 |
| Actinobacteria | Corynebacteriales | Corynebacterium | Corynebacterium caspium | 0.00018 |
| Actinobacteria | Corynebacteriales | Corynebacterium | Corynebacterium ciconiae | 0.00050 |
| Actinobacteria | Corynebacteriales | Corynebacterium | Corynebacterium crenatum | 0.00002 |
| Actinobacteria | Corynebacteriales | Corynebacterium | Corynebacterium diphtheriae | 0.05326 |
| Actinobacteria | Corynebacteriales | Corynebacterium | Corynebacterium doosanense | 0.00014 |
| Actinobacteria | Corynebacteriales | Corynebacterium | Corynebacterium durum | 0.00125 |
| Actinobacteria | Corynebacteriales | Corynebacterium | Corynebacterium efficiens | 0.00105 |
| Actinobacteria | Corynebacteriales | Corynebacterium | Corynebacterium falsenii | 0.00081 |
| Actinobacteria | Corynebacteriales | Corynebacterium | Corynebacterium felinum | 0.00006 |
| Actinobacteria | Corynebacteriales | Corynebacterium | Corynebacterium freiburgense | 0.00184 |
| Actinobacteria | Corynebacteriales | Corynebacterium | Corynebacterium freneyi | 0.00019 |
| Actinobacteria | Corynebacteriales | Corynebacterium | Corynebacterium genitalium | 0.00023 |
| Actinobacteria | Corynebacteriales | Corynebacterium | Corynebacterium glucuronolyticum | 0.00010 |
| Actinobacteria | Corynebacteriales | Corynebacterium | Corynebacterium glutamicum | 0.00130 |
| Actinobacteria | Corynebacteriales | Corynebacterium | Corynebacterium glyciniphilum | 0.00004 |
| Actinobacteria | Corynebacteriales | Corynebacterium | Corynebacterium halotolerans | 0.00081 |
| Actinobacteria | Corynebacteriales | Corynebacterium | Corynebacterium humireducens | 0.00044 |
| Actinobacteria | Corynebacteriales | Corynebacterium | Corynebacterium imitans | 0.00011 |
| Actinobacteria | Corynebacteriales | Corynebacterium | Corynebacterium jeikeium | 0.00073 |
| Actinobacteria | Corynebacteriales | Corynebacterium | Corynebacterium kroppenstedtii | 0.00002 |
| Actinobacteria | Corynebacteriales | Corynebacterium | Corynebacterium lipophiloflavum | 0.00026 |
| Actinobacteria | Corynebacteriales | Corynebacterium | Corynebacterium lubricantis | 0.00027 |
| Actinobacteria | Corynebacteriales | Corynebacterium | Corynebacterium marinum | 0.00093 |
| Actinobacteria | Corynebacteriales | Corynebacterium | Corynebacterium maris | 0.00019 |
| Actinobacteria | Corynebacteriales | Corynebacterium | Corynebacterium massiliense | 0.00003 |
| Actinobacteria | Corynebacteriales | Corynebacterium | Corynebacterium mastitidis | 0.00011 |
| Actinobacteria | Corynebacteriales | Corynebacterium | Corynebacterium matruchotii | 0.01321 |
| Actinobacteria | Corynebacteriales | Corynebacterium | Corynebacterium minutissimum | 0.00020 |
| Actinobacteria | Corynebacteriales | Corynebacterium | Corynebacterium nuruki | 0.00010 |
| Actinobacteria | Corynebacteriales | Corynebacterium | Corynebacterium pilosum | 0.00011 |
| Actinobacteria | Corynebacteriales | Corynebacterium | Corynebacterium propinquum | 0.00011 |
| Actinobacteria | Corynebacteriales | Corynebacterium | Corynebacterium pseudodiphtheriticum | 0.00007 |
| Actinobacteria | Corynebacteriales | Corynebacterium | Corynebacterium pseudogenitalium | 0.00008 |
| Actinobacteria | Corynebacteriales | Corynebacterium | Corynebacterium pseudotuberculosis | 0.00199 |
| Actinobacteria | Corynebacteriales | Corynebacterium | Corynebacterium pyruviciproducens | 0.00032 |
| Actinobacteria | Corynebacteriales | Corynebacterium | Corynebacterium resistens | 0.00035 |
| Actinobacteria | Corynebacteriales | Corynebacterium | Corynebacterium singulare | 0.00015 |
| Actinobacteria | Corynebacteriales | Corynebacterium | Corynebacterium sp. ATCC 6931 | 0.00013 |
| Actinobacteria | Corynebacteriales | Corynebacterium | Corynebacterium sp. GD7 | 0.00001 |
| Actinobacteria | Corynebacteriales | Corynebacterium | Corynebacterium sp. HFH0082 | 0.00002 |
| Actinobacteria | Corynebacteriales | Corynebacterium | Corynebacterium sp. JCB | 0.00004 |
| Actinobacteria | Corynebacteriales | Corynebacterium | Corynebacterium sp. KPL1818 | 0.00003 |
| Actinobacteria | Corynebacteriales | Corynebacterium | Corynebacterium sp. KPL1824 | 0.00002 |
| Actinobacteria | Corynebacteriales | Corynebacterium | Corynebacterium sp. KPL1856 | 0.00001 |
| Actinobacteria | Corynebacteriales | Corynebacterium | Corynebacterium sp. KPL1859 | 0.00002 |
| Actinobacteria | Corynebacteriales | Corynebacterium | Corynebacterium sp. KPL1986 | 0.00019 |
| Actinobacteria | Corynebacteriales | Corynebacterium | Corynebacterium sp. KPL1996 | 0.00003 |
| Actinobacteria | Corynebacteriales | Corynebacterium | Corynebacterium sp. L2-79-05 | 0.00020 |
| Actinobacteria | Corynebacteriales | Corynebacterium | Corynebacterium sputi | 0.00036 |
| Actinobacteria | Corynebacteriales | Corynebacterium | Corynebacterium striatum | 0.00023 |
| Actinobacteria | Corynebacteriales | Corynebacterium | Corynebacterium timonense | 0.00003 |
| Actinobacteria | Corynebacteriales | Corynebacterium | Corynebacterium tuberculostearicum | 0.00003 |
| Actinobacteria | Corynebacteriales | Corynebacterium | Corynebacterium tuscaniense | 0.00006 |
| Actinobacteria | Corynebacteriales | Corynebacterium | Corynebacterium ulcerans | 0.00911 |
| Actinobacteria | Corynebacteriales | Corynebacterium | Corynebacterium ulceribovis | 0.00012 |
| Actinobacteria | Corynebacteriales | Corynebacterium | Corynebacterium unclassified | 0.00085 |
| Actinobacteria | Corynebacteriales | Corynebacterium | Corynebacterium urealyticum | 0.00016 |
| Actinobacteria | Corynebacteriales | Corynebacterium | Corynebacterium ureicelerivorans | 0.00004 |
| Actinobacteria | Corynebacteriales | Corynebacterium | Corynebacterium variabile | 0.00006 |
| Actinobacteria | Corynebacteriales | Corynebacterium | Corynebacterium vitaeruminis | 0.00843 |
| Actinobacteria | Corynebacteriales | Dietzia | Dietzia alimentaria | 0.00017 |
| Actinobacteria | Corynebacteriales | Dietzia | Dietzia cinnamea | 0.00002 |
| Actinobacteria | Corynebacteriales | Gordonia | Gordonia aichiensis | 0.00004 |
| Actinobacteria | Corynebacteriales | Gordonia | Gordonia alkanivorans | 0.00041 |
| Actinobacteria | Corynebacteriales | Gordonia | Gordonia amarae | 0.00014 |
| Actinobacteria | Corynebacteriales | Gordonia | Gordonia araii | 0.00008 |
| Actinobacteria | Corynebacteriales | Gordonia | Gordonia bronchialis | 0.00005 |
| Actinobacteria | Corynebacteriales | Gordonia | Gordonia hirsuta | 0.00013 |
| Actinobacteria | Corynebacteriales | Gordonia | Gordonia malaquae | 0.00005 |
| Actinobacteria | Corynebacteriales | Gordonia | Gordonia paraffinivorans | 0.00008 |
| Actinobacteria | Corynebacteriales | Gordonia | Gordonia shandongensis | 0.00006 |
| Actinobacteria | Corynebacteriales | Gordonia | Gordonia sp. NB4-1Y | 0.00002 |
| Actinobacteria | Corynebacteriales | Gordonia | Gordonia sp. no. 9 | 0.00002 |
| Actinobacteria | Corynebacteriales | Mycobacterium | Mycobacterium austroafricanum | 0.00003 |
| Actinobacteria | Corynebacteriales | Mycobacterium | Mycobacterium avium | 0.00001 |
| Actinobacteria | Corynebacteriales | Mycobacterium | Mycobacterium cosmeticum | 0.00005 |
| Actinobacteria | Corynebacteriales | Mycobacterium | Mycobacterium gilvum | 0.00022 |
| Actinobacteria | Corynebacteriales | Mycobacterium | Mycobacterium hassiacum | 0.00003 |
| Actinobacteria | Corynebacteriales | Mycobacterium | Mycobacterium iranicum | 0.00003 |
| Actinobacteria | Corynebacteriales | Mycobacterium | Mycobacterium mageritense | 0.00001 |
| Actinobacteria | Corynebacteriales | Mycobacterium | Mycobacterium marinum | 0.00011 |
| Actinobacteria | Corynebacteriales | Mycobacterium | Mycobacterium parascrofulaceum | 0.00003 |
| Actinobacteria | Corynebacteriales | Mycobacterium | Mycobacterium sp. UM WGJ | 0.00002 |
| Actinobacteria | Corynebacteriales | Mycobacterium | Mycobacterium sp. UNC280MFTsu5.1 | 0.00004 |
| Actinobacteria | Corynebacteriales | Mycobacterium | Mycobacterium sp. UNC410CL29Cvi84 | 0.00002 |
| Actinobacteria | Corynebacteriales | Mycobacterium | Mycobacterium thermoresistibile | 0.00002 |
| Actinobacteria | Corynebacteriales | Mycobacterium | Mycobacterium tuberculosis | 0.00108 |
| Actinobacteria | Corynebacteriales | Mycobacterium | Mycobacterium unclassified | 0.00005 |
| Actinobacteria | Corynebacteriales | Mycobacterium | Mycobacterium xenopi | 0.00017 |
| Actinobacteria | Corynebacteriales | Nocardia | Nocardia abscessus | 0.00003 |
| Actinobacteria | Corynebacteriales | Nocardia | Nocardia araoensis | 0.00003 |
| Actinobacteria | Corynebacteriales | Nocardia | Nocardia asiatica | 0.00001 |
| Actinobacteria | Corynebacteriales | Nocardia | Nocardia concava | 0.00003 |
| Actinobacteria | Corynebacteriales | Nocardia | Nocardia higoensis | 0.00003 |
| Actinobacteria | Corynebacteriales | Nocardia | Nocardia otitidiscaviarum | 0.00002 |
| Actinobacteria | Corynebacteriales | Nocardia | Nocardia thailandica | 0.00002 |
| Actinobacteria | Corynebacteriales | Nocardia | Nocardia veterana | 0.00002 |
| Actinobacteria | Corynebacteriales | Nocardia | Nocardia vinacea | 0.00003 |
| Actinobacteria | Corynebacteriales | Rhodococcus | Rhodococcus defluvii | 0.00004 |
| Actinobacteria | Corynebacteriales | Rhodococcus | Rhodococcus erythropolis | 0.00004 |
| Actinobacteria | Corynebacteriales | Rhodococcus | Rhodococcus fascians | 0.00003 |
| Actinobacteria | Corynebacteriales | Rhodococcus | Rhodococcus opacus | 0.00002 |
| Actinobacteria | Corynebacteriales | Rhodococcus | Rhodococcus pyridinivorans | 0.00005 |
| Actinobacteria | Corynebacteriales | Rhodococcus | Rhodococcus rhodnii | 0.00006 |
| Actinobacteria | Corynebacteriales | Rhodococcus | Rhodococcus rhodochrous | 0.00005 |
| Actinobacteria | Corynebacteriales | Rhodococcus | Rhodococcus sp. DK17 | 0.00003 |
| Actinobacteria | Corynebacteriales | Rhodococcus | Rhodococcus sp. R1101 | 0.00003 |
| Actinobacteria | Corynebacteriales | Rhodococcus | Rhodococcus unclassified | 0.00002 |
| Actinobacteria | Eggerthellales | Adlercreutzia | Adlercreutzia equolifaciens | 0.02725 |
| Actinobacteria | Eggerthellales | Cryptobacterium | Cryptobacterium curtum | 0.00230 |
| Actinobacteria | Eggerthellales | Cryptobacterium | Cryptobacterium sp. CAG:338 | 0.00196 |
| Actinobacteria | Eggerthellales | Eggerthella | Eggerthella lenta | 0.01447 |
| Actinobacteria | Eggerthellales | Eggerthella | Eggerthella sp. 1 3 56FAA | 0.00050 |
| Actinobacteria | Eggerthellales | Eggerthella | Eggerthella sp. CAG:1427 | 0.00107 |
| Actinobacteria | Eggerthellales | Eggerthella | Eggerthella sp. CAG:209 | 0.00516 |
| Actinobacteria | Eggerthellales | Eggerthella | Eggerthella sp. CAG:298 | 0.00068 |
| Actinobacteria | Eggerthellales | Eggerthella | Eggerthella sp. CAG:368 | 0.03876 |
| Actinobacteria | Eggerthellales | Eggerthella | Eggerthella sp. HGA1 | 0.00543 |
| Actinobacteria | Eggerthellales | Eggerthella | Eggerthella sp. YY7918 | 0.02264 |
| Actinobacteria | Eggerthellales | Eggerthella | Eggerthella unclassified | 0.00944 |
| Actinobacteria | Eggerthellales | Enterorhabdus | Enterorhabdus caecimuris | 0.03685 |
| Actinobacteria | Eggerthellales | Enterorhabdus | Enterorhabdus mucosicola | 0.00217 |
| Actinobacteria | Eggerthellales | Enterorhabdus | Enterorhabdus unclassified | 0.00009 |
| Actinobacteria | Eggerthellales | Gordonibacter | Gordonibacter pamelaeae | 0.01926 |
| Actinobacteria | Eggerthellales | Slackia | Slackia exigua | 0.00096 |
| Actinobacteria | Eggerthellales | Slackia | Slackia heliotrinireducens | 0.04383 |
| Actinobacteria | Eggerthellales | Slackia | Slackia piriformis | 0.11831 |
| Actinobacteria | Eggerthellales | Slackia | Slackia sp. CM382 | 0.00038 |
| Actinobacteria | Eggerthellales | Slackia | Slackia unclassified | 0.00036 |
| Actinobacteria | Frankiales | Cryptosporangium | Cryptosporangium arvum | 0.00002 |
| Actinobacteria | Frankiales | Frankia | Frankia sp. CN3 | 0.00005 |
| Actinobacteria | Frankiales | Frankia | Frankia sp. EAN1pec | 0.00001 |
| Actinobacteria | Frankiales | Frankia | Frankia sp. EuI1c | 0.00001 |
| Actinobacteria | Frankiales | Frankia | Frankia sp. EUN1f | 0.00002 |
| Actinobacteria | Frankiales | Frankia | Frankia sp. Iso899 | 0.00002 |
| Actinobacteria | Frankiales | Frankia | Frankia symbiont of Datisca glomerata | 0.00037 |
| Actinobacteria | Frankiales | Sporichthya | Sporichthya polymorpha | 0.00009 |
| Actinobacteria | Geodermatophilales | Geodermatophilaceae noname | Geodermatophilaceae bacterium URHB0048 | 0.00001 |
| Actinobacteria | Geodermatophilales | Geodermatophilaceae noname | Geodermatophilaceae bacterium URHB0062 | 0.00002 |
| Actinobacteria | Geodermatophilales | Modestobacter | Modestobacter sp. KNN45-2b | 0.00004 |
| Actinobacteria | Glycomycetales | Haloglycomyces | Haloglycomyces albus | 0.00001 |
| Actinobacteria | Glycomycetales | Stackebrandtia | Stackebrandtia nassauensis | 0.00003 |
| Actinobacteria | Jiangellales | Jiangella | Jiangella gansuensis | 0.00002 |
| Actinobacteria | Kineosporiales | Kineococcus | Kineococcus radiotolerans | 0.00008 |
| Actinobacteria | Micrococcales | Acaricomes | Acaricomes phytoseiuli | 0.00004 |
| Actinobacteria | Micrococcales | Actinotalea | Actinotalea fermentans | 0.00037 |
| Actinobacteria | Micrococcales | Actinotalea | Actinotalea ferrariae | 0.00024 |
| Actinobacteria | Micrococcales | Agreia | Agreia bicolorata | 0.00012 |
| Actinobacteria | Micrococcales | Agrococcus | Agrococcus lahaulensis | 0.00023 |
| Actinobacteria | Micrococcales | Agrococcus | Agrococcus pavilionensis | 0.00010 |
| Actinobacteria | Micrococcales | Agromyces | Agromyces italicus | 0.00027 |
| Actinobacteria | Micrococcales | Agromyces | Agromyces sp. KY5R | 0.00002 |
| Actinobacteria | Micrococcales | Agromyces | Agromyces subbeticus | 0.00015 |
| Actinobacteria | Micrococcales | Arsenicicoccus | Arsenicicoccus bolidensis | 0.00002 |
| Actinobacteria | Micrococcales | Arthrobacter | Arthrobacter arilaitensis | 0.00010 |
| Actinobacteria | Micrococcales | Arthrobacter | Arthrobacter castelli | 0.00008 |
| Actinobacteria | Micrococcales | Arthrobacter | Arthrobacter chlorophenolicus | 0.00004 |
| Actinobacteria | Micrococcales | Arthrobacter | Arthrobacter crystallopoietes | 0.00004 |
| Actinobacteria | Micrococcales | Arthrobacter | Arthrobacter gangotriensis | 0.00041 |
| Actinobacteria | Micrococcales | Arthrobacter | Arthrobacter globiformis | 0.00006 |
| Actinobacteria | Micrococcales | Arthrobacter | Arthrobacter nicotinovorans | 0.00005 |
| Actinobacteria | Micrococcales | Arthrobacter | Arthrobacter nitrophenolicus | 0.00004 |
| Actinobacteria | Micrococcales | Arthrobacter | Arthrobacter phenanthrenivorans | 0.00005 |
| Actinobacteria | Micrococcales | Arthrobacter | Arthrobacter sanguinis | 0.00008 |
| Actinobacteria | Micrococcales | Arthrobacter | Arthrobacter siccitolerans | 0.00002 |
| Actinobacteria | Micrococcales | Arthrobacter | Arthrobacter sp. 11W110 air | 0.00012 |
| Actinobacteria | Micrococcales | Arthrobacter | Arthrobacter sp. 135MFCol5.1 | 0.00003 |
| Actinobacteria | Micrococcales | Arthrobacter | Arthrobacter sp. 162MFSha1.1 | 0.00002 |
| Actinobacteria | Micrococcales | Arthrobacter | Arthrobacter sp. 31Y | 0.00007 |
| Actinobacteria | Micrococcales | Arthrobacter | Arthrobacter sp. 35W | 0.00019 |
| Actinobacteria | Micrococcales | Arthrobacter | Arthrobacter sp. A3 | 0.00004 |
| Actinobacteria | Micrococcales | Arthrobacter | Arthrobacter sp. AK-YN10 | 0.00003 |
| Actinobacteria | Micrococcales | Arthrobacter | Arthrobacter sp. Br18 | 0.00001 |
| Actinobacteria | Micrococcales | Arthrobacter | Arthrobacter sp. CAL618 | 0.00002 |
| Actinobacteria | Micrococcales | Arthrobacter | Arthrobacter sp. FB24 | 0.00006 |
| Actinobacteria | Micrococcales | Arthrobacter | Arthrobacter sp. H14 | 0.00002 |
| Actinobacteria | Micrococcales | Arthrobacter | Arthrobacter sp. H20 | 0.00003 |
| Actinobacteria | Micrococcales | Arthrobacter | Arthrobacter sp. H5 | 0.00004 |
| Actinobacteria | Micrococcales | Arthrobacter | Arthrobacter sp. I3 | 0.00011 |
| Actinobacteria | Micrococcales | Arthrobacter | Arthrobacter sp. IHBB 11108 | 0.00004 |
| Actinobacteria | Micrococcales | Arthrobacter | Arthrobacter sp. L77 | 0.00009 |
| Actinobacteria | Micrococcales | Arthrobacter | Arthrobacter sp. M2012083 | 0.00001 |
| Actinobacteria | Micrococcales | Arthrobacter | Arthrobacter sp. MA-N2 | 0.00004 |
| Actinobacteria | Micrococcales | Arthrobacter | Arthrobacter sp. PAMC25486 | 0.00002 |
| Actinobacteria | Micrococcales | Arthrobacter | Arthrobacter sp. PAO19 | 0.00016 |
| Actinobacteria | Micrococcales | Arthrobacter | Arthrobacter sp. SPG23 | 0.00003 |
| Actinobacteria | Micrococcales | Arthrobacter | Arthrobacter sp. TB 23 | 0.00001 |
| Actinobacteria | Micrococcales | Arthrobacter | Arthrobacter sp. TB 26 | 0.00002 |
| Actinobacteria | Micrococcales | Arthrobacter | Arthrobacter sp. W1 | 0.00002 |
| Actinobacteria | Micrococcales | Arthrobacter | Arthrobacter unclassified | 0.00004 |
| Actinobacteria | Micrococcales | Austwickia | Austwickia chelonae | 0.00020 |
| Actinobacteria | Micrococcales | Beutenbergia | Beutenbergia cavernae | 0.00054 |
| Actinobacteria | Micrococcales | Brachybacterium | Brachybacterium faecium | 0.00011 |
| Actinobacteria | Micrococcales | Brachybacterium | Brachybacterium muris | 0.00007 |
| Actinobacteria | Micrococcales | Brachybacterium | Brachybacterium paraconglomeratum | 0.00002 |
| Actinobacteria | Micrococcales | Brachybacterium | Brachybacterium phenoliresistens | 0.00014 |
| Actinobacteria | Micrococcales | Brachybacterium | Brachybacterium squillarum | 0.00006 |
| Actinobacteria | Micrococcales | Brevibacterium | Brevibacterium album | 0.00018 |
| Actinobacteria | Micrococcales | Brevibacterium | Brevibacterium casei | 0.00007 |
| Actinobacteria | Micrococcales | Brevibacterium | Brevibacterium linens | 0.00020 |
| Actinobacteria | Micrococcales | Brevibacterium | Brevibacterium massiliense | 0.00023 |
| Actinobacteria | Micrococcales | Brevibacterium | Brevibacterium mcbrellneri | 0.00040 |
| Actinobacteria | Micrococcales | Brevibacterium | Brevibacterium sp. VCM10 | 0.00006 |
| Actinobacteria | Micrococcales | Cellulomonas | [Cellvibrio] gilvus | 0.00011 |
| Actinobacteria | Micrococcales | Cellulomonas | Cellulomonas bogoriensis | 0.00005 |
| Actinobacteria | Micrococcales | Cellulomonas | Cellulomonas carbonis | 0.00022 |
| Actinobacteria | Micrococcales | Cellulomonas | Cellulomonas cellasea | 0.00014 |
| Actinobacteria | Micrococcales | Cellulomonas | Cellulomonas fimi | 0.00017 |
| Actinobacteria | Micrococcales | Cellulomonas | Cellulomonas flavigena | 0.00017 |
| Actinobacteria | Micrococcales | Cellulomonas | Cellulomonas massiliensis | 0.00029 |
| Actinobacteria | Micrococcales | Cellulomonas | Cellulomonas sp. HZM | 0.00013 |
| Actinobacteria | Micrococcales | Cellulomonas | Cellulomonas sp. KRMCY2 | 0.00016 |
| Actinobacteria | Micrococcales | Cellulomonas | Cellulomonas sp. URHD0024 | 0.00009 |
| Actinobacteria | Micrococcales | Cellulomonas | Cellulomonas sp. URHE0023 | 0.00008 |
| Actinobacteria | Micrococcales | Cellulomonas | Cellulomonas unclassified | 0.00004 |
| Actinobacteria | Micrococcales | Cellulosimicrobium | Cellulosimicrobium cellulans | 0.00040 |
| Actinobacteria | Micrococcales | Cellulosimicrobium | Cellulosimicrobium sp. MM | 0.00009 |
| Actinobacteria | Micrococcales | Cellulosimicrobium | Cellulosimicrobium unclassified | 0.00004 |
| Actinobacteria | Micrococcales | Citricoccus | Citricoccus sp. CH26A | 0.00022 |
| Actinobacteria | Micrococcales | Clavibacter | Clavibacter michiganensis | 0.00020 |
| Actinobacteria | Micrococcales | Cryobacterium | Cryobacterium roopkundense | 0.00005 |
| Actinobacteria | Micrococcales | Cryobacterium | Cryobacterium sp. MLB-32 | 0.00005 |
| Actinobacteria | Micrococcales | Cryocola | Cryocola sp. 340MFSha3.1 | 0.00006 |
| Actinobacteria | Micrococcales | Curtobacterium | Curtobacterium flaccumfaciens | 0.00006 |
| Actinobacteria | Micrococcales | Curtobacterium | Curtobacterium sp. B18 | 0.00009 |
| Actinobacteria | Micrococcales | Curtobacterium | Curtobacterium sp. B8 | 0.00008 |
| Actinobacteria | Micrococcales | Curtobacterium | Curtobacterium sp. S6 | 0.00019 |
| Actinobacteria | Micrococcales | Curtobacterium | Curtobacterium sp. UNCCL17 | 0.00005 |
| Actinobacteria | Micrococcales | Dermabacter | Dermabacter hominis | 0.00028 |
| Actinobacteria | Micrococcales | Dermabacter | Dermabacter sp. HFH0086 | 0.00013 |
| Actinobacteria | Micrococcales | Dermabacter | Dermabacter unclassified | 0.00004 |
| Actinobacteria | Micrococcales | Dermacoccus | Dermacoccus sp. Ellin185 | 0.00007 |
| Actinobacteria | Micrococcales | Dermatophilus | Dermatophilus congolensis | 0.00005 |
| Actinobacteria | Micrococcales | Frigoribacterium | Frigoribacterium sp. MEB024 | 0.00008 |
| Actinobacteria | Micrococcales | Georgenia | Georgenia sp. SUBG003 | 0.00277 |
| Actinobacteria | Micrococcales | Glaciibacter | Glaciibacter superstes | 0.00006 |
| Actinobacteria | Micrococcales | Gryllotalpicola | Gryllotalpicola ginsengisoli | 0.00011 |
| Actinobacteria | Micrococcales | Gulosibacter | Gulosibacter molinativorax | 0.00028 |
| Actinobacteria | Micrococcales | Humibacter | Humibacter albus | 0.00016 |
| Actinobacteria | Micrococcales | Intrasporangium | Intrasporangium calvum | 0.00002 |
| Actinobacteria | Micrococcales | Intrasporangium | Intrasporangium chromatireducens | 0.00004 |
| Actinobacteria | Micrococcales | Intrasporangium | Intrasporangium oryzae | 0.00010 |
| Actinobacteria | Micrococcales | Isoptericola | Isoptericola variabilis | 0.00026 |
| Actinobacteria | Micrococcales | Janibacter | Janibacter hoylei | 0.00005 |
| Actinobacteria | Micrococcales | Janibacter | Janibacter sp. HTCC2649 | 0.00003 |
| Actinobacteria | Micrococcales | Jonesia | Jonesia denitrificans | 0.00022 |
| Actinobacteria | Micrococcales | Jonesia | Jonesia quinghaiensis | 0.00011 |
| Actinobacteria | Micrococcales | Kineosphaera | Kineosphaera limosa | 0.00020 |
| Actinobacteria | Micrococcales | Knoellia | Knoellia flava | 0.00003 |
| Actinobacteria | Micrococcales | Knoellia | Knoellia subterranea | 0.00007 |
| Actinobacteria | Micrococcales | Kocuria | Kocuria marina | 0.00001 |
| Actinobacteria | Micrococcales | Kocuria | Kocuria palustris | 0.00012 |
| Actinobacteria | Micrococcales | Kocuria | Kocuria polaris | 0.00011 |
| Actinobacteria | Micrococcales | Kocuria | Kocuria rhizophila | 0.00005 |
| Actinobacteria | Micrococcales | Kocuria | Kocuria unclassified | 0.00003 |
| Actinobacteria | Micrococcales | Kocuria | Kocuria varians | 0.00004 |
| Actinobacteria | Micrococcales | Kytococcus | Kytococcus sedentarius | 0.00013 |
| Actinobacteria | Micrococcales | Leifsonia | Leifsonia aquatica | 0.00021 |
| Actinobacteria | Micrococcales | Leifsonia | Leifsonia rubra | 0.00005 |
| Actinobacteria | Micrococcales | Leifsonia | Leifsonia sp. 109 | 0.00004 |
| Actinobacteria | Micrococcales | Leifsonia | Leifsonia xyli | 0.00007 |
| Actinobacteria | Micrococcales | Leucobacter | Leucobacter chironomi | 0.00278 |
| Actinobacteria | Micrococcales | Leucobacter | Leucobacter chromiiresistens | 0.00266 |
| Actinobacteria | Micrococcales | Leucobacter | Leucobacter komagatae | 0.00243 |
| Actinobacteria | Micrococcales | Leucobacter | Leucobacter salsicius | 0.00227 |
| Actinobacteria | Micrococcales | Leucobacter | Leucobacter sp. PH1c | 0.00364 |
| Actinobacteria | Micrococcales | Leucobacter | Leucobacter sp. UCD-THU | 0.00325 |
| Actinobacteria | Micrococcales | Leucobacter | Leucobacter unclassified | 0.00029 |
| Actinobacteria | Micrococcales | Lysinimicrobium | Lysinimicrobium mangrovi | 0.00004 |
| Actinobacteria | Micrococcales | Microbacteriaceae unclassified | Microbacteriaceae unclassified | 0.00003 |
| Actinobacteria | Micrococcales | Microbacterium | Microbacterium azadirachtae | 0.00016 |
| Actinobacteria | Micrococcales | Microbacterium | Microbacterium barkeri | 0.00012 |
| Actinobacteria | Micrococcales | Microbacterium | Microbacterium foliorum | 0.00002 |
| Actinobacteria | Micrococcales | Microbacterium | Microbacterium ginsengisoli | 0.00015 |
| Actinobacteria | Micrococcales | Microbacterium | Microbacterium gubbeenense | 0.00016 |
| Actinobacteria | Micrococcales | Microbacterium | Microbacterium hominis | 0.00003 |
| Actinobacteria | Micrococcales | Microbacterium | Microbacterium indicum | 0.00002 |
| Actinobacteria | Micrococcales | Microbacterium | Microbacterium ketosireducens | 0.00014 |
| Actinobacteria | Micrococcales | Microbacterium | Microbacterium luticocti | 0.00035 |
| Actinobacteria | Micrococcales | Microbacterium | Microbacterium mangrovi | 0.00014 |
| Actinobacteria | Micrococcales | Microbacterium | Microbacterium maritypicum | 0.00004 |
| Actinobacteria | Micrococcales | Microbacterium | Microbacterium oleivorans | 0.00015 |
| Actinobacteria | Micrococcales | Microbacterium | Microbacterium oxydans | 0.00012 |
| Actinobacteria | Micrococcales | Microbacterium | Microbacterium profundi | 0.00006 |
| Actinobacteria | Micrococcales | Microbacterium | Microbacterium sp. 11MF | 0.00003 |
| Actinobacteria | Micrococcales | Microbacterium | Microbacterium sp. 292MF | 0.00006 |
| Actinobacteria | Micrococcales | Microbacterium | Microbacterium sp. B24 | 0.00002 |
| Actinobacteria | Micrococcales | Microbacterium | Microbacterium sp. C448 | 0.00006 |
| Actinobacteria | Micrococcales | Microbacterium | Microbacterium sp. CH12i | 0.00004 |
| Actinobacteria | Micrococcales | Microbacterium | Microbacterium sp. G3 | 0.00016 |
| Actinobacteria | Micrococcales | Microbacterium | Microbacterium sp. MEJ108Y | 0.00021 |
| Actinobacteria | Micrococcales | Microbacterium | Microbacterium sp. MRS-1 | 0.00014 |
| Actinobacteria | Micrococcales | Microbacterium | Microbacterium sp. SA39 | 0.00004 |
| Actinobacteria | Micrococcales | Microbacterium | Microbacterium sp. SUBG005 | 0.00005 |
| Actinobacteria | Micrococcales | Microbacterium | Microbacterium sp. TS-1 | 0.00019 |
| Actinobacteria | Micrococcales | Microbacterium | Microbacterium sp. UCD-TDU | 0.00005 |
| Actinobacteria | Micrococcales | Microbacterium | Microbacterium sp. URHA0036 | 0.00006 |
| Actinobacteria | Micrococcales | Microbacterium | Microbacterium testaceum | 0.00009 |
| Actinobacteria | Micrococcales | Microbacterium | Microbacterium trichothecenolyticum | 0.00002 |
| Actinobacteria | Micrococcales | Microbacterium | Microbacterium unclassified | 0.00025 |
| Actinobacteria | Micrococcales | Micrococcales unclassified | Micrococcales unclassified | 0.00007 |
| Actinobacteria | Micrococcales | Micrococcus | Micrococcus luteus | 0.00004 |
| Actinobacteria | Micrococcales | Micrococcus | Micrococcus sp. A1 | 0.00002 |
| Actinobacteria | Micrococcales | Micrococcus | Micrococcus sp. MS-ASIII-49 | 0.00003 |
| Actinobacteria | Micrococcales | Mobilicoccus | Mobilicoccus pelagius | 0.00012 |
| Actinobacteria | Micrococcales | Mobilicoccus | Mobilicoccus sp. SIT2 | 0.00044 |
| Actinobacteria | Micrococcales | Mycetocola | Mycetocola saprophilus | 0.00030 |
| Actinobacteria | Micrococcales | Nesterenkonia | Nesterenkonia alba | 0.00002 |
| Actinobacteria | Micrococcales | Nesterenkonia | Nesterenkonia sp. AN1 | 0.00005 |
| Actinobacteria | Micrococcales | Nesterenkonia | Nesterenkonia sp. F | 0.00001 |
| Actinobacteria | Micrococcales | Nesterenkonia | Nesterenkonia sp. NP1 | 0.00023 |
| Actinobacteria | Micrococcales | Oerskovia | Oerskovia turbata | 0.00030 |
| Actinobacteria | Micrococcales | Paraoerskovia | Paraoerskovia marina | 0.00016 |
| Actinobacteria | Micrococcales | Promicromonospora | Promicromonospora kroppenstedtii | 0.00007 |
| Actinobacteria | Micrococcales | Promicromonospora | Promicromonospora sukumoe | 0.00013 |
| Actinobacteria | Micrococcales | Promicromonospora | Promicromonospora unclassified | 0.00007 |
| Actinobacteria | Micrococcales | Promicromonosporaceae noname | Promicromonosporaceae bacterium W15 | 0.00007 |
| Actinobacteria | Micrococcales | Pseudoclavibacter | Pseudoclavibacter faecalis | 0.00022 |
| Actinobacteria | Micrococcales | Pseudoclavibacter | Pseudoclavibacter soli | 0.00002 |
| Actinobacteria | Micrococcales | Rathayibacter | Rathayibacter toxicus | 0.00003 |
| Actinobacteria | Micrococcales | Rhodoluna | Rhodoluna lacicola | 0.00015 |
| Actinobacteria | Micrococcales | Rothia | Rothia aeria | 0.00014 |
| Actinobacteria | Micrococcales | Rothia | Rothia dentocariosa | 0.00024 |
| Actinobacteria | Micrococcales | Rothia | Rothia mucilaginosa | 0.00016 |
| Actinobacteria | Micrococcales | Ruania | Ruania albidiflava | 0.00094 |
| Actinobacteria | Micrococcales | Salinibacterium | Salinibacterium sp. PAMC 21357 | 0.00005 |
| Actinobacteria | Micrococcales | Sanguibacter | Sanguibacter keddieii | 0.00025 |
| Actinobacteria | Micrococcales | Serinicoccus | Serinicoccus profundi | 0.00003 |
| Actinobacteria | Micrococcales | Sinomonas | Sinomonas sp. MUSC 117 | 0.00003 |
| Actinobacteria | Micrococcales | Terrabacter | Terrabacter sp. 28 | 0.00003 |
| Actinobacteria | Micrococcales | Tetrasphaera | Tetrasphaera australiensis | 0.00007 |
| Actinobacteria | Micrococcales | Tetrasphaera | Tetrasphaera elongata | 0.00006 |
| Actinobacteria | Micrococcales | Tetrasphaera | Tetrasphaera japonica | 0.00002 |
| Actinobacteria | Micrococcales | Timonella | Timonella senegalensis | 0.00022 |
| Actinobacteria | Micrococcales | Xylanimonas | Xylanimonas cellulosilytica | 0.00023 |
| Actinobacteria | Micrococcales | Yaniella | Yaniella halotolerans | 0.00003 |
| Actinobacteria | Micromonosporales | Actinoplanes | Actinoplanes sp. N902-109 | 0.00003 |
| Actinobacteria | Micromonosporales | Actinoplanes | Actinoplanes sp. SE50/110 | 0.00002 |
| Actinobacteria | Micromonosporales | Actinoplanes | Actinoplanes utahensis | 0.00004 |
| Actinobacteria | Micromonosporales | Hamadaea | Hamadaea tsunoensis | 0.00003 |
| Actinobacteria | Micromonosporales | Longispora | Longispora albida | 0.00004 |
| Actinobacteria | Micromonosporales | Salinispora | Salinispora arenicola | 0.00001 |
| Actinobacteria | Micromonosporales | Salinispora | Salinispora pacifica | 0.00010 |
| Actinobacteria | Nakamurellales | Nakamurella | Nakamurella lactea | 0.00003 |
| Actinobacteria | Nakamurellales | Nakamurella | Nakamurella multipartita | 0.00005 |
| Actinobacteria | Propionibacteriales | Actinopolymorpha | Actinopolymorpha alba | 0.00007 |
| Actinobacteria | Propionibacteriales | Aeromicrobium | Aeromicrobium marinum | 0.00002 |
| Actinobacteria | Propionibacteriales | Aeromicrobium | Aeromicrobium massiliense | 0.00004 |
| Actinobacteria | Propionibacteriales | Aestuariimicrobium | Aestuariimicrobium kwangyangense | 0.00004 |
| Actinobacteria | Propionibacteriales | Granulicoccus | Granulicoccus phenolivorans | 0.00008 |
| Actinobacteria | Propionibacteriales | Kribbella | Kribbella catacumbae | 0.00013 |
| Actinobacteria | Propionibacteriales | Kribbella | Kribbella flavida | 0.00010 |
| Actinobacteria | Propionibacteriales | Marmoricola | Marmoricola aequoreus | 0.00004 |
| Actinobacteria | Propionibacteriales | Microlunatus | Microlunatus phosphovorus | 0.00009 |
| Actinobacteria | Propionibacteriales | Mumia | Mumia flava | 0.00004 |
| Actinobacteria | Propionibacteriales | Nocardioidaceae noname | Nocardioidaceae bacterium Broad-1 | 0.00005 |
| Actinobacteria | Propionibacteriales | Nocardioides | Nocardioides alkalitolerans | 0.00003 |
| Actinobacteria | Propionibacteriales | Nocardioides | Nocardioides luteus | 0.00001 |
| Actinobacteria | Propionibacteriales | Nocardioides | Nocardioides sp. CF8 | 0.00004 |
| Actinobacteria | Propionibacteriales | Nocardioides | Nocardioides sp. Iso805N | 0.00002 |
| Actinobacteria | Propionibacteriales | Nocardioides | Nocardioides sp. J54 | 0.00003 |
| Actinobacteria | Propionibacteriales | Nocardioides | Nocardioides sp. JS614 | 0.00006 |
| Actinobacteria | Propionibacteriales | Nocardioides | Nocardioides sp. URHA0020 | 0.00001 |
| Actinobacteria | Propionibacteriales | Pimelobacter | Pimelobacter simplex | 0.00004 |
| Actinobacteria | Propionibacteriales | Propionibacteriaceae noname | Propionibacteriaceae bacterium P6A17 | 0.00031 |
| Actinobacteria | Propionibacteriales | Propionibacterium | Propionibacterium acidifaciens | 0.00022 |
| Actinobacteria | Propionibacteriales | Propionibacterium | Propionibacterium acidipropionici | 0.00011 |
| Actinobacteria | Propionibacteriales | Propionibacterium | Propionibacterium acnes | 0.00013 |
| Actinobacteria | Propionibacteriales | Propionibacterium | Propionibacterium avidum | 0.00013 |
| Actinobacteria | Propionibacteriales | Propionibacterium | Propionibacterium freudenreichii | 0.00060 |
| Actinobacteria | Propionibacteriales | Propionibacterium | Propionibacterium granulosum | 0.00002 |
| Actinobacteria | Propionibacteriales | Propionibacterium | Propionibacterium humerusii | 0.00004 |
| Actinobacteria | Propionibacteriales | Propionibacterium | Propionibacterium jensenii | 0.00005 |
| Actinobacteria | Propionibacteriales | Propionibacterium | Propionibacterium propionicum | 0.00198 |
| Actinobacteria | Propionibacteriales | Propionibacterium | Propionibacterium sp. 5 U 42AFAA | 0.00004 |
| Actinobacteria | Propionibacteriales | Propionibacterium | Propionibacterium sp. KPL1838 | 0.00004 |
| Actinobacteria | Propionibacteriales | Propionibacterium | Propionibacterium sp. KPL1844 | 0.00008 |
| Actinobacteria | Propionibacteriales | Propionibacterium | Propionibacterium sp. oral taxon 192 | 0.00017 |
| Actinobacteria | Propionibacteriales | Propionibacterium | Propionibacterium thoenii | 0.00002 |
| Actinobacteria | Propionibacteriales | Propionibacterium | Propionibacterium unclassified | 0.00045 |
| Actinobacteria | Propionibacteriales | Propionicicella | Propionicicella superfundia | 0.00003 |
| Actinobacteria | Propionibacteriales | Propionimicrobium | Propionimicrobium lymphophilum | 0.01186 |
| Actinobacteria | Propionibacteriales | Propionimicrobium | Propionimicrobium sp. BV2F7 | 0.00003 |
| Actinobacteria | Propionibacteriales | Propionimicrobium | Propionimicrobium unclassified | 0.00009 |
| Actinobacteria | Propionibacteriales | Tessaracoccus | Tessaracoccus sp. SIT6 | 0.00136 |
| Actinobacteria | Pseudonocardiales | Actinoalloteichus | Actinoalloteichus cyanogriseus | 0.00004 |
| Actinobacteria | Pseudonocardiales | Actinokineospora | Actinokineospora enzanensis | 0.00004 |
| Actinobacteria | Pseudonocardiales | Actinokineospora | Actinokineospora inagensis | 0.00001 |
| Actinobacteria | Pseudonocardiales | Actinomycetospora | Actinomycetospora chiangmaiensis | 0.00058 |
| Actinobacteria | Pseudonocardiales | Actinosynnema | Actinosynnema mirum | 0.00004 |
| Actinobacteria | Pseudonocardiales | Amycolatopsis | Amycolatopsis nigrescens | 0.00001 |
| Actinobacteria | Pseudonocardiales | Amycolatopsis | Amycolatopsis rifamycinica | 0.00004 |
| Actinobacteria | Pseudonocardiales | Amycolatopsis | Amycolatopsis taiwanensis | 0.00002 |
| Actinobacteria | Pseudonocardiales | Amycolatopsis | Amycolatopsis vancoresmycina | 0.00001 |
| Actinobacteria | Pseudonocardiales | Kibdelosporangium | Kibdelosporangium aridum | 0.00002 |
| Actinobacteria | Pseudonocardiales | Kibdelosporangium | Kibdelosporangium sp. MJ126-NF4 | 0.00003 |
| Actinobacteria | Pseudonocardiales | Kutzneria | Kutzneria albida | 0.00002 |
| Actinobacteria | Pseudonocardiales | Pseudonocardia | Pseudonocardia autotrophica | 0.00007 |
| Actinobacteria | Pseudonocardiales | Pseudonocardia | Pseudonocardia dioxanivorans | 0.00003 |
| Actinobacteria | Pseudonocardiales | Pseudonocardia | Pseudonocardia sp. P1 | 0.00003 |
| Actinobacteria | Pseudonocardiales | Saccharomonospora | Saccharomonospora cyanea | 0.00002 |
| Actinobacteria | Pseudonocardiales | Saccharomonospora | Saccharomonospora halophila | 0.00002 |
| Actinobacteria | Pseudonocardiales | Saccharomonospora | Saccharomonospora marina | 0.00001 |
| Actinobacteria | Pseudonocardiales | Saccharopolyspora | Saccharopolyspora erythraea | 0.00003 |
| Actinobacteria | Pseudonocardiales | Saccharopolyspora | Saccharopolyspora rectivirgula | 0.00002 |
| Actinobacteria | Pseudonocardiales | Saccharopolyspora | Saccharopolyspora spinosa | 0.00002 |
| Actinobacteria | Solirubrobacterales | Conexibacter | Conexibacter woesei | 0.00002 |
| Actinobacteria | Streptomycetales | Kitasatospora | Kitasatospora azatica | 0.00003 |
| Actinobacteria | Streptomycetales | Streptacidiphilus | Streptacidiphilus albus | 0.00012 |
| Actinobacteria | Streptomycetales | Streptacidiphilus | Streptacidiphilus anmyonensis | 0.00003 |
| Actinobacteria | Streptomycetales | Streptacidiphilus | Streptacidiphilus melanogenes | 0.00002 |
| Actinobacteria | Streptomycetales | Streptacidiphilus | Streptacidiphilus oryzae | 0.00002 |
| Actinobacteria | Streptomycetales | Streptacidiphilus | Streptacidiphilus rugosus | 0.00004 |
| Actinobacteria | Streptomycetales | Streptomyces | Streptomyces albus | 0.00003 |
| Actinobacteria | Streptomycetales | Streptomyces | Streptomyces collinus | 0.00002 |
| Actinobacteria | Streptomycetales | Streptomyces | Streptomyces lavendulae | 0.00004 |
| Actinobacteria | Streptomycetales | Streptomyces | Streptomyces mobaraensis | 0.00003 |
| Actinobacteria | Streptomycetales | Streptomyces | Streptomyces roseochromogenus | 0.00003 |
| Actinobacteria | Streptomycetales | Streptomyces | Streptomyces scabrisporus | 0.00006 |
| Actinobacteria | Streptomycetales | Streptomyces | Streptomyces scopuliridis | 0.00002 |
| Actinobacteria | Streptomycetales | Streptomyces | Streptomyces sp. AA4 | 0.00002 |
| Actinobacteria | Streptomycetales | Streptomyces | Streptomyces sp. BoleA5 | 0.00001 |
| Actinobacteria | Streptomycetales | Streptomyces | Streptomyces sp. CNS606 | 0.00009 |
| Actinobacteria | Streptomycetales | Streptomyces | Streptomyces sp. HmicA12 | 0.00004 |
| Actinobacteria | Streptomycetales | Streptomyces | Streptomyces sp. LaPpAH-108 | 0.00001 |
| Actinobacteria | Streptomycetales | Streptomyces | Streptomyces sp. NRRL F-6131 | 0.00013 |
| Actinobacteria | Streptomycetales | Streptomyces | Streptomyces sp. NRRL WC-3719 | 0.00003 |
| Actinobacteria | Streptomycetales | Streptomyces | Streptomyces sp. NTK 937 | 0.00007 |
| Actinobacteria | Streptomycetales | Streptomyces | Streptomyces toyocaensis | 0.00003 |
| Actinobacteria | Streptomycetales | Streptomyces | Streptomyces unclassified | 0.00003 |
| Actinobacteria | Streptomycetales | Streptomyces | Streptomyces viridochromogenes | 0.00006 |
| Actinobacteria | Streptomycetales | Streptomyces | Streptomyces vitaminophilus | 0.00004 |
| Actinobacteria | Streptomycetales | Streptomyces | Streptomyces wedmorensis | 0.00006 |
| Actinobacteria | Streptomycetales | Streptomyces | Streptomyces yerevanensis | 0.00001 |
| Actinobacteria | Streptosporangiales | Actinomadura | Actinomadura oligospora | 0.00005 |
| Actinobacteria | Streptosporangiales | Actinomadura | Actinomadura rifamycini | 0.00002 |
| Actinobacteria | Streptosporangiales | Nocardiopsis | Nocardiopsis alkaliphila | 0.00003 |
| Actinobacteria | Streptosporangiales | Nocardiopsis | Nocardiopsis dassonvillei | 0.00002 |
| Actinobacteria | Streptosporangiales | Nocardiopsis | Nocardiopsis kunsanensis | 0.00014 |
| Actinobacteria | Streptosporangiales | Nocardiopsis | Nocardiopsis potens | 0.00007 |
| Actinobacteria | Streptosporangiales | Nocardiopsis | Nocardiopsis salina | 0.00002 |
| Actinobacteria | Streptosporangiales | Nocardiopsis | Nocardiopsis synnemataformans | 0.00002 |
| Actinobacteria | Streptosporangiales | Nocardiopsis | Nocardiopsis unclassified | 0.00002 |
| Actinobacteria | Streptosporangiales | Nocardiopsis | Nocardiopsis valliformis | 0.00004 |
| Actinobacteria | Streptosporangiales | Spirillospora | Spirillospora albida | 0.00005 |
| Actinobacteria | Streptosporangiales | Thermobifida | Thermobifida fusca | 0.00003 |
| Aminicenantes | Aminicenantes noname | Aminicenantes noname | Aminicenantes bacterium SCGC AAA255-E10 | 0.00002 |
| Aquificae | Aquificales | Persephonella | Persephonella marina | 0.00006 |
| Aquificae | Desulfurobacteriales | Desulfurobacterium | Desulfurobacterium sp. TC5-1 | 0.00009 |
| Atribacteria | Atribacteria noname | Candidatus Caldatribacteirum | Candidatus Caldatribacterium saccharofermentans | 0.00002 |
| Bacteria noname | Bacteria noname | Bacteria noname | bacterium LF-3 | 0.02257 |
| Bacteria noname | Bacteria noname | Bacteria noname | bacterium MS4 | 0.01065 |
| Bacteria noname | Bacteria noname | Bacteria noname | bacterium OL-1 | 0.11242 |
| Bacteria noname | Bacteria noname | Bacteria noname | bacterium SIT7 | 0.00010 |
| Bacteria noname | Bacteria noname | Bacteria noname | bacterium UASB14 | 0.00048 |
| Bacteria noname | Bacteria noname | Bacteria noname | bacterium UASB270 | 0.00008 |
| Bacteria noname | Bacteria noname | Bacteria noname | bacterium YEK0313 | 0.00006 |
| Bacteria noname | Bacteria noname | Bacteria noname | candidate division SR1 bacterium RAAC1 SR1 1 | 0.00003 |
| Bacteria noname | Bacteria noname | Bacteria noname | candidate division WWE3 bacterium RAAC2 WWE3 1 | 0.00001 |
| Bacteria noname | Bacteria noname | Bacteria noname | uncultured bacterium | 2.29106 |
| Bacteria noname | Bacteria noname | Bacteria noname | uncultured bacterium (gcode 4) | 0.00012 |
| Bacteria noname | Bacteria noname | Bacteria noname | uncultured bacterium A1Q1 fos 150 | 0.00002 |
| Bacteria noname | Bacteria noname | Bacteria noname | uncultured bacterium AST2 | 0.00029 |
| Bacteria noname | Bacteria noname | Bacteria noname | uncultured bacterium Contig1338 | 0.00088 |
| Bacteria noname | Bacteria noname | Bacteria noname | uncultured bacterium Contig1450 | 0.00024 |
| Bacteria noname | Bacteria noname | Bacteria noname | uncultured bacterium Contig1495 | 0.00037 |
| Bacteria noname | Bacteria noname | Bacteria noname | uncultured bacterium Contig27 | 0.00052 |
| Bacteria noname | Bacteria noname | Bacteria noname | uncultured bacterium DCM002Carb01 | 0.00010 |
| Bacteria noname | Bacteria noname | Bacteria noname | uncultured bacterium DCM004Carb02 | 0.00003 |
| Bacteria noname | Bacteria noname | Bacteria noname | uncultured bacterium DCM008Kan04 | 0.00014 |
| Bacteria noname | Bacteria noname | Bacteria noname | uncultured bacterium EB1 | 0.00072 |
| Bacteria noname | Bacteria noname | Bacteria noname | uncultured bacterium EB3 | 0.01023 |
| Bacteria noname | Bacteria noname | Bacteria noname | uncultured bacterium EB4 | 0.00044 |
| Bacteria noname | Bacteria noname | Bacteria noname | uncultured bacterium pBIO2152 | 0.00020 |
| Bacteria noname | Bacteria noname | Bacteria noname | uncultured bacterium scaffold00056 | 0.00005 |
| Bacteria noname | Bacteria noname | Bacteria noname | uncultured eubacterium pIE1115 | 0.00025 |
| Bacteria noname | Bacteria noname | Bacteria noname | uncultured murine large bowel bacterium BAC 31B | 0.00039 |
| Bacteria noname | Bacteria noname | Bacteria noname | uncultured rumen bacterium | 0.00002 |
| Bacteria noname | Haloplasmatales | Haloplasma | Haloplasma contractile | 0.00013 |
| Bacteria unclassified | Bacteria unclassified | Bacteria unclassified | Bacteria unclassified | 0.37122 |
| Bacteroidetes | Bacteroidales | Alistipes | Alistipes finegoldii | 0.00099 |
| Bacteroidetes | Bacteroidales | Alistipes | Alistipes finegoldii CAG:68 | 0.00007 |
| Bacteroidetes | Bacteroidales | Alistipes | Alistipes indistinctus | 0.00001 |
| Bacteroidetes | Bacteroidales | Alistipes | Alistipes onderdonkii | 0.00006 |
| Bacteroidetes | Bacteroidales | Alistipes | Alistipes putredinis | 0.00166 |
| Bacteroidetes | Bacteroidales | Alistipes | Alistipes senegalensis | 0.00014 |
| Bacteroidetes | Bacteroidales | Alistipes | Alistipes shahii | 0.00026 |
| Bacteroidetes | Bacteroidales | Alistipes | Alistipes sp. 627 | 0.00008 |
| Bacteroidetes | Bacteroidales | Alistipes | Alistipes sp. AL-1 | 0.00010 |
| Bacteroidetes | Bacteroidales | Alistipes | Alistipes sp. CAG:157 | 0.00011 |
| Bacteroidetes | Bacteroidales | Alistipes | Alistipes sp. CAG:268 | 0.00009 |
| Bacteroidetes | Bacteroidales | Alistipes | Alistipes sp. CAG:29 | 0.00011 |
| Bacteroidetes | Bacteroidales | Alistipes | Alistipes sp. CAG:435 | 0.00020 |
| Bacteroidetes | Bacteroidales | Alistipes | Alistipes sp. CAG:514 | 0.00007 |
| Bacteroidetes | Bacteroidales | Alistipes | Alistipes sp. CAG:53 | 0.00006 |
| Bacteroidetes | Bacteroidales | Alistipes | Alistipes sp. CAG:831 | 0.00009 |
| Bacteroidetes | Bacteroidales | Alistipes | Alistipes sp. HGB5 | 0.00020 |
| Bacteroidetes | Bacteroidales | Alistipes | Alistipes unclassified | 0.00015 |
| Bacteroidetes | Bacteroidales | Alistipes | Candidatus Alistipes marseilloanorexicus | 0.00005 |
| Bacteroidetes | Bacteroidales | Alkaliflexus | Alkaliflexus imshenetskii | 0.00001 |
| Bacteroidetes | Bacteroidales | Alloprevotella | Alloprevotella rava | 0.00006 |
| Bacteroidetes | Bacteroidales | Alloprevotella | Prevotella sp. oral taxon 473 | 0.00026 |
| Bacteroidetes | Bacteroidales | Anaerophaga | Anaerophaga thermohalophila | 0.00028 |
| Bacteroidetes | Bacteroidales | Bacteroidaceae noname | Bacteroidaceae bacterium MS4 | 0.00110 |
| Bacteroidetes | Bacteroidales | Bacteroidales noname | Bacteroidales bacterium CF | 0.00001 |
| Bacteroidetes | Bacteroidales | Bacteroidales unclassified | Bacteroidales unclassified | 0.05520 |
| Bacteroidetes | Bacteroidales | Bacteroides | Bacteroides acidifaciens | 0.01399 |
| Bacteroidetes | Bacteroidales | Bacteroides | Bacteroides barnesiae | 0.01168 |
| Bacteroidetes | Bacteroidales | Bacteroides | Bacteroides caccae | 0.01213 |
| Bacteroidetes | Bacteroidales | Bacteroides | Bacteroides caccae CAG:21 | 0.00251 |
| Bacteroidetes | Bacteroidales | Bacteroides | Bacteroides cellulosilyticus | 0.00322 |
| Bacteroidetes | Bacteroidales | Bacteroides | Bacteroides cellulosilyticus CAG:158 | 0.00061 |
| Bacteroidetes | Bacteroidales | Bacteroides | Bacteroides clarus | 0.00756 |
| Bacteroidetes | Bacteroidales | Bacteroides | Bacteroides clarus CAG:160 | 0.00148 |
| Bacteroidetes | Bacteroidales | Bacteroides | Bacteroides coprocola | 0.03757 |
| Bacteroidetes | Bacteroidales | Bacteroides | Bacteroides coprocola CAG:162 | 0.01490 |
| Bacteroidetes | Bacteroidales | Bacteroides | Bacteroides coprophilus | 0.02230 |
| Bacteroidetes | Bacteroidales | Bacteroides | Bacteroides coprophilus CAG:333 | 0.01888 |
| Bacteroidetes | Bacteroidales | Bacteroides | Bacteroides coprosuis | 0.00001 |
| Bacteroidetes | Bacteroidales | Bacteroides | Bacteroides dorei | 0.00976 |
| Bacteroidetes | Bacteroidales | Bacteroides | Bacteroides dorei CAG:222 | 0.00008 |
| Bacteroidetes | Bacteroidales | Bacteroides | Bacteroides eggerthii | 0.00267 |
| Bacteroidetes | Bacteroidales | Bacteroides | Bacteroides eggerthii CAG:109 | 0.00066 |
| Bacteroidetes | Bacteroidales | Bacteroides | Bacteroides faecichinchillae | 0.00287 |
| Bacteroidetes | Bacteroidales | Bacteroides | Bacteroides faecis | 0.00153 |
| Bacteroidetes | Bacteroidales | Bacteroides | Bacteroides faecis CAG:32 | 0.00072 |
| Bacteroidetes | Bacteroidales | Bacteroides | Bacteroides finegoldii | 0.02809 |
| Bacteroidetes | Bacteroidales | Bacteroides | Bacteroides finegoldii CAG:203 | 0.00430 |
| Bacteroidetes | Bacteroidales | Bacteroides | Bacteroides fluxus | 0.00590 |
| Bacteroidetes | Bacteroidales | Bacteroides | Bacteroides fragilis | 0.06753 |
| Bacteroidetes | Bacteroidales | Bacteroides | Bacteroides fragilis CAG:47 | 0.00041 |
| Bacteroidetes | Bacteroidales | Bacteroides | Bacteroides fragilis CAG:558 | 0.00012 |
| Bacteroidetes | Bacteroidales | Bacteroides | Bacteroides gallinarum | 0.00339 |
| Bacteroidetes | Bacteroidales | Bacteroides | Bacteroides graminisolvens | 0.00083 |
| Bacteroidetes | Bacteroidales | Bacteroides | Bacteroides helcogenes | 0.00199 |
| Bacteroidetes | Bacteroidales | Bacteroides | Bacteroides intestinalis | 0.00235 |
| Bacteroidetes | Bacteroidales | Bacteroides | Bacteroides intestinalis CAG:315 | 0.00063 |
| Bacteroidetes | Bacteroidales | Bacteroides | Bacteroides intestinalis CAG:564 | 0.00009 |
| Bacteroidetes | Bacteroidales | Bacteroides | Bacteroides massiliensis | 0.01192 |
| Bacteroidetes | Bacteroidales | Bacteroides | Bacteroides nordii | 0.00054 |
| Bacteroidetes | Bacteroidales | Bacteroides | Bacteroides oleiciplenus | 0.00268 |
| Bacteroidetes | Bacteroidales | Bacteroides | Bacteroides ovatus | 0.00607 |
| Bacteroidetes | Bacteroidales | Bacteroides | Bacteroides ovatus CAG:22 | 0.00032 |
| Bacteroidetes | Bacteroidales | Bacteroides | Bacteroides paurosaccharolyticus | 0.00017 |
| Bacteroidetes | Bacteroidales | Bacteroides | Bacteroides pectinophilus CAG:437 | 0.00669 |
| Bacteroidetes | Bacteroidales | Bacteroides | Bacteroides plebeius | 0.03628 |
| Bacteroidetes | Bacteroidales | Bacteroides | Bacteroides plebeius CAG:211 | 0.02550 |
| Bacteroidetes | Bacteroidales | Bacteroides | Bacteroides propionicifaciens | 0.00006 |
| Bacteroidetes | Bacteroidales | Bacteroides | Bacteroides pyogenes | 0.00583 |
| Bacteroidetes | Bacteroidales | Bacteroides | Bacteroides reticulotermitis | 0.00429 |
| Bacteroidetes | Bacteroidales | Bacteroides | Bacteroides salanitronis | 0.01193 |
| Bacteroidetes | Bacteroidales | Bacteroides | Bacteroides salyersiae | 0.00134 |
| Bacteroidetes | Bacteroidales | Bacteroides | Bacteroides sartorii | 0.00130 |
| Bacteroidetes | Bacteroidales | Bacteroides | Bacteroides sp. 1 1 14 | 0.00090 |
| Bacteroidetes | Bacteroidales | Bacteroides | Bacteroides sp. 1 1 30 | 0.00094 |
| Bacteroidetes | Bacteroidales | Bacteroides | Bacteroides sp. 1 1 6 | 0.00109 |
| Bacteroidetes | Bacteroidales | Bacteroides | Bacteroides sp. 14(A) | 0.00028 |
| Bacteroidetes | Bacteroidales | Bacteroides | Bacteroides sp. 2 1 16 | 0.00066 |
| Bacteroidetes | Bacteroidales | Bacteroides | Bacteroides sp. 2 1 22 | 0.00047 |
| Bacteroidetes | Bacteroidales | Bacteroides | Bacteroides sp. 2 1 33B | 0.02609 |
| Bacteroidetes | Bacteroidales | Bacteroides | Bacteroides sp. 2 1 56FAA | 0.00135 |
| Bacteroidetes | Bacteroidales | Bacteroides | Bacteroides sp. 2 2 4 | 0.00150 |
| Bacteroidetes | Bacteroidales | Bacteroides | Bacteroides sp. 3 1 19 | 0.00290 |
| Bacteroidetes | Bacteroidales | Bacteroides | Bacteroides sp. 3 1 23 | 0.00388 |
| Bacteroidetes | Bacteroidales | Bacteroides | Bacteroides sp. 3 1 33FAA | 0.00131 |
| Bacteroidetes | Bacteroidales | Bacteroides | Bacteroides sp. 3 1 40A | 0.00330 |
| Bacteroidetes | Bacteroidales | Bacteroides | Bacteroides sp. 3 2 5 | 0.00033 |
| Bacteroidetes | Bacteroidales | Bacteroides | Bacteroides sp. 4 1 36 | 0.00030 |
| Bacteroidetes | Bacteroidales | Bacteroides | Bacteroides sp. 4 3 47FAA | 0.00332 |
| Bacteroidetes | Bacteroidales | Bacteroides | Bacteroides sp. 9 1 42FAA | 0.00180 |
| Bacteroidetes | Bacteroidales | Bacteroides | Bacteroides sp. CAG:1060 | 0.00001 |
| Bacteroidetes | Bacteroidales | Bacteroides | Bacteroides sp. CAG:1076 | 0.01979 |
| Bacteroidetes | Bacteroidales | Bacteroides | Bacteroides sp. CAG:189 | 0.00014 |
| Bacteroidetes | Bacteroidales | Bacteroides | Bacteroides sp. CAG:20 | 0.00003 |
| Bacteroidetes | Bacteroidales | Bacteroides | Bacteroides sp. CAG:443 | 0.02297 |
| Bacteroidetes | Bacteroidales | Bacteroides | Bacteroides sp. CAG:462 | 0.00453 |
| Bacteroidetes | Bacteroidales | Bacteroides | Bacteroides sp. CAG:530 | 0.01274 |
| Bacteroidetes | Bacteroidales | Bacteroides | Bacteroides sp. CAG:545 | 0.00014 |
| Bacteroidetes | Bacteroidales | Bacteroides | Bacteroides sp. CAG:598 | 0.00334 |
| Bacteroidetes | Bacteroidales | Bacteroides | Bacteroides sp. CAG:633 | 0.00784 |
| Bacteroidetes | Bacteroidales | Bacteroides | Bacteroides sp. CAG:661 | 0.00219 |
| Bacteroidetes | Bacteroidales | Bacteroides | Bacteroides sp. CAG:702 | 0.00568 |
| Bacteroidetes | Bacteroidales | Bacteroides | Bacteroides sp. CAG:709 | 0.00007 |
| Bacteroidetes | Bacteroidales | Bacteroides | Bacteroides sp. CAG:714 | 0.00968 |
| Bacteroidetes | Bacteroidales | Bacteroides | Bacteroides sp. CAG:754 | 0.01228 |
| Bacteroidetes | Bacteroidales | Bacteroides | Bacteroides sp. CAG:770 | 0.00018 |
| Bacteroidetes | Bacteroidales | Bacteroides | Bacteroides sp. CAG:875 | 0.02598 |
| Bacteroidetes | Bacteroidales | Bacteroides | Bacteroides sp. CAG:927 | 0.00018 |
| Bacteroidetes | Bacteroidales | Bacteroides | Bacteroides sp. CAG:98 | 0.00095 |
| Bacteroidetes | Bacteroidales | Bacteroides | Bacteroides sp. D1 | 0.00011 |
| Bacteroidetes | Bacteroidales | Bacteroides | Bacteroides sp. D2 | 0.00318 |
| Bacteroidetes | Bacteroidales | Bacteroides | Bacteroides sp. D20 | 0.00211 |
| Bacteroidetes | Bacteroidales | Bacteroides | Bacteroides sp. D22 | 0.00148 |
| Bacteroidetes | Bacteroidales | Bacteroides | Bacteroides sp. HPS0048 | 0.00214 |
| Bacteroidetes | Bacteroidales | Bacteroides | Bacteroides stercorirosoris | 0.00188 |
| Bacteroidetes | Bacteroidales | Bacteroides | Bacteroides stercoris | 0.02794 |
| Bacteroidetes | Bacteroidales | Bacteroides | Bacteroides stercoris CAG:120 | 0.00937 |
| Bacteroidetes | Bacteroidales | Bacteroides | Bacteroides thetaiotaomicron | 0.00233 |
| Bacteroidetes | Bacteroidales | Bacteroides | Bacteroides thetaiotaomicron CAG:40 | 0.00071 |
| Bacteroidetes | Bacteroidales | Bacteroides | Bacteroides unclassified | 0.15644 |
| Bacteroidetes | Bacteroidales | Bacteroides | Bacteroides uniformis | 0.01360 |
| Bacteroidetes | Bacteroidales | Bacteroides | Bacteroides uniformis CAG:3 | 0.00061 |
| Bacteroidetes | Bacteroidales | Bacteroides | Bacteroides vulgatus | 0.01943 |
| Bacteroidetes | Bacteroidales | Bacteroides | Bacteroides vulgatus CAG:6 | 0.00173 |
| Bacteroidetes | Bacteroidales | Bacteroides | Bacteroides xylanisolvens | 0.00455 |
| Bacteroidetes | Bacteroidales | Bacteroides | Candidatus Bacteroides timonensis | 0.00109 |
| Bacteroidetes | Bacteroidales | Bacteroides | uncultured Bacteroides sp. | 0.00003 |
| Bacteroidetes | Bacteroidales | Barnesiella | Barnesiella intestinihominis | 0.00005 |
| Bacteroidetes | Bacteroidales | Barnesiella | Barnesiella viscericola | 0.00048 |
| Bacteroidetes | Bacteroidales | Butyricimonas | Butyricimonas synergistica | 0.00015 |
| Bacteroidetes | Bacteroidales | Butyricimonas | Butyricimonas virosa | 0.00120 |
| Bacteroidetes | Bacteroidales | Coprobacter | Coprobacter fastidiosus | 0.00024 |
| Bacteroidetes | Bacteroidales | Coprobacter | Coprobacter sp. 177 | 0.00007 |
| Bacteroidetes | Bacteroidales | Draconibacterium | Draconibacterium sp. JN14CK-3 | 0.00001 |
| Bacteroidetes | Bacteroidales | Dysgonomonas | Dysgonomonas capnocytophagoides | 0.00031 |
| Bacteroidetes | Bacteroidales | Dysgonomonas | Dysgonomonas gadei | 0.00013 |
| Bacteroidetes | Bacteroidales | Dysgonomonas | Dysgonomonas mossii | 0.00004 |
| Bacteroidetes | Bacteroidales | Mucinivorans | Mucinivorans hirudinis | 0.00026 |
| Bacteroidetes | Bacteroidales | Odoribacter | Odoribacter laneus | 0.00160 |
| Bacteroidetes | Bacteroidales | Odoribacter | Odoribacter splanchnicus | 0.00041 |
| Bacteroidetes | Bacteroidales | Odoribacter | Odoribacter splanchnicus CAG:14 | 0.00007 |
| Bacteroidetes | Bacteroidales | Odoribacter | Odoribacter unclassified | 0.00007 |
| Bacteroidetes | Bacteroidales | Paludibacter | Paludibacter propionicigenes | 0.00052 |
| Bacteroidetes | Bacteroidales | Parabacteroides | Parabacteroides distasonis | 0.03572 |
| Bacteroidetes | Bacteroidales | Parabacteroides | Parabacteroides goldsteinii | 0.00179 |
| Bacteroidetes | Bacteroidales | Parabacteroides | Parabacteroides gordonii | 0.00254 |
| Bacteroidetes | Bacteroidales | Parabacteroides | Parabacteroides johnsonii | 0.00199 |
| Bacteroidetes | Bacteroidales | Parabacteroides | Parabacteroides johnsonii CAG:246 | 0.00042 |
| Bacteroidetes | Bacteroidales | Parabacteroides | Parabacteroides merdae | 0.00309 |
| Bacteroidetes | Bacteroidales | Parabacteroides | Parabacteroides merdae CAG:48 | 0.00027 |
| Bacteroidetes | Bacteroidales | Parabacteroides | Parabacteroides sp. 2 1 7 | 0.00266 |
| Bacteroidetes | Bacteroidales | Parabacteroides | Parabacteroides sp. 20 3 | 0.00142 |
| Bacteroidetes | Bacteroidales | Parabacteroides | Parabacteroides sp. ASF519 | 0.00039 |
| Bacteroidetes | Bacteroidales | Parabacteroides | Parabacteroides sp. CAG:2 | 0.02744 |
| Bacteroidetes | Bacteroidales | Parabacteroides | Parabacteroides sp. CAG:409 | 0.00345 |
| Bacteroidetes | Bacteroidales | Parabacteroides | Parabacteroides sp. D13 | 0.00848 |
| Bacteroidetes | Bacteroidales | Parabacteroides | Parabacteroides sp. D25 | 0.00019 |
| Bacteroidetes | Bacteroidales | Parabacteroides | Parabacteroides sp. HGS0025 | 0.00066 |
| Bacteroidetes | Bacteroidales | Parabacteroides | Parabacteroides unclassified | 0.05028 |
| Bacteroidetes | Bacteroidales | Paraprevotella | Paraprevotella clara | 0.00569 |
| Bacteroidetes | Bacteroidales | Paraprevotella | Paraprevotella clara CAG:116 | 0.00002 |
| Bacteroidetes | Bacteroidales | Paraprevotella | Paraprevotella xylaniphila | 0.00087 |
| Bacteroidetes | Bacteroidales | Porphyromonadaceae noname | Porphyromonadaceae bacterium COT-184 OH4590 | 0.00042 |
| Bacteroidetes | Bacteroidales | Porphyromonadaceae noname | Porphyromonadaceae bacterium ING2-E5B | 0.00025 |
| Bacteroidetes | Bacteroidales | Porphyromonadaceae unclassified | Porphyromonadaceae unclassified | 0.00012 |
| Bacteroidetes | Bacteroidales | Porphyromonas | Porphyromonas bennonis | 0.00002 |
| Bacteroidetes | Bacteroidales | Porphyromonas | Porphyromonas cangingivalis | 0.00028 |
| Bacteroidetes | Bacteroidales | Porphyromonas | Porphyromonas canoris | 0.00003 |
| Bacteroidetes | Bacteroidales | Porphyromonas | Porphyromonas cansulci | 0.00004 |
| Bacteroidetes | Bacteroidales | Porphyromonas | Porphyromonas catoniae | 0.00006 |
| Bacteroidetes | Bacteroidales | Porphyromonas | Porphyromonas crevioricanis | 0.00003 |
| Bacteroidetes | Bacteroidales | Porphyromonas | Porphyromonas gingivalis | 0.00157 |
| Bacteroidetes | Bacteroidales | Porphyromonas | Porphyromonas gulae | 0.01142 |
| Bacteroidetes | Bacteroidales | Porphyromonas | Porphyromonas macacae | 0.00023 |
| Bacteroidetes | Bacteroidales | Porphyromonas | Porphyromonas sp. 31 2 | 0.00362 |
| Bacteroidetes | Bacteroidales | Porphyromonas | Porphyromonas sp. CAG:1061 | 0.00007 |
| Bacteroidetes | Bacteroidales | Porphyromonas | Porphyromonas sp. COT-052 OH4946 | 0.00065 |
| Bacteroidetes | Bacteroidales | Porphyromonas | Porphyromonas sp. COT-108 OH1349 | 0.00003 |
| Bacteroidetes | Bacteroidales | Porphyromonas | Porphyromonas sp. COT-108 OH2963 | 0.00005 |
| Bacteroidetes | Bacteroidales | Porphyromonas | Porphyromonas sp. COT-239 OH1446 | 0.00008 |
| Bacteroidetes | Bacteroidales | Porphyromonas | Porphyromonas sp. COT-290 OH860 | 0.00008 |
| Bacteroidetes | Bacteroidales | Porphyromonas | Porphyromonas sp. KLE 1280 | 0.00002 |
| Bacteroidetes | Bacteroidales | Porphyromonas | Porphyromonas sp. oral taxon 278 | 0.00003 |
| Bacteroidetes | Bacteroidales | Porphyromonas | Porphyromonas sp. oral taxon 279 | 0.00106 |
| Bacteroidetes | Bacteroidales | Porphyromonas | Porphyromonas unclassified | 0.00157 |
| Bacteroidetes | Bacteroidales | Prevotella | Prevotella albensis | 0.00002 |
| Bacteroidetes | Bacteroidales | Prevotella | Prevotella amnii | 0.00012 |
| Bacteroidetes | Bacteroidales | Prevotella | Prevotella baroniae | 0.00024 |
| Bacteroidetes | Bacteroidales | Prevotella | Prevotella bergensis | 0.00031 |
| Bacteroidetes | Bacteroidales | Prevotella | Prevotella bivia | 0.00055 |
| Bacteroidetes | Bacteroidales | Prevotella | Prevotella brevis | 0.00010 |
| Bacteroidetes | Bacteroidales | Prevotella | Prevotella bryantii | 0.00091 |
| Bacteroidetes | Bacteroidales | Prevotella | Prevotella buccae | 0.00012 |
| Bacteroidetes | Bacteroidales | Prevotella | Prevotella buccalis | 0.00039 |
| Bacteroidetes | Bacteroidales | Prevotella | Prevotella copri | 0.00069 |
| Bacteroidetes | Bacteroidales | Prevotella | Prevotella copri CAG:164 | 0.00013 |
| Bacteroidetes | Bacteroidales | Prevotella | Prevotella corporis | 0.00002 |
| Bacteroidetes | Bacteroidales | Prevotella | Prevotella dentalis | 0.00014 |
| Bacteroidetes | Bacteroidales | Prevotella | Prevotella dentasini | 0.00037 |
| Bacteroidetes | Bacteroidales | Prevotella | Prevotella denticola | 0.00006 |
| Bacteroidetes | Bacteroidales | Prevotella | Prevotella disiens | 0.00648 |
| Bacteroidetes | Bacteroidales | Prevotella | Prevotella enoeca | 0.00004 |
| Bacteroidetes | Bacteroidales | Prevotella | Prevotella fusca | 0.00004 |
| Bacteroidetes | Bacteroidales | Prevotella | Prevotella histicola | 0.00003 |
| Bacteroidetes | Bacteroidales | Prevotella | Prevotella intermedia | 0.00003 |
| Bacteroidetes | Bacteroidales | Prevotella | Prevotella loescheii | 0.00020 |
| Bacteroidetes | Bacteroidales | Prevotella | Prevotella maculosa | 0.00010 |
| Bacteroidetes | Bacteroidales | Prevotella | Prevotella marshii | 0.00016 |
| Bacteroidetes | Bacteroidales | Prevotella | Prevotella melaninogenica | 0.00195 |
| Bacteroidetes | Bacteroidales | Prevotella | Prevotella multiformis | 0.00015 |
| Bacteroidetes | Bacteroidales | Prevotella | Prevotella multisaccharivorax | 0.00275 |
| Bacteroidetes | Bacteroidales | Prevotella | Prevotella nanceiensis | 0.00013 |
| Bacteroidetes | Bacteroidales | Prevotella | Prevotella nigrescens | 0.00023 |
| Bacteroidetes | Bacteroidales | Prevotella | Prevotella oralis | 0.00016 |
| Bacteroidetes | Bacteroidales | Prevotella | Prevotella oris | 0.00010 |
| Bacteroidetes | Bacteroidales | Prevotella | Prevotella oryzae | 0.00027 |
| Bacteroidetes | Bacteroidales | Prevotella | Prevotella oulorum | 0.00090 |
| Bacteroidetes | Bacteroidales | Prevotella | Prevotella pleuritidis | 0.00033 |
| Bacteroidetes | Bacteroidales | Prevotella | Prevotella ruminicola | 0.00032 |
| Bacteroidetes | Bacteroidales | Prevotella | Prevotella saccharolytica | 0.00008 |
| Bacteroidetes | Bacteroidales | Prevotella | Prevotella scopos | 0.00006 |
| Bacteroidetes | Bacteroidales | Prevotella | Prevotella shahii | 0.00046 |
| Bacteroidetes | Bacteroidales | Prevotella | Prevotella sp. 10(H) | 0.00009 |
| Bacteroidetes | Bacteroidales | Prevotella | Prevotella sp. AGR2160 | 0.00027 |
| Bacteroidetes | Bacteroidales | Prevotella | Prevotella sp. C561 | 0.00021 |
| Bacteroidetes | Bacteroidales | Prevotella | Prevotella sp. CAG:1031 | 0.00032 |
| Bacteroidetes | Bacteroidales | Prevotella | Prevotella sp. CAG:1058 | 0.00073 |
| Bacteroidetes | Bacteroidales | Prevotella | Prevotella sp. CAG:1092 | 0.00036 |
| Bacteroidetes | Bacteroidales | Prevotella | Prevotella sp. CAG:1124 | 0.00029 |
| Bacteroidetes | Bacteroidales | Prevotella | Prevotella sp. CAG:1185 | 0.00040 |
| Bacteroidetes | Bacteroidales | Prevotella | Prevotella sp. CAG:1320 | 0.00030 |
| Bacteroidetes | Bacteroidales | Prevotella | Prevotella sp. CAG:255 | 0.00079 |
| Bacteroidetes | Bacteroidales | Prevotella | Prevotella sp. CAG:279 | 0.00001 |
| Bacteroidetes | Bacteroidales | Prevotella | Prevotella sp. CAG:474 | 0.00571 |
| Bacteroidetes | Bacteroidales | Prevotella | Prevotella sp. CAG:485 | 0.00002 |
| Bacteroidetes | Bacteroidales | Prevotella | Prevotella sp. CAG:487 | 0.00040 |
| Bacteroidetes | Bacteroidales | Prevotella | Prevotella sp. CAG:520 | 0.00120 |
| Bacteroidetes | Bacteroidales | Prevotella | Prevotella sp. CAG:5226 | 0.00032 |
| Bacteroidetes | Bacteroidales | Prevotella | Prevotella sp. CAG:592 | 0.00009 |
| Bacteroidetes | Bacteroidales | Prevotella | Prevotella sp. CAG:604 | 0.00006 |
| Bacteroidetes | Bacteroidales | Prevotella | Prevotella sp. CAG:617 | 0.00037 |
| Bacteroidetes | Bacteroidales | Prevotella | Prevotella sp. CAG:732 | 0.00032 |
| Bacteroidetes | Bacteroidales | Prevotella | Prevotella sp. CAG:755 | 0.00029 |
| Bacteroidetes | Bacteroidales | Prevotella | Prevotella sp. CAG:873 | 0.00013 |
| Bacteroidetes | Bacteroidales | Prevotella | Prevotella sp. CAG:891 | 0.00323 |
| Bacteroidetes | Bacteroidales | Prevotella | Prevotella sp. CAG:924 | 0.00039 |
| Bacteroidetes | Bacteroidales | Prevotella | Prevotella sp. F0091 | 0.00031 |
| Bacteroidetes | Bacteroidales | Prevotella | Prevotella sp. FD3004 | 0.00001 |
| Bacteroidetes | Bacteroidales | Prevotella | Prevotella sp. HUN102 | 0.00031 |
| Bacteroidetes | Bacteroidales | Prevotella | Prevotella sp. ICM33 | 0.00094 |
| Bacteroidetes | Bacteroidales | Prevotella | Prevotella sp. MA2016 | 0.00027 |
| Bacteroidetes | Bacteroidales | Prevotella | Prevotella sp. oral taxon 299 | 0.00004 |
| Bacteroidetes | Bacteroidales | Prevotella | Prevotella sp. oral taxon 306 | 0.00004 |
| Bacteroidetes | Bacteroidales | Prevotella | Prevotella sp. oral taxon 317 | 0.00036 |
| Bacteroidetes | Bacteroidales | Prevotella | Prevotella sp. P4-65 | 0.00003 |
| Bacteroidetes | Bacteroidales | Prevotella | Prevotella sp. P4-76 | 0.00029 |
| Bacteroidetes | Bacteroidales | Prevotella | Prevotella sp. P5-119 | 0.00044 |
| Bacteroidetes | Bacteroidales | Prevotella | Prevotella sp. P5-125 | 0.00016 |
| Bacteroidetes | Bacteroidales | Prevotella | Prevotella sp. P5-60 | 0.00224 |
| Bacteroidetes | Bacteroidales | Prevotella | Prevotella sp. P6B1 | 0.00002 |
| Bacteroidetes | Bacteroidales | Prevotella | Prevotella sp. P6B4 | 0.00001 |
| Bacteroidetes | Bacteroidales | Prevotella | Prevotella sp. RM4 | 0.00004 |
| Bacteroidetes | Bacteroidales | Prevotella | Prevotella sp. S7 MS 2 | 0.00013 |
| Bacteroidetes | Bacteroidales | Prevotella | Prevotella sp. S7-1-8 | 0.00030 |
| Bacteroidetes | Bacteroidales | Prevotella | Prevotella stercorea | 0.00021 |
| Bacteroidetes | Bacteroidales | Prevotella | Prevotella stercorea CAG:629 | 0.00002 |
| Bacteroidetes | Bacteroidales | Prevotella | Prevotella timonensis | 0.00661 |
| Bacteroidetes | Bacteroidales | Prevotella | Prevotella unclassified | 0.00080 |
| Bacteroidetes | Bacteroidales | Prevotella | Prevotella veroralis | 0.00011 |
| Bacteroidetes | Bacteroidales | Prolixibacter | Prolixibacter bellariivorans | 0.00008 |
| Bacteroidetes | Bacteroidales | Proteiniphilum | Proteiniphilum acetatigenes | 0.00004 |
| Bacteroidetes | Bacteroidales | Rikenella | Rikenella microfusus | 0.00010 |
| Bacteroidetes | Bacteroidales | Saccharicrinis | Saccharicrinis fermentans | 0.00004 |
| Bacteroidetes | Bacteroidales | Sanguibacteroides | Sanguibacteroides justesenii | 0.00030 |
| Bacteroidetes | Bacteroidales | Tannerella | Tannerella forsythia | 0.00298 |
| Bacteroidetes | Bacteroidales | Tannerella | Tannerella sp. 6 1 58FAA CT1 | 0.00037 |
| Bacteroidetes | Bacteroidales | Tannerella | Tannerella sp. CAG:118 | 0.00039 |
| Bacteroidetes | Bacteroidales | Tannerella | Tannerella sp. CAG:51 | 0.00005 |
| Bacteroidetes | Bacteroidales | Tannerella | Tannerella sp. oral taxon BU063 | 0.00017 |
| Bacteroidetes | Bacteroidales | Thermophagus | Thermophagus xiamenensis | 0.00078 |
| Bacteroidetes | Bacteroidetes noname | Bacteroidetes noname | Bacteroidetes bacterium oral taxon 272 | 0.00003 |
| Bacteroidetes | Bacteroidetes noname | Bacteroidetes noname | Bacteroidetes oral taxon 274 | 0.00170 |
| Bacteroidetes | Bacteroidetes noname | Bacteroidetes noname | unidentified eubacterium SCB49 | 0.00004 |
| Bacteroidetes | Bacteroidetes unclassified | Bacteroidetes unclassified | Bacteroidetes unclassified | 0.00058 |
| Bacteroidetes | Cytophagales | Adhaeribacter | Adhaeribacter aquaticus | 0.00005 |
| Bacteroidetes | Cytophagales | Algoriphagus | Algoriphagus mannitolivorans | 0.00002 |
| Bacteroidetes | Cytophagales | Algoriphagus | Algoriphagus marincola | 0.00004 |
| Bacteroidetes | Cytophagales | Algoriphagus | Algoriphagus terrigena | 0.00001 |
| Bacteroidetes | Cytophagales | Algoriphagus | Algoriphagus vanfongensis | 0.00003 |
| Bacteroidetes | Cytophagales | Cyclobacterium | Cyclobacterium marinum | 0.00009 |
| Bacteroidetes | Cytophagales | Cyclobacterium | Cyclobacterium qasimii | 0.00003 |
| Bacteroidetes | Cytophagales | Cytophagaceae noname | Cytophagaceae bacterium JGI 0001001-B3 | 0.00003 |
| Bacteroidetes | Cytophagales | Echinicola | Echinicola pacifica | 0.00002 |
| Bacteroidetes | Cytophagales | Echinicola | Echinicola vietnamensis | 0.00003 |
| Bacteroidetes | Cytophagales | Flectobacillus | Flectobacillus major | 0.00006 |
| Bacteroidetes | Cytophagales | Hymenobacter | Hymenobacter aerophilus | 0.00015 |
| Bacteroidetes | Cytophagales | Hymenobacter | Hymenobacter sp. DG25B | 0.00004 |
| Bacteroidetes | Cytophagales | Hymenobacter | Hymenobacter sp. IS2118 | 0.00004 |
| Bacteroidetes | Cytophagales | Hymenobacter | Hymenobacter swuensis | 0.00001 |
| Bacteroidetes | Cytophagales | Pontibacter | Pontibacter roseus | 0.00006 |
| Bacteroidetes | Cytophagales | Runella | Runella limosa | 0.00010 |
| Bacteroidetes | Flavobacteriales | Algibacter | Algibacter lectus | 0.00008 |
| Bacteroidetes | Flavobacteriales | Aquimarina | Aquimarina latercula | 0.00005 |
| Bacteroidetes | Flavobacteriales | Aquimarina | Aquimarina muelleri | 0.00002 |
| Bacteroidetes | Flavobacteriales | Aquimarina | Aquimarina sp. 22II-S11-z7 | 0.00029 |
| Bacteroidetes | Flavobacteriales | Arenibacter | Arenibacter algicola | 0.00002 |
| Bacteroidetes | Flavobacteriales | Bergeyella | Bergeyella zoohelcum | 0.03137 |
| Bacteroidetes | Flavobacteriales | Capnocytophaga | Capnocytophaga canimorsus | 0.01336 |
| Bacteroidetes | Flavobacteriales | Capnocytophaga | Capnocytophaga cynodegmi | 0.01447 |
| Bacteroidetes | Flavobacteriales | Capnocytophaga | Capnocytophaga granulosa | 0.00003 |
| Bacteroidetes | Flavobacteriales | Capnocytophaga | Capnocytophaga ochracea | 0.00003 |
| Bacteroidetes | Flavobacteriales | Capnocytophaga | Capnocytophaga sp. CM59 | 0.00013 |
| Bacteroidetes | Flavobacteriales | Capnocytophaga | Capnocytophaga sp. oral taxon 324 | 0.00003 |
| Bacteroidetes | Flavobacteriales | Capnocytophaga | Capnocytophaga sp. oral taxon 326 | 0.00082 |
| Bacteroidetes | Flavobacteriales | Capnocytophaga | Capnocytophaga sp. oral taxon 329 | 0.00004 |
| Bacteroidetes | Flavobacteriales | Capnocytophaga | Capnocytophaga sp. oral taxon 332 | 0.00025 |
| Bacteroidetes | Flavobacteriales | Capnocytophaga | Capnocytophaga sp. oral taxon 336 | 0.00002 |
| Bacteroidetes | Flavobacteriales | Capnocytophaga | Capnocytophaga sp. oral taxon 338 | 0.00015 |
| Bacteroidetes | Flavobacteriales | Capnocytophaga | Capnocytophaga sp. oral taxon 863 | 0.00027 |
| Bacteroidetes | Flavobacteriales | Capnocytophaga | Capnocytophaga sputigena | 0.00141 |
| Bacteroidetes | Flavobacteriales | Capnocytophaga | Capnocytophaga unclassified | 0.00148 |
| Bacteroidetes | Flavobacteriales | Cellulophaga | Cellulophaga algicola | 0.00002 |
| Bacteroidetes | Flavobacteriales | Cellulophaga | Cellulophaga geojensis | 0.00007 |
| Bacteroidetes | Flavobacteriales | Chryseobacterium | Chryseobacterium antarcticum | 0.00016 |
| Bacteroidetes | Flavobacteriales | Chryseobacterium | Chryseobacterium caeni | 0.00033 |
| Bacteroidetes | Flavobacteriales | Chryseobacterium | Chryseobacterium daeguense | 0.00147 |
| Bacteroidetes | Flavobacteriales | Chryseobacterium | Chryseobacterium formosense | 0.00017 |
| Bacteroidetes | Flavobacteriales | Chryseobacterium | Chryseobacterium gleum | 0.00039 |
| Bacteroidetes | Flavobacteriales | Chryseobacterium | Chryseobacterium haifense | 0.00025 |
| Bacteroidetes | Flavobacteriales | Chryseobacterium | Chryseobacterium hispalense | 0.00075 |
| Bacteroidetes | Flavobacteriales | Chryseobacterium | Chryseobacterium jeonii | 0.00046 |
| Bacteroidetes | Flavobacteriales | Chryseobacterium | Chryseobacterium luteum | 0.00002 |
| Bacteroidetes | Flavobacteriales | Chryseobacterium | Chryseobacterium oranimense | 0.00009 |
| Bacteroidetes | Flavobacteriales | Chryseobacterium | Chryseobacterium palustre | 0.00016 |
| Bacteroidetes | Flavobacteriales | Chryseobacterium | Chryseobacterium piperi | 0.00017 |
| Bacteroidetes | Flavobacteriales | Chryseobacterium | Chryseobacterium soli | 0.00014 |
| Bacteroidetes | Flavobacteriales | Chryseobacterium | Chryseobacterium solincola | 0.00061 |
| Bacteroidetes | Flavobacteriales | Chryseobacterium | Chryseobacterium sp. CF365 | 0.00027 |
| Bacteroidetes | Flavobacteriales | Chryseobacterium | Chryseobacterium sp. JM1 | 0.00024 |
| Bacteroidetes | Flavobacteriales | Chryseobacterium | Chryseobacterium sp. OV259 | 0.00338 |
| Bacteroidetes | Flavobacteriales | Chryseobacterium | Chryseobacterium sp. P1-3 | 0.00030 |
| Bacteroidetes | Flavobacteriales | Chryseobacterium | Chryseobacterium sp. StRB126 | 0.00069 |
| Bacteroidetes | Flavobacteriales | Chryseobacterium | Chryseobacterium taiwanense | 0.00167 |
| Bacteroidetes | Flavobacteriales | Chryseobacterium | Chryseobacterium unclassified | 0.00030 |
| Bacteroidetes | Flavobacteriales | Chryseobacterium | Chryseobacterium vrystaatense | 0.00024 |
| Bacteroidetes | Flavobacteriales | Dokdonia | Dokdonia sp. 4H-3-7-5 | 0.00001 |
| Bacteroidetes | Flavobacteriales | Dokdonia | Dokdonia sp. PRO95 | 0.00004 |
| Bacteroidetes | Flavobacteriales | Elizabethkingia | Elizabethkingia anophelis | 0.00004 |
| Bacteroidetes | Flavobacteriales | Elizabethkingia | Elizabethkingia meningoseptica | 0.00034 |
| Bacteroidetes | Flavobacteriales | Elizabethkingia | Elizabethkingia miricola | 0.00011 |
| Bacteroidetes | Flavobacteriales | Elizabethkingia | Elizabethkingia sp. BM10 | 0.00003 |
| Bacteroidetes | Flavobacteriales | Elizabethkingia | Elizabethkingia unclassified | 0.00016 |
| Bacteroidetes | Flavobacteriales | Empedobacter | Empedobacter brevis | 0.00082 |
| Bacteroidetes | Flavobacteriales | Empedobacter | Empedobacter falsenii | 0.00006 |
| Bacteroidetes | Flavobacteriales | Epilithonimonas | Epilithonimonas lactis | 0.00022 |
| Bacteroidetes | Flavobacteriales | Epilithonimonas | Epilithonimonas sp. FH1 | 0.00024 |
| Bacteroidetes | Flavobacteriales | Epilithonimonas | Epilithonimonas tenax | 0.00095 |
| Bacteroidetes | Flavobacteriales | Epilithonimonas | Epilithonimonas unclassified | 0.00031 |
| Bacteroidetes | Flavobacteriales | Flaviramulus | Flaviramulus ichthyoenteri | 0.00003 |
| Bacteroidetes | Flavobacteriales | Flavobacteriaceae noname | Flavobacteriaceae bacterium 3519-10 | 0.00110 |
| Bacteroidetes | Flavobacteriales | Flavobacteriaceae unclassified | Flavobacteriaceae unclassified | 0.00022 |
| Bacteroidetes | Flavobacteriales | Flavobacteriales noname | Flavobacteria bacterium BAL38 | 0.00007 |
| Bacteroidetes | Flavobacteriales | Flavobacteriales noname | Flavobacteriales bacterium BRH c54 | 0.00020 |
| Bacteroidetes | Flavobacteriales | Flavobacterium | Flavobacterium antarcticum | 0.00008 |
| Bacteroidetes | Flavobacteriales | Flavobacterium | Flavobacterium aquatile | 0.00004 |
| Bacteroidetes | Flavobacteriales | Flavobacterium | Flavobacterium beibuense | 0.00006 |
| Bacteroidetes | Flavobacteriales | Flavobacterium | Flavobacterium branchiophilum | 0.00016 |
| Bacteroidetes | Flavobacteriales | Flavobacterium | Flavobacterium cauense | 0.00058 |
| Bacteroidetes | Flavobacteriales | Flavobacterium | Flavobacterium chungangense | 0.00002 |
| Bacteroidetes | Flavobacteriales | Flavobacterium | Flavobacterium columnare | 0.00062 |
| Bacteroidetes | Flavobacteriales | Flavobacterium | Flavobacterium daejeonense | 0.00001 |
| Bacteroidetes | Flavobacteriales | Flavobacterium | Flavobacterium enshiense | 0.00106 |
| Bacteroidetes | Flavobacteriales | Flavobacterium | Flavobacterium filum | 0.00003 |
| Bacteroidetes | Flavobacteriales | Flavobacterium | Flavobacterium frigidarium | 0.00014 |
| Bacteroidetes | Flavobacteriales | Flavobacterium | Flavobacterium frigoris | 0.00002 |
| Bacteroidetes | Flavobacteriales | Flavobacterium | Flavobacterium gelidilacus | 0.00004 |
| Bacteroidetes | Flavobacteriales | Flavobacterium | Flavobacterium indicum | 0.00012 |
| Bacteroidetes | Flavobacteriales | Flavobacterium | Flavobacterium johnsoniae | 0.00002 |
| Bacteroidetes | Flavobacteriales | Flavobacterium | Flavobacterium limnosediminis | 0.00017 |
| Bacteroidetes | Flavobacteriales | Flavobacterium | Flavobacterium psychrophilum | 0.00057 |
| Bacteroidetes | Flavobacteriales | Flavobacterium | Flavobacterium reichenbachii | 0.00002 |
| Bacteroidetes | Flavobacteriales | Flavobacterium | Flavobacterium saliperosum | 0.00015 |
| Bacteroidetes | Flavobacteriales | Flavobacterium | Flavobacterium sasangense | 0.00049 |
| Bacteroidetes | Flavobacteriales | Flavobacterium | Flavobacterium soli | 0.00002 |
| Bacteroidetes | Flavobacteriales | Flavobacterium | Flavobacterium sp. 316 | 0.00003 |
| Bacteroidetes | Flavobacteriales | Flavobacterium | Flavobacterium sp. 83 | 0.00004 |
| Bacteroidetes | Flavobacteriales | Flavobacterium | Flavobacterium sp. ACAM 123 | 0.00001 |
| Bacteroidetes | Flavobacteriales | Flavobacterium | Flavobacterium sp. B17 | 0.00002 |
| Bacteroidetes | Flavobacteriales | Flavobacterium | Flavobacterium sp. EM1308 | 0.00004 |
| Bacteroidetes | Flavobacteriales | Flavobacterium | Flavobacterium sp. F52 | 0.00019 |
| Bacteroidetes | Flavobacteriales | Flavobacterium | Flavobacterium sp. MEB061 | 0.00015 |
| Bacteroidetes | Flavobacteriales | Flavobacterium | Flavobacterium subsaxonicum | 0.00060 |
| Bacteroidetes | Flavobacteriales | Flavobacterium | Flavobacterium suncheonense | 0.00020 |
| Bacteroidetes | Flavobacteriales | Flavobacterium | Flavobacterium tegetincola | 0.00023 |
| Bacteroidetes | Flavobacteriales | Flavobacterium | Flavobacterium unclassified | 0.00002 |
| Bacteroidetes | Flavobacteriales | Fluviicola | Fluviicola taffensis | 0.00020 |
| Bacteroidetes | Flavobacteriales | Galbibacter | Galbibacter marinus | 0.00002 |
| Bacteroidetes | Flavobacteriales | Gelidibacter | Gelidibacter mesophilus | 0.00002 |
| Bacteroidetes | Flavobacteriales | Gramella | Gramella forsetii | 0.00003 |
| Bacteroidetes | Flavobacteriales | Imtechella | Imtechella halotolerans | 0.00009 |
| Bacteroidetes | Flavobacteriales | Jejuia | Jejuia pallidilutea | 0.00002 |
| Bacteroidetes | Flavobacteriales | Leeuwenhoekiella | Leeuwenhoekiella blandensis | 0.00077 |
| Bacteroidetes | Flavobacteriales | Leeuwenhoekiella | Leeuwenhoekiella sp. MAR 2009 132 | 0.00003 |
| Bacteroidetes | Flavobacteriales | Maribacter | Maribacter sp. HTCC2170 | 0.00024 |
| Bacteroidetes | Flavobacteriales | Muricauda | Muricauda ruestringensis | 0.00001 |
| Bacteroidetes | Flavobacteriales | Muricauda | Muricauda sp. MAR 2010 75 | 0.00001 |
| Bacteroidetes | Flavobacteriales | Myroides | Myroides injenensis | 0.00081 |
| Bacteroidetes | Flavobacteriales | Myroides | Myroides odoratimimus | 0.00246 |
| Bacteroidetes | Flavobacteriales | Myroides | Myroides profundi | 0.00006 |
| Bacteroidetes | Flavobacteriales | Myroides | Myroides unclassified | 0.00051 |
| Bacteroidetes | Flavobacteriales | Ornithobacterium | Ornithobacterium rhinotracheale | 0.00215 |
| Bacteroidetes | Flavobacteriales | Riemerella | Riemerella anatipestifer | 0.00333 |
| Bacteroidetes | Flavobacteriales | Riemerella | Riemerella columbina | 0.00170 |
| Bacteroidetes | Flavobacteriales | Riemerella | Riemerella unclassified | 0.00004 |
| Bacteroidetes | Flavobacteriales | Salinimicrobium | Salinimicrobium terrae | 0.00007 |
| Bacteroidetes | Flavobacteriales | Schleiferia | Schleiferia thermophila | 0.00001 |
| Bacteroidetes | Flavobacteriales | Tamlana | Tamlana sedimentorum | 0.00001 |
| Bacteroidetes | Flavobacteriales | Tenacibaculum | Tenacibaculum ovolyticum | 0.00011 |
| Bacteroidetes | Flavobacteriales | Tenacibaculum | Tenacibaculum sp. 47A GOM-205m | 0.00004 |
| Bacteroidetes | Flavobacteriales | Weeksella | Weeksella sp. FF8 | 0.00002 |
| Bacteroidetes | Flavobacteriales | Weeksella | Weeksella virosa | 0.00007 |
| Bacteroidetes | Flavobacteriales | Winogradskyella | Winogradskyella sp. PG-2 | 0.00002 |
| Bacteroidetes | Flavobacteriales | Zunongwangia | Zunongwangia profunda | 0.00007 |
| Bacteroidetes | Flavobacteriia noname | Flavobacteriia noname | uncultured Flavobacteriia bacterium | 0.00004 |
| Bacteroidetes | Sphingobacteriales | Chitinophaga | Chitinophaga pinensis | 0.00003 |
| Bacteroidetes | Sphingobacteriales | Flavihumibacter | Flavihumibacter petaseus | 0.00001 |
| Bacteroidetes | Sphingobacteriales | Niabella | Niabella aurantiaca | 0.00007 |
| Bacteroidetes | Sphingobacteriales | Pedobacter | Pedobacter sp. R20-19 | 0.00028 |
| Bacteroidetes | Sphingobacteriales | Phaeodactylibacter | Phaeodactylibacter xiamenensis | 0.00034 |
| Bacteroidetes | Sphingobacteriales | Pseudopedobacter | Pseudopedobacter saltans | 0.00004 |
| Bacteroidetes | Sphingobacteriales | Saprospira | Saprospira grandis | 0.00018 |
| Bacteroidetes | Sphingobacteriales | Sediminibacterium | Sediminibacterium sp. C3 | 0.00007 |
| Bacteroidetes | Sphingobacteriales | Sediminibacterium | Sediminibacterium sp. OR53 | 0.00013 |
| Bacteroidetes | Sphingobacteriales | Solitalea | Solitalea canadensis | 0.00002 |
| Bacteroidetes | Sphingobacteriales | Sphingobacterium | Sphingobacterium paucimobilis | 0.00006 |
| Bacteroidetes | Sphingobacteriales | Sphingobacterium | Sphingobacterium sp. 21 | 0.00004 |
| Bacteroidetes | Sphingobacteriales | Sphingobacterium | Sphingobacterium sp. H1ai | 0.00002 |
| Bacteroidetes | Sphingobacteriales | Sphingobacterium | Sphingobacterium sp. ML3W | 0.00003 |
| Bacteroidetes | Sphingobacteriales | Sphingobacterium | Sphingobacterium sp. PM2-P1-29 | 0.00001 |
| Candidatus Saccharibacteria | Candidatus Saccharibacteria noname | Candidatus Saccharibacteria noname | candidate division TM7 bacterium JGI 0001002-L20 | 0.01184 |
| Candidatus Saccharibacteria | Candidatus Saccharibacteria noname | Candidatus Saccharibacteria noname | candidate division TM7 genomosp. GTL1 | 0.00224 |
| Candidatus Saccharibacteria | Candidatus Saccharibacteria noname | Candidatus Saccharibacteria noname | candidate division TM7 single-cell isolate TM7c | 0.01184 |
| Candidatus Saccharibacteria | Candidatus Saccharibacteria noname | Candidatus Saccharibacteria noname | Candidatus Saccharibacteria bacterium RAAC3 TM7 1 | 0.01786 |
| Candidatus Saccharibacteria | Candidatus Saccharibacteria noname | Candidatus Saccharibacteria noname | Candidatus Saccharibacteria oral taxon TM7x | 0.05427 |
| Candidatus Saccharibacteria | Candidatus Saccharibacteria noname | Candidatus Saccharimonas | Candidatus Saccharimonas aalborgensis | 0.01128 |
| Candidatus Saccharibacteria | Candidatus Saccharibacteria unclassified | Candidatus Saccharibacteria unclassified | Candidatus Saccharibacteria unclassified | 0.00035 |
| Chlamydiae | Chlamydiales | Chlamydia | Chlamydia psittaci | 0.00131 |
| Chlamydiae | Chlamydiales | Chlamydia | Chlamydia sp. 'Rubis' | 0.00005 |
| Chlorobi | Chlorobiales | Chlorobium | Chlorobium chlorochromatii | 0.00015 |
| Chlorobi | Chlorobiales | Chlorobium | Chlorobium phaeobacteroides | 0.00002 |
| Chlorobi | Chlorobiales | Chloroherpeton | Chloroherpeton thalassium | 0.00006 |
| Chloroflexi | Anaerolineales | Anaerolinea | Anaerolinea thermophila | 0.00009 |
| Chloroflexi | Chloroflexales | Chloroflexus | Chloroflexus aggregans | 0.00001 |
| Chloroflexi | Chloroflexales | Roseiflexus | Roseiflexus castenholzii | 0.00002 |
| Chloroflexi | Chloroflexi noname | Chloroflexi noname | Chloroflexi bacterium oral taxon 439 | 0.00010 |
| Chloroflexi | Dehalococcoidales | Dehalococcoides | Dehalococcoides mccartyi | 0.00084 |
| Chloroflexi | Herpetosiphonales | Herpetosiphon | Herpetosiphon aurantiacus | 0.00002 |
| Chloroflexi | Sphaerobacterales | Sphaerobacter | Sphaerobacter thermophilus | 0.00001 |
| Chrysiogenetes | Chrysiogenales | Chrysiogenes | Chrysiogenes arsenatis | 0.00001 |
| Cloacimonetes | Cloacimonetes unclassified | Cloacimonetes unclassified | Cloacimonetes unclassified | 0.00004 |
| Cyanobacteria | Chroococcales | Crocosphaera | Crocosphaera watsonii | 0.00003 |
| Cyanobacteria | Chroococcales | Cyanobium | Cyanobium sp. CACIAM 14 | 0.00001 |
| Cyanobacteria | Chroococcales | Cyanothece | Cyanothece sp. PCC 7822 | 0.00002 |
| Cyanobacteria | Chroococcales | Dactylococcopsis | Dactylococcopsis salina | 0.00023 |
| Cyanobacteria | Chroococcales | Microcystis | Microcystis aeruginosa | 0.00002 |
| Cyanobacteria | Chroococcales | Synechococcus | Synechococcus sp. PCC 6312 | 0.00002 |
| Cyanobacteria | Nostocales | Anabaena | Anabaena sp. PCC 7108 | 0.00001 |
| Cyanobacteria | Nostocales | Scytonema | Scytonema tolypothrichoides | 0.00024 |
| Cyanobacteria | Oscillatoriales | Crinalium | Crinalium epipsammum | 0.00004 |
| Cyanobacteria | Oscillatoriales | Leptolyngbya | Leptolyngbya sp. Heron Island J | 0.00015 |
| Cyanobacteria | Oscillatoriales | Leptolyngbya | Leptolyngbya sp. PCC 7375 | 0.00009 |
| Cyanobacteria | Oscillatoriales | Planktothrix | Planktothrix agardhii | 0.00009 |
| Cyanobacteria | Oscillatoriales | Planktothrix | Planktothrix unclassified | 0.00004 |
| Cyanobacteria | Oscillatoriales | Spirulina | Spirulina subsalsa | 0.00001 |
| Cyanobacteria | Pleurocapsales | Stanieria | Stanieria cyanosphaera | 0.00002 |
| Cyanobacteria | Pleurocapsales | Xenococcus | Xenococcus sp. PCC 7305 | 0.00004 |
| Cyanobacteria | Stigonematales | Fischerella | Fischerella muscicola | 0.00029 |
| Deferribacteres | Deferribacterales | Calditerrivibrio | Calditerrivibrio nitroreducens | 0.00026 |
| Deferribacteres | Deferribacterales | Deferribacter | Deferribacter desulfuricans | 0.00004 |
| Deferribacteres | Deferribacterales | Geovibrio | Geovibrio sp. L21-Ace-BES | 0.00003 |
| Deferribacteres | Deferribacterales | Mucispirillum | Mucispirillum schaedleri | 0.00029 |
| Deinococcus-Thermus | Deinococcales | Deinococcus | Deinococcus misasensis | 0.00001 |
| Deinococcus-Thermus | Deinococcales | Deinococcus | Deinococcus proteolyticus | 0.00003 |
| Deinococcus-Thermus | Deinococcales | Deinococcus | Deinococcus unclassified | 0.00004 |
| Deinococcus-Thermus | Deinococcales | Deinococcus | uncultured Deinococcus sp. | 0.00002 |
| Fibrobacteres | Chitinivibrionales | Chitinivibrio | Chitinivibrio alkaliphilus | 0.00007 |
| Fibrobacteres | Fibrobacterales | Fibrobacter | Fibrobacter succinogenes | 0.00013 |
| Firmicutes | Bacillales | Alicyclobacillus | Alicyclobacillus acidocaldarius | 0.00012 |
| Firmicutes | Bacillales | Alicyclobacillus | Alicyclobacillus hesperidum | 0.00002 |
| Firmicutes | Bacillales | Amphibacillus | Amphibacillus jilinensis | 0.00019 |
| Firmicutes | Bacillales | Amphibacillus | Amphibacillus xylanus | 0.00010 |
| Firmicutes | Bacillales | Aneurinibacillus | Aneurinibacillus aneurinilyticus | 0.00025 |
| Firmicutes | Bacillales | Aneurinibacillus | Aneurinibacillus migulanus | 0.00041 |
| Firmicutes | Bacillales | Aneurinibacillus | Aneurinibacillus terranovensis | 0.00089 |
| Firmicutes | Bacillales | Anoxybacillus | Anoxybacillus ayderensis | 0.00004 |
| Firmicutes | Bacillales | Anoxybacillus | Anoxybacillus flavithermus | 0.00016 |
| Firmicutes | Bacillales | Anoxybacillus | Anoxybacillus gonensis | 0.00006 |
| Firmicutes | Bacillales | Anoxybacillus | Anoxybacillus sp. ATCC BAA-2555 | 0.00044 |
| Firmicutes | Bacillales | Anoxybacillus | Anoxybacillus sp. DT3-1 | 0.00003 |
| Firmicutes | Bacillales | Anoxybacillus | Anoxybacillus thermarum | 0.00004 |
| Firmicutes | Bacillales | Bacillaceae noname | Bacillaceae bacterium MTCC 10057 | 0.00002 |
| Firmicutes | Bacillales | Bacillaceae unclassified | Bacillaceae unclassified | 0.00008 |
| Firmicutes | Bacillales | Bacillales noname | Geomicrobium sp. JCM 19037 | 0.00009 |
| Firmicutes | Bacillales | Bacillales unclassified | Bacillales unclassified | 0.00069 |
| Firmicutes | Bacillales | Bacillus | Bacillus acidiproducens | 0.00003 |
| Firmicutes | Bacillales | Bacillus | Bacillus akibai | 0.00001 |
| Firmicutes | Bacillales | Bacillus | Bacillus alcalophilus | 0.00003 |
| Firmicutes | Bacillales | Bacillus | Bacillus altitudinis | 0.00006 |
| Firmicutes | Bacillales | Bacillus | Bacillus amyloliquefaciens | 0.00102 |
| Firmicutes | Bacillales | Bacillus | Bacillus anthracis | 0.00001 |
| Firmicutes | Bacillales | Bacillus | Bacillus aquimaris | 0.00002 |
| Firmicutes | Bacillales | Bacillus | Bacillus atrophaeus | 0.00001 |
| Firmicutes | Bacillales | Bacillus | Bacillus aurantiacus | 0.00066 |
| Firmicutes | Bacillales | Bacillus | Bacillus azotoformans | 0.00837 |
| Firmicutes | Bacillales | Bacillus | Bacillus badius | 0.00543 |
| Firmicutes | Bacillales | Bacillus | Bacillus bogoriensis | 0.01236 |
| Firmicutes | Bacillales | Bacillus | Bacillus bombysepticus | 0.00001 |
| Firmicutes | Bacillales | Bacillus | Bacillus boroniphilus | 0.00007 |
| Firmicutes | Bacillales | Bacillus | Bacillus cellulosilyticus | 0.00013 |
| Firmicutes | Bacillales | Bacillus | Bacillus cereus | 0.00775 |
| Firmicutes | Bacillales | Bacillus | Bacillus clausii | 0.00015 |
| Firmicutes | Bacillales | Bacillus | Bacillus coagulans | 0.00021 |
| Firmicutes | Bacillales | Bacillus | Bacillus coahuilensis | 0.00002 |
| Firmicutes | Bacillales | Bacillus | Bacillus cytotoxicus | 0.00004 |
| Firmicutes | Bacillales | Bacillus | Bacillus endophyticus | 0.00007 |
| Firmicutes | Bacillales | Bacillus | Bacillus firmus | 0.00003 |
| Firmicutes | Bacillales | Bacillus | Bacillus flexus | 0.00004 |
| Firmicutes | Bacillales | Bacillus | Bacillus fordii | 0.00001 |
| Firmicutes | Bacillales | Bacillus | Bacillus gaemokensis | 0.00005 |
| Firmicutes | Bacillales | Bacillus | Bacillus ginsengihumi | 0.00020 |
| Firmicutes | Bacillales | Bacillus | Bacillus halodurans | 0.00010 |
| Firmicutes | Bacillales | Bacillus | Bacillus hemicellulosilyticus | 0.00065 |
| Firmicutes | Bacillales | Bacillus | Bacillus kribbensis | 0.00032 |
| Firmicutes | Bacillales | Bacillus | Bacillus lehensis | 0.00002 |
| Firmicutes | Bacillales | Bacillus | Bacillus licheniformis | 0.00041 |
| Firmicutes | Bacillales | Bacillus | Bacillus manliponensis | 0.00005 |
| Firmicutes | Bacillales | Bacillus | Bacillus marmarensis | 0.00002 |
| Firmicutes | Bacillales | Bacillus | Bacillus massilioanorexius | 0.00019 |
| Firmicutes | Bacillales | Bacillus | Bacillus massiliosenegalensis | 0.00155 |
| Firmicutes | Bacillales | Bacillus | Bacillus megaterium | 0.00284 |
| Firmicutes | Bacillales | Bacillus | Bacillus methanolicus | 0.00012 |
| Firmicutes | Bacillales | Bacillus | Bacillus mycoides | 0.00067 |
| Firmicutes | Bacillales | Bacillus | Bacillus nealsonii | 0.00029 |
| Firmicutes | Bacillales | Bacillus | Bacillus niacini | 0.00485 |
| Firmicutes | Bacillales | Bacillus | Bacillus oceanisediminis | 0.00011 |
| Firmicutes | Bacillales | Bacillus | Bacillus okhensis | 0.00002 |
| Firmicutes | Bacillales | Bacillus | Bacillus panaciterrae | 0.00007 |
| Firmicutes | Bacillales | Bacillus | Bacillus pseudofirmus | 0.00002 |
| Firmicutes | Bacillales | Bacillus | Bacillus pumilus | 0.00035 |
| Firmicutes | Bacillales | Bacillus | Bacillus siamensis | 0.00003 |
| Firmicutes | Bacillales | Bacillus | Bacillus simplex | 0.00019 |
| Firmicutes | Bacillales | Bacillus | Bacillus sonorensis | 0.00241 |
| Firmicutes | Bacillales | Bacillus | Bacillus sp. 171095 106 | 0.00006 |
| Firmicutes | Bacillales | Bacillus | Bacillus sp. 1NLA3E | 0.00007 |
| Firmicutes | Bacillales | Bacillus | Bacillus sp. 2 A 57 CT2 | 0.00004 |
| Firmicutes | Bacillales | Bacillus | Bacillus sp. 278922 107 | 0.00002 |
| Firmicutes | Bacillales | Bacillus | Bacillus sp. 37MA | 0.00004 |
| Firmicutes | Bacillales | Bacillus | Bacillus sp. 72 | 0.00214 |
| Firmicutes | Bacillales | Bacillus | Bacillus sp. B-jedd | 0.00026 |
| Firmicutes | Bacillales | Bacillus | Bacillus sp. BS-01 | 0.00002 |
| Firmicutes | Bacillales | Bacillus | Bacillus sp. BS-02 | 0.00004 |
| Firmicutes | Bacillales | Bacillus | Bacillus sp. CAG:988 | 0.00009 |
| Firmicutes | Bacillales | Bacillus | Bacillus sp. EB01 | 0.00002 |
| Firmicutes | Bacillales | Bacillus | Bacillus sp. EGD-AK10 | 0.00016 |
| Firmicutes | Bacillales | Bacillus | Bacillus sp. FF3 | 0.00003 |
| Firmicutes | Bacillales | Bacillus | Bacillus sp. FF4 | 0.00007 |
| Firmicutes | Bacillales | Bacillus | Bacillus sp. FJAT-13831 | 0.00009 |
| Firmicutes | Bacillales | Bacillus | Bacillus sp. FJAT-14515 | 0.00024 |
| Firmicutes | Bacillales | Bacillus | Bacillus sp. FJAT-14578 | 0.00064 |
| Firmicutes | Bacillales | Bacillus | Bacillus sp. G2(2012b) | 0.00013 |
| Firmicutes | Bacillales | Bacillus | Bacillus sp. HJ171 | 0.00007 |
| Firmicutes | Bacillales | Bacillus | Bacillus sp. J13 | 0.00006 |
| Firmicutes | Bacillales | Bacillus | Bacillus sp. J33 | 0.00002 |
| Firmicutes | Bacillales | Bacillus | Bacillus sp. J37 | 0.00284 |
| Firmicutes | Bacillales | Bacillus | Bacillus sp. JGI 001011-F15 | 0.00003 |
| Firmicutes | Bacillales | Bacillus | Bacillus sp. KW-12 | 0.00141 |
| Firmicutes | Bacillales | Bacillus | Bacillus sp. L1(2012) | 0.00003 |
| Firmicutes | Bacillales | Bacillus | Bacillus sp. m3-13 | 0.00005 |
| Firmicutes | Bacillales | Bacillus | Bacillus sp. MB2021 | 0.00018 |
| Firmicutes | Bacillales | Bacillus | Bacillus sp. MSP13 | 0.00176 |
| Firmicutes | Bacillales | Bacillus | Bacillus sp. MT2 | 0.00011 |
| Firmicutes | Bacillales | Bacillus | Bacillus sp. NRRL B-14911 | 0.00086 |
| Firmicutes | Bacillales | Bacillus | Bacillus sp. OxB-1 | 0.00003 |
| Firmicutes | Bacillales | Bacillus | Bacillus sp. REN51N | 0.00115 |
| Firmicutes | Bacillales | Bacillus | Bacillus sp. SG-1 | 0.00003 |
| Firmicutes | Bacillales | Bacillus | Bacillus sp. TS-2 | 0.00017 |
| Firmicutes | Bacillales | Bacillus | Bacillus sp. UNC322MFChir4.1 | 0.00021 |
| Firmicutes | Bacillales | Bacillus | Bacillus sp. UNC41MFS5 | 0.00044 |
| Firmicutes | Bacillales | Bacillus | Bacillus sp. UNC437CL72CviS29 | 0.00002 |
| Firmicutes | Bacillales | Bacillus | Bacillus sp. UNC438CL73TsuS30 | 0.00103 |
| Firmicutes | Bacillales | Bacillus | Bacillus sp. URHB0009 | 0.00020 |
| Firmicutes | Bacillales | Bacillus | Bacillus sp. ZYK | 0.00026 |
| Firmicutes | Bacillales | Bacillus | Bacillus subterraneus | 0.00030 |
| Firmicutes | Bacillales | Bacillus | Bacillus subtilis | 0.00129 |
| Firmicutes | Bacillales | Bacillus | Bacillus thermoamylovorans | 0.00121 |
| Firmicutes | Bacillales | Bacillus | Bacillus thermotolerans | 0.00001 |
| Firmicutes | Bacillales | Bacillus | Bacillus thuringiensis | 0.00026 |
| Firmicutes | Bacillales | Bacillus | Bacillus timonensis | 0.00018 |
| Firmicutes | Bacillales | Bacillus | Bacillus unclassified | 0.00287 |
| Firmicutes | Bacillales | Bacillus | Bacillus vallismortis | 0.00006 |
| Firmicutes | Bacillales | Bacillus | Bacillus vireti | 0.00008 |
| Firmicutes | Bacillales | Bacillus | Bacillus wakoensis | 0.00008 |
| Firmicutes | Bacillales | Bacillus | Bacillus weihenstephanensis | 0.00002 |
| Firmicutes | Bacillales | Bhargavaea | Bhargavaea cecembensis | 0.00035 |
| Firmicutes | Bacillales | Brevibacillus | Brevibacillus borstelensis | 0.00015 |
| Firmicutes | Bacillales | Brevibacillus | Brevibacillus brevis | 0.00002 |
| Firmicutes | Bacillales | Brevibacillus | Brevibacillus laterosporus | 0.00025 |
| Firmicutes | Bacillales | Brevibacillus | Brevibacillus massiliensis | 0.00005 |
| Firmicutes | Bacillales | Brevibacillus | Brevibacillus panacihumi | 0.00004 |
| Firmicutes | Bacillales | Brevibacillus | Brevibacillus unclassified | 0.00002 |
| Firmicutes | Bacillales | Brochothrix | Brochothrix campestris | 0.00002 |
| Firmicutes | Bacillales | Brochothrix | Brochothrix thermosphacta | 0.00003 |
| Firmicutes | Bacillales | Caldalkalibacillus | Caldalkalibacillus thermarum | 0.00004 |
| Firmicutes | Bacillales | Caldibacillus | Caldibacillus debilis | 0.00006 |
| Firmicutes | Bacillales | Cohnella | Cohnella panacarvi | 0.00006 |
| Firmicutes | Bacillales | Domibacillus | Domibacillus enclensis | 0.00006 |
| Firmicutes | Bacillales | Domibacillus | Domibacillus indicus | 0.00326 |
| Firmicutes | Bacillales | Domibacillus | Domibacillus sp. PAMC 80007 | 0.00003 |
| Firmicutes | Bacillales | Effusibacillus | Effusibacillus pohliae | 0.00002 |
| Firmicutes | Bacillales | Exiguobacterium | Exiguobacterium alkaliphilum | 0.00005 |
| Firmicutes | Bacillales | Exiguobacterium | Exiguobacterium marinum | 0.00003 |
| Firmicutes | Bacillales | Exiguobacterium | Exiguobacterium mexicanum | 0.00002 |
| Firmicutes | Bacillales | Exiguobacterium | Exiguobacterium pavilionensis | 0.00030 |
| Firmicutes | Bacillales | Exiguobacterium | Exiguobacterium sibiricum | 0.00003 |
| Firmicutes | Bacillales | Exiguobacterium | Exiguobacterium sp. AB2 | 0.00008 |
| Firmicutes | Bacillales | Exiguobacterium | Exiguobacterium sp. S17 | 0.00004 |
| Firmicutes | Bacillales | Exiguobacterium | Exiguobacterium undae | 0.00007 |
| Firmicutes | Bacillales | Gemella | Gemella bergeri | 0.00640 |
| Firmicutes | Bacillales | Gemella | Gemella cuniculi | 0.00838 |
| Firmicutes | Bacillales | Gemella | Gemella haemolysans | 0.01070 |
| Firmicutes | Bacillales | Gemella | Gemella morbillorum | 0.00796 |
| Firmicutes | Bacillales | Gemella | Gemella sanguinis | 0.00826 |
| Firmicutes | Bacillales | Gemella | Gemella unclassified | 0.00059 |
| Firmicutes | Bacillales | Geobacillus | Geobacillus caldoxylosilyticus | 0.00003 |
| Firmicutes | Bacillales | Geobacillus | Geobacillus kaustophilus | 0.00130 |
| Firmicutes | Bacillales | Geobacillus | Geobacillus sp. A8 | 0.00053 |
| Firmicutes | Bacillales | Geobacillus | Geobacillus sp. C56-T3 | 0.00003 |
| Firmicutes | Bacillales | Geobacillus | Geobacillus sp. G11MC16 | 0.00002 |
| Firmicutes | Bacillales | Geobacillus | Geobacillus sp. G1w1 | 0.00748 |
| Firmicutes | Bacillales | Geobacillus | Geobacillus sp. MO-1 | 0.00010 |
| Firmicutes | Bacillales | Geobacillus | Geobacillus stearothermophilus | 0.00009 |
| Firmicutes | Bacillales | Geobacillus | Geobacillus thermoglucosidasius | 0.00009 |
| Firmicutes | Bacillales | Geobacillus | Geobacillus unclassified | 0.00004 |
| Firmicutes | Bacillales | Halobacillus | Halobacillus dabanensis | 0.00002 |
| Firmicutes | Bacillales | Halobacillus | Halobacillus halophilus | 0.00018 |
| Firmicutes | Bacillales | Halobacillus | Halobacillus sp. BBL2006 | 0.00007 |
| Firmicutes | Bacillales | Halobacillus | Halobacillus trueperi | 0.10049 |
| Firmicutes | Bacillales | Halobacillus | Halobacillus unclassified | 0.00014 |
| Firmicutes | Bacillales | Jeotgalibacillus | Jeotgalibacillus alimentarius | 0.00015 |
| Firmicutes | Bacillales | Jeotgalibacillus | Jeotgalibacillus sp. D5 | 0.00039 |
| Firmicutes | Bacillales | Jeotgalicoccus | Jeotgalicoccus psychrophilus | 0.00009 |
| Firmicutes | Bacillales | Kurthia | Kurthia huakuii | 0.00011 |
| Firmicutes | Bacillales | Kurthia | Kurthia massiliensis | 0.00003 |
| Firmicutes | Bacillales | Kurthia | Kurthia sp. Dielmo | 0.00003 |
| Firmicutes | Bacillales | Kurthia | Kurthia sp. JC8E | 0.00019 |
| Firmicutes | Bacillales | Laceyella | Laceyella sacchari | 0.00006 |
| Firmicutes | Bacillales | Listeria | Listeria aquatica | 0.00008 |
| Firmicutes | Bacillales | Listeria | Listeria booriae | 0.00009 |
| Firmicutes | Bacillales | Listeria | Listeria cornellensis | 0.00007 |
| Firmicutes | Bacillales | Listeria | Listeria fleischmannii | 0.00026 |
| Firmicutes | Bacillales | Listeria | Listeria floridensis | 0.00002 |
| Firmicutes | Bacillales | Listeria | Listeria grandensis | 0.00004 |
| Firmicutes | Bacillales | Listeria | Listeria grayi | 0.00002 |
| Firmicutes | Bacillales | Listeria | Listeria innocua | 0.00072 |
| Firmicutes | Bacillales | Listeria | Listeria ivanovii | 0.00015 |
| Firmicutes | Bacillales | Listeria | Listeria marthii | 0.00003 |
| Firmicutes | Bacillales | Listeria | Listeria monocytogenes | 0.00091 |
| Firmicutes | Bacillales | Listeria | Listeria newyorkensis | 0.00002 |
| Firmicutes | Bacillales | Listeria | Listeria riparia | 0.00002 |
| Firmicutes | Bacillales | Listeria | Listeria rocourtiae | 0.00010 |
| Firmicutes | Bacillales | Listeria | Listeria seeligeri | 0.00011 |
| Firmicutes | Bacillales | Listeria | Listeria weihenstephanensis | 0.00004 |
| Firmicutes | Bacillales | Listeria | Listeria welshimeri | 0.00002 |
| Firmicutes | Bacillales | Listeriaceae noname | Listeriaceae bacterium FSL A5-0209 | 0.00033 |
| Firmicutes | Bacillales | Lysinibacillus | Lysinibacillus fusiformis | 0.00028 |
| Firmicutes | Bacillales | Lysinibacillus | Lysinibacillus manganicus | 0.00039 |
| Firmicutes | Bacillales | Lysinibacillus | Lysinibacillus massiliensis | 0.00004 |
| Firmicutes | Bacillales | Lysinibacillus | Lysinibacillus odysseyi | 0.00002 |
| Firmicutes | Bacillales | Lysinibacillus | Lysinibacillus sinduriensis | 0.00002 |
| Firmicutes | Bacillales | Lysinibacillus | Lysinibacillus sp. 13S34 air | 0.00009 |
| Firmicutes | Bacillales | Lysinibacillus | Lysinibacillus sp. BF-4 | 0.00015 |
| Firmicutes | Bacillales | Lysinibacillus | Lysinibacillus sphaericus | 0.00130 |
| Firmicutes | Bacillales | Lysinibacillus | Lysinibacillus unclassified | 0.00009 |
| Firmicutes | Bacillales | Lysinibacillus | Lysinibacillus varians | 0.00188 |
| Firmicutes | Bacillales | Nosocomiicoccus | Nosocomiicoccus sp. NP2 | 0.00004 |
| Firmicutes | Bacillales | Oceanobacillus | Oceanobacillus kimchii | 0.00003 |
| Firmicutes | Bacillales | Oceanobacillus | Oceanobacillus massiliensis | 0.00008 |
| Firmicutes | Bacillales | Oceanobacillus | Oceanobacillus oncorhynchi | 0.00028 |
| Firmicutes | Bacillales | Oceanobacillus | Oceanobacillus picturae | 0.00004 |
| Firmicutes | Bacillales | Oceanobacillus | Oceanobacillus sp. S5 | 0.00052 |
| Firmicutes | Bacillales | Paenibacillaceae noname | Paenibacillaceae bacterium G5 | 0.00001 |
| Firmicutes | Bacillales | Paenibacillus | Paenibacillus alginolyticus | 0.00061 |
| Firmicutes | Bacillales | Paenibacillus | Paenibacillus alvei | 0.00125 |
| Firmicutes | Bacillales | Paenibacillus | Paenibacillus assamensis | 0.00004 |
| Firmicutes | Bacillales | Paenibacillus | Paenibacillus chitinolyticus | 0.00008 |
| Firmicutes | Bacillales | Paenibacillus | Paenibacillus daejeonensis | 0.00009 |
| Firmicutes | Bacillales | Paenibacillus | Paenibacillus darwinianus | 0.00003 |
| Firmicutes | Bacillales | Paenibacillus | Paenibacillus dauci | 0.00018 |
| Firmicutes | Bacillales | Paenibacillus | Paenibacillus dendritiformis | 0.00010 |
| Firmicutes | Bacillales | Paenibacillus | Paenibacillus durus | 0.00065 |
| Firmicutes | Bacillales | Paenibacillus | Paenibacillus ehimensis | 0.00002 |
| Firmicutes | Bacillales | Paenibacillus | Paenibacillus elgii | 0.00053 |
| Firmicutes | Bacillales | Paenibacillus | Paenibacillus ginsengihumi | 0.00003 |
| Firmicutes | Bacillales | Paenibacillus | Paenibacillus graminis | 0.00013 |
| Firmicutes | Bacillales | Paenibacillus | Paenibacillus harenae | 0.00009 |
| Firmicutes | Bacillales | Paenibacillus | Paenibacillus larvae | 0.00193 |
| Firmicutes | Bacillales | Paenibacillus | Paenibacillus macerans | 0.00038 |
| Firmicutes | Bacillales | Paenibacillus | Paenibacillus mucilaginosus | 0.00002 |
| Firmicutes | Bacillales | Paenibacillus | Paenibacillus pasadenensis | 0.00006 |
| Firmicutes | Bacillales | Paenibacillus | Paenibacillus peoriae | 0.00003 |
| Firmicutes | Bacillales | Paenibacillus | Paenibacillus pini | 0.00003 |
| Firmicutes | Bacillales | Paenibacillus | Paenibacillus polymyxa | 0.00985 |
| Firmicutes | Bacillales | Paenibacillus | Paenibacillus riograndensis | 0.00057 |
| Firmicutes | Bacillales | Paenibacillus | Paenibacillus sanguinis | 0.00002 |
| Firmicutes | Bacillales | Paenibacillus | Paenibacillus senegalensis | 0.00004 |
| Firmicutes | Bacillales | Paenibacillus | Paenibacillus sonchi | 0.00062 |
| Firmicutes | Bacillales | Paenibacillus | Paenibacillus sophorae | 0.00026 |
| Firmicutes | Bacillales | Paenibacillus | Paenibacillus sp. 1-49 | 0.00029 |
| Firmicutes | Bacillales | Paenibacillus | Paenibacillus sp. D9 | 0.00001 |
| Firmicutes | Bacillales | Paenibacillus | Paenibacillus sp. E194 | 0.00004 |
| Firmicutes | Bacillales | Paenibacillus | Paenibacillus sp. FSL H7-0357 | 0.00006 |
| Firmicutes | Bacillales | Paenibacillus | Paenibacillus sp. FSL H7-0737 | 0.00043 |
| Firmicutes | Bacillales | Paenibacillus | Paenibacillus sp. FSL H8-237 | 0.00002 |
| Firmicutes | Bacillales | Paenibacillus | Paenibacillus sp. FSL R5-0345 | 0.00006 |
| Firmicutes | Bacillales | Paenibacillus | Paenibacillus sp. FSL R7-0331 | 0.00156 |
| Firmicutes | Bacillales | Paenibacillus | Paenibacillus sp. FSL R7-269 | 0.00005 |
| Firmicutes | Bacillales | Paenibacillus | Paenibacillus sp. GD11 | 0.00009 |
| Firmicutes | Bacillales | Paenibacillus | Paenibacillus sp. HGF5 | 0.00019 |
| Firmicutes | Bacillales | Paenibacillus | Paenibacillus sp. HW567 | 0.00001 |
| Firmicutes | Bacillales | Paenibacillus | Paenibacillus sp. IHB B 3415 | 0.00586 |
| Firmicutes | Bacillales | Paenibacillus | Paenibacillus sp. IHBB 10380 | 0.00174 |
| Firmicutes | Bacillales | Paenibacillus | Paenibacillus sp. JDR-2 | 0.00007 |
| Firmicutes | Bacillales | Paenibacillus | Paenibacillus sp. MSt1 | 0.00005 |
| Firmicutes | Bacillales | Paenibacillus | Paenibacillus sp. oral taxon 786 | 0.00008 |
| Firmicutes | Bacillales | Paenibacillus | Paenibacillus sp. OSY-SE | 0.00005 |
| Firmicutes | Bacillales | Paenibacillus | Paenibacillus sp. P1XP2 | 0.00002 |
| Firmicutes | Bacillales | Paenibacillus | Paenibacillus sp. P22 | 0.00344 |
| Firmicutes | Bacillales | Paenibacillus | Paenibacillus sp. TCA20 | 0.00103 |
| Firmicutes | Bacillales | Paenibacillus | Paenibacillus sp. UNC217MF | 0.00007 |
| Firmicutes | Bacillales | Paenibacillus | Paenibacillus sp. UNC451MF | 0.00003 |
| Firmicutes | Bacillales | Paenibacillus | Paenibacillus sp. URHA0014 | 0.00004 |
| Firmicutes | Bacillales | Paenibacillus | Paenibacillus sp. VKM B-2647 | 0.00014 |
| Firmicutes | Bacillales | Paenibacillus | Paenibacillus sp. Y412MC10 | 0.00003 |
| Firmicutes | Bacillales | Paenibacillus | Paenibacillus terrae | 0.00015 |
| Firmicutes | Bacillales | Paenibacillus | Paenibacillus terrigena | 0.00005 |
| Firmicutes | Bacillales | Paenibacillus | Paenibacillus unclassified | 0.00031 |
| Firmicutes | Bacillales | Paenibacillus | Paenibacillus wynnii | 0.00097 |
| Firmicutes | Bacillales | Paenisporosarcina | Paenisporosarcina sp. HGH0030 | 0.00003 |
| Firmicutes | Bacillales | Paenisporosarcina | Paenisporosarcina sp. TG-14 | 0.00006 |
| Firmicutes | Bacillales | Planococcus | Planococcus antarcticus | 0.00005 |
| Firmicutes | Bacillales | Planococcus | Planococcus citreus | 0.00001 |
| Firmicutes | Bacillales | Planococcus | Planococcus donghaensis | 0.00002 |
| Firmicutes | Bacillales | Planococcus | Planococcus halocryophilus | 0.00002 |
| Firmicutes | Bacillales | Planococcus | Planococcus sp. CAU13 | 0.00005 |
| Firmicutes | Bacillales | Planococcus | Planococcus sp. PAMC 21323 | 0.00012 |
| Firmicutes | Bacillales | Planomicrobium | Planomicrobium sp. ES2 | 0.00006 |
| Firmicutes | Bacillales | Pontibacillus | Pontibacillus chungwhensis | 0.00017 |
| Firmicutes | Bacillales | Pontibacillus | Pontibacillus litoralis | 0.00963 |
| Firmicutes | Bacillales | Pontibacillus | Pontibacillus marinus | 0.00079 |
| Firmicutes | Bacillales | Pontibacillus | Pontibacillus yanchengensis | 0.00003 |
| Firmicutes | Bacillales | Saccharibacillus | Saccharibacillus kuerlensis | 0.00005 |
| Firmicutes | Bacillales | Saccharibacillus | Saccharibacillus sacchari | 0.00008 |
| Firmicutes | Bacillales | Salimicrobium | Salimicrobium jeotgali | 0.00029 |
| Firmicutes | Bacillales | Salinicoccus | Salinicoccus carnicancri | 0.00003 |
| Firmicutes | Bacillales | Salinicoccus | Salinicoccus luteus | 0.00002 |
| Firmicutes | Bacillales | Salinicoccus | Salinicoccus roseus | 0.00005 |
| Firmicutes | Bacillales | Salsuginibacillus | Salsuginibacillus kocurii | 0.00009 |
| Firmicutes | Bacillales | Sediminibacillus | Sediminibacillus halophilus | 0.00008 |
| Firmicutes | Bacillales | Shimazuella | Shimazuella kribbensis | 0.00019 |
| Firmicutes | Bacillales | Sporolactobacillaceae noname | [Bacillus] selenitireducens | 0.00069 |
| Firmicutes | Bacillales | Sporolactobacillus | Sporolactobacillus terrae | 0.00012 |
| Firmicutes | Bacillales | Sporolactobacillus | Sporolactobacillus vineae | 0.00004 |
| Firmicutes | Bacillales | Sporosarcina | Sporosarcina newyorkensis | 0.00036 |
| Firmicutes | Bacillales | Sporosarcina | Sporosarcina sp. D27 | 0.00001 |
| Firmicutes | Bacillales | Sporosarcina | Sporosarcina sp. ZBG7A | 0.00008 |
| Firmicutes | Bacillales | Staphylococcus | Staphylococcus aureus | 0.00369 |
| Firmicutes | Bacillales | Staphylococcus | Staphylococcus capitis | 0.00007 |
| Firmicutes | Bacillales | Staphylococcus | Staphylococcus caprae | 0.00008 |
| Firmicutes | Bacillales | Staphylococcus | Staphylococcus carnosus | 0.00002 |
| Firmicutes | Bacillales | Staphylococcus | Staphylococcus chromogenes | 0.00036 |
| Firmicutes | Bacillales | Staphylococcus | Staphylococcus delphini | 0.00003 |
| Firmicutes | Bacillales | Staphylococcus | Staphylococcus epidermidis | 0.00038 |
| Firmicutes | Bacillales | Staphylococcus | Staphylococcus haemolyticus | 0.00003 |
| Firmicutes | Bacillales | Staphylococcus | Staphylococcus lentus | 0.00002 |
| Firmicutes | Bacillales | Staphylococcus | Staphylococcus massiliensis | 0.00002 |
| Firmicutes | Bacillales | Staphylococcus | Staphylococcus microti | 0.00006 |
| Firmicutes | Bacillales | Staphylococcus | Staphylococcus saprophyticus | 0.00007 |
| Firmicutes | Bacillales | Staphylococcus | Staphylococcus simulans | 0.00005 |
| Firmicutes | Bacillales | Staphylococcus | Staphylococcus sp. AL1 | 0.00005 |
| Firmicutes | Bacillales | Staphylococcus | Staphylococcus sp. M0480 | 0.00101 |
| Firmicutes | Bacillales | Staphylococcus | Staphylococcus unclassified | 0.00011 |
| Firmicutes | Bacillales | Staphylococcus | Staphylococcus vitulinus | 0.00003 |
| Firmicutes | Bacillales | Staphylococcus | Staphylococcus warneri | 0.00069 |
| Firmicutes | Bacillales | Staphylococcus | Staphylococcus xylosus | 0.00011 |
| Firmicutes | Bacillales | Thermicanus | Thermicanus aegyptius | 0.00443 |
| Firmicutes | Bacillales | Thermoactinomyces | Thermoactinomyces daqus | 0.00006 |
| Firmicutes | Bacillales | Thermoactinomycetaceae noname | Thermoactinomycetaceae bacterium GD1 | 0.00011 |
| Firmicutes | Bacillales | Tumebacillus | Tumebacillus flagellatus | 0.00008 |
| Firmicutes | Bacillales | Virgibacillus | Virgibacillus halodenitrificans | 0.00051 |
| Firmicutes | Bacillales | Virgibacillus | Virgibacillus sp. SK37 | 0.00124 |
| Firmicutes | Bacillales | Virgibacillus | Virgibacillus sp. Vm-5 | 0.00017 |
| Firmicutes | Bacillales | Viridibacillus | Viridibacillus arenosi | 0.00294 |
| Firmicutes | Bacilli unclassified | Bacilli unclassified | Bacilli unclassified | 0.01283 |
| Firmicutes | Clostridiales | Acetivibrio | Acetivibrio cellulolyticus | 0.00050 |
| Firmicutes | Clostridiales | Acetobacterium | Acetobacterium dehalogenans | 0.00036 |
| Firmicutes | Clostridiales | Acetobacterium | Acetobacterium woodii | 0.00373 |
| Firmicutes | Clostridiales | Alkaliphilus | Alkaliphilus metalliredigens | 0.00057 |
| Firmicutes | Clostridiales | Alkaliphilus | Alkaliphilus oremlandii | 0.00012 |
| Firmicutes | Clostridiales | Alkaliphilus | Alkaliphilus transvaalensis | 0.00022 |
| Firmicutes | Clostridiales | Anaerococcus | Anaerococcus hydrogenalis | 0.00017 |
| Firmicutes | Clostridiales | Anaerococcus | Anaerococcus lactolyticus | 0.00033 |
| Firmicutes | Clostridiales | Anaerococcus | Anaerococcus obesiensis | 0.00437 |
| Firmicutes | Clostridiales | Anaerococcus | Anaerococcus pacaensis | 0.01549 |
| Firmicutes | Clostridiales | Anaerococcus | Anaerococcus prevotii | 0.00018 |
| Firmicutes | Clostridiales | Anaerococcus | Anaerococcus senegalensis | 0.00013 |
| Firmicutes | Clostridiales | Anaerococcus | Anaerococcus sp. 9402080 | 0.00050 |
| Firmicutes | Clostridiales | Anaerococcus | Anaerococcus sp. PH9 | 0.00400 |
| Firmicutes | Clostridiales | Anaerococcus | Anaerococcus tetradius | 0.00040 |
| Firmicutes | Clostridiales | Anaerococcus | Anaerococcus unclassified | 0.00001 |
| Firmicutes | Clostridiales | Anaerococcus | Anaerococcus vaginalis | 0.00016 |
| Firmicutes | Clostridiales | Anaerofustis | Anaerofustis stercorihominis | 0.01534 |
| Firmicutes | Clostridiales | Anaerosalibacter | Anaerosalibacter sp. ND1 | 0.00817 |
| Firmicutes | Clostridiales | Anaerostipes | Anaerostipes caccae | 0.00480 |
| Firmicutes | Clostridiales | Anaerostipes | Anaerostipes hadrus | 0.02664 |
| Firmicutes | Clostridiales | Anaerostipes | Anaerostipes sp. 3 2 56FAA | 0.00027 |
| Firmicutes | Clostridiales | Anaerostipes | Anaerostipes sp. CAG:276 | 0.00626 |
| Firmicutes | Clostridiales | Anaerostipes | Anaerostipes unclassified | 0.00604 |
| Firmicutes | Clostridiales | Anaerotruncus | Anaerotruncus colihominis | 0.00640 |
| Firmicutes | Clostridiales | Anaerotruncus | Anaerotruncus sp. CAG:390 | 0.00178 |
| Firmicutes | Clostridiales | Anaerotruncus | Anaerotruncus sp. CAG:528 | 0.01190 |
| Firmicutes | Clostridiales | Anaerotruncus | Anaerotruncus sp. G3(2012) | 0.00973 |
| Firmicutes | Clostridiales | Anaerovorax | Anaerovorax odorimutans | 0.00137 |
| Firmicutes | Clostridiales | Blautia | [Ruminococcus] gnavus | 2.19196 |
| Firmicutes | Clostridiales | Blautia | [Ruminococcus] obeum | 0.04446 |
| Firmicutes | Clostridiales | Blautia | [Ruminococcus] torques | 0.07765 |
| Firmicutes | Clostridiales | Blautia | Blautia hansenii | 0.43164 |
| Firmicutes | Clostridiales | Blautia | Blautia hydrogenotrophica | 0.01803 |
| Firmicutes | Clostridiales | Blautia | Blautia hydrogenotrophica CAG:147 | 0.02101 |
| Firmicutes | Clostridiales | Blautia | Blautia producta | 0.04451 |
| Firmicutes | Clostridiales | Blautia | Blautia schinkii | 0.08462 |
| Firmicutes | Clostridiales | Blautia | Blautia sp. CAG:237 | 0.02363 |
| Firmicutes | Clostridiales | Blautia | Blautia sp. CAG:257 | 0.69302 |
| Firmicutes | Clostridiales | Blautia | Blautia sp. CAG:37 | 0.00227 |
| Firmicutes | Clostridiales | Blautia | Blautia sp. CAG:52 | 0.00770 |
| Firmicutes | Clostridiales | Blautia | Blautia sp. KLE 1732 | 0.01547 |
| Firmicutes | Clostridiales | Blautia | Blautia unclassified | 0.01721 |
| Firmicutes | Clostridiales | Blautia | Blautia wexlerae | 0.07108 |
| Firmicutes | Clostridiales | Butyricicoccus | Butyricicoccus pullicaecorum | 0.00779 |
| Firmicutes | Clostridiales | Butyrivibrio | Butyrivibrio crossotus | 0.01020 |
| Firmicutes | Clostridiales | Butyrivibrio | Butyrivibrio crossotus CAG:259 | 0.00048 |
| Firmicutes | Clostridiales | Butyrivibrio | Butyrivibrio fibrisolvens | 0.01862 |
| Firmicutes | Clostridiales | Butyrivibrio | Butyrivibrio hungatei | 0.00003 |
| Firmicutes | Clostridiales | Butyrivibrio | Butyrivibrio proteoclasticus | 0.00615 |
| Firmicutes | Clostridiales | Butyrivibrio | Butyrivibrio sp. AC2005 | 0.00147 |
| Firmicutes | Clostridiales | Butyrivibrio | Butyrivibrio sp. AD3002 | 0.00021 |
| Firmicutes | Clostridiales | Butyrivibrio | Butyrivibrio sp. AE2005 | 0.00017 |
| Firmicutes | Clostridiales | Butyrivibrio | Butyrivibrio sp. AE2015 | 0.00040 |
| Firmicutes | Clostridiales | Butyrivibrio | Butyrivibrio sp. AE2032 | 0.00074 |
| Firmicutes | Clostridiales | Butyrivibrio | Butyrivibrio sp. AE3003 | 0.00549 |
| Firmicutes | Clostridiales | Butyrivibrio | Butyrivibrio sp. AE3004 | 0.01212 |
| Firmicutes | Clostridiales | Butyrivibrio | Butyrivibrio sp. AE3006 | 0.00005 |
| Firmicutes | Clostridiales | Butyrivibrio | Butyrivibrio sp. AE3009 | 0.01125 |
| Firmicutes | Clostridiales | Butyrivibrio | Butyrivibrio sp. CAG:318 | 0.00309 |
| Firmicutes | Clostridiales | Butyrivibrio | Butyrivibrio sp. FC2001 | 0.00021 |
| Firmicutes | Clostridiales | Butyrivibrio | Butyrivibrio sp. FCS006 | 0.00017 |
| Firmicutes | Clostridiales | Butyrivibrio | Butyrivibrio sp. FCS014 | 0.01722 |
| Firmicutes | Clostridiales | Butyrivibrio | Butyrivibrio sp. LB2008 | 0.00519 |
| Firmicutes | Clostridiales | Butyrivibrio | Butyrivibrio sp. LC3010 | 0.00690 |
| Firmicutes | Clostridiales | Butyrivibrio | Butyrivibrio sp. MB2005 | 0.00072 |
| Firmicutes | Clostridiales | Butyrivibrio | Butyrivibrio sp. MC2013 | 0.00054 |
| Firmicutes | Clostridiales | Butyrivibrio | Butyrivibrio sp. NC2002 | 0.00084 |
| Firmicutes | Clostridiales | Butyrivibrio | Butyrivibrio sp. NC2007 | 0.00638 |
| Firmicutes | Clostridiales | Butyrivibrio | Butyrivibrio sp. NC3005 | 0.03189 |
| Firmicutes | Clostridiales | Butyrivibrio | Butyrivibrio sp. VCB2001 | 0.00685 |
| Firmicutes | Clostridiales | Butyrivibrio | Butyrivibrio sp. VCB2006 | 0.00009 |
| Firmicutes | Clostridiales | Butyrivibrio | Butyrivibrio sp. VCD2006 | 0.00577 |
| Firmicutes | Clostridiales | Butyrivibrio | Butyrivibrio sp. WCD2001 | 0.00134 |
| Firmicutes | Clostridiales | Butyrivibrio | Butyrivibrio sp. WCD3002 | 0.00857 |
| Firmicutes | Clostridiales | Butyrivibrio | Butyrivibrio sp. WCE2006 | 0.00040 |
| Firmicutes | Clostridiales | Butyrivibrio | Butyrivibrio sp. XBB1001 | 0.00129 |
| Firmicutes | Clostridiales | Butyrivibrio | Butyrivibrio sp. XPD2002 | 0.00004 |
| Firmicutes | Clostridiales | Butyrivibrio | Butyrivibrio sp. XPD2006 | 0.00019 |
| Firmicutes | Clostridiales | Butyrivibrio | Butyrivibrio unclassified | 0.00487 |
| Firmicutes | Clostridiales | Caldicoprobacter | Caldicoprobacter oshimai | 0.00027 |
| Firmicutes | Clostridiales | Caldisalinibacter | Caldisalinibacter kiritimatiensis | 0.00120 |
| Firmicutes | Clostridiales | Caloramator | Caloramator australicus | 0.00122 |
| Firmicutes | Clostridiales | Caloramator | Caloramator sp. ALD01 | 0.00121 |
| Firmicutes | Clostridiales | Caloranaerobacter | Caloranaerobacter azorensis | 0.00130 |
| Firmicutes | Clostridiales | Candidatus Arthromitus | Candidatus Arthromitus sp. SFB-mouse | 0.00112 |
| Firmicutes | Clostridiales | Candidatus Arthromitus | Candidatus Arthromitus sp. SFB-mouse-NL | 0.00042 |
| Firmicutes | Clostridiales | Candidatus Arthromitus | Candidatus Arthromitus sp. SFB-rat-Yit | 0.00019 |
| Firmicutes | Clostridiales | Candidatus Arthromitus | Candidatus Arthromitus unclassified | 0.00221 |
| Firmicutes | Clostridiales | Candidatus Soleaferrea | Candidatus Soleaferrea massiliensis | 0.00854 |
| Firmicutes | Clostridiales | Carboxydocella | uncultured Carboxydocella sp. | 0.00009 |
| Firmicutes | Clostridiales | Catonella | Catonella morbi | 0.02392 |
| Firmicutes | Clostridiales | Cellulosilyticum | Cellulosilyticum lentocellum | 0.00461 |
| Firmicutes | Clostridiales | Clostridiaceae noname | Clostridiaceae bacterium BRH c20a | 0.00005 |
| Firmicutes | Clostridiales | Clostridiaceae noname | Clostridiaceae bacterium GM1 | 0.00245 |
| Firmicutes | Clostridiales | Clostridiaceae noname | Clostridiaceae bacterium MS3 | 0.00550 |
| Firmicutes | Clostridiales | Clostridiaceae unclassified | Clostridiaceae unclassified | 0.00018 |
| Firmicutes | Clostridiales | Clostridiales Family XI. Incertae Sedis noname | Clostridiales bacterium S5-A11 | 0.00038 |
| Firmicutes | Clostridiales | Clostridiales Family XI. Incertae Sedis noname | Clostridiales bacterium S7-1-4 | 0.00054 |
| Firmicutes | Clostridiales | Clostridiales Family XIII. Incertae Sedis noname | [Eubacterium] infirmum | 0.01801 |
| Firmicutes | Clostridiales | Clostridiales Family XIII. Incertae Sedis noname | [Eubacterium] nodatum | 0.00107 |
| Firmicutes | Clostridiales | Clostridiales Family XIII. Incertae Sedis noname | [Eubacterium] sulci | 0.01614 |
| Firmicutes | Clostridiales | Clostridiales Family XIII. Incertae Sedis noname | Clostridiales bacterium S5-A14a | 0.00093 |
| Firmicutes | Clostridiales | Clostridiales noname | [Bacteroides] pectinophilus | 0.00224 |
| Firmicutes | Clostridiales | Clostridiales noname | butyrate-producing bacterium SM4/1 | 0.00254 |
| Firmicutes | Clostridiales | Clostridiales noname | butyrate-producing bacterium SS3/4 | 0.01380 |
| Firmicutes | Clostridiales | Clostridiales noname | butyrate-producing bacterium SSC/2 | 0.01338 |
| Firmicutes | Clostridiales | Clostridiales noname | Clostridiales bacterium 1 7 47FAA | 0.01170 |
| Firmicutes | Clostridiales | Clostridiales noname | Clostridiales bacterium 9401234 | 0.00049 |
| Firmicutes | Clostridiales | Clostridiales noname | Clostridiales bacterium 9403326 | 0.00015 |
| Firmicutes | Clostridiales | Clostridiales noname | Clostridiales bacterium DRI-13 | 0.00042 |
| Firmicutes | Clostridiales | Clostridiales noname | Clostridiales bacterium NK3B98 | 0.00209 |
| Firmicutes | Clostridiales | Clostridiales noname | Clostridiales bacterium oral taxon 876 | 0.00456 |
| Firmicutes | Clostridiales | Clostridiales noname | Clostridiales bacterium VE202-01 | 0.01440 |
| Firmicutes | Clostridiales | Clostridiales noname | Clostridiales bacterium VE202-03 | 0.01543 |
| Firmicutes | Clostridiales | Clostridiales noname | Clostridiales bacterium VE202-06 | 0.01308 |
| Firmicutes | Clostridiales | Clostridiales noname | Clostridiales bacterium VE202-07 | 0.00044 |
| Firmicutes | Clostridiales | Clostridiales noname | Clostridiales bacterium VE202-08 | 0.02144 |
| Firmicutes | Clostridiales | Clostridiales noname | Clostridiales bacterium VE202-09 | 0.01069 |
| Firmicutes | Clostridiales | Clostridiales noname | Clostridiales bacterium VE202-13 | 0.00190 |
| Firmicutes | Clostridiales | Clostridiales noname | Clostridiales bacterium VE202-14 | 0.02980 |
| Firmicutes | Clostridiales | Clostridiales noname | Clostridiales bacterium VE202-15 | 0.00210 |
| Firmicutes | Clostridiales | Clostridiales noname | Clostridiales bacterium VE202-16 | 0.00861 |
| Firmicutes | Clostridiales | Clostridiales noname | Clostridiales bacterium VE202-18 | 0.00134 |
| Firmicutes | Clostridiales | Clostridiales noname | Clostridiales bacterium VE202-21 | 0.02860 |
| Firmicutes | Clostridiales | Clostridiales noname | Clostridiales bacterium VE202-26 | 0.00222 |
| Firmicutes | Clostridiales | Clostridiales noname | Clostridiales bacterium VE202-27 | 0.01322 |
| Firmicutes | Clostridiales | Clostridiales noname | Clostridiales bacterium VE202-28 | 0.00488 |
| Firmicutes | Clostridiales | Clostridiales unclassified | Clostridiales unclassified | 1.00663 |
| Firmicutes | Clostridiales | Clostridiisalibacter | Clostridiisalibacter paucivorans | 0.00109 |
| Firmicutes | Clostridiales | Clostridium | Candidatus Clostridium anorexicamassiliense | 0.01789 |
| Firmicutes | Clostridiales | Clostridium | Clostridium aceticum | 0.00348 |
| Firmicutes | Clostridiales | Clostridium | Clostridium acetobutylicum | 0.00157 |
| Firmicutes | Clostridiales | Clostridium | Clostridium akagii | 0.00036 |
| Firmicutes | Clostridiales | Clostridium | Clostridium algidicarnis | 0.00053 |
| Firmicutes | Clostridiales | Clostridium | Clostridium arbusti | 0.00343 |
| Firmicutes | Clostridiales | Clostridium | Clostridium argentinense | 0.02523 |
| Firmicutes | Clostridiales | Clostridium | Clostridium autoethanogenum | 0.00038 |
| Firmicutes | Clostridiales | Clostridium | Clostridium baratii | 0.07389 |
| Firmicutes | Clostridiales | Clostridium | Clostridium bartlettii CAG:1329 | 0.00909 |
| Firmicutes | Clostridiales | Clostridium | Clostridium beijerinckii | 0.01107 |
| Firmicutes | Clostridiales | Clostridium | Clostridium bolteae CAG:59 | 0.00202 |
| Firmicutes | Clostridiales | Clostridium | Clostridium botulinum | 0.05600 |
| Firmicutes | Clostridiales | Clostridium | Clostridium butyricum | 0.01912 |
| Firmicutes | Clostridiales | Clostridium | Clostridium cadaveris | 0.00912 |
| Firmicutes | Clostridiales | Clostridium | Clostridium carboxidivorans | 0.00080 |
| Firmicutes | Clostridiales | Clostridium | Clostridium celatum | 0.00505 |
| Firmicutes | Clostridiales | Clostridium | Clostridium cellulovorans | 0.00289 |
| Firmicutes | Clostridiales | Clostridium | Clostridium chauvoei | 0.00694 |
| Firmicutes | Clostridiales | Clostridium | Clostridium clostridioforme CAG:132 | 0.00332 |
| Firmicutes | Clostridiales | Clostridium | Clostridium clostridioforme CAG:511 | 0.00041 |
| Firmicutes | Clostridiales | Clostridium | Clostridium colicanis | 0.03400 |
| Firmicutes | Clostridiales | Clostridium | Clostridium diolis | 0.00123 |
| Firmicutes | Clostridiales | Clostridium | Clostridium drakei | 0.00073 |
| Firmicutes | Clostridiales | Clostridium | Clostridium haemolyticum | 0.00090 |
| Firmicutes | Clostridiales | Clostridium | Clostridium hathewayi CAG:224 | 0.00779 |
| Firmicutes | Clostridiales | Clostridium | Clostridium hydrogeniformans | 0.00351 |
| Firmicutes | Clostridiales | Clostridium | Clostridium intestinale | 0.01357 |
| Firmicutes | Clostridiales | Clostridium | Clostridium kluyveri | 0.00857 |
| Firmicutes | Clostridiales | Clostridium | Clostridium leptum CAG:27 | 0.00841 |
| Firmicutes | Clostridiales | Clostridium | Clostridium ljungdahlii | 0.00147 |
| Firmicutes | Clostridiales | Clostridium | Clostridium lundense | 0.00118 |
| Firmicutes | Clostridiales | Clostridium | Clostridium nexile CAG:348 | 0.04713 |
| Firmicutes | Clostridiales | Clostridium | Clostridium novyi | 0.01833 |
| Firmicutes | Clostridiales | Clostridium | Clostridium paraputrificum | 0.01953 |
| Firmicutes | Clostridiales | Clostridium | Clostridium pasteurianum | 0.00598 |
| Firmicutes | Clostridiales | Clostridium | Clostridium perfringens | 1.45863 |
| Firmicutes | Clostridiales | Clostridium | Clostridium saccharobutylicum | 0.00300 |
| Firmicutes | Clostridiales | Clostridium | Clostridium saccharoperbutylacetonicum | 0.00250 |
| Firmicutes | Clostridiales | Clostridium | Clostridium sartagoforme | 0.00385 |
| Firmicutes | Clostridiales | Clostridium | Clostridium scatologenes | 0.00129 |
| Firmicutes | Clostridiales | Clostridium | Clostridium senegalense | 0.01300 |
| Firmicutes | Clostridiales | Clostridium | Clostridium septicum | 0.00044 |
| Firmicutes | Clostridiales | Clostridium | Clostridium sp. 01 | 0.60482 |
| Firmicutes | Clostridiales | Clostridium | Clostridium sp. 12(A) | 0.01092 |
| Firmicutes | Clostridiales | Clostridium | Clostridium sp. 7 2 43FAA | 0.00887 |
| Firmicutes | Clostridiales | Clostridium | Clostridium sp. 7 3 54FAA | 0.00097 |
| Firmicutes | Clostridiales | Clostridium | Clostridium sp. Ade.TY | 0.10944 |
| Firmicutes | Clostridiales | Clostridium | Clostridium sp. ASBs410 | 0.00340 |
| Firmicutes | Clostridiales | Clostridium | Clostridium sp. ASF356 | 0.00292 |
| Firmicutes | Clostridiales | Clostridium | Clostridium sp. ASF502 | 0.02981 |
| Firmicutes | Clostridiales | Clostridium | Clostridium sp. ATCC 29733 | 0.00274 |
| Firmicutes | Clostridiales | Clostridium | Clostridium sp. ATCC BAA-442 | 0.01294 |
| Firmicutes | Clostridiales | Clostridium | Clostridium sp. BL8 | 0.00602 |
| Firmicutes | Clostridiales | Clostridium | Clostridium sp. BNL1100 | 0.00030 |
| Firmicutes | Clostridiales | Clostridium | Clostridium sp. CAG:1000 | 0.00001 |
| Firmicutes | Clostridiales | Clostridium | Clostridium sp. CAG:1013 | 0.01569 |
| Firmicutes | Clostridiales | Clostridium | Clostridium sp. CAG:1024 | 0.00324 |
| Firmicutes | Clostridiales | Clostridium | Clostridium sp. CAG:1193 | 0.00012 |
| Firmicutes | Clostridiales | Clostridium | Clostridium sp. CAG:1219 | 0.00002 |
| Firmicutes | Clostridiales | Clostridium | Clostridium sp. CAG:122 | 0.00159 |
| Firmicutes | Clostridiales | Clostridium | Clostridium sp. CAG:127 | 0.00208 |
| Firmicutes | Clostridiales | Clostridium | Clostridium sp. CAG:138 | 0.00112 |
| Firmicutes | Clostridiales | Clostridium | Clostridium sp. CAG:149 | 0.00170 |
| Firmicutes | Clostridiales | Clostridium | Clostridium sp. CAG:167 | 0.00702 |
| Firmicutes | Clostridiales | Clostridium | Clostridium sp. CAG:169 | 0.15908 |
| Firmicutes | Clostridiales | Clostridium | Clostridium sp. CAG:217 | 0.00110 |
| Firmicutes | Clostridiales | Clostridium | Clostridium sp. CAG:221 | 0.00793 |
| Firmicutes | Clostridiales | Clostridium | Clostridium sp. CAG:226 | 0.00174 |
| Firmicutes | Clostridiales | Clostridium | Clostridium sp. CAG:230 | 0.00073 |
| Firmicutes | Clostridiales | Clostridium | Clostridium sp. CAG:242 | 0.54106 |
| Firmicutes | Clostridiales | Clostridium | Clostridium sp. CAG:245 | 0.00008 |
| Firmicutes | Clostridiales | Clostridium | Clostridium sp. CAG:253 | 0.01285 |
| Firmicutes | Clostridiales | Clostridium | Clostridium sp. CAG:264 | 0.00876 |
| Firmicutes | Clostridiales | Clostridium | Clostridium sp. CAG:265 | 0.00306 |
| Firmicutes | Clostridiales | Clostridium | Clostridium sp. CAG:273 | 0.00008 |
| Firmicutes | Clostridiales | Clostridium | Clostridium sp. CAG:277 | 0.00259 |
| Firmicutes | Clostridiales | Clostridium | Clostridium sp. CAG:288 | 0.00031 |
| Firmicutes | Clostridiales | Clostridium | Clostridium sp. CAG:299 | 0.00970 |
| Firmicutes | Clostridiales | Clostridium | Clostridium sp. CAG:302 | 0.00005 |
| Firmicutes | Clostridiales | Clostridium | Clostridium sp. CAG:306 | 0.00030 |
| Firmicutes | Clostridiales | Clostridium | Clostridium sp. CAG:307 | 0.00002 |
| Firmicutes | Clostridiales | Clostridium | Clostridium sp. CAG:343 | 0.00002 |
| Firmicutes | Clostridiales | Clostridium | Clostridium sp. CAG:349 | 0.00043 |
| Firmicutes | Clostridiales | Clostridium | Clostridium sp. CAG:352 | 0.00313 |
| Firmicutes | Clostridiales | Clostridium | Clostridium sp. CAG:354 | 0.00001 |
| Firmicutes | Clostridiales | Clostridium | Clostridium sp. CAG:411 | 0.00071 |
| Firmicutes | Clostridiales | Clostridium | Clostridium sp. CAG:413 | 0.00062 |
| Firmicutes | Clostridiales | Clostridium | Clostridium sp. CAG:417 | 0.00175 |
| Firmicutes | Clostridiales | Clostridium | Clostridium sp. CAG:43 | 0.01027 |
| Firmicutes | Clostridiales | Clostridium | Clostridium sp. CAG:433 | 0.00004 |
| Firmicutes | Clostridiales | Clostridium | Clostridium sp. CAG:440 | 0.00192 |
| Firmicutes | Clostridiales | Clostridium | Clostridium sp. CAG:448 | 0.00046 |
| Firmicutes | Clostridiales | Clostridium | Clostridium sp. CAG:451 | 0.00001 |
| Firmicutes | Clostridiales | Clostridium | Clostridium sp. CAG:452 | 0.00002 |
| Firmicutes | Clostridiales | Clostridium | Clostridium sp. CAG:465 | 0.00028 |
| Firmicutes | Clostridiales | Clostridium | Clostridium sp. CAG:470 | 0.00608 |
| Firmicutes | Clostridiales | Clostridium | Clostridium sp. CAG:492 | 0.00001 |
| Firmicutes | Clostridiales | Clostridium | Clostridium sp. CAG:505 | 0.00893 |
| Firmicutes | Clostridiales | Clostridium | Clostridium sp. CAG:508 | 0.00004 |
| Firmicutes | Clostridiales | Clostridium | Clostridium sp. CAG:510 | 0.00144 |
| Firmicutes | Clostridiales | Clostridium | Clostridium sp. CAG:524 | 0.00045 |
| Firmicutes | Clostridiales | Clostridium | Clostridium sp. CAG:557 | 0.00018 |
| Firmicutes | Clostridiales | Clostridium | Clostridium sp. CAG:567 | 0.00004 |
| Firmicutes | Clostridiales | Clostridium | Clostridium sp. CAG:571 | 0.00421 |
| Firmicutes | Clostridiales | Clostridium | Clostridium sp. CAG:575 | 0.00024 |
| Firmicutes | Clostridiales | Clostridium | Clostridium sp. CAG:58 | 0.02404 |
| Firmicutes | Clostridiales | Clostridium | Clostridium sp. CAG:590 | 0.00078 |
| Firmicutes | Clostridiales | Clostridium | Clostridium sp. CAG:609 | 0.00004 |
| Firmicutes | Clostridiales | Clostridium | Clostridium sp. CAG:62 | 0.00075 |
| Firmicutes | Clostridiales | Clostridium | Clostridium sp. CAG:628 | 0.00005 |
| Firmicutes | Clostridiales | Clostridium | Clostridium sp. CAG:632 | 0.00076 |
| Firmicutes | Clostridiales | Clostridium | Clostridium sp. CAG:678 | 0.00324 |
| Firmicutes | Clostridiales | Clostridium | Clostridium sp. CAG:7 | 0.05121 |
| Firmicutes | Clostridiales | Clostridium | Clostridium sp. CAG:710 | 0.00007 |
| Firmicutes | Clostridiales | Clostridium | Clostridium sp. CAG:715 | 0.00005 |
| Firmicutes | Clostridiales | Clostridium | Clostridium sp. CAG:729 | 0.00031 |
| Firmicutes | Clostridiales | Clostridium | Clostridium sp. CAG:75 | 0.00047 |
| Firmicutes | Clostridiales | Clostridium | Clostridium sp. CAG:762 | 0.00001 |
| Firmicutes | Clostridiales | Clostridium | Clostridium sp. CAG:768 | 0.00019 |
| Firmicutes | Clostridiales | Clostridium | Clostridium sp. CAG:780 | 0.00050 |
| Firmicutes | Clostridiales | Clostridium | Clostridium sp. CAG:793 | 0.00005 |
| Firmicutes | Clostridiales | Clostridium | Clostridium sp. CAG:798 | 0.00001 |
| Firmicutes | Clostridiales | Clostridium | Clostridium sp. CAG:81 | 0.00354 |
| Firmicutes | Clostridiales | Clostridium | Clostridium sp. CAG:91 | 0.00085 |
| Firmicutes | Clostridiales | Clostridium | Clostridium sp. CAG:914 | 0.00004 |
| Firmicutes | Clostridiales | Clostridium | Clostridium sp. CAG:921 | 0.00002 |
| Firmicutes | Clostridiales | Clostridium | Clostridium sp. CAG:964 | 0.00061 |
| Firmicutes | Clostridiales | Clostridium | Clostridium sp. CL-2 | 0.02162 |
| Firmicutes | Clostridiales | Clostridium | Clostridium sp. CL-6 | 0.01717 |
| Firmicutes | Clostridiales | Clostridium | Clostridium sp. D5 | 0.03502 |
| Firmicutes | Clostridiales | Clostridium | Clostridium sp. DL-VIII | 0.00444 |
| Firmicutes | Clostridiales | Clostridium | Clostridium sp. FS41 | 0.00319 |
| Firmicutes | Clostridiales | Clostridium | Clostridium sp. HGF2 | 0.00979 |
| Firmicutes | Clostridiales | Clostridium | Clostridium sp. HMP27 | 0.00109 |
| Firmicutes | Clostridiales | Clostridium | Clostridium sp. IBUN125C | 0.00058 |
| Firmicutes | Clostridiales | Clostridium | Clostridium sp. IBUN13A | 0.00002 |
| Firmicutes | Clostridiales | Clostridium | Clostridium sp. IBUN22A | 0.00008 |
| Firmicutes | Clostridiales | Clostridium | Clostridium sp. IBUN62F | 0.00022 |
| Firmicutes | Clostridiales | Clostridium | Clostridium sp. JCC | 0.00547 |
| Firmicutes | Clostridiales | Clostridium | Clostridium sp. JCD | 0.00060 |
| Firmicutes | Clostridiales | Clostridium | Clostridium sp. K25 | 0.00073 |
| Firmicutes | Clostridiales | Clostridium | Clostridium sp. KLE 1755 | 0.01979 |
| Firmicutes | Clostridiales | Clostridium | Clostridium sp. KNHs205 | 0.00097 |
| Firmicutes | Clostridiales | Clostridium | Clostridium sp. KNHs209 | 0.00554 |
| Firmicutes | Clostridiales | Clostridium | Clostridium sp. KNHs214 | 0.00204 |
| Firmicutes | Clostridiales | Clostridium | Clostridium sp. L2-50 | 0.00596 |
| Firmicutes | Clostridiales | Clostridium | Clostridium sp. LF2 | 0.00704 |
| Firmicutes | Clostridiales | Clostridium | Clostridium sp. M2/40 | 0.00585 |
| Firmicutes | Clostridiales | Clostridium | Clostridium sp. M62/1 | 0.01392 |
| Firmicutes | Clostridiales | Clostridium | Clostridium sp. Maddingley MBC34-26 | 0.00492 |
| Firmicutes | Clostridiales | Clostridium | Clostridium sp. MS1 | 0.01285 |
| Firmicutes | Clostridiales | Clostridium | Clostridium sp. MSTE9 | 0.00142 |
| Firmicutes | Clostridiales | Clostridium | Clostridium sp. NCR | 0.07181 |
| Firmicutes | Clostridiales | Clostridium | Clostridium sp. SS2/1 | 0.02705 |
| Firmicutes | Clostridiales | Clostridium | Clostridium sp. SY8519 | 0.01572 |
| Firmicutes | Clostridiales | Clostridium | Clostridium sporogenes | 0.00298 |
| Firmicutes | Clostridiales | Clostridium | Clostridium sulfidigenes | 0.00545 |
| Firmicutes | Clostridiales | Clostridium | Clostridium tetani | 0.01304 |
| Firmicutes | Clostridiales | Clostridium | Clostridium tetanomorphum | 0.00137 |
| Firmicutes | Clostridiales | Clostridium | Clostridium tunisiense | 0.00292 |
| Firmicutes | Clostridiales | Clostridium | Clostridium tyrobutyricum | 0.00701 |
| Firmicutes | Clostridiales | Clostridium | Clostridium ultunense | 0.00049 |
| Firmicutes | Clostridiales | Clostridium | Clostridium unclassified | 0.02143 |
| Firmicutes | Clostridiales | Coprococcus | Coprococcus catus | 0.01780 |
| Firmicutes | Clostridiales | Coprococcus | Coprococcus comes | 0.03024 |
| Firmicutes | Clostridiales | Coprococcus | Coprococcus comes CAG:19 | 0.01661 |
| Firmicutes | Clostridiales | Coprococcus | Coprococcus eutactus | 0.00039 |
| Firmicutes | Clostridiales | Coprococcus | Coprococcus eutactus CAG:665 | 0.00772 |
| Firmicutes | Clostridiales | Coprococcus | Coprococcus sp. ART55/1 | 0.00096 |
| Firmicutes | Clostridiales | Coprococcus | Coprococcus sp. CAG:131 | 0.00072 |
| Firmicutes | Clostridiales | Coprococcus | Coprococcus sp. CAG:782 | 0.00336 |
| Firmicutes | Clostridiales | Coprococcus | Coprococcus sp. HPP0048 | 0.19010 |
| Firmicutes | Clostridiales | Coprococcus | Coprococcus sp. HPP0074 | 0.26751 |
| Firmicutes | Clostridiales | Coprococcus | Coprococcus unclassified | 0.12660 |
| Firmicutes | Clostridiales | Dehalobacter | Dehalobacter restrictus | 0.00209 |
| Firmicutes | Clostridiales | Dehalobacter | Dehalobacter sp. CF | 0.00014 |
| Firmicutes | Clostridiales | Dehalobacter | Dehalobacter sp. FTH1 | 0.00079 |
| Firmicutes | Clostridiales | Dehalobacter | Dehalobacter unclassified | 0.00267 |
| Firmicutes | Clostridiales | Desulfitibacter | Desulfitibacter alkalitolerans | 0.00428 |
| Firmicutes | Clostridiales | Desulfitobacterium | Desulfitobacterium dehalogenans | 0.00029 |
| Firmicutes | Clostridiales | Desulfitobacterium | Desulfitobacterium dichloroeliminans | 0.00094 |
| Firmicutes | Clostridiales | Desulfitobacterium | Desulfitobacterium hafniense | 0.00359 |
| Firmicutes | Clostridiales | Desulfitobacterium | Desulfitobacterium metallireducens | 0.00019 |
| Firmicutes | Clostridiales | Desulfitobacterium | Desulfitobacterium sp. PCE1 | 0.00019 |
| Firmicutes | Clostridiales | Desulfitobacterium | Desulfitobacterium unclassified | 0.00010 |
| Firmicutes | Clostridiales | Desulfosporosinus | Desulfosporosinus orientis | 0.00005 |
| Firmicutes | Clostridiales | Desulfosporosinus | Desulfosporosinus sp. BICA1-9 | 0.00055 |
| Firmicutes | Clostridiales | Desulfosporosinus | Desulfosporosinus sp. HMP52 | 0.00016 |
| Firmicutes | Clostridiales | Desulfosporosinus | Desulfosporosinus sp. I2 | 0.00184 |
| Firmicutes | Clostridiales | Desulfosporosinus | Desulfosporosinus sp. Tol-M | 0.00026 |
| Firmicutes | Clostridiales | Desulfosporosinus | Desulfosporosinus youngiae | 0.00096 |
| Firmicutes | Clostridiales | Desulfotomaculum | Desulfotomaculum acetoxidans | 0.00015 |
| Firmicutes | Clostridiales | Desulfotomaculum | Desulfotomaculum alcoholivorax | 0.00061 |
| Firmicutes | Clostridiales | Desulfotomaculum | Desulfotomaculum alkaliphilum | 0.00038 |
| Firmicutes | Clostridiales | Desulfotomaculum | Desulfotomaculum gibsoniae | 0.00078 |
| Firmicutes | Clostridiales | Desulfotomaculum | Desulfotomaculum nigrificans | 0.00009 |
| Firmicutes | Clostridiales | Desulfotomaculum | Desulfotomaculum reducens | 0.00027 |
| Firmicutes | Clostridiales | Desulfotomaculum | Desulfotomaculum ruminis | 0.00059 |
| Firmicutes | Clostridiales | Desulfotomaculum | Desulfotomaculum sp. BICA1-6 | 0.00021 |
| Firmicutes | Clostridiales | Desulfurispora | Desulfurispora thermophila | 0.00002 |
| Firmicutes | Clostridiales | Dethiobacter | Dethiobacter alkaliphilus | 0.00016 |
| Firmicutes | Clostridiales | Dorea | Dorea formicigenerans | 0.03529 |
| Firmicutes | Clostridiales | Dorea | Dorea formicigenerans CAG:28 | 0.00860 |
| Firmicutes | Clostridiales | Dorea | Dorea longicatena | 0.06287 |
| Firmicutes | Clostridiales | Dorea | Dorea longicatena CAG:42 | 0.00191 |
| Firmicutes | Clostridiales | Dorea | Dorea sp. 5-2 | 0.07405 |
| Firmicutes | Clostridiales | Dorea | Dorea sp. AGR2135 | 0.03981 |
| Firmicutes | Clostridiales | Dorea | Dorea sp. CAG:105 | 0.01455 |
| Firmicutes | Clostridiales | Dorea | Dorea sp. CAG:317 | 0.01917 |
| Firmicutes | Clostridiales | Dorea | Dorea unclassified | 0.00654 |
| Firmicutes | Clostridiales | Epulopiscium | Epulopiscium sp. 'N.t. morphotype B' | 0.00477 |
| Firmicutes | Clostridiales | Ethanoligenens | Ethanoligenens harbinense | 0.00071 |
| Firmicutes | Clostridiales | Eubacterium | [Eubacterium] cellulosolvens | 0.04620 |
| Firmicutes | Clostridiales | Eubacterium | [Eubacterium] eligens | 0.00150 |
| Firmicutes | Clostridiales | Eubacterium | [Eubacterium] hallii | 0.00698 |
| Firmicutes | Clostridiales | Eubacterium | Eubacterium acidaminophilum | 0.00176 |
| Firmicutes | Clostridiales | Eubacterium | Eubacterium brachy | 0.02296 |
| Firmicutes | Clostridiales | Eubacterium | Eubacterium desmolans | 0.02706 |
| Firmicutes | Clostridiales | Eubacterium | Eubacterium dolichum CAG:375 | 0.07212 |
| Firmicutes | Clostridiales | Eubacterium | Eubacterium eligens CAG:72 | 0.00002 |
| Firmicutes | Clostridiales | Eubacterium | Eubacterium hallii CAG:12 | 0.00166 |
| Firmicutes | Clostridiales | Eubacterium | Eubacterium limosum | 0.02141 |
| Firmicutes | Clostridiales | Eubacterium | Eubacterium plexicaudatum | 0.01813 |
| Firmicutes | Clostridiales | Eubacterium | Eubacterium ramulus | 0.03227 |
| Firmicutes | Clostridiales | Eubacterium | Eubacterium rectale | 0.02678 |
| Firmicutes | Clostridiales | Eubacterium | Eubacterium rectale CAG:36 | 0.00578 |
| Firmicutes | Clostridiales | Eubacterium | Eubacterium saphenum | 0.00005 |
| Firmicutes | Clostridiales | Eubacterium | Eubacterium siraeum CAG:80 | 0.00051 |
| Firmicutes | Clostridiales | Eubacterium | Eubacterium sp. 14-2 | 0.00303 |
| Firmicutes | Clostridiales | Eubacterium | Eubacterium sp. 3 1 31 | 0.06437 |
| Firmicutes | Clostridiales | Eubacterium | Eubacterium sp. AB3007 | 0.00075 |
| Firmicutes | Clostridiales | Eubacterium | Eubacterium sp. CAG:115 | 0.00570 |
| Firmicutes | Clostridiales | Eubacterium | Eubacterium sp. CAG:146 | 0.00589 |
| Firmicutes | Clostridiales | Eubacterium | Eubacterium sp. CAG:156 | 0.00724 |
| Firmicutes | Clostridiales | Eubacterium | Eubacterium sp. CAG:161 | 0.01316 |
| Firmicutes | Clostridiales | Eubacterium | Eubacterium sp. CAG:180 | 0.00031 |
| Firmicutes | Clostridiales | Eubacterium | Eubacterium sp. CAG:192 | 0.00119 |
| Firmicutes | Clostridiales | Eubacterium | Eubacterium sp. CAG:202 | 0.00068 |
| Firmicutes | Clostridiales | Eubacterium | Eubacterium sp. CAG:248 | 0.01343 |
| Firmicutes | Clostridiales | Eubacterium | Eubacterium sp. CAG:251 | 0.00573 |
| Firmicutes | Clostridiales | Eubacterium | Eubacterium sp. CAG:252 | 0.00066 |
| Firmicutes | Clostridiales | Eubacterium | Eubacterium sp. CAG:274 | 0.00047 |
| Firmicutes | Clostridiales | Eubacterium | Eubacterium sp. CAG:38 | 0.00107 |
| Firmicutes | Clostridiales | Eubacterium | Eubacterium sp. CAG:581 | 0.00020 |
| Firmicutes | Clostridiales | Eubacterium | Eubacterium sp. CAG:603 | 0.00091 |
| Firmicutes | Clostridiales | Eubacterium | Eubacterium sp. CAG:76 | 0.00078 |
| Firmicutes | Clostridiales | Eubacterium | Eubacterium sp. CAG:786 | 0.00045 |
| Firmicutes | Clostridiales | Eubacterium | Eubacterium sp. CAG:841 | 0.00799 |
| Firmicutes | Clostridiales | Eubacterium | Eubacterium sp. CAG:86 | 0.00121 |
| Firmicutes | Clostridiales | Eubacterium | Eubacterium sp. ER2 | 0.03962 |
| Firmicutes | Clostridiales | Eubacterium | Eubacterium unclassified | 0.00104 |
| Firmicutes | Clostridiales | Eubacterium | Eubacterium ventriosum | 0.00965 |
| Firmicutes | Clostridiales | Eubacterium | Eubacterium xylanophilum | 0.00611 |
| Firmicutes | Clostridiales | Faecalibacterium | Faecalibacterium prausnitzii | 0.06989 |
| Firmicutes | Clostridiales | Faecalibacterium | Faecalibacterium sp. CAG:1138 | 0.00523 |
| Firmicutes | Clostridiales | Faecalibacterium | Faecalibacterium sp. CAG:74 | 0.00036 |
| Firmicutes | Clostridiales | Faecalibacterium | Faecalibacterium sp. CAG:82 | 0.01060 |
| Firmicutes | Clostridiales | Fervidicella | Fervidicella metallireducens | 0.00096 |
| Firmicutes | Clostridiales | Filifactor | Filifactor alocis | 0.00194 |
| Firmicutes | Clostridiales | Finegoldia | Finegoldia magna | 0.02304 |
| Firmicutes | Clostridiales | Flavonifractor | Flavonifractor plautii | 0.02720 |
| Firmicutes | Clostridiales | Gottschalkia | Gottschalkia acidurici | 0.00776 |
| Firmicutes | Clostridiales | Helcococcus | Helcococcus kunzii | 0.00747 |
| Firmicutes | Clostridiales | Helcococcus | Helcococcus sueciensis | 0.00086 |
| Firmicutes | Clostridiales | Heliobacterium | Heliobacterium modesticaldum | 0.00023 |
| Firmicutes | Clostridiales | Hungatella | Hungatella hathewayi | 0.03830 |
| Firmicutes | Clostridiales | Intestinibacter | Intestinibacter bartlettii | 0.02519 |
| Firmicutes | Clostridiales | Intestinimonas | Intestinimonas butyriciproducens | 0.00118 |
| Firmicutes | Clostridiales | Johnsonella | Johnsonella ignava | 0.00091 |
| Firmicutes | Clostridiales | Kallipyga | Kallipyga massiliensis | 0.00015 |
| Firmicutes | Clostridiales | Lachnoanaerobaculum | Lachnoanaerobaculum saburreum | 0.00116 |
| Firmicutes | Clostridiales | Lachnoanaerobaculum | Lachnoanaerobaculum sp. ICM7 | 0.00126 |
| Firmicutes | Clostridiales | Lachnoanaerobaculum | Lachnoanaerobaculum sp. MSX33 | 0.00605 |
| Firmicutes | Clostridiales | Lachnoanaerobaculum | Lachnoanaerobaculum sp. OBRC5-5 | 0.00220 |
| Firmicutes | Clostridiales | Lachnoanaerobaculum | Lachnoanaerobaculum unclassified | 0.00050 |
| Firmicutes | Clostridiales | Lachnobacterium | Lachnobacterium bovis | 0.00190 |
| Firmicutes | Clostridiales | Lachnoclostridium | [Clostridium] aerotolerans | 0.00113 |
| Firmicutes | Clostridiales | Lachnoclostridium | [Clostridium] aminophilum | 0.00115 |
| Firmicutes | Clostridiales | Lachnoclostridium | [Clostridium] asparagiforme | 0.00581 |
| Firmicutes | Clostridiales | Lachnoclostridium | [Clostridium] bolteae | 0.06260 |
| Firmicutes | Clostridiales | Lachnoclostridium | [Clostridium] celerecrescens | 0.01833 |
| Firmicutes | Clostridiales | Lachnoclostridium | [Clostridium] citroniae | 0.01322 |
| Firmicutes | Clostridiales | Lachnoclostridium | [Clostridium] clostridioforme | 0.11244 |
| Firmicutes | Clostridiales | Lachnoclostridium | [Clostridium] hylemonae | 0.00849 |
| Firmicutes | Clostridiales | Lachnoclostridium | [Clostridium] methoxybenzovorans | 0.00832 |
| Firmicutes | Clostridiales | Lachnoclostridium | [Clostridium] saccharolyticum | 0.03495 |
| Firmicutes | Clostridiales | Lachnoclostridium | [Clostridium] scindens | 0.08776 |
| Firmicutes | Clostridiales | Lachnoclostridium | [Clostridium] symbiosum | 0.04526 |
| Firmicutes | Clostridiales | Lachnoclostridium | Lachnoclostridium phytofermentans | 0.00962 |
| Firmicutes | Clostridiales | Lachnoclostridium | Lachnoclostridium unclassified | 0.01072 |
| Firmicutes | Clostridiales | Lachnospira | Lachnospira multipara | 0.00140 |
| Firmicutes | Clostridiales | Lachnospiraceae noname | Lachnospiraceae bacterium 1 1 57FAA | 0.00077 |
| Firmicutes | Clostridiales | Lachnospiraceae noname | Lachnospiraceae bacterium 1 4 56FAA | 0.07009 |
| Firmicutes | Clostridiales | Lachnospiraceae noname | Lachnospiraceae bacterium 10-1 | 0.09198 |
| Firmicutes | Clostridiales | Lachnospiraceae noname | Lachnospiraceae bacterium 2 1 46FAA | 0.43276 |
| Firmicutes | Clostridiales | Lachnospiraceae noname | Lachnospiraceae bacterium 2 1 58FAA | 0.09749 |
| Firmicutes | Clostridiales | Lachnospiraceae noname | Lachnospiraceae bacterium 28-4 | 0.00667 |
| Firmicutes | Clostridiales | Lachnospiraceae noname | Lachnospiraceae bacterium 3 1 46FAA | 0.00396 |
| Firmicutes | Clostridiales | Lachnospiraceae noname | Lachnospiraceae bacterium 3 1 57FAA CT1 | 0.00528 |
| Firmicutes | Clostridiales | Lachnospiraceae noname | Lachnospiraceae bacterium 3-1 | 0.00702 |
| Firmicutes | Clostridiales | Lachnospiraceae noname | Lachnospiraceae bacterium 3-2 | 0.02691 |
| Firmicutes | Clostridiales | Lachnospiraceae noname | Lachnospiraceae bacterium 5 1 57FAA | 0.01063 |
| Firmicutes | Clostridiales | Lachnospiraceae noname | Lachnospiraceae bacterium 5 1 63FAA | 0.00223 |
| Firmicutes | Clostridiales | Lachnospiraceae noname | Lachnospiraceae bacterium 6 1 37FAA | 0.18640 |
| Firmicutes | Clostridiales | Lachnospiraceae noname | Lachnospiraceae bacterium 6 1 63FAA | 0.11250 |
| Firmicutes | Clostridiales | Lachnospiraceae noname | Lachnospiraceae bacterium 7 1 58FAA | 0.00434 |
| Firmicutes | Clostridiales | Lachnospiraceae noname | Lachnospiraceae bacterium 8 1 57FAA | 0.00600 |
| Firmicutes | Clostridiales | Lachnospiraceae noname | Lachnospiraceae bacterium 9 1 43BFAA | 0.08307 |
| Firmicutes | Clostridiales | Lachnospiraceae noname | Lachnospiraceae bacterium A2 | 0.01926 |
| Firmicutes | Clostridiales | Lachnospiraceae noname | Lachnospiraceae bacterium A4 | 0.01792 |
| Firmicutes | Clostridiales | Lachnospiraceae noname | Lachnospiraceae bacterium AB2028 | 0.00130 |
| Firmicutes | Clostridiales | Lachnospiraceae noname | Lachnospiraceae bacterium AC2012 | 0.00024 |
| Firmicutes | Clostridiales | Lachnospiraceae noname | Lachnospiraceae bacterium AC2014 | 0.00162 |
| Firmicutes | Clostridiales | Lachnospiraceae noname | Lachnospiraceae bacterium AC2028 | 0.00872 |
| Firmicutes | Clostridiales | Lachnospiraceae noname | Lachnospiraceae bacterium AC2029 | 0.00705 |
| Firmicutes | Clostridiales | Lachnospiraceae noname | Lachnospiraceae bacterium AC2031 | 0.00025 |
| Firmicutes | Clostridiales | Lachnospiraceae noname | Lachnospiraceae bacterium AC3007 | 0.00002 |
| Firmicutes | Clostridiales | Lachnospiraceae noname | Lachnospiraceae bacterium AD3010 | 0.00018 |
| Firmicutes | Clostridiales | Lachnospiraceae noname | Lachnospiraceae bacterium C6A11 | 0.00005 |
| Firmicutes | Clostridiales | Lachnospiraceae noname | Lachnospiraceae bacterium CAG:215 | 0.02839 |
| Firmicutes | Clostridiales | Lachnospiraceae noname | Lachnospiraceae bacterium CAG:25 | 0.00042 |
| Firmicutes | Clostridiales | Lachnospiraceae noname | Lachnospiraceae bacterium CAG:364 | 0.09141 |
| Firmicutes | Clostridiales | Lachnospiraceae noname | Lachnospiraceae bacterium COE1 | 0.01409 |
| Firmicutes | Clostridiales | Lachnospiraceae noname | Lachnospiraceae bacterium FD2005 | 0.00535 |
| Firmicutes | Clostridiales | Lachnospiraceae noname | Lachnospiraceae bacterium FE2018 | 0.00573 |
| Firmicutes | Clostridiales | Lachnospiraceae noname | Lachnospiraceae bacterium JC7 | 0.03571 |
| Firmicutes | Clostridiales | Lachnospiraceae noname | Lachnospiraceae bacterium M18-1 | 0.01517 |
| Firmicutes | Clostridiales | Lachnospiraceae noname | Lachnospiraceae bacterium MA2020 | 0.02632 |
| Firmicutes | Clostridiales | Lachnospiraceae noname | Lachnospiraceae bacterium MC2017 | 0.00531 |
| Firmicutes | Clostridiales | Lachnospiraceae noname | Lachnospiraceae bacterium MD2004 | 0.00001 |
| Firmicutes | Clostridiales | Lachnospiraceae noname | Lachnospiraceae bacterium NC2004 | 0.00012 |
| Firmicutes | Clostridiales | Lachnospiraceae noname | Lachnospiraceae bacterium NC2008 | 0.00693 |
| Firmicutes | Clostridiales | Lachnospiraceae noname | Lachnospiraceae bacterium ND2006 | 0.02807 |
| Firmicutes | Clostridiales | Lachnospiraceae noname | Lachnospiraceae bacterium NK4A136 | 0.00014 |
| Firmicutes | Clostridiales | Lachnospiraceae noname | Lachnospiraceae bacterium NK4A144 | 0.00456 |
| Firmicutes | Clostridiales | Lachnospiraceae noname | Lachnospiraceae bacterium NK4A179 | 0.00206 |
| Firmicutes | Clostridiales | Lachnospiraceae noname | Lachnospiraceae bacterium oral taxon 082 | 0.01040 |
| Firmicutes | Clostridiales | Lachnospiraceae noname | Lachnospiraceae bacterium P6A3 | 0.00054 |
| Firmicutes | Clostridiales | Lachnospiraceae noname | Lachnospiraceae bacterium P6B14 | 0.00033 |
| Firmicutes | Clostridiales | Lachnospiraceae noname | Lachnospiraceae bacterium TWA4 | 0.01073 |
| Firmicutes | Clostridiales | Lachnospiraceae noname | Lachnospiraceae bacterium V9D3004 | 0.01519 |
| Firmicutes | Clostridiales | Lachnospiraceae noname | Lachnospiraceae bacterium VE202-12 | 0.00002 |
| Firmicutes | Clostridiales | Lachnospiraceae noname | Lachnospiraceae bacterium YSB2008 | 0.00263 |
| Firmicutes | Clostridiales | Lachnospiraceae noname | Lachnospiraceae oral taxon 107 | 0.01350 |
| Firmicutes | Clostridiales | Lachnospiraceae unclassified | Lachnospiraceae unclassified | 0.21535 |
| Firmicutes | Clostridiales | Mageeibacillus | Mageeibacillus indolicus | 0.00239 |
| Firmicutes | Clostridiales | Marvinbryantia | Marvinbryantia formatexigens | 0.04831 |
| Firmicutes | Clostridiales | Mogibacterium | Mogibacterium sp. CM50 | 0.00100 |
| Firmicutes | Clostridiales | Mogibacterium | Mogibacterium timidum | 0.00052 |
| Firmicutes | Clostridiales | Mogibacterium | Mogibacterium unclassified | 0.00003 |
| Firmicutes | Clostridiales | Oribacterium | Oribacterium asaccharolyticum | 0.00142 |
| Firmicutes | Clostridiales | Oribacterium | Oribacterium parvum | 0.00076 |
| Firmicutes | Clostridiales | Oribacterium | Oribacterium sinus | 0.00123 |
| Firmicutes | Clostridiales | Oribacterium | Oribacterium sp. FC2011 | 0.00227 |
| Firmicutes | Clostridiales | Oribacterium | Oribacterium sp. NK2B42 | 0.00329 |
| Firmicutes | Clostridiales | Oribacterium | Oribacterium sp. oral taxon 078 | 0.00190 |
| Firmicutes | Clostridiales | Oribacterium | Oribacterium sp. oral taxon 108 | 0.00149 |
| Firmicutes | Clostridiales | Oribacterium | Oribacterium sp. P6A1 | 0.01789 |
| Firmicutes | Clostridiales | Oribacterium | Oribacterium unclassified | 0.00012 |
| Firmicutes | Clostridiales | Oscillibacter | Oscillibacter ruminantium | 0.00516 |
| Firmicutes | Clostridiales | Oscillibacter | Oscillibacter sp. 1-3 | 0.00158 |
| Firmicutes | Clostridiales | Oscillibacter | Oscillibacter sp. CAG:155 | 0.00303 |
| Firmicutes | Clostridiales | Oscillibacter | Oscillibacter sp. CAG:241 | 0.00111 |
| Firmicutes | Clostridiales | Oscillibacter | Oscillibacter sp. ER4 | 0.04205 |
| Firmicutes | Clostridiales | Oscillibacter | Oscillibacter sp. KLE 1728 | 0.00164 |
| Firmicutes | Clostridiales | Oscillibacter | Oscillibacter sp. KLE 1745 | 0.00419 |
| Firmicutes | Clostridiales | Oscillibacter | Oscillibacter unclassified | 0.00205 |
| Firmicutes | Clostridiales | Oscillibacter | Oscillibacter valericigenes | 0.00606 |
| Firmicutes | Clostridiales | Oscillospiraceae noname | Oscillospiraceae bacterium VE202-24 | 0.00364 |
| Firmicutes | Clostridiales | Parvimonas | Parvimonas micra | 0.05561 |
| Firmicutes | Clostridiales | Parvimonas | Parvimonas sp. oral taxon 110 | 0.00067 |
| Firmicutes | Clostridiales | Parvimonas | Parvimonas sp. oral taxon 393 | 0.00031 |
| Firmicutes | Clostridiales | Parvimonas | Parvimonas unclassified | 0.00008 |
| Firmicutes | Clostridiales | Pelotomaculum | Pelotomaculum thermopropionicum | 0.00027 |
| Firmicutes | Clostridiales | Peptoclostridium | [Clostridium] bifermentans | 0.30597 |
| Firmicutes | Clostridiales | Peptoclostridium | [Clostridium] hiranonis | 2.08251 |
| Firmicutes | Clostridiales | Peptoclostridium | [Clostridium] litorale | 0.00136 |
| Firmicutes | Clostridiales | Peptoclostridium | [Clostridium] mangenotii | 0.02023 |
| Firmicutes | Clostridiales | Peptoclostridium | [Clostridium] sordellii | 1.13268 |
| Firmicutes | Clostridiales | Peptoclostridium | [Clostridium] sticklandii | 0.00120 |
| Firmicutes | Clostridiales | Peptoclostridium | [Eubacterium] yurii | 0.00038 |
| Firmicutes | Clostridiales | Peptoclostridium | Peptoclostridium difficile | 0.41546 |
| Firmicutes | Clostridiales | Peptoclostridium | Peptoclostridium unclassified | 0.00083 |
| Firmicutes | Clostridiales | Peptococcaceae noname | Peptococcaceae bacterium BICA1-7 | 0.00033 |
| Firmicutes | Clostridiales | Peptococcaceae noname | Peptococcaceae bacterium BICA1-8 | 0.00021 |
| Firmicutes | Clostridiales | Peptococcaceae noname | Peptococcaceae bacterium BRH c4a | 0.00003 |
| Firmicutes | Clostridiales | Peptococcaceae noname | Peptococcaceae bacterium BRH c8a | 0.00026 |
| Firmicutes | Clostridiales | Peptoniphilus | Peptoniphilus duerdenii | 0.00043 |
| Firmicutes | Clostridiales | Peptoniphilus | Peptoniphilus grossensis | 0.00035 |
| Firmicutes | Clostridiales | Peptoniphilus | Peptoniphilus harei | 0.00047 |
| Firmicutes | Clostridiales | Peptoniphilus | Peptoniphilus indolicus | 0.00118 |
| Firmicutes | Clostridiales | Peptoniphilus | Peptoniphilus lacrimalis | 0.00097 |
| Firmicutes | Clostridiales | Peptoniphilus | Peptoniphilus obesi | 0.00069 |
| Firmicutes | Clostridiales | Peptoniphilus | Peptoniphilus rhinitidis | 0.00022 |
| Firmicutes | Clostridiales | Peptoniphilus | Peptoniphilus senegalensis | 0.00030 |
| Firmicutes | Clostridiales | Peptoniphilus | Peptoniphilus sp. 1-1 | 0.00137 |
| Firmicutes | Clostridiales | Peptoniphilus | Peptoniphilus sp. BV3AC2 | 0.00046 |
| Firmicutes | Clostridiales | Peptoniphilus | Peptoniphilus sp. BV3C26 | 0.00124 |
| Firmicutes | Clostridiales | Peptoniphilus | Peptoniphilus sp. ChDC B134 | 0.00119 |
| Firmicutes | Clostridiales | Peptoniphilus | Peptoniphilus sp. oral taxon 375 | 0.00010 |
| Firmicutes | Clostridiales | Peptoniphilus | Peptoniphilus sp. oral taxon 386 | 0.00283 |
| Firmicutes | Clostridiales | Peptoniphilus | Peptoniphilus sp. oral taxon 836 | 0.00022 |
| Firmicutes | Clostridiales | Peptoniphilus | Peptoniphilus timonensis | 0.00036 |
| Firmicutes | Clostridiales | Peptoniphilus | Peptoniphilus unclassified | 0.00151 |
| Firmicutes | Clostridiales | Peptostreptococcaceae noname | Peptostreptococcaceae bacterium ACC19a | 0.00084 |
| Firmicutes | Clostridiales | Peptostreptococcaceae noname | Peptostreptococcaceae bacterium AS15 | 0.00535 |
| Firmicutes | Clostridiales | Peptostreptococcaceae noname | Peptostreptococcaceae bacterium CM2 | 0.00225 |
| Firmicutes | Clostridiales | Peptostreptococcaceae noname | Peptostreptococcaceae bacterium CM5 | 0.00197 |
| Firmicutes | Clostridiales | Peptostreptococcaceae noname | Peptostreptococcaceae bacterium OBRC8 | 0.00072 |
| Firmicutes | Clostridiales | Peptostreptococcaceae noname | Peptostreptococcaceae bacterium oral taxon 113 | 0.00047 |
| Firmicutes | Clostridiales | Peptostreptococcaceae noname | Peptostreptococcaceae bacterium VA2 | 0.28708 |
| Firmicutes | Clostridiales | Peptostreptococcaceae unclassified | Peptostreptococcaceae unclassified | 0.00143 |
| Firmicutes | Clostridiales | Peptostreptococcus | Peptostreptococcus anaerobius | 0.02305 |
| Firmicutes | Clostridiales | Peptostreptococcus | Peptostreptococcus anaerobius CAG:621 | 0.00637 |
| Firmicutes | Clostridiales | Peptostreptococcus | Peptostreptococcus sp. MV1 | 0.03754 |
| Firmicutes | Clostridiales | Peptostreptococcus | Peptostreptococcus stomatis | 0.02403 |
| Firmicutes | Clostridiales | Peptostreptococcus | Peptostreptococcus unclassified | 0.01433 |
| Firmicutes | Clostridiales | Proteiniclasticum | Proteiniclasticum ruminis | 0.00048 |
| Firmicutes | Clostridiales | Proteocatella | Proteocatella sphenisci | 0.00074 |
| Firmicutes | Clostridiales | Pseudobacteroides | Pseudobacteroides cellulosolvens | 0.00088 |
| Firmicutes | Clostridiales | Pseudobutyrivibrio | Pseudobutyrivibrio ruminis | 0.00726 |
| Firmicutes | Clostridiales | Pseudobutyrivibrio | Pseudobutyrivibrio sp. LB2011 | 0.00559 |
| Firmicutes | Clostridiales | Pseudobutyrivibrio | Pseudobutyrivibrio sp. MD2005 | 0.00615 |
| Firmicutes | Clostridiales | Pseudoflavonifractor | Pseudoflavonifractor capillosus | 0.01366 |
| Firmicutes | Clostridiales | Pseudoramibacter | Pseudoramibacter alactolyticus | 0.00062 |
| Firmicutes | Clostridiales | Robinsoniella | Robinsoniella peoriensis | 0.00315 |
| Firmicutes | Clostridiales | Robinsoniella | Robinsoniella sp. KNHs210 | 0.00737 |
| Firmicutes | Clostridiales | Robinsoniella | Robinsoniella unclassified | 0.00126 |
| Firmicutes | Clostridiales | Roseburia | Roseburia hominis | 0.01831 |
| Firmicutes | Clostridiales | Roseburia | Roseburia intestinalis | 0.02232 |
| Firmicutes | Clostridiales | Roseburia | Roseburia intestinalis CAG:13 | 0.00209 |
| Firmicutes | Clostridiales | Roseburia | Roseburia inulinivorans | 0.00380 |
| Firmicutes | Clostridiales | Roseburia | Roseburia inulinivorans CAG:15 | 0.01313 |
| Firmicutes | Clostridiales | Roseburia | Roseburia sp. CAG:100 | 0.00141 |
| Firmicutes | Clostridiales | Roseburia | Roseburia sp. CAG:18 | 0.01117 |
| Firmicutes | Clostridiales | Roseburia | Roseburia sp. CAG:182 | 0.01288 |
| Firmicutes | Clostridiales | Roseburia | Roseburia sp. CAG:197 | 0.00345 |
| Firmicutes | Clostridiales | Roseburia | Roseburia sp. CAG:303 | 0.00452 |
| Firmicutes | Clostridiales | Roseburia | Roseburia sp. CAG:309 | 0.00080 |
| Firmicutes | Clostridiales | Roseburia | Roseburia sp. CAG:380 | 0.00195 |
| Firmicutes | Clostridiales | Roseburia | Roseburia sp. CAG:45 | 0.00064 |
| Firmicutes | Clostridiales | Roseburia | Roseburia sp. CAG:471 | 0.01312 |
| Firmicutes | Clostridiales | Roseburia | Roseburia sp. CAG:50 | 0.00119 |
| Firmicutes | Clostridiales | Roseburia | Roseburia unclassified | 0.00487 |
| Firmicutes | Clostridiales | Ruminiclostridium | [Clostridium] cellobioparum | 0.00033 |
| Firmicutes | Clostridiales | Ruminiclostridium | [Clostridium] cellulolyticum | 0.00006 |
| Firmicutes | Clostridiales | Ruminiclostridium | [Clostridium] cellulosi | 0.00552 |
| Firmicutes | Clostridiales | Ruminiclostridium | [Clostridium] clariflavum | 0.00574 |
| Firmicutes | Clostridiales | Ruminiclostridium | [Clostridium] josui | 0.00022 |
| Firmicutes | Clostridiales | Ruminiclostridium | [Clostridium] leptum | 0.02173 |
| Firmicutes | Clostridiales | Ruminiclostridium | [Clostridium] methylpentosum | 0.00178 |
| Firmicutes | Clostridiales | Ruminiclostridium | [Clostridium] papyrosolvens | 0.03223 |
| Firmicutes | Clostridiales | Ruminiclostridium | [Clostridium] sporosphaeroides | 0.00338 |
| Firmicutes | Clostridiales | Ruminiclostridium | [Clostridium] stercorarium | 0.00035 |
| Firmicutes | Clostridiales | Ruminiclostridium | [Clostridium] straminisolvens | 0.00467 |
| Firmicutes | Clostridiales | Ruminiclostridium | [Clostridium] termitidis | 0.00748 |
| Firmicutes | Clostridiales | Ruminiclostridium | [Clostridium] viride | 0.00855 |
| Firmicutes | Clostridiales | Ruminiclostridium | [Eubacterium] siraeum | 0.00845 |
| Firmicutes | Clostridiales | Ruminiclostridium | Ruminiclostridium thermocellum | 0.00522 |
| Firmicutes | Clostridiales | Ruminiclostridium | Ruminiclostridium unclassified | 0.00641 |
| Firmicutes | Clostridiales | Ruminococcaceae noname | Ruminococcaceae bacterium 585-1 | 0.00958 |
| Firmicutes | Clostridiales | Ruminococcaceae noname | Ruminococcaceae bacterium AB4001 | 0.00572 |
| Firmicutes | Clostridiales | Ruminococcaceae noname | Ruminococcaceae bacterium AE2021 | 0.02825 |
| Firmicutes | Clostridiales | Ruminococcaceae noname | Ruminococcaceae bacterium D16 | 0.03609 |
| Firmicutes | Clostridiales | Ruminococcaceae unclassified | Ruminococcaceae unclassified | 0.01493 |
| Firmicutes | Clostridiales | Ruminococcus | Ruminococcus albus | 0.00694 |
| Firmicutes | Clostridiales | Ruminococcus | Ruminococcus bicirculans | 0.00134 |
| Firmicutes | Clostridiales | Ruminococcus | Ruminococcus bromii | 0.00644 |
| Firmicutes | Clostridiales | Ruminococcus | Ruminococcus callidus | 0.00956 |
| Firmicutes | Clostridiales | Ruminococcus | Ruminococcus champanellensis | 0.00072 |
| Firmicutes | Clostridiales | Ruminococcus | Ruminococcus flavefaciens | 0.00992 |
| Firmicutes | Clostridiales | Ruminococcus | Ruminococcus gauvreauii | 0.01031 |
| Firmicutes | Clostridiales | Ruminococcus | Ruminococcus gnavus CAG:126 | 0.61244 |
| Firmicutes | Clostridiales | Ruminococcus | Ruminococcus lactaris | 0.04594 |
| Firmicutes | Clostridiales | Ruminococcus | Ruminococcus obeum CAG:39 | 0.01166 |
| Firmicutes | Clostridiales | Ruminococcus | Ruminococcus sp. 5 1 39BFAA | 0.00760 |
| Firmicutes | Clostridiales | Ruminococcus | Ruminococcus sp. CAG:108 | 0.01338 |
| Firmicutes | Clostridiales | Ruminococcus | Ruminococcus sp. CAG:17 | 0.02613 |
| Firmicutes | Clostridiales | Ruminococcus | Ruminococcus sp. CAG:177 | 0.00080 |
| Firmicutes | Clostridiales | Ruminococcus | Ruminococcus sp. CAG:254 | 0.00029 |
| Firmicutes | Clostridiales | Ruminococcus | Ruminococcus sp. CAG:353 | 0.00011 |
| Firmicutes | Clostridiales | Ruminococcus | Ruminococcus sp. CAG:379 | 0.00510 |
| Firmicutes | Clostridiales | Ruminococcus | Ruminococcus sp. CAG:382 | 0.00629 |
| Firmicutes | Clostridiales | Ruminococcus | Ruminococcus sp. CAG:403 | 0.00745 |
| Firmicutes | Clostridiales | Ruminococcus | Ruminococcus sp. CAG:488 | 0.00130 |
| Firmicutes | Clostridiales | Ruminococcus | Ruminococcus sp. CAG:55 | 0.00622 |
| Firmicutes | Clostridiales | Ruminococcus | Ruminococcus sp. CAG:563 | 0.01818 |
| Firmicutes | Clostridiales | Ruminococcus | Ruminococcus sp. CAG:57 | 0.01738 |
| Firmicutes | Clostridiales | Ruminococcus | Ruminococcus sp. CAG:579 | 0.00239 |
| Firmicutes | Clostridiales | Ruminococcus | Ruminococcus sp. CAG:60 | 0.01725 |
| Firmicutes | Clostridiales | Ruminococcus | Ruminococcus sp. CAG:624 | 0.00042 |
| Firmicutes | Clostridiales | Ruminococcus | Ruminococcus sp. CAG:9 | 0.00497 |
| Firmicutes | Clostridiales | Ruminococcus | Ruminococcus sp. CAG:90 | 0.00295 |
| Firmicutes | Clostridiales | Ruminococcus | Ruminococcus sp. FC2018 | 0.00045 |
| Firmicutes | Clostridiales | Ruminococcus | Ruminococcus sp. HUN007 | 0.00114 |
| Firmicutes | Clostridiales | Ruminococcus | Ruminococcus sp. JC304 | 0.02100 |
| Firmicutes | Clostridiales | Ruminococcus | Ruminococcus sp. NK3A76 | 0.00007 |
| Firmicutes | Clostridiales | Ruminococcus | Ruminococcus sp. SR1/5 | 0.00911 |
| Firmicutes | Clostridiales | Ruminococcus | Ruminococcus torques CAG:61 | 0.00083 |
| Firmicutes | Clostridiales | Ruminococcus | Ruminococcus unclassified | 0.00108 |
| Firmicutes | Clostridiales | Sedimentibacter | Sedimentibacter sp. B4 | 0.00083 |
| Firmicutes | Clostridiales | Shuttleworthia | Shuttleworthia satelles | 0.00192 |
| Firmicutes | Clostridiales | Shuttleworthia | Shuttleworthia sp. MSX8B | 0.00607 |
| Firmicutes | Clostridiales | Shuttleworthia | Shuttleworthia unclassified | 0.00010 |
| Firmicutes | Clostridiales | Stomatobaculum | Stomatobaculum longum | 0.00264 |
| Firmicutes | Clostridiales | Subdoligranulum | Subdoligranulum sp. 4 3 54A2FAA | 0.01250 |
| Firmicutes | Clostridiales | Subdoligranulum | Subdoligranulum variabile | 0.01876 |
| Firmicutes | Clostridiales | Symbiobacterium | Symbiobacterium thermophilum | 0.00028 |
| Firmicutes | Clostridiales | Syntrophobotulus | Syntrophobotulus glycolicus | 0.00039 |
| Firmicutes | Clostridiales | Syntrophomonas | Syntrophomonas wolfei | 0.00008 |
| Firmicutes | Clostridiales | Syntrophothermus | Syntrophothermus lipocalidus | 0.00010 |
| Firmicutes | Clostridiales | Terrisporobacter | Terrisporobacter glycolicus | 0.06118 |
| Firmicutes | Clostridiales | Terrisporobacter | Terrisporobacter othiniensis | 0.16890 |
| Firmicutes | Clostridiales | Terrisporobacter | Terrisporobacter unclassified | 0.00139 |
| Firmicutes | Clostridiales | Thermincola | Thermincola potens | 0.00006 |
| Firmicutes | Clostridiales | Thermobrachium | Thermobrachium celere | 0.05145 |
| Firmicutes | Clostridiales | Tyzzerella | Tyzzerella nexilis | 0.10561 |
| Firmicutes | Clostridiales | Youngiibacter | Youngiibacter fragilis | 0.00202 |
| Firmicutes | Erysipelotrichales | Allobaculum | Allobaculum stercoricanis | 0.13265 |
| Firmicutes | Erysipelotrichales | Bulleidia | Bulleidia extructa | 0.11879 |
| Firmicutes | Erysipelotrichales | Candidatus Stoquefichus | Candidatus Stoquefichus massiliensis | 0.03132 |
| Firmicutes | Erysipelotrichales | Catenibacterium | Catenibacterium mitsuokai | 0.02497 |
| Firmicutes | Erysipelotrichales | Catenibacterium | Catenibacterium sp. CAG:290 | 0.00628 |
| Firmicutes | Erysipelotrichales | Coprobacillus | Coprobacillus sp. 3 3 56FAA | 0.00178 |
| Firmicutes | Erysipelotrichales | Coprobacillus | Coprobacillus sp. 8 2 54BFAA | 0.00883 |
| Firmicutes | Erysipelotrichales | Coprobacillus | Coprobacillus sp. CAG:183 | 0.00011 |
| Firmicutes | Erysipelotrichales | Coprobacillus | Coprobacillus sp. CAG:235 | 0.00949 |
| Firmicutes | Erysipelotrichales | Coprobacillus | Coprobacillus sp. CAG:605 | 0.00004 |
| Firmicutes | Erysipelotrichales | Coprobacillus | Coprobacillus sp. CAG:698 | 0.00032 |
| Firmicutes | Erysipelotrichales | Coprobacillus | Coprobacillus sp. CAG:826 | 0.00004 |
| Firmicutes | Erysipelotrichales | Coprobacillus | Coprobacillus sp. D6 | 0.00002 |
| Firmicutes | Erysipelotrichales | Coprobacillus | Coprobacillus sp. D7 | 0.01225 |
| Firmicutes | Erysipelotrichales | Coprobacillus | Coprobacillus unclassified | 0.01517 |
| Firmicutes | Erysipelotrichales | Dielma | Dielma fastidiosa | 0.02476 |
| Firmicutes | Erysipelotrichales | Eggerthia | Eggerthia catenaformis | 0.01516 |
| Firmicutes | Erysipelotrichales | Erysipelatoclostridium | [Clostridium] innocuum | 0.12625 |
| Firmicutes | Erysipelotrichales | Erysipelatoclostridium | [Clostridium] saccharogumia | 0.01007 |
| Firmicutes | Erysipelotrichales | Erysipelatoclostridium | [Clostridium] spiroforme | 0.03028 |
| Firmicutes | Erysipelotrichales | Erysipelatoclostridium | Erysipelatoclostridium ramosum | 0.00022 |
| Firmicutes | Erysipelotrichales | Erysipelothrix | Erysipelothrix rhusiopathiae | 0.00018 |
| Firmicutes | Erysipelotrichales | Erysipelothrix | Erysipelothrix tonsillarum | 0.00723 |
| Firmicutes | Erysipelotrichales | Erysipelotrichaceae noname | [Eubacterium] dolichum | 0.14855 |
| Firmicutes | Erysipelotrichales | Erysipelotrichaceae noname | Erysipelotrichaceae bacterium 2 2 44A | 0.00142 |
| Firmicutes | Erysipelotrichales | Erysipelotrichaceae noname | Erysipelotrichaceae bacterium 21 3 | 0.00104 |
| Firmicutes | Erysipelotrichales | Erysipelotrichaceae noname | Erysipelotrichaceae bacterium 3 1 53 | 0.01512 |
| Firmicutes | Erysipelotrichales | Erysipelotrichaceae noname | Erysipelotrichaceae bacterium 5 2 54FAA | 0.01174 |
| Firmicutes | Erysipelotrichales | Erysipelotrichaceae noname | Erysipelotrichaceae bacterium 6 1 45 | 0.00963 |
| Firmicutes | Erysipelotrichales | Erysipelotrichaceae noname | Erysipelotrichaceae bacterium CAG:64 | 0.03934 |
| Firmicutes | Erysipelotrichales | Erysipelotrichaceae noname | Erysipelotrichaceae bacterium NK3D112 | 2.27031 |
| Firmicutes | Erysipelotrichales | Erysipelotrichaceae unclassified | Erysipelotrichaceae unclassified | 0.01884 |
| Firmicutes | Erysipelotrichales | Faecalicoccus | Faecalicoccus pleomorphus | 0.12777 |
| Firmicutes | Erysipelotrichales | Faecalitalea | Faecalitalea cylindroides | 0.31392 |
| Firmicutes | Erysipelotrichales | Holdemanella | Holdemanella biformis | 0.44946 |
| Firmicutes | Erysipelotrichales | Holdemania | Holdemania filiformis | 0.03453 |
| Firmicutes | Erysipelotrichales | Holdemania | Holdemania massiliensis | 0.06721 |
| Firmicutes | Erysipelotrichales | Holdemania | Holdemania unclassified | 0.00045 |
| Firmicutes | Erysipelotrichales | Kandleria | Kandleria vitulina | 0.01363 |
| Firmicutes | Erysipelotrichales | Solobacterium | Solobacterium moorei | 1.90017 |
| Firmicutes | Erysipelotrichales | Turicibacter | Turicibacter sanguinis | 0.00095 |
| Firmicutes | Erysipelotrichales | Turicibacter | Turicibacter sp. HGF1 | 0.00239 |
| Firmicutes | Erysipelotrichales | Turicibacter | Turicibacter unclassified | 0.00058 |
| Firmicutes | Firmicutes noname | Firmicutes noname | Firmicutes bacterium ASF500 | 0.00396 |
| Firmicutes | Firmicutes noname | Firmicutes noname | Firmicutes bacterium CAG:102 | 0.00130 |
| Firmicutes | Firmicutes noname | Firmicutes noname | Firmicutes bacterium CAG:103 | 0.00048 |
| Firmicutes | Firmicutes noname | Firmicutes noname | Firmicutes bacterium CAG:110 | 0.00312 |
| Firmicutes | Firmicutes noname | Firmicutes noname | Firmicutes bacterium CAG:114 | 0.00859 |
| Firmicutes | Firmicutes noname | Firmicutes noname | Firmicutes bacterium CAG:124 | 0.00237 |
| Firmicutes | Firmicutes noname | Firmicutes noname | Firmicutes bacterium CAG:129 | 0.00005 |
| Firmicutes | Firmicutes noname | Firmicutes noname | Firmicutes bacterium CAG:137 | 0.01480 |
| Firmicutes | Firmicutes noname | Firmicutes noname | Firmicutes bacterium CAG:145 | 0.00910 |
| Firmicutes | Firmicutes noname | Firmicutes noname | Firmicutes bacterium CAG:170 | 0.00319 |
| Firmicutes | Firmicutes noname | Firmicutes noname | Firmicutes bacterium CAG:176 | 0.00522 |
| Firmicutes | Firmicutes noname | Firmicutes noname | Firmicutes bacterium CAG:194 | 0.01285 |
| Firmicutes | Firmicutes noname | Firmicutes noname | Firmicutes bacterium CAG:212 | 0.02990 |
| Firmicutes | Firmicutes noname | Firmicutes noname | Firmicutes bacterium CAG:227 | 0.00381 |
| Firmicutes | Firmicutes noname | Firmicutes noname | Firmicutes bacterium CAG:238 | 0.00707 |
| Firmicutes | Firmicutes noname | Firmicutes noname | Firmicutes bacterium CAG:24 | 0.11588 |
| Firmicutes | Firmicutes noname | Firmicutes noname | Firmicutes bacterium CAG:240 | 0.00102 |
| Firmicutes | Firmicutes noname | Firmicutes noname | Firmicutes bacterium CAG:270 | 0.00099 |
| Firmicutes | Firmicutes noname | Firmicutes noname | Firmicutes bacterium CAG:272 | 0.00098 |
| Firmicutes | Firmicutes noname | Firmicutes noname | Firmicutes bacterium CAG:308 | 0.14377 |
| Firmicutes | Firmicutes noname | Firmicutes noname | Firmicutes bacterium CAG:313 | 0.00001 |
| Firmicutes | Firmicutes noname | Firmicutes noname | Firmicutes bacterium CAG:341 | 0.00002 |
| Firmicutes | Firmicutes noname | Firmicutes noname | Firmicutes bacterium CAG:345 | 0.00720 |
| Firmicutes | Firmicutes noname | Firmicutes noname | Firmicutes bacterium CAG:41 | 0.00700 |
| Firmicutes | Firmicutes noname | Firmicutes noname | Firmicutes bacterium CAG:424 | 0.12290 |
| Firmicutes | Firmicutes noname | Firmicutes noname | Firmicutes bacterium CAG:449 | 0.00011 |
| Firmicutes | Firmicutes noname | Firmicutes noname | Firmicutes bacterium CAG:460 | 0.00002 |
| Firmicutes | Firmicutes noname | Firmicutes noname | Firmicutes bacterium CAG:466 | 0.00221 |
| Firmicutes | Firmicutes noname | Firmicutes noname | Firmicutes bacterium CAG:475 | 0.00031 |
| Firmicutes | Firmicutes noname | Firmicutes noname | Firmicutes bacterium CAG:534 | 0.00155 |
| Firmicutes | Firmicutes noname | Firmicutes noname | Firmicutes bacterium CAG:536 | 0.16581 |
| Firmicutes | Firmicutes noname | Firmicutes noname | Firmicutes bacterium CAG:555 | 0.00004 |
| Firmicutes | Firmicutes noname | Firmicutes noname | Firmicutes bacterium CAG:56 | 0.01568 |
| Firmicutes | Firmicutes noname | Firmicutes noname | Firmicutes bacterium CAG:582 | 0.00006 |
| Firmicutes | Firmicutes noname | Firmicutes noname | Firmicutes bacterium CAG:631 | 0.00001 |
| Firmicutes | Firmicutes noname | Firmicutes noname | Firmicutes bacterium CAG:646 | 0.04394 |
| Firmicutes | Firmicutes noname | Firmicutes noname | Firmicutes bacterium CAG:65 | 0.00158 |
| Firmicutes | Firmicutes noname | Firmicutes noname | Firmicutes bacterium CAG:791 | 0.08917 |
| Firmicutes | Firmicutes noname | Firmicutes noname | Firmicutes bacterium CAG:822 | 0.00097 |
| Firmicutes | Firmicutes noname | Firmicutes noname | Firmicutes bacterium CAG:83 | 0.00040 |
| Firmicutes | Firmicutes noname | Firmicutes noname | Firmicutes bacterium CAG:882 | 0.00268 |
| Firmicutes | Firmicutes noname | Firmicutes noname | Firmicutes bacterium CAG:94 | 0.02436 |
| Firmicutes | Firmicutes noname | Firmicutes noname | Firmicutes bacterium CAG:95 | 0.00924 |
| Firmicutes | Firmicutes noname | Firmicutes noname | Firmicutes bacterium JGI 0000112-L22 | 0.00017 |
| Firmicutes | Firmicutes noname | Firmicutes noname | Firmicutes bacterium M10-2 | 0.03771 |
| Firmicutes | Firmicutes unclassified | Firmicutes unclassified | Firmicutes unclassified | 0.08756 |
| Firmicutes | Halanaerobiales | Halanaerobium | Halanaerobium hydrogeniformans | 0.00243 |
| Firmicutes | Halanaerobiales | Halanaerobium | Halanaerobium praevalens | 0.00014 |
| Firmicutes | Halanaerobiales | Halanaerobium | Halanaerobium saccharolyticum | 0.00003 |
| Firmicutes | Halanaerobiales | Halobacteroides | Halobacteroides halobius | 0.00010 |
| Firmicutes | Halanaerobiales | Halothermothrix | Halothermothrix orenii | 0.00002 |
| Firmicutes | Halanaerobiales | Orenia | Orenia marismortui | 0.00015 |
| Firmicutes | Lactobacillales | Abiotrophia | Abiotrophia defectiva | 0.02280 |
| Firmicutes | Lactobacillales | Aerococcus | Aerococcus urinae | 0.00025 |
| Firmicutes | Lactobacillales | Aerococcus | Aerococcus urinaeequi | 0.00012 |
| Firmicutes | Lactobacillales | Aerococcus | Aerococcus viridans | 0.00020 |
| Firmicutes | Lactobacillales | Alkalibacterium | Alkalibacterium sp. AK22 | 0.00023 |
| Firmicutes | Lactobacillales | Allofustis | Allofustis seminis | 0.00290 |
| Firmicutes | Lactobacillales | Alloiococcus | Alloiococcus otitis | 0.00032 |
| Firmicutes | Lactobacillales | Atopobacter | Atopobacter phocae | 0.00044 |
| Firmicutes | Lactobacillales | Atopococcus | Atopococcus tabaci | 0.00036 |
| Firmicutes | Lactobacillales | Bavariicoccus | Bavariicoccus seileri | 0.00079 |
| Firmicutes | Lactobacillales | Carnobacterium | Carnobacterium alterfunditum | 0.00004 |
| Firmicutes | Lactobacillales | Carnobacterium | Carnobacterium divergens | 0.00023 |
| Firmicutes | Lactobacillales | Carnobacterium | Carnobacterium gallinarum | 0.00080 |
| Firmicutes | Lactobacillales | Carnobacterium | Carnobacterium inhibens | 0.00013 |
| Firmicutes | Lactobacillales | Carnobacterium | Carnobacterium jeotgali | 0.00003 |
| Firmicutes | Lactobacillales | Carnobacterium | Carnobacterium maltaromaticum | 0.00067 |
| Firmicutes | Lactobacillales | Carnobacterium | Carnobacterium mobile | 0.00040 |
| Firmicutes | Lactobacillales | Carnobacterium | Carnobacterium pleistocenium | 0.00006 |
| Firmicutes | Lactobacillales | Carnobacterium | Carnobacterium sp. 17-4 | 0.00017 |
| Firmicutes | Lactobacillales | Carnobacterium | Carnobacterium sp. AT7 | 0.00004 |
| Firmicutes | Lactobacillales | Carnobacterium | Carnobacterium sp. WN1359 | 0.00011 |
| Firmicutes | Lactobacillales | Carnobacterium | Carnobacterium sp. WN1374 | 0.00005 |
| Firmicutes | Lactobacillales | Carnobacterium | Carnobacterium unclassified | 0.00007 |
| Firmicutes | Lactobacillales | Dolosigranulum | Dolosigranulum pigrum | 0.00037 |
| Firmicutes | Lactobacillales | Enterococcus | Enterococcus asini | 0.00040 |
| Firmicutes | Lactobacillales | Enterococcus | Enterococcus avium | 0.00028 |
| Firmicutes | Lactobacillales | Enterococcus | Enterococcus caccae | 0.00045 |
| Firmicutes | Lactobacillales | Enterococcus | Enterococcus casseliflavus | 0.00040 |
| Firmicutes | Lactobacillales | Enterococcus | Enterococcus cecorum | 0.02024 |
| Firmicutes | Lactobacillales | Enterococcus | Enterococcus columbae | 0.00328 |
| Firmicutes | Lactobacillales | Enterococcus | Enterococcus dispar | 0.00020 |
| Firmicutes | Lactobacillales | Enterococcus | Enterococcus durans | 0.00305 |
| Firmicutes | Lactobacillales | Enterococcus | Enterococcus faecalis | 0.00558 |
| Firmicutes | Lactobacillales | Enterococcus | Enterococcus faecium | 0.04515 |
| Firmicutes | Lactobacillales | Enterococcus | Enterococcus gallinarum | 0.00057 |
| Firmicutes | Lactobacillales | Enterococcus | Enterococcus gilvus | 0.00001 |
| Firmicutes | Lactobacillales | Enterococcus | Enterococcus haemoperoxidus | 0.00182 |
| Firmicutes | Lactobacillales | Enterococcus | Enterococcus hirae | 0.00007 |
| Firmicutes | Lactobacillales | Enterococcus | Enterococcus italicus | 0.00020 |
| Firmicutes | Lactobacillales | Enterococcus | Enterococcus malodoratus | 0.00046 |
| Firmicutes | Lactobacillales | Enterococcus | Enterococcus moraviensis | 0.00004 |
| Firmicutes | Lactobacillales | Enterococcus | Enterococcus mundtii | 0.00024 |
| Firmicutes | Lactobacillales | Enterococcus | Enterococcus pallens | 0.00097 |
| Firmicutes | Lactobacillales | Enterococcus | Enterococcus phoeniculicola | 0.00085 |
| Firmicutes | Lactobacillales | Enterococcus | Enterococcus raffinosus | 0.00518 |
| Firmicutes | Lactobacillales | Enterococcus | Enterococcus saccharolyticus | 0.00155 |
| Firmicutes | Lactobacillales | Enterococcus | Enterococcus sp. C1 | 0.00004 |
| Firmicutes | Lactobacillales | Enterococcus | Enterococcus sp. HSIEG1 | 0.00002 |
| Firmicutes | Lactobacillales | Enterococcus | Enterococcus sp. TR | 0.00266 |
| Firmicutes | Lactobacillales | Enterococcus | Enterococcus sulfureus | 0.00019 |
| Firmicutes | Lactobacillales | Enterococcus | Enterococcus unclassified | 0.00215 |
| Firmicutes | Lactobacillales | Enterococcus | Enterococcus villorum | 0.00027 |
| Firmicutes | Lactobacillales | Eremococcus | Eremococcus coleocola | 0.00330 |
| Firmicutes | Lactobacillales | Facklamia | Facklamia hominis | 0.00136 |
| Firmicutes | Lactobacillales | Facklamia | Facklamia ignava | 0.00134 |
| Firmicutes | Lactobacillales | Facklamia | Facklamia languida | 0.00151 |
| Firmicutes | Lactobacillales | Facklamia | Facklamia sourekii | 0.00265 |
| Firmicutes | Lactobacillales | Granulicatella | Granulicatella adiacens | 0.00378 |
| Firmicutes | Lactobacillales | Granulicatella | Granulicatella elegans | 0.00567 |
| Firmicutes | Lactobacillales | Lacticigenium | Lacticigenium naphtae | 0.00013 |
| Firmicutes | Lactobacillales | Lactobacillaceae unclassified | Lactobacillaceae unclassified | 0.00004 |
| Firmicutes | Lactobacillales | Lactobacillales unclassified | Lactobacillales unclassified | 0.00043 |
| Firmicutes | Lactobacillales | Lactobacillus | Lactobacillus acidipiscis | 0.00003 |
| Firmicutes | Lactobacillales | Lactobacillus | Lactobacillus acidophilus | 0.00005 |
| Firmicutes | Lactobacillales | Lactobacillus | Lactobacillus amylovorus | 0.00002 |
| Firmicutes | Lactobacillales | Lactobacillus | Lactobacillus animalis | 0.00007 |
| Firmicutes | Lactobacillales | Lactobacillus | Lactobacillus apodemi | 0.00005 |
| Firmicutes | Lactobacillales | Lactobacillus | Lactobacillus brevis | 0.00034 |
| Firmicutes | Lactobacillales | Lactobacillus | Lactobacillus buchneri | 0.00011 |
| Firmicutes | Lactobacillales | Lactobacillus | Lactobacillus casei | 0.00012 |
| Firmicutes | Lactobacillales | Lactobacillus | Lactobacillus ceti | 0.00071 |
| Firmicutes | Lactobacillales | Lactobacillus | Lactobacillus composti | 0.00003 |
| Firmicutes | Lactobacillales | Lactobacillus | Lactobacillus coryniformis | 0.00008 |
| Firmicutes | Lactobacillales | Lactobacillus | Lactobacillus crispatus | 0.00571 |
| Firmicutes | Lactobacillales | Lactobacillus | Lactobacillus curvatus | 0.00004 |
| Firmicutes | Lactobacillales | Lactobacillus | Lactobacillus delbrueckii | 0.01141 |
| Firmicutes | Lactobacillales | Lactobacillus | Lactobacillus equi | 0.00009 |
| Firmicutes | Lactobacillales | Lactobacillus | Lactobacillus equicursoris | 0.01040 |
| Firmicutes | Lactobacillales | Lactobacillus | Lactobacillus fabifermentans | 0.00008 |
| Firmicutes | Lactobacillales | Lactobacillus | Lactobacillus farciminis | 0.00016 |
| Firmicutes | Lactobacillales | Lactobacillus | Lactobacillus fermentum | 0.00066 |
| Firmicutes | Lactobacillales | Lactobacillus | Lactobacillus fuchuensis | 0.00005 |
| Firmicutes | Lactobacillales | Lactobacillus | Lactobacillus gasseri | 0.00569 |
| Firmicutes | Lactobacillales | Lactobacillus | Lactobacillus gastricus | 0.00006 |
| Firmicutes | Lactobacillales | Lactobacillus | Lactobacillus gigeriorum | 0.00004 |
| Firmicutes | Lactobacillales | Lactobacillus | Lactobacillus hamsteri | 0.00001 |
| Firmicutes | Lactobacillales | Lactobacillus | Lactobacillus harbinensis | 0.00004 |
| Firmicutes | Lactobacillales | Lactobacillus | Lactobacillus helveticus | 0.00618 |
| Firmicutes | Lactobacillales | Lactobacillus | Lactobacillus hokkaidonensis | 0.00003 |
| Firmicutes | Lactobacillales | Lactobacillus | Lactobacillus hominis | 0.00075 |
| Firmicutes | Lactobacillales | Lactobacillus | Lactobacillus iners | 0.00033 |
| Firmicutes | Lactobacillales | Lactobacillus | Lactobacillus jensenii | 0.00001 |
| Firmicutes | Lactobacillales | Lactobacillus | Lactobacillus kefiranofaciens | 0.00007 |
| Firmicutes | Lactobacillales | Lactobacillus | Lactobacillus kisonensis | 0.00006 |
| Firmicutes | Lactobacillales | Lactobacillus | Lactobacillus kullabergensis | 0.00008 |
| Firmicutes | Lactobacillales | Lactobacillus | Lactobacillus mali | 0.00012 |
| Firmicutes | Lactobacillales | Lactobacillus | Lactobacillus mellis | 0.00005 |
| Firmicutes | Lactobacillales | Lactobacillus | Lactobacillus mucosae | 0.00015 |
| Firmicutes | Lactobacillales | Lactobacillus | Lactobacillus nodensis | 0.00136 |
| Firmicutes | Lactobacillales | Lactobacillus | Lactobacillus oris | 0.00004 |
| Firmicutes | Lactobacillales | Lactobacillus | Lactobacillus oryzae | 0.00003 |
| Firmicutes | Lactobacillales | Lactobacillus | Lactobacillus parabrevis | 0.00130 |
| Firmicutes | Lactobacillales | Lactobacillus | Lactobacillus paracasei | 0.00005 |
| Firmicutes | Lactobacillales | Lactobacillus | Lactobacillus paralimentarius | 0.00079 |
| Firmicutes | Lactobacillales | Lactobacillus | Lactobacillus pasteurii | 0.00024 |
| Firmicutes | Lactobacillales | Lactobacillus | Lactobacillus plantarum | 0.00189 |
| Firmicutes | Lactobacillales | Lactobacillus | Lactobacillus pobuzihii | 0.00004 |
| Firmicutes | Lactobacillales | Lactobacillus | Lactobacillus reuteri | 0.00492 |
| Firmicutes | Lactobacillales | Lactobacillus | Lactobacillus rossiae | 0.00019 |
| Firmicutes | Lactobacillales | Lactobacillus | Lactobacillus ruminis | 0.00569 |
| Firmicutes | Lactobacillales | Lactobacillus | Lactobacillus ruminis CAG:367 | 0.00229 |
| Firmicutes | Lactobacillales | Lactobacillus | Lactobacillus saerimneri | 0.00133 |
| Firmicutes | Lactobacillales | Lactobacillus | Lactobacillus sakei | 0.00001 |
| Firmicutes | Lactobacillales | Lactobacillus | Lactobacillus salivarius | 0.00021 |
| Firmicutes | Lactobacillales | Lactobacillus | Lactobacillus sanfranciscensis | 0.00002 |
| Firmicutes | Lactobacillales | Lactobacillus | Lactobacillus sp. 7 1 47FAA | 0.00058 |
| Firmicutes | Lactobacillales | Lactobacillus | Lactobacillus sp. WDC04 | 0.00004 |
| Firmicutes | Lactobacillales | Lactobacillus | Lactobacillus spicheri | 0.00005 |
| Firmicutes | Lactobacillales | Lactobacillus | Lactobacillus sucicola | 0.00017 |
| Firmicutes | Lactobacillales | Lactobacillus | Lactobacillus suebicus | 0.00506 |
| Firmicutes | Lactobacillales | Lactobacillus | Lactobacillus ultunensis | 0.00268 |
| Firmicutes | Lactobacillales | Lactobacillus | Lactobacillus unclassified | 0.01096 |
| Firmicutes | Lactobacillales | Lactobacillus | Lactobacillus zeae | 0.00320 |
| Firmicutes | Lactobacillales | Lactococcus | Lactococcus garvieae | 0.00082 |
| Firmicutes | Lactobacillales | Lactococcus | Lactococcus lactis | 0.00287 |
| Firmicutes | Lactobacillales | Lactococcus | Lactococcus piscium | 0.00033 |
| Firmicutes | Lactobacillales | Lactococcus | Lactococcus raffinolactis | 0.00656 |
| Firmicutes | Lactobacillales | Lactococcus | Lactococcus unclassified | 0.00008 |
| Firmicutes | Lactobacillales | Leuconostoc | Leuconostoc citreum | 0.00012 |
| Firmicutes | Lactobacillales | Leuconostoc | Leuconostoc fallax | 0.00002 |
| Firmicutes | Lactobacillales | Leuconostoc | Leuconostoc lactis | 0.00006 |
| Firmicutes | Lactobacillales | Leuconostoc | Leuconostoc mesenteroides | 0.00014 |
| Firmicutes | Lactobacillales | Leuconostoc | Leuconostoc sp. DORA 2 | 0.00008 |
| Firmicutes | Lactobacillales | Melissococcus | Melissococcus plutonius | 0.00002 |
| Firmicutes | Lactobacillales | Oenococcus | Oenococcus oeni | 0.00002 |
| Firmicutes | Lactobacillales | Pediococcus | Pediococcus unclassified | 0.00006 |
| Firmicutes | Lactobacillales | Sharpea | Sharpea azabuensis | 0.21908 |
| Firmicutes | Lactobacillales | Streptococcus | Streptococcus agalactiae | 0.02943 |
| Firmicutes | Lactobacillales | Streptococcus | Streptococcus anginosus | 0.00558 |
| Firmicutes | Lactobacillales | Streptococcus | Streptococcus australis | 0.00073 |
| Firmicutes | Lactobacillales | Streptococcus | Streptococcus caballi | 0.00894 |
| Firmicutes | Lactobacillales | Streptococcus | Streptococcus canis | 0.00655 |
| Firmicutes | Lactobacillales | Streptococcus | Streptococcus castoreus | 0.00318 |
| Firmicutes | Lactobacillales | Streptococcus | Streptococcus constellatus | 0.00265 |
| Firmicutes | Lactobacillales | Streptococcus | Streptococcus criceti | 0.00410 |
| Firmicutes | Lactobacillales | Streptococcus | Streptococcus cristatus | 0.00111 |
| Firmicutes | Lactobacillales | Streptococcus | Streptococcus dentisani | 0.00014 |
| Firmicutes | Lactobacillales | Streptococcus | Streptococcus devriesei | 0.00723 |
| Firmicutes | Lactobacillales | Streptococcus | Streptococcus didelphis | 0.00187 |
| Firmicutes | Lactobacillales | Streptococcus | Streptococcus downei | 0.00053 |
| Firmicutes | Lactobacillales | Streptococcus | Streptococcus dysgalactiae | 0.00722 |
| Firmicutes | Lactobacillales | Streptococcus | Streptococcus entericus | 0.02423 |
| Firmicutes | Lactobacillales | Streptococcus | Streptococcus equi | 0.01358 |
| Firmicutes | Lactobacillales | Streptococcus | Streptococcus equinus | 0.01424 |
| Firmicutes | Lactobacillales | Streptococcus | Streptococcus ferus | 0.01220 |
| Firmicutes | Lactobacillales | Streptococcus | Streptococcus gallolyticus | 0.01156 |
| Firmicutes | Lactobacillales | Streptococcus | Streptococcus gordonii | 0.00213 |
| Firmicutes | Lactobacillales | Streptococcus | Streptococcus henryi | 0.01261 |
| Firmicutes | Lactobacillales | Streptococcus | Streptococcus hongkongensis | 0.01741 |
| Firmicutes | Lactobacillales | Streptococcus | Streptococcus hyovaginalis | 0.00371 |
| Firmicutes | Lactobacillales | Streptococcus | Streptococcus ictaluri | 0.00270 |
| Firmicutes | Lactobacillales | Streptococcus | Streptococcus infantarius | 0.00253 |
| Firmicutes | Lactobacillales | Streptococcus | Streptococcus infantis | 0.00135 |
| Firmicutes | Lactobacillales | Streptococcus | Streptococcus iniae | 0.00368 |
| Firmicutes | Lactobacillales | Streptococcus | Streptococcus intermedius | 0.00280 |
| Firmicutes | Lactobacillales | Streptococcus | Streptococcus lutetiensis | 0.01172 |
| Firmicutes | Lactobacillales | Streptococcus | Streptococcus macacae | 0.00427 |
| Firmicutes | Lactobacillales | Streptococcus | Streptococcus macedonicus | 0.00140 |
| Firmicutes | Lactobacillales | Streptococcus | Streptococcus marimammalium | 0.01402 |
| Firmicutes | Lactobacillales | Streptococcus | Streptococcus massiliensis | 0.00209 |
| Firmicutes | Lactobacillales | Streptococcus | Streptococcus merionis | 0.01076 |
| Firmicutes | Lactobacillales | Streptococcus | Streptococcus minor | 0.01146 |
| Firmicutes | Lactobacillales | Streptococcus | Streptococcus mitis | 0.01188 |
| Firmicutes | Lactobacillales | Streptococcus | Streptococcus mutans | 0.00857 |
| Firmicutes | Lactobacillales | Streptococcus | Streptococcus oligofermentans | 0.00126 |
| Firmicutes | Lactobacillales | Streptococcus | Streptococcus oralis | 0.00193 |
| Firmicutes | Lactobacillales | Streptococcus | Streptococcus orisratti | 0.00811 |
| Firmicutes | Lactobacillales | Streptococcus | Streptococcus ovis | 0.00733 |
| Firmicutes | Lactobacillales | Streptococcus | Streptococcus parasanguinis | 0.00351 |
| Firmicutes | Lactobacillales | Streptococcus | Streptococcus parauberis | 0.00761 |
| Firmicutes | Lactobacillales | Streptococcus | Streptococcus pasteurianus | 0.00060 |
| Firmicutes | Lactobacillales | Streptococcus | Streptococcus peroris | 0.00604 |
| Firmicutes | Lactobacillales | Streptococcus | Streptococcus phocae | 0.00100 |
| Firmicutes | Lactobacillales | Streptococcus | Streptococcus plurextorum | 0.00852 |
| Firmicutes | Lactobacillales | Streptococcus | Streptococcus pneumoniae | 0.00714 |
| Firmicutes | Lactobacillales | Streptococcus | Streptococcus porci | 0.02757 |
| Firmicutes | Lactobacillales | Streptococcus | Streptococcus porcinus | 0.00867 |
| Firmicutes | Lactobacillales | Streptococcus | Streptococcus pseudopneumoniae | 0.00588 |
| Firmicutes | Lactobacillales | Streptococcus | Streptococcus pseudoporcinus | 0.00143 |
| Firmicutes | Lactobacillales | Streptococcus | Streptococcus pyogenes | 0.00655 |
| Firmicutes | Lactobacillales | Streptococcus | Streptococcus ratti | 0.00479 |
| Firmicutes | Lactobacillales | Streptococcus | Streptococcus salivarius | 0.00638 |
| Firmicutes | Lactobacillales | Streptococcus | Streptococcus salivarius CAG:79 | 0.00004 |
| Firmicutes | Lactobacillales | Streptococcus | Streptococcus sanguinis | 0.00701 |
| Firmicutes | Lactobacillales | Streptococcus | Streptococcus sinensis | 0.00744 |
| Firmicutes | Lactobacillales | Streptococcus | Streptococcus sobrinus | 0.00689 |
| Firmicutes | Lactobacillales | Streptococcus | Streptococcus sp. 2 1 36FAA | 0.00032 |
| Firmicutes | Lactobacillales | Streptococcus | Streptococcus sp. ACS2 | 0.00057 |
| Firmicutes | Lactobacillales | Streptococcus | Streptococcus sp. AS14 | 0.00006 |
| Firmicutes | Lactobacillales | Streptococcus | Streptococcus sp. AS20 | 0.00032 |
| Firmicutes | Lactobacillales | Streptococcus | Streptococcus sp. C150 | 0.00046 |
| Firmicutes | Lactobacillales | Streptococcus | Streptococcus sp. C300 | 0.00018 |
| Firmicutes | Lactobacillales | Streptococcus | Streptococcus sp. CM6 | 0.00004 |
| Firmicutes | Lactobacillales | Streptococcus | Streptococcus sp. CM7 | 0.00051 |
| Firmicutes | Lactobacillales | Streptococcus | Streptococcus sp. DBCMS | 0.00008 |
| Firmicutes | Lactobacillales | Streptococcus | Streptococcus sp. DORA 10 | 0.00004 |
| Firmicutes | Lactobacillales | Streptococcus | Streptococcus sp. F0441 | 0.00023 |
| Firmicutes | Lactobacillales | Streptococcus | Streptococcus sp. F0442 | 0.00092 |
| Firmicutes | Lactobacillales | Streptococcus | Streptococcus sp. GMD5S | 0.00002 |
| Firmicutes | Lactobacillales | Streptococcus | Streptococcus sp. GMD6S | 0.00006 |
| Firmicutes | Lactobacillales | Streptococcus | Streptococcus sp. HPH0090 | 0.00034 |
| Firmicutes | Lactobacillales | Streptococcus | Streptococcus sp. HSISB1 | 0.00020 |
| Firmicutes | Lactobacillales | Streptococcus | Streptococcus sp. HSISM1 | 0.00018 |
| Firmicutes | Lactobacillales | Streptococcus | Streptococcus sp. HSISS1 | 0.00006 |
| Firmicutes | Lactobacillales | Streptococcus | Streptococcus sp. HSISS2 | 0.00026 |
| Firmicutes | Lactobacillales | Streptococcus | Streptococcus sp. HSISS3 | 0.00033 |
| Firmicutes | Lactobacillales | Streptococcus | Streptococcus sp. HSISS4 | 0.00004 |
| Firmicutes | Lactobacillales | Streptococcus | Streptococcus sp. I-G2 | 0.00047 |
| Firmicutes | Lactobacillales | Streptococcus | Streptococcus sp. I-P16 | 0.00076 |
| Firmicutes | Lactobacillales | Streptococcus | Streptococcus sp. M143 | 0.00016 |
| Firmicutes | Lactobacillales | Streptococcus | Streptococcus sp. M334 | 0.00021 |
| Firmicutes | Lactobacillales | Streptococcus | Streptococcus sp. OBRC6 | 0.00059 |
| Firmicutes | Lactobacillales | Streptococcus | Streptococcus sp. oral taxon 056 | 0.00067 |
| Firmicutes | Lactobacillales | Streptococcus | Streptococcus sp. oral taxon 058 | 0.00019 |
| Firmicutes | Lactobacillales | Streptococcus | Streptococcus sp. oral taxon 071 | 0.00021 |
| Firmicutes | Lactobacillales | Streptococcus | Streptococcus sp. SK140 | 0.00031 |
| Firmicutes | Lactobacillales | Streptococcus | Streptococcus sp. SK643 | 0.00042 |
| Firmicutes | Lactobacillales | Streptococcus | Streptococcus sp. SR1 | 0.00004 |
| Firmicutes | Lactobacillales | Streptococcus | Streptococcus sp. SR4 | 0.00035 |
| Firmicutes | Lactobacillales | Streptococcus | Streptococcus sp. VT 162 | 0.00006 |
| Firmicutes | Lactobacillales | Streptococcus | Streptococcus suis | 0.10265 |
| Firmicutes | Lactobacillales | Streptococcus | Streptococcus thermophilus | 0.00195 |
| Firmicutes | Lactobacillales | Streptococcus | Streptococcus thoraltensis | 0.00643 |
| Firmicutes | Lactobacillales | Streptococcus | Streptococcus tigurinus | 0.00025 |
| Firmicutes | Lactobacillales | Streptococcus | Streptococcus uberis | 0.02558 |
| Firmicutes | Lactobacillales | Streptococcus | Streptococcus unclassified | 0.03152 |
| Firmicutes | Lactobacillales | Streptococcus | Streptococcus urinalis | 0.00342 |
| Firmicutes | Lactobacillales | Streptococcus | Streptococcus vestibularis | 0.00234 |
| Firmicutes | Lactobacillales | Tetragenococcus | Tetragenococcus halophilus | 0.00018 |
| Firmicutes | Lactobacillales | Tetragenococcus | Tetragenococcus muriaticus | 0.00070 |
| Firmicutes | Lactobacillales | Vagococcus | Vagococcus lutrae | 0.00097 |
| Firmicutes | Lactobacillales | Weissella | Weissella cibaria | 0.00014 |
| Firmicutes | Lactobacillales | Weissella | Weissella confusa | 0.00009 |
| Firmicutes | Lactobacillales | Weissella | Weissella hellenica | 0.00006 |
| Firmicutes | Lactobacillales | Weissella | Weissella koreensis | 0.00005 |
| Firmicutes | Lactobacillales | Weissella | Weissella thailandensis | 0.00001 |
| Firmicutes | Natranaerobiales | Natranaerobius | Natranaerobius thermophilus | 0.00013 |
| Firmicutes | Selenomonadales | Acetonema | Acetonema longum | 0.00388 |
| Firmicutes | Selenomonadales | Acidaminococcus | Acidaminococcus fermentans | 0.00428 |
| Firmicutes | Selenomonadales | Acidaminococcus | Acidaminococcus intestini | 0.00087 |
| Firmicutes | Selenomonadales | Acidaminococcus | Acidaminococcus intestini CAG:325 | 0.00026 |
| Firmicutes | Selenomonadales | Acidaminococcus | Acidaminococcus sp. BV3L6 | 0.00002 |
| Firmicutes | Selenomonadales | Acidaminococcus | Acidaminococcus sp. CAG:542 | 0.00350 |
| Firmicutes | Selenomonadales | Acidaminococcus | Acidaminococcus sp. CAG:917 | 0.00008 |
| Firmicutes | Selenomonadales | Acidaminococcus | Acidaminococcus sp. D21 | 0.00001 |
| Firmicutes | Selenomonadales | Acidaminococcus | Acidaminococcus unclassified | 0.00002 |
| Firmicutes | Selenomonadales | Anaeroarcus | Anaeroarcus burkinensis | 0.00004 |
| Firmicutes | Selenomonadales | Anaeroglobus | Anaeroglobus geminatus | 0.00116 |
| Firmicutes | Selenomonadales | Anaeromusa | Anaeromusa acidaminophila | 0.00030 |
| Firmicutes | Selenomonadales | Anaerovibrio | Anaerovibrio lipolyticus | 0.00815 |
| Firmicutes | Selenomonadales | Anaerovibrio | Anaerovibrio sp. RM50 | 0.00096 |
| Firmicutes | Selenomonadales | Centipeda | Centipeda periodontii | 0.00009 |
| Firmicutes | Selenomonadales | Dialister | Dialister invisus | 0.00091 |
| Firmicutes | Selenomonadales | Dialister | Dialister invisus CAG:218 | 0.00020 |
| Firmicutes | Selenomonadales | Dialister | Dialister micraerophilus | 0.00020 |
| Firmicutes | Selenomonadales | Dialister | Dialister sp. CAG:357 | 0.02934 |
| Firmicutes | Selenomonadales | Dialister | Dialister sp. CAG:486 | 0.00326 |
| Firmicutes | Selenomonadales | Dialister | Dialister succinatiphilus | 0.00012 |
| Firmicutes | Selenomonadales | Megamonas | Megamonas funiformis | 0.93109 |
| Firmicutes | Selenomonadales | Megamonas | Megamonas funiformis CAG:377 | 0.49101 |
| Firmicutes | Selenomonadales | Megamonas | Megamonas hypermegale | 0.25619 |
| Firmicutes | Selenomonadales | Megamonas | Megamonas rupellensis | 0.68236 |
| Firmicutes | Selenomonadales | Megamonas | Megamonas unclassified | 0.71316 |
| Firmicutes | Selenomonadales | Megasphaera | Megasphaera elsdenii | 0.00148 |
| Firmicutes | Selenomonadales | Megasphaera | Megasphaera elsdenii CAG:570 | 0.00128 |
| Firmicutes | Selenomonadales | Megasphaera | Megasphaera genomosp. type 1 | 0.00004 |
| Firmicutes | Selenomonadales | Megasphaera | Megasphaera micronuciformis | 0.00045 |
| Firmicutes | Selenomonadales | Megasphaera | Megasphaera sp. BL7 | 0.15919 |
| Firmicutes | Selenomonadales | Megasphaera | Megasphaera sp. BV3C16-1 | 0.01060 |
| Firmicutes | Selenomonadales | Megasphaera | Megasphaera sp. NP3 | 0.00371 |
| Firmicutes | Selenomonadales | Megasphaera | Megasphaera sp. UPII 135-E | 0.00126 |
| Firmicutes | Selenomonadales | Megasphaera | Megasphaera sp. UPII 199-6 | 0.00011 |
| Firmicutes | Selenomonadales | Megasphaera | Megasphaera unclassified | 0.00144 |
| Firmicutes | Selenomonadales | Mitsuokella | Mitsuokella jalaludinii | 0.00290 |
| Firmicutes | Selenomonadales | Mitsuokella | Mitsuokella multacida | 0.00251 |
| Firmicutes | Selenomonadales | Mitsuokella | Mitsuokella sp. oral taxon 131 | 0.00150 |
| Firmicutes | Selenomonadales | Negativicoccus | Negativicoccus succinicivorans | 0.00222 |
| Firmicutes | Selenomonadales | Pelosinus | Pelosinus fermentans | 0.02615 |
| Firmicutes | Selenomonadales | Pelosinus | Pelosinus sp. UFO1 | 0.00910 |
| Firmicutes | Selenomonadales | Pelosinus | Pelosinus unclassified | 0.00264 |
| Firmicutes | Selenomonadales | Phascolarctobacterium | Phascolarctobacterium sp. CAG:207 | 0.04052 |
| Firmicutes | Selenomonadales | Phascolarctobacterium | Phascolarctobacterium sp. CAG:266 | 0.00484 |
| Firmicutes | Selenomonadales | Phascolarctobacterium | Phascolarctobacterium succinatutens | 0.01475 |
| Firmicutes | Selenomonadales | Propionispira | Propionispira raffinosivorans | 0.01409 |
| Firmicutes | Selenomonadales | Selenomonadales unclassified | Selenomonadales unclassified | 0.00002 |
| Firmicutes | Selenomonadales | Selenomonas | Selenomonas artemidis | 0.00083 |
| Firmicutes | Selenomonadales | Selenomonas | Selenomonas bovis | 0.00816 |
| Firmicutes | Selenomonadales | Selenomonas | Selenomonas flueggei | 0.00013 |
| Firmicutes | Selenomonadales | Selenomonas | Selenomonas infelix | 0.00040 |
| Firmicutes | Selenomonadales | Selenomonas | Selenomonas noxia | 0.00013 |
| Firmicutes | Selenomonadales | Selenomonas | Selenomonas ruminantium | 0.02101 |
| Firmicutes | Selenomonadales | Selenomonas | Selenomonas sp. AE3005 | 0.00207 |
| Firmicutes | Selenomonadales | Selenomonas | Selenomonas sp. CM52 | 0.00047 |
| Firmicutes | Selenomonadales | Selenomonas | Selenomonas sp. FC4001 | 0.00087 |
| Firmicutes | Selenomonadales | Selenomonas | Selenomonas sp. FOBRC6 | 0.00083 |
| Firmicutes | Selenomonadales | Selenomonas | Selenomonas sp. FOBRC9 | 0.00023 |
| Firmicutes | Selenomonadales | Selenomonas | Selenomonas sp. ND2010 | 0.00189 |
| Firmicutes | Selenomonadales | Selenomonas | Selenomonas sp. oral taxon 137 | 0.00107 |
| Firmicutes | Selenomonadales | Selenomonas | Selenomonas sp. oral taxon 138 | 0.01100 |
| Firmicutes | Selenomonadales | Selenomonas | Selenomonas sp. oral taxon 892 | 0.00049 |
| Firmicutes | Selenomonadales | Selenomonas | Selenomonas sputigena | 0.00166 |
| Firmicutes | Selenomonadales | Sporomusa | Sporomusa ovata | 0.00151 |
| Firmicutes | Selenomonadales | Succinispira | Succinispira mobilis | 0.00003 |
| Firmicutes | Selenomonadales | Thermosinus | Thermosinus carboxydivorans | 0.00007 |
| Firmicutes | Selenomonadales | Veillonella | Veillonella atypica | 0.00033 |
| Firmicutes | Selenomonadales | Veillonella | Veillonella dispar | 0.00246 |
| Firmicutes | Selenomonadales | Veillonella | Veillonella magna | 0.00017 |
| Firmicutes | Selenomonadales | Veillonella | Veillonella montpellierensis | 0.00439 |
| Firmicutes | Selenomonadales | Veillonella | Veillonella parvula | 0.00013 |
| Firmicutes | Selenomonadales | Veillonella | Veillonella ratti | 0.00053 |
| Firmicutes | Selenomonadales | Veillonella | Veillonella sp. 3 1 44 | 0.00010 |
| Firmicutes | Selenomonadales | Veillonella | Veillonella sp. 6 1 27 | 0.00040 |
| Firmicutes | Selenomonadales | Veillonella | Veillonella sp. CAG:933 | 0.02202 |
| Firmicutes | Selenomonadales | Veillonella | Veillonella sp. DORA A 3 16 22 | 0.00014 |
| Firmicutes | Selenomonadales | Veillonella | Veillonella sp. DORA B 18 19 23 | 0.00134 |
| Firmicutes | Selenomonadales | Veillonella | Veillonella sp. ICM51a | 0.00009 |
| Firmicutes | Selenomonadales | Veillonella | Veillonella sp. oral taxon 780 | 0.00013 |
| Firmicutes | Selenomonadales | Veillonella | Veillonella unclassified | 0.00023 |
| Firmicutes | Thermoanaerobacterales | Caldanaerobacter | Caldanaerobacter subterraneus | 0.00027 |
| Firmicutes | Thermoanaerobacterales | Caldanaerobius | Caldanaerobius polysaccharolyticus | 0.00032 |
| Firmicutes | Thermoanaerobacterales | Caldicellulosiruptor | Caldicellulosiruptor kristjanssonii | 0.00004 |
| Firmicutes | Thermoanaerobacterales | Carboxydothermus | Carboxydothermus hydrogenoformans | 0.00001 |
| Firmicutes | Thermoanaerobacterales | Mahella | Mahella australiensis | 0.00025 |
| Firmicutes | Thermoanaerobacterales | Moorella | Moorella thermoacetica | 0.00002 |
| Firmicutes | Thermoanaerobacterales | Syntrophaceticus | Syntrophaceticus schinkii | 0.00061 |
| Firmicutes | Thermoanaerobacterales | Tepidanaerobacter | Tepidanaerobacter acetatoxydans | 0.00073 |
| Firmicutes | Thermoanaerobacterales | Thermacetogenium | Thermacetogenium phaeum | 0.00003 |
| Firmicutes | Thermoanaerobacterales | Thermoanaerobacter | Thermoanaerobacter italicus | 0.00003 |
| Firmicutes | Thermoanaerobacterales | Thermoanaerobacter | Thermoanaerobacter kivui | 0.00009 |
| Firmicutes | Thermoanaerobacterales | Thermoanaerobacter | Thermoanaerobacter mathranii | 0.00008 |
| Firmicutes | Thermoanaerobacterales | Thermoanaerobacter | Thermoanaerobacter pseudethanolicus | 0.00002 |
| Firmicutes | Thermoanaerobacterales | Thermoanaerobacter | Thermoanaerobacter siderophilus | 0.00023 |
| Firmicutes | Thermoanaerobacterales | Thermoanaerobacter | Thermoanaerobacter sp. A7A | 0.00004 |
| Firmicutes | Thermoanaerobacterales | Thermoanaerobacter | Thermoanaerobacter sp. X561 | 0.00002 |
| Firmicutes | Thermoanaerobacterales | Thermoanaerobacter | Thermoanaerobacter thermocopriae | 0.00004 |
| Firmicutes | Thermoanaerobacterales | Thermoanaerobacter | Thermoanaerobacter unclassified | 0.00024 |
| Firmicutes | Thermoanaerobacterales | Thermoanaerobacter | Thermoanaerobacter wiegelii | 0.00005 |
| Firmicutes | Thermoanaerobacterales | Thermoanaerobacterium | Thermoanaerobacterium saccharolyticum | 0.00003 |
| Firmicutes | Thermoanaerobacterales | Thermoanaerobacterium | Thermoanaerobacterium thermosaccharolyticum | 0.02752 |
| Firmicutes | Thermoanaerobacterales | Thermoanaerobacterium | Thermoanaerobacterium unclassified | 0.00076 |
| Firmicutes | Thermoanaerobacterales | Thermoanaerobacterium | Thermoanaerobacterium xylanolyticum | 0.00004 |
| Fusobacteria | Fusobacteriales | Cetobacterium | Cetobacterium somerae | 0.18426 |
| Fusobacteria | Fusobacteriales | Fusobacterium | Fusobacterium gonidiaformans | 0.08986 |
| Fusobacteria | Fusobacteriales | Fusobacterium | Fusobacterium hwasookii | 0.01044 |
| Fusobacteria | Fusobacteriales | Fusobacterium | Fusobacterium mortiferum | 1.06172 |
| Fusobacteria | Fusobacteriales | Fusobacterium | Fusobacterium necrophorum | 0.09474 |
| Fusobacteria | Fusobacteriales | Fusobacterium | Fusobacterium nucleatum | 0.13174 |
| Fusobacteria | Fusobacteriales | Fusobacterium | Fusobacterium perfoetens | 0.29215 |
| Fusobacteria | Fusobacteriales | Fusobacterium | Fusobacterium periodonticum | 0.03007 |
| Fusobacteria | Fusobacteriales | Fusobacterium | Fusobacterium russii | 0.08259 |
| Fusobacteria | Fusobacteriales | Fusobacterium | Fusobacterium sp. CAG:649 | 0.00632 |
| Fusobacteria | Fusobacteriales | Fusobacterium | Fusobacterium sp. CAG:815 | 0.00004 |
| Fusobacteria | Fusobacteriales | Fusobacterium | Fusobacterium sp. CM1 | 0.00049 |
| Fusobacteria | Fusobacteriales | Fusobacterium | Fusobacterium sp. CM21 | 0.00026 |
| Fusobacteria | Fusobacteriales | Fusobacterium | Fusobacterium sp. CM22 | 0.00682 |
| Fusobacteria | Fusobacteriales | Fusobacterium | Fusobacterium sp. OBRC1 | 0.01155 |
| Fusobacteria | Fusobacteriales | Fusobacterium | Fusobacterium sp. oral taxon 370 | 0.00046 |
| Fusobacteria | Fusobacteriales | Fusobacterium | Fusobacterium ulcerans | 3.00135 |
| Fusobacteria | Fusobacteriales | Fusobacterium | Fusobacterium unclassified | 0.17847 |
| Fusobacteria | Fusobacteriales | Fusobacterium | Fusobacterium varium | 2.89551 |
| Fusobacteria | Fusobacteriales | Ilyobacter | Ilyobacter polytropus | 0.01503 |
| Fusobacteria | Fusobacteriales | Leptotrichia | Leptotrichia buccalis | 0.00382 |
| Fusobacteria | Fusobacteriales | Leptotrichia | Leptotrichia goodfellowii | 0.00800 |
| Fusobacteria | Fusobacteriales | Leptotrichia | Leptotrichia hofstadii | 0.05093 |
| Fusobacteria | Fusobacteriales | Leptotrichia | Leptotrichia shahii | 0.00814 |
| Fusobacteria | Fusobacteriales | Leptotrichia | Leptotrichia sp. oral taxon 215 | 0.00180 |
| Fusobacteria | Fusobacteriales | Leptotrichia | Leptotrichia sp. oral taxon 225 | 0.00299 |
| Fusobacteria | Fusobacteriales | Leptotrichia | Leptotrichia sp. oral taxon 879 | 0.00012 |
| Fusobacteria | Fusobacteriales | Leptotrichia | Leptotrichia trevisanii | 0.00111 |
| Fusobacteria | Fusobacteriales | Leptotrichia | Leptotrichia wadei | 0.00875 |
| Fusobacteria | Fusobacteriales | Psychrilyobacter | Psychrilyobacter atlanticus | 0.00674 |
| Fusobacteria | Fusobacteriales | Sebaldella | Sebaldella termitidis | 0.00439 |
| Fusobacteria | Fusobacteriales | Sneathia | Sneathia sp. Sn35 | 0.00404 |
| Fusobacteria | Fusobacteriales | Streptobacillus | Streptobacillus moniliformis | 0.00056 |
| Nitrospirae | Nitrospirales | Candidatus Magnetobacterium | Candidatus Magnetobacterium bavaricum | 0.00007 |
| Nitrospirae | Nitrospirales | Leptospirillum | Leptospirillum sp. Group II 'C75' | 0.00002 |
| Parcubacteria | Parcubacteria noname | Parcubacteria noname | Parcubacteria bacterium RAAC4 OD1 1 | 0.00006 |
| Planctomycetes | Candidatus Brocadiales | Candidatus Kuenenia | Candidatus Kuenenia stuttgartiensis | 0.00004 |
| Proteobacteria | Acidithiobacillales | Acidithiobacillus | Acidithiobacillus caldus | 0.00017 |
| Proteobacteria | Acidithiobacillales | Acidithiobacillus | Acidithiobacillus thiooxidans | 0.00003 |
| Proteobacteria | Aeromonadales | Aeromonas | Aeromonas allosaccharophila | 0.00005 |
| Proteobacteria | Aeromonadales | Aeromonas | Aeromonas bestiarum | 0.00013 |
| Proteobacteria | Aeromonadales | Aeromonas | Aeromonas caviae | 0.00024 |
| Proteobacteria | Aeromonadales | Aeromonas | Aeromonas enteropelogenes | 0.00010 |
| Proteobacteria | Aeromonadales | Aeromonas | Aeromonas fluvialis | 0.00003 |
| Proteobacteria | Aeromonadales | Aeromonas | Aeromonas hydrophila | 0.00037 |
| Proteobacteria | Aeromonadales | Aeromonas | Aeromonas media | 0.00002 |
| Proteobacteria | Aeromonadales | Aeromonas | Aeromonas popoffii | 0.00004 |
| Proteobacteria | Aeromonadales | Aeromonas | Aeromonas salmonicida | 0.00011 |
| Proteobacteria | Aeromonadales | Aeromonas | Aeromonas sanarellii | 0.00007 |
| Proteobacteria | Aeromonadales | Aeromonas | Aeromonas simiae | 0.00002 |
| Proteobacteria | Aeromonadales | Aeromonas | Aeromonas sp. 159 | 0.00001 |
| Proteobacteria | Aeromonadales | Aeromonas | Aeromonas sp. 4287D | 0.00010 |
| Proteobacteria | Aeromonadales | Aeromonas | Aeromonas sp. C3 | 0.00817 |
| Proteobacteria | Aeromonadales | Aeromonas | Aeromonas unclassified | 0.00027 |
| Proteobacteria | Aeromonadales | Aeromonas | Aeromonas veronii | 0.00013 |
| Proteobacteria | Aeromonadales | Anaerobiospirillum | Anaerobiospirillum succiniciproducens | 0.00795 |
| Proteobacteria | Aeromonadales | Oceanimonas | Oceanimonas smirnovii | 0.00016 |
| Proteobacteria | Aeromonadales | Oceanimonas | Oceanimonas sp. GK1 | 0.00003 |
| Proteobacteria | Aeromonadales | Ruminobacter | Ruminobacter sp. RM87 | 0.00196 |
| Proteobacteria | Aeromonadales | Succinatimonas | Succinatimonas hippei | 0.01931 |
| Proteobacteria | Aeromonadales | Succinatimonas | Succinatimonas sp. CAG:777 | 0.05166 |
| Proteobacteria | Aeromonadales | Succinimonas | Succinimonas amylolytica | 0.00142 |
| Proteobacteria | Aeromonadales | Succinivibrio | Succinivibrio dextrinosolvens | 0.03507 |
| Proteobacteria | Aeromonadales | Succinivibrionaceae noname | Succinivibrionaceae bacterium WG-1 | 0.00021 |
| Proteobacteria | Aeromonadales | Succinivibrionaceae unclassified | Succinivibrionaceae unclassified | 0.00025 |
| Proteobacteria | Aeromonadales | Tolumonas | Tolumonas sp. BRL6-1 | 0.00005 |
| Proteobacteria | Alphaproteobacteria noname | Alphaproteobacteria noname | alpha proteobacterium MA2 | 0.00002 |
| Proteobacteria | Alphaproteobacteria noname | Alphaproteobacteria noname | alpha proteobacterium SCGC AAA158-B04 | 0.00002 |
| Proteobacteria | Alphaproteobacteria noname | Candidatus Pelagibacter | Candidatus Pelagibacter ubique | 0.00002 |
| Proteobacteria | Alphaproteobacteria noname | Candidatus Phaeomarinobacter | Candidatus Phaeomarinobacter ectocarpi | 0.00002 |
| Proteobacteria | Alphaproteobacteria noname | Thermopetrobacter | Thermopetrobacter sp. TC1 | 0.00002 |
| Proteobacteria | Alteromonadales | Algicola | Algicola sagamiensis | 0.00028 |
| Proteobacteria | Alteromonadales | Aliagarivorans | Aliagarivorans taiwanensis | 0.00001 |
| Proteobacteria | Alteromonadales | Alishewanella | Alishewanella aestuarii | 0.00002 |
| Proteobacteria | Alteromonadales | Alishewanella | Alishewanella unclassified | 0.00003 |
| Proteobacteria | Alteromonadales | Alteromonadales unclassified | Alteromonadales unclassified | 0.00001 |
| Proteobacteria | Alteromonadales | Alteromonas | Alteromonas macleodii | 0.00003 |
| Proteobacteria | Alteromonadales | Catenovulum | Catenovulum agarivorans | 0.00016 |
| Proteobacteria | Alteromonadales | Colwellia | Colwellia psychrerythraea | 0.00005 |
| Proteobacteria | Alteromonadales | Ferrimonas | Ferrimonas balearica | 0.00004 |
| Proteobacteria | Alteromonadales | Ferrimonas | Ferrimonas futtsuensis | 0.00010 |
| Proteobacteria | Alteromonadales | Ferrimonas | Ferrimonas kyonanensis | 0.00010 |
| Proteobacteria | Alteromonadales | Glaciecola | Glaciecola nitratireducens | 0.00001 |
| Proteobacteria | Alteromonadales | Haliea | Haliea salexigens | 0.00001 |
| Proteobacteria | Alteromonadales | Idiomarina | Idiomarina sp. MCCC 1A10513 | 0.00004 |
| Proteobacteria | Alteromonadales | Marinobacter | Marinobacter daepoensis | 0.00004 |
| Proteobacteria | Alteromonadales | Marinobacter | Marinobacter excellens | 0.00007 |
| Proteobacteria | Alteromonadales | Marinobacter | Marinobacter lipolyticus | 0.00004 |
| Proteobacteria | Alteromonadales | Marinobacter | Marinobacter nanhaiticus | 0.00002 |
| Proteobacteria | Alteromonadales | Marinobacter | Marinobacter similis | 0.00013 |
| Proteobacteria | Alteromonadales | Marinobacter | Marinobacter sp. AK21 | 0.00001 |
| Proteobacteria | Alteromonadales | Marinobacter | Marinobacter sp. ELB17 | 0.00003 |
| Proteobacteria | Alteromonadales | Marinobacterium | Marinobacterium litorale | 0.00001 |
| Proteobacteria | Alteromonadales | Marinobacterium | Marinobacterium sp. AK27 | 0.00006 |
| Proteobacteria | Alteromonadales | Marinobacterium | Marinobacterium stanieri | 0.00005 |
| Proteobacteria | Alteromonadales | Microbulbifer | Microbulbifer agarilyticus | 0.00002 |
| Proteobacteria | Alteromonadales | Moritella | Moritella marina | 0.00028 |
| Proteobacteria | Alteromonadales | Moritella | Moritella sp. PE36 | 0.00005 |
| Proteobacteria | Alteromonadales | Paraglaciecola | Paraglaciecola arctica | 0.00014 |
| Proteobacteria | Alteromonadales | Paraglaciecola | Paraglaciecola polaris | 0.00002 |
| Proteobacteria | Alteromonadales | Pseudoalteromonas | Pseudoalteromonas agarivorans | 0.00003 |
| Proteobacteria | Alteromonadales | Pseudoalteromonas | Pseudoalteromonas haloplanktis | 0.00015 |
| Proteobacteria | Alteromonadales | Pseudoalteromonas | Pseudoalteromonas luteoviolacea | 0.00002 |
| Proteobacteria | Alteromonadales | Pseudoalteromonas | Pseudoalteromonas rubra | 0.00005 |
| Proteobacteria | Alteromonadales | Pseudoalteromonas | Pseudoalteromonas sp. 643A | 0.00004 |
| Proteobacteria | Alteromonadales | Pseudoalteromonas | Pseudoalteromonas sp. BSi20311 | 0.00006 |
| Proteobacteria | Alteromonadales | Pseudoalteromonas | Pseudoalteromonas sp. BSi20429 | 0.00004 |
| Proteobacteria | Alteromonadales | Pseudoalteromonas | Pseudoalteromonas unclassified | 0.00005 |
| Proteobacteria | Alteromonadales | Psychromonas | Psychromonas arctica | 0.00031 |
| Proteobacteria | Alteromonadales | Psychromonas | Psychromonas sp. SP041 | 0.00005 |
| Proteobacteria | Alteromonadales | Shewanella | Shewanella algae | 0.00011 |
| Proteobacteria | Alteromonadales | Shewanella | Shewanella baltica | 0.00082 |
| Proteobacteria | Alteromonadales | Shewanella | Shewanella halifaxensis | 0.00009 |
| Proteobacteria | Alteromonadales | Shewanella | Shewanella haliotis | 0.00010 |
| Proteobacteria | Alteromonadales | Shewanella | Shewanella loihica | 0.00003 |
| Proteobacteria | Alteromonadales | Shewanella | Shewanella marina | 0.00014 |
| Proteobacteria | Alteromonadales | Shewanella | Shewanella pealeana | 0.00001 |
| Proteobacteria | Alteromonadales | Shewanella | Shewanella piezotolerans | 0.00011 |
| Proteobacteria | Alteromonadales | Shewanella | Shewanella sp. MR-4 | 0.00003 |
| Proteobacteria | Alteromonadales | Shewanella | Shewanella sp. POL2 | 0.00004 |
| Proteobacteria | Alteromonadales | Shewanella | Shewanella sp. W3-18-1 | 0.00001 |
| Proteobacteria | Alteromonadales | Shewanella | Shewanella sp. YQH10 | 0.00002 |
| Proteobacteria | Alteromonadales | Shewanella | Shewanella unclassified | 0.00002 |
| Proteobacteria | Alteromonadales | Shewanella | Shewanella waksmanii | 0.00002 |
| Proteobacteria | Alteromonadales | Spongiibacter | Spongiibacter marinus | 0.00001 |
| Proteobacteria | Alteromonadales | Teredinibacter | Teredinibacter turnerae | 0.00014 |
| Proteobacteria | Bdellovibrionales | Bacteriovorax | Bacteriovorax sp. BSW11 IV | 0.00002 |
| Proteobacteria | Betaproteobacteria noname | Betaproteobacteria noname | beta proteobacterium CB | 0.00002 |
| Proteobacteria | Betaproteobacteria noname | Betaproteobacteria noname | beta proteobacterium L13 | 0.00001 |
| Proteobacteria | Betaproteobacteria noname | Betaproteobacteria noname | beta proteobacterium SCGC AAA027-C02 | 0.00001 |
| Proteobacteria | Betaproteobacteria noname | Betaproteobacteria noname | beta proteobacterium SCGC AAA027-K21 | 0.00009 |
| Proteobacteria | Betaproteobacteria noname | Candidatus Accumulibacter | Candidatus Accumulibacter phosphatis | 0.00001 |
| Proteobacteria | Betaproteobacteria noname | Candidatus Accumulibacter | Candidatus Accumulibacter sp. SK-01 | 0.00002 |
| Proteobacteria | Betaproteobacteria noname | Candidatus Accumulibacter | Candidatus Accumulibacter sp. SK-11 | 0.00001 |
| Proteobacteria | Burkholderiales | Achromobacter | Achromobacter arsenitoxydans | 0.00008 |
| Proteobacteria | Burkholderiales | Achromobacter | Achromobacter insuavis | 0.00012 |
| Proteobacteria | Burkholderiales | Achromobacter | Achromobacter piechaudii | 0.00020 |
| Proteobacteria | Burkholderiales | Achromobacter | Achromobacter sp. DH1f | 0.00010 |
| Proteobacteria | Burkholderiales | Achromobacter | Achromobacter sp. RTa | 0.00012 |
| Proteobacteria | Burkholderiales | Achromobacter | Achromobacter xylosoxidans | 0.00038 |
| Proteobacteria | Burkholderiales | Acidovorax | Acidovorax citrulli | 0.00013 |
| Proteobacteria | Burkholderiales | Acidovorax | Acidovorax delafieldii | 0.00023 |
| Proteobacteria | Burkholderiales | Acidovorax | Acidovorax oryzae | 0.00004 |
| Proteobacteria | Burkholderiales | Acidovorax | Acidovorax sp. CF316 | 0.00002 |
| Proteobacteria | Burkholderiales | Acidovorax | Acidovorax sp. JHL-9 | 0.00001 |
| Proteobacteria | Burkholderiales | Acidovorax | Acidovorax sp. JS42 | 0.00002 |
| Proteobacteria | Burkholderiales | Acidovorax | Acidovorax sp. KKS102 | 0.00011 |
| Proteobacteria | Burkholderiales | Acidovorax | Acidovorax sp. MR-S7 | 0.00008 |
| Proteobacteria | Burkholderiales | Acidovorax | Acidovorax sp. NO-1 | 0.00002 |
| Proteobacteria | Burkholderiales | Acidovorax | Acidovorax temperans | 0.00001 |
| Proteobacteria | Burkholderiales | Advenella | Advenella kashmirensis | 0.00025 |
| Proteobacteria | Burkholderiales | Advenella | Advenella mimigardefordensis | 0.00012 |
| Proteobacteria | Burkholderiales | Advenella | Advenella unclassified | 0.00003 |
| Proteobacteria | Burkholderiales | Alcaligenaceae unclassified | Alcaligenaceae unclassified | 0.00005 |
| Proteobacteria | Burkholderiales | Alcaligenes | Alcaligenes faecalis | 0.00606 |
| Proteobacteria | Burkholderiales | Alcaligenes | Alcaligenes sp. EGD-AK7 | 0.00033 |
| Proteobacteria | Burkholderiales | Alcaligenes | Alcaligenes sp. HPC1271 | 0.00019 |
| Proteobacteria | Burkholderiales | Alcaligenes | Alcaligenes unclassified | 0.00165 |
| Proteobacteria | Burkholderiales | Alicycliphilus | Alicycliphilus denitrificans | 0.00037 |
| Proteobacteria | Burkholderiales | Alicycliphilus | Alicycliphilus sp. CRZ1 | 0.00002 |
| Proteobacteria | Burkholderiales | Aquincola | Aquincola tertiaricarbonis | 0.00002 |
| Proteobacteria | Burkholderiales | Basilea | Basilea psittacipulmonis | 0.00003 |
| Proteobacteria | Burkholderiales | Bordetella | Bordetella avium | 0.00034 |
| Proteobacteria | Burkholderiales | Bordetella | Bordetella bronchiseptica | 0.00041 |
| Proteobacteria | Burkholderiales | Bordetella | Bordetella hinzii | 0.00015 |
| Proteobacteria | Burkholderiales | Bordetella | Bordetella holmesii | 0.00007 |
| Proteobacteria | Burkholderiales | Bordetella | Bordetella parapertussis | 0.00003 |
| Proteobacteria | Burkholderiales | Bordetella | Bordetella pertussis | 0.00005 |
| Proteobacteria | Burkholderiales | Bordetella | Bordetella petrii | 0.00057 |
| Proteobacteria | Burkholderiales | Bordetella | Bordetella sp. FB-8 | 0.00005 |
| Proteobacteria | Burkholderiales | Bordetella | Bordetella trematum | 0.00026 |
| Proteobacteria | Burkholderiales | Bordetella | Bordetella unclassified | 0.00001 |
| Proteobacteria | Burkholderiales | Brackiella | Brackiella oedipodis | 0.00005 |
| Proteobacteria | Burkholderiales | Burkholderia | Burkholderia ambifaria | 0.00001 |
| Proteobacteria | Burkholderiales | Burkholderia | Burkholderia bannensis | 0.00012 |
| Proteobacteria | Burkholderiales | Burkholderia | Burkholderia cepacia | 0.00003 |
| Proteobacteria | Burkholderiales | Burkholderia | Burkholderia dolosa | 0.00002 |
| Proteobacteria | Burkholderiales | Burkholderia | Burkholderia fungorum | 0.00067 |
| Proteobacteria | Burkholderiales | Burkholderia | Burkholderia multivorans | 0.15266 |
| Proteobacteria | Burkholderiales | Burkholderia | Burkholderia oklahomensis | 0.00003 |
| Proteobacteria | Burkholderiales | Burkholderia | Burkholderia phymatum | 0.00004 |
| Proteobacteria | Burkholderiales | Burkholderia | Burkholderia phytofirmans | 0.00002 |
| Proteobacteria | Burkholderiales | Burkholderia | Burkholderia pseudomallei | 0.00046 |
| Proteobacteria | Burkholderiales | Burkholderia | Burkholderia rhizoxinica | 0.00006 |
| Proteobacteria | Burkholderiales | Burkholderia | Burkholderia sacchari | 0.00001 |
| Proteobacteria | Burkholderiales | Burkholderia | Burkholderia sp. BT03 | 0.00002 |
| Proteobacteria | Burkholderiales | Burkholderia | Burkholderia sp. CCGE1003 | 0.00001 |
| Proteobacteria | Burkholderiales | Burkholderia | Burkholderia sp. JPY347 | 0.00001 |
| Proteobacteria | Burkholderiales | Burkholderia | Burkholderia sp. MR1 | 0.00003 |
| Proteobacteria | Burkholderiales | Burkholderia | Burkholderia sp. MSHR3999 | 0.00001 |
| Proteobacteria | Burkholderiales | Burkholderia | Burkholderia sp. RPE64 | 0.00003 |
| Proteobacteria | Burkholderiales | Burkholderia | Burkholderia sp. WP42 | 0.00002 |
| Proteobacteria | Burkholderiales | Burkholderia | Burkholderia terrae | 0.00004 |
| Proteobacteria | Burkholderiales | Burkholderia | Burkholderia thailandensis | 0.00003 |
| Proteobacteria | Burkholderiales | Burkholderia | Burkholderia ubonensis | 0.00002 |
| Proteobacteria | Burkholderiales | Burkholderia | Burkholderia unclassified | 0.00007 |
| Proteobacteria | Burkholderiales | Burkholderia | Burkholderia vietnamiensis | 0.00008 |
| Proteobacteria | Burkholderiales | Burkholderia | Burkholderia xenovorans | 0.00000 |
| Proteobacteria | Burkholderiales | Burkholderiaceae noname | bacterium endosymbiont of Mortierella elongata FMR23-6 | 0.00003 |
| Proteobacteria | Burkholderiales | Burkholderiaceae noname | Burkholderiaceae bacterium 26 | 0.00002 |
| Proteobacteria | Burkholderiales | Burkholderiales noname | Burkholderiales bacterium 1 1 47 | 0.00225 |
| Proteobacteria | Burkholderiales | Castellaniella | Castellaniella defragrans | 0.00060 |
| Proteobacteria | Burkholderiales | Chitinimonas | Chitinimonas koreensis | 0.00014 |
| Proteobacteria | Burkholderiales | Collimonas | Collimonas arenae | 0.00012 |
| Proteobacteria | Burkholderiales | Collimonas | Collimonas fungivorans | 0.00004 |
| Proteobacteria | Burkholderiales | Comamonadaceae noname | Comamonadaceae bacterium A1 | 0.00001 |
| Proteobacteria | Burkholderiales | Comamonas | Comamonas badia | 0.00003 |
| Proteobacteria | Burkholderiales | Comamonas | Comamonas composti | 0.00003 |
| Proteobacteria | Burkholderiales | Comamonas | Comamonas sp. B-9 | 0.00002 |
| Proteobacteria | Burkholderiales | Comamonas | Comamonas sp. DJ-12 | 0.00004 |
| Proteobacteria | Burkholderiales | Comamonas | Comamonas testosteroni | 0.00030 |
| Proteobacteria | Burkholderiales | Cupriavidus | Cupriavidus basilensis | 0.00009 |
| Proteobacteria | Burkholderiales | Cupriavidus | Cupriavidus metallidurans | 0.00004 |
| Proteobacteria | Burkholderiales | Cupriavidus | Cupriavidus necator | 0.00005 |
| Proteobacteria | Burkholderiales | Cupriavidus | Cupriavidus pinatubonensis | 0.00005 |
| Proteobacteria | Burkholderiales | Cupriavidus | Cupriavidus sp. amp6 | 0.00001 |
| Proteobacteria | Burkholderiales | Cupriavidus | Cupriavidus sp. HPC(L) | 0.00003 |
| Proteobacteria | Burkholderiales | Cupriavidus | Cupriavidus sp. JGI 0001016-D21 | 0.00004 |
| Proteobacteria | Burkholderiales | Cupriavidus | Cupriavidus sp. SK-3 | 0.00002 |
| Proteobacteria | Burkholderiales | Cupriavidus | Cupriavidus sp. SK-4 | 0.00001 |
| Proteobacteria | Burkholderiales | Cupriavidus | Cupriavidus sp. UYPR2.512 | 0.00001 |
| Proteobacteria | Burkholderiales | Curvibacter | Curvibacter gracilis | 0.00003 |
| Proteobacteria | Burkholderiales | Curvibacter | Curvibacter lanceolatus | 0.00006 |
| Proteobacteria | Burkholderiales | Delftia | Delftia acidovorans | 0.00020 |
| Proteobacteria | Burkholderiales | Delftia | Delftia sp. Cs1-4 | 0.00004 |
| Proteobacteria | Burkholderiales | Delftia | Delftia sp. RIT313 | 0.00003 |
| Proteobacteria | Burkholderiales | Delftia | Delftia unclassified | 0.00002 |
| Proteobacteria | Burkholderiales | Duganella | Duganella zoogloeoides | 0.00001 |
| Proteobacteria | Burkholderiales | Herbaspirillum | Herbaspirillum frisingense | 0.00002 |
| Proteobacteria | Burkholderiales | Herbaspirillum | Herbaspirillum massiliense | 0.00003 |
| Proteobacteria | Burkholderiales | Herbaspirillum | Herbaspirillum rubrisubalbicans | 0.00088 |
| Proteobacteria | Burkholderiales | Herbaspirillum | Herbaspirillum sp. CF444 | 0.00015 |
| Proteobacteria | Burkholderiales | Herbaspirillum | Herbaspirillum sp. RV1423 | 0.00001 |
| Proteobacteria | Burkholderiales | Herbaspirillum | Herbaspirillum sp. TSA66 | 0.00005 |
| Proteobacteria | Burkholderiales | Herminiimonas | Herminiimonas sp. CN | 0.00007 |
| Proteobacteria | Burkholderiales | Ideonella | Ideonella sp. B508-1 | 0.00007 |
| Proteobacteria | Burkholderiales | Janthinobacterium | Janthinobacterium agaricidamnosum | 0.00018 |
| Proteobacteria | Burkholderiales | Janthinobacterium | Janthinobacterium lividum | 0.00002 |
| Proteobacteria | Burkholderiales | Janthinobacterium | Janthinobacterium sp. HH01 | 0.00002 |
| Proteobacteria | Burkholderiales | Janthinobacterium | Janthinobacterium sp. Marseille | 0.00001 |
| Proteobacteria | Burkholderiales | Janthinobacterium | Janthinobacterium unclassified | 0.00002 |
| Proteobacteria | Burkholderiales | Lautropia | Lautropia mirabilis | 0.00007 |
| Proteobacteria | Burkholderiales | Limnobacter | Limnobacter sp. MED105 | 0.00002 |
| Proteobacteria | Burkholderiales | Limnohabitans | Limnohabitans sp. Rim47 | 0.00001 |
| Proteobacteria | Burkholderiales | Massilia | Massilia alkalitolerans | 0.00025 |
| Proteobacteria | Burkholderiales | Massilia | Massilia niastensis | 0.00052 |
| Proteobacteria | Burkholderiales | Massilia | Massilia sp. 9096 | 0.00017 |
| Proteobacteria | Burkholderiales | Massilia | Massilia sp. BSC265 | 0.00028 |
| Proteobacteria | Burkholderiales | Massilia | Massilia sp. JS1662 | 0.00017 |
| Proteobacteria | Burkholderiales | Massilia | Massilia sp. LC238 | 0.00026 |
| Proteobacteria | Burkholderiales | Massilia | Massilia timonae | 0.00036 |
| Proteobacteria | Burkholderiales | Massilia | Massilia unclassified | 0.00003 |
| Proteobacteria | Burkholderiales | Methylibium | Methylibium petroleiphilum | 0.00001 |
| Proteobacteria | Burkholderiales | Oligella | Oligella ureolytica | 0.00054 |
| Proteobacteria | Burkholderiales | Oligella | Oligella urethralis | 0.00046 |
| Proteobacteria | Burkholderiales | Ottowia | Ottowia thiooxydans | 0.00011 |
| Proteobacteria | Burkholderiales | Oxalobacter | Oxalobacter formigenes | 0.00021 |
| Proteobacteria | Burkholderiales | Oxalobacteraceae noname | Oxalobacteraceae bacterium AB 14 | 0.00003 |
| Proteobacteria | Burkholderiales | Oxalobacteraceae noname | Oxalobacteraceae bacterium JGI 0001004-K23 | 0.00004 |
| Proteobacteria | Burkholderiales | Oxalobacteraceae noname | Oxalobacteraceae bacterium JGI 001010-B17 | 0.00001 |
| Proteobacteria | Burkholderiales | Pandoraea | Pandoraea apista | 0.00005 |
| Proteobacteria | Burkholderiales | Pandoraea | Pandoraea pnomenusa | 0.00002 |
| Proteobacteria | Burkholderiales | Pandoraea | Pandoraea sp. B-6 | 0.00007 |
| Proteobacteria | Burkholderiales | Pandoraea | Pandoraea sputorum | 0.00003 |
| Proteobacteria | Burkholderiales | Parasutterella | Parasutterella excrementihominis | 0.00277 |
| Proteobacteria | Burkholderiales | Parasutterella | Parasutterella excrementihominis CAG:233 | 0.00063 |
| Proteobacteria | Burkholderiales | Parasutterella | Parasutterella unclassified | 0.00059 |
| Proteobacteria | Burkholderiales | Pelistega | Pelistega sp. HM-7 | 0.00187 |
| Proteobacteria | Burkholderiales | Polaromonas | Polaromonas naphthalenivorans | 0.00005 |
| Proteobacteria | Burkholderiales | Polaromonas | Polaromonas sp. CF318 | 0.00006 |
| Proteobacteria | Burkholderiales | Pseudacidovorax | Pseudacidovorax intermedius | 0.00004 |
| Proteobacteria | Burkholderiales | Pseudoduganella | Pseudoduganella violaceinigra | 0.00004 |
| Proteobacteria | Burkholderiales | Pusillimonas | Pusillimonas noertemannii | 0.00133 |
| Proteobacteria | Burkholderiales | Pusillimonas | Pusillimonas sp. T7-7 | 0.00237 |
| Proteobacteria | Burkholderiales | Ralstonia | Ralstonia pickettii | 0.00016 |
| Proteobacteria | Burkholderiales | Ralstonia | Ralstonia solanacearum | 0.00002 |
| Proteobacteria | Burkholderiales | Ralstonia | Ralstonia sp. A12 | 0.00001 |
| Proteobacteria | Burkholderiales | Ralstonia | Ralstonia sp. AU12-08 | 0.00001 |
| Proteobacteria | Burkholderiales | Rubrivivax | Rubrivivax benzoatilyticus | 0.00001 |
| Proteobacteria | Burkholderiales | Simplicispira | Simplicispira psychrophila | 0.00001 |
| Proteobacteria | Burkholderiales | Sutterella | Sutterella parvirubra | 0.00403 |
| Proteobacteria | Burkholderiales | Sutterella | Sutterella sp. CAG:351 | 0.00201 |
| Proteobacteria | Burkholderiales | Sutterella | Sutterella sp. CAG:397 | 0.00349 |
| Proteobacteria | Burkholderiales | Sutterella | Sutterella sp. CAG:521 | 0.00155 |
| Proteobacteria | Burkholderiales | Sutterella | Sutterella unclassified | 0.00184 |
| Proteobacteria | Burkholderiales | Sutterella | Sutterella wadsworthensis | 0.25027 |
| Proteobacteria | Burkholderiales | Sutterella | Sutterella wadsworthensis CAG:135 | 0.00384 |
| Proteobacteria | Burkholderiales | Taylorella | Taylorella asinigenitalis | 0.00005 |
| Proteobacteria | Burkholderiales | Taylorella | Taylorella equigenitalis | 0.00005 |
| Proteobacteria | Burkholderiales | Taylorella | Taylorella unclassified | 0.00003 |
| Proteobacteria | Burkholderiales | Tepidimonas | Tepidimonas taiwanensis | 0.00001 |
| Proteobacteria | Burkholderiales | Thiomonas | Thiomonas sp. FB-6 | 0.00002 |
| Proteobacteria | Burkholderiales | Variovorax | Variovorax paradoxus | 0.00010 |
| Proteobacteria | Burkholderiales | Variovorax | Variovorax sp. JGI 0001013-L10 | 0.00013 |
| Proteobacteria | Burkholderiales | Variovorax | Variovorax unclassified | 0.00001 |
| Proteobacteria | Burkholderiales | Verminephrobacter | Verminephrobacter eiseniae | 0.00001 |
| Proteobacteria | Burkholderiales | Xenophilus | Xenophilus azovorans | 0.00002 |
| Proteobacteria | Campylobacterales | Arcobacter | Arcobacter butzleri | 0.00004 |
| Proteobacteria | Campylobacterales | Arcobacter | Arcobacter sp. L | 0.00002 |
| Proteobacteria | Campylobacterales | Campylobacter | Campylobacter coli | 0.01339 |
| Proteobacteria | Campylobacterales | Campylobacter | Campylobacter concisus | 0.00006 |
| Proteobacteria | Campylobacterales | Campylobacter | Campylobacter fetus | 0.00022 |
| Proteobacteria | Campylobacterales | Campylobacter | Campylobacter gracilis | 0.00171 |
| Proteobacteria | Campylobacterales | Campylobacter | Campylobacter insulaenigrae | 0.00005 |
| Proteobacteria | Campylobacterales | Campylobacter | Campylobacter jejuni | 0.00522 |
| Proteobacteria | Campylobacterales | Campylobacter | Campylobacter peloridis | 0.00006 |
| Proteobacteria | Campylobacterales | Campylobacter | Campylobacter rectus | 0.00001 |
| Proteobacteria | Campylobacterales | Campylobacter | Campylobacter showae | 0.00012 |
| Proteobacteria | Campylobacterales | Campylobacter | Campylobacter sp. FOBRC14 | 0.00003 |
| Proteobacteria | Campylobacterales | Campylobacter | Campylobacter unclassified | 0.00028 |
| Proteobacteria | Campylobacterales | Campylobacter | Campylobacter ureolyticus | 0.00005 |
| Proteobacteria | Campylobacterales | Helicobacter | Helicobacter apodemus | 0.00003 |
| Proteobacteria | Campylobacterales | Helicobacter | Helicobacter bilis | 0.00006 |
| Proteobacteria | Campylobacterales | Helicobacter | Helicobacter canis | 0.00001 |
| Proteobacteria | Campylobacterales | Helicobacter | Helicobacter cinaedi | 0.00004 |
| Proteobacteria | Campylobacterales | Helicobacter | Helicobacter mustelae | 0.00002 |
| Proteobacteria | Campylobacterales | Helicobacter | Helicobacter pylori | 0.00071 |
| Proteobacteria | Campylobacterales | Helicobacter | Helicobacter sanguini | 0.00002 |
| Proteobacteria | Campylobacterales | Helicobacter | Helicobacter trogontum | 0.00002 |
| Proteobacteria | Campylobacterales | Sulfuricurvum | Candidatus Sulfuricurvum sp. RIFRC-1 | 0.00002 |
| Proteobacteria | Campylobacterales | Sulfuricurvum | Sulfuricurvum sp. MLSB | 0.00003 |
| Proteobacteria | Campylobacterales | Sulfurospirillum | Sulfurospirillum arsenophilum | 0.00001 |
| Proteobacteria | Campylobacterales | Sulfurospirillum | Sulfurospirillum barnesii | 0.00007 |
| Proteobacteria | Campylobacterales | Sulfurospirillum | Sulfurospirillum deleyianum | 0.00002 |
| Proteobacteria | Campylobacterales | Sulfurospirillum | Sulfurospirillum sp. SCADC | 0.00003 |
| Proteobacteria | Campylobacterales | Wolinella | Wolinella succinogenes | 0.00002 |
| Proteobacteria | Cardiobacteriales | Cardiobacterium | Cardiobacterium hominis | 0.00004 |
| Proteobacteria | Cardiobacteriales | Cardiobacterium | Cardiobacterium valvarum | 0.00017 |
| Proteobacteria | Cardiobacteriales | Dichelobacter | Dichelobacter nodosus | 0.00042 |
| Proteobacteria | Caulobacterales | Asticcacaulis | Asticcacaulis sp. AC466 | 0.00002 |
| Proteobacteria | Caulobacterales | Brevundimonas | Brevundimonas diminuta | 0.00001 |
| Proteobacteria | Caulobacterales | Brevundimonas | Brevundimonas nasdae | 0.00002 |
| Proteobacteria | Chromatiales | Allochromatium | Allochromatium vinosum | 0.00001 |
| Proteobacteria | Chromatiales | Ectothiorhodospira | Ectothiorhodospira haloalkaliphila | 0.00014 |
| Proteobacteria | Chromatiales | Ectothiorhodospira | Ectothiorhodospira sp. PHS-1 | 0.00005 |
| Proteobacteria | Chromatiales | Nitrosococcus | Nitrosococcus oceani | 0.00001 |
| Proteobacteria | Chromatiales | Rheinheimera | Rheinheimera baltica | 0.00001 |
| Proteobacteria | Chromatiales | Rheinheimera | Rheinheimera perlucida | 0.00002 |
| Proteobacteria | Chromatiales | Thioalkalivibrio | Thioalkalivibrio sp. ALJ17 | 0.00001 |
| Proteobacteria | Chromatiales | Thioalkalivibrio | Thioalkalivibrio sp. ALMg11 | 0.00002 |
| Proteobacteria | Chromatiales | Thioalkalivibrio | Thioalkalivibrio sp. ALSr1 | 0.00001 |
| Proteobacteria | Chromatiales | Thioalkalivibrio | Thioalkalivibrio sp. HK1 | 0.00005 |
| Proteobacteria | Chromatiales | Thioalkalivibrio | Thioalkalivibrio thiocyanoxidans | 0.00001 |
| Proteobacteria | Chromatiales | Thiocapsa | Thiocapsa marina | 0.00005 |
| Proteobacteria | Chromatiales | Thiorhodospira | Thiorhodospira sibirica | 0.00003 |
| Proteobacteria | Chromatiales | Thiorhodovibrio | Thiorhodovibrio sp. 970 | 0.00003 |
| Proteobacteria | Deltaproteobacteria noname | Candidatus Entotheonella | Candidatus Entotheonella sp. TSY2 | 0.00001 |
| Proteobacteria | Deltaproteobacteria unclassified | Deltaproteobacteria unclassified | Deltaproteobacteria unclassified | 0.00002 |
| Proteobacteria | Desulfobacterales | Candidatus Magnetoglobus | Candidatus Magnetoglobus multicellularis | 0.00007 |
| Proteobacteria | Desulfobacterales | Desulfatibacillum | Desulfatibacillum aliphaticivorans | 0.00012 |
| Proteobacteria | Desulfobacterales | Desulfobacter | Desulfobacter postgatei | 0.00002 |
| Proteobacteria | Desulfobacterales | Desulfobacula | Desulfobacula sp. TS | 0.00001 |
| Proteobacteria | Desulfobacterales | Desulfobulbus | Desulfobulbus japonicus | 0.00012 |
| Proteobacteria | Desulfobacterales | Desulfobulbus | Desulfobulbus sp. Tol-SR | 0.00006 |
| Proteobacteria | Desulfobacterales | Desulfococcus | Desulfococcus oleovorans | 0.00004 |
| Proteobacteria | Desulfovibrionales | Bilophila | Bilophila sp. 4 1 30 | 0.00081 |
| Proteobacteria | Desulfovibrionales | Bilophila | Bilophila wadsworthia | 0.00024 |
| Proteobacteria | Desulfovibrionales | Desulfomicrobium | Desulfomicrobium baculatum | 0.00016 |
| Proteobacteria | Desulfovibrionales | Desulfonatronovibrio | Desulfonatronovibrio hydrogenovorans | 0.00003 |
| Proteobacteria | Desulfovibrionales | Desulfovibrio | Desulfovibrio africanus | 0.00005 |
| Proteobacteria | Desulfovibrionales | Desulfovibrio | Desulfovibrio alaskensis | 0.00001 |
| Proteobacteria | Desulfovibrionales | Desulfovibrio | Desulfovibrio aminophilus | 0.00003 |
| Proteobacteria | Desulfovibrionales | Desulfovibrio | Desulfovibrio bastinii | 0.00007 |
| Proteobacteria | Desulfovibrionales | Desulfovibrio | Desulfovibrio desulfuricans | 0.00009 |
| Proteobacteria | Desulfovibrionales | Desulfovibrio | Desulfovibrio piger | 0.00044 |
| Proteobacteria | Desulfovibrionales | Desulfovibrio | Desulfovibrio salexigens | 0.00002 |
| Proteobacteria | Desulfovibrionales | Desulfovibrio | Desulfovibrio sp. 3 1 syn3 | 0.00002 |
| Proteobacteria | Desulfovibrionales | Desulfovibrio | Desulfovibrio sp. 6 1 46AFAA | 0.00010 |
| Proteobacteria | Desulfovibrionales | Desulfovibrio | Desulfovibrio sp. A2 | 0.00003 |
| Proteobacteria | Desulfovibrionales | Desulfovibrio | Desulfovibrio sp. Dsv1 | 0.00005 |
| Proteobacteria | Desulfovibrionales | Desulfovibrio | Desulfovibrio sp. U5L | 0.00002 |
| Proteobacteria | Desulfovibrionales | Desulfovibrio | Desulfovibrio unclassified | 0.00008 |
| Proteobacteria | Desulfovibrionales | Desulfovibrio | Desulfovibrio vulgaris | 0.00003 |
| Proteobacteria | Desulfurellales | Hippea | Hippea maritima | 0.00004 |
| Proteobacteria | Desulfuromonadales | Geobacter | Geobacter lovleyi | 0.00006 |
| Proteobacteria | Desulfuromonadales | Geobacter | Geobacter sp. GSS01 | 0.00007 |
| Proteobacteria | Desulfuromonadales | Geobacter | Geobacter sulfurreducens | 0.00001 |
| Proteobacteria | Enterobacteriales | Arsenophonus | Arsenophonus nasoniae | 0.00005 |
| Proteobacteria | Enterobacteriales | Brenneria | Brenneria sp. EniD312 | 0.00002 |
| Proteobacteria | Enterobacteriales | Budvicia | Budvicia aquatica | 0.00007 |
| Proteobacteria | Enterobacteriales | Buttiauxella | Buttiauxella agrestis | 0.00050 |
| Proteobacteria | Enterobacteriales | Candidatus Hamiltonella | Candidatus Hamiltonella defensa | 0.00038 |
| Proteobacteria | Enterobacteriales | Cedecea | Cedecea davisae | 0.00008 |
| Proteobacteria | Enterobacteriales | Cedecea | Cedecea neteri | 0.00045 |
| Proteobacteria | Enterobacteriales | Citrobacter | Citrobacter amalonaticus | 0.00252 |
| Proteobacteria | Enterobacteriales | Citrobacter | Citrobacter braakii | 0.00020 |
| Proteobacteria | Enterobacteriales | Citrobacter | Citrobacter freundii | 0.00203 |
| Proteobacteria | Enterobacteriales | Citrobacter | Citrobacter koseri | 0.00426 |
| Proteobacteria | Enterobacteriales | Citrobacter | Citrobacter rodentium | 0.00040 |
| Proteobacteria | Enterobacteriales | Citrobacter | Citrobacter sedlakii | 0.00004 |
| Proteobacteria | Enterobacteriales | Citrobacter | Citrobacter sp. 30 2 | 0.00011 |
| Proteobacteria | Enterobacteriales | Citrobacter | Citrobacter sp. CIP 55.13 | 0.00012 |
| Proteobacteria | Enterobacteriales | Citrobacter | Citrobacter sp. KTE151 | 0.00006 |
| Proteobacteria | Enterobacteriales | Citrobacter | Citrobacter sp. KTE32 | 0.00007 |
| Proteobacteria | Enterobacteriales | Citrobacter | Citrobacter sp. MGH 55 | 0.00003 |
| Proteobacteria | Enterobacteriales | Citrobacter | Citrobacter sp. S-77 | 0.00067 |
| Proteobacteria | Enterobacteriales | Citrobacter | Citrobacter unclassified | 0.00003 |
| Proteobacteria | Enterobacteriales | Citrobacter | Citrobacter werkmanii | 0.00002 |
| Proteobacteria | Enterobacteriales | Citrobacter | Citrobacter youngae | 0.00004 |
| Proteobacteria | Enterobacteriales | Cronobacter | Cronobacter condimenti | 0.00006 |
| Proteobacteria | Enterobacteriales | Cronobacter | Cronobacter malonaticus | 0.00022 |
| Proteobacteria | Enterobacteriales | Cronobacter | Cronobacter sakazakii | 0.00133 |
| Proteobacteria | Enterobacteriales | Cronobacter | Cronobacter sp. 1383 | 0.00003 |
| Proteobacteria | Enterobacteriales | Cronobacter | Cronobacter unclassified | 0.00026 |
| Proteobacteria | Enterobacteriales | Cronobacter | Cronobacter universalis | 0.01111 |
| Proteobacteria | Enterobacteriales | Dickeya | Dickeya chrysanthemi | 0.00005 |
| Proteobacteria | Enterobacteriales | Dickeya | Dickeya dadantii | 0.00002 |
| Proteobacteria | Enterobacteriales | Dickeya | Dickeya dianthicola | 0.00002 |
| Proteobacteria | Enterobacteriales | Dickeya | Dickeya solani | 0.00003 |
| Proteobacteria | Enterobacteriales | Dickeya | Dickeya sp. 2B12 | 0.00003 |
| Proteobacteria | Enterobacteriales | Dickeya | Dickeya sp. DW 0440 | 0.00011 |
| Proteobacteria | Enterobacteriales | Dickeya | Dickeya unclassified | 0.00006 |
| Proteobacteria | Enterobacteriales | Dickeya | Dickeya zeae | 0.00125 |
| Proteobacteria | Enterobacteriales | Edwardsiella | Edwardsiella hoshinae | 0.00059 |
| Proteobacteria | Enterobacteriales | Edwardsiella | Edwardsiella ictaluri | 0.00081 |
| Proteobacteria | Enterobacteriales | Edwardsiella | Edwardsiella piscicida | 0.01043 |
| Proteobacteria | Enterobacteriales | Edwardsiella | Edwardsiella tarda | 0.00262 |
| Proteobacteria | Enterobacteriales | Edwardsiella | Edwardsiella unclassified | 0.00131 |
| Proteobacteria | Enterobacteriales | Enterobacter | Enterobacter aerogenes | 0.00491 |
| Proteobacteria | Enterobacteriales | Enterobacter | Enterobacter asburiae | 0.00064 |
| Proteobacteria | Enterobacteriales | Enterobacter | Enterobacter cloacae | 0.01409 |
| Proteobacteria | Enterobacteriales | Enterobacter | Enterobacter hormaechei | 0.00304 |
| Proteobacteria | Enterobacteriales | Enterobacter | Enterobacter ludwigii | 0.00002 |
| Proteobacteria | Enterobacteriales | Enterobacter | Enterobacter massiliensis | 0.00042 |
| Proteobacteria | Enterobacteriales | Enterobacter | Enterobacter sp. 35666 | 0.00003 |
| Proteobacteria | Enterobacteriales | Enterobacter | Enterobacter sp. 35669 | 0.00001 |
| Proteobacteria | Enterobacteriales | Enterobacter | Enterobacter sp. 35683 | 0.00001 |
| Proteobacteria | Enterobacteriales | Enterobacter | Enterobacter sp. 35699 | 0.00005 |
| Proteobacteria | Enterobacteriales | Enterobacter | Enterobacter sp. 35730 | 0.00168 |
| Proteobacteria | Enterobacteriales | Enterobacter | Enterobacter sp. 42324 | 0.00010 |
| Proteobacteria | Enterobacteriales | Enterobacter | Enterobacter sp. 44593 | 0.00023 |
| Proteobacteria | Enterobacteriales | Enterobacter | Enterobacter sp. 5-4 | 0.00013 |
| Proteobacteria | Enterobacteriales | Enterobacter | Enterobacter sp. 638 | 0.00003 |
| Proteobacteria | Enterobacteriales | Enterobacter | Enterobacter sp. B509 | 0.00047 |
| Proteobacteria | Enterobacteriales | Enterobacter | Enterobacter sp. BIDMC 30 | 0.00001 |
| Proteobacteria | Enterobacteriales | Enterobacter | Enterobacter sp. Bisph1 | 0.00001 |
| Proteobacteria | Enterobacteriales | Enterobacter | Enterobacter sp. DC1 | 0.00034 |
| Proteobacteria | Enterobacteriales | Enterobacter | Enterobacter sp. E20 | 0.00008 |
| Proteobacteria | Enterobacteriales | Enterobacter | Enterobacter sp. EGD-HP1 | 0.01094 |
| Proteobacteria | Enterobacteriales | Enterobacter | Enterobacter sp. FB | 0.00019 |
| Proteobacteria | Enterobacteriales | Enterobacter | Enterobacter sp. MGH 16 | 0.00008 |
| Proteobacteria | Enterobacteriales | Enterobacter | Enterobacter sp. MGH 23 | 0.00012 |
| Proteobacteria | Enterobacteriales | Enterobacter | Enterobacter sp. MGH 33 | 0.00006 |
| Proteobacteria | Enterobacteriales | Enterobacter | Enterobacter sp. MGH 34 | 0.00026 |
| Proteobacteria | Enterobacteriales | Enterobacter | Enterobacter sp. SST3 | 0.00002 |
| Proteobacteria | Enterobacteriales | Enterobacter | Enterobacter sp. YD4 | 0.00002 |
| Proteobacteria | Enterobacteriales | Enterobacter | Enterobacter unclassified | 0.00266 |
| Proteobacteria | Enterobacteriales | Enterobacteriaceae noname | Enterobacteriaceae bacterium 9 2 54FAA | 0.00061 |
| Proteobacteria | Enterobacteriales | Enterobacteriaceae noname | Enterobacteriaceae bacterium ATCC 29904 | 0.00002 |
| Proteobacteria | Enterobacteriales | Enterobacteriaceae noname | Enterobacteriaceae bacterium B14 | 0.00002 |
| Proteobacteria | Enterobacteriales | Enterobacteriaceae noname | Enterobacteriaceae bacterium bta3-1 | 0.00013 |
| Proteobacteria | Enterobacteriales | Enterobacteriaceae noname | Enterobacteriaceae bacterium LSJC7 | 0.00003 |
| Proteobacteria | Enterobacteriales | Enterobacteriaceae noname | Enterobacteriaceae bacterium strain FGI 57 | 0.00002 |
| Proteobacteria | Enterobacteriales | Enterobacteriaceae unclassified | Enterobacteriaceae unclassified | 16.71924 |
| Proteobacteria | Enterobacteriales | Erwinia | Erwinia amylovora | 0.00004 |
| Proteobacteria | Enterobacteriales | Erwinia | Erwinia oleae | 0.00003 |
| Proteobacteria | Enterobacteriales | Erwinia | Erwinia pyrifoliae | 0.00365 |
| Proteobacteria | Enterobacteriales | Erwinia | Erwinia tasmaniensis | 0.00001 |
| Proteobacteria | Enterobacteriales | Erwinia | Erwinia toletana | 0.00005 |
| Proteobacteria | Enterobacteriales | Erwinia | Erwinia typographi | 0.00002 |
| Proteobacteria | Enterobacteriales | Escherichia | Escherichia albertii | 0.04111 |
| Proteobacteria | Enterobacteriales | Escherichia | Escherichia coli | 22.44728 |
| Proteobacteria | Enterobacteriales | Escherichia | Escherichia coli CAG:4 | 0.00789 |
| Proteobacteria | Enterobacteriales | Escherichia | Escherichia fergusonii | 0.09368 |
| Proteobacteria | Enterobacteriales | Escherichia | Escherichia hermannii | 0.00014 |
| Proteobacteria | Enterobacteriales | Escherichia | Escherichia sp. 1 1 43 | 0.02699 |
| Proteobacteria | Enterobacteriales | Escherichia | Escherichia sp. 3 2 53FAA | 0.02439 |
| Proteobacteria | Enterobacteriales | Escherichia | Escherichia sp. HT073016 | 0.00045 |
| Proteobacteria | Enterobacteriales | Escherichia | Escherichia sp. KTE114 | 0.00012 |
| Proteobacteria | Enterobacteriales | Escherichia | Escherichia sp. KTE159 | 0.00025 |
| Proteobacteria | Enterobacteriales | Escherichia | Escherichia sp. KTE52 | 0.00030 |
| Proteobacteria | Enterobacteriales | Escherichia | Escherichia sp. TW09231 | 0.01502 |
| Proteobacteria | Enterobacteriales | Escherichia | Escherichia sp. TW09276 | 0.00110 |
| Proteobacteria | Enterobacteriales | Escherichia | Escherichia sp. TW09308 | 0.00824 |
| Proteobacteria | Enterobacteriales | Escherichia | Escherichia sp. TW10509 | 0.00039 |
| Proteobacteria | Enterobacteriales | Escherichia | Escherichia sp. TW14182 | 0.00269 |
| Proteobacteria | Enterobacteriales | Escherichia | Escherichia sp. TW15838 | 0.00601 |
| Proteobacteria | Enterobacteriales | Escherichia | Escherichia unclassified | 1.68247 |
| Proteobacteria | Enterobacteriales | Escherichia | Escherichia vulneris | 0.00060 |
| Proteobacteria | Enterobacteriales | Ewingella | Ewingella americana | 0.00004 |
| Proteobacteria | Enterobacteriales | Franconibacter | Franconibacter pulveris | 0.00003 |
| Proteobacteria | Enterobacteriales | Hafnia | Hafnia alvei | 0.00167 |
| Proteobacteria | Enterobacteriales | Hafnia | Hafnia paralvei | 0.00075 |
| Proteobacteria | Enterobacteriales | Hafnia | Hafnia unclassified | 0.00017 |
| Proteobacteria | Enterobacteriales | Klebsiella | Klebsiella michiganensis | 0.00007 |
| Proteobacteria | Enterobacteriales | Klebsiella | Klebsiella oxytoca | 0.00037 |
| Proteobacteria | Enterobacteriales | Klebsiella | Klebsiella pneumoniae | 0.14015 |
| Proteobacteria | Enterobacteriales | Klebsiella | Klebsiella sp. | 0.00004 |
| Proteobacteria | Enterobacteriales | Klebsiella | Klebsiella sp. 07A044 | 0.00001 |
| Proteobacteria | Enterobacteriales | Klebsiella | Klebsiella sp. 10982 | 0.00057 |
| Proteobacteria | Enterobacteriales | Klebsiella | Klebsiella sp. 18A069 | 0.00082 |
| Proteobacteria | Enterobacteriales | Klebsiella | Klebsiella sp. AS10 | 0.00002 |
| Proteobacteria | Enterobacteriales | Klebsiella | Klebsiella sp. MS 92-3 | 0.00157 |
| Proteobacteria | Enterobacteriales | Klebsiella | Klebsiella unclassified | 0.00064 |
| Proteobacteria | Enterobacteriales | Klebsiella | Klebsiella variicola | 0.00075 |
| Proteobacteria | Enterobacteriales | Kluyvera | Kluyvera ascorbata | 0.00032 |
| Proteobacteria | Enterobacteriales | Kosakonia | Kosakonia oryzae | 0.00005 |
| Proteobacteria | Enterobacteriales | Leclercia | Leclercia adecarboxylata | 0.00021 |
| Proteobacteria | Enterobacteriales | Leminorella | Leminorella grimontii | 0.00019 |
| Proteobacteria | Enterobacteriales | Mangrovibacter | Mangrovibacter sp. MFB070 | 0.00014 |
| Proteobacteria | Enterobacteriales | Morganella | Morganella morganii | 0.00822 |
| Proteobacteria | Enterobacteriales | Pantoea | Pantoea agglomerans | 0.00001 |
| Proteobacteria | Enterobacteriales | Pantoea | Pantoea ananatis | 0.00001 |
| Proteobacteria | Enterobacteriales | Pantoea | Pantoea dispersa | 0.00009 |
| Proteobacteria | Enterobacteriales | Pantoea | Pantoea rodasii | 0.00000 |
| Proteobacteria | Enterobacteriales | Pantoea | Pantoea sp. A4 | 0.00001 |
| Proteobacteria | Enterobacteriales | Pantoea | Pantoea sp. AS-PWVM4 | 0.00008 |
| Proteobacteria | Enterobacteriales | Pantoea | Pantoea sp. At-9b | 0.00005 |
| Proteobacteria | Enterobacteriales | Pantoea | Pantoea sp. FF5 | 0.00004 |
| Proteobacteria | Enterobacteriales | Pantoea | Pantoea sp. GM01 | 0.00004 |
| Proteobacteria | Enterobacteriales | Pantoea | Pantoea sp. IMH | 0.00154 |
| Proteobacteria | Enterobacteriales | Pantoea | Pantoea sp. PSNIH1 | 0.00013 |
| Proteobacteria | Enterobacteriales | Pantoea | Pantoea sp. SM3 | 0.00008 |
| Proteobacteria | Enterobacteriales | Pantoea | Pantoea stewartii | 0.00002 |
| Proteobacteria | Enterobacteriales | Pantoea | Pantoea unclassified | 0.00006 |
| Proteobacteria | Enterobacteriales | Pectobacterium | Pectobacterium atrosepticum | 0.00025 |
| Proteobacteria | Enterobacteriales | Pectobacterium | Pectobacterium betavasculorum | 0.00004 |
| Proteobacteria | Enterobacteriales | Pectobacterium | Pectobacterium carotovorum | 0.00055 |
| Proteobacteria | Enterobacteriales | Pectobacterium | Pectobacterium sp. SCC3193 | 0.00015 |
| Proteobacteria | Enterobacteriales | Pectobacterium | Pectobacterium wasabiae | 0.00005 |
| Proteobacteria | Enterobacteriales | Photorhabdus | Photorhabdus asymbiotica | 0.00020 |
| Proteobacteria | Enterobacteriales | Photorhabdus | Photorhabdus luminescens | 0.00046 |
| Proteobacteria | Enterobacteriales | Photorhabdus | Photorhabdus temperata | 0.00017 |
| Proteobacteria | Enterobacteriales | Plesiomonas | Plesiomonas shigelloides | 0.07272 |
| Proteobacteria | Enterobacteriales | Pluralibacter | Pluralibacter gergoviae | 0.00012 |
| Proteobacteria | Enterobacteriales | Proteus | Proteus hauseri | 0.00008 |
| Proteobacteria | Enterobacteriales | Proteus | Proteus mirabilis | 0.00112 |
| Proteobacteria | Enterobacteriales | Proteus | Proteus penneri | 0.00007 |
| Proteobacteria | Enterobacteriales | Proteus | Proteus vulgaris | 0.00005 |
| Proteobacteria | Enterobacteriales | Providencia | Providencia alcalifaciens | 0.00100 |
| Proteobacteria | Enterobacteriales | Providencia | Providencia burhodogranariea | 0.00014 |
| Proteobacteria | Enterobacteriales | Providencia | Providencia rettgeri | 0.00038 |
| Proteobacteria | Enterobacteriales | Providencia | Providencia rustigianii | 0.00028 |
| Proteobacteria | Enterobacteriales | Providencia | Providencia sneebia | 0.00035 |
| Proteobacteria | Enterobacteriales | Providencia | Providencia stuartii | 0.00053 |
| Proteobacteria | Enterobacteriales | Rahnella | Rahnella aquatilis | 0.00058 |
| Proteobacteria | Enterobacteriales | Rahnella | Rahnella sp. WMR104 | 0.00030 |
| Proteobacteria | Enterobacteriales | Rahnella | Rahnella sp. WMR66 | 0.00010 |
| Proteobacteria | Enterobacteriales | Rahnella | Rahnella sp. WP5 | 0.00007 |
| Proteobacteria | Enterobacteriales | Rahnella | Rahnella unclassified | 0.00048 |
| Proteobacteria | Enterobacteriales | Raoultella | Raoultella ornithinolytica | 0.00092 |
| Proteobacteria | Enterobacteriales | Rouxiella | Rouxiella chamberiensis | 0.00005 |
| Proteobacteria | Enterobacteriales | Salmonella | Salmonella bongori | 0.00003 |
| Proteobacteria | Enterobacteriales | Salmonella | Salmonella enterica | 0.25144 |
| Proteobacteria | Enterobacteriales | Serratia | Serratia entomophila | 0.00001 |
| Proteobacteria | Enterobacteriales | Serratia | Serratia fonticola | 0.00020 |
| Proteobacteria | Enterobacteriales | Serratia | Serratia liquefaciens | 0.00004 |
| Proteobacteria | Enterobacteriales | Serratia | Serratia marcescens | 0.00029 |
| Proteobacteria | Enterobacteriales | Serratia | Serratia nematodiphila | 0.00032 |
| Proteobacteria | Enterobacteriales | Serratia | Serratia odorifera | 0.00002 |
| Proteobacteria | Enterobacteriales | Serratia | Serratia plymuthica | 0.00048 |
| Proteobacteria | Enterobacteriales | Serratia | Serratia proteamaculans | 0.00009 |
| Proteobacteria | Enterobacteriales | Serratia | Serratia sp. DD3 | 0.00102 |
| Proteobacteria | Enterobacteriales | Serratia | Serratia sp. FS14 | 0.00004 |
| Proteobacteria | Enterobacteriales | Serratia | Serratia symbiotica | 0.00088 |
| Proteobacteria | Enterobacteriales | Serratia | Serratia unclassified | 0.00045 |
| Proteobacteria | Enterobacteriales | Shigella | Shigella boydii | 0.11318 |
| Proteobacteria | Enterobacteriales | Shigella | Shigella dysenteriae | 0.34741 |
| Proteobacteria | Enterobacteriales | Shigella | Shigella flexneri | 0.50471 |
| Proteobacteria | Enterobacteriales | Shigella | Shigella sonnei | 0.27367 |
| Proteobacteria | Enterobacteriales | Shigella | Shigella sp. MO17 | 0.00021 |
| Proteobacteria | Enterobacteriales | Shigella | Shigella unclassified | 0.04448 |
| Proteobacteria | Enterobacteriales | Shimwellia | Shimwellia blattae | 0.00043 |
| Proteobacteria | Enterobacteriales | Siccibacter | Siccibacter turicensis | 0.01020 |
| Proteobacteria | Enterobacteriales | Sodalis | Candidatus Sodalis pierantonius | 0.00002 |
| Proteobacteria | Enterobacteriales | Sodalis | Sodalis glossinidius | 0.00001 |
| Proteobacteria | Enterobacteriales | Sodalis | Sodalis praecaptivus | 0.00014 |
| Proteobacteria | Enterobacteriales | Tatumella | Tatumella morbirosei | 0.00013 |
| Proteobacteria | Enterobacteriales | Tatumella | Tatumella sp. UCD-D suzukii | 0.00001 |
| Proteobacteria | Enterobacteriales | Trabulsiella | Trabulsiella guamensis | 0.00020 |
| Proteobacteria | Enterobacteriales | Xenorhabdus | Xenorhabdus bovienii | 0.00040 |
| Proteobacteria | Enterobacteriales | Xenorhabdus | Xenorhabdus cabanillasii | 0.00027 |
| Proteobacteria | Enterobacteriales | Xenorhabdus | Xenorhabdus doucetiae | 0.00009 |
| Proteobacteria | Enterobacteriales | Xenorhabdus | Xenorhabdus nematophila | 0.00045 |
| Proteobacteria | Enterobacteriales | Xenorhabdus | Xenorhabdus szentirmaii | 0.00008 |
| Proteobacteria | Enterobacteriales | Yersinia | Yersinia aleksiciae | 0.00001 |
| Proteobacteria | Enterobacteriales | Yersinia | Yersinia bercovieri | 0.00002 |
| Proteobacteria | Enterobacteriales | Yersinia | Yersinia enterocolitica | 0.00077 |
| Proteobacteria | Enterobacteriales | Yersinia | Yersinia frederiksenii | 0.00014 |
| Proteobacteria | Enterobacteriales | Yersinia | Yersinia massiliensis | 0.00005 |
| Proteobacteria | Enterobacteriales | Yersinia | Yersinia pestis | 0.00831 |
| Proteobacteria | Enterobacteriales | Yersinia | Yersinia pseudotuberculosis | 0.01047 |
| Proteobacteria | Enterobacteriales | Yersinia | Yersinia rohdei | 0.00003 |
| Proteobacteria | Enterobacteriales | Yersinia | Yersinia ruckeri | 0.00035 |
| Proteobacteria | Enterobacteriales | Yersinia | Yersinia unclassified | 0.00003 |
| Proteobacteria | Enterobacteriales | Yokenella | Yokenella regensburgei | 0.00023 |
| Proteobacteria | Epsilonproteobacteria noname | Nitratiruptor | Nitratiruptor sp. SB155-2 | 0.00002 |
| Proteobacteria | Epsilonproteobacteria noname | Sulfurovum | Sulfurovum sp. AR | 0.00001 |
| Proteobacteria | Epsilonproteobacteria noname | Sulfurovum | Sulfurovum sp. AS07-7 | 0.00065 |
| Proteobacteria | Gammaproteobacteria noname | Candidatus Competibacter | Candidatus Competibacter denitrificans | 0.00005 |
| Proteobacteria | Gammaproteobacteria noname | Candidatus Contendobacter | Candidatus Contendobacter odensis | 0.00003 |
| Proteobacteria | Gammaproteobacteria noname | Gallaecimonas | Gallaecimonas xiamenensis | 0.00004 |
| Proteobacteria | Gammaproteobacteria noname | Gammaproteobacteria noname | Gammaproteobacteria bacterium MFB021 | 0.00001 |
| Proteobacteria | Gammaproteobacteria noname | Porticoccus | Porticoccus hydrocarbonoclasticus | 0.00002 |
| Proteobacteria | Gammaproteobacteria noname | Sedimenticola | Sedimenticola selenatireducens | 0.00007 |
| Proteobacteria | Gammaproteobacteria noname | Thiolapillus | Thiolapillus brandeum | 0.00001 |
| Proteobacteria | Gammaproteobacteria unclassified | Gammaproteobacteria unclassified | Gammaproteobacteria unclassified | 0.12821 |
| Proteobacteria | Hydrogenophilales | Thiobacillus | Thiobacillus denitrificans | 0.00005 |
| Proteobacteria | Kordiimonadales | Kordiimonas | Kordiimonas gwangyangensis | 0.00011 |
| Proteobacteria | Legionellales | Fluoribacter | Fluoribacter dumoffii | 0.00002 |
| Proteobacteria | Legionellales | Legionella | Legionella drancourtii | 0.00001 |
| Proteobacteria | Legionellales | Legionella | Legionella longbeachae | 0.00212 |
| Proteobacteria | Legionellales | Legionella | Legionella shakespearei | 0.00001 |
| Proteobacteria | Methylococcales | Methylobacter | Methylobacter tundripaludum | 0.00002 |
| Proteobacteria | Methylococcales | Methylomicrobium | Methylomicrobium agile | 0.00002 |
| Proteobacteria | Methylococcales | Methylomicrobium | Methylomicrobium album | 0.00004 |
| Proteobacteria | Methylophilales | Methylophilaceae noname | Methylophilaceae bacterium MMS-2-53 | 0.00017 |
| Proteobacteria | Methylophilales | Methylophilus | Methylophilus unclassified | 0.00003 |
| Proteobacteria | Methylophilales | Methylotenera | Methylotenera mobilis | 0.00002 |
| Proteobacteria | Methylophilales | Methylotenera | Methylotenera sp. 1P/1 | 0.00007 |
| Proteobacteria | Myxococcales | Cystobacter | Cystobacter fuscus | 0.00003 |
| Proteobacteria | Nautiliales | Lebetimonas | Lebetimonas sp. JS032 | 0.00005 |
| Proteobacteria | Neisseriales | Alysiella | Alysiella crassa | 0.00044 |
| Proteobacteria | Neisseriales | Aquitalea | Aquitalea magnusonii | 0.00003 |
| Proteobacteria | Neisseriales | Chromobacterium | Chromobacterium haemolyticum | 0.00017 |
| Proteobacteria | Neisseriales | Chromobacterium | Chromobacterium piscinae | 0.00033 |
| Proteobacteria | Neisseriales | Chromobacterium | Chromobacterium sp. C-61 | 0.00022 |
| Proteobacteria | Neisseriales | Chromobacterium | Chromobacterium subtsugae | 0.00003 |
| Proteobacteria | Neisseriales | Chromobacterium | Chromobacterium vaccinii | 0.00003 |
| Proteobacteria | Neisseriales | Chromobacterium | Chromobacterium violaceum | 0.00009 |
| Proteobacteria | Neisseriales | Conchiformibius | Conchiformibius steedae | 0.00030 |
| Proteobacteria | Neisseriales | Eikenella | Eikenella corrodens | 0.00001 |
| Proteobacteria | Neisseriales | Kingella | Kingella kingae | 0.00036 |
| Proteobacteria | Neisseriales | Kingella | Kingella oralis | 0.00003 |
| Proteobacteria | Neisseriales | Kingella | Kingella sp. Sch538 | 0.00005 |
| Proteobacteria | Neisseriales | Laribacter | Laribacter hongkongensis | 0.00139 |
| Proteobacteria | Neisseriales | Leeia | Leeia oryzae | 0.00003 |
| Proteobacteria | Neisseriales | Microvirgula | Microvirgula aerodenitrificans | 0.00007 |
| Proteobacteria | Neisseriales | Neisseria | Neisseria bacilliformis | 0.00005 |
| Proteobacteria | Neisseriales | Neisseria | Neisseria elongata | 0.00013 |
| Proteobacteria | Neisseriales | Neisseria | Neisseria gonorrhoeae | 0.00019 |
| Proteobacteria | Neisseriales | Neisseria | Neisseria lactamica | 0.00029 |
| Proteobacteria | Neisseriales | Neisseria | Neisseria meningitidis | 0.00014 |
| Proteobacteria | Neisseriales | Neisseria | Neisseria polysaccharea | 0.00006 |
| Proteobacteria | Neisseriales | Neisseria | Neisseria shayeganii | 0.00012 |
| Proteobacteria | Neisseriales | Neisseria | Neisseria sicca | 0.00011 |
| Proteobacteria | Neisseriales | Neisseria | Neisseria sp. oral taxon 014 | 0.00008 |
| Proteobacteria | Neisseriales | Neisseria | Neisseria sp. oral taxon 020 | 0.00009 |
| Proteobacteria | Neisseriales | Neisseria | Neisseria subflava | 0.00006 |
| Proteobacteria | Neisseriales | Neisseria | Neisseria unclassified | 0.00003 |
| Proteobacteria | Neisseriales | Neisseria | Neisseria wadsworthii | 0.00014 |
| Proteobacteria | Neisseriales | Neisseria | Neisseria weaveri | 0.00010 |
| Proteobacteria | Neisseriales | Paludibacterium | Paludibacterium yongneupense | 0.00008 |
| Proteobacteria | Neisseriales | Pseudogulbenkiania | Pseudogulbenkiania ferrooxidans | 0.00007 |
| Proteobacteria | Neisseriales | Pseudogulbenkiania | Pseudogulbenkiania sp. MAI-1 | 0.00011 |
| Proteobacteria | Neisseriales | Simonsiella | Simonsiella muelleri | 0.00007 |
| Proteobacteria | Neisseriales | Snodgrassella | Snodgrassella alvi | 0.00079 |
| Proteobacteria | Neisseriales | Stenoxybacter | Stenoxybacter acetivorans | 0.00011 |
| Proteobacteria | Neisseriales | Vitreoscilla | Vitreoscilla stercoraria | 0.00040 |
| Proteobacteria | Nitrosomonadales | Nitrosomonas | Nitrosomonas sp. AL212 | 0.00004 |
| Proteobacteria | Nitrosomonadales | Nitrosospira | Nitrosospira briensis | 0.00003 |
| Proteobacteria | Nitrosomonadales | Nitrosospira | Nitrosospira multiformis | 0.00002 |
| Proteobacteria | Oceanospirillales | Alcanivorax | Alcanivorax hongdengensis | 0.00001 |
| Proteobacteria | Oceanospirillales | Alcanivorax | Alcanivorax jadensis | 0.00003 |
| Proteobacteria | Oceanospirillales | Alcanivorax | Alcanivorax pacificus | 0.00012 |
| Proteobacteria | Oceanospirillales | Alcanivorax | Alcanivorax sp. 19-m-6 | 0.00005 |
| Proteobacteria | Oceanospirillales | Alcanivorax | Alcanivorax sp. 97CO-5 | 0.00001 |
| Proteobacteria | Oceanospirillales | Alcanivorax | Alcanivorax sp. PN-3 | 0.00004 |
| Proteobacteria | Oceanospirillales | Carnimonas | Carnimonas nigrificans | 0.00003 |
| Proteobacteria | Oceanospirillales | Chromohalobacter | Chromohalobacter salexigens | 0.00003 |
| Proteobacteria | Oceanospirillales | Endozoicomonas | Endozoicomonas elysicola | 0.00011 |
| Proteobacteria | Oceanospirillales | Endozoicomonas | Endozoicomonas montiporae | 0.00142 |
| Proteobacteria | Oceanospirillales | Endozoicomonas | Endozoicomonas numazuensis | 0.00007 |
| Proteobacteria | Oceanospirillales | Hahella | Hahella chejuensis | 0.00002 |
| Proteobacteria | Oceanospirillales | Halomonas | Halomonas alkaliantarctica | 0.00001 |
| Proteobacteria | Oceanospirillales | Halomonas | Halomonas anticariensis | 0.00006 |
| Proteobacteria | Oceanospirillales | Halomonas | Halomonas campaniensis | 0.00007 |
| Proteobacteria | Oceanospirillales | Halomonas | Halomonas halocynthiae | 0.00005 |
| Proteobacteria | Oceanospirillales | Halomonas | Halomonas meridiana | 0.00001 |
| Proteobacteria | Oceanospirillales | Halomonas | Halomonas sp. A3H3 | 0.00001 |
| Proteobacteria | Oceanospirillales | Halomonas | Halomonas sp. BJGMM-B45 | 0.00008 |
| Proteobacteria | Oceanospirillales | Halomonas | Halomonas sp. HAL1 | 0.00001 |
| Proteobacteria | Oceanospirillales | Halomonas | Halomonas sp. HL-48 | 0.00005 |
| Proteobacteria | Oceanospirillales | Halomonas | Halomonas sp. S2151 | 0.00001 |
| Proteobacteria | Oceanospirillales | Halomonas | Halomonas sp. TG39a | 0.00001 |
| Proteobacteria | Oceanospirillales | Halomonas | Halomonas zhanjiangensis | 0.00001 |
| Proteobacteria | Oceanospirillales | Halotalea | Halotalea alkalilenta | 0.00003 |
| Proteobacteria | Oceanospirillales | Kangiella | Kangiella aquimarina | 0.00001 |
| Proteobacteria | Oceanospirillales | Kangiella | Kangiella koreensis | 0.00008 |
| Proteobacteria | Oceanospirillales | Kushneria | Kushneria aurantia | 0.00001 |
| Proteobacteria | Oceanospirillales | Marinomonas | Marinomonas profundimaris | 0.00002 |
| Proteobacteria | Oceanospirillales | Marinospirillum | Marinospirillum insulare | 0.00011 |
| Proteobacteria | Oceanospirillales | Marinospirillum | Marinospirillum minutulum | 0.00010 |
| Proteobacteria | Oceanospirillales | Nitrincola | Nitrincola lacisaponensis | 0.00001 |
| Proteobacteria | Oceanospirillales | Nitrincola | Nitrincola sp. AK23 | 0.00002 |
| Proteobacteria | Oceanospirillales | Oceanospirillum | Oceanospirillum beijerinckii | 0.00003 |
| Proteobacteria | Oceanospirillales | Oceanospirillum | Oceanospirillum maris | 0.00002 |
| Proteobacteria | Oceanospirillales | Oleispira | Oleispira antarctica | 0.00016 |
| Proteobacteria | Oceanospirillales | Zooshikella | Zooshikella ganghwensis | 0.00026 |
| Proteobacteria | Oceanospirillales | Zymobacter | Zymobacter palmae | 0.00008 |
| Proteobacteria | Orbales | Candidatus Schmidhempelia | Candidatus Schmidhempelia bombi | 0.00002 |
| Proteobacteria | Orbales | Frischella | Frischella perrara | 0.00012 |
| Proteobacteria | Orbales | Gilliamella | Gilliamella apicola | 0.00058 |
| Proteobacteria | Parvularculales | Parvularcula | Parvularcula bermudensis | 0.00056 |
| Proteobacteria | Pasteurellales | Actinobacillus | Actinobacillus capsulatus | 0.00009 |
| Proteobacteria | Pasteurellales | Actinobacillus | Actinobacillus equuli | 0.00014 |
| Proteobacteria | Pasteurellales | Actinobacillus | Actinobacillus minor | 0.00011 |
| Proteobacteria | Pasteurellales | Actinobacillus | Actinobacillus pleuropneumoniae | 0.00295 |
| Proteobacteria | Pasteurellales | Actinobacillus | Actinobacillus unclassified | 0.00003 |
| Proteobacteria | Pasteurellales | Actinobacillus | Actinobacillus ureae | 0.00012 |
| Proteobacteria | Pasteurellales | Aggregatibacter | Aggregatibacter actinomycetemcomitans | 0.00078 |
| Proteobacteria | Pasteurellales | Aggregatibacter | Aggregatibacter aphrophilus | 0.00008 |
| Proteobacteria | Pasteurellales | Aggregatibacter | Aggregatibacter sp. oral taxon 458 | 0.00002 |
| Proteobacteria | Pasteurellales | Avibacterium | Avibacterium paragallinarum | 0.00135 |
| Proteobacteria | Pasteurellales | Basfia | [Mannheimia] succiniciproducens | 0.00098 |
| Proteobacteria | Pasteurellales | Bibersteinia | Bibersteinia trehalosi | 0.00001 |
| Proteobacteria | Pasteurellales | Chelonobacter | Chelonobacter oris | 0.00153 |
| Proteobacteria | Pasteurellales | Gallibacterium | Gallibacterium anatis | 0.00200 |
| Proteobacteria | Pasteurellales | Gallibacterium | Gallibacterium genomosp. 1 | 0.00011 |
| Proteobacteria | Pasteurellales | Gallibacterium | Gallibacterium genomosp. 2 | 0.00020 |
| Proteobacteria | Pasteurellales | Haemophilus | Haemophilus haemolyticus | 0.00054 |
| Proteobacteria | Pasteurellales | Haemophilus | Haemophilus influenzae | 0.00254 |
| Proteobacteria | Pasteurellales | Haemophilus | Haemophilus parahaemolyticus | 0.00066 |
| Proteobacteria | Pasteurellales | Haemophilus | Haemophilus parainfluenzae | 0.00002 |
| Proteobacteria | Pasteurellales | Haemophilus | Haemophilus paraphrohaemolyticus | 0.00067 |
| Proteobacteria | Pasteurellales | Haemophilus | Haemophilus parasuis | 0.00324 |
| Proteobacteria | Pasteurellales | Haemophilus | Haemophilus pittmaniae | 0.00002 |
| Proteobacteria | Pasteurellales | Haemophilus | Haemophilus sp. FF7 | 0.00006 |
| Proteobacteria | Pasteurellales | Haemophilus | Haemophilus sputorum | 0.00005 |
| Proteobacteria | Pasteurellales | Haemophilus | Haemophilus unclassified | 0.00005 |
| Proteobacteria | Pasteurellales | Histophilus | Histophilus somni | 0.00670 |
| Proteobacteria | Pasteurellales | Mannheimia | Mannheimia granulomatis | 0.00005 |
| Proteobacteria | Pasteurellales | Mannheimia | Mannheimia haemolytica | 0.00532 |
| Proteobacteria | Pasteurellales | Mannheimia | Mannheimia unclassified | 0.00002 |
| Proteobacteria | Pasteurellales | Mannheimia | Mannheimia varigena | 0.00057 |
| Proteobacteria | Pasteurellales | Pasteurella | [Pasteurella] pneumotropica | 0.00225 |
| Proteobacteria | Pasteurellales | Pasteurella | Pasteurella bettyae | 0.00023 |
| Proteobacteria | Pasteurellales | Pasteurella | Pasteurella dagmatis | 0.00319 |
| Proteobacteria | Pasteurellales | Pasteurella | Pasteurella multocida | 0.01118 |
| Proteobacteria | Pasteurellales | Pasteurella | Pasteurella sp. FF6 | 0.00014 |
| Proteobacteria | Pasteurellales | Pasteurellaceae unclassified | Pasteurellaceae unclassified | 0.00003 |
| Proteobacteria | Proteobacteria noname | Proteobacteria noname | Proteobacteria bacterium CAG:139 | 0.00043 |
| Proteobacteria | Proteobacteria noname | Proteobacteria noname | Proteobacteria bacterium CAG:495 | 0.00020 |
| Proteobacteria | Proteobacteria noname | Proteobacteria noname | Proteobacteria bacterium JGI 0000113-E04 | 0.00013 |
| Proteobacteria | Proteobacteria noname | Proteobacteria noname | Proteobacteria bacterium JGI 0000113-L05 | 0.00049 |
| Proteobacteria | Proteobacteria noname | Proteobacteria noname | Proteobacteria bacterium JGI 0000113-P07 | 0.00170 |
| Proteobacteria | Proteobacteria noname | Proteobacteria noname | Proteobacteria bacterium JGI 0001009-D17 | 0.00001 |
| Proteobacteria | Proteobacteria unclassified | Proteobacteria unclassified | Proteobacteria unclassified | 0.04181 |
| Proteobacteria | Pseudomonadales | Acinetobacter | Acinetobacter baumannii | 0.07170 |
| Proteobacteria | Pseudomonadales | Acinetobacter | Acinetobacter bereziniae | 0.00011 |
| Proteobacteria | Pseudomonadales | Acinetobacter | Acinetobacter bouvetii | 0.00020 |
| Proteobacteria | Pseudomonadales | Acinetobacter | Acinetobacter brisouii | 0.00012 |
| Proteobacteria | Pseudomonadales | Acinetobacter | Acinetobacter calcoaceticus | 0.00024 |
| Proteobacteria | Pseudomonadales | Acinetobacter | Acinetobacter gerneri | 0.00030 |
| Proteobacteria | Pseudomonadales | Acinetobacter | Acinetobacter guillouiae | 0.00023 |
| Proteobacteria | Pseudomonadales | Acinetobacter | Acinetobacter gyllenbergii | 0.00002 |
| Proteobacteria | Pseudomonadales | Acinetobacter | Acinetobacter haemolyticus | 0.00014 |
| Proteobacteria | Pseudomonadales | Acinetobacter | Acinetobacter harbinensis | 0.00017 |
| Proteobacteria | Pseudomonadales | Acinetobacter | Acinetobacter indicus | 0.00004 |
| Proteobacteria | Pseudomonadales | Acinetobacter | Acinetobacter johnsonii | 0.00012 |
| Proteobacteria | Pseudomonadales | Acinetobacter | Acinetobacter junii | 0.00009 |
| Proteobacteria | Pseudomonadales | Acinetobacter | Acinetobacter lwoffii | 0.00010 |
| Proteobacteria | Pseudomonadales | Acinetobacter | Acinetobacter nosocomialis | 0.00005 |
| Proteobacteria | Pseudomonadales | Acinetobacter | Acinetobacter pittii | 0.00009 |
| Proteobacteria | Pseudomonadales | Acinetobacter | Acinetobacter rudis | 0.00042 |
| Proteobacteria | Pseudomonadales | Acinetobacter | Acinetobacter schindleri | 0.00004 |
| Proteobacteria | Pseudomonadales | Acinetobacter | Acinetobacter soli | 0.00003 |
| Proteobacteria | Pseudomonadales | Acinetobacter | Acinetobacter sp. A47 | 0.00094 |
| Proteobacteria | Pseudomonadales | Acinetobacter | Acinetobacter sp. ANC 3789 | 0.00002 |
| Proteobacteria | Pseudomonadales | Acinetobacter | Acinetobacter sp. ANC 3862 | 0.00003 |
| Proteobacteria | Pseudomonadales | Acinetobacter | Acinetobacter sp. ATCC 27244 | 0.00001 |
| Proteobacteria | Pseudomonadales | Acinetobacter | Acinetobacter sp. CAG:196 | 0.00258 |
| Proteobacteria | Pseudomonadales | Acinetobacter | Acinetobacter sp. CIP 101934 | 0.00002 |
| Proteobacteria | Pseudomonadales | Acinetobacter | Acinetobacter sp. CIP 102136 | 0.00003 |
| Proteobacteria | Pseudomonadales | Acinetobacter | Acinetobacter sp. CIP 53.82 | 0.00004 |
| Proteobacteria | Pseudomonadales | Acinetobacter | Acinetobacter sp. CIP 64.2 | 0.00009 |
| Proteobacteria | Pseudomonadales | Acinetobacter | Acinetobacter sp. CIP 64.7 | 0.00002 |
| Proteobacteria | Pseudomonadales | Acinetobacter | Acinetobacter sp. CIP-A165 | 0.00007 |
| Proteobacteria | Pseudomonadales | Acinetobacter | Acinetobacter sp. COS3 | 0.00005 |
| Proteobacteria | Pseudomonadales | Acinetobacter | Acinetobacter sp. ETR1 | 0.00003 |
| Proteobacteria | Pseudomonadales | Acinetobacter | Acinetobacter sp. HR7 | 0.00009 |
| Proteobacteria | Pseudomonadales | Acinetobacter | Acinetobacter sp. MDS7A | 0.00023 |
| Proteobacteria | Pseudomonadales | Acinetobacter | Acinetobacter sp. MII | 0.00005 |
| Proteobacteria | Pseudomonadales | Acinetobacter | Acinetobacter sp. NBRC 110496 | 0.00002 |
| Proteobacteria | Pseudomonadales | Acinetobacter | Acinetobacter sp. neg1 | 0.00005 |
| Proteobacteria | Pseudomonadales | Acinetobacter | Acinetobacter sp. NIPH 2168 | 0.00011 |
| Proteobacteria | Pseudomonadales | Acinetobacter | Acinetobacter sp. NIPH 2171 | 0.00003 |
| Proteobacteria | Pseudomonadales | Acinetobacter | Acinetobacter sp. NIPH 236 | 0.00001 |
| Proteobacteria | Pseudomonadales | Acinetobacter | Acinetobacter sp. NIPH 713 | 0.00002 |
| Proteobacteria | Pseudomonadales | Acinetobacter | Acinetobacter sp. NIPH 899 | 0.00001 |
| Proteobacteria | Pseudomonadales | Acinetobacter | Acinetobacter sp. NIPH 973 | 0.00001 |
| Proteobacteria | Pseudomonadales | Acinetobacter | Acinetobacter sp. P8-3-8 | 0.00001 |
| Proteobacteria | Pseudomonadales | Acinetobacter | Acinetobacter sp. UNC436CL71CviS28 | 0.00012 |
| Proteobacteria | Pseudomonadales | Acinetobacter | Acinetobacter sp. WC-323 | 0.00002 |
| Proteobacteria | Pseudomonadales | Acinetobacter | Acinetobacter towneri | 0.00004 |
| Proteobacteria | Pseudomonadales | Acinetobacter | Acinetobacter unclassified | 0.00739 |
| Proteobacteria | Pseudomonadales | Acinetobacter | Acinetobacter ursingii | 0.00013 |
| Proteobacteria | Pseudomonadales | Acinetobacter | Acinetobacter venetianus | 0.00002 |
| Proteobacteria | Pseudomonadales | Alkanindiges | Alkanindiges illinoisensis | 0.00010 |
| Proteobacteria | Pseudomonadales | Azotobacter | Azotobacter chroococcum | 0.00008 |
| Proteobacteria | Pseudomonadales | Azotobacter | Azotobacter vinelandii | 0.00017 |
| Proteobacteria | Pseudomonadales | Cellvibrio | Cellvibrio mixtus | 0.00008 |
| Proteobacteria | Pseudomonadales | Enhydrobacter | Enhydrobacter aerosaccus | 0.00018 |
| Proteobacteria | Pseudomonadales | Moraxella | Moraxella boevrei | 0.00006 |
| Proteobacteria | Pseudomonadales | Moraxella | Moraxella bovoculi | 0.00012 |
| Proteobacteria | Pseudomonadales | Moraxella | Moraxella caprae | 0.00022 |
| Proteobacteria | Pseudomonadales | Moraxella | Moraxella catarrhalis | 0.00145 |
| Proteobacteria | Pseudomonadales | Moraxella | Moraxella macacae | 0.00006 |
| Proteobacteria | Pseudomonadales | Moraxella | Moraxella sp. TA144 | 0.00008 |
| Proteobacteria | Pseudomonadales | Moraxellaceae unclassified | Moraxellaceae unclassified | 0.00006 |
| Proteobacteria | Pseudomonadales | Pseudomonas | Pseudomonas aeruginosa | 0.00063 |
| Proteobacteria | Pseudomonadales | Pseudomonas | Pseudomonas alcaligenes | 0.00001 |
| Proteobacteria | Pseudomonadales | Pseudomonas | Pseudomonas alcaliphila | 0.00002 |
| Proteobacteria | Pseudomonadales | Pseudomonas | Pseudomonas alkylphenolia | 0.00002 |
| Proteobacteria | Pseudomonadales | Pseudomonas | Pseudomonas azotifigens | 0.00006 |
| Proteobacteria | Pseudomonadales | Pseudomonas | Pseudomonas balearica | 0.00001 |
| Proteobacteria | Pseudomonadales | Pseudomonas | Pseudomonas bauzanensis | 0.00001 |
| Proteobacteria | Pseudomonadales | Pseudomonas | Pseudomonas brassicacearum | 0.00003 |
| Proteobacteria | Pseudomonadales | Pseudomonas | Pseudomonas caeni | 0.00051 |
| Proteobacteria | Pseudomonadales | Pseudomonas | Pseudomonas chloritidismutans | 0.00001 |
| Proteobacteria | Pseudomonadales | Pseudomonas | Pseudomonas chlororaphis | 0.00004 |
| Proteobacteria | Pseudomonadales | Pseudomonas | Pseudomonas cichorii | 0.00002 |
| Proteobacteria | Pseudomonadales | Pseudomonas | Pseudomonas cremoricolorata | 0.00005 |
| Proteobacteria | Pseudomonadales | Pseudomonas | Pseudomonas extremaustralis | 0.00007 |
| Proteobacteria | Pseudomonadales | Pseudomonas | Pseudomonas fluorescens | 0.00061 |
| Proteobacteria | Pseudomonadales | Pseudomonas | Pseudomonas frederiksbergensis | 0.00001 |
| Proteobacteria | Pseudomonadales | Pseudomonas | Pseudomonas fulva | 0.00069 |
| Proteobacteria | Pseudomonadales | Pseudomonas | Pseudomonas gingeri | 0.00002 |
| Proteobacteria | Pseudomonadales | Pseudomonas | Pseudomonas japonica | 0.00003 |
| Proteobacteria | Pseudomonadales | Pseudomonas | Pseudomonas knackmussii | 0.00013 |
| Proteobacteria | Pseudomonadales | Pseudomonas | Pseudomonas luteola | 0.00006 |
| Proteobacteria | Pseudomonadales | Pseudomonas | Pseudomonas mandelii | 0.00003 |
| Proteobacteria | Pseudomonadales | Pseudomonas | Pseudomonas mediterranea | 0.00003 |
| Proteobacteria | Pseudomonadales | Pseudomonas | Pseudomonas mendocina | 0.00001 |
| Proteobacteria | Pseudomonadales | Pseudomonas | Pseudomonas migulae | 0.00003 |
| Proteobacteria | Pseudomonadales | Pseudomonas | Pseudomonas mosselii | 0.00008 |
| Proteobacteria | Pseudomonadales | Pseudomonas | Pseudomonas nitroreducens | 0.00002 |
| Proteobacteria | Pseudomonadales | Pseudomonas | Pseudomonas parafulva | 0.00004 |
| Proteobacteria | Pseudomonadales | Pseudomonas | Pseudomonas pelagia | 0.00002 |
| Proteobacteria | Pseudomonadales | Pseudomonas | Pseudomonas putida | 0.00028 |
| Proteobacteria | Pseudomonadales | Pseudomonas | Pseudomonas resinovorans | 0.00001 |
| Proteobacteria | Pseudomonadales | Pseudomonas | Pseudomonas rhodesiae | 0.00001 |
| Proteobacteria | Pseudomonadales | Pseudomonas | Pseudomonas sp. 12M76 air | 0.00007 |
| Proteobacteria | Pseudomonadales | Pseudomonas | Pseudomonas sp. 2(2015) | 0.00003 |
| Proteobacteria | Pseudomonadales | Pseudomonas | Pseudomonas sp. 21 | 0.00001 |
| Proteobacteria | Pseudomonadales | Pseudomonas | Pseudomonas sp. 313 | 0.00002 |
| Proteobacteria | Pseudomonadales | Pseudomonas | Pseudomonas sp. 7197 | 0.00013 |
| Proteobacteria | Pseudomonadales | Pseudomonas | Pseudomonas sp. AAC | 0.00003 |
| Proteobacteria | Pseudomonadales | Pseudomonas | Pseudomonas sp. Ant30-3 | 0.00001 |
| Proteobacteria | Pseudomonadales | Pseudomonas | Pseudomonas sp. BRH c35 | 0.00002 |
| Proteobacteria | Pseudomonadales | Pseudomonas | Pseudomonas sp. C5pp | 0.00082 |
| Proteobacteria | Pseudomonadales | Pseudomonas | Pseudomonas sp. CB1 | 0.00003 |
| Proteobacteria | Pseudomonadales | Pseudomonas | Pseudomonas sp. CF149 | 0.00003 |
| Proteobacteria | Pseudomonadales | Pseudomonas | Pseudomonas sp. CF150 | 0.00002 |
| Proteobacteria | Pseudomonadales | Pseudomonas | Pseudomonas sp. CF161 | 0.00002 |
| Proteobacteria | Pseudomonadales | Pseudomonas | Pseudomonas sp. CFT9 | 0.00003 |
| Proteobacteria | Pseudomonadales | Pseudomonas | Pseudomonas sp. CHM02 | 0.00007 |
| Proteobacteria | Pseudomonadales | Pseudomonas | Pseudomonas sp. Chol1 | 0.00001 |
| Proteobacteria | Pseudomonadales | Pseudomonas | Pseudomonas sp. EGD-AK9 | 0.00002 |
| Proteobacteria | Pseudomonadales | Pseudomonas | Pseudomonas sp. ES3-33 | 0.00004 |
| Proteobacteria | Pseudomonadales | Pseudomonas | Pseudomonas sp. FH4 | 0.00004 |
| Proteobacteria | Pseudomonadales | Pseudomonas | Pseudomonas sp. GLE121 | 0.00010 |
| Proteobacteria | Pseudomonadales | Pseudomonas | Pseudomonas sp. GM17 | 0.00001 |
| Proteobacteria | Pseudomonadales | Pseudomonas | Pseudomonas sp. GM49 | 0.00004 |
| Proteobacteria | Pseudomonadales | Pseudomonas | Pseudomonas sp. HPB0071 | 0.00005 |
| Proteobacteria | Pseudomonadales | Pseudomonas | Pseudomonas sp. K-62 | 0.00002 |
| Proteobacteria | Pseudomonadales | Pseudomonas | Pseudomonas sp. MT-1 | 0.00003 |
| Proteobacteria | Pseudomonadales | Pseudomonas | Pseudomonas sp. P818 | 0.00011 |
| Proteobacteria | Pseudomonadales | Pseudomonas | Pseudomonas sp. PH1b | 0.00003 |
| Proteobacteria | Pseudomonadales | Pseudomonas | Pseudomonas sp. PI1 | 0.00009 |
| Proteobacteria | Pseudomonadales | Pseudomonas | Pseudomonas sp. S9 | 0.00003 |
| Proteobacteria | Pseudomonadales | Pseudomonas | Pseudomonas sp. TJI-51 | 0.00001 |
| Proteobacteria | Pseudomonadales | Pseudomonas | Pseudomonas sp. URMO17WK12:I4 | 0.00002 |
| Proteobacteria | Pseudomonadales | Pseudomonas | Pseudomonas sp. URMO17WK12:I8 | 0.00015 |
| Proteobacteria | Pseudomonadales | Pseudomonas | Pseudomonas sp. UW4 | 0.00008 |
| Proteobacteria | Pseudomonadales | Pseudomonas | Pseudomonas sp. WCS358 | 0.00002 |
| Proteobacteria | Pseudomonadales | Pseudomonas | Pseudomonas sp. WCS374 | 0.00003 |
| Proteobacteria | Pseudomonadales | Pseudomonas | Pseudomonas stutzeri | 0.00018 |
| Proteobacteria | Pseudomonadales | Pseudomonas | Pseudomonas syringae | 0.00020 |
| Proteobacteria | Pseudomonadales | Pseudomonas | Pseudomonas taeanensis | 0.00006 |
| Proteobacteria | Pseudomonadales | Pseudomonas | Pseudomonas thermotolerans | 0.00005 |
| Proteobacteria | Pseudomonadales | Pseudomonas | Pseudomonas unclassified | 0.00011 |
| Proteobacteria | Pseudomonadales | Pseudomonas | Pseudomonas veronii | 0.00002 |
| Proteobacteria | Pseudomonadales | Pseudomonas | Pseudomonas viridiflava | 0.00001 |
| Proteobacteria | Pseudomonadales | Psychrobacter | Psychrobacter aquaticus | 0.02136 |
| Proteobacteria | Pseudomonadales | Psychrobacter | Psychrobacter arcticus | 0.03149 |
| Proteobacteria | Pseudomonadales | Psychrobacter | Psychrobacter cryohalolentis | 0.02961 |
| Proteobacteria | Pseudomonadales | Psychrobacter | Psychrobacter faecalis | 0.00014 |
| Proteobacteria | Pseudomonadales | Psychrobacter | Psychrobacter lutiphocae | 0.00111 |
| Proteobacteria | Pseudomonadales | Psychrobacter | Psychrobacter maritimus | 0.00011 |
| Proteobacteria | Pseudomonadales | Psychrobacter | Psychrobacter phenylpyruvicus | 0.00091 |
| Proteobacteria | Pseudomonadales | Psychrobacter | Psychrobacter sp. 1501(2011) | 0.00417 |
| Proteobacteria | Pseudomonadales | Psychrobacter | Psychrobacter sp. 7195 | 0.00025 |
| Proteobacteria | Pseudomonadales | Psychrobacter | Psychrobacter sp. AC24 | 0.00040 |
| Proteobacteria | Pseudomonadales | Psychrobacter | Psychrobacter sp. DAB AL12 | 0.00011 |
| Proteobacteria | Pseudomonadales | Psychrobacter | Psychrobacter sp. DAB AL32B | 0.00002 |
| Proteobacteria | Pseudomonadales | Psychrobacter | Psychrobacter sp. DAB AL43B | 0.00036 |
| Proteobacteria | Pseudomonadales | Psychrobacter | Psychrobacter sp. DAB AL60 | 0.00020 |
| Proteobacteria | Pseudomonadales | Psychrobacter | Psychrobacter sp. DAB AL62B | 0.00003 |
| Proteobacteria | Pseudomonadales | Psychrobacter | Psychrobacter sp. G | 0.03722 |
| Proteobacteria | Pseudomonadales | Psychrobacter | Psychrobacter sp. J466 | 0.00034 |
| Proteobacteria | Pseudomonadales | Psychrobacter | Psychrobacter sp. JCM 18900 | 0.00735 |
| Proteobacteria | Pseudomonadales | Psychrobacter | Psychrobacter sp. JCM 18901 | 0.01080 |
| Proteobacteria | Pseudomonadales | Psychrobacter | Psychrobacter sp. JCM 18902 | 0.04446 |
| Proteobacteria | Pseudomonadales | Psychrobacter | Psychrobacter sp. JCM 18903 | 0.03190 |
| Proteobacteria | Pseudomonadales | Psychrobacter | Psychrobacter sp. PAMC 21119 | 0.02415 |
| Proteobacteria | Pseudomonadales | Psychrobacter | Psychrobacter sp. PRwf-1 | 0.00312 |
| Proteobacteria | Pseudomonadales | Psychrobacter | Psychrobacter sp. T-3 | 0.00013 |
| Proteobacteria | Pseudomonadales | Psychrobacter | Psychrobacter sp. TB15 | 0.00034 |
| Proteobacteria | Pseudomonadales | Psychrobacter | Psychrobacter sp. TB2 | 0.00005 |
| Proteobacteria | Pseudomonadales | Psychrobacter | Psychrobacter sp. TB47 | 0.00043 |
| Proteobacteria | Pseudomonadales | Psychrobacter | Psychrobacter sp. TB67 | 0.00112 |
| Proteobacteria | Pseudomonadales | Psychrobacter | Psychrobacter unclassified | 0.08313 |
| Proteobacteria | Pseudomonadales | Serpens | Serpens flexibilis | 0.00004 |
| Proteobacteria | Rhizobiales | Afipia | Afipia sp. P52-10 | 0.00003 |
| Proteobacteria | Rhizobiales | Agrobacterium | Agrobacterium tumefaciens | 0.00008 |
| Proteobacteria | Rhizobiales | Agrobacterium | Agrobacterium vitis | 0.00011 |
| Proteobacteria | Rhizobiales | Aurantimonas | Aurantimonas coralicida | 0.00002 |
| Proteobacteria | Rhizobiales | Aureimonas | Aureimonas altamirensis | 0.00001 |
| Proteobacteria | Rhizobiales | Aureimonas | Aureimonas ureilytica | 0.00002 |
| Proteobacteria | Rhizobiales | Bartonella | Bartonella grahamii | 0.00005 |
| Proteobacteria | Rhizobiales | Bartonella | Bartonella tamiae | 0.00008 |
| Proteobacteria | Rhizobiales | Bosea | Bosea sp. LC85 | 0.00002 |
| Proteobacteria | Rhizobiales | Bradyrhizobium | Bradyrhizobium elkanii | 0.00001 |
| Proteobacteria | Rhizobiales | Bradyrhizobium | Bradyrhizobium japonicum | 0.00009 |
| Proteobacteria | Rhizobiales | Bradyrhizobium | Bradyrhizobium sp. Ec3.3 | 0.00003 |
| Proteobacteria | Rhizobiales | Bradyrhizobium | Bradyrhizobium sp. ORS 278 | 0.00005 |
| Proteobacteria | Rhizobiales | Bradyrhizobium | Bradyrhizobium sp. S23321 | 0.00001 |
| Proteobacteria | Rhizobiales | Brucella | Brucella abortus | 0.00012 |
| Proteobacteria | Rhizobiales | Ensifer | Ensifer sojae | 0.00007 |
| Proteobacteria | Rhizobiales | Ensifer | Ensifer sp. ZNC0028 | 0.00007 |
| Proteobacteria | Rhizobiales | Hoeflea | Hoeflea sp. 108 | 0.00005 |
| Proteobacteria | Rhizobiales | Hyphomicrobium | Hyphomicrobium zavarzinii | 0.00003 |
| Proteobacteria | Rhizobiales | Kaistia | Kaistia adipata | 0.00006 |
| Proteobacteria | Rhizobiales | Mesorhizobium | Mesorhizobium sp. LSJC265A00 | 0.00007 |
| Proteobacteria | Rhizobiales | Mesorhizobium | Mesorhizobium sp. LSJC280B00 | 0.00007 |
| Proteobacteria | Rhizobiales | Mesorhizobium | Mesorhizobium sp. UASWS1009 | 0.00013 |
| Proteobacteria | Rhizobiales | Mesorhizobium | Mesorhizobium sp. URHA0056 | 0.00003 |
| Proteobacteria | Rhizobiales | Methylobacterium | Methylobacterium sp. 285MFTsu5.1 | 0.00023 |
| Proteobacteria | Rhizobiales | Methylocystis | Methylocystis sp. ATCC 49242 | 0.00002 |
| Proteobacteria | Rhizobiales | Methyloferula | Methyloferula stellata | 0.00003 |
| Proteobacteria | Rhizobiales | Methylosinus | Methylosinus sp. LW3 | 0.00003 |
| Proteobacteria | Rhizobiales | Microvirga | Microvirga flocculans | 0.00002 |
| Proteobacteria | Rhizobiales | Microvirga | Microvirga lotononidis | 0.00007 |
| Proteobacteria | Rhizobiales | Nitratireductor | Nitratireductor indicus | 0.00002 |
| Proteobacteria | Rhizobiales | Ochrobactrum | Ochrobactrum anthropi | 0.00003 |
| Proteobacteria | Rhizobiales | Ochrobactrum | Ochrobactrum sp. CDB2 | 0.00003 |
| Proteobacteria | Rhizobiales | Phyllobacterium | Phyllobacterium sp. YR531 | 0.00001 |
| Proteobacteria | Rhizobiales | Pseudaminobacter | Pseudaminobacter salicylatoxidans | 0.00004 |
| Proteobacteria | Rhizobiales | Pseudochrobactrum | Pseudochrobactrum sp. AO18b | 0.00012 |
| Proteobacteria | Rhizobiales | Rhizobiales unclassified | Rhizobiales unclassified | 0.00005 |
| Proteobacteria | Rhizobiales | Rhizobium | Rhizobium alamii | 0.00003 |
| Proteobacteria | Rhizobiales | Rhizobium | Rhizobium etli | 0.00006 |
| Proteobacteria | Rhizobiales | Rhizobium | Rhizobium grahamii | 0.00010 |
| Proteobacteria | Rhizobiales | Rhizobium | Rhizobium leguminosarum | 0.00007 |
| Proteobacteria | Rhizobiales | Rhizobium | Rhizobium sp. | 0.00288 |
| Proteobacteria | Rhizobiales | Rhizobium | Rhizobium sp. CF097 | 0.00002 |
| Proteobacteria | Rhizobiales | Rhodopseudomonas | Rhodopseudomonas palustris | 0.00002 |
| Proteobacteria | Rhizobiales | Sinorhizobium | Sinorhizobium fredii | 0.00005 |
| Proteobacteria | Rhizobiales | Sinorhizobium | Sinorhizobium meliloti | 0.00018 |
| Proteobacteria | Rhodobacterales | Celeribacter | Celeribacter indicus | 0.00001 |
| Proteobacteria | Rhodobacterales | Citreicella | Citreicella sp. 357 | 0.00001 |
| Proteobacteria | Rhodobacterales | Falsirhodobacter | Falsirhodobacter sp. alg1 | 0.00002 |
| Proteobacteria | Rhodobacterales | Haematobacter | Haematobacter massiliensis | 0.00001 |
| Proteobacteria | Rhodobacterales | Hyphomonas | Hyphomonas oceanitis | 0.00002 |
| Proteobacteria | Rhodobacterales | Hyphomonas | Hyphomonas sp. L-53-1-40 | 0.00001 |
| Proteobacteria | Rhodobacterales | Loktanella | Loktanella cinnabarina | 0.00002 |
| Proteobacteria | Rhodobacterales | Nesiotobacter | Nesiotobacter exalbescens | 0.00034 |
| Proteobacteria | Rhodobacterales | Paracoccus | Paracoccus aminophilus | 0.00003 |
| Proteobacteria | Rhodobacterales | Paracoccus | Paracoccus sp. 4681 | 0.00008 |
| Proteobacteria | Rhodobacterales | Paracoccus | Paracoccus sphaerophysae | 0.00002 |
| Proteobacteria | Rhodobacterales | Paracoccus | Paracoccus unclassified | 0.00003 |
| Proteobacteria | Rhodobacterales | Paracoccus | Paracoccus versutus | 0.00004 |
| Proteobacteria | Rhodobacterales | Paracoccus | Paracoccus yeei | 0.00004 |
| Proteobacteria | Rhodobacterales | Rhodobacter | Rhodobacter capsulatus | 0.00009 |
| Proteobacteria | Rhodobacterales | Rhodobacteraceae noname | Rhodobacteraceae bacterium HTCC2083 | 0.00001 |
| Proteobacteria | Rhodobacterales | Roseobacter | Roseobacter denitrificans | 0.00003 |
| Proteobacteria | Rhodobacterales | Roseobacter | Roseobacter sp. MED193 | 0.00002 |
| Proteobacteria | Rhodobacterales | Roseovarius | Roseovarius sp. MCTG156(2b) | 0.00002 |
| Proteobacteria | Rhodobacterales | Ruegeria | Ruegeria pomeroyi | 0.00003 |
| Proteobacteria | Rhodobacterales | Sulfitobacter | Sulfitobacter donghicola | 0.00002 |
| Proteobacteria | Rhodobacterales | Sulfitobacter | Sulfitobacter sp. CB2047 | 0.00010 |
| Proteobacteria | Rhodobacterales | Thioclava | Thioclava dalianensis | 0.00061 |
| Proteobacteria | Rhodocyclales | Aromatoleum | Aromatoleum aromaticum | 0.00004 |
| Proteobacteria | Rhodocyclales | Azoarcus | Azoarcus sp. BH72 | 0.00002 |
| Proteobacteria | Rhodocyclales | Azoarcus | Azoarcus sp. KH32C | 0.00001 |
| Proteobacteria | Rhodocyclales | Azoarcus | Azoarcus toluclasticus | 0.00006 |
| Proteobacteria | Rhodocyclales | Azospira | Azospira oryzae | 0.00003 |
| Proteobacteria | Rhodocyclales | Dechloromonas | Dechloromonas aromatica | 0.00002 |
| Proteobacteria | Rhodocyclales | Methyloversatilis | Methyloversatilis sp. NVD | 0.00011 |
| Proteobacteria | Rhodocyclales | Methyloversatilis | Methyloversatilis universalis | 0.00002 |
| Proteobacteria | Rhodocyclales | Sulfuritalea | Sulfuritalea hydrogenivorans | 0.00001 |
| Proteobacteria | Rhodocyclales | Thauera | Thauera phenylacetica | 0.00016 |
| Proteobacteria | Rhodocyclales | Thauera | Thauera sp. 28 | 0.00005 |
| Proteobacteria | Rhodocyclales | Thauera | Thauera sp. 63 | 0.00009 |
| Proteobacteria | Rhodocyclales | Thauera | Thauera unclassified | 0.00004 |
| Proteobacteria | Rhodocyclales | Uliginosibacterium | Uliginosibacterium gangwonense | 0.00007 |
| Proteobacteria | Rhodospirillales | Acetobacter | Acetobacter indonesiensis | 0.00001 |
| Proteobacteria | Rhodospirillales | Acetobacter | Acetobacter sp. CAG:267 | 0.00034 |
| Proteobacteria | Rhodospirillales | Acetobacteraceae noname | Acetobacteraceae bacterium AT-5844 | 0.00001 |
| Proteobacteria | Rhodospirillales | Acidiphilium | Acidiphilium sp. PM | 0.00003 |
| Proteobacteria | Rhodospirillales | Azospirillum | Azospirillum sp. CAG:239 | 0.00018 |
| Proteobacteria | Rhodospirillales | Azospirillum | Azospirillum sp. CAG:260 | 0.00004 |
| Proteobacteria | Rhodospirillales | Caenispirillum | Caenispirillum salinarum | 0.00001 |
| Proteobacteria | Rhodospirillales | Commensalibacter | Commensalibacter intestini | 0.00004 |
| Proteobacteria | Rhodospirillales | Commensalibacter | Commensalibacter sp. MX01 | 0.00010 |
| Proteobacteria | Rhodospirillales | Granulibacter | Granulibacter bethesdensis | 0.00001 |
| Proteobacteria | Rhodospirillales | Inquilinus | Inquilinus limosus | 0.00003 |
| Proteobacteria | Rhodospirillales | Magnetospirillum | Magnetospirillum gryphiswaldense | 0.00001 |
| Proteobacteria | Rhodospirillales | Novispirillum | Novispirillum itersonii | 0.00011 |
| Proteobacteria | Rhodospirillales | Oceanibaculum | Oceanibaculum indicum | 0.00005 |
| Proteobacteria | Rhodospirillales | Pararhodospirillum | Pararhodospirillum photometricum | 0.00006 |
| Proteobacteria | Rhodospirillales | Rhodospirillum | Rhodospirillum rubrum | 0.00007 |
| Proteobacteria | Rhodospirillales | Thalassospira | Thalassospira australica | 0.00001 |
| Proteobacteria | Rhodospirillales | Thalassospira | Thalassospira xiamenensis | 0.00003 |
| Proteobacteria | Rickettsiales | Candidatus Midichloria | Candidatus Midichloria mitochondrii | 0.00007 |
| Proteobacteria | Rickettsiales | Rickettsiaceae noname | Rickettsiaceae bacterium Os18 | 0.00006 |
| Proteobacteria | Rickettsiales | Rickettsiales noname | Rickettsiales bacterium Ac37b | 0.00003 |
| Proteobacteria | Sphingomonadales | Sphingobium | Sphingobium chlorophenolicum | 0.00006 |
| Proteobacteria | Sphingomonadales | Sphingobium | Sphingobium japonicum | 0.00002 |
| Proteobacteria | Sphingomonadales | Sphingobium | Sphingobium sp. ba1 | 0.00003 |
| Proteobacteria | Sphingomonadales | Sphingobium | Sphingobium sp. C100 | 0.00001 |
| Proteobacteria | Sphingomonadales | Sphingobium | Sphingobium sp. DC-2 | 0.00002 |
| Proteobacteria | Sphingomonadales | Sphingobium | Sphingobium sp. YBL2 | 0.00003 |
| Proteobacteria | Sphingomonadales | Sphingobium | Sphingobium yanoikuyae | 0.00053 |
| Proteobacteria | Sphingomonadales | Sphingomonadaceae unclassified | Sphingomonadaceae unclassified | 0.00003 |
| Proteobacteria | Sphingomonadales | Sphingomonas | Sphingomonas melonis | 0.00005 |
| Proteobacteria | Sphingomonadales | Sphingomonas | Sphingomonas paucimobilis | 0.00002 |
| Proteobacteria | Sphingomonadales | Sphingomonas | Sphingomonas sp. Ant20 | 0.00003 |
| Proteobacteria | Sphingomonadales | Sphingomonas | Sphingomonas sp. JGI 0001003-C6 | 0.00002 |
| Proteobacteria | Sphingomonadales | Sphingomonas | Sphingomonas sp. LH128 | 0.00003 |
| Proteobacteria | Sphingomonadales | Sphingomonas | Sphingomonas sp. S17 | 0.00001 |
| Proteobacteria | Sphingomonadales | Sphingomonas | Sphingomonas sp. SKA58 | 0.00001 |
| Proteobacteria | Sphingomonadales | Sphingomonas | Sphingomonas unclassified | 0.00008 |
| Proteobacteria | Sphingomonadales | Sphingopyxis | Sphingopyxis sp. Kp5.2 | 0.00001 |
| Proteobacteria | Sphingomonadales | Sphingopyxis | Sphingopyxis sp. MC1 | 0.00070 |
| Proteobacteria | Syntrophobacterales | Desulfomonile | Desulfomonile tiedjei | 0.00003 |
| Proteobacteria | Syntrophobacterales | Syntrophus | Syntrophus aciditrophicus | 0.00005 |
| Proteobacteria | Thiotrichales | Cycloclasticus | Cycloclasticus sp. P1 | 0.00004 |
| Proteobacteria | Thiotrichales | Fangia | Fangia hongkongensis | 0.00002 |
| Proteobacteria | Thiotrichales | Francisella | Francisella tularensis | 0.00017 |
| Proteobacteria | Thiotrichales | Leucothrix | Leucothrix mucor | 0.00005 |
| Proteobacteria | Thiotrichales | Methylophaga | Methylophaga aminisulfidivorans | 0.00001 |
| Proteobacteria | Thiotrichales | Methylophaga | Methylophaga thiooxydans | 0.00002 |
| Proteobacteria | Thiotrichales | Thioalkalimicrobium | Thioalkalimicrobium aerophilum | 0.00013 |
| Proteobacteria | Thiotrichales | Thioalkalimicrobium | Thioalkalimicrobium microaerophilum | 0.00001 |
| Proteobacteria | Thiotrichales | Thiomicrospira | Thiomicrospira chilensis | 0.00001 |
| Proteobacteria | Thiotrichales | Thiomicrospira | Thiomicrospira pelophila | 0.00001 |
| Proteobacteria | Thiotrichales | Thiomicrospira | Thiomicrospira sp. Milos-T2 | 0.00004 |
| Proteobacteria | Thiotrichales | Thioploca | Thioploca ingrica | 0.00007 |
| Proteobacteria | Vibrionales | Aliivibrio | Aliivibrio fischeri | 0.00036 |
| Proteobacteria | Vibrionales | Aliivibrio | Aliivibrio salmonicida | 0.00002 |
| Proteobacteria | Vibrionales | Enterovibrio | Enterovibrio calviensis | 0.00002 |
| Proteobacteria | Vibrionales | Enterovibrio | Enterovibrio norvegicus | 0.00003 |
| Proteobacteria | Vibrionales | Grimontia | Grimontia hollisae | 0.00009 |
| Proteobacteria | Vibrionales | Photobacterium | Photobacterium angustum | 0.00002 |
| Proteobacteria | Vibrionales | Photobacterium | Photobacterium damselae | 0.00001 |
| Proteobacteria | Vibrionales | Photobacterium | Photobacterium gaetbulicola | 0.00013 |
| Proteobacteria | Vibrionales | Photobacterium | Photobacterium halotolerans | 0.00009 |
| Proteobacteria | Vibrionales | Photobacterium | Photobacterium kishitanii | 0.00012 |
| Proteobacteria | Vibrionales | Photobacterium | Photobacterium leiognathi | 0.00005 |
| Proteobacteria | Vibrionales | Photobacterium | Photobacterium phosphoreum | 0.00716 |
| Proteobacteria | Vibrionales | Photobacterium | Photobacterium profundum | 0.00012 |
| Proteobacteria | Vibrionales | Photobacterium | Photobacterium sp. AK15 | 0.00002 |
| Proteobacteria | Vibrionales | Salinivibrio | Salinivibrio socompensis | 0.00002 |
| Proteobacteria | Vibrionales | Vibrio | Vibrio albensis | 0.00001 |
| Proteobacteria | Vibrionales | Vibrio | Vibrio anguillarum | 0.00002 |
| Proteobacteria | Vibrionales | Vibrio | Vibrio campbellii | 0.00016 |
| Proteobacteria | Vibrionales | Vibrio | Vibrio caribbeanicus | 0.00005 |
| Proteobacteria | Vibrionales | Vibrio | Vibrio cholerae | 0.00972 |
| Proteobacteria | Vibrionales | Vibrio | Vibrio coralliilyticus | 0.00002 |
| Proteobacteria | Vibrionales | Vibrio | Vibrio diazotrophicus | 0.00007 |
| Proteobacteria | Vibrionales | Vibrio | Vibrio fortis | 0.00006 |
| Proteobacteria | Vibrionales | Vibrio | Vibrio genomosp. F10 | 0.00008 |
| Proteobacteria | Vibrionales | Vibrio | Vibrio halioticoli | 0.00002 |
| Proteobacteria | Vibrionales | Vibrio | Vibrio harveyi | 0.00016 |
| Proteobacteria | Vibrionales | Vibrio | Vibrio ichthyoenteri | 0.00012 |
| Proteobacteria | Vibrionales | Vibrio | Vibrio kanaloae | 0.00001 |
| Proteobacteria | Vibrionales | Vibrio | Vibrio maritimus | 0.00009 |
| Proteobacteria | Vibrionales | Vibrio | Vibrio mimicus | 0.00027 |
| Proteobacteria | Vibrionales | Vibrio | Vibrio natriegens | 0.00001 |
| Proteobacteria | Vibrionales | Vibrio | Vibrio nigripulchritudo | 0.00005 |
| Proteobacteria | Vibrionales | Vibrio | Vibrio ordalii | 0.00009 |
| Proteobacteria | Vibrionales | Vibrio | Vibrio owensii | 0.00001 |
| Proteobacteria | Vibrionales | Vibrio | Vibrio parahaemolyticus | 0.00524 |
| Proteobacteria | Vibrionales | Vibrio | Vibrio ponticus | 0.00001 |
| Proteobacteria | Vibrionales | Vibrio | Vibrio proteolyticus | 0.00003 |
| Proteobacteria | Vibrionales | Vibrio | Vibrio rhizosphaerae | 0.00002 |
| Proteobacteria | Vibrionales | Vibrio | Vibrio rotiferianus | 0.00030 |
| Proteobacteria | Vibrionales | Vibrio | Vibrio rumoiensis | 0.00003 |
| Proteobacteria | Vibrionales | Vibrio | Vibrio scophthalmi | 0.00012 |
| Proteobacteria | Vibrionales | Vibrio | Vibrio shilonii | 0.00010 |
| Proteobacteria | Vibrionales | Vibrio | Vibrio sinaloensis | 0.00007 |
| Proteobacteria | Vibrionales | Vibrio | Vibrio sp. 090810a | 0.00001 |
| Proteobacteria | Vibrionales | Vibrio | Vibrio sp. C7 | 0.00003 |
| Proteobacteria | Vibrionales | Vibrio | Vibrio sp. ECSMB14106 | 0.00003 |
| Proteobacteria | Vibrionales | Vibrio | Vibrio sp. EJY3 | 0.00001 |
| Proteobacteria | Vibrionales | Vibrio | Vibrio sp. Ex25 | 0.00004 |
| Proteobacteria | Vibrionales | Vibrio | Vibrio sp. N418 | 0.00001 |
| Proteobacteria | Vibrionales | Vibrio | Vibrio sp. RC341 | 0.00002 |
| Proteobacteria | Vibrionales | Vibrio | Vibrio sp. S234-5 | 0.00001 |
| Proteobacteria | Vibrionales | Vibrio | Vibrio splendidus | 0.00007 |
| Proteobacteria | Vibrionales | Vibrio | Vibrio tubiashii | 0.00002 |
| Proteobacteria | Vibrionales | Vibrio | Vibrio unclassified | 0.00018 |
| Proteobacteria | Vibrionales | Vibrio | Vibrio vulnificus | 0.00023 |
| Proteobacteria | Xanthomonadales | Arenimonas | Arenimonas malthae | 0.00007 |
| Proteobacteria | Xanthomonadales | Dyella | Dyella japonica | 0.00002 |
| Proteobacteria | Xanthomonadales | Dyella | Dyella jiangningensis | 0.00004 |
| Proteobacteria | Xanthomonadales | Frateuria | Frateuria aurantia | 0.00015 |
| Proteobacteria | Xanthomonadales | Hydrocarboniphaga | Hydrocarboniphaga effusa | 0.00002 |
| Proteobacteria | Xanthomonadales | Ignatzschineria | Ignatzschineria larvae | 0.08677 |
| Proteobacteria | Xanthomonadales | Luteimonas | Luteimonas huabeiensis | 0.00001 |
| Proteobacteria | Xanthomonadales | Luteimonas | Luteimonas sp. J29 | 0.00004 |
| Proteobacteria | Xanthomonadales | Lysobacter | Lysobacter antibioticus | 0.00002 |
| Proteobacteria | Xanthomonadales | Lysobacter | Lysobacter daejeonensis | 0.00009 |
| Proteobacteria | Xanthomonadales | Lysobacter | Lysobacter defluvii | 0.00002 |
| Proteobacteria | Xanthomonadales | Mizugakiibacter | Mizugakiibacter sediminis | 0.00001 |
| Proteobacteria | Xanthomonadales | Oleiagrimonas | Oleiagrimonas soli | 0.00011 |
| Proteobacteria | Xanthomonadales | Polycyclovorans | Polycyclovorans algicola | 0.00002 |
| Proteobacteria | Xanthomonadales | Pseudoxanthomonas | Pseudoxanthomonas sp. GW2 | 0.00004 |
| Proteobacteria | Xanthomonadales | Pseudoxanthomonas | Pseudoxanthomonas suwonensis | 0.00009 |
| Proteobacteria | Xanthomonadales | Rhodanobacter | Rhodanobacter thiooxydans | 0.00005 |
| Proteobacteria | Xanthomonadales | Stenotrophomonas | Stenotrophomonas maltophilia | 0.00004 |
| Proteobacteria | Xanthomonadales | Stenotrophomonas | Stenotrophomonas sp. SKA14 | 0.00002 |
| Proteobacteria | Xanthomonadales | Stenotrophomonas | Stenotrophomonas sp. TA57 | 0.00006 |
| Proteobacteria | Xanthomonadales | Wohlfahrtiimonas | Wohlfahrtiimonas chitiniclastica | 0.00931 |
| Proteobacteria | Xanthomonadales | Xanthomonas | Xanthomonas arboricola | 0.00003 |
| Proteobacteria | Xanthomonadales | Xanthomonas | Xanthomonas axonopodis | 0.00002 |
| Proteobacteria | Xanthomonadales | Xanthomonas | Xanthomonas campestris | 0.00002 |
| Proteobacteria | Xanthomonadales | Xanthomonas | Xanthomonas oryzae | 0.00003 |
| Proteobacteria | Xanthomonadales | Xanthomonas | Xanthomonas unclassified | 0.00066 |
| Proteobacteria | Xanthomonadales | Xanthomonas | Xanthomonas vesicatoria | 0.00002 |
| Proteobacteria | Zetaproteobacteria noname | Zetaproteobacteria noname | zeta proteobacterium SCGC AB-137-I08 | 0.00003 |
| Proteobacteria | Zetaproteobacteria noname | Zetaproteobacteria noname | Zetaproteobacteria bacterium TAG-1 | 0.00002 |
| Spirochaetes | Brachyspirales | Brachyspira | Brachyspira alvinipulli | 0.00023 |
| Spirochaetes | Brachyspirales | Brachyspira | Brachyspira hampsonii | 0.00015 |
| Spirochaetes | Brachyspirales | Brachyspira | Brachyspira hyodysenteriae | 0.00007 |
| Spirochaetes | Brachyspirales | Brachyspira | Brachyspira innocens | 0.00005 |
| Spirochaetes | Brachyspirales | Brachyspira | Brachyspira intermedia | 0.00021 |
| Spirochaetes | Brachyspirales | Brachyspira | Brachyspira murdochii | 0.00020 |
| Spirochaetes | Brachyspirales | Brachyspira | Brachyspira pilosicoli | 0.00053 |
| Spirochaetes | Brachyspirales | Brachyspira | Brachyspira sp. CAG:700 | 0.00022 |
| Spirochaetes | Spirochaetales | Salinispira | Salinispira pacifica | 0.00002 |
| Spirochaetes | Spirochaetales | Sphaerochaeta | Sphaerochaeta coccoides | 0.00015 |
| Spirochaetes | Spirochaetales | Sphaerochaeta | Sphaerochaeta globosa | 0.00019 |
| Spirochaetes | Spirochaetales | Sphaerochaeta | Sphaerochaeta pleomorpha | 0.00014 |
| Spirochaetes | Spirochaetales | Spirochaeta | Spirochaeta bajacaliforniensis | 0.00243 |
| Spirochaetes | Spirochaetales | Treponema | Treponema azotonutricium | 0.00038 |
| Spirochaetes | Spirochaetales | Treponema | Treponema bryantii | 0.00004 |
| Spirochaetes | Spirochaetales | Treponema | Treponema caldaria | 0.00003 |
| Spirochaetes | Spirochaetales | Treponema | Treponema denticola | 0.00282 |
| Spirochaetes | Spirochaetales | Treponema | Treponema lecithinolyticum | 0.00023 |
| Spirochaetes | Spirochaetales | Treponema | Treponema maltophilum | 0.00035 |
| Spirochaetes | Spirochaetales | Treponema | Treponema medium | 0.00022 |
| Spirochaetes | Spirochaetales | Treponema | Treponema pedis | 0.00077 |
| Spirochaetes | Spirochaetales | Treponema | Treponema phagedenis | 0.00058 |
| Spirochaetes | Spirochaetales | Treponema | Treponema primitia | 0.00165 |
| Spirochaetes | Spirochaetales | Treponema | Treponema putidum | 0.00027 |
| Spirochaetes | Spirochaetales | Treponema | Treponema socranskii | 0.00065 |
| Spirochaetes | Spirochaetales | Treponema | Treponema sp. JC4 | 0.00003 |
| Spirochaetes | Spirochaetales | Treponema | Treponema sp. OMZ 838 | 0.00001 |
| Spirochaetes | Spirochaetales | Treponema | Treponema succinifaciens | 0.00008 |
| Spirochaetes | Spirochaetales | Treponema | Treponema vincentii | 0.00009 |
| Spirochaetes | Spirochaetia noname | Leptospira | Leptospira interrogans | 0.00011 |
| Synergistetes | Synergistales | Aminobacterium | Aminobacterium mobile | 0.00016 |
| Synergistetes | Synergistales | Anaerobaculum | Anaerobaculum mobile | 0.00004 |
| Synergistetes | Synergistales | Cloacibacillus | Cloacibacillus evryensis | 0.00467 |
| Synergistetes | Synergistales | Fretibacterium | Fretibacterium fastidiosum | 0.00752 |
| Synergistetes | Synergistales | Jonquetella | Jonquetella anthropi | 0.00013 |
| Synergistetes | Synergistales | Jonquetella | Jonquetella unclassified | 0.00007 |
| Synergistetes | Synergistales | Pyramidobacter | Pyramidobacter piscolens | 0.00091 |
| Synergistetes | Synergistales | Synergistes | Synergistes jonesii | 0.00507 |
| Synergistetes | Synergistales | Synergistes | Synergistes sp. 3 1 syn1 | 0.00098 |
| Tenericutes | Acholeplasmatales | Acholeplasma | Acholeplasma axanthum | 0.00009 |
| Tenericutes | Acholeplasmatales | Acholeplasma | Acholeplasma equifetale | 0.00015 |
| Tenericutes | Acholeplasmatales | Acholeplasma | Acholeplasma granularum | 0.00013 |
| Tenericutes | Acholeplasmatales | Acholeplasma | Acholeplasma hippikon | 0.00006 |
| Tenericutes | Acholeplasmatales | Acholeplasma | Acholeplasma laidlawii | 0.00005 |
| Tenericutes | Acholeplasmatales | Acholeplasma | Acholeplasma palmae | 0.00008 |
| Tenericutes | Acholeplasmatales | Acholeplasma | Acholeplasma sp. CAG:878 | 0.00005 |
| Tenericutes | Acholeplasmatales | Candidatus Phytoplasma | Candidatus Phytoplasma solani | 0.00003 |
| Tenericutes | Acholeplasmatales | Candidatus Phytoplasma | Chrysanthemum coronarium' phytoplasma | 0.00005 |
| Tenericutes | Acholeplasmatales | Candidatus Phytoplasma | Chrysanthemum yellows phytoplasma | 0.00006 |
| Tenericutes | Acholeplasmatales | Candidatus Phytoplasma | Rehmannia glutinosa' phytoplasma | 0.00001 |
| Tenericutes | Mollicutes noname | Mollicutes noname | Mollicutes bacterium HR1 | 0.00021 |
| Tenericutes | Mycoplasmatales | Mycoplasma | Mycoplasma agalactiae | 0.00002 |
| Tenericutes | Mycoplasmatales | Mycoplasma | Mycoplasma arginini | 0.00001 |
| Tenericutes | Mycoplasmatales | Mycoplasma | Mycoplasma bovigenitalium | 0.00007 |
| Tenericutes | Mycoplasmatales | Mycoplasma | Mycoplasma bovoculi | 0.00001 |
| Tenericutes | Mycoplasmatales | Mycoplasma | Mycoplasma buteonis | 0.00005 |
| Tenericutes | Mycoplasmatales | Mycoplasma | Mycoplasma californicum | 0.00051 |
| Tenericutes | Mycoplasmatales | Mycoplasma | Mycoplasma canis | 0.00008 |
| Tenericutes | Mycoplasmatales | Mycoplasma | Mycoplasma cynos | 0.00009 |
| Tenericutes | Mycoplasmatales | Mycoplasma | Mycoplasma dispar | 0.00001 |
| Tenericutes | Mycoplasmatales | Mycoplasma | Mycoplasma felis | 0.00001 |
| Tenericutes | Mycoplasmatales | Mycoplasma | Mycoplasma gallinarum | 0.00022 |
| Tenericutes | Mycoplasmatales | Mycoplasma | Mycoplasma gallisepticum | 0.00009 |
| Tenericutes | Mycoplasmatales | Mycoplasma | Mycoplasma hominis | 0.00205 |
| Tenericutes | Mycoplasmatales | Mycoplasma | Mycoplasma hyosynoviae | 0.00017 |
| Tenericutes | Mycoplasmatales | Mycoplasma | Mycoplasma leonicaptivi | 0.00002 |
| Tenericutes | Mycoplasmatales | Mycoplasma | Mycoplasma meleagridis | 0.00003 |
| Tenericutes | Mycoplasmatales | Mycoplasma | Mycoplasma opalescens | 0.00135 |
| Tenericutes | Mycoplasmatales | Mycoplasma | Mycoplasma ovipneumoniae | 0.00313 |
| Tenericutes | Mycoplasmatales | Mycoplasma | Mycoplasma putrefaciens | 0.00003 |
| Tenericutes | Mycoplasmatales | Mycoplasma | Mycoplasma simbae | 0.00030 |
| Tenericutes | Mycoplasmatales | Mycoplasma | Mycoplasma sp. CAG:472 | 0.00001 |
| Tenericutes | Mycoplasmatales | Mycoplasma | Mycoplasma sp. CAG:611 | 0.00003 |
| Tenericutes | Mycoplasmatales | Mycoplasma | Mycoplasma sp. CAG:776 | 0.00609 |
| Tenericutes | Mycoplasmatales | Mycoplasma | Mycoplasma sp. CAG:877 | 0.00059 |
| Tenericutes | Mycoplasmatales | Mycoplasma | Mycoplasma sp. G5847 | 0.00015 |
| Tenericutes | Mycoplasmatales | Ureaplasma | Ureaplasma canigenitalium | 0.00002 |
| Tenericutes | Mycoplasmatales | Ureaplasma | Ureaplasma diversum | 0.00006 |
| Tenericutes | Mycoplasmatales | Ureaplasma | Ureaplasma urealyticum | 0.00041 |
| Thermodesulfobacteria | Thermodesulfobacteriales | Thermodesulfobacterium | Thermodesulfobacterium hydrogeniphilum | 0.00062 |
| Thermotogae | Kosmotogales | Kosmotoga | Kosmotoga olearia | 0.00015 |
| Thermotogae | Kosmotogales | Mesotoga | Mesotoga infera | 0.00018 |
| Thermotogae | Petrotogales | Defluviitoga | Defluviitoga tunisiensis | 0.00002 |
| Thermotogae | Petrotogales | Marinitoga | Marinitoga piezophila | 0.00004 |
| Thermotogae | Petrotogales | Petrotoga | Petrotoga mobilis | 0.00002 |
| Thermotogae | Thermotogales | Fervidobacterium | Fervidobacterium islandicum | 0.00004 |
| Thermotogae | Thermotogales | Fervidobacterium | Fervidobacterium nodosum | 0.00002 |
| Verrucomicrobia | Opitutales | Opitutus | Opitutus terrae | 0.00003 |
| Verrucomicrobia | Puniceicoccales | Coraliomargarita | Coraliomargarita sp. CAG:312 | 0.00001 |
| Verrucomicrobia | Verrucomicrobia noname | Verrucomicrobia noname | Verrucomicrobia bacterium SCGC AAA164-I21 | 0.00001 |
| Verrucomicrobia | Verrucomicrobia noname | Verrucomicrobia noname | Verrucomicrobia bacterium SCGC AAA164-O14 | 0.00001 |
| Verrucomicrobia | Verrucomicrobiales | Akkermansia | Akkermansia muciniphila | 0.00004 |
| Verrucomicrobia | Verrucomicrobiales | Verrucomicrobiales noname | uncultured Verrucomicrobiales bacterium HF0010 05E02 | 0.00004 |
| Verrucomicrobia | Verrucomicrobiales | Verrucomicrobium | Verrucomicrobium sp. BvORR106 | 0.00005 |

* Percentage of sequences identified in metagenome of Amur tiger.
