# Supplementary material for: Metagenomic analysis of captive Amur tiger faecal microbiome
Source: BMC Vet Res. 2018 Dec 4;14:379. doi: 10.1186/s12917-018-1696-5 (PMC6278063; doi:10.1186/s12917-018-1696-5)
Supplement: Supplementary file 3 — Phylogenetic classification of Eukaryota in the Amur tiger metagenome. (DOCX 22 kb) [file 12917_2018_1696_MOESM3_ESM.docx]

**Additional file 3: Phylogenetic classification of Eukaryota in the Amur tiger metagenome.**

| Phylum | Class | Order | Genus | Species | ptg*  (%) |
| --- | --- | --- | --- | --- | --- |
| Ascomycota | Eurotiomycetes | Chaetothyriales | Exophiala | Exophiala oligosperma | 0.00003 |
|  |  | Eurotiales | Aspergillus | Aspergillus flavus | 0.00006 |
|  |  | Onygenales | Paracoccidioides | Paracoccidioides sp. 'lutzii' | 0.00005 |
|  |  |  | Trichophyton | Trichophyton tonsurans | 0.01007 |
|  | Leotiomycetes | Leotiomycetes noname | Pseudogymnoascus | Pseudogymnoascus pannorum | 0.00205 |
|  | Orbiliomycetes | Orbiliales | Orbilia | Arthrobotrys oligospora | 0.00003 |
|  | Saccharomycetes | Saccharomycetales | Cyberlindnera | Cyberlindnera fabianii | 0.00001 |
|  |  |  | Debaryomyces | Debaryomyces hansenii | 0.00004 |
|  |  |  | Kuraishia | Kuraishia capsulata | 0.00003 |
|  | Sordariomycetes | Hypocreales | Beauveria | Beauveria bassiana | 0.00002 |
| Basidiomycota | Agaricomycetes | Agaricales | Fistulina | Fistulina hepatica | 0.00002 |
|  |  |  | Moniliophthora | Moniliophthora perniciosa | 0.00005 |
|  |  |  | Pleurotus | Pleurotus ostreatus | 0.00004 |
|  |  |  | Schizophyllum | Schizophyllum commune | 0.00001 |
|  |  | Boletales | Pisolithus | Pisolithus microcarpus | 0.00002 |
|  |  | Polyporales | Gelatoporia | Gelatoporia subvermispora | 0.00001 |
|  |  | Sebacinales | Serendipita | Serendipita vermifera | 0.00009 |
|  | Exobasidiomycetes | Georgefischeriales | Tilletiaria | Tilletiaria anomala | 0.00005 |
|  | Malasseziomycetes | Malasseziales | Malassezia | Malassezia sympodialis | 0.00002 |
|  | Pucciniomycetes | Pucciniales | Puccinia | Puccinia graminis | 0.00008 |
|  | Tremellomycetes | Tremellales | Filobasidiella | Cryptococcus gattii | 0.00002 |
|  | Ustilaginomycetes | Ustilaginales | Pseudozyma | Pseudozyma brasiliensis | 0.00004 |
|  |  |  |  | Pseudozyma flocculosa | 0.00004 |
|  |  |  | Sporisorium | Sporisorium reilianum | 0.00006 |
|  |  |  | Ustilago | Ustilago maydis | 0.00025 |
| Blastocladiomycota | Blastocladiomycetes | Blastocladiales | Blastocladiella | Blastocladiella emersonii | 0.00009 |
| Chytridiomycota | Chytridiomycetes | Rhizophydiales | Batrachochytrium | Batrachochytrium dendrobatidis | 0.00002 |
| Cryptomycota | Cryptomycota noname | Cryptomycota noname | Rozella | Rozella allomycis | 0.00008 |
| Eukaryota noname | Eukaryota noname | Kickxellales | Spiromyces | Spiromyces aspiralis | 0.00003 |
|  |  | Mortierellales | Mortierella | Mortierella verticillata | 0.00003 |
|  |  | Mucorales | Absidia | Absidia idahoensis | 0.00003 |
|  |  |  | Lichtheimia | Lichtheimia corymbifera | 0.00008 |
|  |  |  | Mucor | Mucor ambiguus | 0.00004 |
|  |  |  | Parasitella | Parasitella parasitica | 0.00003 |
|  |  |  | Rhizopus | Rhizopus microsporus | 0.00015 |
| Glomeromycota | Glomeromycetes | Glomerales | Rhizophagus | Rhizophagus irregularis | 0.00026 |
| Microsporidia | Microsporidia noname | Microsporidia noname | Mitosporidium | Mitosporidium daphniae | 0.00008 |

* Percentage of sequences identified in metagenome of Amur tiger.
