# Supplementary material for: Metagenomic analysis of captive Amur tiger faecal microbiome
Source: BMC Vet Res. 2018 Dec 4;14:379. doi: 10.1186/s12917-018-1696-5 (PMC6278063; doi:10.1186/s12917-018-1696-5)
Supplement: Supplementary file 4 — Phylogenetic classification of Archaea in the Amur tiger metagenome. (DOCX 21 kb) [file 12917_2018_1696_MOESM4_ESM.docx]

**Additional file 4: Phylogenetic classification of Archaea in the Amur tiger metagenome.**

| Phylum | Class | Order | Genus | Species | ptg*  (%) |
| --- | --- | --- | --- | --- | --- |
| Archaea noname | Archaea noname | Archaea noname | Archaea noname | archaeon GW2011 AR15 | 0.00004 |
|  |  |  |  | archaeon GW2011 AR4 | 0.00012 |
| Crenarchaeota | Thermoprotei | Desulfurococcales | Desulfurococcus | Desulfurococcus kamchatkensis | 0.00003 |
| Euryarchaeota | Methanobacteria | Methanobacteriales | Methanobacterium | Methanobacterium paludis | 0.00001 |
|  |  |  | Methanobrevibacter | Methanobrevibacter boviskoreani | 0.00006 |
|  |  |  |  | Methanobrevibacter oralis | 0.00007 |
|  |  |  |  | Methanobrevibacter ruminantium | 0.00002 |
|  | Methanococci | Methanococcales | Methanocaldococcus | Methanocaldococcus sp. FS406-22 | 0.00002 |
|  | Methanomicrobia | Methanomicrobiales | Methanocorpusculum | Methanocorpusculum bavaricum | 0.00002 |
|  |  |  | Methanoculleus | Methanoculleus marisnigri | 0.00003 |
|  |  |  | Methanomicrobium | Methanomicrobium mobile | 0.00004 |
|  |  |  | Methanoplanus | Methanoplanus limicola | 0.00005 |
|  |  | Methanosarcinales | Methanococcoides | Methanococcoides methylutens | 0.00002 |
|  |  |  | Methanolobus | Methanolobus tindarius | 0.00001 |
|  |  |  | Methanomethylovorans | Methanomethylovorans hollandica | 0.00087 |
|  |  |  | Methanosaeta | Methanosaeta concilii | 0.00002 |
|  |  |  | Methanosarcina | Methanosarcina acetivorans | 0.00004 |
|  |  |  |  | Methanosarcina barkeri | 0.00002 |
|  |  |  |  | Methanosarcina lacustris | 0.00002 |
|  |  |  |  | Methanosarcina mazei | 0.00002 |
|  |  |  |  | Methanosarcina siciliae | 0.00007 |
|  |  |  |  | Methanosarcina sp. MTP4 | 0.00003 |
|  |  |  |  | Methanosarcina sp. WH1 | 0.00097 |
|  |  |  |  | Methanosarcina thermophila | 0.00004 |
|  | Thermococci | Thermococcales | Palaeococcus | Palaeococcus pacificus | 0.00009 |
|  | Thermoplasmata | Methanomassiliicoccales | Candidatus Methanoplasma | Candidatus Methanoplasma termitum | 0.00006 |
|  |  |  | Methanomassiliicoccus | Candidatus Methanomassiliicoccus intestinalis | 0.00002 |
|  |  | Thermoplasmatales | Thermoplasma | Thermoplasma acidophilum | 0.00006 |
|  |  |  |  | uncultured Thermoplasma sp. | 0.00004 |
| Thaumarchaeota | Thaumarchaeota noname | Nitrosopumilales | Nitrosopumilus | Nitrosopumilus unclassified | 0.00002 |

* Percentage of sequences identified in metagenome of Amur tiger.
