# Supplementary material for: Metagenomic analysis of captive Amur tiger faecal microbiome
Source: BMC Vet Res. 2018 Dec 4;14:379. doi: 10.1186/s12917-018-1696-5 (PMC6278063; doi:10.1186/s12917-018-1696-5)
Supplement: Supplementary file 5 — Phylogenetic classification of the viruses in the Amur tiger metagenome. (DOCX 43 kb) [file 12917_2018_1696_MOESM5_ESM.docx]

**Additional file 5: Phylogenetic classification of viruses in the Amur tiger metagenome.**

| Phylum | Order | Family | Species | ptg*  (%) |
| --- | --- | --- | --- | --- |
| Viruses noname | Caudovirales | Caudovirales noname | Bacillus virus 1 | 0.00049 |
|  |  | Myoviridae | Acinetobacter bacteriophage AP22 | 0.00040 |
|  |  |  | Acinetobacter phage vB AbaM Acibel004 | 0.00011 |
|  |  |  | Acinetobacter phage YMC13/03/R2096 | 0.00008 |
|  |  |  | Aeromonas phage vB AsaM-56 | 0.00007 |
|  |  |  | Aggregatibacter phage S1249 | 0.00002 |
|  |  |  | Bacillus phage BCD7 | 0.00065 |
|  |  |  | Bacillus phage BCP78 | 0.00009 |
|  |  |  | Bacillus phage BCP8-2 | 0.00010 |
|  |  |  | Brochothrix phage A9 | 0.00001 |
|  |  |  | Burkholderia phage Bcep781 | 0.00002 |
|  |  |  | Clostridium phage c-st | 0.00013 |
|  |  |  | Clostridium phage phiCD119 | 0.00017 |
|  |  |  | Clostridium phage phiCD27 | 0.00020 |
|  |  |  | Clostridium phage phiCDHM13 | 0.00002 |
|  |  |  | Edwardsiella phage GF-2 | 0.00006 |
|  |  |  | Enterobacteria phage JSE | 0.00009 |
|  |  |  | Enterobacteria phage P1 | 0.00778 |
|  |  |  | Enterobacteria phage P2 | 0.00457 |
|  |  |  | Enterobacteria phage phiP27 | 0.00017 |
|  |  |  | Enterobacteria phage PsP3 | 0.00023 |
|  |  |  | Enterobacteria phage SfV | 0.00057 |
|  |  |  | Enterobacteria phage vB EcoM-FV3 | 0.01386 |
|  |  |  | Escherichia phage 2 JES-2013 | 0.00235 |
|  |  |  | Escherichia phage Av-05 | 0.00017 |
|  |  |  | Escherichia phage Cba120 | 0.00002 |
|  |  |  | Escherichia phage RCS47 | 0.00100 |
|  |  |  | Escherichia phage rv5 | 0.00194 |
|  |  |  | Escherichia phage vB EcoM FFH2 | 0.00118 |
|  |  |  | Haemophilus phage Aaphi23 | 0.00002 |
|  |  |  | Lactobacillus phage phiAQ113 | 0.00032 |
|  |  |  | Paenibacillus phage Jimmer2 | 0.00020 |
|  |  |  | Prochlorococcus phage P-SSM2 | 0.00003 |
|  |  |  | Shigella phage SfII | 0.00002 |
|  |  |  | Stenotrophomonas phage Smp14 | 0.00002 |
|  |  |  | Synechococcus phage S-RSM4 | 0.00001 |
|  |  |  | Synechococcus phage syn9 | 0.00002 |
|  |  |  | Vibrio phage vB VpaM MAR | 0.00001 |
|  |  |  | Yersinia phage phiR1-37 | 0.00003 |
|  |  |  | Yersinia phage PY100 | 0.00010 |
|  |  | Podoviridae | Bacillus phage B103 | 0.00186 |
|  |  |  | Bacillus phage MG-B1 | 0.00031 |
|  |  |  | Bacillus phage phi29 | 0.00058 |
|  |  |  | Brucella phage Tb | 0.00002 |
|  |  |  | Cellulophaga phage phi14:2 | 0.00001 |
|  |  |  | Cellulophaga phage phi4:1 | 0.00014 |
|  |  |  | Citrobacter phage CR44b | 0.00004 |
|  |  |  | Citrobacter phage phiCFP-1 | 0.00004 |
|  |  |  | Clostridium phage CpV1 | 0.03265 |
|  |  |  | Clostridium phage phi24R | 0.00638 |
|  |  |  | Clostridium phage phiCP7R | 0.67098 |
|  |  |  | Clostridium phage phiCPV4 | 0.21035 |
|  |  |  | Clostridium phage phiZP2 | 0.83736 |
|  |  |  | Cronobacter phage Dev2 | 0.00004 |
|  |  |  | Enterobacter phage E-2 | 0.00004 |
|  |  |  | Enterobacter phage IME11 | 0.00041 |
|  |  |  | Enterobacteria phage 13a | 0.00024 |
|  |  |  | Enterobacteria phage Bp4 | 0.00153 |
|  |  |  | Enterobacteria phage EcoDS1 | 0.00019 |
|  |  |  | Enterobacteria phage IME10 | 0.00031 |
|  |  |  | Enterobacteria phage K1F | 0.00014 |
|  |  |  | Enterobacteria phage Sf101 | 0.00004 |
|  |  |  | Enterobacteria phage T7 | 0.00064 |
|  |  |  | Escherichia phage CICC 80001 | 0.00080 |
|  |  |  | Escherichia phage EC1-UPM | 0.00045 |
|  |  |  | Escherichia phage ECBP1 | 0.00277 |
|  |  |  | Escherichia phage N4 | 0.00025 |
|  |  |  | Escherichia phage PE3-1 | 0.00004 |
|  |  |  | Escherichia phage phiV10 | 0.00106 |
|  |  |  | Escherichia phage vB EcoP G7C | 0.00032 |
|  |  |  | Escherichia phage vB EcoP PhAPEC5 | 0.00007 |
|  |  |  | Escherichia phage vB EcoP PhAPEC7 | 0.00027 |
|  |  |  | Phage PhiI | 0.00006 |
|  |  |  | Ralstonia phage RSB3 | 0.00028 |
|  |  |  | Salmonella phage HK620 | 0.00003 |
|  |  |  | Salmonella phage SPN9CC | 0.00031 |
|  |  |  | Shigella phage pSb-1 | 0.00042 |
|  |  |  | Shigella phage Sf6 | 0.00044 |
|  |  |  | Yersinia phage phiA1122 | 0.00036 |
|  |  |  | Yersinia phage phiYeO3-12 | 0.00002 |
|  |  | Siphoviridae | Bacillus phage BCJA1c | 0.00110 |
|  |  |  | Bacteroides phage B124-14 | 0.00217 |
|  |  |  | Bacteroides phage B40-8 | 0.00084 |
|  |  |  | Burkholderia phage BcepGomr | 0.00005 |
|  |  |  | Cellulophaga phage phi12:1 | 0.00001 |
|  |  |  | Cellulophaga phage phi19:1 | 0.00018 |
|  |  |  | Clostridium phage phi3626 | 0.00019 |
|  |  |  | Clostridium phage phi8074-B1 | 0.00009 |
|  |  |  | Clostridium phage phiCD146 | 0.00002 |
|  |  |  | Clostridium phage phiCD24-1 | 0.00020 |
|  |  |  | Clostridium phage phiCD6356 | 0.00006 |
|  |  |  | Clostridium phage phiCP13O | 0.00042 |
|  |  |  | Clostridium phage phiCP26F | 0.00021 |
|  |  |  | Clostridium phage phiCP34O | 0.00002 |
|  |  |  | Clostridium phage phiCP39-O | 0.00055 |
|  |  |  | Clostridium phage PhiS63 | 0.00169 |
|  |  |  | Clostridium phage vB CpeS-CP51 | 0.00628 |
|  |  |  | Cronobacter phage ESP2949-1 | 0.00018 |
|  |  |  | Enterobacteria phage cdtI | 0.00463 |
|  |  |  | Enterobacteria phage DT57C | 0.00008 |
|  |  |  | Enterobacteria phage EK99P-1 | 0.00018 |
|  |  |  | Enterobacteria phage JL1 | 0.00056 |
|  |  |  | Enterobacteria phage lambda | 0.00742 |
|  |  |  | Enterobacteria phage mEp235 | 0.00006 |
|  |  |  | Enterobacteria phage mEp237 | 0.00005 |
|  |  |  | Enterobacteria phage mEp460 | 0.01177 |
|  |  |  | Enterobacteria phage PA-2 | 0.00002 |
|  |  |  | Enterobacteria phage SSL2009a | 0.00099 |
|  |  |  | Enterobacteria phage vB EcoS ACG-M12 | 0.00010 |
|  |  |  | Enterobacterio phage phiKP26 | 0.00002 |
|  |  |  | Enterococcus phage AUEF3 | 0.00044 |
|  |  |  | Enterococcus phage EfaCPT1 | 0.00030 |
|  |  |  | Enterococcus phage EFAP-1 | 0.00004 |
|  |  |  | Enterococcus phage EFRM31 | 0.00049 |
|  |  |  | Enterococcus phage IME EF3 | 0.00040 |
|  |  |  | Enterococcus phage IME-EF4 | 0.00067 |
|  |  |  | Enterococcus phage IME-EFm1 | 0.00005 |
|  |  |  | Escherichia phage Akfv33 | 0.00042 |
|  |  |  | Escherichia phage Bf23 | 0.00002 |
|  |  |  | Escherichia phage EB49 | 0.00002 |
|  |  |  | Escherichia phage Eps7 | 0.00006 |
|  |  |  | Escherichia phage HK578 | 0.00040 |
|  |  |  | Escherichia phage K1-dep(1) | 0.00101 |
|  |  |  | Escherichia phage K1-dep(4) | 0.00231 |
|  |  |  | Escherichia phage K1-ind(1) | 0.00081 |
|  |  |  | Escherichia phage K1-ind(2) | 0.00025 |
|  |  |  | Escherichia phage K1-ind(3) | 0.00170 |
|  |  |  | Escherichia phage RES-2009a | 0.00003 |
|  |  |  | Escherichia phage Rtp | 0.00008 |
|  |  |  | Escherichia phage T5 | 0.00026 |
|  |  |  | Escherichia phage Tls | 0.00024 |
|  |  |  | Escherichia phage vB EcoS FFH1 | 0.00028 |
|  |  |  | Escherichia Stx1 converting phage | 0.00011 |
|  |  |  | Flavobacterium phage 11b | 0.00009 |
|  |  |  | Klebsiella phage 1513 | 0.00037 |
|  |  |  | Lactobacillus phage c5 | 0.00025 |
|  |  |  | Lactobacillus phage Ld25A | 0.00012 |
|  |  |  | Lactobacillus phage phiLdb | 0.00108 |
|  |  |  | Lactococcus phage 936 sensu lato | 0.00036 |
|  |  |  | Lactococcus phage P335 sensu lato | 0.00044 |
|  |  |  | Lactococcus phage phiLC3 | 0.00002 |
|  |  |  | Lactococcus phage WRP3 | 0.00004 |
|  |  |  | Listeria phage LP-037 | 0.00215 |
|  |  |  | Mycobacterium phage HelDan | 0.00006 |
|  |  |  | Pseudomonas phage vB PaeS PAO1 Ab18 | 0.00004 |
|  |  |  | Psychrobacter phage Psymv2 | 0.00002 |
|  |  |  | Rhodococcus phage ReqiPepy6 | 0.00149 |
|  |  |  | Rhodococcus phage ReqiPoco6 | 0.00110 |
|  |  |  | Salmonella phage 5 | 0.00002 |
|  |  |  | Salmonella phage 9NA | 0.00035 |
|  |  |  | Salmonella phage FSL SP-031 | 0.00002 |
|  |  |  | Salmonella phage FSL SP-049 | 0.00024 |
|  |  |  | Salmonella phage FSL SP-062 | 0.00049 |
|  |  |  | Salmonella phage Jersey | 0.00050 |
|  |  |  | Salmonella phage L13 | 0.00015 |
|  |  |  | Salmonella phage LSPA1 | 0.00071 |
|  |  |  | Salmonella phage SETP13 | 0.00020 |
|  |  |  | Salmonella phage Shivani | 0.00023 |
|  |  |  | Salmonella phage Spc35 | 0.00066 |
|  |  |  | Salmonella phage Stitch | 0.00004 |
|  |  |  | Salmonella phage vB SenS AG11 | 0.00032 |
|  |  |  | Salmonella phage Vi II-E1 | 0.00400 |
|  |  |  | Shigella phage EP23 | 0.00057 |
|  |  |  | Siphoviridae phage YD-2008.s | 0.00030 |
|  |  |  | Siphovirus contig89 | 0.00014 |
|  |  |  | Sodalis phage SO1 | 0.00054 |
|  |  |  | Staphylococcus phage 6ec | 0.00003 |
|  |  |  | Streptococcus phage 5093 | 0.00005 |
|  |  |  | Streptococcus phage Alq132 | 0.00036 |
|  |  |  | Streptococcus phage PH15 | 0.00007 |
|  |  |  | Streptococcus phage SM1 | 0.00004 |
|  |  |  | Streptococcus phage SMP | 0.00007 |
|  |  |  | Streptococcus phage TP-778L | 0.00025 |
|  |  |  | Yersinia phage phiR201 | 0.00003 |
|  | Herpesvirales | Herpesviridae | Murid herpesvirus 1 | 0.00002 |
|  | Viruses noname | Circoviridae | Duck circovirus | 0.00028 |
|  |  | Microviridae | Chlamydia pneumoniae phage CPAR39 | 0.00003 |
|  |  | Mimiviridae | Megavirus courdo7 | 0.00005 |
|  |  | Parvoviridae | Blattella germanica densovirus-like virus | 0.00013 |
|  |  | Polyomaviridae | Raccoon polyomavirus | 0.00001 |
|  |  | Retroviridae | Avian leukosis virus | 0.00006 |
|  |  |  | Baboon endogenous virus | 0.00016 |
|  |  |  | Bat gammaretrovirus | 0.00031 |
|  |  |  | Feline endogenous virus | 0.00003 |
|  |  |  | Galidia ERV | 0.00003 |
|  |  |  | Human endogenous retrovirus | 0.00035 |
|  |  |  | Murine leukemia virus | 0.00003 |
|  |  |  | Simian retrovirus Y | 0.00002 |
|  |  | Viruses noname | Clostridium phage D-1873 | 0.00080 |
|  |  |  | Clostridium phage phiCT453B | 0.00002 |
|  |  |  | Clostridium phage phiCT9441A | 0.00010 |
|  |  |  | Clostridium phage phiSM101 | 0.00068 |
|  |  |  | Enterobacteria phage mEp332 | 0.00009 |
|  |  |  | Enterococcus phage BE2/ARG/2005 | 0.00003 |
|  |  |  | Escherichia phage P483 | 0.00005 |
|  |  |  | Staphylococcus phage phiIPLA-C1C | 0.00002 |
|  |  |  | Streptococcus phage phi30c | 0.00041 |
|  |  |  | Streptococcus phage phi5218 | 0.00003 |
|  |  |  | Streptococcus phage phiD12 | 0.00039 |
|  |  |  | Streptococcus phage phi-m46.1 | 0.00026 |
|  |  |  | Streptococcus phage Str-PAP-1 | 0.00030 |
|  |  |  | Streptococcus phi-m46.1-like phage MB56Spyo045 | 0.00025 |
|  |  |  | Sulfitobacter phage pCB2047-C | 0.00013 |
|  |  |  | uncultured Mediterranean phage uvMED | 0.00076 |
|  |  |  | uncultured phage crAssphage | 0.00037 |
|  |  |  | unidentified phage | 0.00109 |
|  |  |  | Vibrio phage pYD38-A | 0.00093 |

* Percentage of sequences identified in metagenome of Amur tiger.
