# Supplementary material for: Metagenomic analysis of captive Amur tiger faecal microbiome
Source: BMC Vet Res. 2018 Dec 4;14:379. doi: 10.1186/s12917-018-1696-5 (PMC6278063; doi:10.1186/s12917-018-1696-5)
Supplement: Supplementary file 6 — GO annotations of the Amur tiger faecal metagenome. (DOCX 683 kb) [file 12917_2018_1696_MOESM6_ESM.docx]

**Additional file 6: GO annotations of the Amur tiger fecal metagenome.**

| GO Term | GO Function | Count |
| --- | --- | --- |
| biological process | biological process | 14436 |
| biological process | transport | 5531 |
| biological process | regulation of transcription, DNA-templated | 5065 |
| biological process | metabolic process | 3025 |
| biological process | proteolysis | 2451 |
| biological process | oxidation-reduction process | 2442 |
| biological process | translation | 2397 |
| biological process | phosphorelay signal transduction system | 2229 |
| biological process | DNA recombination | 1873 |
| biological process | DNA repair | 1688 |
| biological process | carbohydrate metabolic process | 1675 |
| biological process | peptidoglycan biosynthetic process | 1639 |
| biological process | DNA replication | 1539 |
| biological process | growth | 1406 |
| biological process | transmembrane transport | 1320 |
| biological process | transcription, DNA-templated | 1054 |
| biological process | drug transmembrane transport | 986 |
| biological process | phosphoenolpyruvate-dependent sugar phosphotransferase system | 980 |
| biological process | phosphorylation | 943 |
| biological process | cellular response to DNA damage stimulus | 915 |
| biological process | asexual sporulation | 894 |
| biological process | carbohydrate transport | 802 |
| biological process | glycolytic process | 799 |
| biological process | DNA topological change | 750 |
| biological process | protein processing | 742 |
| biological process | DNA unwinding involved in DNA replication | 723 |
| biological process | polysaccharide biosynthetic process | 706 |
| biological process | transposition, DNA-mediated | 699 |
| biological process | fatty acid biosynthetic process | 660 |
| biological process | response to antibiotic | 633 |
| biological process | protein folding | 630 |
| biological process | lipopolysaccharide biosynthetic process | 624 |
| biological process | tRNA modification | 604 |
| biological process | peptide metabolic process | 581 |
| biological process | protein secretion | 558 |
| biological process | methylglyoxal catabolic process to D-lactate via S-lactoyl-glutathione | 533 |
| biological process | pathogenesis | 519 |
| biological process | amino acid transport | 518 |
| biological process | chromosome segregation | 518 |
| biological process | response to oxidative stress | 505 |
| biological process | tricarboxylic acid cycle | 486 |
| biological process | fermentation | 484 |
| biological process | thiamine biosynthetic process | 474 |
| biological process | purine ribonucleotide biosynthetic process | 463 |
| biological process | methylation | 453 |
| biological process | carbohydrate transmembrane transport | 452 |
| biological process | ATP synthesis coupled proton transport | 452 |
| biological process | ion transport | 448 |
| biological process | cobalamin biosynthetic process | 444 |
| biological process | negative regulation of transcription, DNA-templated | 437 |
| biological process | response to heat | 431 |
| biological process | chorismate biosynthetic process | 409 |
| biological process | enzyme-directed rRNA pseudouridine synthesis | 405 |
| biological process | DNA-templated transcription, initiation | 395 |
| biological process | nucleobase-containing small molecule interconversion | 394 |
| biological process | sodium ion transport | 379 |
| biological process | DNA integration | 379 |
| biological process | RNA secondary structure unwinding | 373 |
| biological process | DNA restriction-modification system | 373 |
| biological process | cell division | 372 |
| biological process | arginine biosynthetic process | 364 |
| biological process | cellular amino acid biosynthetic process | 360 |
| biological process | oligopeptide transport | 358 |
| biological process | gluconeogenesis | 349 |
| biological process | anaerobic respiration | 348 |
| biological process | double-strand break repair | 342 |
| biological process | pyrimidine ribonucleotide biosynthetic process | 336 |
| biological process | N-terminal protein amino acid acetylation | 334 |
| biological process | positive regulation of transcription, DNA-templated | 328 |
| biological process | potassium ion transport | 328 |
| biological process | iron ion transport | 326 |
| biological process | lipid metabolic process | 323 |
| biological process | porphyrin-containing compound biosynthetic process | 322 |
| biological process | RNA processing | 318 |
| biological process | signal transduction | 316 |
| biological process | cellular protein modification process | 315 |
| biological process | DNA methylation | 314 |
| biological process | DNA metabolic process | 313 |
| biological process | rRNA processing | 309 |
| biological process | small molecule biosynthetic process | 304 |
| biological process | ribosome biogenesis | 303 |
| biological process | DNA-dependent DNA replication | 302 |
| biological process | urea cycle | 296 |
| biological process | cell wall organization | 295 |
| biological process | mismatch repair | 294 |
| biological process | protein transport | 294 |
| biological process | fatty acid metabolic process | 293 |
| biological process | nucleic acid phosphodiester bond hydrolysis | 291 |
| biological process | lysine biosynthetic process via diaminopimelate | 287 |
| biological process | cellular amino acid metabolic process | 283 |
| biological process | regulation of cell shape | 282 |
| biological process | response to zinc ion | 280 |
| biological process | histidine biosynthetic process | 279 |
| biological process | DNA modification | 274 |
| biological process | biosynthetic process | 273 |
| biological process | phosphate ion transport | 270 |
| biological process | aromatic amino acid family biosynthetic process | 268 |
| biological process | pentose-phosphate shunt | 262 |
| biological process | response to drug | 262 |
| biological process | glycogen biosynthetic process | 260 |
| biological process | DNA replication initiation | 255 |
| biological process | pilus assembly | 255 |
| biological process | cell redox homeostasis | 254 |
| biological process | putrescine transport | 244 |
| biological process | virion assembly | 244 |
| biological process | protein phosphorylation | 243 |
| biological process | ribosomal large subunit assembly | 238 |
| biological process | macropinocytosis | 236 |
| biological process | cell adhesion | 235 |
| biological process | 'de novo' IMP biosynthetic process | 234 |
| biological process | proton transport | 233 |
| biological process | deoxyribonucleotide biosynthetic process | 232 |
| biological process | DNA catabolic process | 232 |
| biological process | tRNA threonylcarbamoyladenosine modification | 231 |
| biological process | folic acid-containing compound metabolic process | 231 |
| biological process | rRNA base methylation | 229 |
| biological process | one-carbon metabolic process | 227 |
| biological process | peptide transport | 226 |
| biological process | methionine biosynthetic process | 224 |
| biological process | lipopolysaccharide core region biosynthetic process | 220 |
| biological process | signal peptide processing | 216 |
| biological process | pantothenate biosynthetic process | 216 |
| biological process | nucleobase-containing compound metabolic process | 213 |
| biological process | glutamine metabolic process | 208 |
| biological process | base-excision repair | 208 |
| biological process | chemotaxis | 203 |
| biological process | aerobic respiration | 202 |
| biological process | phenylalanyl-tRNA aminoacylation | 202 |
| biological process | ribosomal small subunit assembly | 202 |
| biological process | protein tetramerization | 201 |
| biological process | translational initiation | 201 |
| biological process | translational elongation | 200 |
| biological process | tRNA aminoacylation for protein translation | 200 |
| biological process | mRNA catabolic process | 200 |
| biological process | queuosine biosynthetic process | 199 |
| biological process | protein catabolic process | 198 |
| biological process | riboflavin biosynthetic process | 197 |
| biological process | anion transmembrane transport | 197 |
| biological process | 2'-deoxyribonucleotide biosynthetic process | 192 |
| biological process | cellular response to oxidative stress | 191 |
| biological process | lipid A biosynthetic process | 187 |
| biological process | glycine metabolic process | 187 |
| biological process | phospholipid biosynthetic process | 186 |
| biological process | protein secretion by the type II secretion system | 186 |
| biological process | purine nucleotide biosynthetic process | 186 |
| biological process | RNA catabolic process | 186 |
| biological process | dephosphorylation | 185 |
| biological process | pyrimidine nucleobase metabolic process | 185 |
| biological process | glycerol metabolic process | 185 |
| biological process | tRNA methylthiolation | 184 |
| biological process | transposition | 184 |
| biological process | cation transmembrane transport | 183 |
| biological process | biotin biosynthetic process | 183 |
| biological process | nitrogen compound metabolic process | 182 |
| biological process | peptidoglycan catabolic process | 181 |
| biological process | response to starvation | 179 |
| biological process | viral DNA genome packaging | 178 |
| biological process | ferrous iron transport | 176 |
| biological process | sulfur compound metabolic process | 175 |
| biological process | NAD biosynthetic process | 174 |
| biological process | enzyme-directed rRNA 2'-O-methylation | 174 |
| biological process | spermidine transport | 172 |
| biological process | barrier septum assembly | 172 |
| biological process | O antigen biosynthetic process | 170 |
| biological process | C4-dicarboxylate transport | 170 |
| biological process | cation transport | 170 |
| biological process | peptidoglycan turnover | 170 |
| biological process | galactose metabolic process | 169 |
| biological process | guanosine tetraphosphate metabolic process | 168 |
| biological process | pyrimidine nucleoside salvage | 168 |
| biological process | isoleucine biosynthetic process | 167 |
| biological process | glutamyl-tRNA aminoacylation | 165 |
| biological process | tRNA processing | 163 |
| biological process | coenzyme A biosynthetic process | 163 |
| biological process | RNA modification | 163 |
| biological process | sulfate assimilation | 162 |
| biological process | cellular phosphate ion homeostasis | 161 |
| biological process | cobalt ion transport | 159 |
| biological process | regulation of carbohydrate utilization | 159 |
| biological process | proline biosynthetic process | 155 |
| biological process | chaperone-mediated protein folding | 153 |
| biological process | SOS response | 153 |
| biological process | branched-chain amino acid biosynthetic process | 151 |
| biological process | iron-sulfur cluster assembly | 150 |
| biological process | fructose 6-phosphate metabolic process | 149 |
| biological process | molybdopterin cofactor biosynthetic process | 148 |
| biological process | glycogen catabolic process | 147 |
| biological process | isoleucyl-tRNA aminoacylation | 147 |
| biological process | extracellular polysaccharide biosynthetic process | 144 |
| biological process | fatty acid beta-oxidation using acyl-CoA dehydrogenase | 144 |
| biological process | lipid homeostasis | 144 |
| biological process | amino acid transmembrane transport | 144 |
| biological process | response to toxic substance | 143 |
| biological process | glutamate biosynthetic process | 141 |
| biological process | UDP-N-acetylglucosamine metabolic process | 139 |
| biological process | purine nucleobase biosynthetic process | 139 |
| biological process | threonine biosynthetic process | 138 |
| biological process | cysteine biosynthetic process from serine | 136 |
| biological process | purine ribonucleoside salvage | 136 |
| biological process | alanyl-tRNA aminoacylation | 136 |
| biological process | provirus excision | 136 |
| biological process | RNA phosphodiester bond hydrolysis, endonucleolytic | 136 |
| biological process | protein autophosphorylation | 135 |
| biological process | cellular water homeostasis | 135 |
| biological process | DNA replication, synthesis of RNA primer | 135 |
| biological process | establishment of competence for transformation | 134 |
| biological process | response to stress | 133 |
| biological process | pentose-phosphate shunt, non-oxidative branch | 133 |
| biological process | valyl-tRNA aminoacylation | 132 |
| biological process | threonine catabolic process | 132 |
| biological process | regulation of translational fidelity | 132 |
| biological process | bacterial-type flagellum-dependent swarming motility | 131 |
| biological process | leucyl-tRNA aminoacylation | 131 |
| biological process | cell wall macromolecule catabolic process | 131 |
| biological process | DNA-templated transcription, termination | 130 |
| biological process | signal transduction by protein phosphorylation | 129 |
| biological process | peptidoglycan-based cell wall biogenesis | 129 |
| biological process | fucose metabolic process | 129 |
| biological process | ribonucleoside monophosphate biosynthetic process | 129 |
| biological process | DNA replication, removal of RNA primer | 128 |
| biological process | peptide catabolic process | 127 |
| biological process | regulation of sequestering of zinc ion | 127 |
| biological process | peptidyl-histidine phosphorylation | 127 |
| biological process | valine biosynthetic process | 126 |
| biological process | regulation of transcription from RNA polymerase II promoter | 126 |
| biological process | DNA synthesis involved in DNA repair | 126 |
| biological process | D-ribose transport | 126 |
| biological process | N-acetylglucosamine transport | 125 |
| biological process | cellular iron ion homeostasis | 124 |
| biological process | adenine biosynthetic process | 123 |
| biological process | pyridine nucleotide biosynthetic process | 123 |
| biological process | cardiolipin biosynthetic process | 122 |
| biological process | malate metabolic process | 122 |
| biological process | intracellular protein transport | 121 |
| biological process | protein metabolic process | 121 |
| biological process | DNA duplex unwinding | 120 |
| biological process | lipid biosynthetic process | 120 |
| biological process | response to ionizing radiation | 120 |
| biological process | pentose catabolic process | 119 |
| biological process | GMP biosynthetic process | 119 |
| biological process | single-species biofilm formation | 117 |
| biological process | isopentenyl diphosphate biosynthetic process, methylerythritol 4-phosphate pathway | 117 |
| biological process | dUTP catabolic process | 117 |
| biological process | pyridoxine biosynthetic process | 117 |
| biological process | methionyl-tRNA aminoacylation | 117 |
| biological process | D-ribose metabolic process | 116 |
| biological process | protein lipoylation | 116 |
| biological process | glycerol-3-phosphate transport | 116 |
| biological process | cellular response to hydrogen peroxide | 115 |
| biological process | regulation of nucleic acid-templated transcription | 114 |
| biological process | base-excision repair, DNA ligation | 113 |
| biological process | leucine biosynthetic process | 112 |
| biological process | dTDP-rhamnose biosynthetic process | 112 |
| biological process | DNA mediated transformation | 111 |
| biological process | tryptophan biosynthetic process | 111 |
| biological process | aspartate biosynthetic process | 111 |
| biological process | positive regulation of sporulation resulting in formation of a cellular spore | 110 |
| biological process | glycerol catabolic process | 110 |
| biological process | transcription antitermination | 110 |
| biological process | isoprenoid biosynthetic process | 109 |
| biological process | glucan catabolic process | 109 |
| biological process | UDP-glucuronate biosynthetic process | 108 |
| biological process | phosphorus metabolic process | 108 |
| biological process | fatty acid catabolic process | 108 |
| biological process | phosphate-containing compound metabolic process | 108 |
| biological process | aspartyl-tRNA aminoacylation | 108 |
| biological process | potassium ion transmembrane transport | 108 |
| biological process | cellular response to heat | 106 |
| biological process | dGTP catabolic process | 106 |
| biological process | rRNA methylation | 105 |
| biological process | response to cold | 105 |
| biological process | tRNA aminoacylation | 105 |
| biological process | cytochrome complex assembly | 105 |
| biological process | ion transmembrane transport | 105 |
| biological process | cytoplasmic translation | 104 |
| biological process | anion transport | 104 |
| biological process | nitrogen compound transport | 103 |
| biological process | N-acetylglucosamine catabolic process | 103 |
| biological process | 'de novo' pyrimidine nucleobase biosynthetic process | 103 |
| biological process | iron ion homeostasis | 103 |
| biological process | leucine transport | 102 |
| biological process | single-organism carbohydrate catabolic process | 102 |
| biological process | mitotic recombination | 102 |
| biological process | protein maturation by iron-sulfur cluster transfer | 101 |
| biological process | fatty acid elongation | 101 |
| biological process | RNA methylation | 101 |
| biological process | chromosome condensation | 101 |
| biological process | folic acid-containing compound biosynthetic process | 100 |
| biological process | cellobiose transport | 100 |
| biological process | glycosyl compound metabolic process | 99 |
| biological process | cysteinyl-tRNA aminoacylation | 99 |
| biological process | cellular response to phosphate starvation | 99 |
| biological process | arginyl-tRNA aminoacylation | 98 |
| biological process | nucleoside transport | 98 |
| biological process | phospholipid catabolic process | 97 |
| biological process | protein dephosphorylation | 97 |
| biological process | Mo-molybdopterin cofactor biosynthetic process | 96 |
| biological process | carbohydrate phosphorylation | 95 |
| biological process | maturation of SSU-rRNA from tricistronic rRNA transcript (SSU-rRNA, 5.8S rRNA, LSU-rRNA) | 95 |
| biological process | antibiotic transport | 95 |
| biological process | viral process | 95 |
| biological process | menaquinone biosynthetic process | 95 |
| biological process | cytokinesis | 94 |
| biological process | pilus organization | 94 |
| biological process | polysaccharide metabolic process | 94 |
| biological process | UDP-N-acetylglucosamine biosynthetic process | 94 |
| biological process | isoleucine transport | 93 |
| biological process | selenocysteine metabolic process | 93 |
| biological process | microtubule-based movement | 93 |
| biological process | response to hydrogen peroxide | 92 |
| biological process | fructose import | 92 |
| biological process | D-glucuronate catabolic process | 91 |
| biological process | purine nucleotide metabolic process | 91 |
| biological process | protein stabilization | 91 |
| biological process | galactose transport | 91 |
| biological process | N-terminal protein amino acid modification | 90 |
| biological process | strand invasion | 90 |
| biological process | prolyl-tRNA aminoacylation | 90 |
| biological process | histidyl-tRNA aminoacylation | 90 |
| biological process | DNA recombinase assembly | 89 |
| biological process | cytolysis | 89 |
| biological process | protein maturation | 88 |
| biological process | protein homotetramerization | 88 |
| biological process | L-serine biosynthetic process | 87 |
| biological process | glucose import into cell | 87 |
| biological process | GPI anchor biosynthetic process | 86 |
| biological process | co-translational protein modification | 86 |
| biological process | asymmetric cell division | 86 |
| biological process | hydrogen peroxide catabolic process | 86 |
| biological process | 10-formyltetrahydrofolate biosynthetic process | 86 |
| biological process | fructose transport | 85 |
| biological process | nucleotide-sugar metabolic process | 85 |
| biological process | xanthine catabolic process | 85 |
| biological process | [2Fe-2S] cluster assembly | 85 |
| biological process | tryptophanyl-tRNA aminoacylation | 84 |
| biological process | arginine metabolic process | 84 |
| biological process | N-acetylglucosamine metabolic process | 84 |
| biological process | protein N-linked glycosylation | 84 |
| biological process | drug transport | 84 |
| biological process | ubiquinone biosynthetic process | 84 |
| biological process | bacterial-type flagellum-dependent cell motility | 83 |
| biological process | mannose transport | 83 |
| biological process | seryl-tRNA aminoacylation | 82 |
| biological process | DNA-templated transcription, elongation | 82 |
| biological process | cellular carbohydrate metabolic process | 81 |
| biological process | glycerophosphodiester transport | 80 |
| biological process | branched-chain amino acid transport | 80 |
| biological process | viral entry into host cell | 80 |
| biological process | protein O-linked mannosylation | 79 |
| biological process | heme biosynthetic process | 79 |
| biological process | regulation of DNA repair | 79 |
| biological process | tRNA wobble guanine modification | 79 |
| biological process | homing of group II introns | 79 |
| biological process | pyruvate metabolic process | 78 |
| biological process | rhamnose catabolic process | 78 |
| biological process | acetyl-CoA biosynthetic process from pyruvate | 78 |
| biological process | recombinational repair | 78 |
| biological process | lipid translocation | 78 |
| biological process | reciprocal meiotic recombination | 78 |
| biological process | carbon utilization | 78 |
| biological process | dUMP biosynthetic process | 77 |
| biological process | protein repair | 77 |
| biological process | antibiotic biosynthetic process | 77 |
| biological process | L-glutamate transport | 77 |
| biological process | response to unfolded protein | 77 |
| biological process | carbohydrate catabolic process | 77 |
| biological process | regulation of translation | 76 |
| biological process | nucleotide-excision repair | 76 |
| biological process | L-fucose catabolic process | 75 |
| biological process | regulation of gene expression | 75 |
| biological process | cytokinin biosynthetic process | 75 |
| biological process | dolichol metabolic process | 75 |
| biological process | L-amino acid transport | 74 |
| biological process | D-methionine transport | 74 |
| biological process | rRNA modification | 74 |
| biological process | protein deglycosylation | 73 |
| biological process | positive regulation of translational fidelity | 73 |
| biological process | glucose transport | 73 |
| biological process | tRNA wobble position uridine thiolation | 73 |
| biological process | L-alpha-amino acid transmembrane transport | 73 |
| biological process | glycerophospholipid biosynthetic process | 72 |
| biological process | arginine deiminase pathway | 72 |
| biological process | removal of superoxide radicals | 72 |
| biological process | lysyl-tRNA aminoacylation | 72 |
| biological process | transcription elongation from RNA polymerase II promoter | 72 |
| biological process | magnesium ion transport | 72 |
| biological process | double-strand break repair via homologous recombination | 71 |
| biological process | tetrahydrofolate biosynthetic process | 71 |
| biological process | siderophore transport | 71 |
| biological process | diaminopimelate biosynthetic process | 71 |
| biological process | uracil transport | 71 |
| biological process | entry into host cell | 71 |
| biological process | chaperone mediated protein folding requiring cofactor | 70 |
| biological process | peptidoglycan metabolic process | 70 |
| biological process | FMN biosynthetic process | 69 |
| biological process | phosphatidylglycerol biosynthetic process | 69 |
| biological process | glucose metabolic process | 69 |
| biological process | protein N-linked glycosylation via asparagine | 69 |
| biological process | D-galacturonate catabolic process | 69 |
| biological process | maltose transport | 69 |
| biological process | purine nucleobase metabolic process | 68 |
| biological process | oligosaccharide catabolic process | 68 |
| biological process | maturation of LSU-rRNA | 68 |
| biological process | siroheme biosynthetic process | 68 |
| biological process | tetrahydrofolate interconversion | 68 |
| biological process | lipopolysaccharide transport | 68 |
| biological process | amino sugar metabolic process | 67 |
| biological process | arginine biosynthetic process via ornithine | 67 |
| biological process | nicotinate nucleotide salvage | 67 |
| biological process | glyceraldehyde-3-phosphate biosynthetic process | 67 |
| biological process | pseudouridine synthesis | 67 |
| biological process | cyclic threonylcarbamoyladenosine biosynthetic process | 66 |
| biological process | chitin catabolic process | 66 |
| biological process | colanic acid biosynthetic process | 66 |
| biological process | RNA (guanine-N7)-methylation | 65 |
| biological process | nitrate assimilation | 65 |
| biological process | spermidine biosynthetic process | 65 |
| biological process | oligosaccharide metabolic process | 65 |
| biological process | polyamine transport | 65 |
| biological process | aromatic compound catabolic process | 65 |
| biological process | shikimate metabolic process | 65 |
| biological process | fumarate metabolic process | 65 |
| biological process | CDP-diacylglycerol biosynthetic process | 64 |
| biological process | cytolysis by virus of host cell | 64 |
| biological process | glycine decarboxylation via glycine cleavage system | 64 |
| biological process | fucose catabolic process | 64 |
| biological process | mRNA pseudouridine synthesis | 64 |
| biological process | UDP-glucose metabolic process | 64 |
| biological process | mitochondrial translation | 64 |
| biological process | asparagine biosynthetic process | 64 |
| biological process | negative regulation of sporulation resulting in formation of a cellular spore | 64 |
| biological process | negative regulation of translation | 64 |
| biological process | regulation of sporulation | 63 |
| biological process | histidine catabolic process | 63 |
| biological process | response to bacterium | 63 |
| biological process | cellular response to cold | 63 |
| biological process | cellular calcium ion homeostasis | 63 |
| biological process | methionine metabolic process | 63 |
| biological process | purine nucleoside catabolic process | 62 |
| biological process | uracil catabolic process | 62 |
| biological process | hydrogen transport | 62 |
| biological process | nickel cation transmembrane transport | 62 |
| biological process | vacuolar acidification | 62 |
| biological process | isopentenyl diphosphate biosynthetic process, methylerythritol 4-phosphate pathway involved in terpenoid biosynthetic process | 62 |
| biological process | positive regulation of catalytic activity | 61 |
| biological process | metal ion transport | 61 |
| biological process | positive regulation of single-species biofilm formation | 61 |
| biological process | ethanolamine catabolic process | 60 |
| biological process | protein homooligomerization | 60 |
| biological process | stringent response | 60 |
| biological process | cytoplasmic translational termination | 59 |
| biological process | aerobic electron transport chain | 59 |
| biological process | tyrosine biosynthetic process | 59 |
| biological process | 'de novo' NAD biosynthetic process from aspartate | 59 |
| biological process | phosphate ion transmembrane transport | 59 |
| biological process | regulation of sporulation resulting in formation of a cellular spore | 59 |
| biological process | response to tellurium ion | 59 |
| biological process | misfolded or incompletely synthesized protein catabolic process | 59 |
| biological process | tRNA methylation | 58 |
| biological process | hemolysis by symbiont of host erythrocytes | 58 |
| biological process | FAD biosynthetic process | 58 |
| biological process | dTTP biosynthetic process | 58 |
| biological process | cellular response to acidic pH | 58 |
| biological process | sulfate reduction | 57 |
| biological process | dicarboxylic acid transport | 57 |
| biological process | electron transport chain | 57 |
| biological process | translational termination | 57 |
| biological process | mRNA processing | 57 |
| biological process | L-serine catabolic process | 57 |
| biological process | nucleoside metabolic process | 57 |
| biological process | negative regulation of catalytic activity | 57 |
| biological process | asparaginyl-tRNA aminoacylation | 57 |
| biological process | glutathione metabolic process | 56 |
| biological process | serine family amino acid biosynthetic process | 56 |
| biological process | riboflavin transport | 56 |
| biological process | tRNA metabolic process | 56 |
| biological process | mitochondrial electron transport, NADH to ubiquinone | 56 |
| biological process | tRNA pseudouridine synthesis | 56 |
| biological process | deoxyribonucleotide catabolic process | 56 |
| biological process | enterobacterial common antigen biosynthetic process | 55 |
| biological process | regulation of phosphorelay signal transduction system | 55 |
| biological process | threonyl-tRNA aminoacylation | 55 |
| biological process | aspartate catabolic process | 55 |
| biological process | glutaminyl-tRNAGln biosynthesis via transamidation | 55 |
| biological process | polyamine transmembrane transport | 54 |
| biological process | protein transport by the Sec complex | 54 |
| biological process | DNA biosynthetic process | 54 |
| biological process | secondary metabolite biosynthetic process | 54 |
| biological process | establishment of integrated proviral latency | 54 |
| biological process | glutamine biosynthetic process | 54 |
| biological process | polysaccharide catabolic process | 53 |
| biological process | carboxylic acid metabolic process | 53 |
| biological process | polyamine biosynthetic process | 53 |
| biological process | glutaminyl-tRNA aminoacylation | 53 |
| biological process | aspartate metabolic process | 53 |
| biological process | monosaccharide metabolic process | 53 |
| biological process | glutamine transport | 52 |
| biological process | ER to Golgi vesicle-mediated transport | 52 |
| biological process | response to hydroperoxide | 52 |
| biological process | response to osmotic stress | 52 |
| biological process | L-serine metabolic process | 52 |
| biological process | ATP hydrolysis coupled proton transport | 51 |
| biological process | cellular response to oxygen levels | 51 |
| biological process | cellular response to alkaline pH | 51 |
| biological process | cellular response to salt stress | 51 |
| biological process | chromate transport | 50 |
| biological process | tRNA wobble adenosine to inosine editing | 50 |
| biological process | nitrite transport | 50 |
| biological process | C-5 methylation of cytosine | 50 |
| biological process | nucleoside triphosphate biosynthetic process | 50 |
| biological process | hydrogen ion transmembrane transport | 50 |
| biological process | response to hypoxia | 50 |
| biological process | response to cadmium ion | 49 |
| biological process | respiratory electron transport chain | 49 |
| biological process | amine metabolic process | 49 |
| biological process | type IV pilus-dependent motility | 49 |
| biological process | aspartate family amino acid biosynthetic process | 48 |
| biological process | tRNA wobble uridine modification | 48 |
| biological process | pyrimidine nucleotide metabolic process | 48 |
| biological process | propionate metabolic process, methylcitrate cycle | 48 |
| biological process | maltose metabolic process | 48 |
| biological process | FtsZ-dependent cytokinesis | 48 |
| biological process | molybdate ion transport | 47 |
| biological process | zinc II ion transmembrane transport | 47 |
| biological process | wobble position uridine ribose methylation | 47 |
| biological process | sulfur amino acid metabolic process | 47 |
| biological process | glycine biosynthetic process | 47 |
| biological process | 'de novo' AMP biosynthetic process | 47 |
| biological process | glutathione biosynthetic process | 47 |
| biological process | DNA dealkylation involved in DNA repair | 47 |
| biological process | wobble position cytosine ribose methylation | 47 |
| biological process | heme transport | 46 |
| biological process | tetrahydrobiopterin biosynthetic process | 46 |
| biological process | glyoxylate cycle | 46 |
| biological process | cellular response to osmotic stress | 46 |
| biological process | response to copper ion | 45 |
| biological process | polyamine catabolic process | 45 |
| biological process | mitochondrion organization | 45 |
| biological process | ATP biosynthetic process | 45 |
| biological process | adhesion of symbiont to host cell | 44 |
| biological process | IMP metabolic process | 44 |
| biological process | L-phenylalanine biosynthetic process | 44 |
| biological process | defense response to bacterium | 44 |
| biological process | response to nitrosative stress | 44 |
| biological process | thiamine diphosphate biosynthetic process | 44 |
| biological process | cellular response to nutrient levels | 44 |
| biological process | Golgi vesicle docking | 44 |
| biological process | fatty acid beta-oxidation | 43 |
| biological process | multicellular organism development | 43 |
| biological process | regulation of fatty acid metabolic process | 43 |
| biological process | plasmid maintenance | 43 |
| biological process | dTDP biosynthetic process | 43 |
| biological process | dUDP biosynthetic process | 43 |
| biological process | trehalose catabolic process | 42 |
| biological process | self proteolysis | 42 |
| biological process | UTP catabolic process | 42 |
| biological process | dipeptide transport | 42 |
| biological process | sucrose transport | 42 |
| biological process | galactitol metabolic process | 42 |
| biological process | sodium ion transmembrane transport | 42 |
| biological process | dTTP catabolic process | 42 |
| biological process | entry into host via enzymatic degradation of host anatomical structure | 42 |
| biological process | evasion or tolerance by symbiont of host-produced reactive oxygen species | 42 |
| biological process | dATP catabolic process | 42 |
| biological process | TTP catabolic process | 42 |
| biological process | fumarate transport | 41 |
| biological process | adenine salvage | 41 |
| biological process | glycerol-3-phosphate metabolic process | 41 |
| biological process | cellular amino acid catabolic process | 41 |
| biological process | acetate biosynthetic process | 41 |
| biological process | single-species biofilm formation on inanimate substrate | 41 |
| biological process | chaperone-mediated protein complex assembly | 41 |
| biological process | copper ion transport | 41 |
| biological process | cellular response to sulfur starvation | 41 |
| biological process | galactitol transport | 41 |
| biological process | L-aspartate transmembrane transport | 41 |
| biological process | response to salt stress | 40 |
| biological process | cell adhesion involved in biofilm formation | 40 |
| biological process | D-arabinose catabolic process | 40 |
| biological process | sporulation resulting in formation of a cellular spore | 40 |
| biological process | spore germination | 40 |
| biological process | negative regulation of ribosome biogenesis | 40 |
| biological process | lysine biosynthetic process | 40 |
| biological process | cellular response to antibiotic | 40 |
| biological process | response to host immune response | 40 |
| biological process | lipoate biosynthetic process | 40 |
| biological process | SNARE complex assembly | 40 |
| biological process | replication fork reversal | 39 |
| biological process | N-acetylneuraminate catabolic process | 39 |
| biological process | pyridoxal 5'-phosphate salvage | 39 |
| biological process | regulation of cell division | 39 |
| biological process | cellular sodium ion homeostasis | 39 |
| biological process | purine nucleoside transmembrane transport | 39 |
| biological process | cell motility | 38 |
| biological process | glutathione transmembrane transport | 38 |
| biological process | protoporphyrinogen IX biosynthetic process | 38 |
| biological process | protein secretion by the type IV secretion system | 38 |
| biological process | homocysteine metabolic process | 38 |
| biological process | positive regulation by symbiont of host immune response | 38 |
| biological process | cadmium ion transmembrane transport | 38 |
| biological process | threonine metabolic process | 38 |
| biological process | bacteriocin transport | 37 |
| biological process | membrane lipid biosynthetic process | 37 |
| biological process | cellular response to starvation | 37 |
| biological process | protein refolding | 37 |
| biological process | propanediol metabolic process | 37 |
| biological process | quinolinate catabolic process | 37 |
| biological process | defense response to virus | 37 |
| biological process | adenosine catabolic process | 37 |
| biological process | succinate metabolic process | 37 |
| biological process | thymidine metabolic process | 37 |
| biological process | negative regulation of transcription from RNA polymerase II promoter | 37 |
| biological process | protein deacetylation | 37 |
| biological process | short-chain fatty acid catabolic process | 37 |
| biological process | cellular aromatic compound metabolic process | 37 |
| biological process | nucleotide metabolic process | 37 |
| biological process | selenocysteine incorporation | 37 |
| biological process | barrier septum site selection | 37 |
| biological process | arginine catabolic process | 37 |
| biological process | RNA metabolic process | 36 |
| biological process | intracellular sequestering of iron ion | 36 |
| biological process | Gram-negative-bacterium-type cell outer membrane assembly | 36 |
| biological process | selenocysteinyl-tRNA(Sec) biosynthetic process | 36 |
| biological process | cytoskeleton organization | 36 |
| biological process | vitamin B6 metabolic process | 36 |
| biological process | L-glutamate transmembrane transport | 36 |
| biological process | ATP metabolic process | 36 |
| biological process | virion attachment to host cell | 36 |
| biological process | phage shock | 36 |
| biological process | lipid catabolic process | 35 |
| biological process | protein peptidyl-prolyl isomerization | 35 |
| biological process | anaerobic electron transport chain | 35 |
| biological process | cellular copper ion homeostasis | 35 |
| biological process | bacteriocin immunity | 35 |
| biological process | lipid transport | 35 |
| biological process | mannose metabolic process | 35 |
| biological process | xanthine metabolic process | 35 |
| biological process | folic acid biosynthetic process | 35 |
| biological process | cell growth | 35 |
| biological process | D-gluconate metabolic process | 35 |
| biological process | purine-containing compound salvage | 34 |
| biological process | acetyl-CoA biosynthetic process | 34 |
| biological process | copper ion homeostasis | 34 |
| biological process | Entner-Doudoroff pathway through 6-phosphogluconate | 34 |
| biological process | hypoxanthine salvage | 34 |
| biological process | regulation of catalytic activity | 34 |
| biological process | carnitine metabolic process | 34 |
| biological process | cytidine deamination | 34 |
| biological process | response to aluminum ion | 34 |
| biological process | regulation of pentose-phosphate shunt | 34 |
| biological process | succinyl-CoA metabolic process | 34 |
| biological process | inosine biosynthetic process | 33 |
| biological process | plasma membrane ATP synthesis coupled proton transport | 33 |
| biological process | cell adhesion involved in single-species biofilm formation | 33 |
| biological process | regulation of nitrogen utilization | 33 |
| biological process | cellular metabolic process | 33 |
| biological process | organic phosphonate catabolic process | 33 |
| biological process | regulation of pH | 33 |
| biological process | nucleotide biosynthetic process | 33 |
| biological process | positive regulation of gene expression | 33 |
| biological process | glutamine catabolic process | 33 |
| biological process | mRNA polyadenylation | 33 |
| biological process | chloride transport | 32 |
| biological process | alkanesulfonate transport | 32 |
| biological process | trehalose transport | 32 |
| biological process | glycosylation | 32 |
| biological process | cellular response to iron ion starvation | 32 |
| biological process | protein homotrimerization | 32 |
| biological process | farnesyl diphosphate biosynthetic process | 32 |
| biological process | sulfate transport | 32 |
| biological process | starch biosynthetic process | 32 |
| biological process | D-tagatose 6-phosphate catabolic process | 32 |
| biological process | glucuronate catabolic process | 32 |
| biological process | copper ion export | 32 |
| biological process | pyrimidine nucleotide biosynthetic process | 32 |
| biological process | cobalamin transport | 32 |
| biological process | vacuolar proton-transporting V-type ATPase complex assembly | 32 |
| biological process | pantothenate metabolic process | 32 |
| biological process | teichoic acid biosynthetic process | 32 |
| biological process | sucrose catabolic process | 32 |
| biological process | endospore formation | 31 |
| biological process | intracellular signal transduction | 31 |
| biological process | sporulation | 31 |
| biological process | DNA methylation on cytosine | 31 |
| biological process | carbohydrate utilization | 31 |
| biological process | cellular hyperosmotic salinity response | 31 |
| biological process | alanine biosynthetic process | 31 |
| biological process | response to arsenic-containing substance | 31 |
| biological process | protein localization | 31 |
| biological process | lactose catabolic process | 31 |
| biological process | glycolipid biosynthetic process | 30 |
| biological process | developmental process | 30 |
| biological process | cilium assembly | 30 |
| biological process | phenylacetate catabolic process | 30 |
| biological process | single organismal cell-cell adhesion | 30 |
| biological process | inositol phosphate dephosphorylation | 30 |
| biological process | glycol catabolic process | 30 |
| biological process | intracellular protein transmembrane transport | 30 |
| biological process | cellular homeostasis | 30 |
| biological process | mannan catabolic process | 30 |
| biological process | glutamate metabolic process | 30 |
| biological process | negative regulation of translational initiation | 30 |
| biological process | inositol metabolic process | 30 |
| biological process | positive regulation of cell division | 29 |
| biological process | lactate transport | 29 |
| biological process | protein targeting | 29 |
| biological process | muscle contraction | 29 |
| biological process | protein import | 29 |
| biological process | trans-translation-dependent protein tagging | 29 |
| biological process | pectin catabolic process | 29 |
| biological process | cellular response to drug | 29 |
| biological process | ethanolamine metabolic process | 29 |
| biological process | propionate catabolic process, 2-methylcitrate cycle | 29 |
| biological process | terpenoid biosynthetic process | 29 |
| biological process | collagen fibril organization | 29 |
| biological process | methylglyoxal biosynthetic process | 29 |
| biological process | carbohydrate biosynthetic process | 29 |
| biological process | intracellular pH elevation | 29 |
| biological process | nucleotide catabolic process | 29 |
| biological process | bacterial-type flagellum assembly | 28 |
| biological process | response to reactive oxygen species | 28 |
| biological process | isocitrate metabolic process | 28 |
| biological process | DNA synthesis involved in double-strand break repair via homologous recombination | 28 |
| biological process | argininosuccinate metabolic process | 28 |
| biological process | capsule polysaccharide biosynthetic process | 28 |
| biological process | response to metal ion | 28 |
| biological process | DNA methylation on adenine | 28 |
| biological process | dTMP biosynthetic process | 28 |
| biological process | monosaccharide transport | 28 |
| biological process | quaternary ammonium group transport | 28 |
| biological process | cilium or flagellum-dependent cell motility | 28 |
| biological process | methylglyoxal catabolic process | 28 |
| biological process | regulation of potassium ion transport | 27 |
| biological process | maintenance of CRISPR repeat elements | 27 |
| biological process | UV protection | 27 |
| biological process | negative regulation of strand invasion | 27 |
| biological process | L-arabinose transport | 27 |
| biological process | long-chain fatty acid metabolic process | 27 |
| biological process | pyridoxal phosphate biosynthetic process | 27 |
| biological process | putrescine catabolic process | 26 |
| biological process | 3-phenylpropionate catabolic process | 26 |
| biological process | sorocarp morphogenesis | 26 |
| biological process | protein adenylylation | 26 |
| biological process | response to iron ion | 26 |
| biological process | glycine betaine transport | 26 |
| biological process | lipoprotein transport | 26 |
| biological process | homoserine biosynthetic process | 26 |
| biological process | putrescine biosynthetic process from ornithine | 26 |
| biological process | visual perception | 26 |
| biological process | oligopeptide transmembrane transport | 26 |
| biological process | detoxification of copper ion | 26 |
| biological process | D-alanine biosynthetic process | 26 |
| biological process | fructose metabolic process | 26 |
| biological process | evasion or tolerance by symbiont of host-produced nitric oxide | 26 |
| biological process | RNA-dependent DNA biosynthetic process | 26 |
| biological process | siderophore biosynthetic process | 26 |
| biological process | allantoin catabolic process | 26 |
| biological process | quorum sensing | 26 |
| biological process | glycoside catabolic process | 25 |
| biological process | Gram-negative-bacterium-type cell wall biogenesis | 25 |
| biological process | acyl-CoA metabolic process | 25 |
| biological process | movement of cell or subcellular component | 25 |
| biological process | cellular response to amino acid stimulus | 25 |
| biological process | 2'-deoxyribonucleotide metabolic process | 25 |
| biological process | formate oxidation | 25 |
| biological process | glycerol-3-phosphate catabolic process | 25 |
| biological process | melibiose transport | 25 |
| biological process | L-methionine biosynthetic process from methylthioadenosine | 25 |
| biological process | phospholipid transport | 25 |
| biological process | nitrate transport | 25 |
| biological process | response to temperature stimulus | 25 |
| biological process | tetrapyrrole biosynthetic process | 25 |
| biological process | hyperosmotic response | 25 |
| biological process | drug export | 25 |
| biological process | ornithine biosynthetic process | 25 |
| biological process | cellular response to nutrient | 25 |
| biological process | bacterial-type flagellum organization | 25 |
| biological process | methylgalactoside transport | 25 |
| biological process | oxygen metabolic process | 25 |
| biological process | DNA import into cell involved in transformation | 24 |
| biological process | ATP synthesis coupled electron transport | 24 |
| biological process | regulation of DNA replication | 24 |
| biological process | protein insertion into membrane | 24 |
| biological process | keto-3-deoxy-D-manno-octulosonic acid biosynthetic process | 24 |
| biological process | nitrogen fixation | 24 |
| biological process | growth of symbiont in host | 24 |
| biological process | thylakoid membrane organization | 24 |
| biological process | N-terminal peptidyl-alanine acetylation | 24 |
| biological process | L-ascorbic acid catabolic process | 24 |
| biological process | aromatic amino acid family biosynthetic process, prephenate pathway | 24 |
| biological process | exocytosis | 24 |
| biological process | chloride transmembrane transport | 24 |
| biological process | biotin metabolic process | 24 |
| biological process | photosynthesis | 24 |
| biological process | fungal-type cell wall organization | 24 |
| biological process | base-excision repair, AP site formation | 23 |
| biological process | histidine metabolic process | 23 |
| biological process | sorocarp stalk cell differentiation | 23 |
| biological process | chemokine-mediated signaling pathway | 23 |
| biological process | cell separation after cytokinesis | 23 |
| biological process | mitotic sister chromatid segregation | 23 |
| biological process | response to acidic pH | 23 |
| biological process | extracellular matrix organization | 23 |
| biological process | cofactor catabolic process | 23 |
| biological process | glyceraldehyde-3-phosphate metabolic process | 23 |
| biological process | methane metabolic process | 23 |
| biological process | glycyl-tRNA aminoacylation | 23 |
| biological process | histidine catabolic process to glutamate and formamide | 23 |
| biological process | ribosome assembly | 23 |
| biological process | protein targeting to Golgi | 23 |
| biological process | L-ascorbic acid transport | 23 |
| biological process | N,N'-diacetylchitobiose import | 23 |
| biological process | GDP-mannose biosynthetic process | 23 |
| biological process | slime layer polysaccharide biosynthetic process | 23 |
| biological process | lysine metabolic process | 23 |
| biological process | sorocarp spore cell differentiation | 23 |
| biological process | ganglioside catabolic process | 23 |
| biological process | rRNA catabolic process | 23 |
| biological process | Actinobacterium-type cell wall biogenesis | 22 |
| biological process | histidine catabolic process to glutamate and formate | 22 |
| biological process | endocytosis | 22 |
| biological process | protein methylation | 22 |
| biological process | formate transport | 22 |
| biological process | cysteine biosynthetic process | 22 |
| biological process | peptidyl-diphthamide biosynthetic process from peptidyl-histidine | 22 |
| biological process | glycogen metabolic process | 22 |
| biological process | microcin transport | 22 |
| biological process | starch catabolic process | 22 |
| biological process | D-ribose catabolic process | 22 |
| biological process | negative regulation of DNA-templated transcription, termination | 22 |
| biological process | protein ubiquitination | 22 |
| biological process | AMP salvage | 22 |
| biological process | conjugation | 22 |
| biological process | 3,4-dihydroxybenzoate biosynthetic process | 22 |
| biological process | nickel cation transport | 22 |
| biological process | spermidine transmembrane transport | 21 |
| biological process | ketone catabolic process | 21 |
| biological process | proteolysis involved in cellular protein catabolic process | 21 |
| biological process | asparagine metabolic process | 21 |
| biological process | carbon fixation | 21 |
| biological process | adenine catabolic process | 21 |
| biological process | steroid metabolic process | 21 |
| biological process | response to hypochlorite | 21 |
| biological process | axon development | 21 |
| biological process | manganese ion transmembrane transport | 21 |
| biological process | glycine betaine biosynthetic process from choline | 21 |
| biological process | negative regulation of immune response | 21 |
| biological process | galactarate catabolic process | 21 |
| biological process | ammonia assimilation cycle | 21 |
| biological process | selenium compound metabolic process | 21 |
| biological process | L-proline biosynthetic process | 21 |
| biological process | protein complex assembly | 21 |
| biological process | guanine metabolic process | 21 |
| biological process | ferric-enterobactin transport | 21 |
| biological process | DNA ligation | 21 |
| biological process | anaerobic glycerol catabolic process | 21 |
| biological process | plasma membrane copper ion transport | 21 |
| biological process | gamma-aminobutyric acid catabolic process | 21 |
| biological process | proteasomal protein catabolic process | 21 |
| biological process | xylan catabolic process | 21 |
| biological process | organic acid transport | 20 |
| biological process | 1-butanol biosynthetic process | 20 |
| biological process | regulation of response to stress | 20 |
| biological process | cysteine metabolic process | 20 |
| biological process | lactose biosynthetic process | 20 |
| biological process | protein unfolding | 20 |
| biological process | alkylphosphonate transport | 20 |
| biological process | cellulose biosynthetic process | 20 |
| biological process | mitochondrial genome maintenance | 20 |
| biological process | hypoxanthine biosynthetic process | 20 |
| biological process | viral tail assembly | 20 |
| biological process | generation of precursor metabolites and energy | 20 |
| biological process | zinc II ion transport | 20 |
| biological process | xyloglucan metabolic process | 20 |
| biological process | valine catabolic process | 20 |
| biological process | choline catabolic process | 20 |
| biological process | lysine catabolic process | 20 |
| biological process | L-cystine transport | 20 |
| biological process | pyrimidine-containing compound salvage | 20 |
| biological process | cell death | 20 |
| biological process | sister chromatid cohesion | 20 |
| biological process | response to pH | 20 |
| biological process | growth of symbiont in host cell | 20 |
| biological process | cysteine transport | 20 |
| biological process | L-alanine transport | 20 |
| biological process | galactose catabolic process | 20 |
| biological process | branched-chain amino acid catabolic process | 20 |
| biological process | embryo development ending in seed dormancy | 20 |
| biological process | 'de novo' UMP biosynthetic process | 20 |
| biological process | formate metabolic process | 20 |
| biological process | pyrimidine nucleobase catabolic process | 19 |
| biological process | protein autoprocessing | 19 |
| biological process | short-chain fatty acid metabolic process | 19 |
| biological process | thiamine transport | 19 |
| biological process | telomere maintenance via recombination | 19 |
| biological process | ascospore formation | 19 |
| biological process | electron transport coupled proton transport | 19 |
| biological process | nucleoside transmembrane transport | 19 |
| biological process | L-phenylalanine metabolic process | 19 |
| biological process | alpha-glucan catabolic process | 19 |
| biological process | sarcomere organization | 19 |
| biological process | cysteine transmembrane transport | 19 |
| biological process | maltodextrin transport | 19 |
| biological process | regulation of phosphate metabolic process | 19 |
| biological process | allantoin assimilation pathway | 19 |
| biological process | D-allose catabolic process | 19 |
| biological process | organic phosphonate transport | 19 |
| biological process | NADPH regeneration | 19 |
| biological process | tryptophan metabolic process | 19 |
| biological process | cofactor biosynthetic process | 19 |
| biological process | cytoskeleton-dependent intracellular transport | 19 |
| biological process | regulation of cell cycle | 19 |
| biological process | D-xylose transport | 19 |
| biological process | actin filament organization | 18 |
| biological process | RNA phosphodiester bond hydrolysis | 18 |
| biological process | glucose catabolic process | 18 |
| biological process | sorocarp development | 18 |
| biological process | skin development | 18 |
| biological process | DNA catabolic process, exonucleolytic | 18 |
| biological process | chlorophyll biosynthetic process | 18 |
| biological process | glucose import | 18 |
| biological process | hexose metabolic process | 18 |
| biological process | carbon fixation by acetyl-CoA pathway | 18 |
| biological process | D-glucarate catabolic process | 18 |
| biological process | 5-phosphoribose 1-diphosphate biosynthetic process | 18 |
| biological process | active evasion of host immune response | 18 |
| biological process | tryptophan catabolic process to kynurenine | 18 |
| biological process | trehalose metabolic process | 18 |
| biological process | negative regulation of sequence-specific DNA binding transcription factor activity | 18 |
| biological process | glutamate catabolic process via 2-hydroxyglutarate | 18 |
| biological process | glucan biosynthetic process | 18 |
| biological process | cellular glucan metabolic process | 18 |
| biological process | negative regulation of nucleic acid-templated transcription | 18 |
| biological process | cell cycle | 18 |
| biological process | ferric iron transport | 18 |
| biological process | RNA phosphodiester bond hydrolysis, exonucleolytic | 18 |
| biological process | choline transport | 18 |
| biological process | cellular oligosaccharide catabolic process | 18 |
| biological process | microtubule cytoskeleton organization | 18 |
| biological process | bidirectional double-stranded viral DNA replication | 18 |
| biological process | cellular respiration | 18 |
| biological process | positive regulation of translation | 17 |
| biological process | culmination involved in sorocarp development | 17 |
| biological process | cell morphogenesis | 17 |
| biological process | negative regulation of cell growth | 17 |
| biological process | mycelium development | 17 |
| biological process | NADH oxidation | 17 |
| biological process | cellular transition metal ion homeostasis | 17 |
| biological process | regulation of carbohydrate metabolic process | 17 |
| biological process | alginic acid biosynthetic process | 17 |
| biological process | 6-sulfoquinovose(1-) catabolic process | 17 |
| biological process | iron assimilation | 17 |
| biological process | iron assimilation by chelation and transport | 17 |
| biological process | post-translational protein modification | 17 |
| biological process | xenobiotic metabolic process | 17 |
| biological process | auxin metabolic process | 17 |
| biological process | RNA repair | 17 |
| biological process | negative regulation of protein kinase activity | 17 |
| biological process | positive chemotaxis | 17 |
| biological process | guanosine tetraphosphate biosynthetic process | 17 |
| biological process | wound healing | 17 |
| biological process | positive regulation of rRNA processing | 17 |
| biological process | enterobactin biosynthetic process | 17 |
| biological process | negative regulation of single-species biofilm formation on inanimate substrate | 17 |
| biological process | attachment of mitotic spindle microtubules to kinetochore | 17 |
| biological process | skeletal system development | 17 |
| biological process | cellular response to acid chemical | 17 |
| biological process | L-arginine import into cell | 17 |
| biological process | ventricular system development | 17 |
| biological process | cellular response to bacteriocin | 17 |
| biological process | protein secretion by the type VI secretion system | 16 |
| biological process | peptidyl-tyrosine dephosphorylation | 16 |
| biological process | positive regulation of transcription from RNA polymerase II promoter | 16 |
| biological process | protein localization to outer membrane | 16 |
| biological process | L-threonine catabolic process to propionate | 16 |
| biological process | lactate metabolic process | 16 |
| biological process | protein flavinylation | 16 |
| biological process | cell wall assembly | 16 |
| biological process | calcium ion transport | 16 |
| biological process | negative regulation of bacterial-type flagellum-dependent cell motility | 16 |
| biological process | primary metabolic process | 16 |
| biological process | oligosaccharide biosynthetic process | 16 |
| biological process | cell wall modification | 16 |
| biological process | calcium ion transmembrane transport | 16 |
| biological process | mitotic chromosome condensation | 16 |
| biological process | regulation of chromosome segregation | 16 |
| biological process | negative regulation of neutrophil activation | 16 |
| biological process | chromosome organization | 16 |
| biological process | ADP-L-glycero-beta-D-manno-heptose biosynthetic process | 16 |
| biological process | peptidoglycan transport | 16 |
| biological process | fucosylation | 16 |
| biological process | lipoprotein localization to outer membrane | 16 |
| biological process | transposition, RNA-mediated | 16 |
| biological process | lipopolysaccharide export | 16 |
| biological process | 'de novo' cotranslational protein folding | 16 |
| biological process | polyphosphate metabolic process | 16 |
| biological process | regulation of chaperone-mediated protein complex assembly | 16 |
| biological process | galactose catabolic process via UDP-galactose | 16 |
| biological process | negative regulation of fatty acid biosynthetic process | 16 |
| biological process | protoporphyrinogen IX biosynthetic process from glutamate | 15 |
| biological process | regulation of DNA recombination | 15 |
| biological process | regulation of single-species biofilm formation | 15 |
| biological process | type IV pilus biogenesis | 15 |
| biological process | lipid A metabolic process | 15 |
| biological process | arginine transport | 15 |
| biological process | L-idonate catabolic process | 15 |
| biological process | 'de novo' GDP-L-fucose biosynthetic process | 15 |
| biological process | pteridine-containing compound metabolic process | 15 |
| biological process | telomere maintenance via telomerase | 15 |
| biological process | propionate metabolic process, methylmalonyl pathway | 15 |
| biological process | sulfate transmembrane transport | 15 |
| biological process | phosphatidylethanolamine biosynthetic process | 15 |
| biological process | lipoprotein biosynthetic process | 15 |
| biological process | cilium morphogenesis | 15 |
| biological process | chromosome organization involved in meiotic cell cycle | 15 |
| biological process | negative regulation of DNA-dependent DNA replication initiation | 15 |
| biological process | sensory perception of sound | 15 |
| biological process | putrescine biosynthetic process from arginine | 15 |
| biological process | nitrogen utilization | 15 |
| biological process | organic acid phosphorylation | 15 |
| biological process | negative regulation of gene expression | 15 |
| biological process | threonine transport | 15 |
| biological process | leucine metabolic process | 15 |
| biological process | cell migration | 15 |
| biological process | L-phenylalanine biosynthetic process from chorismate via phenylpyruvate | 15 |
| biological process | small GTPase mediated signal transduction | 15 |
| biological process | regulation of membrane potential | 15 |
| biological process | membrane organization | 14 |
| biological process | isopentenyl diphosphate biosynthetic process, mevalonate pathway | 14 |
| biological process | chromatin remodeling | 14 |
| biological process | NADP biosynthetic process | 14 |
| biological process | pantothenate biosynthetic process from valine | 14 |
| biological process | dolichol-linked oligosaccharide biosynthetic process | 14 |
| biological process | G-quadruplex DNA unwinding | 14 |
| biological process | maturation of SSU-rRNA | 14 |
| biological process | defense response | 14 |
| biological process | epithelial cilium movement | 14 |
| biological process | retinal rod cell development | 14 |
| biological process | galactitol catabolic process | 14 |
| biological process | acetyl-CoA metabolic process | 14 |
| biological process | regulation of DNA-templated transcription, elongation | 14 |
| biological process | molybdenum incorporation into molybdenum-molybdopterin complex | 14 |
| biological process | glycosaminoglycan catabolic process | 14 |
| biological process | mitotic nuclear division | 14 |
| biological process | L-threonine catabolic process to glycine | 14 |
| biological process | response to biotic stimulus | 14 |
| biological process | negative regulation of phosphate transmembrane transport | 14 |
| biological process | tyrosyl-tRNA aminoacylation | 14 |
| biological process | vitamin transmembrane transport | 14 |
| biological process | ubiquitin-dependent protein catabolic process | 14 |
| biological process | septin ring assembly | 14 |
| biological process | mitotic cytokinesis | 14 |
| biological process | response to UV | 14 |
| biological process | heart morphogenesis | 14 |
| biological process | telomere capping | 14 |
| biological process | glutamate catabolic process | 14 |
| biological process | thiamine metabolic process | 14 |
| biological process | histone deacetylation | 14 |
| biological process | branched-chain amino acid metabolic process | 14 |
| biological process | protein pupylation | 14 |
| biological process | ribosomal small subunit biogenesis | 14 |
| biological process | establishment of mitotic spindle orientation | 14 |
| biological process | gluconate transmembrane transport | 14 |
| biological process | chromatin modification | 14 |
| biological process | response to wounding | 14 |
| biological process | lipid modification | 14 |
| biological process | blood vessel development | 14 |
| biological process | negative regulation of endopeptidase activity | 14 |
| biological process | negative regulation of phosphate metabolic process | 14 |
| biological process | proline transmembrane transport | 14 |
| biological process | protein oligomerization | 13 |
| biological process | geranylgeranyl diphosphate biosynthetic process | 13 |
| biological process | protein processing involved in protein targeting to mitochondrion | 13 |
| biological process | chromosome separation | 13 |
| biological process | acetate biosynthetic process from carbon monoxide | 13 |
| biological process | modification-dependent protein catabolic process | 13 |
| biological process | termination of RNA polymerase I transcription | 13 |
| biological process | hypoxanthine catabolic process | 13 |
| biological process | extracellular fibril organization | 13 |
| biological process | response to silver ion | 13 |
| biological process | programmed cell death | 13 |
| biological process | cell differentiation | 13 |
| biological process | UMP salvage | 13 |
| biological process | lactate oxidation | 13 |
| biological process | cellular response to peptidoglycan | 13 |
| biological process | cytokinesis, site selection | 13 |
| biological process | peptidyl-serine phosphorylation | 13 |
| biological process | cellular response to copper ion | 13 |
| biological process | protein targeting to membrane | 13 |
| biological process | SRP-dependent cotranslational protein targeting to membrane | 13 |
| biological process | stress response to copper ion | 13 |
| biological process | adhesion of symbiont to host | 13 |
| biological process | mycothiol biosynthetic process | 13 |
| biological process | unsaturated fatty acid biosynthetic process | 13 |
| biological process | plasma membrane acetate transport | 13 |
| biological process | female mating behavior | 13 |
| biological process | nitrate metabolic process | 13 |
| biological process | malonyl-CoA biosynthetic process | 13 |
| biological process | peptidyl-tyrosine autophosphorylation | 13 |
| biological process | mitochondrial respiratory chain complex assembly | 13 |
| biological process | D-amino acid catabolic process | 13 |
| biological process | dipeptide transmembrane transport | 13 |
| biological process | cellulose catabolic process | 13 |
| biological process | response to cytokinin | 13 |
| biological process | negative regulation of neuron differentiation | 13 |
| biological process | isopentenyl diphosphate biosynthetic process | 13 |
| biological process | positive regulation of cell motility | 13 |
| biological process | photorespiration | 13 |
| biological process | NAD salvage | 13 |
| biological process | D-xylose catabolic process | 13 |
| biological process | glyoxylate catabolic process | 13 |
| biological process | cytosine catabolic process | 13 |
| biological process | regulation of protein stability | 13 |
| biological process | autolysis | 13 |
| biological process | nucleocytoplasmic transport | 13 |
| biological process | purine nucleobase transport | 13 |
| biological process | axon guidance | 12 |
| biological process | xyloglucan catabolic process | 12 |
| biological process | mannitol transport | 12 |
| biological process | endochondral ossification | 12 |
| biological process | L-methylmalonyl-CoA metabolic process | 12 |
| biological process | anaerobic glutamate catabolic process | 12 |
| biological process | evasion or tolerance of host immune response | 12 |
| biological process | positive regulation of axon extension | 12 |
| biological process | transcytosis | 12 |
| biological process | acetyl-CoA biosynthetic process from acetate | 12 |
| biological process | stress response to cadmium ion | 12 |
| biological process | hydrogen sulfide biosynthetic process | 12 |
| biological process | carnitine catabolic process | 12 |
| biological process | response to light stimulus | 12 |
| biological process | long-chain fatty acid biosynthetic process | 12 |
| biological process | superoxide metabolic process | 12 |
| biological process | enzyme active site formation | 12 |
| biological process | tryptophan transport | 12 |
| biological process | in utero embryonic development | 12 |
| biological process | glutamine family amino acid biosynthetic process | 12 |
| biological process | tRNA catabolic process | 12 |
| biological process | cellular metabolic compound salvage | 12 |
| biological process | peptidyl-pyrromethane cofactor linkage | 12 |
| biological process | leucine catabolic process | 12 |
| biological process | response to mercury ion | 12 |
| biological process | silver ion transmembrane transport | 12 |
| biological process | heparan sulfate proteoglycan catabolic process | 12 |
| biological process | glycine catabolic process | 12 |
| biological process | pyrimidine nucleotide transport | 12 |
| biological process | pyrimidine ribonucleoside catabolic process | 12 |
| biological process | bradykinin catabolic process | 12 |
| biological process | striated muscle contraction | 12 |
| biological process | actin cytoskeleton organization | 12 |
| biological process | beta-ketoadipate pathway | 12 |
| biological process | Golgi vesicle transport | 12 |
| biological process | meiotic nuclear division | 12 |
| biological process | lactate biosynthetic process | 12 |
| biological process | apoptotic process | 12 |
| biological process | regulation of blood pressure | 12 |
| biological process | mutualism | 12 |
| biological process | Golgi to plasma membrane protein transport | 12 |
| biological process | single strand break repair | 12 |
| biological process | nucleoside diphosphate metabolic process | 12 |
| biological process | urea metabolic process | 12 |
| biological process | glycerol transport | 12 |
| biological process | glycolate catabolic process | 12 |
| biological process | membrane fusion | 12 |
| biological process | cellular response to hypoxia | 12 |
| biological process | transcription from RNA polymerase II promoter | 12 |
| biological process | positive regulation of lyase activity | 12 |
| biological process | telomere maintenance | 12 |
| biological process | peptidyl-glutamine methylation | 12 |
| biological process | response to acid chemical | 12 |
| biological process | oxygen transport | 12 |
| biological process | negative regulation of cell division | 12 |
| biological process | dormancy process | 12 |
| biological process | mannitol metabolic process | 11 |
| biological process | creatinine catabolic process | 11 |
| biological process | locomotor rhythm | 11 |
| biological process | positive regulation of cytoskeleton organization | 11 |
| biological process | S-adenosylmethioninamine biosynthetic process | 11 |
| biological process | negative regulation of cell killing | 11 |
| biological process | regulation of sequence-specific DNA binding transcription factor activity | 11 |
| biological process | killing of cells of other organism | 11 |
| biological process | negative regulation of DNA-templated transcription, initiation | 11 |
| biological process | L-lyxose metabolic process | 11 |
| biological process | alanine metabolic process | 11 |
| biological process | photoreceptor cell maintenance | 11 |
| biological process | xanthine transport | 11 |
| biological process | aromatic amino acid transport | 11 |
| biological process | viral DNA genome replication | 11 |
| biological process | DNA ligation involved in DNA repair | 11 |
| biological process | L-ascorbic acid metabolic process | 11 |
| biological process | negative regulation of proteolysis | 11 |
| biological process | mitotic cell cycle | 11 |
| biological process | archaeal or bacterial-type flagellum-dependent cell motility | 11 |
| biological process | catechol-containing compound catabolic process | 11 |
| biological process | skeletal muscle tissue development | 11 |
| biological process | bacterial cellulose biosynthetic process | 11 |
| biological process | negative regulation of DNA binding | 11 |
| biological process | regulation of glycolytic process | 11 |
| biological process | chorismate metabolic process | 11 |
| biological process | prosthetic group biosynthetic process | 11 |
| biological process | cysteine catabolic process | 11 |
| biological process | mitotic metaphase plate congression | 11 |
| biological process | protein secretion by the type III secretion system | 11 |
| biological process | D-serine catabolic process | 11 |
| biological process | negative regulation of telomerase activity | 11 |
| biological process | protein glycosylation | 11 |
| biological process | autophagy | 11 |
| biological process | cell-matrix adhesion | 11 |
| biological process | gamma-aminobutyric acid metabolic process | 11 |
| biological process | attachment of spindle microtubules to kinetochore | 11 |
| biological process | N-acetylmuramic acid catabolic process | 11 |
| biological process | negative regulation of telomere maintenance via telomerase | 11 |
| biological process | cell wall macromolecule biosynthetic process | 11 |
| biological process | cellular response to iron ion | 11 |
| biological process | methionine catabolic process | 11 |
| biological process | glycolytic process from galactose | 11 |
| biological process | negative regulation of calcium-dependent ATPase activity | 11 |
| biological process | protein depalmitoylation | 11 |
| biological process | L-phenylalanine catabolic process | 11 |
| biological process | retina development in camera-type eye | 11 |
| biological process | S-adenosylmethionine cycle | 11 |
| biological process | arginine catabolic process to glutamate | 11 |
| biological process | response to host iron concentration | 11 |
| biological process | S-adenosylmethionine biosynthetic process | 11 |
| biological process | response to abscisic acid | 11 |
| biological process | creatine biosynthetic process | 11 |
| biological process | folic acid catabolic process | 11 |
| biological process | protein localization to organelle | 11 |
| biological process | response to nickel cation | 11 |
| biological process | D-serine biosynthetic process | 11 |
| biological process | oviposition | 11 |
| biological process | negative regulation of ribonuclease activity | 11 |
| biological process | protein polyubiquitination | 10 |
| biological process | viral capsid assembly | 10 |
| biological process | hemolysis in other organism | 10 |
| biological process | polysaccharide transport | 10 |
| biological process | negative regulation of translational elongation | 10 |
| biological process | dUTP metabolic process | 10 |
| biological process | mitotic spindle assembly checkpoint | 10 |
| biological process | glycoprotein catabolic process | 10 |
| biological process | D-alanine catabolic process | 10 |
| biological process | tRNA splicing, via endonucleolytic cleavage and ligation | 10 |
| biological process | progesterone metabolic process | 10 |
| biological process | cerebellar Purkinje cell layer development | 10 |
| biological process | peptidyl-L-beta-methylthioaspartic acid biosynthetic process from peptidyl-aspartic acid | 10 |
| biological process | response to xenobiotic stimulus | 10 |
| biological process | aggregation involved in sorocarp development | 10 |
| biological process | carbon catabolite repression of transcription | 10 |
| biological process | regulation of DNA-templated transcription, initiation | 10 |
| biological process | L-lysine catabolic process | 10 |
| biological process | methylglyoxal metabolic process | 10 |
| biological process | rhamnose metabolic process | 10 |
| biological process | L-ascorbic acid biosynthetic process | 10 |
| biological process | galactolipid biosynthetic process | 10 |
| biological process | mannitol catabolic process | 10 |
| biological process | cellular response to organic substance | 10 |
| biological process | cartilage development involved in endochondral bone morphogenesis | 10 |
| biological process | phosphatidylinositol phosphorylation | 10 |
| biological process | tRNA aminoacylation for mitochondrial protein translation | 10 |
| biological process | negative regulation of apoptotic process | 10 |
| biological process | detection of light stimulus involved in visual perception | 10 |
| biological process | proteasome storage granule assembly | 10 |
| biological process | 2-oxoglutarate metabolic process | 10 |
| biological process | nucleotide phosphorylation | 10 |
| biological process | tRNA thio-modification | 10 |
| biological process | UMP biosynthetic process | 10 |
| biological process | cellular response to silver ion | 10 |
| biological process | transcription initiation from bacterial-type RNA polymerase promoter | 10 |
| biological process | glycerol ether metabolic process | 10 |
| biological process | L-asparagine biosynthetic process | 10 |
| biological process | transforming growth factor beta receptor signaling pathway | 10 |
| biological process | lysine biosynthetic process via aminoadipic acid | 10 |
| biological process | tRNA wobble base modification | 10 |
| biological process | neurogenesis | 10 |
| biological process | spermatogenesis | 10 |
| biological process | phenylalanine transport | 10 |
| biological process | receptor-mediated endocytosis | 10 |
| biological process | fructose 1,6-bisphosphate metabolic process | 10 |
| biological process | cardiac muscle fiber development | 10 |
| biological process | benzene-containing compound metabolic process | 10 |
| biological process | hormone metabolic process | 10 |
| biological process | regulation of translational termination | 10 |
| biological process | rRNA (guanine-N7)-methylation | 10 |
| biological process | organic hydroxy compound metabolic process | 10 |
| biological process | chromatin silencing at telomere | 10 |
| biological process | L-alanine oxidation to pyruvate via D-alanine | 10 |
| biological process | ADP biosynthetic process | 10 |
| biological process | regulation of entry of bacterium into host cell | 10 |
| biological process | arginine catabolic process to succinate | 10 |
| biological process | vesicle fusion with Golgi apparatus | 10 |
| biological process | cellular response to UV | 9 |
| biological process | acetate catabolic process | 9 |
| biological process | collagen biosynthetic process | 9 |
| biological process | bioluminescence | 9 |
| biological process | glycolipid metabolic process | 9 |
| biological process | sucrose metabolic process | 9 |
| biological process | cellular manganese ion homeostasis | 9 |
| biological process | sulfolipid biosynthetic process | 9 |
| biological process | toll-like receptor 5 signaling pathway | 9 |
| biological process | L-lysine catabolic process to acetyl-CoA via saccharopine | 9 |
| biological process | glucose 6-phosphate metabolic process | 9 |
| biological process | response to mechanical stimulus | 9 |
| biological process | ribosomal large subunit biogenesis | 9 |
| biological process | DNA strand elongation involved in DNA replication | 9 |
| biological process | oxalate metabolic process | 9 |
| biological process | response to ethanol | 9 |
| biological process | stress-activated protein kinase signaling cascade | 9 |
| biological process | filamentous growth | 9 |
| biological process | rRNA 5'-end processing | 9 |
| biological process | GDP-mannose metabolic process | 9 |
| biological process | negative regulation by symbiont of host apoptotic process | 9 |
| biological process | proline transport | 9 |
| biological process | mRNA export from nucleus | 9 |
| biological process | nucleosome assembly | 9 |
| biological process | tRNA 3'-end processing | 9 |
| biological process | acquisition of nutrients from host | 9 |
| biological process | regulation of bacterial-type flagellum-dependent cell motility | 9 |
| biological process | UMP catabolic process | 9 |
| biological process | cellular response to amino acid starvation | 9 |
| biological process | acquisition by symbiont of nutrients from host via siderophores | 9 |
| biological process | protein ubiquitination involved in ubiquitin-dependent protein catabolic process | 9 |
| biological process | macrolide biosynthetic process | 9 |
| biological process | bile acid catabolic process | 9 |
| biological process | negative regulation of growth | 9 |
| biological process | L-arabinose metabolic process | 9 |
| biological process | protein de-ADP-ribosylation | 9 |
| biological process | polyprenol biosynthetic process | 9 |
| biological process | vesicle fusion | 9 |
| biological process | proteoglycan metabolic process | 9 |
| biological process | retina layer formation | 9 |
| biological process | protein heterotrimerization | 9 |
| biological process | bile acid and bile salt transport | 9 |
| biological process | alcohol metabolic process | 9 |
| biological process | amylopectin biosynthetic process | 9 |
| biological process | chitin metabolic process | 9 |
| biological process | regulation of mRNA stability | 9 |
| biological process | protein transport by the Tat complex | 9 |
| biological process | cAMP biosynthetic process | 9 |
| biological process | response to nitric oxide | 9 |
| biological process | immune response | 9 |
| biological process | chromatin silencing | 9 |
| biological process | MyD88-dependent toll-like receptor signaling pathway | 9 |
| biological process | targeting of mRNA for destruction involved in RNA interference | 9 |
| biological process | maintenance of DNA methylation | 9 |
| biological process | multicellular organism growth | 9 |
| biological process | D-gluconate catabolic process | 9 |
| biological process | notochord development | 9 |
| biological process | beta-lactam antibiotic catabolic process | 9 |
| biological process | entry into host | 9 |
| biological process | epithelial cilium movement involved in determination of left/right asymmetry | 9 |
| biological process | response to iron(III) ion | 9 |
| biological process | lysosome organization | 9 |
| biological process | glyoxylate metabolic process | 9 |
| biological process | heart contraction | 9 |
| biological process | cytidine transport | 9 |
| biological process | chondroitin sulfate catabolic process | 9 |
| biological process | nucleobase catabolic process | 9 |
| biological process | cellular response to sulfate starvation | 9 |
| biological process | mitochondrial ATP synthesis coupled proton transport | 9 |
| biological process | DNA methylation on cytosine within a CG sequence | 9 |
| biological process | poly(A)+ mRNA export from nucleus | 9 |
| biological process | mRNA splicing, via spliceosome | 9 |
| biological process | locomotion | 9 |
| biological process | regulation of carbohydrate catabolic process | 9 |
| biological process | chaperone cofactor-dependent protein refolding | 9 |
| biological process | peptidyl-methionine modification | 9 |
| biological process | positive regulation of DNA-templated transcription, initiation | 9 |
| biological process | genetic transfer | 9 |
| biological process | ferric iron import | 9 |
| biological process | central nervous system development | 9 |
| biological process | response to organic substance | 9 |
| biological process | organic substance metabolic process | 9 |
| biological process | serine transport | 9 |
| biological process | regulation of cell proliferation | 9 |
| biological process | gamma-aminobutyric acid signaling pathway | 9 |
| biological process | cartilage condensation | 8 |
| biological process | ethanol biosynthetic process | 8 |
| biological process | response to methylglyoxal | 8 |
| biological process | intron homing | 8 |
| biological process | GMP metabolic process | 8 |
| biological process | negative regulation of protein polymerization | 8 |
| biological process | protein hexamerization | 8 |
| biological process | cellular response to arsenic-containing substance | 8 |
| biological process | acetate metabolic process | 8 |
| biological process | proline catabolic process to glutamate | 8 |
| biological process | gamete generation | 8 |
| biological process | selenocysteine catabolic process | 8 |
| biological process | sensory perception of pain | 8 |
| biological process | aspartate family amino acid metabolic process | 8 |
| biological process | chondrocyte differentiation | 8 |
| biological process | maltose catabolic process | 8 |
| biological process | inositol catabolic process | 8 |
| biological process | D-galactonate catabolic process | 8 |
| biological process | negative regulation of GTPase activity | 8 |
| biological process | siderophore transmembrane transport | 8 |
| biological process | lactose transport | 8 |
| biological process | oxaloacetate metabolic process | 8 |
| biological process | 3,4-dihydroxybenzoate catabolic process | 8 |
| biological process | replication fork processing | 8 |
| biological process | arabinose catabolic process | 8 |
| biological process | cell-cell signaling | 8 |
| biological process | positive regulation of hydrolase activity | 8 |
| biological process | silver ion transport | 8 |
| biological process | alanine catabolic process | 8 |
| biological process | protein O-linked glycosylation | 8 |
| biological process | L-lysine transmembrane transport | 8 |
| biological process | positive regulation of protein kinase activity | 8 |
| biological process | regulation of reactive oxygen species metabolic process | 8 |
| biological process | protein initiator methionine removal involved in protein maturation | 8 |
| biological process | long-chain fatty acid transport | 8 |
| biological process | purine nucleoside metabolic process | 8 |
| biological process | neuromuscular process controlling balance | 8 |
| biological process | glycerol-3-phosphate biosynthetic process | 8 |
| biological process | guanine catabolic process | 8 |
| biological process | positive regulation of GTPase activity | 8 |
| biological process | N-acetylmannosamine metabolic process | 8 |
| biological process | asparagine catabolic process | 8 |
| biological process | selenocysteine biosynthetic process | 8 |
| biological process | viral release from host cell | 8 |
| biological process | tyrosine transport | 8 |
| biological process | nucleoside monophosphate metabolic process | 8 |
| biological process | pyrroloquinoline quinone biosynthetic process | 8 |
| biological process | cytosine transport | 8 |
| biological process | arsenite transport | 8 |
| biological process | carbohydrate derivative transport | 8 |
| biological process | striated muscle myosin thick filament assembly | 8 |
| biological process | ER-associated ubiquitin-dependent protein catabolic process | 8 |
| biological process | eye morphogenesis | 8 |
| biological process | negative regulation of endodermal cell differentiation | 8 |
| biological process | iron ion transmembrane transport | 8 |
| biological process | 3-(3-hydroxy)phenylpropionate catabolic process | 8 |
| biological process | nucleobase-containing small molecule metabolic process | 8 |
| biological process | NADH metabolic process | 8 |
| biological process | negative regulation of secondary metabolite biosynthetic process | 8 |
| biological process | cell proliferation | 8 |
| biological process | cell envelope organization | 8 |
| biological process | clearance of foreign intracellular DNA | 8 |
| biological process | cellular response to cadmium ion | 8 |
| biological process | collagen catabolic process | 8 |
| biological process | oxidative phosphorylation | 8 |
| biological process | regulation of apoptotic process | 8 |
| biological process | heart development | 8 |
| biological process | hyphal growth | 8 |
| biological process | actin filament-based movement | 8 |
| biological process | trehalose biosynthetic process | 8 |
| biological process | pyrimidine nucleoside metabolic process | 8 |
| biological process | innate immune response | 8 |
| biological process | biomineral tissue development | 8 |
| biological process | sterigmatocystin biosynthetic process | 8 |
| biological process | AMP metabolic process | 8 |
| biological process | cell septum assembly | 8 |
| biological process | nucleoside triphosphate metabolic process | 8 |
| biological process | bacterial-type flagellum-dependent swimming motility | 8 |
| biological process | inner ear morphogenesis | 8 |
| biological process | endodermal cell differentiation | 8 |
| biological process | N-glycan processing | 8 |
| biological process | G-protein coupled receptor signaling pathway | 8 |
| biological process | rRNA 3'-end processing | 8 |
| biological process | rescue of stalled ribosome | 8 |
| biological process | skeletal muscle myosin thick filament assembly | 7 |
| biological process | L-arabinose catabolic process to xylulose 5-phosphate | 7 |
| biological process | positive regulation of carbohydrate metabolic process | 7 |
| biological process | mitotic spindle organization | 7 |
| biological process | cellular response to zinc ion starvation | 7 |
| biological process | phagocytosis, engulfment | 7 |
| biological process | adenine transport | 7 |
| biological process | negative regulation of extrinsic apoptotic signaling pathway in absence of ligand | 7 |
| biological process | positive regulation of cell proliferation | 7 |
| biological process | polyol catabolic process | 7 |
| biological process | sterol metabolic process | 7 |
| biological process | histone acetylation | 7 |
| biological process | positive regulation of apoptotic process | 7 |
| biological process | inositol biosynthetic process | 7 |
| biological process | cellular protein localization | 7 |
| biological process | histidine transport | 7 |
| biological process | regulation of cardiac muscle cell membrane potential | 7 |
| biological process | DNA replication, Okazaki fragment processing | 7 |
| biological process | phosphatidylserine catabolic process | 7 |
| biological process | regulation of translational initiation | 7 |
| biological process | tissue homeostasis | 7 |
| biological process | lipid-linked peptidoglycan transport | 7 |
| biological process | amino sugar catabolic process | 7 |
| biological process | antimonite transport | 7 |
| biological process | N-acetylmuramic acid metabolic process | 7 |
| biological process | skin morphogenesis | 7 |
| biological process | phosphatidylinositol-mediated signaling | 7 |
| biological process | negative regulation of angiogenesis | 7 |
| biological process | purine nucleobase catabolic process | 7 |
| biological process | tendon development | 7 |
| biological process | glycolytic process through fructose-6-phosphate | 7 |
| biological process | inner ear receptor cell development | 7 |
| biological process | positive regulation of glycogen catabolic process | 7 |
| biological process | GDP metabolic process | 7 |
| biological process | osmosensory signaling via phosphorelay pathway | 7 |
| biological process | regulation of cell communication by electrical coupling | 7 |
| biological process | coenzyme biosynthetic process | 7 |
| biological process | cellular process | 7 |
| biological process | cell projection organization | 7 |
| biological process | protein-pyridoxal-5-phosphate linkage via peptidyl-N6-pyridoxal phosphate-L-lysine | 7 |
| biological process | citrate transport | 7 |
| biological process | cell morphogenesis involved in conjugation with cellular fusion | 7 |
| biological process | palate development | 7 |
| biological process | protein polymerization | 7 |
| biological process | deoxyguanosine catabolic process | 7 |
| biological process | regulation of actin cytoskeleton organization | 7 |
| biological process | chloroplast organization | 7 |
| biological process | cellular response to reactive oxygen species | 7 |
| biological process | defense response to fungus | 7 |
| biological process | protein import into nucleus | 7 |
| biological process | peptidoglycan-protein cross-linking | 7 |
| biological process | piRNA metabolic process | 7 |
| biological process | neutral amino acid transport | 7 |
| biological process | vulval development | 7 |
| biological process | IMP salvage | 7 |
| biological process | mycolic acid biosynthetic process | 7 |
| biological process | detection of virus | 7 |
| biological process | nuclear-transcribed mRNA catabolic process, endonucleolytic cleavage-dependent decay | 7 |
| biological process | vasculogenesis | 7 |
| biological process | nonribosomal peptide biosynthetic process | 7 |
| biological process | L-alanine biosynthetic process from pyruvate | 7 |
| biological process | myofibril assembly | 7 |
| biological process | 2-oxobutyrate biosynthetic process | 7 |
| biological process | organic phosphonate metabolic process | 7 |
| biological process | lagging strand elongation | 7 |
| biological process | multicellular pellicle formation | 7 |
| biological process | lipopolysaccharide-mediated virion attachment to host cell | 7 |
| biological process | histone exchange | 7 |
| biological process | microtubule bundle formation | 7 |
| biological process | negative regulation of cell proliferation | 7 |
| biological process | mycolate cell wall layer assembly | 7 |
| biological process | carnitine transport | 7 |
| biological process | cellular hyperosmotic response | 7 |
| biological process | peptidyl-tyrosine phosphorylation | 7 |
| biological process | induction by symbiont of host defense response | 7 |
| biological process | plasmid partitioning | 7 |
| biological process | DNA catabolic process, endonucleolytic | 7 |
| biological process | urea transmembrane transport | 7 |
| biological process | urate transport | 7 |
| biological process | middle ear morphogenesis | 7 |
| biological process | tRNA nucleoside ribose methylation | 7 |
| biological process | ribose phosphate metabolic process | 7 |
| biological process | cellular response to superoxide | 7 |
| biological process | xenobiotic transport | 7 |
| biological process | muscle organ development | 7 |
| biological process | cysteine export | 7 |
| biological process | tRNA 3'-terminal CCA addition | 7 |
| biological process | urea catabolic process | 7 |
| biological process | peptide cross-linking | 7 |
| biological process | tricarboxylic acid transport | 7 |
| biological process | phototaxis | 7 |
| biological process | regulation of mitotic metaphase/anaphase transition | 7 |
| biological process | muscle attachment | 7 |
| biological process | dimethylallyl diphosphate biosynthetic process | 7 |
| biological process | alkane catabolic process | 7 |
| biological process | resolution of recombination intermediates | 7 |
| biological process | cellular carbohydrate catabolic process | 7 |
| biological process | 'de novo' CTP biosynthetic process | 7 |
| biological process | DNA packaging | 7 |
| biological process | response to host | 7 |
| biological process | translesion synthesis | 7 |
| biological process | cell chemotaxis | 7 |
| biological process | ossification | 7 |
| biological process | axon extension | 7 |
| biological process | starch metabolic process | 7 |
| biological process | triglyceride biosynthetic process | 7 |
| biological process | cellular response to BMP stimulus | 7 |
| biological process | embryonic skeletal joint morphogenesis | 7 |
| biological process | regulation of release of sequestered calcium ion into cytosol by sarcoplasmic reticulum | 7 |
| biological process | positive regulation of ryanodine-sensitive calcium-release channel activity | 7 |
| biological process | ammonium transmembrane transport | 7 |
| biological process | lipopolysaccharide metabolic process | 7 |
| biological process | tripeptide transport | 7 |
| biological process | ribosome disassembly | 6 |
| biological process | TOR signaling | 6 |
| biological process | colicin transport | 6 |
| biological process | ATP generation from ADP | 6 |
| biological process | oxalate catabolic process | 6 |
| biological process | tripeptide transmembrane transport | 6 |
| biological process | mitotic sister chromatid cohesion | 6 |
| biological process | glycine biosynthetic process, by transamination of glyoxylate | 6 |
| biological process | otic vesicle development | 6 |
| biological process | cerebral cortex development | 6 |
| biological process | positive regulation of I-kappaB kinase/NF-kappaB signaling | 6 |
| biological process | determination of adult lifespan | 6 |
| biological process | response to estrogen | 6 |
| biological process | regulation of glutamine family amino acid metabolic process | 6 |
| biological process | replicative cell aging | 6 |
| biological process | cardiac myofibril assembly | 6 |
| biological process | ornithine transport | 6 |
| biological process | multicellular organism reproduction | 6 |
| biological process | phototransduction | 6 |
| biological process | formaldehyde catabolic process | 6 |
| biological process | nucleoside diphosphate phosphorylation | 6 |
| biological process | positive regulation of lipid biosynthetic process | 6 |
| biological process | lamellipodium assembly | 6 |
| biological process | response to amino acid | 6 |
| biological process | axonogenesis | 6 |
| biological process | positive regulation of cellular response to phosphate starvation | 6 |
| biological process | establishment of monopolar cell polarity | 6 |
| biological process | wound healing, spreading of epidermal cells | 6 |
| biological process | single-species biofilm formation in or on host organism | 6 |
| biological process | bacteriocin biosynthetic process | 6 |
| biological process | sulfathiazole transmembrane transport | 6 |
| biological process | response to water deprivation | 6 |
| biological process | Kdo2-lipid A biosynthetic process | 6 |
| biological process | elastic fiber assembly | 6 |
| biological process | nucleoside biosynthetic process | 6 |
| biological process | proline catabolic process | 6 |
| biological process | locomotory behavior | 6 |
| biological process | regulation of ubiquinone biosynthetic process | 6 |
| biological process | peptidyl-lysine lipoylation | 6 |
| biological process | cellular catabolic process | 6 |
| biological process | proteasome-mediated ubiquitin-dependent protein catabolic process | 6 |
| biological process | nucleus organization | 6 |
| biological process | detection of chemical stimulus involved in sensory perception of smell | 6 |
| biological process | photoreceptor cell outer segment organization | 6 |
| biological process | brain development | 6 |
| biological process | regulation of barrier septum assembly | 6 |
| biological process | lipid storage | 6 |
| biological process | negative regulation of transposon integration | 6 |
| biological process | adult heart development | 6 |
| biological process | limb bud formation | 6 |
| biological process | cell-substrate adhesion | 6 |
| biological process | amino acid activation for nonribosomal peptide biosynthetic process | 6 |
| biological process | histone H2A acetylation | 6 |
| biological process | filamentous growth of a population of unicellular organisms in response to biotic stimulus | 6 |
| biological process | negative regulation of intracellular transport | 6 |
| biological process | mRNA cleavage | 6 |
| biological process | trans-translation | 6 |
| biological process | transcription-coupled nucleotide-excision repair | 6 |
| biological process | cellular response to unfolded protein | 6 |
| biological process | protein sumoylation | 6 |
| biological process | arabinan catabolic process | 6 |
| biological process | response to ammonium ion | 6 |
| biological process | regulation of nitrate assimilation | 6 |
| biological process | mitochondrion transport along microtubule | 6 |
| biological process | secondary metabolic process | 6 |
| biological process | cellular protein metabolic process | 6 |
| biological process | otic placode formation | 6 |
| biological process | detection of gravity | 6 |
| biological process | protein deubiquitination | 6 |
| biological process | tryptophan catabolic process | 6 |
| biological process | regulation of oxygen metabolic process | 6 |
| biological process | chromatin silencing at silent mating-type cassette | 6 |
| biological process | activation of protein kinase B activity | 6 |
| biological process | nucleoside triphosphate catabolic process | 6 |
| biological process | phosphatidylcholine biosynthetic process | 6 |
| biological process | L-arginine transport | 6 |
| biological process | fatty acid elongation, saturated fatty acid | 6 |
| biological process | reactive oxygen species metabolic process | 6 |
| biological process | histone H4 acetylation | 6 |
| biological process | nuclear migration | 6 |
| biological process | IMP biosynthetic process | 6 |
| biological process | cellular lipid metabolic process | 6 |
| biological process | regulation of cellular component organization | 6 |
| biological process | stress response to metal ion | 6 |
| biological process | cell gliding | 6 |
| biological process | cellular response to carbohydrate stimulus | 6 |
| biological process | negative regulation of DNA replication | 6 |
| biological process | organic cation transport | 6 |
| biological process | regulation of systemic acquired resistance | 6 |
| biological process | melibiose catabolic process | 6 |
| biological process | hepatocyte proliferation | 6 |
| biological process | response to superoxide | 6 |
| biological process | wobble base lysidine biosynthesis | 6 |
| biological process | nylon catabolic process | 6 |
| biological process | response to radiation | 6 |
| biological process | negative regulation of protein catabolic process | 6 |
| biological process | protein biotinylation | 6 |
| biological process | heme catabolic process | 6 |
| biological process | integrin biosynthetic process | 6 |
| biological process | dendrite development | 6 |
| biological process | pyruvate catabolic process | 6 |
| biological process | regulation of GTPase activity | 6 |
| biological process | transcription-coupled nucleotide-excision repair, DNA damage recognition | 6 |
| biological process | aromatic amino acid family metabolic process | 6 |
| biological process | cytoplasmic microtubule organization | 6 |
| biological process | protein transmembrane transport | 6 |
| biological process | actomyosin structure organization | 6 |
| biological process | positive regulation of ATPase activity | 6 |
| biological process | oxazole or thiazole biosynthetic process | 6 |
| biological process | filamentous growth of a population of unicellular organisms in response to starvation | 6 |
| biological process | cortical actin cytoskeleton organization | 6 |
| biological process | lysine transport | 6 |
| biological process | neuron projection development | 6 |
| biological process | negative regulation of endoribonuclease activity | 6 |
| biological process | protein-chromophore linkage | 6 |
| biological process | macromolecule modification | 6 |
| biological process | amine transport | 6 |
| biological process | positive regulation of DNA binding | 6 |
| biological process | erythrocyte homeostasis | 6 |
| biological process | cellular response to nitrogen starvation | 6 |
| biological process | digestive tract development | 6 |
| biological process | vitamin B6 biosynthetic process | 6 |
| biological process | regulation of neuron projection development | 6 |
| biological process | heme oxidation | 6 |
| biological process | protein initiator methionine removal | 6 |
| biological process | polyphosphate biosynthetic process | 6 |
| biological process | nitric oxide catabolic process | 6 |
| biological process | apoptotic signaling pathway | 6 |
| biological process | positive regulation of protein ubiquitination | 5 |
| biological process | response to virus | 5 |
| biological process | late endosome to Golgi transport | 5 |
| biological process | water transport | 5 |
| biological process | hexose transmembrane transport | 5 |
| biological process | aerobic cobalamin biosynthetic process | 5 |
| biological process | divalent inorganic cation transport | 5 |
| biological process | mitochondrial DNA repair | 5 |
| biological process | amino-acid betaine biosynthetic process | 5 |
| biological process | inorganic anion transport | 5 |
| biological process | cell wall polysaccharide biosynthetic process | 5 |
| biological process | nervous system development | 5 |
| biological process | liver development | 5 |
| biological process | shikimate transport | 5 |
| biological process | asparagine catabolic process via L-aspartate | 5 |
| biological process | regulation of protein kinase activity | 5 |
| biological process | ribonucleoside catabolic process | 5 |
| biological process | cellular aldehyde metabolic process | 5 |
| biological process | cellular response to nitric oxide | 5 |
| biological process | contractile vacuole organization | 5 |
| biological process | nuclear-transcribed mRNA catabolic process, deadenylation-dependent decay | 5 |
| biological process | actin filament depolymerization | 5 |
| biological process | seed development | 5 |
| biological process | fatty acid beta-oxidation, unsaturated, even number, reductase/isomerase pathway | 5 |
| biological process | execution phase of apoptosis | 5 |
| biological process | hippo signaling | 5 |
| biological process | response to water | 5 |
| biological process | Golgi ribbon formation | 5 |
| biological process | negative regulation of hydrolase activity | 5 |
| biological process | protein import into mitochondrial matrix | 5 |
| biological process | canonical glycolysis | 5 |
| biological process | regulation of cell migration | 5 |
| biological process | charged-tRNA amino acid modification | 5 |
| biological process | regulation of DNA-dependent DNA replication initiation | 5 |
| biological process | establishment of planar polarity | 5 |
| biological process | tRNA seleno-modification | 5 |
| biological process | L-methionine biosynthetic process from S-adenosylmethionine | 5 |
| biological process | cell wall macromolecule metabolic process | 5 |
| biological process | positive regulation of ion transmembrane transporter activity | 5 |
| biological process | tRNA wobble cytosine modification | 5 |
| biological process | neurofilament bundle assembly | 5 |
| biological process | sorbitol transport | 5 |
| biological process | tellurite transport | 5 |
| biological process | actin filament bundle assembly | 5 |
| biological process | adenosine biosynthetic process | 5 |
| biological process | galactolipid metabolic process | 5 |
| biological process | monocarboxylic acid transport | 5 |
| biological process | ethanol oxidation | 5 |
| biological process | mannosylglycerate transport | 5 |
| biological process | lignin biosynthetic process | 5 |
| biological process | quinate catabolic process | 5 |
| biological process | negative regulation of smooth muscle cell proliferation | 5 |
| biological process | negative regulation of extrinsic apoptotic signaling pathway via death domain receptors | 5 |
| biological process | regulation of response to reactive oxygen species | 5 |
| biological process | aspartate transport | 5 |
| biological process | recycling endosome to Golgi transport | 5 |
| biological process | cellular response to nitrate | 5 |
| biological process | aorta smooth muscle tissue morphogenesis | 5 |
| biological process | establishment of cell polarity | 5 |
| biological process | positive regulation of transport | 5 |
| biological process | negative regulation of translation, ncRNA-mediated | 5 |
| biological process | tRNA acetylation | 5 |
| biological process | viral genome ejection through host cell envelope | 5 |
| biological process | hypoxanthine oxidation | 5 |
| biological process | protein targeting to lysosome | 5 |
| biological process | establishment of cell polarity involved in ameboidal cell migration | 5 |
| biological process | siderophore biosynthetic process from catechol | 5 |
| biological process | pollen development | 5 |
| biological process | organic acid metabolic process | 5 |
| biological process | protein lipidation | 5 |
| biological process | plasmid recombination | 5 |
| biological process | regulation of cytosolic calcium ion concentration | 5 |
| biological process | embryo development ending in birth or egg hatching | 5 |
| biological process | detection of chemical stimulus | 5 |
| biological process | homoserine metabolic process | 5 |
| biological process | iron chelate transport | 5 |
| biological process | sulfur compound catabolic process | 5 |
| biological process | enterobactin transport | 5 |
| biological process | N-acetylmannosamine catabolic process | 5 |
| biological process | corrin metabolic process | 5 |
| biological process | positive regulation of protein catabolic process | 5 |
| biological process | AMP biosynthetic process | 5 |
| biological process | termination of RNA polymerase II transcription | 5 |
| biological process | peptidyl-lysine hydroxylation involved in bacterial-type EF-P lysine modification | 5 |
| biological process | pyridoxine metabolic process | 5 |
| biological process | response to cytokine | 5 |
| biological process | propionate metabolic process | 5 |
| biological process | asexual reproduction | 5 |
| biological process | arabinose transport | 5 |
| biological process | DNA double-strand break processing | 5 |
| biological process | aldonic acid catabolic process | 5 |
| biological process | rolling circle DNA replication | 5 |
| biological process | 'de novo' L-methionine biosynthetic process | 5 |
| biological process | response to nicotine | 5 |
| biological process | bacterial-type DNA replication | 5 |
| biological process | disaccharide biosynthetic process | 5 |
| biological process | intermediate filament cytoskeleton organization | 5 |
| biological process | regulation of transcription from RNA polymerase II promoter in response to iron | 5 |
| biological process | osteoblast differentiation | 5 |
| biological process | positive regulation of smooth muscle cell proliferation | 5 |
| biological process | putrescine biosynthetic process | 5 |
| biological process | organic substance transport | 5 |
| biological process | peptidyl-lysine trimethylation | 5 |
| biological process | cellular response to magnesium ion | 5 |
| biological process | wound healing involved in inflammatory response | 5 |
| biological process | pyoverdine biosynthetic process | 5 |
| biological process | positive regulation of translation, ncRNA-mediated | 5 |
| biological process | regulation of glyoxylate cycle | 5 |
| biological process | cellular potassium ion homeostasis | 5 |
| biological process | xanthosine transport | 5 |
| biological process | glycerophospholipid metabolic process | 5 |
| biological process | glycolipid translocation | 5 |
| biological process | cellular response to nitrite | 5 |
| biological process | regulation of small GTPase mediated signal transduction | 5 |
| biological process | deoxyribonucleoside diphosphate metabolic process | 5 |
| biological process | positive regulation of protein phosphorylation | 5 |
| biological process | monocyte differentiation | 5 |
| biological process | microtubule organizing center organization | 5 |
| biological process | pantothenate transmembrane transport | 5 |
| biological process | N-terminal peptidyl-alanine trimethylation | 5 |
| biological process | cell volume homeostasis | 5 |
| biological process | extracellular polysaccharide metabolic process | 5 |
| biological process | positive regulation of Rho protein signal transduction | 5 |
| biological process | ammonium transport | 5 |
| biological process | conversion of methionyl-tRNA to N-formyl-methionyl-tRNA | 5 |
| biological process | sulfathiazole transport | 5 |
| biological process | protein destabilization | 5 |
| biological process | retrograde transport, endosome to Golgi | 5 |
| biological process | GTP biosynthetic process | 5 |
| biological process | apoptotic DNA fragmentation | 5 |
| biological process | CTP biosynthetic process | 5 |
| biological process | copper ion transmembrane transport | 5 |
| biological process | ubiquinone-6 biosynthetic process | 5 |
| biological process | positive regulation of blood vessel endothelial cell migration | 5 |
| biological process | mitotic spindle elongation | 5 |
| biological process | cellular stress response to acid chemical | 5 |
| biological process | cuticle development | 5 |
| biological process | actin-myosin filament sliding | 5 |
| biological process | dUMP catabolic process | 5 |
| biological process | microtubule anchoring | 5 |
| biological process | maintenance of stationary phase | 5 |
| biological process | integrin-mediated signaling pathway | 5 |
| biological process | positive regulation of oxidoreductase activity | 5 |
| biological process | Okazaki fragment processing involved in mitotic DNA replication | 5 |
| biological process | mRNA metabolic process | 5 |
| biological process | isoprenoid biosynthetic process via mevalonate | 5 |
| biological process | phenylpropanoid catabolic process | 5 |
| biological process | response to high light intensity | 5 |
| biological process | cell wall mannoprotein biosynthetic process | 5 |
| biological process | cellular biogenic amine metabolic process | 5 |
| biological process | extracellular transport | 5 |
| biological process | flower development | 5 |
| biological process | nucleobase transport | 5 |
| biological process | GMP salvage | 5 |
| biological process | L-alanine biosynthetic process | 5 |
| biological process | centriole-centriole cohesion | 5 |
| biological process | L-glutamate biosynthetic process | 5 |
| biological process | centrosome organization | 5 |
| biological process | DNA strand renaturation | 5 |
| biological process | replication fork progression beyond termination site | 5 |
| biological process | regulation of protein phosphorylation | 5 |
| biological process | microtubule nucleation | 5 |
| biological process | regulation of protein exit from endoplasmic reticulum | 5 |
| biological process | ascospore wall assembly | 5 |
| biological process | heme metabolic process | 5 |
| biological process | cilium-dependent cell motility | 5 |
| biological process | gene silencing | 5 |
| biological process | glomerular basement membrane development | 5 |
| biological process | sorbitol catabolic process | 5 |
| biological process | cellular response to transforming growth factor beta stimulus | 5 |
| biological process | protein targeting to plasma membrane | 5 |
| biological process | GDP-L-fucose biosynthetic process | 5 |
| biological process | cellular response to biotic stimulus | 5 |
| biological process | negative regulation of protein secretion | 5 |
| biological process | Rho protein signal transduction | 5 |
| biological process | negative regulation of neuron migration | 5 |
| biological process | skeletal muscle contraction | 5 |
| biological process | regulation of histone H3-K4 methylation | 5 |
| biological process | blood vessel endothelial cell migration | 5 |
| biological process | cAMP-mediated signaling | 5 |
| biological process | zinc II ion transmembrane import | 4 |
| biological process | glucan metabolic process | 4 |
| biological process | cell migration involved in gastrulation | 4 |
| biological process | positive regulation of proteasomal ubiquitin-dependent protein catabolic process | 4 |
| biological process | aorta development | 4 |
| biological process | 3'-phosphoadenosine 5'-phosphosulfate metabolic process | 4 |
| biological process | protein S-linked glycosylation via cysteine | 4 |
| biological process | DNA-templated viral transcription | 4 |
| biological process | canonical Wnt signaling pathway | 4 |
| biological process | pyrimidine nucleobase biosynthetic process | 4 |
| biological process | resolution of meiotic recombination intermediates | 4 |
| biological process | carotenoid biosynthetic process | 4 |
| biological process | lung epithelial cell differentiation | 4 |
| biological process | cardiac muscle contraction | 4 |
| biological process | sleep | 4 |
| biological process | UDP-N-acetylgalactosamine biosynthetic process | 4 |
| biological process | cellular response to mercury ion | 4 |
| biological process | cellular response to magnesium starvation | 4 |
| biological process | organic anion transport | 4 |
| biological process | Roundabout signaling pathway | 4 |
| biological process | cellular response to glucose stimulus | 4 |
| biological process | vegetative to reproductive phase transition of meristem | 4 |
| biological process | positive regulation of proteolysis | 4 |
| biological process | positive regulation of cell growth | 4 |
| biological process | negative regulation of glycolytic process | 4 |
| biological process | regulation of chlorophyll biosynthetic process | 4 |
| biological process | cytokinin-activated signaling pathway | 4 |
| biological process | response to chromate | 4 |
| biological process | suppression by virus of host translation | 4 |
| biological process | negative chemotaxis | 4 |
| biological process | spermatid development | 4 |
| biological process | regulation of defense response to virus by host | 4 |
| biological process | cilium movement | 4 |
| biological process | post-embryonic development | 4 |
| biological process | positive regulation of embryonic development | 4 |
| biological process | G2/M transition of mitotic cell cycle | 4 |
| biological process | cytoplasmic actin-based contraction involved in forward cell motility | 4 |
| biological process | hexose transport | 4 |
| biological process | NADP metabolic process | 4 |
| biological process | adenosine metabolic process | 4 |
| biological process | regulation of transferase activity | 4 |
| biological process | regulation of axonogenesis | 4 |
| biological process | growth of symbiont in host organelle | 4 |
| biological process | manganese ion transport | 4 |
| biological process | chaperone-mediated protein transport across periplasmic space | 4 |
| biological process | inosine catabolic process | 4 |
| biological process | border follicle cell migration | 4 |
| biological process | ketogluconate metabolic process | 4 |
| biological process | response to glucose | 4 |
| biological process | regulation of mitotic nuclear division | 4 |
| biological process | regulation of growth | 4 |
| biological process | SMAD protein signal transduction | 4 |
| biological process | long-chain fatty-acyl-CoA metabolic process | 4 |
| biological process | regulation of cellular component movement | 4 |
| biological process | positive regulation of intracellular protein transport | 4 |
| biological process | glycine biosynthetic process from serine | 4 |
| biological process | regulation of viral process | 4 |
| biological process | taurine transport | 4 |
| biological process | nuclear migration along microtubule | 4 |
| biological process | lysophospholipid transport | 4 |
| biological process | filopodium assembly | 4 |
| biological process | D-glycero-D-manno-heptose 7-phosphate biosynthetic process | 4 |
| biological process | tRNA dihydrouridine synthesis | 4 |
| biological process | establishment of localization in cell | 4 |
| biological process | fatty acid oxidation | 4 |
| biological process | myoblast fusion | 4 |
| biological process | regulation of cell motility | 4 |
| biological process | wax biosynthetic process | 4 |
| biological process | 2,4,6-trinitrotoluene catabolic process | 4 |
| biological process | sphingolipid metabolic process | 4 |
| biological process | oligosaccharide transport | 4 |
| biological process | cellular response to zinc ion | 4 |
| biological process | intra-Golgi vesicle-mediated transport | 4 |
| biological process | lactate transmembrane transport | 4 |
| biological process | denitrification pathway | 4 |
| biological process | response to alkyl hydroperoxide | 4 |
| biological process | regulation of signal transduction | 4 |
| biological process | isopeptide cross-linking via N6-(L-isoglutamyl)-L-lysine | 4 |
| biological process | cyanate catabolic process | 4 |
| biological process | RNA polyadenylation | 4 |
| biological process | negative regulation of neuron death | 4 |
| biological process | sodium-dependent phosphate transport | 4 |
| biological process | microtubule nucleation by spindle pole body | 4 |
| biological process | bleb assembly | 4 |
| biological process | regulation of G2/M transition of mitotic cell cycle | 4 |
| biological process | regulation of ATPase activity | 4 |
| biological process | toxin catabolic process | 4 |
| biological process | cellular response to selenite ion | 4 |
| biological process | pseudopodium retraction | 4 |
| biological process | meiotic cell cycle | 4 |
| biological process | nicotinamide metabolic process | 4 |
| biological process | response to methotrexate | 4 |
| biological process | IMP catabolic process | 4 |
| biological process | response to fatty acid | 4 |
| biological process | regulation of organelle transport along microtubule | 4 |
| biological process | lactate catabolic process | 4 |
| biological process | posttranslational protein targeting to membrane | 4 |
| biological process | rhythmic process | 4 |
| biological process | hypotonic response | 4 |
| biological process | quinolinate biosynthetic process | 4 |
| biological process | biological adhesion | 4 |
| biological process | internal peptidyl-lysine acetylation | 4 |
| biological process | chromatin silencing at rDNA | 4 |
| biological process | chemotaxis to cAMP | 4 |
| biological process | terpenoid biosynthetic process, mevalonate-independent | 4 |
| biological process | negative regulation of actin filament polymerization | 4 |
| biological process | establishment of nucleus localization | 4 |
| biological process | protein palmitoylation | 4 |
| biological process | vacuole fusion, non-autophagic | 4 |
| biological process | lysine biosynthetic process via diaminopimelate, diaminopimelate-aminotransferase pathway | 4 |
| biological process | formaldehyde metabolic process | 4 |
| biological process | succinate transmembrane transport | 4 |
| biological process | negative regulation of cytoplasmic translation | 4 |
| biological process | protein complex disassembly | 4 |
| biological process | triglyceride metabolic process | 4 |
| biological process | protein heterotetramerization | 4 |
| biological process | negative regulation of lipid biosynthetic process | 4 |
| biological process | DNA replication-dependent nucleosome assembly | 4 |
| biological process | negative regulation of vascular permeability | 4 |
| biological process | peptidoglycan-associated peptide transport | 4 |
| biological process | galactomannan catabolic process | 4 |
| biological process | regulation of endoribonuclease activity | 4 |
| biological process | respiratory chain complex IV assembly | 4 |
| biological process | aerobactin biosynthetic process | 4 |
| biological process | sensory perception of smell | 4 |
| biological process | regulation of bacterial-type flagellum assembly | 4 |
| biological process | carboxylic acid transport | 4 |
| biological process | glycolate transmembrane transport | 4 |
| biological process | positive regulation of metabolic process | 4 |
| biological process | intraciliary transport | 4 |
| biological process | kidney development | 4 |
| biological process | 1,6-anhydro-N-acetyl-beta-muramic acid catabolic process | 4 |
| biological process | response to stimulus | 4 |
| biological process | positive regulation of mRNA cleavage | 4 |
| biological process | ventricular cardiac muscle tissue morphogenesis | 4 |
| biological process | nonmotile primary cilium assembly | 4 |
| biological process | regulation of tube length, open tracheal system | 4 |
| biological process | inositol lipid-mediated signaling | 4 |
| biological process | positive regulation of protein secretion | 4 |
| biological process | Mo-molybdopterin cofactor metabolic process | 4 |
| biological process | lipid glycosylation | 4 |
| biological process | epidermis development | 4 |
| biological process | negative regulation of bacterial-type flagellum assembly | 4 |
| biological process | pharyngeal pumping | 4 |
| biological process | cellular response to chromate | 4 |
| biological process | tRNA 4-thiouridine biosynthesis | 4 |
| biological process | bis(molybdopterin guanine dinucleotide)molybdenum biosynthetic process | 4 |
| biological process | XMP biosynthetic process | 4 |
| biological process | glomerular filtration | 4 |
| biological process | pollen tube growth | 4 |
| biological process | Wnt signaling pathway | 4 |
| biological process | 1-deoxy-D-xylulose 5-phosphate biosynthetic process | 4 |
| biological process | D-serine transport | 4 |
| biological process | actin cortical patch assembly | 4 |
| biological process | L-glutamate import | 4 |
| biological process | synapse assembly | 4 |
| biological process | kanamycin biosynthetic process | 4 |
| biological process | arabinan biosynthetic process | 4 |
| biological process | protein targeting to chloroplast | 4 |
| biological process | D-aspartate import | 4 |
| biological process | endonucleolytic cleavage involved in rRNA processing | 4 |
| biological process | uropod retraction | 4 |
| biological process | immune system process | 4 |
| biological process | tyrosine biosynthetic process from chorismate via 4-hydroxyphenylpyruvate | 4 |
| biological process | nuclear-transcribed mRNA catabolic process, 3'-5' exonucleolytic nonsense-mediated decay | 4 |
| biological process | tetrahydrofolylpolyglutamate biosynthetic process | 4 |
| biological process | positive regulation of biological process | 4 |
| biological process | response to external stimulus | 4 |
| biological process | phospholipid metabolic process | 4 |
| biological process | UDP-D-xylose biosynthetic process | 4 |
| biological process | peripheral nervous system neuron axonogenesis | 4 |
| biological process | phosphate ion homeostasis | 4 |
| biological process | single fertilization | 4 |
| biological process | phosphatidic acid biosynthetic process | 4 |
| biological process | DNA replication checkpoint | 4 |
| biological process | cell cycle arrest | 4 |
| biological process | maintenance of translational fidelity | 4 |
| biological process | phosphatidylcholine metabolic process | 4 |
| biological process | mRNA stabilization | 4 |
| biological process | negative regulation of chemotaxis | 4 |
| biological process | acetaldehyde catabolic process | 4 |
| biological process | anther dehiscence | 4 |
| biological process | D-amino acid metabolic process | 4 |
| biological process | Golgi organization | 4 |
| biological process | mitochondrial protein processing | 4 |
| biological process | inner dynein arm assembly | 4 |
| biological process | monosaccharide catabolic process | 4 |
| biological process | detection of maltose stimulus | 4 |
| biological process | thiamine salvage | 4 |
| biological process | visual behavior | 4 |
| biological process | ergosterol biosynthetic process | 4 |
| biological process | thiazole biosynthetic process | 4 |
| biological process | UDP biosynthetic process | 4 |
| biological process | nicotinate nucleotide biosynthetic process | 4 |
| biological process | filamentous growth of a population of unicellular organisms | 4 |
| biological process | regulation of ion transmembrane transport | 4 |
| biological process | negative regulation by symbiont of host immune response | 4 |
| biological process | myosin filament assembly | 4 |
| biological process | negative regulation of induction of conjugation with cellular fusion | 4 |
| biological process | ribonucleoside diphosphate catabolic process | 4 |
| biological process | contractile actin filament bundle assembly | 4 |
| biological process | interaction with host via substance in symbiont cell outer membrane | 4 |
| biological process | negative regulation of chromatin silencing | 4 |
| biological process | geranyl diphosphate biosynthetic process | 4 |
| biological process | nucleotide-sugar biosynthetic process | 4 |
| biological process | posttranscriptional regulation of gene expression | 4 |
| biological process | dihydrofolate biosynthetic process | 4 |
| biological process | regulation of protein secretion | 4 |
| biological process | cellular response to misfolded protein | 4 |
| biological process | protein secretion by the type VII secretion system | 4 |
| biological process | steroid biosynthetic process | 4 |
| biological process | uridine transport | 4 |
| biological process | endoplasmic reticulum organization | 4 |
| biological process | gastrulation with mouth forming second | 4 |
| biological process | mitochondrial DNA metabolic process | 4 |
| biological process | regulation of single-species biofilm formation on inanimate substrate | 4 |
| biological process | positive regulation of sporulation | 4 |
| biological process | deoxyribonucleoside monophosphate biosynthetic process | 4 |
| biological process | hematopoietic progenitor cell differentiation | 4 |
| biological process | regulation of glucose metabolic process | 4 |
| biological process | axon extension involved in axon guidance | 4 |
| biological process | tyrosine catabolic process | 4 |
| biological process | positive regulation of protein dephosphorylation | 4 |
| biological process | reproduction | 4 |
| biological process | pigment metabolic process involved in developmental pigmentation | 4 |
| biological process | response to hydrostatic pressure | 4 |
| biological process | positive regulation of potassium ion transmembrane transport | 4 |
| biological process | regulation of cell growth | 4 |
| biological process | response to peptide | 4 |
| biological process | peptidyl-lysine biotinylation | 4 |
| biological process | autophagosome assembly | 3 |
| biological process | chaperone-mediated protein transport | 3 |
| biological process | coronary vasculature development | 3 |
| biological process | rDNA condensation | 3 |
| biological process | negative regulation of glycogen biosynthetic process | 3 |
| biological process | axoneme assembly | 3 |
| biological process | regulation of response to osmotic stress | 3 |
| biological process | 4-amino-4-deoxy-alpha-L-arabinopyranosyl undecaprenyl phosphate biosynthetic process | 3 |
| biological process | chain elongation of O-linked mannose residue | 3 |
| biological process | cell-abiotic substrate adhesion | 3 |
| biological process | neuron migration | 3 |
| biological process | peptidyl-lysine demalonylation | 3 |
| biological process | alginic acid acetylation | 3 |
| biological process | mitochondrial DNA replication | 3 |
| biological process | positive regulation of phagocytosis | 3 |
| biological process | regulation of striated muscle tissue development | 3 |
| biological process | synaptonemal complex assembly | 3 |
| biological process | regulation of cellular pH | 3 |
| biological process | glycolipid transport | 3 |
| biological process | tRNA gene clustering | 3 |
| biological process | cadaverine transport | 3 |
| biological process | isoprenoid catabolic process | 3 |
| biological process | pentose-phosphate shunt, oxidative branch | 3 |
| biological process | protein urmylation | 3 |
| biological process | third ventricle development | 3 |
| biological process | histone H3 acetylation | 3 |
| biological process | L-ornithine transmembrane transport | 3 |
| biological process | negative regulation of protein modification process | 3 |
| biological process | fructose catabolic process | 3 |
| biological process | transsulfuration | 3 |
| biological process | negative regulation of gibberellic acid mediated signaling pathway | 3 |
| biological process | response to UV-B | 3 |
| biological process | detection of mechanical stimulus | 3 |
| biological process | pyrimidine nucleoside catabolic process | 3 |
| biological process | autophagic cell death | 3 |
| biological process | tooth mineralization | 3 |
| biological process | glycopeptide catabolic process | 3 |
| biological process | protein kinase C-activating G-protein coupled receptor signaling pathway | 3 |
| biological process | exonucleolytic trimming to generate mature 3'-end of 5.8S rRNA from tricistronic rRNA transcript (SSU-rRNA, 5.8S rRNA, LSU-rRNA) | 3 |
| biological process | response to leucine | 3 |
| biological process | dorsal/ventral axis specification | 3 |
| biological process | female pregnancy | 3 |
| biological process | regulation of polyamine biosynthetic process | 3 |
| biological process | deoxyinosine catabolic process | 3 |
| biological process | phenazine biosynthetic process | 3 |
| biological process | cardiac muscle tissue morphogenesis | 3 |
| biological process | protein targeting to mitochondrion | 3 |
| biological process | regulation of starch biosynthetic process | 3 |
| biological process | molting cycle process | 3 |
| biological process | glomerulus development | 3 |
| biological process | taurine catabolic process | 3 |
| biological process | homoserine transport | 3 |
| biological process | regulation of nitric oxide metabolic process | 3 |
| biological process | pyrimidine nucleobase salvage | 3 |
| biological process | guanine import into cell | 3 |
| biological process | beta-glucan biosynthetic process | 3 |
| biological process | cranial nerve development | 3 |
| biological process | response to sucrose | 3 |
| biological process | xanthosine catabolic process | 3 |
| biological process | 2-aminoethylphosphonate metabolic process | 3 |
| biological process | mRNA transport | 3 |
| biological process | response to organonitrogen compound | 3 |
| biological process | dorsal closure | 3 |
| biological process | protein localization to nuclear pore | 3 |
| biological process | regulation of protein localization | 3 |
| biological process | cellular response to desiccation | 3 |
| biological process | mitotic cell cycle arrest | 3 |
| biological process | fourth ventricle development | 3 |
| biological process | wing disc development | 3 |
| biological process | adenosine transport | 3 |
| biological process | abscisic acid-activated signaling pathway | 3 |
| biological process | cellular zinc ion homeostasis | 3 |
| biological process | tRNA guanine ribose methylation | 3 |
| biological process | positive regulation of growth | 3 |
| biological process | atrial cardiac muscle tissue morphogenesis | 3 |
| biological process | regulation of cell projection assembly | 3 |
| biological process | neuron cell-cell adhesion | 3 |
| biological process | lysine biosynthetic process via diaminopimelate and N-succinyl-2-amino-6-ketopimelate | 3 |
| biological process | 2-keto-3-deoxygluconate transport | 3 |
| biological process | polyphosphate catabolic process | 3 |
| biological process | cellular response to temperature stimulus | 3 |
| biological process | fatty acid beta-oxidation, unsaturated, even number | 3 |
| biological process | intramembranous ossification | 3 |
| biological process | cytolysis in other organism | 3 |
| biological process | cytochrome c-heme linkage | 3 |
| biological process | retrograde vesicle-mediated transport, Golgi to ER | 3 |
| biological process | establishment of protein localization to plasma membrane | 3 |
| biological process | skeletal muscle thin filament assembly | 3 |
| biological process | protein-heme linkage | 3 |
| biological process | brown fat cell differentiation | 3 |
| biological process | positive regulation of stress-activated MAPK cascade | 3 |
| biological process | epithelial cell differentiation | 3 |
| biological process | cellular response to cell envelope stress | 3 |
| biological process | guanosine pentaphosphate catabolic process | 3 |
| biological process | intermediate filament bundle assembly | 3 |
| biological process | protein localization to cilium | 3 |
| biological process | actin filament capping | 3 |
| biological process | positive regulation of chromosome segregation | 3 |
| biological process | protein secretion by the type I secretion system | 3 |
| biological process | iron incorporation into metallo-sulfur cluster | 3 |
| biological process | regulation of peptidase activity | 3 |
| biological process | evasion or tolerance of host defenses | 3 |
| biological process | positive phototaxis | 3 |
| biological process | UV-damage excision repair | 3 |
| biological process | cartilage development | 3 |
| biological process | guanine transmembrane transport | 3 |
| biological process | capsular polysaccharide transport | 3 |
| biological process | NAD metabolic process | 3 |
| biological process | actin cytoskeleton reorganization | 3 |
| biological process | UTP biosynthetic process | 3 |
| biological process | trehalose metabolism in response to cold stress | 3 |
| biological process | DNA replication proofreading | 3 |
| biological process | neurofilament cytoskeleton organization | 3 |
| biological process | uroporphyrinogen III biosynthetic process | 3 |
| biological process | positive regulation of single-species biofilm formation on inanimate substrate | 3 |
| biological process | regulation of heme biosynthetic process | 3 |
| biological process | dihydrofolate metabolic process | 3 |
| biological process | leaf morphogenesis | 3 |
| biological process | stress response to zinc ion | 3 |
| biological process | regulation of cell adhesion | 3 |
| biological process | XMP salvage | 3 |
| biological process | negative regulation of cell proliferation involved in contact inhibition | 3 |
| biological process | anaerobic amino acid catabolic process | 3 |
| biological process | nucleus-associated proteasomal ubiquitin-dependent protein catabolic process | 3 |
| biological process | gibberellic acid mediated signaling pathway | 3 |
| biological process | nematode larval development | 3 |
| biological process | eye photoreceptor cell development | 3 |
| biological process | regulation of protein glycosylation | 3 |
| biological process | negative regulation of ethylene-activated signaling pathway | 3 |
| biological process | centriole replication | 3 |
| biological process | regulation of cellular respiration | 3 |
| biological process | tRNA 5'-leader removal | 3 |
| biological process | regulation of the force of heart contraction | 3 |
| biological process | cell-cell signaling involved in quorum sensing | 3 |
| biological process | sperm motility | 3 |
| biological process | photosystem II repair | 3 |
| biological process | atrazine catabolic process to isopropylamine | 3 |
| biological process | pyridine nucleotide salvage | 3 |
| biological process | cysteine biosynthetic process via cystathionine | 3 |
| biological process | regulation of collagen catabolic process | 3 |
| biological process | response to jasmonic acid | 3 |
| biological process | mesoderm development | 3 |
| biological process | establishment of protein localization to Golgi | 3 |
| biological process | peptidyl-S-carbamoyl-L-cysteine dehydration | 3 |
| biological process | glycerophospholipid catabolic process | 3 |
| biological process | positive regulation of RNA splicing | 3 |
| biological process | mercury ion transport | 3 |
| biological process | ornithine metabolic process | 3 |
| biological process | Ras protein signal transduction | 3 |
| biological process | proline metabolic process | 3 |
| biological process | positive regulation of NF-kappaB import into nucleus | 3 |
| biological process | plasma membrane repair | 3 |
| biological process | rRNA pseudouridine synthesis | 3 |
| biological process | regulation of gluconeogenesis involved in cellular glucose homeostasis | 3 |
| biological process | chemotaxis to folate | 3 |
| biological process | positive regulation of peptidyl-serine phosphorylation | 3 |
| biological process | negative regulation of DNA topoisomerase (ATP-hydrolyzing) activity | 3 |
| biological process | positive regulation of epithelial to mesenchymal transition | 3 |
| biological process | cellular response to anoxia | 3 |
| biological process | dendrite morphogenesis | 3 |
| biological process | antibiotic catabolic process | 3 |
| biological process | response to red or far red light | 3 |
| biological process | dermatan sulfate catabolic process | 3 |
| biological process | regulation of rhodopsin mediated signaling pathway | 3 |
| biological process | circadian rhythm | 3 |
| biological process | nucleotide-excision repair, DNA incision, 5'-to lesion | 3 |
| biological process | inner ear development | 3 |
| biological process | valine transport | 3 |
| biological process | positive regulation of adenylate cyclase activity | 3 |
| biological process | fructoselysine metabolic process | 3 |
| biological process | protein carbamoylation | 3 |
| biological process | bone development | 3 |
| biological process | peptidyl-lysine deacetylation | 3 |
| biological process | regulation of lipid biosynthetic process | 3 |
| biological process | positive regulation of cell cycle arrest | 3 |
| biological process | positive regulation of cytokine secretion | 3 |
| biological process | establishment of organelle localization | 3 |
| biological process | mitochondrial electron transport, succinate to ubiquinone | 3 |
| biological process | regulation of DNA-templated transcription, termination | 3 |
| biological process | pyrimidine nucleoside transport | 3 |
| biological process | COPII vesicle coating | 3 |
| biological process | nucleotide-excision repair involved in interstrand cross-link repair | 3 |
| biological process | axon regeneration | 3 |
| biological process | tetracycline transport | 3 |
| biological process | peptidyl-serine phosphopantetheinylation | 3 |
| biological process | angiogenesis | 3 |
| biological process | aging | 3 |
| biological process | nucleosome positioning | 3 |
| biological process | regulation of cell-substrate adhesion | 3 |
| biological process | allantoin transport | 3 |
| biological process | meiotic chromosome condensation | 3 |
| biological process | actin filament polymerization | 3 |
| biological process | protein auto-ADP-ribosylation | 3 |
| biological process | negative regulation of cell-substrate adhesion | 3 |
| biological process | positive regulation of cell death | 3 |
| biological process | negative regulation of phagocytosis | 3 |
| biological process | regulation of heart contraction | 3 |
| biological process | fin development | 3 |
| biological process | protein localization to nucleus | 3 |
| biological process | nucleotide salvage | 3 |
| biological process | NLS-bearing protein import into nucleus | 3 |
| biological process | cadmium ion transport | 3 |
| biological process | histamine transport | 3 |
| biological process | response to carbon starvation | 3 |
| biological process | reductive pentose-phosphate cycle | 3 |
| biological process | glomerular capillary formation | 3 |
| biological process | auxin homeostasis | 3 |
| biological process | epithelial structure maintenance | 3 |
| biological process | karyogamy involved in conjugation with cellular fusion | 3 |
| biological process | nuclear retention of unspliced pre-mRNA at the site of transcription | 3 |
| biological process | negative regulation of protein import into nucleus during spindle assembly checkpoint | 3 |
| biological process | nucleosome disassembly | 3 |
| biological process | bacteriochlorophyll biosynthetic process | 3 |
| biological process | zinc ion homeostasis | 3 |
| biological process | ketone body catabolic process | 3 |
| biological process | epithelial cell development | 3 |
| biological process | negative regulation of peptidase activity | 3 |
| biological process | regulation of mitotic cell cycle | 3 |
| biological process | brassinosteroid biosynthetic process | 3 |
| biological process | cytosolic valyl-tRNA aminoacylation | 3 |
| biological process | purine ribonucleotide interconversion | 3 |
| biological process | auditory behavior | 3 |
| biological process | negative regulation of glucokinase activity | 3 |
| biological process | regulation of fatty acid biosynthetic process | 3 |
| biological process | penicillin biosynthetic process | 3 |
| biological process | face morphogenesis | 3 |
| biological process | protein ADP-ribosylation | 3 |
| biological process | sphingosine biosynthetic process | 3 |
| biological process | positive regulation of canonical Wnt signaling pathway | 3 |
| biological process | thymine metabolic process | 3 |
| biological process | negative regulation of cellular component movement | 3 |
| biological process | postreplication repair | 3 |
| biological process | ciliary basal body organization | 3 |
| biological process | protein heterooligomerization | 3 |
| biological process | muscle filament sliding | 3 |
| biological process | positive regulation of growth rate | 3 |
| biological process | adaptive immune response | 3 |
| biological process | cardiac septum development | 3 |
| biological process | positive regulation of DNA-dependent DNA replication initiation | 3 |
| biological process | regulation of transposon integration | 3 |
| biological process | transcriptional activation by promoter-terminator looping | 3 |
| biological process | response to manganese ion | 3 |
| biological process | regulation of protein homooligomerization | 3 |
| biological process | p-cresol catabolic process | 3 |
| biological process | intracellular mRNA localization | 3 |
| biological process | negative regulation by host of viral transcription | 3 |
| biological process | L-arabinose catabolic process | 3 |
| biological process | positive regulation of phosphorelay signal transduction system involved in hydrogen peroxide mediated signaling pathway | 3 |
| biological process | glomerular visceral epithelial cell development | 3 |
| biological process | tetrapyrrole metabolic process | 3 |
| biological process | deoxyribonucleoside catabolic process | 3 |
| biological process | retina homeostasis | 3 |
| biological process | mitotic spindle assembly | 3 |
| biological process | negative regulation of protein binding | 3 |
| biological process | cytosine metabolic process | 3 |
| biological process | ventricular cardiac muscle cell development | 3 |
| biological process | lateral ventricle development | 3 |
| biological process | aromatic compound biosynthetic process | 3 |
| biological process | positive regulation of NF-kappaB transcription factor activity | 3 |
| biological process | negative regulation of actin filament depolymerization | 3 |
| biological process | response to hyperoxia | 3 |
| biological process | melanocyte apoptotic process | 3 |
| biological process | establishment or maintenance of cell polarity regulating cell shape | 3 |
| biological process | regulation of polysaccharide biosynthetic process | 3 |
| biological process | sulfur oxidation | 3 |
| biological process | sulfur incorporation into metallo-sulfur cluster | 3 |
| biological process | pilus retraction | 3 |
| biological process | transition metal ion transport | 3 |
| biological process | sterol biosynthetic process | 3 |
| biological process | response to auxin | 3 |
| biological process | positive regulation of cell migration | 3 |
| biological process | modulation of synaptic transmission | 3 |
| biological process | D-xylose metabolic process | 3 |
| biological process | cytochrome c-heme linkage via heme-L-cysteine | 3 |
| biological process | cellular response to selenium ion | 3 |
| biological process | protein localization to nuclear envelope | 3 |
| biological process | detection of oxidative stress | 3 |
| biological process | transfer RNA gene-mediated silencing | 3 |
| biological process | positive regulation of execution phase of apoptosis | 3 |
| biological process | acetylcholine biosynthetic process | 3 |
| biological process | developmental growth | 3 |
| biological process | Type IV pili-dependent localized adherence to host | 3 |
| biological process | peptidyl-serine autophosphorylation | 3 |
| biological process | root development | 3 |
| biological process | hypoxanthine transport | 3 |
| biological process | carbamoyl phosphate catabolic process | 3 |
| biological process | PSII associated light-harvesting complex II catabolic process | 3 |
| biological process | gamma-aminobutyric acid biosynthetic process | 3 |
| biological process | larval somatic muscle development | 3 |
| biological process | positive regulation of leucine biosynthetic process | 3 |
| biological process | endothelial cell migration | 3 |
| biological process | guanosine catabolic process | 3 |
| biological process | lipid localization | 3 |
| biological process | negative regulation of molecular function | 3 |
| biological process | peptidyl-lysine desuccinylation | 3 |
| biological process | protein uridylylation | 3 |
| biological process | L-lysine transport | 3 |
| biological process | mitotic G1 DNA damage checkpoint | 3 |
| biological process | regulation of hemocyte proliferation | 3 |
| biological process | mitochondrial tRNA thio-modification | 3 |
| biological process | L-fucose metabolic process | 3 |
| biological process | metaphase plate congression | 3 |
| biological process | catabolism by organism of cell wall peptidoglycan in other organism | 3 |
| biological process | vacuole inheritance | 3 |
| biological process | positive regulation of histone H3-K4 methylation | 3 |
| biological process | protein maturation by protein folding | 3 |
| biological process | regulation of nitrogen compound metabolic process | 3 |
| biological process | cell projection assembly | 3 |
| biological process | pyrimidine-containing compound transmembrane transport | 3 |
| biological process | negative regulation of extracellular matrix disassembly | 3 |
| biological process | skeletal system morphogenesis | 3 |
| biological process | retrograde protein transport, ER to cytosol | 3 |
| biological process | protein import into mitochondrial intermembrane space | 3 |
| biological process | carbohydrate derivative biosynthetic process | 3 |
| biological process | diacetylchitobiose catabolic process | 3 |
| biological process | fin morphogenesis | 3 |
| biological process | cellular response to pH | 3 |
| biological process | ovarian follicle atresia | 3 |
| biological process | regulation of RNA metabolic process | 3 |
| biological process | melanocyte proliferation | 3 |
| biological process | aerobic phenol-containing compound catabolic process | 3 |
| biological process | positive regulation of defense response to virus by host | 3 |
| biological process | pseudohyphal growth | 3 |
| biological process | positive regulation of growth of symbiont in host | 3 |
| biological process | substrate-dependent cell migration, cell extension | 3 |
| biological process | indoleacetic acid biosynthetic process | 3 |
| biological process | telomere tethering at nuclear periphery | 3 |
| biological process | negative regulation of mRNA splicing, via spliceosome | 3 |
| biological process | negative regulation of positive chemotaxis | 3 |
| biological process | catabolic process | 3 |
| biological process | embryonic skeletal system development | 3 |
| biological process | homocysteine catabolic process | 3 |
| biological process | collagen-activated tyrosine kinase receptor signaling pathway | 3 |
| biological process | regulation of proteolysis | 3 |
| biological process | arabitol catabolic process | 3 |
| biological process | gene silencing by RNA | 3 |
| biological process | spermidine hydroxycinnamate conjugate biosynthetic process | 3 |
| biological process | NAD biosynthesis via nicotinamide riboside salvage pathway | 3 |
| biological process | histone H3-K4 methylation | 3 |
| biological process | sulfate assimilation, phosphoadenylyl sulfate reduction by phosphoadenylyl-sulfate reductase (thioredoxin) | 3 |
| biological process | response to organic cyclic compound | 3 |
| biological process | cyclic-nucleotide-mediated signaling | 3 |
| biological process | regulation of DNA-templated transcription in response to stress | 3 |
| biological process | neuron projection morphogenesis | 3 |
| biological process | positive regulation of cell size | 3 |
| biological process | dauer larval development | 3 |
| biological process | brassinosteroid homeostasis | 3 |
| biological process | mitochondrion inheritance | 3 |
| biological process | cell morphogenesis involved in neuron differentiation | 3 |
| biological process | aerobic glycerol catabolic process | 3 |
| biological process | negative regulation of single-species biofilm formation | 3 |
| biological process | photoinhibition | 3 |
| biological process | ornithine catabolic process | 3 |
| biological process | bone trabecula formation | 3 |
| biological process | negative regulation of inflammatory response | 3 |
| biological process | fucose transport | 3 |
| biological process | plus-end-directed vesicle transport along microtubule | 3 |
| biological process | positive regulation of synaptic transmission | 3 |
| biological process | L-galactonate catabolic process | 3 |
| biological process | uracil salvage | 3 |
| biological process | glucuronoside catabolic process | 3 |
| biological process | G1/S transition of mitotic cell cycle | 3 |
| biological process | gastrulation | 3 |
| biological process | stress-induced mitochondrial fusion | 2 |
| biological process | melanosome organization | 2 |
| biological process | phosphatidylinositol biosynthetic process | 2 |
| biological process | regulation of heart growth | 2 |
| biological process | leukocyte cell-cell adhesion | 2 |
| biological process | nerve growth factor signaling pathway | 2 |
| biological process | cellular response to cell-matrix adhesion | 2 |
| biological process | glycine transport | 2 |
| biological process | detoxification of nitrogen compound | 2 |
| biological process | membrane assembly | 2 |
| biological process | inorganic diphosphate transport | 2 |
| biological process | dorsal closure, leading edge cell differentiation | 2 |
| biological process | antigen processing and presentation of exogenous peptide antigen via MHC class II | 2 |
| biological process | ferrous iron import | 2 |
| biological process | dorsal closure, spreading of leading edge cells | 2 |
| biological process | cold acclimation | 2 |
| biological process | establishment of protein localization | 2 |
| biological process | negative regulation of protein sumoylation | 2 |
| biological process | ubiquinone biosynthetic process from chorismate | 2 |
| biological process | response to intra-S DNA damage checkpoint signaling | 2 |
| biological process | short-chain fatty acid transport | 2 |
| biological process | mannosamine biosynthetic process | 2 |
| biological process | negative regulation of interleukin-2 production | 2 |
| biological process | meiotic DNA double-strand break formation | 2 |
| biological process | alkanesulfonate catabolic process | 2 |
| biological process | response to cobalt ion | 2 |
| biological process | vesicle docking | 2 |
| biological process | mycotoxin biosynthetic process | 2 |
| biological process | pyruvate biosynthetic process | 2 |
| biological process | response to endoplasmic reticulum stress | 2 |
| biological process | positive regulation of secondary metabolite biosynthetic process | 2 |
| biological process | response to redox state | 2 |
| biological process | galactonate catabolic process | 2 |
| biological process | drought recovery | 2 |
| biological process | response to fungus | 2 |
| biological process | plant-type cell wall organization | 2 |
| biological process | histone monoubiquitination | 2 |
| biological process | glycerolipid catabolic process | 2 |
| biological process | positive regulation of sulfur utilization | 2 |
| biological process | flight behavior | 2 |
| biological process | protein targeting to peroxisome | 2 |
| biological process | determination of left/right symmetry | 2 |
| biological process | energy reserve metabolic process | 2 |
| biological process | nicotinamide riboside transport | 2 |
| biological process | circadian regulation of gene expression | 2 |
| biological process | regulation of centriole-centriole cohesion | 2 |
| biological process | positive regulation of histone acetylation | 2 |
| biological process | hemicellulose catabolic process | 2 |
| biological process | chromatin assembly | 2 |
| biological process | kynurenic acid biosynthetic process | 2 |
| biological process | purine ribonucleotide catabolic process | 2 |
| biological process | phosphatidylglycerol metabolic process | 2 |
| biological process | renal system development | 2 |
| biological process | mitochondrial calcium ion transport | 2 |
| biological process | histone H3 deacetylation | 2 |
| biological process | late endosome to vacuole transport | 2 |
| biological process | regulation of syncytium formation by plasma membrane fusion | 2 |
| biological process | growth plate cartilage chondrocyte development | 2 |
| biological process | flavonoid biosynthetic process | 2 |
| biological process | opsonization | 2 |
| biological process | ketone body biosynthetic process | 2 |
| biological process | cellular modified amino acid catabolic process | 2 |
| biological process | vocalization behavior | 2 |
| biological process | membrane lipid metabolic process | 2 |
| biological process | regulation of nucleus size | 2 |
| biological process | regulation of protein serine/threonine phosphatase activity | 2 |
| biological process | hypoxanthine metabolic process | 2 |
| biological process | positive regulation of mitochondrial DNA replication | 2 |
| biological process | L-methionine biosynthetic process from homoserine via O-succinyl-L-homoserine and cystathionine | 2 |
| biological process | salivary gland morphogenesis | 2 |
| biological process | neuron apoptotic process | 2 |
| biological process | negative regulation of DNA damage response, signal transduction by p53 class mediator | 2 |
| biological process | cellular senescence | 2 |
| biological process | positive regulation of methylation-dependent chromatin silencing | 2 |
| biological process | protein deglutathionylation | 2 |
| biological process | lactose metabolic process | 2 |
| biological process | protein targeting to vacuole | 2 |
| biological process | S-adenosylhomocysteine metabolic process | 2 |
| biological process | polyketide biosynthetic process | 2 |
| biological process | response to nutrient | 2 |
| biological process | positive regulation of superoxide dismutase activity | 2 |
| biological process | regulation of cell cycle process | 2 |
| biological process | oxidative photosynthetic carbon pathway | 2 |
| biological process | methionine transport | 2 |
| biological process | butyrate metabolic process | 2 |
| biological process | nuclear retention of pre-mRNA with aberrant 3'-ends at the site of transcription | 2 |
| biological process | protein K63-linked ubiquitination | 2 |
| biological process | L-fucose biosynthetic process | 2 |
| biological process | intracellular transport | 2 |
| biological process | modulation of apoptotic process in other organism | 2 |
| biological process | diadenosine tetraphosphate catabolic process | 2 |
| biological process | hemopoiesis | 2 |
| biological process | microtubule-based process | 2 |
| biological process | establishment of meiotic spindle localization | 2 |
| biological process | kinetochore organization | 2 |
| biological process | endoplasmic reticulum unfolded protein response | 2 |
| biological process | modulation by symbiont of host immune response | 2 |
| biological process | centromere clustering | 2 |
| biological process | stress granule assembly | 2 |
| biological process | mitochondrial fusion | 2 |
| biological process | open tracheal system development | 2 |
| biological process | regulation of intracellular pH | 2 |
| biological process | proteasome localization | 2 |
| biological process | mitotic anaphase | 2 |
| biological process | sporocarp development involved in sexual reproduction | 2 |
| biological process | left/right axis specification | 2 |
| biological process | fibril organization | 2 |
| biological process | mitotic spindle organization in nucleus | 2 |
| biological process | regulation of muscle contraction | 2 |
| biological process | rolling circle single-stranded viral DNA replication | 2 |
| biological process | dibenzo-p-dioxin metabolic process | 2 |
| biological process | RNA polymerase II transcriptional preinitiation complex assembly | 2 |
| biological process | phytosphingosine metabolic process | 2 |
| biological process | histone glutamine methylation | 2 |
| biological process | positive regulation of interleukin-8 secretion | 2 |
| biological process | transcription elongation from bacterial-type RNA polymerase promoter | 2 |
| biological process | phenotypic switching | 2 |
| biological process | positive regulation of ERK1 and ERK2 cascade | 2 |
| biological process | protein import into chloroplast stroma | 2 |
| biological process | hyperosmotic salinity response | 2 |
| biological process | response to gamma radiation | 2 |
| biological process | viral latency | 2 |
| biological process | invasive growth in response to glucose limitation | 2 |
| biological process | cellular response to lead ion | 2 |
| biological process | S-methylmethionine transport | 2 |
| biological process | positive regulation of RNA polymerase II transcriptional preinitiation complex assembly | 2 |
| biological process | peptidyl-arginine hydroxylation | 2 |
| biological process | L-xylitol catabolic process | 2 |
| biological process | cellular response to mechanical stimulus | 2 |
| biological process | single-species surface biofilm formation | 2 |
| biological process | imaginal disc-derived wing hair organization | 2 |
| biological process | phenylethylamine catabolic process | 2 |
| biological process | muscle cell differentiation | 2 |
| biological process | peptide biosynthetic process | 2 |
| biological process | glycolate biosynthetic process | 2 |
| biological process | cytoplasmic translational initiation | 2 |
| biological process | cellular response to light stimulus | 2 |
| biological process | regulation of myoblast fusion | 2 |
| biological process | carbazole catabolic process | 2 |
| biological process | intra-S DNA damage checkpoint | 2 |
| biological process | purine nucleoside interconversion | 2 |
| biological process | regulation of liquid surface tension | 2 |
| biological process | coenzyme A catabolic process | 2 |
| biological process | negative regulation of reciprocal meiotic recombination | 2 |
| biological process | microtubule anchoring at centrosome | 2 |
| biological process | fructose biosynthetic process | 2 |
| biological process | random inactivation of X chromosome | 2 |
| biological process | negative regulation of chromatin binding | 2 |
| biological process | negative regulation of T cell proliferation | 2 |
| biological process | actin cortical patch localization | 2 |
| biological process | protein monoubiquitination | 2 |
| biological process | anterior midgut development | 2 |
| biological process | protein-N6-(L-lysyl)-L-lysine modification to protein-N6-(beta-lysyl)-L-lysine | 2 |
| biological process | phagocytosis | 2 |
| biological process | negative regulation of chromatin silencing at rDNA | 2 |
| biological process | phytol metabolic process | 2 |
| biological process | fungal-type cell wall biogenesis | 2 |
| biological process | positive regulation of sequence-specific DNA binding transcription factor activity | 2 |
| biological process | negative regulation of NF-kappaB transcription factor activity | 2 |
| biological process | regulation of striated muscle contraction | 2 |
| biological process | mitotic actomyosin contractile ring contraction | 2 |
| biological process | dormancy exit of symbiont in host | 2 |
| biological process | coflocculation | 2 |
| biological process | myosin II filament assembly | 2 |
| biological process | positive regulation of cellular carbohydrate metabolic process | 2 |
| biological process | conidiophore development | 2 |
| biological process | multicellular organismal response to stress | 2 |
| biological process | cellular protein complex assembly | 2 |
| biological process | lipoprotein metabolic process | 2 |
| biological process | mannosylation | 2 |
| biological process | beta-alanine transport | 2 |
| biological process | DNA methylation on cytosine within a CHH sequence | 2 |
| biological process | detoxification of cadmium ion | 2 |
| biological process | regulation of D-xylose catabolic process | 2 |
| biological process | zygote asymmetric cytokinesis in embryo sac | 2 |
| biological process | surfactant homeostasis | 2 |
| biological process | muscle fiber development | 2 |
| biological process | AMP catabolic process | 2 |
| biological process | positive regulation of proline catabolic process to glutamate | 2 |
| biological process | nucleoid organization | 2 |
| biological process | regulation of stomatal closure | 2 |
| biological process | poly-hydroxybutyrate biosynthetic process | 2 |
| biological process | red or far-red light signaling pathway | 2 |
| biological process | motor neuron axon guidance | 2 |
| biological process | neuronal action potential | 2 |
| biological process | DNA demethylation | 2 |
| biological process | negative regulation of chromatin silencing at telomere | 2 |
| biological process | hemidesmosome assembly | 2 |
| biological process | glial cell apoptotic process | 2 |
| biological process | Malpighian tubule morphogenesis | 2 |
| biological process | dorsal closure, amnioserosa morphology change | 2 |
| biological process | regulation of heart rate | 2 |
| biological process | integrin activation | 2 |
| biological process | negative regulation of histone H3-K9 methylation | 2 |
| biological process | palmitic acid biosynthetic process | 2 |
| biological process | lateral root development | 2 |
| biological process | cell adhesion mediated by integrin | 2 |
| biological process | regulation of endoplasmic reticulum unfolded protein response | 2 |
| biological process | interleukin-2 production | 2 |
| biological process | B cell differentiation | 2 |
| biological process | chlamydospore formation | 2 |
| biological process | cuticle pattern formation | 2 |
| biological process | lung alveolus development | 2 |
| biological process | homeostasis of number of cells within a tissue | 2 |
| biological process | tRNA N1-guanine methylation | 2 |
| biological process | clathrin-mediated endocytosis | 2 |
| biological process | positive gravitropism | 2 |
| biological process | negative regulation of cysteine-type endopeptidase activity involved in apoptotic process | 2 |
| biological process | negative regulation of antisense RNA transcription | 2 |
| biological process | mitochondrion morphogenesis | 2 |
| biological process | spinal cord development | 2 |
| biological process | rubidium ion transport | 2 |
| biological process | miRNA metabolic process | 2 |
| biological process | actin nucleation | 2 |
| biological process | spermidine metabolic process | 2 |
| biological process | response to defense-related host nitric oxide production | 2 |
| biological process | oligosaccharide-lipid intermediate biosynthetic process | 2 |
| biological process | tRNA 3'-trailer cleavage, endonucleolytic | 2 |
| biological process | regulation of post-mating oviposition | 2 |
| biological process | flocculation | 2 |
| biological process | macromolecule deacylation | 2 |
| biological process | plasmodesmata-mediated intercellular transport | 2 |
| biological process | maintenance of protein location in cell | 2 |
| biological process | cellular response to organic cyclic compound | 2 |
| biological process | membrane depolarization during action potential | 2 |
| biological process | kinetochore assembly | 2 |
| biological process | T cell receptor signaling pathway | 2 |
| biological process | morphogenesis of an epithelium | 2 |
| biological process | error-prone translesion synthesis | 2 |
| biological process | filamentous growth of a population of unicellular organisms in response to chemical stimulus | 2 |
| biological process | positive regulation of fatty acid biosynthetic process | 2 |
| biological process | protein-lysine lysylation | 2 |
| biological process | negative regulation of insulin secretion | 2 |
| biological process | striated muscle tissue development | 2 |
| biological process | polyamine metabolic process | 2 |
| biological process | regulation of proteasomal protein catabolic process | 2 |
| biological process | cytoplasmic sequestering of transcription factor | 2 |
| biological process | negative regulation of complement activation | 2 |
| biological process | positive regulation of cytokinesis | 2 |
| biological process | oligodendrocyte apoptotic process | 2 |
| biological process | ovarian follicle cell migration | 2 |
| biological process | karyogamy | 2 |
| biological process | regulation of dosage compensation by inactivation of X chromosome | 2 |
| biological process | aerobactin transport | 2 |
| biological process | histone H2B ubiquitination | 2 |
| biological process | aminophosphonate metabolic process | 2 |
| biological process | anthranilate metabolic process | 2 |
| biological process | cytokine-mediated signaling pathway | 2 |
| biological process | leading strand elongation | 2 |
| biological process | protein localization to nucleolar rDNA repeats | 2 |
| biological process | regulation of tube size, open tracheal system | 2 |
| biological process | regulation of transcription involved in G1/S transition of mitotic cell cycle | 2 |
| biological process | spiracle morphogenesis, open tracheal system | 2 |
| biological process | developmental pigmentation | 2 |
| biological process | seed germination | 2 |
| biological process | isopeptide cross-linking | 2 |
| biological process | chitin-based cuticle development | 2 |
| biological process | amylopectin catabolic process | 2 |
| biological process | head involution | 2 |
| biological process | RNA 3'-end processing | 2 |
| biological process | shikimate biosynthetic process | 2 |
| biological process | alternative mRNA splicing, via spliceosome | 2 |
| biological process | regulation of embryonic development | 2 |
| biological process | nucleobase-containing compound transport | 2 |
| biological process | response to morphine | 2 |
| biological process | glycosphingolipid metabolic process | 2 |
| biological process | learning or memory | 2 |
| biological process | actin filament bundle distribution | 2 |
| biological process | proteasomal ubiquitin-independent protein catabolic process | 2 |
| biological process | S-adenosylmethioninamine metabolic process | 2 |
| biological process | cellular response to insulin stimulus | 2 |
| biological process | macromolecule biosynthetic process | 2 |
| biological process | positive regulation of peptidase activity | 2 |
| biological process | RNA export from nucleus | 2 |
| biological process | lysosomal transport | 2 |
| biological process | carbon catabolite regulation of transcription | 2 |
| biological process | histone H4 deacetylation | 2 |
| biological process | lipid particle organization | 2 |
| biological process | GTP metabolic process | 2 |
| biological process | mitochondrial electron transport, ubiquinol to cytochrome c | 2 |
| biological process | S-adenosylmethionine metabolic process | 2 |
| biological process | zymogen activation | 2 |
| biological process | positive regulation of macroautophagy | 2 |
| biological process | lipid phosphorylation | 2 |
| biological process | positive regulation of programmed cell death | 2 |
| biological process | ribonucleotide metabolic process | 2 |
| biological process | histone H3-K9 methylation | 2 |
| biological process | establishment of centrosome localization | 2 |
| biological process | dorsal trunk growth, open tracheal system | 2 |
| biological process | positive regulation of mitotic metaphase/anaphase transition | 2 |
| biological process | stabilization of membrane potential | 2 |
| biological process | glucarate catabolic process | 2 |
| biological process | negative regulation of double-strand break repair via homologous recombination | 2 |
| biological process | unidimensional cell growth | 2 |
| biological process | GDP-L-fucose salvage | 2 |
| biological process | positive regulation of isotype switching | 2 |
| biological process | apoptotic mitochondrial changes | 2 |
| biological process | double fertilization forming a zygote and endosperm | 2 |
| biological process | negative regulation of transcription from RNA polymerase II promoter in response to iron | 2 |
| biological process | CAAX-box protein processing | 2 |
| biological process | replication fork protection | 2 |
| biological process | SRP-dependent cotranslational protein targeting to membrane, signal sequence recognition | 2 |
| biological process | cell aging | 2 |
| biological process | morphogenesis of a branching structure | 2 |
| biological process | positive regulation of intrinsic apoptotic signaling pathway in response to DNA damage | 2 |
| biological process | tRNA 3'-trailer cleavage | 2 |
| biological process | B cell receptor signaling pathway | 2 |
| biological process | visceral muscle development | 2 |
| biological process | DNA damage checkpoint | 2 |
| biological process | nitric oxide metabolic process | 2 |
| biological process | intrinsic apoptotic signaling pathway by p53 class mediator | 2 |
| biological process | vesicle-mediated transport | 2 |
| biological process | negative regulation of neuron projection development | 2 |
| biological process | macroautophagy | 2 |
| biological process | mRNA splice site selection | 2 |
| biological process | box C/D snoRNA 3'-end processing | 2 |
| biological process | chaeta morphogenesis | 2 |
| biological process | smooth muscle contraction | 2 |
| biological process | somatic hypermutation of immunoglobulin genes | 2 |
| biological process | regulation of RNA polymerase II transcriptional preinitiation complex assembly | 2 |
| biological process | phosphatidylinositol metabolic process | 2 |
| biological process | chronological cell aging | 2 |
| biological process | chemical synaptic transmission | 2 |
| biological process | fibrinolysis | 2 |
| biological process | sorocarp stalk development | 2 |
| biological process | xenobiotic catabolic process | 2 |
| biological process | cellular response to retinoic acid | 2 |
| biological process | positive regulation of transcription elongation from RNA polymerase II promoter | 2 |
| biological process | regulation of transcription involved in G2/M transition of mitotic cell cycle | 2 |
| biological process | histone H2A-H2B dimer displacement | 2 |
| biological process | cell surface receptor signaling pathway | 2 |
| biological process | CMP-keto-3-deoxy-D-manno-octulosonic acid biosynthetic process | 2 |
| biological process | regulation of multicellular organism growth | 2 |
| biological process | nitroglycerin metabolic process | 2 |
| biological process | antigen processing and presentation of endogenous peptide antigen via MHC class I via ER pathway, TAP-dependent | 2 |
| biological process | histone modification | 2 |
| biological process | dGDP phosphorylation | 2 |
| biological process | positive regulation of endothelial cell migration | 2 |
| biological process | chlorophyll catabolic process | 2 |
| biological process | transcription initiation from RNA polymerase II promoter | 2 |
| biological process | epithelium migration | 2 |
| biological process | budding cell apical bud growth | 2 |
| biological process | single-species submerged biofilm formation | 2 |
| biological process | carbohydrate derivative metabolic process | 2 |
| biological process | 2'-(5''-triphosphoribosyl)-3'-dephospho-CoA biosynthetic process | 2 |
| biological process | respiratory gaseous exchange | 2 |
| biological process | response to peptide hormone | 2 |
| biological process | negative regulation of phosphorelay signal transduction system | 2 |
| biological process | thyroid hormone transport | 2 |
| biological process | DNA hypermethylation | 2 |
| biological process | spore wall assembly | 2 |
| biological process | axonogenesis involved in innervation | 2 |
| biological process | septate junction assembly | 2 |
| biological process | spore wall biogenesis | 2 |
| biological process | meiotic gene conversion | 2 |
| biological process | midbrain-hindbrain boundary morphogenesis | 2 |
| biological process | response to hormone | 2 |
| biological process | epithelial cell-cell adhesion | 2 |
| biological process | poly(3-hydroxyalkanoate) biosynthetic process | 2 |
| biological process | regulation of phenotypic switching by regulation of transcription from RNA polymerase II promoter | 2 |
| biological process | positive regulation of release of cytochrome c from mitochondria | 2 |
| biological process | mature ribosome assembly | 2 |
| biological process | arginine catabolic process to ornithine | 2 |
| biological process | response to karrikin | 2 |
| biological process | carotene biosynthetic process | 2 |
| biological process | nuclear-transcribed mRNA catabolic process, nonsense-mediated decay | 2 |
| biological process | regulation of transcription from RNA polymerase II promoter in response to stress | 2 |
| biological process | L-methionine salvage | 2 |
| biological process | meiotic chromosome segregation | 2 |
| biological process | B cell proliferation | 2 |
| biological process | leaf senescence | 2 |
| biological process | energy coupled proton transmembrane transport, against electrochemical gradient | 2 |
| biological process | cyclic nucleotide biosynthetic process | 2 |
| biological process | positive regulation of angiogenesis | 2 |
| biological process | Notch signaling pathway | 2 |
| biological process | regulation of cellular response to heat | 2 |
| biological process | N-terminal peptidyl-serine acetylation | 2 |
| biological process | aldonate transport | 2 |
| biological process | mRNA export from nucleus in response to heat stress | 2 |
| biological process | acetyl-CoA catabolic process | 2 |
| biological process | Group II intron splicing | 2 |
| biological process | negative regulation of thioredoxin peroxidase activity | 2 |
| biological process | somatic recombination of immunoglobulin gene segments | 2 |
| biological process | establishment of neuroblast polarity | 2 |
| biological process | antigen processing and presentation | 2 |
| biological process | cholesterol catabolic process | 2 |
| biological process | jasmonic acid metabolic process | 2 |
| biological process | cellular response to gamma radiation | 2 |
| biological process | cellular response to ethylene stimulus | 2 |
| biological process | tetrahydrofolate metabolic process | 2 |
| biological process | reverse transcription involved in RNA-mediated transposition | 2 |
| biological process | ascospore-type prospore-specific spindle pole body remodeling | 2 |
| biological process | positive regulation of enamel mineralization | 2 |
| biological process | isoleucine transmembrane transport | 2 |
| biological process | propionate biosynthetic process | 2 |
| biological process | D-alanine transport | 2 |
| biological process | BMP signaling pathway | 2 |
| biological process | mitotic prometaphase | 2 |
| biological process | positive regulation of neuron projection development | 2 |
| biological process | maintenance of lens transparency | 2 |
| biological process | guanine salvage | 2 |
| biological process | DNA protection | 2 |
| biological process | carotene metabolic process | 2 |
| biological process | regulation of chemotaxis | 2 |
| biological process | nucleoside catabolic process | 2 |
| biological process | hermaphrodite genitalia development | 2 |
| biological process | mannose biosynthetic process | 2 |
| biological process | antigenic variation | 2 |
| biological process | positive regulation of mitochondrial membrane potential | 2 |
| biological process | phospholipid dephosphorylation | 2 |
| biological process | positive regulation of nitrogen utilization | 2 |
| biological process | response to inorganic substance | 2 |
| biological process | response to lipopolysaccharide | 2 |
| biological process | negative regulation of transcription from RNA polymerase II promoter by pheromones | 2 |
| biological process | beta-alanine metabolic process | 2 |
| biological process | RNA splicing | 2 |
| biological process | protein insertion into membrane from inner side | 2 |
| biological process | 4-nitrophenol catabolic process | 2 |
| biological process | CD4-positive, alpha-beta T cell activation | 2 |
| biological process | positive regulation of cardiolipin metabolic process | 2 |
| biological process | response to growth factor | 2 |
| biological process | response to herbicide | 2 |
| biological process | negative regulation of flower development | 2 |
| biological process | cellular bud site selection | 2 |
| biological process | regulation of calcium-mediated signaling | 2 |
| biological process | non-phosphorylated glucose catabolic process | 2 |
| biological process | regulation of mRNA stability involved in response to oxidative stress | 2 |
| biological process | response to calcium ion | 2 |
| biological process | histone methylation | 2 |
| biological process | negative regulation of Ras protein signal transduction | 2 |
| biological process | monovalent inorganic cation transport | 2 |
| biological process | visual learning | 2 |
| biological process | centrosome duplication | 2 |
| biological process | response to magnesium ion | 2 |
| biological process | positive regulation of microtubule depolymerization | 2 |
| biological process | root hair cell tip growth | 2 |
| biological process | posttranscriptional gene silencing | 2 |
| biological process | response to glucocorticoid | 2 |
| biological process | negative regulation of transcription involved in meiotic cell cycle | 2 |
| biological process | positive regulation of isoprenoid metabolic process | 2 |
| biological process | viral genome packaging | 2 |
| biological process | positive regulation of fibroblast proliferation | 2 |
| biological process | regulation of protein import into nucleus | 2 |
| biological process | DNA replication-independent nucleosome assembly | 2 |
| biological process | amyloid fibril formation | 2 |
| biological process | bacterial-type DNA replication initiation | 2 |
| biological process | chromatin silencing at centromere | 2 |
| biological process | peptidyl-threonine phosphorylation | 2 |
| biological process | response to corticosteroid | 2 |
| biological process | protein localization to Golgi apparatus | 2 |
| biological process | inner ear receptor stereocilium organization | 2 |
| biological process | primary miRNA processing | 2 |
| biological process | glycerol biosynthetic process | 2 |
| biological process | DNA methylation involved in embryo development | 2 |
| biological process | response to alkane | 2 |
| biological process | chromatin assembly or disassembly | 2 |
| biological process | odontogenesis | 2 |
| biological process | FAD transmembrane transport | 2 |
| biological process | negative regulation of ATPase activity | 2 |
| biological process | regulation of immune response | 2 |
| biological process | cristae formation | 2 |
| biological process | activation of JUN kinase activity | 2 |
| biological process | phenylpropanoid metabolic process | 2 |
| biological process | gliotoxin biosynthetic process | 2 |
| biological process | mycothiol metabolic process | 2 |
| biological process | succinyl-CoA catabolic process | 2 |
| biological process | cinnamic acid catabolic process | 2 |
| biological process | pyochelin biosynthetic process | 2 |
| biological process | osteoclast differentiation | 2 |
| biological process | water homeostasis | 2 |
| biological process | hyaluronan metabolic process | 2 |
| biological process | larval locomotory behavior | 2 |
| biological process | cellular polysaccharide metabolic process | 2 |
| biological process | response to selenium ion | 2 |
| biological process | dTTP metabolic process | 2 |
| biological process | negative regulation of chromatin silencing at silent mating-type cassette | 2 |
| biological process | negative regulation of transcription from RNA polymerase I promoter | 2 |
| biological process | pseudopodium organization | 2 |
| biological process | centrosome localization | 2 |
| biological process | gonad development | 2 |
| biological process | Wnt signaling pathway, planar cell polarity pathway | 2 |
| biological process | cellular glucose homeostasis | 2 |
| biological process | head development | 1 |
| biological process | uridine catabolic process | 1 |
| biological process | intrahepatic bile duct development | 1 |
| biological process | cellular modified amino acid biosynthetic process | 1 |
| biological process | ubiquinone metabolic process | 1 |
| biological process | male genitalia development | 1 |
| biological process | ubiquitin-dependent SMAD protein catabolic process | 1 |
| biological process | positive regulation of cellular response to drug | 1 |
| biological process | alginic acid catabolic process | 1 |
| biological process | citrate metabolic process | 1 |
| biological process | dolichol biosynthetic process | 1 |
| biological process | mitotic cell cycle checkpoint | 1 |
| biological process | cellular response to fibroblast growth factor stimulus | 1 |
| biological process | cellular response to chemical stimulus | 1 |
| biological process | regulation of vascular endothelial growth factor signaling pathway | 1 |
| biological process | regulation of Rho protein signal transduction | 1 |
| biological process | translational frameshifting | 1 |
| biological process | exocrine pancreas development | 1 |
| biological process | positive regulation of cell adhesion mediated by integrin | 1 |
| biological process | calcium ion export from cell | 1 |
| biological process | regulation of NAD+ kinase activity | 1 |
| biological process | nuclear import | 1 |
| biological process | pollen exine formation | 1 |
| biological process | regulation of hydrolase activity | 1 |
| biological process | acetate transmembrane transport | 1 |
| biological process | establishment of Golgi localization | 1 |
| biological process | regulation of mitotic recombination | 1 |
| biological process | lens development in camera-type eye | 1 |
| biological process | L-homocysteine biosynthetic process | 1 |
| biological process | triterpenoid biosynthetic process | 1 |
| biological process | developmental process involved in reproduction | 1 |
| biological process | negative regulation of transforming growth factor-beta secretion | 1 |
| biological process | pyruvate transport | 1 |
| biological process | regulation of glucocorticoid metabolic process | 1 |
| biological process | intracellular pH reduction | 1 |
| biological process | response to exogenous dsRNA | 1 |
| biological process | alpha-ketoglutarate transport | 1 |
| biological process | response to symbiotic bacterium | 1 |
| biological process | L-aspartate transport | 1 |
| biological process | negative regulation of gene expression, epigenetic | 1 |
| biological process | non-recombinational repair | 1 |
| biological process | methylamine metabolic process | 1 |
| biological process | cellular response to menadione | 1 |
| biological process | positive regulation by symbiont of defense-related host reactive oxygen species production | 1 |
| biological process | positive regulation of antigen processing and presentation of peptide antigen via MHC class I | 1 |
| biological process | positive thymic T cell selection | 1 |
| biological process | response to lithium ion | 1 |
| biological process | protein deacylation | 1 |
| biological process | intracellular sterol transport | 1 |
| biological process | diacylglycerol biosynthetic process | 1 |
| biological process | negative regulation of glucose mediated signaling pathway | 1 |
| biological process | limb morphogenesis | 1 |
| biological process | bicellular tight junction assembly | 1 |
| biological process | eye development | 1 |
| biological process | nodulation | 1 |
| biological process | regulation of transcription from RNA polymerase II promoter in response to DNA damage | 1 |
| biological process | negative regulation of canonical Wnt signaling pathway | 1 |
| biological process | insulin receptor signaling pathway | 1 |
| biological process | L-arabinose catabolic process to 2-oxoglutarate | 1 |
| biological process | positive regulation of organic acid transport | 1 |
| biological process | intracellular transport of virus | 1 |
| biological process | positive regulation of pseudohyphal growth by positive regulation of transcription from RNA polymerase II promoter | 1 |
| biological process | viral genome replication | 1 |
| biological process | nuclear pore complex assembly | 1 |
| biological process | MAPK import into nucleus | 1 |
| biological process | actin cortical patch internalization | 1 |
| biological process | glutathione transmembrane import into vacuole | 1 |
| biological process | diet induced thermogenesis | 1 |
| biological process | transitional endoplasmic reticulum polarization at cell division site | 1 |
| biological process | positive regulation of type 2 immune response | 1 |
| biological process | positive regulation of B cell proliferation | 1 |
| biological process | regulation of protein dephosphorylation | 1 |
| biological process | propionate catabolic process | 1 |
| biological process | blood vessel remodeling | 1 |
| biological process | cyanate metabolic process | 1 |
| biological process | chromatin organization | 1 |
| biological process | nuclear pore organization | 1 |
| biological process | positive regulation of protein export from nucleus | 1 |
| biological process | positive regulation of skeletal muscle tissue development | 1 |
| biological process | posttranscriptional gene silencing by RNA | 1 |
| biological process | regulation of hair follicle development | 1 |
| biological process | growth involved in symbiotic interaction | 1 |
| biological process | axo-dendritic transport | 1 |
| biological process | mitotic G2 DNA damage checkpoint | 1 |
| biological process | polar body extrusion after meiotic divisions | 1 |
| biological process | spindle assembly | 1 |
| biological process | stress fiber assembly | 1 |
| biological process | blood vessel morphogenesis | 1 |
| biological process | maturation of 5S rRNA | 1 |
| biological process | cytoskeletal matrix organization at active zone | 1 |
| biological process | substantia nigra development | 1 |
| biological process | chloroplast fission | 1 |
| biological process | regulation of ossification | 1 |
| biological process | positive regulation of smoothened signaling pathway | 1 |
| biological process | positive regulation by host of viral process | 1 |
| biological process | novobiocin biosynthetic process | 1 |
| biological process | positive regulation of vascular endothelial growth factor production | 1 |
| biological process | neuromuscular synaptic transmission | 1 |
| biological process | negative regulation of imaginal disc-derived wing size | 1 |
| biological process | mannosylglycerate biosynthetic process | 1 |
| biological process | reelin-mediated signaling pathway | 1 |
| biological process | facial nerve morphogenesis | 1 |
| biological process | inflammatory response to wounding | 1 |
| biological process | protein kinase B signaling | 1 |
| biological process | positive regulation of glucose import | 1 |
| biological process | p-aminobenzoyl-glutamate transport | 1 |
| biological process | positive regulation of defense response to bacterium | 1 |
| biological process | negative regulation of JNK cascade | 1 |
| biological process | negative regulation of epithelial cell migration | 1 |
| biological process | telomeric loop formation | 1 |
| biological process | systemic acquired resistance | 1 |
| biological process | pentacyclic triterpenoid biosynthetic process | 1 |
| biological process | positive regulation of focal adhesion assembly | 1 |
| biological process | N',N'',N'''-triacetylfusarinine C biosynthetic process | 1 |
| biological process | melanin biosynthetic process | 1 |
| biological process | response to cisplatin | 1 |
| biological process | transcription from bacterial-type RNA polymerase promoter | 1 |
| biological process | DNA excision | 1 |
| biological process | 5,6,7,8-tetrahydromethanopterin biosynthetic process | 1 |
| biological process | negative regulation of mitochondrial fusion | 1 |
| biological process | positive regulation of protein localization to plasma membrane | 1 |
| biological process | xylulose catabolic process | 1 |
| biological process | NAD catabolic process | 1 |
| biological process | regulation of lipid metabolic process | 1 |
| biological process | steroid catabolic process | 1 |
| biological process | xylulose metabolic process | 1 |
| biological process | negative regulation of interferon-gamma production | 1 |
| biological process | establishment of protein localization to chromatin | 1 |
| biological process | modulation by symbiont of host defense-related programmed cell death | 1 |
| biological process | cAMP catabolic process | 1 |
| biological process | oxidative demethylation | 1 |
| biological process | D-amino acid biosynthetic process | 1 |
| biological process | malate transmembrane transport | 1 |
| biological process | mesoderm formation | 1 |
| biological process | intraciliary retrograde transport | 1 |
| biological process | protein localization to adherens junction | 1 |
| biological process | CDP-diacylglycerol catabolic process | 1 |
| biological process | transitive RNA interference | 1 |
| biological process | protein localization to synapse | 1 |
| biological process | heterotypic cell-cell adhesion | 1 |
| biological process | establishment of anatomical structure orientation | 1 |
| biological process | semaphorin-plexin signaling pathway involved in axon guidance | 1 |
| biological process | RNA destabilization | 1 |
| biological process | peripheral nervous system neuron development | 1 |
| biological process | deoxycytidine catabolic process | 1 |
| biological process | regulation of chromatin assembly | 1 |
| biological process | innate immune response in mucosa | 1 |
| biological process | pyrimidine deoxyribonucleoside salvage | 1 |
| biological process | positive regulation of dendritic cell antigen processing and presentation | 1 |
| biological process | regulation of receptor internalization | 1 |
| biological process | DNA-dependent DNA replication maintenance of fidelity | 1 |
| biological process | peptidyl-lysine monomethylation | 1 |
| biological process | negative regulation of exocytosis | 1 |
| biological process | fin regeneration | 1 |
| biological process | extracellular matrix disassembly | 1 |
| biological process | S-methylmethionine metabolic process | 1 |
| biological process | positive regulation of bacterial-type flagellum assembly | 1 |
| biological process | cytokinetic process | 1 |
| biological process | arginine homeostasis | 1 |
| biological process | immunoglobulin mediated immune response | 1 |
| biological process | negative regulation of androgen receptor signaling pathway | 1 |
| biological process | positive regulation of T cell differentiation | 1 |
| biological process | enzyme active site formation via cysteine modification to L-cysteine persulfide | 1 |
| biological process | carnitine transmembrane transport | 1 |
| biological process | gene silencing by miRNA | 1 |
| biological process | response to oxygen levels | 1 |
| biological process | positive regulation of chemotaxis | 1 |
| biological process | protein catabolic process in the vacuole | 1 |
| biological process | positive regulation of protein import into nucleus | 1 |
| biological process | deoxyribonucleoside triphosphate biosynthetic process | 1 |
| biological process | GDP catabolic process | 1 |
| biological process | chloroplast-nucleus signaling pathway | 1 |
| biological process | response to nitrate | 1 |
| biological process | hormone-mediated signaling pathway | 1 |
| biological process | calcium-independent cell-matrix adhesion | 1 |
| biological process | peptidyl-arginine methylation | 1 |
| biological process | chloroplast avoidance movement | 1 |
| biological process | sphingomyelin metabolic process | 1 |
| biological process | peptidyl-pyroglutamic acid biosynthetic process, using glutaminyl-peptide cyclotransferase | 1 |
| biological process | regulation of protein complex assembly | 1 |
| biological process | chloroplast accumulation movement | 1 |
| biological process | gene expression | 1 |
| biological process | mitotic actomyosin contractile ring maintenance | 1 |
| biological process | embryonic cleavage | 1 |
| biological process | ketogluconate catabolic process | 1 |
| biological process | response to symbiotic fungus | 1 |
| biological process | CMP biosynthetic process | 1 |
| biological process | regulation of phagocytosis | 1 |
| biological process | very-low-density lipoprotein particle clearance | 1 |
| biological process | negative regulation of insulin receptor signaling pathway | 1 |
| biological process | phloem transport | 1 |
| biological process | auxin-activated signaling pathway | 1 |
| biological process | energy homeostasis | 1 |
| biological process | regulation of ion transmembrane transporter activity | 1 |
| biological process | stem cell population maintenance | 1 |
| biological process | spindle midzone assembly | 1 |
| biological process | L-idonate transport | 1 |
| biological process | p-aminobenzoyl-glutamate transmembrane transport | 1 |
| biological process | deoxyribose phosphate catabolic process | 1 |
| biological process | negative regulation of carbohydrate metabolic process | 1 |
| biological process | red, far-red light phototransduction | 1 |
| biological process | anatomical structure morphogenesis | 1 |
| biological process | pentose metabolic process | 1 |
| biological process | cellular protein catabolic process | 1 |
| biological process | fumiquinazoline C biosynthetic process | 1 |
| biological process | protein acetylation | 1 |
| biological process | response to fructose | 1 |
| biological process | negative regulation of protein transport | 1 |
| biological process | nuclear polyadenylation-dependent mRNA catabolic process | 1 |
| biological process | positive aerotaxis | 1 |
| biological process | response to blue light | 1 |
| biological process | positive regulation of nuclear-transcribed mRNA catabolic process, deadenylation-dependent decay | 1 |
| biological process | cellular response to interleukin-6 | 1 |
| biological process | preassembly of GPI anchor in ER membrane | 1 |
| biological process | otic vesicle formation | 1 |
| biological process | biological regulation | 1 |
| biological process | endosome to lysosome transport | 1 |
| biological process | positive regulation of myelination | 1 |
| biological process | negative regulation of clathrin-mediated endocytosis | 1 |
| biological process | endodermal digestive tract morphogenesis | 1 |
| biological process | sphingomyelin catabolic process | 1 |
| biological process | early endosome to late endosome transport | 1 |
| biological process | intermediate filament polymerization or depolymerization | 1 |
| biological process | rhamnose transport | 1 |
| biological process | response to insect | 1 |
| biological process | regulation of autophagy | 1 |
| biological process | response to axon injury | 1 |
| biological process | regulation of response to drug | 1 |
| biological process | epithelial cell migration, open tracheal system | 1 |
| biological process | hemocyte proliferation | 1 |
| biological process | asymmetric protein localization involved in cell fate determination | 1 |
| biological process | homogentisate catabolic process | 1 |
| biological process | muscle thin filament assembly | 1 |
| biological process | cerebellum development | 1 |
| biological process | embryonic skeletal system morphogenesis | 1 |
| biological process | regulation of developmental growth | 1 |
| biological process | olfactory learning | 1 |
| biological process | dADP catabolic process | 1 |
| biological process | phytochelatin biosynthetic process | 1 |
| biological process | response to hermaphrodite contact | 1 |
| biological process | mixed acid fermentation | 1 |
| biological process | cellular response to testosterone stimulus | 1 |
| biological process | detection of redox state | 1 |
| biological process | ribosomal large subunit export from nucleus | 1 |
| biological process | root epidermal cell differentiation | 1 |
| biological process | negative regulation by symbiont of host cell cycle | 1 |
| biological process | heterochromatin assembly | 1 |
| biological process | atrazine catabolic process | 1 |
| biological process | sexual reproduction | 1 |
| biological process | regulation of bacterial-type flagellum-dependent cell motility by regulation of motor speed | 1 |
| biological process | response to vitamin A | 1 |
| biological process | localization within membrane | 1 |
| biological process | nickel cation homeostasis | 1 |
| biological process | tissue development | 1 |
| biological process | poly-hydroxybutyrate metabolic process | 1 |
| biological process | fruit development | 1 |
| biological process | hematopoietic stem cell proliferation | 1 |
| biological process | actomyosin contractile ring assembly | 1 |
| biological process | anterior/posterior pattern specification | 1 |
| biological process | methanol metabolic process | 1 |
| biological process | regulation of peptidyl-cysteine S-nitrosylation | 1 |
| biological process | regulation of stress fiber assembly | 1 |
| biological process | positive regulation of testosterone secretion | 1 |
| biological process | flight | 1 |
| biological process | anaerobic cobalamin biosynthetic process | 1 |
| biological process | oxidative single-stranded RNA demethylation | 1 |
| biological process | glutamate decarboxylation to succinate | 1 |
| biological process | C-terminal protein amino acid modification | 1 |
| biological process | D-cysteine catabolic process | 1 |
| biological process | production of siRNA involved in RNA interference | 1 |
| biological process | response to nematode | 1 |
| biological process | carotene catabolic process | 1 |
| biological process | axial cellular bud site selection | 1 |
| biological process | positive regulation of osteoblast proliferation | 1 |
| biological process | regulation of Wnt signaling pathway, planar cell polarity pathway | 1 |
| biological process | hemoglobin import | 1 |
| biological process | positive regulation of gastrulation | 1 |
| biological process | molting cycle, collagen and cuticulin-based cuticle | 1 |
| biological process | 9,9'-di-cis-zeta-carotene desaturation to 7,9,7',9'-tetra-cis-lycopene | 1 |
| biological process | positive regulation of reciprocal meiotic recombination | 1 |
| biological process | galactomannan metabolic process | 1 |
| biological process | oocyte maturation | 1 |
| biological process | generation of catalytic spliceosome for second transesterification step | 1 |
| biological process | behavior | 1 |
| biological process | musculoskeletal movement | 1 |
| biological process | insulin-like growth factor receptor signaling pathway | 1 |
| biological process | emericellamide biosynthetic process | 1 |
| biological process | lysine homeostasis | 1 |
| biological process | capsule organization | 1 |
| biological process | negative regulation of mature B cell apoptotic process | 1 |
| biological process | positive regulation of centrosome duplication | 1 |
| biological process | larval lymph gland hemopoiesis | 1 |
| biological process | mitotic spindle orientation checkpoint | 1 |
| biological process | spindle pole body separation | 1 |
| biological process | negative regulation of cAMP-dependent protein kinase activity | 1 |
| biological process | proteasome regulatory particle assembly | 1 |
| biological process | positive regulation of homophilic cell adhesion | 1 |
| biological process | peroxisome organization | 1 |
| biological process | muscle cell cellular homeostasis | 1 |
| biological process | positive regulation of actin cytoskeleton reorganization | 1 |
| biological process | vesicle transport along microtubule | 1 |
| biological process | negative regulation of chlorophyll biosynthetic process | 1 |
| biological process | regulation of vascular endothelial growth factor receptor signaling pathway | 1 |
| biological process | cellular ion homeostasis | 1 |
| biological process | nuclear polyadenylation-dependent rRNA catabolic process | 1 |
| biological process | mitotic DNA replication lagging strand elongation | 1 |
| biological process | meiotic spindle organization | 1 |
| biological process | L-serine transport | 1 |
| biological process | regulation of double-strand break repair via homologous recombination | 1 |
| biological process | Group I intron splicing | 1 |
| biological process | response to glucagon | 1 |
| biological process | L-kynurenine metabolic process | 1 |
| biological process | positive regulation of interferon-gamma secretion | 1 |
| biological process | positive regulation of chemokine (C-X-C motif) ligand 2 production | 1 |
| biological process | MAPK cascade | 1 |
| biological process | convergent extension | 1 |
| biological process | cellular response to water deprivation | 1 |
| biological process | regulation of histone H3-K14 acetylation | 1 |
| biological process | dodecyl sulfate metabolic process | 1 |
| biological process | positive regulation of autophagosome maturation | 1 |
| biological process | regulation of calcium ion transport | 1 |
| biological process | ethanolamine transport | 1 |
| biological process | positive regulation of type IV pilus biogenesis | 1 |
| biological process | positive regulation of innate immune response | 1 |
| biological process | regulation of developmental process | 1 |
| biological process | homophilic cell adhesion via plasma membrane adhesion molecules | 1 |
| biological process | positive regulation of DNA-templated transcription, termination | 1 |
| biological process | regulation of gluconeogenesis | 1 |
| biological process | nuclear matrix organization | 1 |
| biological process | activation of MAPK activity | 1 |
| biological process | adult somatic muscle development | 1 |
| biological process | regulation of cytoplasmic translation | 1 |
| biological process | hexose phosphate transport | 1 |
| biological process | negative regulation of lyase activity | 1 |
| biological process | negative regulation of nuclease activity | 1 |
| biological process | acrosome assembly | 1 |
| biological process | positive regulation of dendrite morphogenesis | 1 |
| biological process | bisulfite reduction | 1 |
| biological process | glycolytic fermentation to ethanol | 1 |
| biological process | regulation of plasma membrane sterol distribution | 1 |
| biological process | negative regulation of MAPK cascade | 1 |
| biological process | viral transcription | 1 |
| biological process | Golgi localization | 1 |
| biological process | aldaric acid metabolic process | 1 |
| biological process | chromatin organization involved in regulation of transcription | 1 |
| biological process | cellular cadmium ion homeostasis | 1 |
| biological process | RNA biosynthetic process | 1 |
| biological process | uropod organization | 1 |
| biological process | response to desiccation | 1 |
| biological process | monodictyphenone biosynthetic process | 1 |
| biological process | polyadenylation-dependent snoRNA 3'-end processing | 1 |
| biological process | re-entry into mitotic cell cycle after pheromone arrest | 1 |
| biological process | positive regulation of ER-associated ubiquitin-dependent protein catabolic process | 1 |
| biological process | regulation of embryonic cell shape | 1 |
| biological process | entrainment of circadian clock | 1 |
| biological process | positive regulation of catabolic process | 1 |
| biological process | regulation of neutrophil chemotaxis | 1 |
| biological process | defense response to Gram-positive bacterium | 1 |
| biological process | negative regulation of bicellular tight junction assembly | 1 |
| biological process | negative regulation of growth of symbiont in host | 1 |
| biological process | definitive hemopoiesis | 1 |
| biological process | negative regulation of cellular carbohydrate metabolic process | 1 |
| biological process | protein kinase C signaling | 1 |
| biological process | negative regulation by symbiont of host innate immune response | 1 |
| biological process | renal tubule development | 1 |
| biological process | establishment of chromosome localization | 1 |
| biological process | regulation of cAMP-mediated signaling | 1 |
| biological process | endothelial cell apoptotic process | 1 |
| biological process | response to methylmercury | 1 |
| biological process | response to selenite ion | 1 |
| biological process | snoRNA 3'-end processing | 1 |
| biological process | negative regulation of Notch signaling pathway | 1 |
| biological process | cellular polysaccharide catabolic process | 1 |
| biological process | response to epidermal growth factor | 1 |
| biological process | double-strand break repair via nonhomologous end joining | 1 |
| biological process | glutathione transport | 1 |
| biological process | ncRNA polyadenylation involved in polyadenylation-dependent ncRNA catabolic process | 1 |
| biological process | mitotic nuclear envelope disassembly | 1 |
| biological process | intraciliary transport involved in cilium morphogenesis | 1 |
| biological process | blood coagulation | 1 |
| biological process | negative regulation of transcription regulatory region DNA binding | 1 |
| biological process | 4-(trimethylammonio)butanoate transport | 1 |
| biological process | Wnt signaling pathway, calcium modulating pathway | 1 |
| biological process | water-soluble vitamin metabolic process | 1 |
| biological process | positive regulation of cellular response to hypoxia | 1 |
| biological process | replication fork arrest involved in DNA replication termination | 1 |
| biological process | myeloid cell differentiation | 1 |
| biological process | salivary gland cell autophagic cell death | 1 |
| biological process | positive regulation of toll-like receptor 3 signaling pathway | 1 |
| biological process | positive regulation of neutrophil chemotaxis | 1 |
| biological process | cellular response to freezing | 1 |
| biological process | JNK cascade | 1 |
| biological process | response to cholesterol | 1 |
| biological process | social behavior | 1 |
| biological process | negative regulation of cyclic-nucleotide phosphodiesterase activity | 1 |
| biological process | positive regulation of Arp2/3 complex-mediated actin nucleation | 1 |
| biological process | mRNA cleavage involved in gene silencing by miRNA | 1 |
| biological process | macrophage differentiation | 1 |
| biological process | negative regulation of T cell differentiation | 1 |
| biological process | positive regulation of osteoclast development | 1 |
| biological process | production of small RNA involved in gene silencing by RNA | 1 |
| biological process | post-embryonic digestive tract morphogenesis | 1 |
| biological process | diacetylchitobiose metabolic process | 1 |
| biological process | Golgi vesicle budding | 1 |
| biological process | negative regulation of myosin-light-chain-phosphatase activity | 1 |
| biological process | protein import into mitochondrial inner membrane | 1 |
| biological process | regulation of neuron death | 1 |
| biological process | antigen processing and presentation of peptide antigen via MHC class I | 1 |
| biological process | high-affinity iron ion transmembrane transport | 1 |
| biological process | negative regulation of cyclase activity | 1 |
| biological process | meiotic mismatch repair | 1 |
| biological process | response to potassium ion | 1 |
| biological process | antigen processing and presentation of endogenous peptide antigen via MHC class Ib via ER pathway, TAP-dependent | 1 |
| biological process | response to nutrient levels | 1 |
| biological process | positive regulation of intracellular estrogen receptor signaling pathway | 1 |
| biological process | xylem development | 1 |
| biological process | hexuronate transport | 1 |
| biological process | 7,8-dihydroneopterin 3'-triphosphate biosynthetic process | 1 |
| biological process | organ morphogenesis | 1 |
| biological process | sulfate assimilation via adenylyl sulfate reduction | 1 |
| biological process | germ cell development | 1 |
| biological process | negative regulation of macroautophagy | 1 |
| biological process | negative regulation of epithelial cell proliferation | 1 |
| biological process | positive regulation of JUN kinase activity | 1 |
| biological process | regulation of short-term neuronal synaptic plasticity | 1 |
| biological process | toll-like receptor signaling pathway | 1 |
| biological process | nuclear migration during mitotic telophase | 1 |
| biological process | Toll signaling pathway | 1 |
| biological process | cell wall biogenesis | 1 |
| biological process | response to activity | 1 |
| biological process | catechol-containing compound metabolic process | 1 |
| biological process | deoxyribonucleoside monophosphate catabolic process | 1 |
| biological process | ferredoxin metabolic process | 1 |
| biological process | sperm axoneme assembly | 1 |
| biological process | engulfment of apoptotic cell | 1 |
| biological process | telencephalon development | 1 |
| biological process | pigmentation | 1 |
| biological process | binding of sperm to zona pellucida | 1 |
| biological process | mitotic cell cycle phase transition | 1 |
| biological process | protein autoubiquitination | 1 |
| biological process | corrin biosynthetic process | 1 |
| biological process | RNA exon ligation | 1 |
| biological process | ferric iron import into cell | 1 |
| biological process | negative regulation of axon regeneration | 1 |
| biological process | receptor clustering | 1 |
| biological process | formation of cytoplasmic translation initiation complex | 1 |
| biological process | regulation of cellular response to oxidative stress | 1 |
| biological process | asymmetric neuroblast division | 1 |
| biological process | protein-phosphoribosyl dephospho-coenzyme A linkage | 1 |
| biological process | DNA damage response, detection of DNA damage | 1 |
| biological process | lateral element assembly | 1 |
| biological process | regulation of protein targeting to membrane | 1 |
| biological process | response to cocaine | 1 |
| biological process | feeding behavior | 1 |
| biological process | convergent extension involved in axis elongation | 1 |
| biological process | luteinizing hormone secretion | 1 |
| biological process | regulation of protein K63-linked ubiquitination | 1 |
| biological process | negative regulation of extracellular matrix organization | 1 |
| biological process | plant-type secondary cell wall biogenesis | 1 |
| biological process | DNA methylation on cytosine within a CNG sequence | 1 |
| biological process | tumor necrosis factor-mediated signaling pathway | 1 |
| biological process | activation of adenylate cyclase activity | 1 |
| biological process | prostaglandin biosynthetic process | 1 |
| biological process | positive regulation of cell junction assembly | 1 |
| biological process | cytidine catabolic process | 1 |
| biological process | ecdysis, chitin-based cuticle | 1 |
| biological process | regulation of axon extension involved in axon guidance | 1 |
| biological process | heat acclimation | 1 |
| biological process | proximal convoluted tubule development | 1 |
| biological process | protein export from nucleus | 1 |
| biological process | UDP-glucuronate metabolic process | 1 |
| biological process | ethanol metabolic process | 1 |
| biological process | response to interferon-gamma | 1 |
| biological process | positive regulation of translational elongation | 1 |
| biological process | detection of bacterium | 1 |
| biological process | protein import into peroxisome matrix, receptor recycling | 1 |
| biological process | central nervous system myelin formation | 1 |
| biological process | flavonoid glucuronidation | 1 |
| biological process | cellular response to farnesol | 1 |
| biological process | cell-substrate junction assembly | 1 |
| biological process | renal sodium ion transport | 1 |
| biological process | positive regulation of tumor necrosis factor production | 1 |
| biological process | regulated exocytosis | 1 |
| biological process | lymphangiogenesis | 1 |
| biological process | phosphatidylglycerol catabolic process | 1 |
| biological process | negative regulation of transcription by competitive promoter binding | 1 |
| biological process | negative regulation of aggregate size involved in sorocarp development | 1 |
| biological process | xanthone-containing compound biosynthetic process | 1 |
| biological process | butanediol metabolic process | 1 |
| biological process | F-9775B biosynthetic process | 1 |
| biological process | positive regulation of peroxidase activity | 1 |
| biological process | regulation of circadian rhythm | 1 |
| biological process | negative regulation of isopentenyl diphosphate biosynthetic process, methylerythritol 4-phosphate pathway | 1 |
| biological process | B cell activation | 1 |
| biological process | diadenosine polyphosphate catabolic process | 1 |
| biological process | positive regulation of MyD88-dependent toll-like receptor signaling pathway | 1 |
| biological process | sulfur utilization | 1 |
| biological process | negative regulation of peptide secretion | 1 |
| biological process | synaptic vesicle targeting | 1 |
| biological process | modulation by symbiont of host signal transduction pathway | 1 |
| biological process | 7-methylguanosine mRNA capping | 1 |
| biological process | phosphatidylserine biosynthetic process | 1 |
| biological process | 'de novo' protein folding | 1 |
| biological process | alkanesulfonate metabolic process | 1 |
| biological process | zonula adherens assembly | 1 |
| biological process | root hair elongation | 1 |
| biological process | autophagosome maturation | 1 |
| biological process | carbon catabolite activation of transcription | 1 |
| biological process | detection of mechanical stimulus involved in sensory perception of sound | 1 |
| biological process | anaerobic phenol-containing compound catabolic process | 1 |
| biological process | pyrimidine dimer repair | 1 |
| biological process | positive regulation of early endosome to late endosome transport | 1 |
| biological process | spindle organization | 1 |
| biological process | regulation of transcriptional start site selection at RNA polymerase II promoter | 1 |
| biological process | L-methionine biosynthetic process from L-homoserine via cystathionine | 1 |
| biological process | myeloid cell development | 1 |
| biological process | N-terminal peptidyl-methionine acetylation | 1 |
| biological process | protein K48-linked deubiquitination | 1 |
| biological process | peptide mating pheromone maturation involved in conjugation with cellular fusion | 1 |
| biological process | positive regulation of flower development | 1 |
| biological process | SCF-dependent proteasomal ubiquitin-dependent protein catabolic process | 1 |
| biological process | regulation of auxin mediated signaling pathway | 1 |
| biological process | chiasma assembly | 1 |
| biological process | vitamin B6 catabolic process | 1 |
| biological process | traversing start control point of mitotic cell cycle | 1 |
| biological process | Mo(VI)-molybdopterin cytosine dinucleotide biosynthetic process | 1 |
| biological process | box H/ACA snoRNP assembly | 1 |
| biological process | regulation of cyclin-dependent protein serine/threonine kinase activity | 1 |
| biological process | progesterone receptor signaling pathway | 1 |
| biological process | protein import into peroxisome matrix, docking | 1 |
| biological process | meiotic DNA recombinase assembly | 1 |
| biological process | somitogenesis | 1 |
| biological process | positive regulation of macrophage cytokine production | 1 |
| biological process | L-xylitol metabolic process | 1 |
| biological process | cellularization | 1 |
| biological process | de novo centriole assembly | 1 |
| biological process | regulation of conidium formation | 1 |
| biological process | response to caffeine | 1 |
| biological process | granulocyte differentiation | 1 |
| biological process | substrate adhesion-dependent cell spreading | 1 |
| biological process | ubiquitin-dependent protein catabolic process via the multivesicular body sorting pathway | 1 |
| biological process | establishment or maintenance of epithelial cell apical/basal polarity | 1 |
| biological process | positive regulation of cyclin-dependent protein serine/threonine kinase activity | 1 |
| biological process | long-chain fatty acid catabolic process | 1 |
| biological process | negative regulation of translation in response to stress | 1 |
| biological process | extrinsic apoptotic signaling pathway via death domain receptors | 1 |
| biological process | positive regulation of cell-substrate adhesion | 1 |
| biological process | toxin biosynthetic process | 1 |
| biological process | transcription of nuclear large rRNA transcript from RNA polymerase I promoter | 1 |
| biological process | positive regulation of cytosolic calcium ion concentration | 1 |
| biological process | regulation of gene expression by genetic imprinting | 1 |
| biological process | regulation of clathrin-mediated endocytosis | 1 |
| biological process | nuclear polyadenylation-dependent tRNA catabolic process | 1 |
| biological process | platelet activation | 1 |
| biological process | catabolism by virus of host DNA | 1 |
| biological process | negative regulation of JAK-STAT cascade | 1 |
| biological process | interstrand cross-link repair | 1 |
| biological process | maturation of 5.8S rRNA | 1 |
| biological process | positive regulation of protein kinase B signaling | 1 |
| biological process | I-kappaB phosphorylation | 1 |
| biological process | kynurenine metabolic process | 1 |
| biological process | sulfur compound transport | 1 |
| biological process | positive regulation of cell-cell adhesion mediated by cadherin | 1 |
| biological process | positive regulation of cellular response to oxidative stress | 1 |
| biological process | propanediol catabolic process | 1 |
| biological process | nitric oxide biosynthetic process | 1 |
| biological process | vulval location | 1 |
| biological process | heparin catabolic process | 1 |
| biological process | negative regulation of protein autophosphorylation | 1 |
| biological process | high-affinity potassium ion import | 1 |
| biological process | regulation of angiogenesis | 1 |
| biological process | RNA import into nucleus | 1 |
| biological process | regulation of ethanol catabolic process | 1 |
| biological process | benzoate catabolic process via CoA ligation | 1 |
| biological process | glucosylceramide biosynthetic process | 1 |
| biological process | vagus nerve morphogenesis | 1 |
| biological process | behavioral response to cocaine | 1 |
| biological process | glutathione catabolic process | 1 |
| biological process | keratinocyte differentiation | 1 |
| biological process | cellular response to sucrose stimulus | 1 |
| biological process | CTP salvage | 1 |
| biological process | bicarbonate transport | 1 |
| biological process | organophosphate ester transport | 1 |
| biological process | positive regulation of hair follicle development | 1 |
| biological process | glycoprotein ERAD pathway | 1 |
| biological process | valine metabolic process | 1 |
| biological process | sperm chromatin condensation | 1 |
| biological process | negative regulation of cytokinesis | 1 |
| biological process | cellular modified amino acid metabolic process | 1 |
| biological process | multi-ciliated epithelial cell differentiation | 1 |
| biological process | regulation of apoptosis involved in tissue homeostasis | 1 |
| biological process | N-terminal protein amino acid methylation | 1 |
| biological process | cellular response to ammonium ion | 1 |
| biological process | actin filament severing | 1 |
| biological process | somatic cell DNA recombination | 1 |
| biological process | fungal-type cell wall polysaccharide biosynthetic process | 1 |
| biological process | positive regulation of mitotic centrosome separation | 1 |
| biological process | erythrocyte maturation | 1 |
| biological process | protein trimerization | 1 |
| biological process | U5 snRNA 3'-end processing | 1 |
| biological process | anterior/posterior pattern specification, imaginal disc | 1 |
| biological process | peptidyl-lysine acetylation | 1 |
| biological process | membrane depolarization | 1 |
| biological process | positive regulation of blood coagulation | 1 |
| biological process | positive regulation by host of symbiont catalytic activity | 1 |
| biological process | negative regulation of transmembrane transport | 1 |
| biological process | negative regulation of pinocytosis | 1 |
| biological process | activation of cysteine-type endopeptidase activity involved in apoptotic process | 1 |
| biological process | regulation of response to salt stress | 1 |
| biological process | regulation of Notch signaling pathway | 1 |
| biological process | high-density lipoprotein particle remodeling | 1 |
| biological process | regulation of cell proliferation involved in imaginal disc-derived wing morphogenesis | 1 |
| biological process | reverse cholesterol transport | 1 |
| biological process | Golgi to endosome transport | 1 |
| biological process | regulation of beta-glucan biosynthetic process | 1 |
| biological process | response to anoxia | 1 |
| biological process | cellular response to growth factor stimulus | 1 |
| biological process | P granule organization | 1 |
| biological process | para-aminobenzoic acid biosynthetic process | 1 |
| biological process | positive regulation of nitric oxide biosynthetic process | 1 |
| biological process | alanine transport | 1 |
| biological process | macrophage migration inhibitory factor signaling pathway | 1 |
| biological process | interaction with host | 1 |
| biological process | positive regulation of multicellular organism growth | 1 |
| biological process | response to cAMP | 1 |
| biological process | glucose mediated signaling pathway | 1 |
| biological process | bone mineralization | 1 |
| biological process | mRNA cis splicing, via spliceosome | 1 |
| biological process | phytochelatin import into vacuole | 1 |
| biological process | histone H2B conserved C-terminal lysine ubiquitination | 1 |
| biological process | transition between fast and slow fiber | 1 |
| biological process | glucosinolate biosynthetic process | 1 |
| biological process | ncRNA polyadenylation | 1 |
| biological process | protein secretion by the type V secretion system | 1 |
| biological process | glycolytic process through glucose-6-phosphate | 1 |
| biological process | ncRNA catabolic process | 1 |
| biological process | positive regulation of heterochromatin assembly | 1 |
| biological process | regulation of cellular carbohydrate catabolic process | 1 |
| biological process | DNA methylation involved in gamete generation | 1 |
| biological process | estrogen biosynthetic process | 1 |
| biological process | protein deubiquitination involved in ubiquitin-dependent protein catabolic process | 1 |
| biological process | regulation of protein export from nucleus | 1 |
| biological process | positive regulation of transporter activity | 1 |
| biological process | microtubule polymerization | 1 |
| biological process | atrazine metabolic process | 1 |
| biological process | cellular response to extracellular stimulus | 1 |
| biological process | xylulose biosynthetic process | 1 |
| biological process | negative regulation of fat cell differentiation | 1 |
| biological process | response to flooding | 1 |
| biological process | insulin secretion | 1 |
| biological process | acetoin catabolic process | 1 |
| biological process | natural killer cell activation involved in immune response | 1 |
| biological process | negative regulation of release of cytochrome c from mitochondria | 1 |
| biological process | regulation of ARF protein signal transduction | 1 |
| biological process | regulation of ERK1 and ERK2 cascade | 1 |
| biological process | vacuolar sequestering | 1 |
| biological process | embryo sac development | 1 |
| biological process | lateral root formation | 1 |
| biological process | dGTP biosynthetic process from dGDP | 1 |
| biological process | citrulline metabolic process | 1 |
| biological process | cobalamin metabolic process | 1 |
| biological process | negative regulation of cyclin-dependent protein serine/threonine kinase by cyclin degradation | 1 |
| biological process | pre-replicative complex assembly involved in nuclear cell cycle DNA replication | 1 |
| biological process | negative regulation of cAMP biosynthetic process | 1 |
| biological process | negative regulation of extrinsic apoptotic signaling pathway | 1 |
| biological process | heterochromatin maintenance involved in chromatin silencing | 1 |
| biological process | regulation of heterochromatin domain assembly | 1 |
| biological process | regulation of heterochromatin island assembly | 1 |
| biological process | slug development involved in sorocarp development | 1 |
| biological process | phytochelatin-metal complex formation | 1 |
| biological process | regulation of superoxide dismutase activity | 1 |
| biological process | L-altrarate catabolic process | 1 |
| biological process | hyaluronan catabolic process | 1 |
| biological process | negative regulation of cytolysis by symbiont of host cells | 1 |
| biological process | sorbitol metabolic process | 1 |
| biological process | positive regulation of peptidyl-serine phosphorylation of STAT protein | 1 |
| biological process | barbed-end actin filament capping | 1 |
| biological process | negative regulation of endoplasmic reticulum unfolded protein response | 1 |
| biological process | apoptotic cell clearance | 1 |
| biological process | cGMP catabolic process | 1 |
| biological process | regulation of blood coagulation | 1 |
| biological process | mitotic spindle midzone assembly | 1 |
| biological process | FAD metabolic process | 1 |
| biological process | mitotic centrosome separation | 1 |
| biological process | eisosome assembly | 1 |
| biological process | positive regulation of synaptic transmission, dopaminergic | 1 |
| biological process | establishment of T cell polarity | 1 |
| biological process | negative regulation of gene silencing | 1 |
| biological process | glycoprotein transport | 1 |
| biological process | raffinose catabolic process | 1 |
| biological process | thymine catabolic process | 1 |
| biological process | negative regulation of epidermal growth factor receptor signaling pathway | 1 |
| biological process | ncRNA processing | 1 |
| biological process | negative regulation of natural killer cell differentiation involved in immune response | 1 |
| biological process | trigeminal nerve morphogenesis | 1 |
| biological process | asymmetric Golgi ribbon formation | 1 |
| biological process | lactation | 1 |
| biological process | thigmotaxis | 1 |
| biological process | platelet aggregation | 1 |
| biological process | amino acid export | 1 |
| biological process | dGDP catabolic process | 1 |
| biological process | regulation of nucleosome density | 1 |
| biological process | positive regulation of cell adhesion molecule production | 1 |
| biological process | positive regulation of G-protein coupled receptor protein signaling pathway | 1 |
| biological process | embryonic axis specification | 1 |
| biological process | actin filament reorganization | 1 |
| biological process | histone H3-K4 dimethylation | 1 |
| biological process | peroxisomal long-chain fatty acid import | 1 |
| biological process | negative thymic T cell selection | 1 |
| biological process | oxidative single-stranded DNA demethylation | 1 |
| biological process | positive regulation of NIK/NF-kappaB signaling | 1 |
| biological process | phosphorylated carbohydrate dephosphorylation | 1 |
| biological process | spicule insertion | 1 |
| biological process | pantothenate catabolic process | 1 |
| biological process | hydrogen peroxide biosynthetic process | 1 |
| biological process | ovarian nurse cell to oocyte transport | 1 |
| biological process | chromatin remodeling at centromere | 1 |
| biological process | regulation of mitotic spindle assembly | 1 |
| biological process | actin polymerization or depolymerization | 1 |
| biological process | regulation of cell morphogenesis | 1 |
| biological process | cellular response to dexamethasone stimulus | 1 |
| biological process | glycolytic fermentation to butanediol | 1 |
| biological process | modulation by symbiont of host phagocytosis | 1 |
| biological process | negative regulation of transcription elongation from RNA polymerase II promoter | 1 |
| biological process | positive regulation of myoblast fusion | 1 |
| biological process | negative regulation of chromosome organization | 1 |
| biological process | maintenance of cell polarity | 1 |
| biological process | regulation of histone H3-K27 acetylation | 1 |
| biological process | osmosensory signaling pathway via Sho1 osmosensor | 1 |
| biological process | mRNA splicing via endonucleolytic cleavage and ligation involved in unfolded protein response | 1 |
| biological process | regulation of gene expression, epigenetic | 1 |
| biological process | retrograde transport, vesicle recycling within Golgi | 1 |
| biological process | RNA folding | 1 |
| biological process | protein kinase A signaling | 1 |
| biological process | spliceosomal snRNP assembly | 1 |
| biological process | vanillin biosynthetic process | 1 |
| biological process | dephosphorylation of RNA polymerase II C-terminal domain | 1 |
| biological process | regulation of systemic arterial blood pressure | 1 |
| biological process | carboxylic acid catabolic process | 1 |
| biological process | tetrose metabolic process | 1 |
| biological process | heme O biosynthetic process | 1 |
| biological process | gluconate transport | 1 |
| biological process | phytochelatin 2 import into vacuole | 1 |
| biological process | vanillin catabolic process | 1 |
| biological process | cilium movement involved in cell motility | 1 |
| biological process | positive regulation of translational termination | 1 |
| biological process | lead ion transport | 1 |
| biological process | T cell activation involved in immune response | 1 |
| biological process | melanin metabolic process | 1 |
| biological process | regulation of endocytosis | 1 |
| biological process | negative regulation of DNA endoreduplication | 1 |
| biological process | histidine homeostasis | 1 |
| biological process | tRNA export from nucleus | 1 |
| biological process | regulation of snoRNA processing | 1 |
| biological process | movement in host | 1 |
| biological process | keratinocyte development | 1 |
| biological process | sensory perception of mechanical stimulus | 1 |
| biological process | very long-chain fatty acid catabolic process | 1 |
| biological process | antifungal humoral response | 1 |
| biological process | RNA splicing, via transesterification reactions | 1 |
| biological process | antigen processing and presentation of exogenous protein antigen via MHC class Ib, TAP-dependent | 1 |
| biological process | polarity specification of anterior/posterior axis | 1 |
| biological process | organic acid catabolic process | 1 |
| biological process | mitotic DNA damage checkpoint | 1 |
| biological process | positive regulation by host of viral genome replication | 1 |
| biological process | zygotic specification of dorsal/ventral axis | 1 |
| biological process | cellular response to glucose-phosphate stress | 1 |
| biological process | ubiquinone biosynthetic process via 3,4-dihydroxy-5-polyprenylbenzoate | 1 |
| biological process | gamma-aminobutyric acid transport | 1 |
| biological process | regulation of ion homeostasis | 1 |
| biological process | regulation of mRNA export from nucleus | 1 |
| biological process | T cell activation | 1 |
| biological process | interphase microtubule nucleation by interphase microtubule organizing center | 1 |
| biological process | error-free translesion synthesis | 1 |
| biological process | ectoine catabolic process | 1 |
| biological process | spinal cord motor neuron differentiation | 1 |
| biological process | positive regulation of B cell activation | 1 |
| biological process | pattern recognition receptor signaling pathway | 1 |
| biological process | phagocytosis, recognition | 1 |
| biological process | Leydig cell differentiation | 1 |
| biological process | neurotransmitter transport | 1 |
| biological process | regulation of actin filament polymerization | 1 |
| biological process | cellular response to exogenous dsRNA | 1 |
| biological process | RNA fragment catabolic process | 1 |
| biological process | intestinal epithelial structure maintenance | 1 |
| biological process | adult locomotory behavior | 1 |
| biological process | F-9775A biosynthetic process | 1 |
| biological process | protein import into chloroplast thylakoid membrane | 1 |
| biological process | NADP catabolic process | 1 |
| biological process | mitochondria-associated ubiquitin-dependent protein catabolic process | 1 |
| biological process | positive regulation of translation in response to stress | 1 |
| biological process | nuclear envelope organization | 1 |
| biological process | oligopeptide export from mitochondrion | 1 |
| biological process | actomyosin contractile ring actin filament bundle assembly | 1 |
| biological process | negative regulation of RNA export from nucleus | 1 |
| biological process | cellular response to lithium ion | 1 |
| biological process | glucose 1-phosphate metabolic process | 1 |
| biological process | signal transduction in response to DNA damage | 1 |
| biological process | retina morphogenesis in camera-type eye | 1 |
| biological process | dissemination or transmission of symbiont from host by vector | 1 |
| biological process | regulation of glucose transport | 1 |
| biological process | heart looping | 1 |
| biological process | glucomannan metabolic process | 1 |
| biological process | glycosylceramide catabolic process | 1 |
| biological process | protein localization to membrane raft | 1 |
| biological process | histone H3-K4 trimethylation | 1 |
| biological process | polynucleotide 3' dephosphorylation | 1 |
| biological process | regulation of dendritic spine development | 1 |
| biological process | polar nucleus fusion | 1 |
| biological process | glucomannan catabolic process | 1 |
| biological process | post-translational protein acetylation | 1 |
| biological process | cell communication | 1 |
| biological process | budding cell isotropic bud growth | 1 |
| biological process | dUTP biosynthetic process | 1 |
| biological process | oviduct morphogenesis | 1 |
| biological process | receptor-mediated endocytosis of virus by host cell | 1 |
| biological process | pyrimidine deoxyribonucleotide salvage | 1 |
| biological process | copper ion import | 1 |
| biological process | regulation of axon diameter | 1 |
| biological process | purine nucleotide interconversion | 1 |
| biological process | histone H3-K36 demethylation | 1 |
| biological process | retinol metabolic process | 1 |
| biological process | positive regulation of adaptive immune response | 1 |
| biological process | compound eye corneal lens development | 1 |
| biological process | ventral spinal cord development | 1 |
| biological process | regulation of vesicle fusion | 1 |
| biological process | positive regulation of transferase activity | 1 |
| biological process | testosterone biosynthetic process | 1 |
| biological process | regulation of cellular organohalogen metabolic process | 1 |
| biological process | positive regulation of Notch signaling pathway | 1 |
| biological process | oogenesis | 1 |
| biological process | meiotic DNA double-strand break processing | 1 |
| biological process | negative regulation of macrophage derived foam cell differentiation | 1 |
| biological process | entry of bacterium into host cell | 1 |
| biological process | hemoglobin catabolic process | 1 |
| biological process | positive regulation of actin nucleation | 1 |
| biological process | ferricrocin biosynthetic process | 1 |
| biological process | response to ethylene | 1 |
| biological process | acetoin metabolic process | 1 |
| biological process | negative regulation of muscle contraction | 1 |
| biological process | regulation of antimicrobial peptide biosynthetic process | 1 |
| biological process | cytoplasmic sequestering of NF-kappaB | 1 |
| biological process | transcription from RNA polymerase I promoter | 1 |
| biological process | protein arginylation | 1 |
| biological process | D-arabitol catabolic process to xylulose 5-phosphate | 1 |
| biological process | pyridoxal metabolic process | 1 |
| biological process | olfactory bulb development | 1 |
| biological process | purine nucleotide salvage | 1 |
| biological process | protein targeting to ER | 1 |
| biological process | cardiac muscle cell development | 1 |
| biological process | regulation of exocytosis | 1 |
| biological process | regulation of arginine catabolic process | 1 |
| biological process | snRNA processing | 1 |
| biological process | camera-type eye photoreceptor cell differentiation | 1 |
| biological process | L-methionine biosynthetic process from methionine sulphoxide | 1 |
| biological process | cardiac muscle hypertrophy in response to stress | 1 |
| biological process | regulation of telomere maintenance via telomerase | 1 |
| biological process | centromere complex assembly | 1 |
| biological process | 7-methylguanosine biosynthetic process | 1 |
| biological process | nicotianamine biosynthetic process | 1 |
| biological process | cellular response to nickel ion | 1 |
| biological process | neuron development | 1 |
| biological process | regulation of timing of transition from vegetative to reproductive phase | 1 |
| biological process | peripheral nervous system development | 1 |
| biological process | antigen processing and presentation of exogenous peptide antigen via MHC class I | 1 |
| biological process | odontogenesis of dentin-containing tooth | 1 |
| biological process | negative regulation of sorocarp spore cell differentiation | 1 |
| biological process | establishment of localization by movement along microtubule | 1 |
| biological process | pexophagy | 1 |
| biological process | response to organophosphorus | 1 |
| biological process | signal transduction involved in filamentous growth | 1 |
| biological process | negative regulation of histone exchange | 1 |
| biological process | type I hypersensitivity | 1 |
| biological process | post-embryonic root development | 1 |
| biological process | S-methylmethionine cycle | 1 |
| biological process | B cell activation involved in immune response | 1 |
| biological process | ectopic germ cell programmed cell death | 1 |
| biological process | plasma membrane raft assembly | 1 |
| biological process | CAAX-box protein maturation | 1 |
| biological process | regulation of gamma-delta T cell differentiation | 1 |
| biological process | establishment or maintenance of cell polarity | 1 |
| biological process | centrosome cycle | 1 |
| biological process | cognition | 1 |
| biological process | bipolar cellular bud site selection | 1 |
| biological process | I-kappaB kinase/NF-kappaB signaling | 1 |
| biological process | negative regulation of ion transmembrane transporter activity | 1 |
| biological process | regulation of centrosome duplication | 1 |
| biological process | nuclear-transcribed mRNA catabolic process, meiosis-specific transcripts | 1 |
| biological process | auxin polar transport | 1 |
| biological process | positive regulation of cytoplasmic translation | 1 |
| biological process | regulation of keratinocyte differentiation | 1 |
| biological process | ATP-dependent chromatin remodeling | 1 |
| biological process | negative regulation of interleukin-6 biosynthetic process | 1 |
| biological process | maturation of LSU-rRNA from tricistronic rRNA transcript (SSU-rRNA, 5.8S rRNA, LSU-rRNA) | 1 |
| biological process | avoidance of host defenses | 1 |
| biological process | heterophilic cell-cell adhesion via plasma membrane cell adhesion molecules | 1 |
| biological process | dibenzofuran catabolic process | 1 |
| biological process | positive regulation of adherens junction organization | 1 |
| biological process | positive regulation of oligodendrocyte differentiation | 1 |
| biological process | receptor localization to synapse | 1 |
| biological process | cellular response to estradiol stimulus | 1 |
| biological process | neuromuscular process controlling posture | 1 |
| biological process | vascular wound healing | 1 |
| biological process | octopine catabolic process | 1 |
| biological process | nucleotide-excision repair, DNA gap filling | 1 |
| biological process | RNA interference | 1 |
| biological process | epithelial to mesenchymal transition involved in endocardial cushion formation | 1 |
| biological process | mitochondrial translational elongation | 1 |
| biological process | regulation of glycolytic process by negative regulation of transcription from RNA polymerase II promoter | 1 |
| biological process | pectoral fin development | 1 |
| biological process | mating behavior | 1 |
| biological process | response to estradiol | 1 |
| biological process | cytoplasmic mRNA processing body assembly | 1 |
| biological process | glucose-6-phosphate transport | 1 |
| biological process | regulation of lipid transport by negative regulation of transcription from RNA polymerase II promoter | 1 |
| biological process | sequestering of triglyceride | 1 |
| biological process | D-serine metabolic process | 1 |
| biological process | protein galactosylation | 1 |
| biological process | enterobactin catabolic process | 1 |
| biological process | negative regulation of G1/S transition of mitotic cell cycle | 1 |
| biological process | negative regulation of DNA-dependent DNA replication | 1 |
| biological process | histone H4-K16 acetylation | 1 |
| biological process | regulation of mitochondrial membrane potential | 1 |
| biological process | pentose biosynthetic process | 1 |
| biological process | myosin filament organization | 1 |
| biological process | cellular response to gravity | 1 |
| biological process | nuclear-transcribed mRNA catabolic process, non-stop decay | 1 |
| biological process | cellular response to interferon-gamma | 1 |
| biological process | nicotine catabolic process | 1 |
| biological process | glucose homeostasis | 1 |
| biological process | neomycin biosynthetic process | 1 |
| biological process | gallate catabolic process | 1 |
| biological process | toxin transport | 1 |
| biological process | UDP-D-galactose biosynthetic process | 1 |
| biological process | cadmium ion import into vacuole | 1 |
| biological process | carbon dioxide transport | 1 |
| biological process | complement activation, classical pathway | 1 |
| biological process | regulation of strand invasion | 1 |
| biological process | hexuronide transport | 1 |
| biological process | formin-nucleated actin cable assembly | 1 |
| biological process | fibroblast growth factor receptor signaling pathway | 1 |
| biological process | cellular nitrogen compound biosynthetic process | 1 |
| biological process | negative regulation of glucocorticoid receptor signaling pathway | 1 |
| biological process | glossopharyngeal nerve morphogenesis | 1 |
| biological process | thermotaxis | 1 |
| biological process | cholesterol efflux | 1 |
| biological process | chondrocyte development | 1 |
| biological process | regulation of DNA methylation | 1 |
| biological process | diaphragm contraction | 1 |
| biological process | adenylate cyclase-activating G-protein coupled receptor signaling pathway | 1 |
| biological process | negative regulation of TOR signaling | 1 |
| biological process | spleen development | 1 |
| biological process | positive regulation of complement activation | 1 |
| biological process | regulation of catecholamine metabolic process | 1 |
| biological process | endosome organization | 1 |
| biological process | photosynthetic electron transport in photosystem II | 1 |
| biological process | midgut development | 1 |
| biological process | mitotic DNA replication | 1 |
| biological process | detoxification of zinc ion | 1 |
| biological process | regulation of transmembrane transporter activity | 1 |
| biological process | mitotic chromosome movement towards spindle pole | 1 |
| biological process | hippocampus development | 1 |
| biological process | regulation of autophagosome assembly | 1 |
| biological process | embryonic pattern specification | 1 |
| biological process | (R)-carnitine transmembrane transport | 1 |
| biological process | establishment or maintenance of polarity of follicular epithelium | 1 |
| biological process | trisporic acid biosynthetic process | 1 |
| biological process | grooming behavior | 1 |
| biological process | nor-spermidine biosynthetic process | 1 |
| biological process | L-kynurenine catabolic process | 1 |
| biological process | negative regulation of intrinsic apoptotic signaling pathway in response to DNA damage by p53 class mediator | 1 |
| biological process | heteroduplex formation | 1 |
| biological process | meiotic DNA repair synthesis | 1 |
| biological process | adult behavior | 1 |
| biological process | humoral immune response | 1 |
| biological process | ascospore wall chitin catabolic process | 1 |
| biological process | protoporphyrinogen IX metabolic process | 1 |
| biological process | regulation of protein autoubiquitination | 1 |
| biological process | protein folding in endoplasmic reticulum | 1 |
| biological process | positive regulation of T cell mediated cytotoxicity | 1 |
| biological process | protection from non-homologous end joining at telomere | 1 |
| biological process | exploration behavior | 1 |
| biological process | cellular detoxification of nitrogen compound | 1 |
| biological process | sexual sporulation resulting in formation of a cellular spore | 1 |
| biological process | iron assimilation by reduction and transport | 1 |
| biological process | regulation of epithelial cell migration, open tracheal system | 1 |
| biological process | dsRNA transport | 1 |
| biological process | blue light signaling pathway | 1 |
| biological process | gonadal mesoderm development | 1 |
| biological process | convergent extension involved in gastrulation | 1 |
| biological process | neural tube closure | 1 |
| biological process | adenine metabolic process | 1 |
| biological process | rRNA transcription | 1 |
| biological process | negative regulation of ERK1 and ERK2 cascade | 1 |
| biological process | cartilage morphogenesis | 1 |
| biological process | platelet formation | 1 |
| biological process | triglyceride catabolic process | 1 |
| biological process | mitotic telomere maintenance via semi-conservative replication | 1 |
| biological process | negative regulation of neuron apoptotic process | 1 |
| biological process | camera-type eye development | 1 |
| biological process | ITP metabolic process | 1 |
| biological process | ethylene-activated signaling pathway | 1 |
| biological process | spermatid nucleus differentiation | 1 |
| biological process | pyrimidine deoxyribonucleotide catabolic process | 1 |
| biological process | hormone transport | 1 |
| biological process | fosmidomycin transport | 1 |
| biological process | positive regulation by symbiont of host apoptotic process | 1 |
| biological process | multicellular organism aging | 1 |
| biological process | positive regulation of protein binding | 1 |
| biological process | mitotic actomyosin contractile ring assembly | 1 |
| biological process | protein localization to plasma membrane | 1 |
| biological process | O-glycan processing | 1 |
| biological process | regulation of histone H3-K9 acetylation | 1 |
| biological process | positive regulation of dendrite development | 1 |
| biological process | sulfur amino acid transport | 1 |
| biological process | positive regulation of DNA replication | 1 |
| biological process | cellular response to interferon-alpha | 1 |
| biological process | syncytium formation by plasma membrane fusion | 1 |
| biological process | regulation of establishment of endothelial barrier | 1 |
| biological process | positive regulation of calcium-mediated signaling | 1 |
| biological process | phosphocreatine metabolic process | 1 |
| biological process | protein retention in Golgi apparatus | 1 |
| biological process | regulation of immune system process | 1 |
| biological process | galactonate metabolic process | 1 |
| biological process | actin crosslink formation | 1 |
| biological process | U4 snRNA 3'-end processing | 1 |
| biological process | lipoate metabolic process | 1 |
| biological process | response to lead ion | 1 |
| biological process | regulation of smooth muscle cell differentiation | 1 |
| biological process | cellular response to L-ascorbic acid | 1 |
| biological process | nicotinamide nucleotide metabolic process | 1 |
| biological process | pre-miRNA processing | 1 |
| biological process | viral life cycle | 1 |
| biological process | clathrin coat assembly | 1 |
| biological process | regulation of trehalose metabolic process | 1 |
| biological process | aldehyde catabolic process | 1 |
| biological process | regulation of myosin-light-chain-phosphatase activity | 1 |
| biological process | positive regulation of p38MAPK cascade | 1 |
| biological process | negative regulation of filamentous growth of a population of unicellular organisms | 1 |
| biological process | histone lysine methylation | 1 |
| biological process | pyrimidine ribonucleoside biosynthetic process | 1 |
| biological process | glycerophosphate shuttle | 1 |
| biological process | enucleate erythrocyte differentiation | 1 |
| biological process | thyroid hormone generation | 1 |
| biological process | determination of dorsal/ventral asymmetry | 1 |
| biological process | post-embryonic organ development | 1 |
| biological process | embryonic cranial skeleton morphogenesis | 1 |
| biological process | positive regulation of termination of RNA polymerase II transcription, poly(A)-coupled | 1 |
| biological process | negative regulation of barrier septum assembly | 1 |
| biological process | axis elongation | 1 |
| biological process | spliceosomal complex assembly | 1 |
| biological process | mitochondrial fission | 1 |
| biological process | ether lipid metabolic process | 1 |
| biological process | negative regulation of cell migration | 1 |
| biological process | positive regulation of heterotypic cell-cell adhesion | 1 |
| biological process | negative regulation of glucose import | 1 |
| biological process | microcin B17 transport | 1 |
| biological process | arsenate ion transmembrane transport | 1 |
| biological process | endoplasmic reticulum tubular network organization | 1 |
| biological process | regulation of mitotic sister chromatid separation | 1 |
| biological process | protein localization to actin cytoskeleton | 1 |
| biological process | 3-hydroxyphenylpropionic acid transport | 1 |
| biological process | positive regulation of protein complex assembly | 1 |
| biological process | male mating behavior | 1 |
| biological process | fungal-type cell wall beta-glucan metabolic process | 1 |
| biological process | regulation of beta-lactamase activity | 1 |
| biological process | sialic acid transport | 1 |
| biological process | positive regulation of interferon-beta production | 1 |
| biological process | positive regulation of endocytosis | 1 |
| biological process | DNA 3' dephosphorylation involved in DNA repair | 1 |
| biological process | negative regulation of microtubule polymerization | 1 |
| biological process | purine ribonucleoside monophosphate biosynthetic process | 1 |
| biological process | activation of protein kinase activity | 1 |
| biological process | pronephros development | 1 |
| biological process | 3',5'-cyclic diguanylic acid metabolic process | 1 |
| biological process | mitochondrial respiratory chain complex I assembly | 1 |
| biological process | response to absence of light | 1 |
| biological process | negative regulation of apoptotic signaling pathway | 1 |
| biological process | mannose catabolic process | 1 |
| biological process | regulation of ornithine metabolic process | 1 |
| biological process | antibiotic metabolic process | 1 |
| biological process | positive regulation of lipopolysaccharide-mediated signaling pathway | 1 |
| biological process | FasL biosynthetic process | 1 |
| biological process | sodium ion export | 1 |
| biological process | benzoate transport | 1 |
| biological process | methylation-dependent chromatin silencing | 1 |
| biological process | embryonic hemopoiesis | 1 |
| biological process | unidirectional conjugation | 1 |
| biological process | establishment or maintenance of polarity of larval imaginal disc epithelium | 1 |
| biological process | negative regulation of siderophore biosynthetic process | 1 |
| biological process | regulation of nucleotide biosynthetic process | 1 |
| biological process | positive regulation of cytokine production | 1 |
| biological process | DNA replication termination | 1 |
| biological process | arginine catabolic process to proline via ornithine | 1 |
| biological process | regulation of NIK/NF-kappaB signaling | 1 |
| biological process | positive regulation of mitotic cell cycle spindle assembly checkpoint | 1 |
| biological process | positive regulation of attachment of spindle microtubules to kinetochore | 1 |
| biological process | positive regulation of peptidyl-tyrosine phosphorylation | 1 |
| biological process | membrane protein ectodomain proteolysis | 1 |
| biological process | N-glycan fucosylation | 1 |
| biological process | protein localization to chloroplast | 1 |
| biological process | positive regulation of cytokine-mediated signaling pathway | 1 |
| cellular component | cytosol | 10337 |
| cellular component | plasma membrane | 8913 |
| cellular component | cellular component | 8487 |
| cellular component | cytoplasm | 8033 |
| cellular component | membrane | 5178 |
| cellular component | integral component of membrane | 4216 |
| cellular component | integral component of plasma membrane | 3714 |
| cellular component | outer membrane-bounded periplasmic space | 1379 |
| cellular component | cell wall | 1354 |
| cellular component | intracellular | 1268 |
| cellular component | cytosolic large ribosomal subunit | 1204 |
| cellular component | nucleus | 843 |
| cellular component | intracellular membrane-bounded organelle | 735 |
| cellular component | extracellular region | 734 |
| cellular component | extracellular space | 683 |
| cellular component | mitochondrion | 598 |
| cellular component | cytosolic small ribosomal subunit | 588 |
| cellular component | cell outer membrane | 583 |
| cellular component | ATP-binding cassette (ABC) transporter complex | 578 |
| cellular component | periplasmic space | 565 |
| cellular component | ATP-binding cassette (ABC) transporter complex, substrate-binding subunit-containing | 494 |
| cellular component | DNA polymerase III complex | 469 |
| cellular component | DNA topoisomerase complex (ATP-hydrolyzing) | 440 |
| cellular component | nucleoid | 388 |
| cellular component | excinuclease repair complex | 358 |
| cellular component | chloroplast | 341 |
| cellular component | provirus | 336 |
| cellular component | DNA-directed RNA polymerase complex | 282 |
| cellular component | extracellular exosome | 273 |
| cellular component | protein acetyltransferase complex | 260 |
| cellular component | small ribosomal subunit | 259 |
| cellular component | cytoplasmic side of plasma membrane | 252 |
| cellular component | Type I site-specific deoxyribonuclease complex | 240 |
| cellular component | ribosome | 219 |
| cellular component | ribonucleoside-diphosphate reductase complex | 218 |
| cellular component | Golgi apparatus | 201 |
| cellular component | proton-transporting ATP synthase complex, catalytic core F(1) | 184 |
| cellular component | bacterial nucleoid | 178 |
| cellular component | viral assembly intermediate | 177 |
| cellular component | cell surface | 168 |
| cellular component | acetyl-CoA carboxylase complex | 167 |
| cellular component | nucleolus | 160 |
| cellular component | mitochondrial matrix | 156 |
| cellular component | chloroplast stroma | 143 |
| cellular component | chromosome | 142 |
| cellular component | outer membrane | 137 |
| cellular component | phenylalanine-tRNA ligase complex | 136 |
| cellular component | pilus | 132 |
| cellular component | intracellular ribonucleoprotein complex | 131 |
| cellular component | endoplasmic reticulum | 130 |
| cellular component | DNA replication factor C complex | 129 |
| cellular component | vacuole | 126 |
| cellular component | mismatch repair complex | 122 |
| cellular component | EKC/KEOPS complex | 120 |
| cellular component | endopeptidase Clp complex | 120 |
| cellular component | plasma membrane proton-transporting ATP synthase complex, catalytic core F(1) | 106 |
| cellular component | nucleoplasm | 102 |
| cellular component | exodeoxyribonuclease VII complex | 102 |
| cellular component | glycine reductase complex | 100 |
| cellular component | exosome (RNase complex) | 90 |
| cellular component | bacterial-type flagellum | 86 |
| cellular component | bacterial-type flagellum hook | 86 |
| cellular component | cell division site | 86 |
| cellular component | plasma membrane proton-transporting ATP synthase complex, coupling factor F(o) | 78 |
| cellular component | type II protein secretion system complex | 77 |
| cellular component | kinesin complex | 77 |
| cellular component | phagocytic vesicle | 76 |
| cellular component | virion | 74 |
| cellular component | proteinaceous extracellular matrix | 74 |
| cellular component | cell septum | 72 |
| cellular component | cytosolic ribosome | 70 |
| cellular component | plasma membrane respiratory chain complex I | 69 |
| cellular component | Elongator holoenzyme complex | 68 |
| cellular component | ferredoxin hydrogenase complex | 66 |
| cellular component | collagen trimer | 66 |
| cellular component | ribonuclease H2 complex | 66 |
| cellular component | endoplasmic reticulum membrane | 65 |
| cellular component | tRNA methyltransferase complex | 64 |
| cellular component | Golgi membrane | 63 |
| cellular component | synaptonemal complex | 63 |
| cellular component | cytoskeleton | 62 |
| cellular component | cytosolic DNA-directed RNA polymerase complex | 62 |
| cellular component | apicoplast | 61 |
| cellular component | intrinsic component of plasma membrane | 60 |
| cellular component | Holliday junction resolvase complex | 58 |
| cellular component | pore complex | 58 |
| cellular component | polyphosphate kinase complex | 58 |
| cellular component | carbamoyl-phosphate synthase complex | 57 |
| cellular component | GroEL-GroES complex | 57 |
| cellular component | lysosome | 57 |
| cellular component | apoplast | 54 |
| cellular component | alpha DNA polymerase:primase complex | 53 |
| cellular component | large ribosomal subunit | 53 |
| cellular component | protein-DNA complex | 51 |
| cellular component | cytochrome complex | 50 |
| cellular component | plastid | 50 |
| cellular component | protein complex | 49 |
| cellular component | plasmodesma | 48 |
| cellular component | myosin complex | 48 |
| cellular component | chloroplast envelope | 48 |
| cellular component | anaerobic ribonucleoside-triphosphate reductase complex | 45 |
| cellular component | acetolactate synthase complex | 45 |
| cellular component | centrosome | 45 |
| cellular component | cell | 45 |
| cellular component | ER to Golgi transport vesicle membrane | 44 |
| cellular component | mitochondrial inner membrane | 44 |
| cellular component | Gram-negative-bacterium-type cell wall | 42 |
| cellular component | plasma membrane proton-transporting ATP synthase complex | 42 |
| cellular component | Holliday junction helicase complex | 42 |
| cellular component | S-layer | 41 |
| cellular component | cytosolic pyruvate dehydrogenase complex | 38 |
| cellular component | oxoglutarate dehydrogenase complex | 38 |
| cellular component | rhoptry | 38 |
| cellular component | endoplasmic reticulum lumen | 37 |
| cellular component | cytoplasmic vesicle | 37 |
| cellular component | integral component of cell outer membrane | 37 |
| cellular component | citrate lyase complex | 36 |
| cellular component | fungal-type vacuole membrane | 36 |
| cellular component | endospore-forming forespore | 35 |
| cellular component | chloroplast thylakoid membrane | 35 |
| cellular component | replication fork | 35 |
| cellular component | extrinsic component of cytoplasmic side of plasma membrane | 34 |
| cellular component | vacuolar membrane | 34 |
| cellular component | extracellular matrix | 34 |
| cellular component | virus tail | 34 |
| cellular component | signal recognition particle, plasma membrane targeting | 34 |
| cellular component | viral portal complex | 33 |
| cellular component | vacuolar proton-transporting V-type ATPase complex | 33 |
| cellular component | peroxisome | 33 |
| cellular component | cell pole | 32 |
| cellular component | peptidoglycan-based cell wall | 32 |
| cellular component | integral component of external side of plasma membrane | 32 |
| cellular component | viral procapsid | 32 |
| cellular component | beta-galactosidase complex | 31 |
| cellular component | proton-transporting ATP synthase complex, coupling factor F(o) | 31 |
| cellular component | endomembrane system | 31 |
| cellular component | glycine cleavage complex | 31 |
| cellular component | perinuclear region of cytoplasm | 30 |
| cellular component | endosome | 30 |
| cellular component | formate dehydrogenase complex | 30 |
| cellular component | ribosomal subunit | 30 |
| cellular component | NADH dehydrogenase complex | 30 |
| cellular component | vacuolar proton-transporting V-type ATPase, V1 domain | 29 |
| cellular component | glycerol-3-phosphate dehydrogenase complex | 28 |
| cellular component | exodeoxyribonuclease V complex | 28 |
| cellular component | pyruvate dehydrogenase complex | 27 |
| cellular component | nucleolar ribonuclease P complex | 27 |
| cellular component | nuclear envelope | 26 |
| cellular component | bacterial-type flagellum basal body | 26 |
| cellular component | cell envelope Sec protein transport complex | 26 |
| cellular component | succinate-CoA ligase complex (ADP-forming) | 26 |
| cellular component | kinetochore | 26 |
| cellular component | basement membrane | 25 |
| cellular component | microtubule | 25 |
| cellular component | cilium | 25 |
| cellular component | nuclear membrane | 25 |
| cellular component | microtubule associated complex | 25 |
| cellular component | fungal-type cell wall | 25 |
| cellular component | mitochondrial proton-transporting ATP synthase complex, catalytic core F(1) | 24 |
| cellular component | transcription elongation factor complex | 23 |
| cellular component | neurofilament | 23 |
| cellular component | cell junction | 22 |
| cellular component | 3-isopropylmalate dehydratase complex | 22 |
| cellular component | histone acetyltransferase complex | 22 |
| cellular component | organelle inner membrane | 22 |
| cellular component | muscle myosin complex | 22 |
| cellular component | nuclear periphery | 22 |
| cellular component | intrinsic component of periplasmic side of plasma membrane | 22 |
| cellular component | ethanolamine degradation polyhedral organelle | 22 |
| cellular component | nuclear chromatin | 22 |
| cellular component | trans-Golgi network | 22 |
| cellular component | primosome complex | 22 |
| cellular component | other organism cytoplasm | 21 |
| cellular component | spore wall | 21 |
| cellular component | extrinsic component of plasma membrane | 21 |
| cellular component | early endosome | 21 |
| cellular component | condensed nuclear chromosome | 20 |
| cellular component | [Ni-Fe] hydrogenase complex | 20 |
| cellular component | cell cortex | 20 |
| cellular component | anchored component of membrane | 20 |
| cellular component | type IV pilus | 20 |
| cellular component | neuron projection | 19 |
| cellular component | viral capsid | 19 |
| cellular component | bacterial-type flagellum filament cap | 19 |
| cellular component | host cell plasma membrane | 19 |
| cellular component | lysosomal membrane | 19 |
| cellular component | intrinsic component of the cytoplasmic side of the plasma membrane | 19 |
| cellular component | bacterial-type flagellum filament | 19 |
| cellular component | Z disc | 19 |
| cellular component | anthranilate synthase complex | 19 |
| cellular component | site of double-strand break | 19 |
| cellular component | myosin filament | 18 |
| cellular component | nitrate reductase complex | 18 |
| cellular component | Cul3-RING ubiquitin ligase complex | 18 |
| cellular component | plant-type cell wall | 18 |
| cellular component | lamellipodium | 18 |
| cellular component | phosphoenolpyruvate-dependent sugar phosphotransferase complex | 18 |
| cellular component | lipid particle | 18 |
| cellular component | apical complex | 18 |
| cellular component | hyphal cell wall | 18 |
| cellular component | nuclear chromosome, telomeric region | 17 |
| cellular component | alkyl hydroperoxide reductase complex | 17 |
| cellular component | myofibril | 17 |
| cellular component | membrane raft | 17 |
| cellular component | proton-transporting two-sector ATPase complex, catalytic domain | 17 |
| cellular component | mitochondrial outer membrane | 17 |
| cellular component | TAM protein secretion complex | 17 |
| cellular component | Golgi stack | 16 |
| cellular component | chloroplast inner membrane | 16 |
| cellular component | stress fiber | 16 |
| cellular component | Bam protein complex | 16 |
| cellular component | tRNA (m1A) methyltransferase complex | 16 |
| cellular component | midbody | 16 |
| cellular component | nuclear pore | 15 |
| cellular component | axon | 15 |
| cellular component | A band | 15 |
| cellular component | synapse | 15 |
| cellular component | myelin sheath | 15 |
| cellular component | mitochondrial intermembrane space | 15 |
| cellular component | glyoxysome | 15 |
| cellular component | focal adhesion | 15 |
| cellular component | ethanolamine ammonia-lyase complex | 15 |
| cellular component | extrinsic component of membrane | 14 |
| cellular component | myosin II complex | 14 |
| cellular component | external side of plasma membrane | 14 |
| cellular component | viral terminase, large subunit | 14 |
| cellular component | neuronal cell body | 14 |
| cellular component | brush border | 14 |
| cellular component | microtubule organizing center | 14 |
| cellular component | Mre11 complex | 14 |
| cellular component | sulfite reductase complex (NADPH) | 14 |
| cellular component | chloroplast membrane | 14 |
| cellular component | virus tail, tube | 14 |
| cellular component | dimethyl sulfoxide reductase complex | 13 |
| cellular component | photoreceptor outer segment | 13 |
| cellular component | mitochondrial inner membrane peptidase complex | 13 |
| cellular component | centriole | 13 |
| cellular component | barrier septum | 13 |
| cellular component | intrinsic component of membrane | 13 |
| cellular component | chromosome, centromeric region | 13 |
| cellular component | HslUV protease complex | 13 |
| cellular component | integral component of endoplasmic reticulum membrane | 13 |
| cellular component | mitochondrial respiratory chain complex I | 13 |
| cellular component | thylakoid | 13 |
| cellular component | mitochondrial membrane | 13 |
| cellular component | DNA polymerase III, core complex | 13 |
| cellular component | chloride channel complex | 13 |
| cellular component | neurofibrillary tangle | 12 |
| cellular component | small-subunit processome | 12 |
| cellular component | intermediate filament | 12 |
| cellular component | actin cytoskeleton | 12 |
| cellular component | I band | 12 |
| cellular component | cleavage furrow | 12 |
| cellular component | fungal-type vacuole | 12 |
| cellular component | condensed nuclear chromosome kinetochore | 11 |
| cellular component | periplasmic side of cell outer membrane | 11 |
| cellular component | spindle | 11 |
| cellular component | TAT protein transport complex | 11 |
| cellular component | DNA topoisomerase IV complex | 11 |
| cellular component | mitochondrial nucleoid | 11 |
| cellular component | glycine-tRNA ligase complex | 11 |
| cellular component | plasma membrane fumarate reductase complex | 11 |
| cellular component | virus tail, baseplate | 11 |
| cellular component | plastid chromosome | 11 |
| cellular component | fungal-type vacuole lumen | 11 |
| cellular component | striated muscle thin filament | 11 |
| cellular component | respiratory chain | 11 |
| cellular component | cytoplasmic stress granule | 11 |
| cellular component | plasma membrane succinate dehydrogenase complex | 11 |
| cellular component | condensin complex | 11 |
| cellular component | magnesium chelatase complex | 11 |
| cellular component | virus tail, fiber | 10 |
| cellular component | proton-transporting V-type ATPase, V1 domain | 10 |
| cellular component | ruffle | 10 |
| cellular component | M band | 10 |
| cellular component | ciliary basal body | 10 |
| cellular component | host cell membrane | 10 |
| cellular component | eisosome | 10 |
| cellular component | type VI protein secretion system complex | 10 |
| cellular component | preribosome, large subunit precursor | 10 |
| cellular component | Golgi cisterna membrane | 10 |
| cellular component | glutamate-tRNA ligase complex | 10 |
| cellular component | respiratory chain complex III | 10 |
| cellular component | postsynaptic membrane | 10 |
| cellular component | Maurer's cleft | 10 |
| cellular component | spindle pole body | 9 |
| cellular component | serine-pyruvate aminotransferase complex | 9 |
| cellular component | axoneme | 9 |
| cellular component | axonal spine | 9 |
| cellular component | host cell cytoplasm | 9 |
| cellular component | GABA-A receptor complex | 9 |
| cellular component | endocytic vesicle | 9 |
| cellular component | mitochondrial proton-transporting ATP synthase complex | 9 |
| cellular component | cell projection | 9 |
| cellular component | NarGHI complex | 9 |
| cellular component | cytoplasmic vesicle membrane | 9 |
| cellular component | replisome | 9 |
| cellular component | microtubule cytoskeleton | 9 |
| cellular component | integral component of fungal-type vacuolar membrane | 9 |
| cellular component | plastid stroma | 9 |
| cellular component | type IV secretion system complex | 9 |
| cellular component | yeast-form cell wall | 9 |
| cellular component | COP9 signalosome | 9 |
| cellular component | DNA helicase complex | 9 |
| cellular component | nascent polypeptide-associated complex | 9 |
| cellular component | cytoplasmic, membrane-bounded vesicle | 8 |
| cellular component | glutaminase complex | 8 |
| cellular component | filamentous actin | 8 |
| cellular component | extrinsic component of periplasmic side of plasma membrane | 8 |
| cellular component | apical part of cell | 8 |
| cellular component | bacterial-type flagellum basal body, MS ring | 8 |
| cellular component | apical plasma membrane | 8 |
| cellular component | mitochondrial proton-transporting ATP synthase, catalytic core | 8 |
| cellular component | maltose transport complex | 8 |
| cellular component | bicellular tight junction | 8 |
| cellular component | anchored component of plasma membrane | 8 |
| cellular component | host cell cytosol | 8 |
| cellular component | catalytic step 2 spliceosome | 8 |
| cellular component | condensed chromosome, centromeric region | 8 |
| cellular component | nuclear outer membrane-endoplasmic reticulum membrane network | 8 |
| cellular component | intrinsic component of periplasmic side of cell outer membrane | 8 |
| cellular component | T=7 icosahedral viral capsid | 8 |
| cellular component | actin cap | 8 |
| cellular component | proton-transporting two-sector ATPase complex | 8 |
| cellular component | tripartite ATP-independent periplasmic transporter complex | 8 |
| cellular component | collagen type V trimer | 8 |
| cellular component | cytoplasmic mRNA processing body | 8 |
| cellular component | signal recognition particle | 8 |
| cellular component | striated muscle myosin thick filament | 8 |
| cellular component | 6-phosphofructokinase complex | 8 |
| cellular component | condensed chromosome | 8 |
| cellular component | mitotic spindle midzone | 8 |
| cellular component | sarcolemma | 8 |
| cellular component | rhoptry neck | 8 |
| cellular component | chromatin | 7 |
| cellular component | molybdopterin synthase complex | 7 |
| cellular component | extrachromosomal circular DNA | 7 |
| cellular component | collagen type II trimer | 7 |
| cellular component | Ndc80 complex | 7 |
| cellular component | cohesin complex | 7 |
| cellular component | junctional sarcoplasmic reticulum membrane | 7 |
| cellular component | contractile vacuole | 7 |
| cellular component | lysosomal lumen | 7 |
| cellular component | acetate CoA-transferase complex | 7 |
| cellular component | mating projection | 7 |
| cellular component | microvillus | 7 |
| cellular component | growth cone | 7 |
| cellular component | endospore coat | 7 |
| cellular component | collagen type I trimer | 7 |
| cellular component | plant-type vacuole membrane | 7 |
| cellular component | chromosome, telomeric region | 7 |
| cellular component | motile cilium | 7 |
| cellular component | endoplasmic reticulum-Golgi intermediate compartment | 7 |
| cellular component | chloroplast thylakoid | 7 |
| cellular component | phosphoribosylaminoimidazole carboxylase complex | 7 |
| cellular component | enterobactin synthetase complex | 7 |
| cellular component | iron-sulfur cluster transfer complex | 7 |
| cellular component | actomyosin | 7 |
| cellular component | late endosome | 7 |
| cellular component | polysome | 7 |
| cellular component | cis-Golgi network | 7 |
| cellular component | mitochondrial large ribosomal subunit | 7 |
| cellular component | sarcomere | 6 |
| cellular component | PML body | 6 |
| cellular component | chloroplast starch grain | 6 |
| cellular component | actin filament | 6 |
| cellular component | cell periphery | 6 |
| cellular component | rough endoplasmic reticulum | 6 |
| cellular component | uropod | 6 |
| cellular component | cytochrome o ubiquinol oxidase complex | 6 |
| cellular component | dendritic spine | 6 |
| cellular component | protein serine/threonine phosphatase complex | 6 |
| cellular component | myosin II filament | 6 |
| cellular component | anchored component of cell outer membrane | 6 |
| cellular component | NuA4 histone acetyltransferase complex | 6 |
| cellular component | nuclear matrix | 6 |
| cellular component | mitotic spindle pole body | 6 |
| cellular component | cytoplasmic ribonucleoprotein granule | 6 |
| cellular component | striated muscle dense body | 6 |
| cellular component | apical cortex | 6 |
| cellular component | spindle pole | 6 |
| cellular component | nuclear chromosome | 6 |
| cellular component | box C/D snoRNP complex | 6 |
| cellular component | mRNA cleavage and polyadenylation specificity factor complex | 6 |
| cellular component | neuromuscular junction | 6 |
| cellular component | anchored component of external side of plasma membrane | 6 |
| cellular component | amyloplast | 6 |
| cellular component | actin cortical patch | 6 |
| cellular component | mitotic spindle | 6 |
| cellular component | virus tail, tip | 6 |
| cellular component | actomyosin contractile ring | 6 |
| cellular component | cell leading edge | 6 |
| cellular component | bacterial-type flagellum basal body, distal rod, P ring | 5 |
| cellular component | nuclear telomeric heterochromatin | 5 |
| cellular component | ciliary rootlet | 5 |
| cellular component | collagen type III trimer | 5 |
| cellular component | spindle microtubule | 5 |
| cellular component | pollen tube | 5 |
| cellular component | cytoplasmic replisome | 5 |
| cellular component | melanosome | 5 |
| cellular component | nuclear condensin complex | 5 |
| cellular component | troponin complex | 5 |
| cellular component | T=3 icosahedral viral capsid | 5 |
| cellular component | mitotic spindle pole | 5 |
| cellular component | keratin filament | 5 |
| cellular component | cysteine synthase complex | 5 |
| cellular component | fibril | 5 |
| cellular component | UDP-N-acetylglucosamine transferase complex | 5 |
| cellular component | chloroplast outer membrane | 5 |
| cellular component | perikaryon | 5 |
| cellular component | respiratory chain complex I | 5 |
| cellular component | nuclear pore cytoplasmic filaments | 5 |
| cellular component | viral capsid, decoration | 5 |
| cellular component | dynein complex | 5 |
| cellular component | molybdenum-iron nitrogenase complex | 5 |
| cellular component | mediator complex | 5 |
| cellular component | postsynaptic density | 5 |
| cellular component | nuclear speck | 5 |
| cellular component | tricarboxylic acid cycle enzyme complex | 5 |
| cellular component | chloroplast isoamylase complex | 5 |
| cellular component | plasma membrane proton-transporting V-type ATPase complex | 4 |
| cellular component | peroxisomal matrix | 4 |
| cellular component | dystrophin-associated glycoprotein complex | 4 |
| cellular component | transcriptional repressor complex | 4 |
| cellular component | extrinsic component of mitochondrial inner membrane | 4 |
| cellular component | phagocytic cup base | 4 |
| cellular component | single-stranded DNA-dependent ATP-dependent DNA helicase complex | 4 |
| cellular component | nuclear pore nuclear basket | 4 |
| cellular component | Set1C/COMPASS complex | 4 |
| cellular component | invadopodium membrane | 4 |
| cellular component | CCR4-NOT complex | 4 |
| cellular component | cell envelope | 4 |
| cellular component | CAF-1 complex | 4 |
| cellular component | eukaryotic translation initiation factor 4F complex | 4 |
| cellular component | cell-cell junction | 4 |
| cellular component | glutamyl-tRNA(Gln) amidotransferase complex | 4 |
| cellular component | intercalated disc | 4 |
| cellular component | pseudopodium | 4 |
| cellular component | terminal bouton | 4 |
| cellular component | 3-methyl-2-oxobutanoate dehydrogenase (lipoamide) complex | 4 |
| cellular component | cell trailing edge | 4 |
| cellular component | cytoplasmic chromosome | 4 |
| cellular component | ciliary transition fiber | 4 |
| cellular component | cytoplasmic nucleosome | 4 |
| cellular component | autophagosome | 4 |
| cellular component | aspartate carbamoyltransferase complex | 4 |
| cellular component | TRC complex | 4 |
| cellular component | nuclear inner membrane | 4 |
| cellular component | sperm flagellum | 4 |
| cellular component | pericentric heterochromatin | 4 |
| cellular component | Sin3-type complex | 4 |
| cellular component | blood microparticle | 4 |
| cellular component | collagen type IV trimer | 4 |
| cellular component | SAGA complex | 4 |
| cellular component | multivesicular body | 4 |
| cellular component | glycosome | 4 |
| cellular component | phosphopyruvate hydratase complex | 4 |
| cellular component | Swr1 complex | 4 |
| cellular component | glucosidase II complex | 4 |
| cellular component | replication fork protection complex | 4 |
| cellular component | cell tip | 4 |
| cellular component | transcription factor TFIID complex | 4 |
| cellular component | polyketide synthase complex | 4 |
| cellular component | early phagosome | 4 |
| cellular component | intrinsic component of cell outer membrane | 4 |
| cellular component | secretory granule | 4 |
| cellular component | integral component of mitochondrial outer membrane | 4 |
| cellular component | trans-Golgi network transport vesicle membrane | 4 |
| cellular component | centriolar satellite | 3 |
| cellular component | external side of cell outer membrane | 3 |
| cellular component | proteasome regulatory particle, base subcomplex | 3 |
| cellular component | intracellular cyclic nucleotide activated cation channel complex | 3 |
| cellular component | intraciliary transport particle B | 3 |
| cellular component | cohesin core heterodimer | 3 |
| cellular component | cellular bud tip | 3 |
| cellular component | respiratory chain complex IV | 3 |
| cellular component | nucleosome | 3 |
| cellular component | host cell | 3 |
| cellular component | cell wall-bounded periplasmic space | 3 |
| cellular component | central element | 3 |
| cellular component | DNA polymerase III, clamp loader complex | 3 |
| cellular component | cortical cytoskeleton | 3 |
| cellular component | L-cysteine desulfurase complex | 3 |
| cellular component | cytoplasmic microtubule | 3 |
| cellular component | pericentriolar material | 3 |
| cellular component | transmembrane transporter complex | 3 |
| cellular component | recycling endosome | 3 |
| cellular component | nitrite reductase complex [NAD(P)H] | 3 |
| cellular component | photoreceptor inner segment | 3 |
| cellular component | hemidesmosome | 3 |
| cellular component | acrosomal matrix | 3 |
| cellular component | nuclear replication fork | 3 |
| cellular component | Cdc73/Paf1 complex | 3 |
| cellular component | mating projection tip | 3 |
| cellular component | nucleotide-excision repair factor 1 complex | 3 |
| cellular component | fatty acid beta-oxidation multienzyme complex | 3 |
| cellular component | precatalytic spliceosome | 3 |
| cellular component | bacterial-type flagellum basal body, distal rod, L ring | 3 |
| cellular component | transcription factor complex | 3 |
| cellular component | mitochondrial respiratory chain complex II, succinate dehydrogenase complex (ubiquinone) | 3 |
| cellular component | extrinsic component of endoplasmic reticulum membrane | 3 |
| cellular component | nuclear mitotic cohesin complex | 3 |
| cellular component | spindle midzone | 3 |
| cellular component | imidazoleglycerol-phosphate synthase complex | 3 |
| cellular component | peroxisomal membrane | 3 |
| cellular component | host cell nucleus | 3 |
| cellular component | transcription export complex | 3 |
| cellular component | viral scaffold | 3 |
| cellular component | centrosomal corona | 3 |
| cellular component | central plaque of spindle pole body | 3 |
| cellular component | vacuolar lumen | 3 |
| cellular component | contractile ring | 3 |
| cellular component | polysomal ribosome | 3 |
| cellular component | condensed chromosome outer kinetochore | 3 |
| cellular component | inner plaque of spindle pole body | 3 |
| cellular component | protein N-acetylglucosaminyltransferase complex | 3 |
| cellular component | Mpp10 complex | 3 |
| cellular component | immunological synapse | 3 |
| cellular component | Cajal body | 3 |
| cellular component | peroxisomal importomer complex | 3 |
| cellular component | fibrillar collagen trimer | 3 |
| cellular component | viral genome | 3 |
| cellular component | food vacuole | 3 |
| cellular component | basal part of cell | 3 |
| cellular component | presynaptic active zone | 3 |
| cellular component | U4/U6 x U5 tri-snRNP complex | 3 |
| cellular component | proton-transporting V-type ATPase complex | 3 |
| cellular component | exon-exon junction complex | 3 |
| cellular component | voltage-gated potassium channel complex | 3 |
| cellular component | cellular bud neck contractile ring | 3 |
| cellular component | retromer complex | 3 |
| cellular component | pronucleus | 3 |
| cellular component | incipient cellular bud site | 3 |
| cellular component | outer dense fiber | 3 |
| cellular component | proteasome-activating nucleotidase complex | 3 |
| cellular component | cell-cell contact zone | 3 |
| cellular component | microneme | 3 |
| cellular component | flotillin complex | 3 |
| cellular component | GMP reductase complex | 3 |
| cellular component | meiotic spindle | 2 |
| cellular component | proteasome core complex | 2 |
| cellular component | bacterial-type flagellum basal body, rod | 2 |
| cellular component | integral component of thylakoid membrane | 2 |
| cellular component | U5 snRNP | 2 |
| cellular component | Elg1 RFC-like complex | 2 |
| cellular component | Rpd3S complex | 2 |
| cellular component | spitzenkorper | 2 |
| cellular component | zonula adherens | 2 |
| cellular component | vacuolar proton-transporting V-type ATPase, V0 domain | 2 |
| cellular component | lateral plasma membrane | 2 |
| cellular component | sex chromosome | 2 |
| cellular component | glycosylphosphatidylinositol-N-acetylglucosaminyltransferase (GPI-GnT) complex | 2 |
| cellular component | dolichyl-phosphate-mannose-protein mannosyltransferase complex | 2 |
| cellular component | viral envelope | 2 |
| cellular component | endosome membrane | 2 |
| cellular component | integral component of Golgi membrane | 2 |
| cellular component | histone methyltransferase complex | 2 |
| cellular component | core mediator complex | 2 |
| cellular component | T cell receptor complex | 2 |
| cellular component | stromule | 2 |
| cellular component | septate junction | 2 |
| cellular component | high-affinity iron permease complex | 2 |
| cellular component | endocytic vesicle lumen | 2 |
| cellular component | intermediate filament cytoskeleton | 2 |
| cellular component | cellular bud neck | 2 |
| cellular component | virion membrane | 2 |
| cellular component | TRAMP complex | 2 |
| cellular component | U1 snRNP | 2 |
| cellular component | SUMO-targeted ubiquitin ligase complex | 2 |
| cellular component | plasma membrane respiratory chain complex III | 2 |
| cellular component | nuclear proteasome complex | 2 |
| cellular component | Rpd3L-Expanded complex | 2 |
| cellular component | type III protein secretion system complex | 2 |
| cellular component | DNA polymerase III, clamp loader chi/psi subcomplex | 2 |
| cellular component | fatty acid synthase complex | 2 |
| cellular component | anaphase-promoting complex | 2 |
| cellular component | plastoglobule | 2 |
| cellular component | THO complex part of transcription export complex | 2 |
| cellular component | site of polarized growth | 2 |
| cellular component | interchromatin granule | 2 |
| cellular component | oxidoreductase complex | 2 |
| cellular component | U2-type prespliceosome | 2 |
| cellular component | Rpd3L complex | 2 |
| cellular component | signal recognition particle, endoplasmic reticulum targeting | 2 |
| cellular component | nuclear lamina | 2 |
| cellular component | endospore cortex | 2 |
| cellular component | DNA-directed RNA polymerase II, holoenzyme | 2 |
| cellular component | dendrite | 2 |
| cellular component | ciliary membrane | 2 |
| cellular component | receptor complex | 2 |
| cellular component | SCF ubiquitin ligase complex | 2 |
| cellular component | intrinsic component of external side of plasma membrane | 2 |
| cellular component | collagen type VII trimer | 2 |
| cellular component | aggresome | 2 |
| cellular component | Clr6 histone deacetylase complex I'' | 2 |
| cellular component | brush border membrane | 2 |
| cellular component | cytoskeleton of presynaptic active zone | 2 |
| cellular component | unconventional myosin complex | 2 |
| cellular component | protein phosphatase type 1 complex | 2 |
| cellular component | spindle pole centrosome | 2 |
| cellular component | mitochondrial envelope | 2 |
| cellular component | proton-transporting two-sector ATPase complex, proton-transporting domain | 2 |
| cellular component | single-stranded DNA-binding protein complex | 2 |
| cellular component | Snt2C complex | 2 |
| cellular component | CHRAC | 2 |
| cellular component | endoplasmic reticulum-Golgi intermediate compartment membrane | 2 |
| cellular component | CCAAT-binding factor complex | 2 |
| cellular component | eukaryotic translation initiation factor 3 complex | 2 |
| cellular component | hemoglobin complex | 2 |
| cellular component | smooth endoplasmic reticulum membrane | 2 |
| cellular component | heterochromatin | 2 |
| cellular component | cytosolic proteasome complex | 2 |
| cellular component | cell-cell adherens junction | 2 |
| cellular component | trimethylamine-N-oxide reductase complex | 2 |
| cellular component | BBSome | 2 |
| cellular component | Smc5-Smc6 complex | 2 |
| cellular component | basolateral plasma membrane | 2 |
| cellular component | phosphatidylinositol 3-kinase complex | 2 |
| cellular component | podosome | 2 |
| cellular component | succinate dehydrogenase complex | 2 |
| cellular component | spot adherens junction | 2 |
| cellular component | meiotic spindle pole body | 2 |
| cellular component | delta DNA polymerase complex | 2 |
| cellular component | Ski complex | 2 |
| cellular component | basal lamina | 2 |
| cellular component | viral tegument | 2 |
| cellular component | histone deacetylase complex | 2 |
| cellular component | Golgi cis cisterna | 2 |
| cellular component | ER to Golgi transport vesicle | 2 |
| cellular component | RNA polymerase complex | 2 |
| cellular component | extrinsic component of mitochondrial outer membrane | 2 |
| cellular component | hyphal septin ring | 1 |
| cellular component | sarcoplasmic reticulum | 1 |
| cellular component | medial cortex | 1 |
| cellular component | telomeric heterochromatin | 1 |
| cellular component | photoreceptor outer segment membrane | 1 |
| cellular component | nuclear pericentric heterochromatin | 1 |
| cellular component | fibrinogen complex | 1 |
| cellular component | B cell receptor complex | 1 |
| cellular component | Set3 complex | 1 |
| cellular component | cone cell pedicle | 1 |
| cellular component | translation initiation complex | 1 |
| cellular component | nuclear microtubule | 1 |
| cellular component | MutLalpha complex | 1 |
| cellular component | mitotic spindle astral microtubule | 1 |
| cellular component | chromosome, centromeric core domain | 1 |
| cellular component | dynactin complex | 1 |
| cellular component | host cell endosome lumen | 1 |
| cellular component | MHC class II protein complex | 1 |
| cellular component | actomyosin, actin portion | 1 |
| cellular component | MLL3/4 complex | 1 |
| cellular component | plasma membrane-derived thylakoid photosystem II | 1 |
| cellular component | U2-type spliceosomal complex | 1 |
| cellular component | U2-type catalytic step 1 spliceosome | 1 |
| cellular component | mitochondrial respiratory chain | 1 |
| cellular component | gamma DNA polymerase complex | 1 |
| cellular component | telomerase holoenzyme complex | 1 |
| cellular component | rod spherule | 1 |
| cellular component | RSC complex | 1 |
| cellular component | SMN complex | 1 |
| cellular component | cortical actin cytoskeleton | 1 |
| cellular component | merozoite dense granule | 1 |
| cellular component | ribose phosphate diphosphokinase complex | 1 |
| cellular component | extrinsic component of external side of plasma membrane | 1 |
| cellular component | collagen type IX trimer | 1 |
| cellular component | caveola | 1 |
| cellular component | adherens junction | 1 |
| cellular component | outer plaque of spindle pole body | 1 |
| cellular component | nuclear euchromatin | 1 |
| cellular component | contractile fiber | 1 |
| cellular component | riboflavin synthase complex | 1 |
| cellular component | chromoplast | 1 |
| cellular component | Golgi trans cisterna | 1 |
| cellular component | methionyl glutamyl tRNA synthetase complex | 1 |
| cellular component | ubiquitin ligase complex | 1 |
| cellular component | septin complex | 1 |
| cellular component | prospore membrane | 1 |
| cellular component | nuclear outer membrane | 1 |
| cellular component | tRNA-splicing ligase complex | 1 |
| cellular component | HU-DNA complex | 1 |
| cellular component | Isw1b complex | 1 |
| cellular component | Cdc48p-Npl4p-Vms1p AAA ATPase complex | 1 |
| cellular component | C-fiber | 1 |
| cellular component | intraciliary transport particle A | 1 |
| cellular component | extrinsic component of Golgi membrane | 1 |
| cellular component | mitochondrial oxoglutarate dehydrogenase complex | 1 |
| cellular component | presequence translocase-associated import motor | 1 |
| cellular component | nuclear inclusion body | 1 |
| cellular component | mitochondrial fatty acid beta-oxidation multienzyme complex | 1 |
| cellular component | Golgi cisterna | 1 |
| cellular component | gamma-tubulin small complex | 1 |
| cellular component | mRNA cap binding complex | 1 |
| cellular component | bacterial-type flagellum basal body, distal rod | 1 |
| cellular component | nucleolar chromatin | 1 |
| cellular component | ascospore wall | 1 |
| cellular component | SNARE complex | 1 |
| cellular component | endoplasmic reticulum tubular network | 1 |
| cellular component | nuclear SCF ubiquitin ligase complex | 1 |
| cellular component | Ctf18 RFC-like complex | 1 |
| cellular component | vanadium-iron nitrogenase complex | 1 |
| cellular component | U2-type catalytic step 2 spliceosome | 1 |
| cellular component | acrosomal vesicle | 1 |
| cellular component | chloroplastic endopeptidase Clp complex | 1 |
| cellular component | CCR4-NOT core complex | 1 |
| cellular component | synaptic vesicle | 1 |
| cellular component | apical junction complex | 1 |
| cellular component | glutathione synthase complex | 1 |
| cellular component | LINC complex | 1 |
| cellular component | sperm fibrous sheath | 1 |
| cellular component | MHC class I protein complex | 1 |
| cellular component | MPP7-DLG1-LIN7 complex | 1 |
| cellular component | integral component of nuclear inner membrane | 1 |
| cellular component | nuclear origin of replication recognition complex | 1 |
| cellular component | Sin3 complex | 1 |
| cellular component | Golgi medial cisterna | 1 |
| cellular component | clathrin coat of coated pit | 1 |
| cellular component | inner membrane complex | 1 |
| cellular component | nuclear nucleosome | 1 |
| cellular component | smooth muscle contractile fiber | 1 |
| cellular component | extraorganismal space | 1 |
| cellular component | inhibitory synapse | 1 |
| cellular component | Y chromosome | 1 |
| cellular component | condensed chromosome kinetochore | 1 |
| cellular component | mitochondrial small ribosomal subunit | 1 |
| cellular component | male germ cell nucleus | 1 |
| cellular component | cornified envelope | 1 |
| cellular component | iron-iron nitrogenase complex | 1 |
| cellular component | acidocalcisome | 1 |
| cellular component | smooth endoplasmic reticulum | 1 |
| cellular component | U2 snRNP | 1 |
| cellular component | photoreceptor connecting cilium | 1 |
| cellular component | ionotropic glutamate receptor complex | 1 |
| cellular component | IkappaB kinase complex | 1 |
| cellular component | transport vesicle | 1 |
| cellular component | COPI-coated vesicle | 1 |
| cellular component | intraciliary transport particle | 1 |
| cellular component | laminin-5 complex | 1 |
| cellular component | NOS2-CD74 complex | 1 |
| cellular component | side of membrane | 1 |
| cellular component | sulfate adenylyltransferase complex (ATP) | 1 |
| cellular component | actin filament bundle | 1 |
| cellular component | integrin complex | 1 |
| cellular component | Rad51B-Rad51C-Rad51D-XRCC2 complex | 1 |
| cellular component | contractile vacuolar membrane | 1 |
| cellular component | efflux pump complex | 1 |
| cellular component | cAMP-dependent protein kinase complex | 1 |
| cellular component | intercellular bridge | 1 |
| cellular component | Golgi lumen | 1 |
| cellular component | collagen type XI trimer | 1 |
| cellular component | bacterial-type flagellum hook-filament junction | 1 |
| cellular component | dense fibrillar component | 1 |
| cellular component | D-amino-acid dehydrogenase complex | 1 |
| cellular component | chromocenter | 1 |
| cellular component | cortical endoplasmic reticulum | 1 |
| cellular component | hyphal tip | 1 |
| cellular component | DNA-directed RNA polymerase II, core complex | 1 |
| cellular component | chromatin silencing complex | 1 |
| cellular component | protein phosphatase 4 complex | 1 |
| cellular component | muscle tendon junction | 1 |
| cellular component | ATG1/ULK1 kinase complex | 1 |
| cellular component | PHA granule | 1 |
| cellular component | semaphorin receptor complex | 1 |
| cellular component | eukaryotic translation elongation factor 1 complex | 1 |
| cellular component | endoplasmic reticulum exit site | 1 |
| cellular component | DNA helicase A complex | 1 |
| cellular component | MLL1 complex | 1 |
| cellular component | Isw1a complex | 1 |
| cellular component | signal peptidase complex | 1 |
| cellular component | NURS complex | 1 |
| cellular component | eukaryotic 48S preinitiation complex | 1 |
| cellular component | Golgi transport complex | 1 |
| cellular component | CoA-synthesizing protein complex | 1 |
| cellular component | Tic complex | 1 |
| cellular component | anchored component of periplasmic side of cell outer membrane | 1 |
| cellular component | mitochondrial proton-transporting ATP synthase, stator stalk | 1 |
| cellular component | nucleus-vacuole junction | 1 |
| cellular component | cytoskeletal calyx | 1 |
| cellular component | chorion | 1 |
| cellular component | mitochondrial outer membrane translocase complex | 1 |
| cellular component | cytoplasmic chromatin | 1 |
| cellular component | HULC complex | 1 |
| cellular component | meiotic cohesin complex | 1 |
| cellular component | eukaryotic translation initiation factor 3 complex, eIF3m | 1 |
| cellular component | AP-2 adaptor complex | 1 |
| cellular component | cytoplasmic side of endosome membrane | 1 |
| cellular component | Gram-positive-bacterium-type cell wall | 1 |
| cellular component | heterochromatin island | 1 |
| cellular component | symbiont-containing vacuole membrane | 1 |
| cellular component | chloroplast ATP synthase complex | 1 |
| cellular component | chloroplast thylakoid lumen | 1 |
| cellular component | nonmotile primary cilium | 1 |
| cellular component | haptoglobin-hemoglobin complex | 1 |
| cellular component | type III intermediate filament | 1 |
| cellular component | alpha,alpha-trehalose-phosphate synthase complex (UDP-forming) | 1 |
| cellular component | DNA-directed RNA polymerase I complex | 1 |
| cellular component | cytoplasmic dynein complex | 1 |
| cellular component | cellular bud neck septin ring | 1 |
| cellular component | phosphatidylinositol 3-kinase complex, class III, type I | 1 |
| cellular component | TSC1-TSC2 complex | 1 |
| cellular component | integral component of peroxisomal membrane | 1 |
| cellular component | endoplasmic reticulum chaperone complex | 1 |
| cellular component | PTW/PP1 phosphatase complex | 1 |
| cellular component | MutLgamma complex | 1 |
| cellular component | proteasome complex | 1 |
| cellular component | lateral element | 1 |
| cellular component | axolemma | 1 |
| cellular component | potassium ion-transporting ATPase complex | 1 |
| cellular component | primary cilium | 1 |
| cellular component | hyphal septin band | 1 |
| cellular component | nuclear exosome (RNase complex) | 1 |
| cellular component | nuclear body | 1 |
| cellular component | NatC complex | 1 |
| cellular component | viral terminase complex | 1 |
| cellular component | viral nucleocapsid | 1 |
| cellular component | Rad17 RFC-like complex | 1 |
| cellular component | equatorial microtubule organizing center | 1 |
| cellular component | NuRD complex | 1 |
| cellular component | membrane-bounded vesicle | 1 |
| cellular component | polarisome | 1 |
| cellular component | SeqA-DNA complex | 1 |
| cellular component | cytoplasmic exosome (RNase complex) | 1 |
| cellular component | Rad6-Rad18 complex | 1 |
| cellular component | Nrd1 complex | 1 |
| cellular component | organellar large ribosomal subunit | 1 |
| cellular component | nuclear telomere cap complex | 1 |
| cellular component | organellar chromatophore | 1 |
| cellular component | MTREC complex | 1 |
| cellular component | macrophage migration inhibitory factor receptor complex | 1 |
| cellular component | protein storage vacuole | 1 |
| cellular component | PeBoW complex | 1 |
| cellular component | polytene chromosome puff | 1 |
| cellular component | DNA replication termination region | 1 |
| cellular component | inaD signaling complex | 1 |
| cellular component | spliceosomal complex | 1 |
| cellular component | CBF3 complex | 1 |
| cellular component | kinesin II complex | 1 |
| cellular component | extracellular vesicle | 1 |
| cellular component | phosphatidylinositol 3-kinase complex, class III, type II | 1 |
| cellular component | mitochondrial isocitrate dehydrogenase complex (NAD+) | 1 |
| cellular component | 90S preribosome | 1 |
| cellular component | elastic fiber | 1 |
| cellular component | perinuclear endoplasmic reticulum | 1 |
| cellular component | pre-autophagosomal structure membrane | 1 |
| cellular component | ribbon synapse | 1 |
| cellular component | Golgi-associated vesicle | 1 |
| cellular component | immunoglobulin complex, circulating | 1 |
| cellular component | transverse filament | 1 |
| cellular component | collagen and cuticulin-based cuticle extracellular matrix | 1 |
| cellular component | Pwp2p-containing subcomplex of 90S preribosome | 1 |
| cellular component | glycolate oxidase complex | 1 |
| cellular component | tubulin complex | 1 |
| cellular component | polar microtubule | 1 |
| molecular function | molecular function | 14659 |
| molecular function | protein binding | 5722 |
| molecular function | ATP binding | 3983 |
| molecular function | catalytic activity | 3664 |
| molecular function | transcription factor activity, sequence-specific DNA binding | 3407 |
| molecular function | DNA binding | 3325 |
| molecular function | ATPase activity, coupled to transmembrane movement of substances | 2744 |
| molecular function | metal ion binding | 2585 |
| molecular function | structural constituent of ribosome | 2511 |
| molecular function | hydrolase activity | 2438 |
| molecular function | oxidoreductase activity | 2400 |
| molecular function | transferase activity | 2317 |
| molecular function | nucleotide binding | 2123 |
| molecular function | identical protein binding | 1655 |
| molecular function | transporter activity | 1476 |
| molecular function | transferase activity, transferring glycosyl groups | 1340 |
| molecular function | zinc ion binding | 1319 |
| molecular function | ATPase activity | 1295 |
| molecular function | magnesium ion binding | 1206 |
| molecular function | phosphorelay sensor kinase activity | 1202 |
| molecular function | RNA binding | 1201 |
| molecular function | phosphorelay response regulator activity | 1123 |
| molecular function | GTPase activity | 1100 |
| molecular function | peptidase activity | 1044 |
| molecular function | 4 iron, 4 sulfur cluster binding | 946 |
| molecular function | electron carrier activity | 945 |
| molecular function | drug transmembrane transporter activity | 903 |
| molecular function | pyridoxal phosphate binding | 833 |
| molecular function | kinase activity | 831 |
| molecular function | transposase activity | 733 |
| molecular function | DNA-directed DNA polymerase activity | 693 |
| molecular function | sequence-specific DNA binding | 605 |
| molecular function | pseudouridine synthase activity | 585 |
| molecular function | lyase activity | 581 |
| molecular function | metalloendopeptidase activity | 562 |
| molecular function | flavin adenine dinucleotide binding | 561 |
| molecular function | structural molecule activity | 544 |
| molecular function | metallocarboxypeptidase activity | 539 |
| molecular function | serine-type carboxypeptidase activity | 529 |
| molecular function | lipid-transporting ATPase activity | 518 |
| molecular function | phosphatase activity | 517 |
| molecular function | glyoxalase III activity | 509 |
| molecular function | methyltransferase activity | 501 |
| molecular function | GTP binding | 497 |
| molecular function | isomerase activity | 495 |
| molecular function | site-specific recombinase activity | 480 |
| molecular function | manganese ion binding | 476 |
| molecular function | ATP-dependent DNA helicase activity | 472 |
| molecular function | DNA topoisomerase type II (ATP-hydrolyzing) activity | 460 |
| molecular function | iron-sulfur cluster binding | 451 |
| molecular function | core promoter proximal region sequence-specific DNA binding | 448 |
| molecular function | substrate-specific transmembrane transporter activity | 447 |
| molecular function | single-stranded DNA binding | 444 |
| molecular function | DNA-directed RNA polymerase activity | 437 |
| molecular function | ATP-dependent RNA helicase activity | 433 |
| molecular function | sigma factor activity | 431 |
| molecular function | transmembrane transporter activity | 431 |
| molecular function | nucleic acid binding | 424 |
| molecular function | proton-transporting ATP synthase activity, rotational mechanism | 423 |
| molecular function | recombinase activity | 420 |
| molecular function | deaminase activity | 407 |
| molecular function | iron ion binding | 400 |
| molecular function | translation elongation factor activity | 398 |
| molecular function | NAD binding | 392 |
| molecular function | metallopeptidase activity | 384 |
| molecular function | helicase activity | 378 |
| molecular function | ligase activity | 374 |
| molecular function | hydrolase activity, hydrolyzing O-glycosyl compounds | 369 |
| molecular function | serine-type endopeptidase activity | 366 |
| molecular function | antiporter activity | 361 |
| molecular function | nucleotidyltransferase activity | 360 |
| molecular function | monosaccharide transmembrane transporter activity | 356 |
| molecular function | excinuclease ABC activity | 355 |
| molecular function | rRNA binding | 351 |
| molecular function | ribonucleoside-diphosphate reductase activity, thioredoxin disulfide as acceptor | 351 |
| molecular function | ribosome binding | 346 |
| molecular function | double-stranded DNA binding | 339 |
| molecular function | peptide transporter activity | 338 |
| molecular function | S-adenosylmethionine-dependent methyltransferase activity | 331 |
| molecular function | exonuclease activity | 322 |
| molecular function | symporter activity | 319 |
| molecular function | signal transducer activity | 318 |
| molecular function | phosphotransferase activity, alcohol group as acceptor | 315 |
| molecular function | protein transporter activity | 304 |
| molecular function | protein homodimerization activity | 304 |
| molecular function | tRNA binding | 303 |
| molecular function | FMN binding | 302 |
| molecular function | hydrolase activity, acting on glycosyl bonds | 299 |
| molecular function | penicillin binding | 296 |
| molecular function | peptidyl-prolyl cis-trans isomerase activity | 295 |
| molecular function | L-amino acid transmembrane transporter activity | 291 |
| molecular function | oligopeptide-transporting ATPase activity | 290 |
| molecular function | amino acid transmembrane transporter activity | 290 |
| molecular function | DNA topoisomerase type I activity | 289 |
| molecular function | phosphate ion transmembrane-transporting ATPase activity | 280 |
| molecular function | ATP-dependent peptidase activity | 276 |
| molecular function | nuclease activity | 267 |
| molecular function | peptide alpha-N-acetyltransferase activity | 262 |
| molecular function | protein-N(PI)-phosphohistidine-sugar phosphotransferase activity | 258 |
| molecular function | endonuclease activity | 257 |
| molecular function | carbohydrate binding | 253 |
| molecular function | cation-transporting ATPase activity | 252 |
| molecular function | serine-type D-Ala-D-Ala carboxypeptidase activity | 248 |
| molecular function | efflux transmembrane transporter activity | 246 |
| molecular function | N-acetylmuramoyl-L-alanine amidase activity | 238 |
| molecular function | NADP binding | 237 |
| molecular function | mRNA binding | 236 |
| molecular function | protein histidine kinase activity | 231 |
| molecular function | NADH dehydrogenase activity | 226 |
| molecular function | transaminase activity | 226 |
| molecular function | transferase activity, transferring acyl groups | 225 |
| molecular function | four-way junction helicase activity | 221 |
| molecular function | P-P-bond-hydrolysis-driven protein transmembrane transporter activity | 218 |
| molecular function | endopeptidase activity | 216 |
| molecular function | inorganic phosphate transmembrane transporter activity | 213 |
| molecular function | beta-glucosidase activity | 213 |
| molecular function | unfolded protein binding | 211 |
| molecular function | polyamine-transporting ATPase activity | 210 |
| molecular function | zinc ion transmembrane transporter activity | 210 |
| molecular function | sugar:proton symporter activity | 210 |
| molecular function | 2 iron, 2 sulfur cluster binding | 208 |
| molecular function | amino acid binding | 208 |
| molecular function | ferrous iron binding | 205 |
| molecular function | phenylalanine-tRNA ligase activity | 202 |
| molecular function | acetyl-CoA carboxylase activity | 200 |
| molecular function | site-specific DNA-methyltransferase (adenine-specific) activity | 200 |
| molecular function | aldehyde dehydrogenase (NAD) activity | 199 |
| molecular function | ATP-dependent 3'-5' DNA helicase activity | 195 |
| molecular function | protein disulfide oxidoreductase activity | 195 |
| molecular function | copper-transporting ATPase activity | 193 |
| molecular function | potassium ion transmembrane transporter activity | 192 |
| molecular function | DNA-methyltransferase activity | 190 |
| molecular function | amino acid-transporting ATPase activity | 189 |
| molecular function | calcium ion binding | 189 |
| molecular function | GTP diphosphokinase activity | 189 |
| molecular function | DNA helicase activity | 187 |
| molecular function | iron ion transmembrane transporter activity | 185 |
| molecular function | N-acetyltransferase activity | 183 |
| molecular function | aminopeptidase activity | 183 |
| molecular function | ribonucleoside-triphosphate reductase activity | 178 |
| molecular function | transferase activity, transferring phosphorus-containing groups | 178 |
| molecular function | carbamoyl-phosphate synthase (glutamine-hydrolyzing) activity | 177 |
| molecular function | transferase activity, transferring hexosyl groups | 171 |
| molecular function | guanosine-3',5'-bis(diphosphate) 3'-diphosphatase activity | 171 |
| molecular function | transketolase activity | 167 |
| molecular function | uridine kinase activity | 167 |
| molecular function | acid-amino acid ligase activity | 166 |
| molecular function | hydro-lyase activity | 165 |
| molecular function | translation release factor activity, codon specific | 163 |
| molecular function | sodium-dependent phosphate transmembrane transporter activity | 163 |
| molecular function | DNA (cytosine-5-)-methyltransferase activity | 162 |
| molecular function | sodium:phosphate symporter activity | 162 |
| molecular function | acyl-CoA dehydrogenase activity | 162 |
| molecular function | 5'-3' exonuclease activity | 161 |
| molecular function | ferrous iron uptake transmembrane transporter activity | 155 |
| molecular function | phosphoribosylaminoimidazole carboxylase activity | 155 |
| molecular function | DNA-(apurinic or apyrimidinic site) lyase activity | 154 |
| molecular function | acetyltransferase activity | 154 |
| molecular function | endoribonuclease activity | 153 |
| molecular function | NADH dehydrogenase (ubiquinone) activity | 152 |
| molecular function | protein-phosphocysteine-sugar phosphotransferase activity | 152 |
| molecular function | transcription regulatory region DNA binding | 151 |
| molecular function | RNA-DNA hybrid ribonuclease activity | 150 |
| molecular function | L-serine ammonia-lyase activity | 150 |
| molecular function | potassium ion binding | 150 |
| molecular function | DNA-dependent ATPase activity | 149 |
| molecular function | deacetylase activity | 148 |
| molecular function | 3-oxoacyl-[acyl-carrier-protein] reductase (NADPH) activity | 148 |
| molecular function | isoleucine-tRNA ligase activity | 147 |
| molecular function | fatty-acyl-CoA binding | 147 |
| molecular function | potassium-transporting ATPase activity | 147 |
| molecular function | translation initiation factor activity | 147 |
| molecular function | heme binding | 146 |
| molecular function | prophage integrase activity | 146 |
| molecular function | carboxy-lyase activity | 145 |
| molecular function | oxidoreductase activity, acting on the CH-CH group of donors, with a flavin as acceptor | 143 |
| molecular function | DNA ligase (NAD+) activity | 142 |
| molecular function | damaged DNA binding | 141 |
| molecular function | alanine-tRNA ligase activity | 141 |
| molecular function | methionine synthase activity | 139 |
| molecular function | siderophore uptake transmembrane transporter activity | 138 |
| molecular function | oxidoreductase activity, acting on a sulfur group of donors, disulfide as acceptor | 138 |
| molecular function | glutamine-fructose-6-phosphate transaminase (isomerizing) activity | 137 |
| molecular function | methenyltetrahydrofolate cyclohydrolase activity | 136 |
| molecular function | tRNA methyltransferase activity | 136 |
| molecular function | proton-transporting ATPase activity, rotational mechanism | 135 |
| molecular function | Type I site-specific deoxyribonuclease activity | 135 |
| molecular function | sodium:proton antiporter activity | 134 |
| molecular function | 3'-5'-exoribonuclease activity | 132 |
| molecular function | valine-tRNA ligase activity | 132 |
| molecular function | fructose-bisphosphate aldolase activity | 132 |
| molecular function | leucine-tRNA ligase activity | 131 |
| molecular function | cardiolipin synthase activity | 131 |
| molecular function | pyruvate kinase activity | 130 |
| molecular function | sulfuric ester hydrolase activity | 129 |
| molecular function | DNA clamp loader activity | 129 |
| molecular function | nickel cation binding | 128 |
| molecular function | endodeoxyribonuclease activity | 127 |
| molecular function | 3-chloroallyl aldehyde dehydrogenase activity | 127 |
| molecular function | 3-oxoacyl-[acyl-carrier-protein] synthase activity | 126 |
| molecular function | C4-dicarboxylate transmembrane transporter activity | 126 |
| molecular function | mechanically-gated ion channel activity | 126 |
| molecular function | hydrolase activity, hydrolyzing N-glycosyl compounds | 125 |
| molecular function | integrase activity | 125 |
| molecular function | DNA primase activity | 125 |
| molecular function | phosphoribosylformylglycinamidine synthase activity | 124 |
| molecular function | rRNA methyltransferase activity | 123 |
| molecular function | cobalt ion binding | 123 |
| molecular function | phosphoribosylaminoimidazolesuccinocarboxamide synthase activity | 122 |
| molecular function | receptor activity | 122 |
| molecular function | ribosomal large subunit binding | 120 |
| molecular function | serine-type peptidase activity | 120 |
| molecular function | actin binding | 119 |
| molecular function | peroxidase activity | 117 |
| molecular function | copper ion binding | 116 |
| molecular function | transferase activity, transferring acyl groups other than amino-acyl groups | 116 |
| molecular function | glycogen phosphorylase activity | 116 |
| molecular function | protein dimerization activity | 116 |
| molecular function | oxidoreductase activity, acting on CH-OH group of donors | 116 |
| molecular function | thioredoxin-disulfide reductase activity | 116 |
| molecular function | oxaloacetate decarboxylase activity | 115 |
| molecular function | aldehyde-lyase activity | 115 |
| molecular function | methylthiotransferase activity | 115 |
| molecular function | nucleoside transmembrane transporter activity | 115 |
| molecular function | GMP synthase (glutamine-hydrolyzing) activity | 114 |
| molecular function | UDP-glucose 6-dehydrogenase activity | 113 |
| molecular function | DNA topoisomerase activity | 113 |
| molecular function | methionine adenosyltransferase activity | 113 |
| molecular function | aspartate kinase activity | 112 |
| molecular function | oxidoreductase activity, acting on the CH-OH group of donors, NAD or NADP as acceptor | 112 |
| molecular function | cadmium ion transmembrane transporter activity | 112 |
| molecular function | ribokinase activity | 111 |
| molecular function | protein serine/threonine kinase activity | 111 |
| molecular function | L-aspartate:2-oxoglutarate aminotransferase activity | 111 |
| molecular function | UDP-N-acetylmuramate-L-alanine ligase activity | 110 |
| molecular function | GMP synthase activity | 110 |
| molecular function | methionine-tRNA ligase activity | 109 |
| molecular function | hydrolase activity, acting on carbon-nitrogen (but not peptide) bonds | 109 |
| molecular function | aspartate-tRNA ligase activity | 109 |
| molecular function | 2-C-methyl-D-erythritol 2,4-cyclodiphosphate synthase activity | 109 |
| molecular function | succinate dehydrogenase activity | 108 |
| molecular function | uridylate kinase activity | 108 |
| molecular function | polyribonucleotide nucleotidyltransferase activity | 105 |
| molecular function | FAD binding | 105 |
| molecular function | L-leucine transmembrane transporter activity | 105 |
| molecular function | D-ribose-importing ATPase activity | 105 |
| molecular function | UDP-glucose 4-epimerase activity | 105 |
| molecular function | cobalt ion transmembrane transporter activity | 104 |
| molecular function | small ribosomal subunit rRNA binding | 104 |
| molecular function | aminoacyl-tRNA ligase activity | 104 |
| molecular function | racemase and epimerase activity, acting on carbohydrates and derivatives | 103 |
| molecular function | phosphotransferase activity, for other substituted phosphate groups | 103 |
| molecular function | alcohol dehydrogenase activity, iron-dependent | 103 |
| molecular function | exodeoxyribonuclease VII activity | 102 |
| molecular function | 3-dehydroquinate synthase activity | 102 |
| molecular function | threonine-tRNA ligase activity | 102 |
| molecular function | glucokinase activity | 101 |
| molecular function | cation transmembrane transporter activity | 101 |
| molecular function | formate C-acetyltransferase activity | 101 |
| molecular function | fumarate hydratase activity | 101 |
| molecular function | NADPH dehydrogenase activity | 101 |
| molecular function | glycine reductase activity | 100 |
| molecular function | tRNA dihydrouridine synthase activity | 100 |
| molecular function | monooxygenase activity | 100 |
| molecular function | ribonuclease activity | 100 |
| molecular function | deoxyribonuclease activity | 100 |
| molecular function | dUTP diphosphatase activity | 99 |
| molecular function | cysteine-tRNA ligase activity | 99 |
| molecular function | formate-tetrahydrofolate ligase activity | 99 |
| molecular function | xanthine dehydrogenase activity | 99 |
| molecular function | arginine-tRNA ligase activity | 98 |
| molecular function | RNA-directed DNA polymerase activity | 98 |
| molecular function | peptide deformylase activity | 97 |
| molecular function | alcohol dehydrogenase (NAD) activity | 97 |
| molecular function | small molecule binding | 97 |
| molecular function | tyrosine-tRNA ligase activity | 96 |
| molecular function | branched-chain amino acid transmembrane transporter activity | 96 |
| molecular function | lysozyme activity | 96 |
| molecular function | protein binding involved in protein folding | 95 |
| molecular function | single-stranded DNA exodeoxyribonuclease activity | 95 |
| molecular function | L-valine transmembrane transporter activity | 95 |
| molecular function | metal ion transmembrane transporter activity | 95 |
| molecular function | protein kinase activity | 94 |
| molecular function | metalloexopeptidase activity | 94 |
| molecular function | L-isoleucine transmembrane transporter activity | 93 |
| molecular function | sodium ion transmembrane transporter activity | 93 |
| molecular function | fimbrial usher porin activity | 93 |
| molecular function | glycerol kinase activity | 93 |
| molecular function | ribose-5-phosphate isomerase activity | 93 |
| molecular function | coproporphyrinogen oxidase activity | 92 |
| molecular function | molybdenum ion binding | 92 |
| molecular function | oxidoreductase activity, acting on the aldehyde or oxo group of donors | 92 |
| molecular function | glucose-1-phosphate adenylyltransferase activity | 92 |
| molecular function | glutamate-tRNA ligase activity | 91 |
| molecular function | queuine tRNA-ribosyltransferase activity | 90 |
| molecular function | alpha-amylase activity | 90 |
| molecular function | four-way junction DNA binding | 90 |
| molecular function | metalloaminopeptidase activity | 90 |
| molecular function | proline-tRNA ligase activity | 90 |
| molecular function | histidine-tRNA ligase activity | 90 |
| molecular function | [formate-C-acetyltransferase]-activating enzyme activity | 90 |
| molecular function | porin activity | 90 |
| molecular function | 3-deoxy-7-phosphoheptulonate synthase activity | 90 |
| molecular function | adenylate kinase activity | 90 |
| molecular function | carbamoyl-phosphate synthase (ammonia) activity | 89 |
| molecular function | purine-nucleoside phosphorylase activity | 89 |
| molecular function | rRNA (guanosine-2'-O-)-methyltransferase activity | 89 |
| molecular function | cystathionine gamma-lyase activity | 89 |
| molecular function | 1,4-alpha-glucan branching enzyme activity | 88 |
| molecular function | poly(A) RNA binding | 88 |
| molecular function | hydrolase activity, acting on ester bonds | 87 |
| molecular function | oligopeptide transporter activity | 87 |
| molecular function | aminoacyl-tRNA hydrolase activity | 87 |
| molecular function | intramolecular oxidoreductase activity, interconverting aldoses and ketoses | 87 |
| molecular function | amidase activity | 87 |
| molecular function | ACP phosphopantetheine attachment site binding involved in fatty acid biosynthetic process | 87 |
| molecular function | sulfate transmembrane transporter activity | 86 |
| molecular function | hydrolase activity, acting on acid anhydrides, catalyzing transmembrane movement of substances | 86 |
| molecular function | structural constituent of cell wall | 85 |
| molecular function | polyphosphate kinase activity | 85 |
| molecular function | uracil DNA N-glycosylase activity | 85 |
| molecular function | ribose phosphate diphosphokinase activity | 85 |
| molecular function | glutamate-ammonia ligase activity | 85 |
| molecular function | dihydroorotate dehydrogenase activity | 85 |
| molecular function | peptide binding | 84 |
| molecular function | tryptophan-tRNA ligase activity | 84 |
| molecular function | N-acetylglucosamine-6-phosphate deacetylase activity | 84 |
| molecular function | phosphoglucosamine mutase activity | 84 |
| molecular function | restriction endodeoxyribonuclease activity | 84 |
| molecular function | ligase activity, forming carbon-nitrogen bonds | 84 |
| molecular function | D-alanine-D-alanine ligase activity | 84 |
| molecular function | glycine-tRNA ligase activity | 83 |
| molecular function | 3'-5' exonuclease activity | 83 |
| molecular function | protein-N(PI)-phosphohistidine-mannose phosphotransferase system transporter activity | 83 |
| molecular function | DNA N-glycosylase activity | 83 |
| molecular function | UTP:glucose-1-phosphate uridylyltransferase activity | 82 |
| molecular function | UDP-N-acetylglucosamine 2-epimerase activity | 82 |
| molecular function | ribonuclease III activity | 82 |
| molecular function | alanine racemase activity | 82 |
| molecular function | aminoacyl-tRNA editing activity | 81 |
| molecular function | rRNA (adenine-N6,N6-)-dimethyltransferase activity | 81 |
| molecular function | antibiotic transporter activity | 81 |
| molecular function | NADH peroxidase activity | 81 |
| molecular function | IMP dehydrogenase activity | 80 |
| molecular function | acetolactate synthase activity | 80 |
| molecular function | extracellular matrix structural constituent | 80 |
| molecular function | lipid-linked peptidoglycan transporter activity | 80 |
| molecular function | phosphoglycerate kinase activity | 80 |
| molecular function | nucleoside-triphosphate diphosphatase activity | 80 |
| molecular function | alpha-L-fucosidase activity | 79 |
| molecular function | chaperone binding | 79 |
| molecular function | N6-(1,2-dicarboxyethyl)AMP AMP-lyase (fumarate-forming) activity | 79 |
| molecular function | dipeptidase activity | 78 |
| molecular function | glyceraldehyde-3-phosphate dehydrogenase (NAD+) (phosphorylating) activity | 78 |
| molecular function | aspartate carbamoyltransferase activity | 78 |
| molecular function | acylglycerol O-acyltransferase activity | 78 |
| molecular function | lysophosphatidic acid acyltransferase activity | 78 |
| molecular function | methionyl-tRNA formyltransferase activity | 78 |
| molecular function | phosphoribosylamine-glycine ligase activity | 78 |
| molecular function | methylenetetrahydrofolate dehydrogenase (NADP+) activity | 78 |
| molecular function | serine-tRNA ligase activity | 78 |
| molecular function | UDP-N-acetylmuramate dehydrogenase activity | 78 |
| molecular function | phospho-N-acetylmuramoyl-pentapeptide-transferase activity | 78 |
| molecular function | dolichyl-phosphate-mannose-protein mannosyltransferase activity | 78 |
| molecular function | long-chain fatty acid-CoA ligase activity | 78 |
| molecular function | inorganic diphosphatase activity | 77 |
| molecular function | phosphopantothenoylcysteine decarboxylase activity | 77 |
| molecular function | S-adenosylmethionine:tRNA ribosyltransferase-isomerase activity | 77 |
| molecular function | 5S rRNA binding | 77 |
| molecular function | motor activity | 77 |
| molecular function | sodium:dicarboxylate symporter activity | 77 |
| molecular function | glucose-1-phosphate thymidylyltransferase activity | 77 |
| molecular function | 6-phosphofructokinase activity | 76 |
| molecular function | phosphoric diester hydrolase activity | 76 |
| molecular function | iron-sulfur transferase activity | 76 |
| molecular function | 2-C-methyl-D-erythritol 4-phosphate cytidylyltransferase activity | 76 |
| molecular function | solute:proton symporter activity | 75 |
| molecular function | 1-deoxy-D-xylulose-5-phosphate synthase activity | 75 |
| molecular function | dGTPase activity | 75 |
| molecular function | NADPH binding | 75 |
| molecular function | glucose-6-phosphate isomerase activity | 75 |
| molecular function | O-acetyltransferase activity | 75 |
| molecular function | dolichyl-phosphate beta-D-mannosyltransferase activity | 75 |
| molecular function | cyclic pyranopterin monophosphate synthase activity | 75 |
| molecular function | solute:sodium symporter activity | 74 |
| molecular function | nicotinate phosphoribosyltransferase activity | 74 |
| molecular function | enoyl-[acyl-carrier-protein] reductase (NADH) activity | 74 |
| molecular function | transferase activity, transferring alkyl or aryl (other than methyl) groups | 74 |
| molecular function | phosphoribosylaminoimidazolecarboxamide formyltransferase activity | 74 |
| molecular function | UDP-N-acetylmuramoylalanine-D-glutamate ligase activity | 73 |
| molecular function | ribulose-phosphate 3-epimerase activity | 73 |
| molecular function | phosphate ion binding | 73 |
| molecular function | L-lactate dehydrogenase activity | 73 |
| molecular function | oxidoreductase activity, acting on diphenols and related substances as donors, oxygen as acceptor | 73 |
| molecular function | adenylosuccinate synthase activity | 73 |
| molecular function | enzyme activator activity | 72 |
| molecular function | riboflavin kinase activity | 72 |
| molecular function | rRNA (cytosine-N4-)-methyltransferase activity | 72 |
| molecular function | triose-phosphate isomerase activity | 72 |
| molecular function | guanylate kinase activity | 72 |
| molecular function | acetate kinase activity | 72 |
| molecular function | cysteine synthase activity | 72 |
| molecular function | (S)-2-(5-amino-1-(5-phospho-D-ribosyl)imidazole-4-carboxamido)succinate AMP-lyase (fumarate-forming) activity | 72 |
| molecular function | glucosamine-6-phosphate deaminase activity | 71 |
| molecular function | AMP binding | 71 |
| molecular function | NAD+ synthase activity | 71 |
| molecular function | thiamine-phosphate diphosphorylase activity | 71 |
| molecular function | 3-phosphoshikimate 1-carboxyvinyltransferase activity | 71 |
| molecular function | histone acetyltransferase activity | 71 |
| molecular function | uroporphyrin-III C-methyltransferase activity | 70 |
| molecular function | oxidoreductase activity, acting on the aldehyde or oxo group of donors, NAD or NADP as acceptor | 70 |
| molecular function | serine O-acetyltransferase activity | 70 |
| molecular function | dihydroorotase activity | 70 |
| molecular function | carboxylic ester hydrolase activity | 70 |
| molecular function | lysine-tRNA ligase activity | 69 |
| molecular function | double-stranded DNA 3'-5' exodeoxyribonuclease activity | 69 |
| molecular function | pyrroline-5-carboxylate reductase activity | 69 |
| molecular function | transcription factor binding | 69 |
| molecular function | peptide-transporting ATPase activity | 69 |
| molecular function | amidophosphoribosyltransferase activity | 69 |
| molecular function | monosaccharide-transporting ATPase activity | 69 |
| molecular function | double-stranded RNA binding | 69 |
| molecular function | phosphoserine phosphatase activity | 68 |
| molecular function | monovalent inorganic cation transmembrane transporter activity | 68 |
| molecular function | glycerol-3-phosphate transmembrane transporter activity | 68 |
| molecular function | tripeptide aminopeptidase activity | 68 |
| molecular function | phosphoprotein phosphatase activity | 68 |
| molecular function | IMP cyclohydrolase activity | 67 |
| molecular function | L-cysteine desulfhydrase activity | 67 |
| molecular function | lysine decarboxylase activity | 67 |
| molecular function | tRNA (guanine-N7-)-methyltransferase activity | 67 |
| molecular function | carboxypeptidase activity | 67 |
| molecular function | diacylglycerol kinase activity | 67 |
| molecular function | 4-(cytidine 5'-diphospho)-2-C-methyl-D-erythritol kinase activity | 67 |
| molecular function | dTDP-glucose 4,6-dehydratase activity | 67 |
| molecular function | phosphatidate cytidylyltransferase activity | 67 |
| molecular function | tRNA dimethylallyltransferase activity | 67 |
| molecular function | RNA methyltransferase activity | 67 |
| molecular function | adenosylhomocysteine nucleosidase activity | 67 |
| molecular function | microtubule motor activity | 67 |
| molecular function | 4-hydroxy-3-methylbut-2-en-1-yl diphosphate synthase activity | 67 |
| molecular function | DNA translocase activity | 67 |
| molecular function | disulfide oxidoreductase activity | 66 |
| molecular function | ADP-heptose-lipopolysaccharide heptosyltransferase activity | 66 |
| molecular function | shikimate kinase activity | 66 |
| molecular function | dephospho-CoA kinase activity | 66 |
| molecular function | glutaminyl-tRNA synthase (glutamine-hydrolyzing) activity | 66 |
| molecular function | glycine hydroxymethyltransferase activity | 66 |
| molecular function | tRNA threonylcarbamoyladenosine dehydratase | 66 |
| molecular function | 1-deoxy-D-xylulose-5-phosphate reductoisomerase activity | 66 |
| molecular function | large ribosomal subunit rRNA binding | 65 |
| molecular function | chorismate synthase activity | 65 |
| molecular function | 4-hydroxy-tetrahydrodipicolinate synthase | 65 |
| molecular function | phosphoribosylformylglycinamidine cyclo-ligase activity | 64 |
| molecular function | asparaginase activity | 64 |
| molecular function | phosphomethylpyrimidine kinase activity | 64 |
| molecular function | oxidoreductase activity, acting on NAD(P)H | 64 |
| molecular function | lytic transglycosylase activity | 63 |
| molecular function | adenosine deaminase activity | 63 |
| molecular function | NAD(P)+ transhydrogenase (B-specific) activity | 63 |
| molecular function | cytidylate kinase activity | 63 |
| molecular function | ornithine carbamoyltransferase activity | 63 |
| molecular function | aspartic acid methylthiotransferase activity | 63 |
| molecular function | tetrahydrofolylpolyglutamate synthase activity | 63 |
| molecular function | aspartate-semialdehyde dehydrogenase activity | 63 |
| molecular function | glycerophosphodiester phosphodiesterase activity | 62 |
| molecular function | nickel cation transmembrane transporter activity | 62 |
| molecular function | chitinase activity | 62 |
| molecular function | glycerophosphodiester transmembrane transporter activity | 62 |
| molecular function | diaminopimelate decarboxylase activity | 62 |
| molecular function | translation release factor activity, codon nonspecific | 61 |
| molecular function | UDP-N-acetylglucosamine diphosphorylase activity | 61 |
| molecular function | NAD+ binding | 60 |
| molecular function | beta-galactosidase activity | 60 |
| molecular function | shikimate 3-dehydrogenase (NADP+) activity | 60 |
| molecular function | serine-type endopeptidase inhibitor activity | 60 |
| molecular function | lipase activity | 60 |
| molecular function | microtubule binding | 60 |
| molecular function | aspartate-ammonia ligase activity | 60 |
| molecular function | rRNA (uridine-C5-)-methyltransferase activity | 60 |
| molecular function | sulfite reductase activity | 59 |
| molecular function | dihydrolipoyl dehydrogenase activity | 59 |
| molecular function | enzyme regulator activity | 59 |
| molecular function | enzyme inhibitor activity | 59 |
| molecular function | sodium ion binding | 58 |
| molecular function | cation binding | 58 |
| molecular function | phosphoribosylglycinamide formyltransferase activity | 58 |
| molecular function | nicotinamide-nucleotide adenylyltransferase activity | 58 |
| molecular function | [acyl-carrier-protein] S-malonyltransferase activity | 58 |
| molecular function | starch synthase activity | 58 |
| molecular function | ATP-dependent helicase activity | 58 |
| molecular function | phosphoglycerate dehydrogenase activity | 58 |
| molecular function | aconitate hydratase activity | 58 |
| molecular function | pantetheine-phosphate adenylyltransferase activity | 58 |
| molecular function | oxidoreductase activity, acting on NAD(P)H, nitrogenous group as acceptor | 58 |
| molecular function | peptide-methionine (S)-S-oxide reductase activity | 57 |
| molecular function | homoserine dehydrogenase activity | 57 |
| molecular function | 5-formyltetrahydrofolate cyclo-ligase activity | 57 |
| molecular function | carbon-oxygen lyase activity | 57 |
| molecular function | dihydrofolate reductase activity | 57 |
| molecular function | glutamate racemase activity | 57 |
| molecular function | asparagine-tRNA ligase activity | 57 |
| molecular function | acetyl-CoA C-acetyltransferase activity | 57 |
| molecular function | purine nucleosidase activity | 57 |
| molecular function | NAD+ kinase activity | 57 |
| molecular function | FK506 binding | 56 |
| molecular function | phosphoric ester hydrolase activity | 56 |
| molecular function | propionyl-CoA carboxylase activity | 56 |
| molecular function | undecaprenol kinase activity | 56 |
| molecular function | sulfurtransferase activity | 56 |
| molecular function | orotidine-5'-phosphate decarboxylase activity | 56 |
| molecular function | siderophore transmembrane transporter activity | 56 |
| molecular function | riboflavin transporter activity | 56 |
| molecular function | nicotinate-nucleotide adenylyltransferase activity | 55 |
| molecular function | sulfite reductase (ferredoxin) activity | 55 |
| molecular function | phosphopentomutase activity | 55 |
| molecular function | beta-N-acetylhexosaminidase activity | 55 |
| molecular function | signal recognition particle binding | 55 |
| molecular function | coenzyme binding | 55 |
| molecular function | asparagine synthase (glutamine-hydrolyzing) activity | 55 |
| molecular function | deoxyribose-phosphate aldolase activity | 55 |
| molecular function | dTDP-4-dehydrorhamnose reductase activity | 55 |
| molecular function | ADP binding | 55 |
| molecular function | 3-dehydroquinate dehydratase activity | 54 |
| molecular function | N6-isopentenyladenosine methylthiotransferase activity | 54 |
| molecular function | phosphatidylglycerol-prolipoprotein diacylglyceryl transferase activity | 54 |
| molecular function | argininosuccinate synthase activity | 54 |
| molecular function | uracil:cation symporter activity | 54 |
| molecular function | carbamate kinase activity | 53 |
| molecular function | galactose transmembrane transporter activity | 53 |
| molecular function | thymidylate kinase activity | 53 |
| molecular function | mismatched DNA binding | 53 |
| molecular function | glutamine-tRNA ligase activity | 53 |
| molecular function | calcium channel activity | 53 |
| molecular function | D-tyrosyl-tRNA(Tyr) deacylase activity | 52 |
| molecular function | solute:proton antiporter activity | 52 |
| molecular function | cob(I)yrinic acid a,c-diamide adenosyltransferase activity | 52 |
| molecular function | histidinol-phosphate transaminase activity | 52 |
| molecular function | L-glutamine transmembrane transporter activity | 52 |
| molecular function | 3'-tRNA processing endoribonuclease activity | 51 |
| molecular function | alcohol dehydrogenase activity, zinc-dependent | 51 |
| molecular function | crossover junction endodeoxyribonuclease activity | 51 |
| molecular function | chromate transmembrane transporter activity | 50 |
| molecular function | phosphotransferase activity, phosphate group as acceptor | 50 |
| molecular function | mRNA 5'-UTR binding | 50 |
| molecular function | hydrolase activity, acting on carbon-nitrogen (but not peptide) bonds, in linear amides | 50 |
| molecular function | dihydropteroate synthase activity | 50 |
| molecular function | calcium, potassium:sodium antiporter activity | 50 |
| molecular function | malate dehydrogenase activity | 50 |
| molecular function | argininosuccinate lyase activity | 50 |
| molecular function | S-adenosylmethionine-homocysteine S-methyltransferase activity | 50 |
| molecular function | N6-threonylcarbomyladenosine methylthiotransferase activity | 49 |
| molecular function | molybdopterin cofactor binding | 49 |
| molecular function | branched-chain-amino-acid transaminase activity | 49 |
| molecular function | FMN adenylyltransferase activity | 49 |
| molecular function | 5'-nucleotidase activity | 49 |
| molecular function | glutamate:sodium symporter activity | 49 |
| molecular function | aldose 1-epimerase activity | 48 |
| molecular function | bacterial-type RNA polymerase core promoter proximal region sequence-specific DNA binding | 48 |
| molecular function | phosphoglycerate mutase activity | 48 |
| molecular function | pyruvate, phosphate dikinase activity | 48 |
| molecular function | hydroxylamine reductase activity | 48 |
| molecular function | geranyltranstransferase activity | 48 |
| molecular function | threonine synthase activity | 48 |
| molecular function | phosphopyruvate hydratase activity | 48 |
| molecular function | protein tyrosine phosphatase activity | 47 |
| molecular function | lactaldehyde reductase activity | 47 |
| molecular function | GTP cyclohydrolase I activity | 47 |
| molecular function | alkyl hydroperoxide reductase activity | 47 |
| molecular function | formate dehydrogenase (NAD+) activity | 47 |
| molecular function | orotate phosphoribosyltransferase activity | 47 |
| molecular function | alpha-galactosidase activity | 47 |
| molecular function | glutamate-5-semialdehyde dehydrogenase activity | 47 |
| molecular function | putrescine-importing ATPase activity | 47 |
| molecular function | phosphorylase activity | 46 |
| molecular function | UDP-N-acetylglucosamine 1-carboxyvinyltransferase activity | 46 |
| molecular function | p-aminobenzoyl-glutamate uptake transmembrane transporter activity | 46 |
| molecular function | carbohydrate-transporting ATPase activity | 46 |
| molecular function | anthranilate synthase activity | 46 |
| molecular function | oxidoreductase activity, acting on the CH-CH group of donors | 46 |
| molecular function | superoxide dismutase activity | 46 |
| molecular function | glutamate dehydrogenase (NADP+) activity | 46 |
| molecular function | UDP-galactopyranose mutase activity | 46 |
| molecular function | succinate-semialdehyde dehydrogenase [NAD(P)+] activity | 46 |
| molecular function | chorismate mutase activity | 46 |
| molecular function | alpha-1,4-glucosidase activity | 45 |
| molecular function | methylated-DNA-[protein]-cysteine S-methyltransferase activity | 45 |
| molecular function | transcription coactivator activity | 45 |
| molecular function | protein heterodimerization activity | 45 |
| molecular function | ion channel activity | 45 |
| molecular function | transition metal ion binding | 45 |
| molecular function | phosphogluconate dehydrogenase (decarboxylating) activity | 45 |
| molecular function | succinate-semialdehyde dehydrogenase (NAD+) activity | 45 |
| molecular function | ATPase activity, coupled | 45 |
| molecular function | hydrogen ion transmembrane transporter activity | 45 |
| molecular function | cytidine deaminase activity | 44 |
| molecular function | phospholipid binding | 44 |
| molecular function | L-aspartate transmembrane transporter activity | 44 |
| molecular function | dipeptidyl-peptidase activity | 44 |
| molecular function | ubiquitin-protein transferase activity | 44 |
| molecular function | nicotinate-nucleotide diphosphorylase (carboxylating) activity | 44 |
| molecular function | nicotinate-nucleotide-dimethylbenzimidazole phosphoribosyltransferase activity | 43 |
| molecular function | thymidylate synthase activity | 43 |
| molecular function | competence pheromone activity | 43 |
| molecular function | GDP binding | 43 |
| molecular function | adenosylmethionine-8-amino-7-oxononanoate transaminase activity | 43 |
| molecular function | CTP reductase activity | 43 |
| molecular function | polynucleotide adenylyltransferase activity | 43 |
| molecular function | magnesium ion transmembrane transporter activity | 43 |
| molecular function | dTDP-4-dehydrorhamnose 3,5-epimerase activity | 43 |
| molecular function | oxidoreductase activity, acting on NAD(P)H, quinone or similar compound as acceptor | 42 |
| molecular function | nitrite uptake transmembrane transporter activity | 42 |
| molecular function | pyruvate decarboxylase activity | 42 |
| molecular function | adenine deaminase activity | 42 |
| molecular function | ribonuclease P activity | 42 |
| molecular function | beta-N-acetylglucosaminidase activity | 42 |
| molecular function | adenyl-nucleotide exchange factor activity | 42 |
| molecular function | chromatin binding | 42 |
| molecular function | hydrolase activity, acting on acid anhydrides, in phosphorus-containing anhydrides | 42 |
| molecular function | isocitrate dehydrogenase (NADP+) activity | 42 |
| molecular function | UDP-glucose:hexose-1-phosphate uridylyltransferase activity | 42 |
| molecular function | dipeptide transporter activity | 42 |
| molecular function | rRNA (uridine-N3-)-methyltransferase activity | 42 |
| molecular function | CoA-transferase activity | 42 |
| molecular function | hydroxymethylbilane synthase activity | 42 |
| molecular function | 1-phosphofructokinase activity | 42 |
| molecular function | porphobilinogen synthase activity | 42 |
| molecular function | methylenetetrahydrofolate reductase (NAD(P)H) activity | 41 |
| molecular function | quinolinate synthetase A activity | 41 |
| molecular function | succinate transmembrane transporter activity | 41 |
| molecular function | quinone binding | 41 |
| molecular function | peptide:proton symporter activity | 41 |
| molecular function | fumarate transmembrane transporter activity | 41 |
| molecular function | pyruvate dehydrogenase (acetyl-transferring) activity | 41 |
| molecular function | phosphate acetyltransferase activity | 41 |
| molecular function | prephenate dehydrogenase activity | 41 |
| molecular function | gamma-glutamyl-gamma-aminobutyrate hydrolase activity | 41 |
| molecular function | ferrous iron transmembrane transporter activity | 41 |
| molecular function | glutathione transferase activity | 41 |
| molecular function | thymidine kinase activity | 40 |
| molecular function | phosphomannomutase activity | 40 |
| molecular function | diaminopimelate epimerase activity | 40 |
| molecular function | glutaminase activity | 40 |
| molecular function | ribosomal small subunit binding | 40 |
| molecular function | ATPase activity, coupled to transmembrane movement of ions, phosphorylative mechanism | 40 |
| molecular function | lysophospholipase activity | 40 |
| molecular function | anion transmembrane transporter activity | 40 |
| molecular function | thiamine pyrophosphate binding | 40 |
| molecular function | protein serine/threonine phosphatase activity | 40 |
| molecular function | peptidoglycan binding | 40 |
| molecular function | protein-phosphocysteine-galactitol-phosphotransferase system transporter activity | 40 |
| molecular function | oxidized pyrimidine nucleobase lesion DNA N-glycosylase activity | 39 |
| molecular function | catalase activity | 39 |
| molecular function | L-aspartate oxidase activity | 39 |
| molecular function | carbohydrate kinase activity | 39 |
| molecular function | glycerol-3-phosphate dehydrogenase activity | 39 |
| molecular function | NAD-dependent protein deacetylase activity | 39 |
| molecular function | 2-isopropylmalate synthase activity | 39 |
| molecular function | tryptophan synthase activity | 38 |
| molecular function | aspartate ammonia-lyase activity | 38 |
| molecular function | L-seryl-tRNASec selenium transferase activity | 38 |
| molecular function | water channel activity | 38 |
| molecular function | dihydropyrimidine dehydrogenase (NADP+) activity | 38 |
| molecular function | cytochrome-c oxidase activity | 38 |
| molecular function | succinate-CoA ligase (ADP-forming) activity | 38 |
| molecular function | 2,3-bisphosphoglycerate-dependent phosphoglycerate mutase activity | 38 |
| molecular function | cytidine kinase activity | 38 |
| molecular function | glycerol channel activity | 38 |
| molecular function | 4-hydroxy-tetrahydrodipicolinate reductase | 38 |
| molecular function | 5-methyltetrahydropteroyltriglutamate-homocysteine S-methyltransferase activity | 38 |
| molecular function | ferric iron binding | 38 |
| molecular function | pyridoxal kinase activity | 38 |
| molecular function | signal sequence binding | 38 |
| molecular function | pyrophosphatase activity | 38 |
| molecular function | fatty acid binding | 37 |
| molecular function | hydrolase activity, acting on carbon-nitrogen (but not peptide) bonds, in cyclic amides | 37 |
| molecular function | gluconate transmembrane transporter activity | 37 |
| molecular function | cofactor binding | 37 |
| molecular function | adenylate cyclase activity | 37 |
| molecular function | homoserine O-succinyltransferase activity | 37 |
| molecular function | nucleoside diphosphate kinase activity | 37 |
| molecular function | N2-acetyl-L-ornithine:2-oxoglutarate 5-aminotransferase activity | 37 |
| molecular function | glutamate 5-kinase activity | 37 |
| molecular function | NADH dehydrogenase (quinone) activity | 37 |
| molecular function | mitochondrial light strand promoter anti-sense binding | 36 |
| molecular function | hypoxanthine phosphoribosyltransferase activity | 36 |
| molecular function | mannonate dehydratase activity | 36 |
| molecular function | glycoside-pentoside-hexuronide:cation symporter activity | 36 |
| molecular function | purine nucleoside transmembrane transporter activity | 36 |
| molecular function | holo-[acyl-carrier-protein] synthase activity | 36 |
| molecular function | fructokinase activity | 36 |
| molecular function | CTP synthase activity | 36 |
| molecular function | citrate (pro-3S)-lyase activity | 36 |
| molecular function | prephenate dehydratase activity | 36 |
| molecular function | selenide, water dikinase activity | 36 |
| molecular function | cation:cation antiporter activity | 36 |
| molecular function | antioxidant activity | 36 |
| molecular function | xanthine phosphoribosyltransferase activity | 35 |
| molecular function | glycerate kinase activity | 35 |
| molecular function | L-threonine ammonia-lyase activity | 35 |
| molecular function | methylthioadenosine nucleosidase activity | 35 |
| molecular function | methylglyoxal synthase activity | 35 |
| molecular function | biotin-[acetyl-CoA-carboxylase] ligase activity | 35 |
| molecular function | ATP-dependent 5'-3' DNA helicase activity | 35 |
| molecular function | ferredoxin hydrogenase activity | 35 |
| molecular function | actin filament binding | 35 |
| molecular function | pyruvate carboxylase activity | 35 |
| molecular function | oxidoreductase activity, acting on iron-sulfur proteins as donors | 35 |
| molecular function | transferase activity, transferring pentosyl groups | 35 |
| molecular function | alpha-mannosidase activity | 34 |
| molecular function | hydrogen dehydrogenase (NADP+) activity | 34 |
| molecular function | acid phosphatase activity | 34 |
| molecular function | chondroitin hydrolase activity | 34 |
| molecular function | N-acetyl-gamma-glutamyl-phosphate reductase activity | 34 |
| molecular function | phosphoglycolate phosphatase activity | 34 |
| molecular function | O-phospho-L-serine:2-oxoglutarate aminotransferase activity | 34 |
| molecular function | histidine phosphotransfer kinase activity | 34 |
| molecular function | intramolecular transferase activity | 34 |
| molecular function | oxidoreductase activity, acting on a sulfur group of donors, NAD(P) as acceptor | 34 |
| molecular function | adenine phosphoribosyltransferase activity | 34 |
| molecular function | butyrate kinase activity | 34 |
| molecular function | selenocysteine insertion sequence binding | 34 |
| molecular function | glutamyl-tRNA reductase activity | 34 |
| molecular function | calmodulin binding | 34 |
| molecular function | monovalent cation:proton antiporter activity | 33 |
| molecular function | histidinol-phosphatase activity | 33 |
| molecular function | GTP cyclohydrolase II activity | 33 |
| molecular function | glycerol-3-phosphate-transporting ATPase activity | 33 |
| molecular function | 3-isopropylmalate dehydrogenase activity | 33 |
| molecular function | transcription corepressor activity | 33 |
| molecular function | phosphopantothenate--cysteine ligase activity | 33 |
| molecular function | lactate transmembrane transporter activity | 33 |
| molecular function | tRNA-specific ribonuclease activity | 33 |
| molecular function | ATPase binding | 33 |
| molecular function | glucose transmembrane transporter activity | 33 |
| molecular function | beta-ketoacyl-acyl-carrier-protein synthase III activity | 33 |
| molecular function | ornithine decarboxylase activity | 33 |
| molecular function | voltage-gated chloride channel activity | 33 |
| molecular function | lipid binding | 32 |
| molecular function | imidazoleglycerol-phosphate synthase activity | 32 |
| molecular function | prephenate dehydrogenase (NADP+) activity | 32 |
| molecular function | alkanesulfonate transporter activity | 32 |
| molecular function | N-methyltransferase activity | 32 |
| molecular function | protein adenylyltransferase activity | 32 |
| molecular function | oxoglutarate dehydrogenase (succinyl-transferring) activity | 32 |
| molecular function | D-proline reductase (dithiol) activity | 32 |
| molecular function | alkaline phosphatase activity | 32 |
| molecular function | tripeptide transporter activity | 32 |
| molecular function | 4-alpha-glucanotransferase activity | 32 |
| molecular function | 3-hydroxyacyl-CoA dehydrogenase activity | 32 |
| molecular function | cysteine-type peptidase activity | 32 |
| molecular function | 3-methyl-2-oxobutanoate hydroxymethyltransferase activity | 32 |
| molecular function | glycerol-3-phosphate dehydrogenase [NAD(P)+] activity | 32 |
| molecular function | DNA replication origin binding | 32 |
| molecular function | 1,3-beta-galactosyl-N-acetylhexosamine phosphorylase activity | 32 |
| molecular function | 4-hydroxy-2-oxoglutarate aldolase activity | 31 |
| molecular function | zinc efflux active transmembrane transporter activity | 31 |
| molecular function | 5-amino-6-(5-phosphoribosylamino)uracil reductase activity | 31 |
| molecular function | 2-amino-4-hydroxy-6-hydroxymethyldihydropteridine diphosphokinase activity | 31 |
| molecular function | phosphatidylserine decarboxylase activity | 31 |
| molecular function | precorrin-4 C11-methyltransferase activity | 31 |
| molecular function | D-lactate dehydrogenase activity | 31 |
| molecular function | sucrose alpha-glucosidase activity | 31 |
| molecular function | precorrin-6Y C5,15-methyltransferase (decarboxylating) activity | 31 |
| molecular function | inositol monophosphate 1-phosphatase activity | 31 |
| molecular function | glycine dehydrogenase (decarboxylating) activity | 31 |
| molecular function | 4-amino-4-deoxychorismate synthase activity | 31 |
| molecular function | toxin transporter activity | 31 |
| molecular function | diaminohydroxyphosphoribosylaminopyrimidine deaminase activity | 31 |
| molecular function | glucuronate isomerase activity | 31 |
| molecular function | hydroxymethylbutenyl pyrophosphate reductase activity | 31 |
| molecular function | nitrate reductase activity | 31 |
| molecular function | phosphoenolpyruvate-protein phosphotransferase activity | 31 |
| molecular function | 3,4-dihydroxy-2-butanone-4-phosphate synthase activity | 31 |
| molecular function | 2-dehydro-3-deoxy-phosphogluconate aldolase activity | 30 |
| molecular function | phosphoenolpyruvate carboxylase activity | 30 |
| molecular function | glutamate decarboxylase activity | 30 |
| molecular function | carbonate dehydratase activity | 30 |
| molecular function | maltose alpha-glucosidase activity | 30 |
| molecular function | copper ion transmembrane transporter activity | 30 |
| molecular function | carbon-oxygen lyase activity, acting on polysaccharides | 30 |
| molecular function | uracil transmembrane transporter activity | 30 |
| molecular function | 8-oxo-7,8-dihydroguanosine triphosphate pyrophosphatase activity | 30 |
| molecular function | protein tyrosine kinase activity | 30 |
| molecular function | ketol-acid reductoisomerase activity | 30 |
| molecular function | glyoxylate reductase (NADP) activity | 29 |
| molecular function | 8-amino-7-oxononanoate synthase activity | 29 |
| molecular function | oxidoreductase activity, acting on the CH-NH group of donors, NAD or NADP as acceptor | 29 |
| molecular function | glutamate synthase (NADPH) activity | 29 |
| molecular function | 3-isopropylmalate dehydratase activity | 29 |
| molecular function | hydroxypyruvate reductase activity | 29 |
| molecular function | dihydroxy-acid dehydratase activity | 29 |
| molecular function | methylmalonyl-CoA mutase activity | 29 |
| molecular function | pantoate-beta-alanine ligase activity | 29 |
| molecular function | aspartic-type endopeptidase activity | 29 |
| molecular function | precorrin-8X methylmutase activity | 28 |
| molecular function | tRNA (5-methylaminomethyl-2-thiouridylate)-methyltransferase activity | 28 |
| molecular function | arabinose isomerase activity | 28 |
| molecular function | glutathione transmembrane transporter activity | 28 |
| molecular function | potassium channel regulator activity | 28 |
| molecular function | sedoheptulose-7-phosphate:D-glyceraldehyde-3-phosphate glyceronetransferase activity | 28 |
| molecular function | aminoacylase activity | 28 |
| molecular function | thymidine phosphorylase activity | 28 |
| molecular function | galactokinase activity | 28 |
| molecular function | riboflavin reductase (NADPH) activity | 28 |
| molecular function | L-fucose isomerase activity | 28 |
| molecular function | carbohydrate transmembrane transporter activity | 28 |
| molecular function | exodeoxyribonuclease V activity | 28 |
| molecular function | heme transporter activity | 28 |
| molecular function | 2,3,4,5-tetrahydropyridine-2,6-dicarboxylate N-succinyltransferase activity | 28 |
| molecular function | oxidized purine nucleobase lesion DNA N-glycosylase activity | 28 |
| molecular function | cobinamide phosphate guanylyltransferase activity | 27 |
| molecular function | dimethyl sulfoxide reductase activity | 27 |
| molecular function | histidine ammonia-lyase activity | 27 |
| molecular function | maltose-transporting ATPase activity | 27 |
| molecular function | pyruvate dehydrogenase activity | 27 |
| molecular function | cysteine-type endopeptidase activity | 27 |
| molecular function | cobinamide kinase activity | 27 |
| molecular function | monosaccharide binding | 27 |
| molecular function | methylenetetrahydrofolate dehydrogenase [NAD(P)+] activity | 27 |
| molecular function | heparin binding | 27 |
| molecular function | phosphoenolpyruvate carboxykinase (ATP) activity | 27 |
| molecular function | UDP-N-acetylmuramoylalanyl-D-glutamate-2,6-diaminopimelate ligase activity | 27 |
| molecular function | thymidylate synthase (FAD) activity | 27 |
| molecular function | riboflavin synthase activity | 27 |
| molecular function | 6-phospho-beta-glucosidase activity | 27 |
| molecular function | hydroxymethyl-, formyl- and related transferase activity | 27 |
| molecular function | agmatinase activity | 26 |
| molecular function | S-adenosylhomocysteine deaminase activity | 26 |
| molecular function | protein-N(PI)-phosphohistidine-fructose phosphotransferase system transporter activity | 26 |
| molecular function | lipopolysaccharide-transporting ATPase activity | 26 |
| molecular function | nucleoside-triphosphatase activity | 26 |
| molecular function | radical SAM enzyme activity | 26 |
| molecular function | NADP+ binding | 26 |
| molecular function | chitin binding | 26 |
| molecular function | enoyl-CoA hydratase activity | 26 |
| molecular function | drug binding | 26 |
| molecular function | glutamate N-acetyltransferase activity | 26 |
| molecular function | ribonuclease M5 activity | 26 |
| molecular function | tagatose-6-phosphate kinase activity | 26 |
| molecular function | 2,3-bisphosphoglycerate-independent phosphoglycerate mutase activity | 26 |
| molecular function | glycine binding | 26 |
| molecular function | melibiose:sodium symporter activity | 25 |
| molecular function | sedoheptulose-bisphosphatase activity | 25 |
| molecular function | phosphoribosyl-AMP cyclohydrolase activity | 25 |
| molecular function | sigma factor antagonist activity | 25 |
| molecular function | cyclic-guanylate-specific phosphodiesterase activity | 25 |
| molecular function | methylgalactoside transmembrane transporter activity | 25 |
| molecular function | phosphoglucomutase activity | 25 |
| molecular function | tetrapyrrole binding | 25 |
| molecular function | thioredoxin peroxidase activity | 25 |
| molecular function | melibiose:monovalent cation symporter activity | 25 |
| molecular function | lactoylglutathione lyase activity | 25 |
| molecular function | fructose 1,6-bisphosphate 1-phosphatase activity | 25 |
| molecular function | L-threonine 3-dehydrogenase activity | 25 |
| molecular function | guanine deaminase activity | 25 |
| molecular function | drug:proton antiporter activity | 25 |
| molecular function | single-stranded RNA binding | 25 |
| molecular function | mannosyltransferase activity | 25 |
| molecular function | protein methyltransferase activity | 25 |
| molecular function | acetylglutamate kinase activity | 25 |
| molecular function | lipoate-protein ligase activity | 25 |
| molecular function | N-acetylneuraminate lyase activity | 25 |
| molecular function | platelet-derived growth factor binding | 25 |
| molecular function | structural constituent of muscle | 25 |
| molecular function | transferase activity, transferring sulfur-containing groups | 25 |
| molecular function | 2-methylcitrate synthase activity | 24 |
| molecular function | hydrogenase (acceptor) activity | 24 |
| molecular function | tetraacyldisaccharide 4'-kinase activity | 24 |
| molecular function | single-stranded DNA endodeoxyribonuclease activity | 24 |
| molecular function | spermidine synthase activity | 24 |
| molecular function | acetylglucosaminyltransferase activity | 24 |
| molecular function | UDP-N-acetylmuramoyl-tripeptide-D-alanyl-D-alanine ligase activity | 24 |
| molecular function | ferrochelatase activity | 24 |
| molecular function | polyamine binding | 24 |
| molecular function | starch binding | 24 |
| molecular function | carbon-monoxide dehydrogenase (acceptor) activity | 24 |
| molecular function | N-acylneuraminate cytidylyltransferase activity | 24 |
| molecular function | 2',3'-cyclic-nucleotide 2'-phosphodiesterase activity | 24 |
| molecular function | bacterial-type RNA polymerase holo enzyme binding | 24 |
| molecular function | prenyltransferase activity | 24 |
| molecular function | calcium-transporting ATPase activity | 24 |
| molecular function | acetyl-CoA:L-glutamate N-acetyltransferase activity | 24 |
| molecular function | glycine C-acetyltransferase activity | 24 |
| molecular function | organic acid transmembrane transporter activity | 24 |
| molecular function | tagatose-bisphosphate aldolase activity | 23 |
| molecular function | cobalamin 5'-phosphate synthase activity | 23 |
| molecular function | glycerol-3-phosphate dehydrogenase [NAD+] activity | 23 |
| molecular function | di-trans,poly-cis-decaprenylcistransferase activity | 23 |
| molecular function | structural constituent of cytoskeleton | 23 |
| molecular function | mRNA 3'-UTR binding | 23 |
| molecular function | carbohydrate-importing ATPase activity | 23 |
| molecular function | lipoprotein transporter activity | 23 |
| molecular function | protein-phosphocysteine-L-ascorbate-phosphotransferase system transporter activity | 23 |
| molecular function | urocanate hydratase activity | 23 |
| molecular function | exo-alpha-sialidase activity | 23 |
| molecular function | arginine binding | 23 |
| molecular function | transmembrane electron transfer carrier | 23 |
| molecular function | cysteine desulfurase activity | 23 |
| molecular function | endoribonuclease activity, producing 3'-phosphomonoesters | 23 |
| molecular function | phospholipid transporter activity | 23 |
| molecular function | uridine phosphorylase activity | 22 |
| molecular function | acylphosphatase activity | 22 |
| molecular function | protein disulfide isomerase activity | 22 |
| molecular function | cytoskeletal protein binding | 22 |
| molecular function | DNA-3-methyladenine glycosylase activity | 22 |
| molecular function | beta-aspartyl-peptidase activity | 22 |
| molecular function | adenosylmethionine decarboxylase activity | 22 |
| molecular function | 3-deoxy-manno-octulosonate cytidylyltransferase activity | 22 |
| molecular function | formate transmembrane transporter activity | 22 |
| molecular function | FMN reductase activity | 22 |
| molecular function | glutathione-disulfide reductase activity | 22 |
| molecular function | metallodipeptidase activity | 22 |
| molecular function | galactose binding | 22 |
| molecular function | dihydroneopterin aldolase activity | 22 |
| molecular function | 2-dehydro-3-deoxygluconokinase activity | 22 |
| molecular function | 6,7-dimethyl-8-ribityllumazine synthase activity | 22 |
| molecular function | 2-methylisocitrate dehydratase activity | 22 |
| molecular function | cysteine transmembrane transporter activity | 21 |
| molecular function | glutathione peroxidase activity | 21 |
| molecular function | diguanylate cyclase activity | 21 |
| molecular function | cation:sugar symporter activity | 21 |
| molecular function | ubiquinol-cytochrome-c reductase activity | 21 |
| molecular function | oleate hydratase activity | 21 |
| molecular function | protein-phosphocysteine-glucosamine phosphotransferase system transporter activity | 21 |
| molecular function | potassium channel activity | 21 |
| molecular function | transcription factor activity, bacterial-type RNA polymerase core promoter proximal region sequence-specific binding | 21 |
| molecular function | protein binding, bridging | 21 |
| molecular function | oxidoreductase activity, acting on paired donors, with incorporation or reduction of molecular oxygen, NAD(P)H as one donor, and incorporation of one atom of oxygen | 21 |
| molecular function | protein-phosphocysteine-N,N'-diacetylchitobiose phosphotransferase system transporter activity | 21 |
| molecular function | enzyme binding | 21 |
| molecular function | biotin binding | 21 |
| molecular function | formyltetrahydrofolate deformylase activity | 21 |
| molecular function | lipopolysaccharide glucosyltransferase I activity | 21 |
| molecular function | alcohol dehydrogenase (NADP+) activity | 21 |
| molecular function | uridine nucleosidase activity | 21 |
| molecular function | voltage-gated potassium channel activity | 21 |
| molecular function | N-acetylglucosamine transmembrane transporter activity | 21 |
| molecular function | glycerol dehydrogenase [NAD+] activity | 21 |
| molecular function | protein-phosphocysteine-N-acetylglucosamine phosphotransferase system transporter activity | 21 |
| molecular function | glutamate dehydrogenase (NAD+) activity | 21 |
| molecular function | citrate synthase activity | 21 |
| molecular function | maltose O-acetyltransferase activity | 21 |
| molecular function | cobalamin-transporting ATPase activity | 21 |
| molecular function | L-arabinose-importing ATPase activity | 21 |
| molecular function | glucosyltransferase activity | 21 |
| molecular function | Ser-tRNA(Ala) hydrolase activity | 21 |
| molecular function | acetate CoA-transferase activity | 21 |
| molecular function | hydrogen-translocating pyrophosphatase activity | 21 |
| molecular function | glycolate oxidase activity | 20 |
| molecular function | uracil phosphoribosyltransferase activity | 20 |
| molecular function | dioxygenase activity | 20 |
| molecular function | dihydrolipoyllysine-residue acetyltransferase activity | 20 |
| molecular function | catalysis of free radical formation | 20 |
| molecular function | translation release factor activity | 20 |
| molecular function | putrescine transmembrane transporter activity | 20 |
| molecular function | galactose:proton symporter activity | 20 |
| molecular function | diphthine synthase activity | 20 |
| molecular function | peroxiredoxin activity | 20 |
| molecular function | dethiobiotin synthase activity | 20 |
| molecular function | biotin carboxylase activity | 20 |
| molecular function | phosphate butyryltransferase activity | 20 |
| molecular function | peptidoglycan glycosyltransferase activity | 20 |
| molecular function | L-cystine transmembrane transporter activity | 20 |
| molecular function | glutamate-cysteine ligase activity | 20 |
| molecular function | phosphoribosylanthranilate isomerase activity | 20 |
| molecular function | acyl-[acyl-carrier-protein]-UDP-N-acetylglucosamine O-acyltransferase activity | 20 |
| molecular function | galactoside O-acetyltransferase activity | 20 |
| molecular function | 3-hydroxybutyryl-CoA dehydrogenase activity | 20 |
| molecular function | quaternary-ammonium-compound-transporting ATPase activity | 20 |
| molecular function | hydroperoxide reductase activity | 20 |
| molecular function | 3 iron, 4 sulfur cluster binding | 20 |
| molecular function | selenocysteine lyase activity | 20 |
| molecular function | fucose binding | 20 |
| molecular function | cadmium ion binding | 19 |
| molecular function | mannose-1-phosphate guanylyltransferase activity | 19 |
| molecular function | alpha,alpha-trehalase activity | 19 |
| molecular function | phosphoribosyl-ATP diphosphatase activity | 19 |
| molecular function | dimethylmaleate hydratase activity | 19 |
| molecular function | arabinose:proton symporter activity | 19 |
| molecular function | glycerone kinase activity | 19 |
| molecular function | ferric-enterobactin transmembrane transporter activity | 19 |
| molecular function | alpha-L-arabinofuranosidase activity | 19 |
| molecular function | proline:sodium symporter activity | 19 |
| molecular function | rRNA (guanine-N2-)-methyltransferase activity | 19 |
| molecular function | CoA hydrolase activity | 19 |
| molecular function | manganese ion transmembrane transporter activity | 19 |
| molecular function | glutamate synthase activity | 19 |
| molecular function | transcription regulatory region sequence-specific DNA binding | 19 |
| molecular function | intramolecular transferase activity, transferring amino groups | 19 |
| molecular function | pantothenate transmembrane transporter activity | 19 |
| molecular function | fucose:proton symporter activity | 19 |
| molecular function | alanine:sodium symporter activity | 19 |
| molecular function | thiosulfate sulfurtransferase activity | 19 |
| molecular function | transferase activity, transferring acyl groups, acyl groups converted into alkyl on transfer | 19 |
| molecular function | intramolecular transferase activity, phosphotransferases | 18 |
| molecular function | [glutamate-ammonia-ligase] adenylyltransferase activity | 18 |
| molecular function | UDP-glycosyltransferase activity | 18 |
| molecular function | hydroxyethylthiazole kinase activity | 18 |
| molecular function | oxidoreductase activity, acting on the CH-CH group of donors, NAD or NADP as acceptor | 18 |
| molecular function | homoserine kinase activity | 18 |
| molecular function | oxygen binding | 18 |
| molecular function | maltose-6'-phosphate glucosidase activity | 18 |
| molecular function | aminomethyltransferase activity | 18 |
| molecular function | tRNA (guanine-N1-)-methyltransferase activity | 18 |
| molecular function | (R)-aminopropanol dehydrogenase activity | 18 |
| molecular function | beta-lactamase activity | 18 |
| molecular function | protein kinase binding | 18 |
| molecular function | protein-phosphocysteine-trehalose phosphotransferase system transporter activity | 18 |
| molecular function | L-malate dehydrogenase activity | 18 |
| molecular function | molybdopterin adenylyltransferase activity | 18 |
| molecular function | 1-acylglycerol-3-phosphate O-acyltransferase activity | 18 |
| molecular function | sirohydrochlorin ferrochelatase activity | 18 |
| molecular function | 1-(5-phosphoribosyl)-5-[(5-phosphoribosylamino)methylideneamino]imidazole-4-carboxamide isomerase activity | 18 |
| molecular function | tRNA (adenine-N1-)-methyltransferase activity | 18 |
| molecular function | alpha,alpha-phosphotrehalase activity | 18 |
| molecular function | ATP phosphoribosyltransferase activity | 18 |
| molecular function | NAD(P)H dehydrogenase (quinone) activity | 18 |
| molecular function | cyclic-di-GMP binding | 18 |
| molecular function | alkylphosphonate transmembrane transporter activity | 18 |
| molecular function | RNA polymerase binding | 18 |
| molecular function | arginine decarboxylase activity | 18 |
| molecular function | formate dehydrogenase (quinone) activity | 17 |
| molecular function | aryl sulfotransferase activity | 17 |
| molecular function | anion:cation symporter activity | 17 |
| molecular function | oxo-acid-lyase activity | 17 |
| molecular function | S-methyl-5-thioribose-1-phosphate isomerase activity | 17 |
| molecular function | spermidine-importing ATPase activity | 17 |
| molecular function | UDP-3-O-[3-hydroxymyristoyl] N-acetylglucosamine deacetylase activity | 17 |
| molecular function | glycogen debranching enzyme activity | 17 |
| molecular function | thiamine diphosphokinase activity | 17 |
| molecular function | N-acyltransferase activity | 17 |
| molecular function | 4-aminobutyrate transaminase activity | 17 |
| molecular function | oligo-1,6-glucosidase activity | 17 |
| molecular function | hydrolase activity, acting on carbon-nitrogen (but not peptide) bonds, in linear amidines | 17 |
| molecular function | sulfate transmembrane-transporting ATPase activity | 17 |
| molecular function | 3-deoxy-8-phosphooctulonate synthase activity | 17 |
| molecular function | pantothenate kinase activity | 17 |
| molecular function | 4-deoxy-L-threo-5-hexosulose-uronate ketol-isomerase activity | 17 |
| molecular function | glucosidase activity | 17 |
| molecular function | uroporphyrinogen decarboxylase activity | 17 |
| molecular function | glycolate transmembrane transporter activity | 17 |
| molecular function | magnesium chelatase activity | 17 |
| molecular function | saccharopine dehydrogenase (NAD+, L-lysine-forming) activity | 17 |
| molecular function | 1,4-dihydroxy-2-naphthoyl-CoA synthase activity | 17 |
| molecular function | microfilament motor activity | 17 |
| molecular function | imidazoleglycerol-phosphate dehydratase activity | 17 |
| molecular function | choline transmembrane transporter activity | 17 |
| molecular function | citrate (Si)-synthase activity | 17 |
| molecular function | nitric oxide dioxygenase activity | 17 |
| molecular function | amino-acid racemase activity | 17 |
| molecular function | sugar-phosphatase activity | 16 |
| molecular function | alkylbase DNA N-glycosylase activity | 16 |
| molecular function | O-methyltransferase activity | 16 |
| molecular function | dihydrolipoyllysine-residue succinyltransferase activity | 16 |
| molecular function | 2,4-dienoyl-CoA reductase (NADPH) activity | 16 |
| molecular function | precorrin-6A reductase activity | 16 |
| molecular function | NAD+ synthase (glutamine-hydrolyzing) activity | 16 |
| molecular function | indole-3-glycerol-phosphate synthase activity | 16 |
| molecular function | aldo-keto reductase (NADP) activity | 16 |
| molecular function | D-methionine transmembrane transporter activity | 16 |
| molecular function | succinate dehydrogenase (ubiquinone) activity | 16 |
| molecular function | NAD(P)+ transhydrogenase (AB-specific) activity | 16 |
| molecular function | tRNA adenylyltransferase activity | 16 |
| molecular function | G-quadruplex DNA binding | 16 |
| molecular function | D-ribose transmembrane transporter activity | 16 |
| molecular function | heat shock protein binding | 16 |
| molecular function | dihydrofolate synthase activity | 16 |
| molecular function | DNA ligase activity | 16 |
| molecular function | 3-hydroxybutyryl-CoA dehydratase activity | 16 |
| molecular function | host cell surface binding | 16 |
| molecular function | NADH pyrophosphatase activity | 16 |
| molecular function | shikimate 3-dehydrogenase (NAD+) activity | 16 |
| molecular function | sn-glycerol-3-phosphate:ubiquinone-8 oxidoreductase activity | 16 |
| molecular function | D-sedoheptulose 7-phosphate isomerase activity | 16 |
| molecular function | ribosomal-protein-alanine N-acetyltransferase activity | 16 |
| molecular function | exopolyphosphatase activity | 16 |
| molecular function | dCTP deaminase activity | 16 |
| molecular function | racemase and epimerase activity | 16 |
| molecular function | single-stranded telomeric DNA binding | 16 |
| molecular function | L-alanine:2-oxoglutarate aminotransferase activity | 16 |
| molecular function | arabinose-5-phosphate isomerase activity | 16 |
| molecular function | 3-hydroxyisobutyrate dehydrogenase activity | 16 |
| molecular function | lipoate synthase activity | 16 |
| molecular function | sulfur carrier activity | 16 |
| molecular function | carboxyl- or carbamoyltransferase activity | 16 |
| molecular function | propionyl-CoA:succinate CoA-transferase activity | 16 |
| molecular function | IgA binding | 16 |
| molecular function | tyrosine-based site-specific recombinase activity | 16 |
| molecular function | quinate 3-dehydrogenase (NAD+) activity | 16 |
| molecular function | quinate 3-dehydrogenase (NADP+) activity | 16 |
| molecular function | toxic substance binding | 16 |
| molecular function | double-stranded telomeric DNA binding | 15 |
| molecular function | potassium:proton antiporter activity | 15 |
| molecular function | acyl-CoA hydrolase activity | 15 |
| molecular function | mannose binding | 15 |
| molecular function | mannose-1-phosphate guanylyltransferase (GDP) activity | 15 |
| molecular function | chloride ion binding | 15 |
| molecular function | diglucosyl diacylglycerol synthase activity | 15 |
| molecular function | threonine efflux transmembrane transporter activity | 15 |
| molecular function | glycerate 2-kinase activity | 15 |
| molecular function | pyrimidine-nucleoside phosphorylase activity | 15 |
| molecular function | nitrate transmembrane transporter activity | 15 |
| molecular function | xanthine oxidase activity | 15 |
| molecular function | 2-dehydropantoate 2-reductase activity | 15 |
| molecular function | betaine-aldehyde dehydrogenase activity | 15 |
| molecular function | chloramphenicol O-acetyltransferase activity | 15 |
| molecular function | transcription antitermination factor activity, RNA binding | 15 |
| molecular function | 1,2-diacylglycerol 3-glucosyltransferase activity | 15 |
| molecular function | thiamine transmembrane transporter activity | 15 |
| molecular function | peptidoglycan L,D-transpeptidase activity | 15 |
| molecular function | NAD+ diphosphatase activity | 15 |
| molecular function | lipoic acid binding | 15 |
| molecular function | beta-phosphoglucomutase activity | 15 |
| molecular function | alanine dehydrogenase activity | 15 |
| molecular function | arylformamidase activity | 15 |
| molecular function | protein-disulfide reductase activity | 15 |
| molecular function | beta-mannosidase activity | 15 |
| molecular function | cellulase activity | 15 |
| molecular function | undecaprenyl-phosphate glucose phosphotransferase activity | 15 |
| molecular function | CDP-diacylglycerol-glycerol-3-phosphate 3-phosphatidyltransferase activity | 14 |
| molecular function | Ala-tRNA(Pro) hydrolase activity | 14 |
| molecular function | IAA-amino acid conjugate hydrolase activity | 14 |
| molecular function | cation:amino acid symporter activity | 14 |
| molecular function | allose 6-phosphate isomerase activity | 14 |
| molecular function | maltose transmembrane transporter activity | 14 |
| molecular function | isochorismate synthase activity | 14 |
| molecular function | thiolester hydrolase activity | 14 |
| molecular function | 3'(2'),5'-bisphosphate nucleotidase activity | 14 |
| molecular function | ATP-dependent microtubule motor activity, plus-end-directed | 14 |
| molecular function | xylan 1,4-beta-xylosidase activity | 14 |
| molecular function | ligase activity, forming aminoacyl-tRNA and related compounds | 14 |
| molecular function | gluconate 5-dehydrogenase activity | 14 |
| molecular function | ubiquitin protein ligase activity | 14 |
| molecular function | nitrite reductase (cytochrome, ammonia-forming) activity | 14 |
| molecular function | anion binding | 14 |
| molecular function | D-serine ammonia-lyase activity | 14 |
| molecular function | acetaldehyde dehydrogenase (acetylating) activity | 14 |
| molecular function | adenosylcobinamide kinase activity | 14 |
| molecular function | oxidoreductase activity, acting on superoxide radicals as acceptor | 14 |
| molecular function | bacterial-type RNA polymerase core enzyme binding | 14 |
| molecular function | molybdopterin molybdotransferase activity | 14 |
| molecular function | rRNA (cytosine) methyltransferase activity | 14 |
| molecular function | aspartate 1-decarboxylase activity | 14 |
| molecular function | folic acid binding | 14 |
| molecular function | histidinol dehydrogenase activity | 14 |
| molecular function | N-acetylglucosaminyldiphosphodolichol N-acetylglucosaminyltransferase activity | 14 |
| molecular function | protein C-terminus binding | 14 |
| molecular function | metallochaperone activity | 13 |
| molecular function | RNA ligase activity | 13 |
| molecular function | purine nucleobase transmembrane transporter activity | 13 |
| molecular function | polar-amino acid-transporting ATPase activity | 13 |
| molecular function | actin-dependent ATPase activity | 13 |
| molecular function | methylmalonate-semialdehyde dehydrogenase (acylating) activity | 13 |
| molecular function | fructose 6-phosphate aldolase activity | 13 |
| molecular function | cobalamin transporter activity | 13 |
| molecular function | dipeptide transmembrane transporter activity | 13 |
| molecular function | D-amino-acid dehydrogenase activity | 13 |
| molecular function | heparin lyase activity | 13 |
| molecular function | cytosine deaminase activity | 13 |
| molecular function | [protein-PII] uridylyltransferase activity | 13 |
| molecular function | coproporphyrinogen dehydrogenase activity | 13 |
| molecular function | acetate transmembrane transporter activity | 13 |
| molecular function | 3'-5'-exodeoxyribonuclease activity | 13 |
| molecular function | phosphopantetheine binding | 13 |
| molecular function | ubiquinone binding | 13 |
| molecular function | methionine gamma-lyase activity | 13 |
| molecular function | lipid transporter activity | 13 |
| molecular function | chloride transmembrane transporter activity | 13 |
| molecular function | precorrin-2 dehydrogenase activity | 13 |
| molecular function | 4-phosphoerythronate dehydrogenase activity | 13 |
| molecular function | integrin binding | 13 |
| molecular function | biotin synthase activity | 13 |
| molecular function | 1-pyrroline-5-carboxylate dehydrogenase activity | 13 |
| molecular function | 5'-flap endonuclease activity | 13 |
| molecular function | GDP-mannose 4,6-dehydratase activity | 13 |
| molecular function | beta-glucuronidase activity | 13 |
| molecular function | L-ribulose-5-phosphate 3-epimerase activity | 13 |
| molecular function | phosphatidylglycerophosphatase activity | 13 |
| molecular function | choline kinase activity | 13 |
| molecular function | 6-aminohexanoate-cyclic-dimer hydrolase activity | 13 |
| molecular function | 1,2-alpha-L-fucosidase activity | 13 |
| molecular function | thiamine-phosphate kinase activity | 13 |
| molecular function | triphosphatase activity | 13 |
| molecular function | isoamylase activity | 13 |
| molecular function | oleic acid binding | 13 |
| molecular function | 2-hydroxy-3-oxopropionate reductase activity | 13 |
| molecular function | arginine deiminase activity | 13 |
| molecular function | xanthan lyase activity | 13 |
| molecular function | rRNA (uridine) methyltransferase activity | 13 |
| molecular function | isoguanine deaminase activity | 13 |
| molecular function | cystathionine beta-lyase activity | 13 |
| molecular function | arsenite transmembrane transporter activity | 13 |
| molecular function | pullulanase activity | 13 |
| molecular function | glucosamine-1-phosphate N-acetyltransferase activity | 13 |
| molecular function | lipoyltransferase activity | 13 |
| molecular function | bis(5'-nucleosyl)-tetraphosphatase (symmetrical) activity | 12 |
| molecular function | 5'-3' exoribonuclease activity | 12 |
| molecular function | [citrate (pro-3S)-lyase] ligase activity | 12 |
| molecular function | RNA polymerase core enzyme binding | 12 |
| molecular function | nickel-transporting ATPase activity | 12 |
| molecular function | bent DNA binding | 12 |
| molecular function | dTDP-4-amino-4,6-dideoxygalactose transaminase activity | 12 |
| molecular function | N-acetyldiaminopimelate deacetylase activity | 12 |
| molecular function | SMAD binding | 12 |
| molecular function | transferase activity, transferring amino-acyl groups | 12 |
| molecular function | methylmalonyl-CoA epimerase activity | 12 |
| molecular function | DNA ligase (ATP) activity | 12 |
| molecular function | proton motive force dependent protein transmembrane transporter activity | 12 |
| molecular function | ribosylpyrimidine nucleosidase activity | 12 |
| molecular function | malic enzyme activity | 12 |
| molecular function | aspartate 4-decarboxylase activity | 12 |
| molecular function | colicin transmembrane transporter activity | 12 |
| molecular function | L-phenylalanine:2-oxoglutarate aminotransferase activity | 12 |
| molecular function | ion transmembrane transporter activity | 12 |
| molecular function | formimidoyltetrahydrofolate cyclodeaminase activity | 12 |
| molecular function | pyrimidine nucleotide transmembrane transporter activity | 12 |
| molecular function | methylcrotonoyl-CoA carboxylase activity | 12 |
| molecular function | arsenate reductase (glutaredoxin) activity | 12 |
| molecular function | polysaccharide binding | 12 |
| molecular function | silver ion transmembrane transporter activity | 12 |
| molecular function | mannose-6-phosphate isomerase activity | 12 |
| molecular function | autotransporter activity | 12 |
| molecular function | glucose binding | 12 |
| molecular function | sulfopyruvate decarboxylase activity | 12 |
| molecular function | glucan exo-1,3-beta-glucosidase activity | 12 |
| molecular function | proline dehydrogenase activity | 12 |
| molecular function | L-tyrosine:2-oxoglutarate aminotransferase activity | 12 |
| molecular function | cAMP binding | 12 |
| molecular function | farnesyltranstransferase activity | 12 |
| molecular function | methylglyoxal reductase (NADPH-dependent, acetol producing) | 12 |
| molecular function | palmitoyl-(protein) hydrolase activity | 11 |
| molecular function | deoxyribonuclease IV (phage-T4-induced) activity | 11 |
| molecular function | transmembrane signaling receptor activity | 11 |
| molecular function | ammonium transmembrane transporter activity | 11 |
| molecular function | undecaprenyldiphospho-muramoylpentapeptide beta-N-acetylglucosaminyltransferase activity | 11 |
| molecular function | proline racemase activity | 11 |
| molecular function | nucleotidase activity | 11 |
| molecular function | kinetochore binding | 11 |
| molecular function | para-aminobenzoyl-glutamate hydrolase activity | 11 |
| molecular function | omega-amidase activity | 11 |
| molecular function | protein-phosphocysteine-N-acetylmuramate phosphotransferase system transporter activity | 11 |
| molecular function | telomerase inhibitor activity | 11 |
| molecular function | acetylgalactosaminyltransferase activity | 11 |
| molecular function | nitric oxide reductase activity | 11 |
| molecular function | isocitrate dehydrogenase (NAD+) activity | 11 |
| molecular function | phosphoenolpyruvate carboxykinase (GTP) activity | 11 |
| molecular function | chloride channel activity | 11 |
| molecular function | xanthine transmembrane transporter activity | 11 |
| molecular function | ATPase coupled ion transmembrane transporter activity | 11 |
| molecular function | AT DNA binding | 11 |
| molecular function | 3-octaprenyl-4-hydroxybenzoate carboxy-lyase activity | 11 |
| molecular function | acid-thiol ligase activity | 11 |
| molecular function | 2-hydroxyglutarate dehydrogenase activity | 11 |
| molecular function | hydrolase activity, acting on acid anhydrides | 11 |
| molecular function | polyphosphate:AMP phosphotransferase activity | 11 |
| molecular function | protein domain specific binding | 11 |
| molecular function | 2',3'-cyclic-nucleotide 3'-phosphodiesterase activity | 11 |
| molecular function | myosin II binding | 11 |
| molecular function | aromatic amino acid transmembrane transporter activity | 11 |
| molecular function | DNA insertion or deletion binding | 11 |
| molecular function | canonical holin activity | 11 |
| molecular function | ferric-transporting ATPase activity | 11 |
| molecular function | GMP reductase activity | 11 |
| molecular function | serine racemase activity | 11 |
| molecular function | cysteine-type carboxypeptidase activity | 11 |
| molecular function | snoRNA binding | 11 |
| molecular function | single-stranded DNA-dependent ATPase activity | 11 |
| molecular function | ADP-ribose diphosphatase activity | 11 |
| molecular function | 3-hydroxybutyryl-CoA epimerase activity | 11 |
| molecular function | lactate dehydrogenase activity | 11 |
| molecular function | cobalamin binding | 11 |
| molecular function | molybdate transmembrane-transporting ATPase activity | 11 |
| molecular function | UDP-galactosyltransferase activity | 10 |
| molecular function | D-glucose transmembrane transporter activity | 10 |
| molecular function | structural molecule activity conferring elasticity | 10 |
| molecular function | steroid hydroxylase activity | 10 |
| molecular function | tRNA (cytosine-2'-O-)-methyltransferase activity | 10 |
| molecular function | triphosphoribosyl-dephospho-CoA synthase activity | 10 |
| molecular function | T/G mismatch-specific endonuclease activity | 10 |
| molecular function | acetylornithine deacetylase activity | 10 |
| molecular function | DNA secondary structure binding | 10 |
| molecular function | exodeoxyribonuclease III activity | 10 |
| molecular function | scavenger receptor activity | 10 |
| molecular function | aspartate racemase activity | 10 |
| molecular function | valine-pyruvate transaminase activity | 10 |
| molecular function | N-acylglucosamine 2-epimerase activity | 10 |
| molecular function | transcriptional repressor activity, bacterial-type RNA polymerase core promoter proximal region sequence-specific binding | 10 |
| molecular function | rRNA (guanine-N7-)-methyltransferase activity | 10 |
| molecular function | pseudouridylate synthase activity | 10 |
| molecular function | trimethylamine-N-oxide reductase (cytochrome c) activity | 10 |
| molecular function | calcium:proton antiporter activity | 10 |
| molecular function | bicarbonate binding | 10 |
| molecular function | L-ascorbate 6-phosphate lactonase activity | 10 |
| molecular function | double-stranded DNA-dependent ATPase activity | 10 |
| molecular function | pyridoxine 5'-phosphate synthase activity | 10 |
| molecular function | acetate-CoA ligase activity | 10 |
| molecular function | glutamate dehydrogenase [NAD(P)+] activity | 10 |
| molecular function | lipopolysaccharide-1,6-galactosyltransferase activity | 10 |
| molecular function | o-succinylbenzoate-CoA ligase activity | 10 |
| molecular function | 3'-nucleotidase activity | 10 |
| molecular function | L-aspartate:fumarate oxidoreductase activity | 10 |
| molecular function | dihydropyrimidinase activity | 10 |
| molecular function | D,D-heptose 1,7-bisphosphate phosphatase activity | 10 |
| molecular function | methylaspartate ammonia-lyase activity | 10 |
| molecular function | oxidoreductase activity, acting on paired donors, with incorporation or reduction of molecular oxygen | 10 |
| molecular function | hydroxymethylpyrimidine kinase activity | 10 |
| molecular function | testosterone 6-beta-hydroxylase activity | 10 |
| molecular function | tRNA (uracil-2'-O-)-methyltransferase activity | 10 |
| molecular function | glucan 1,4-alpha-glucosidase activity | 10 |
| molecular function | uroporphyrinogen-III synthase activity | 10 |
| molecular function | diphosphate-fructose-6-phosphate 1-phosphotransferase activity | 10 |
| molecular function | adenosylcobinamide-GDP ribazoletransferase activity | 10 |
| molecular function | muscle alpha-actinin binding | 10 |
| molecular function | alkyl sulfatase activity | 10 |
| molecular function | malate dehydrogenase (decarboxylating) (NAD+) activity | 10 |
| molecular function | adenosylhomocysteinase activity | 10 |
| molecular function | histone binding | 10 |
| molecular function | mannitol-1-phosphate 5-dehydrogenase activity | 10 |
| molecular function | xylose isomerase activity | 10 |
| molecular function | RNA-dependent ATPase activity | 10 |
| molecular function | protein transmembrane transporter activity | 10 |
| molecular function | 6-pyruvoyltetrahydropterin synthase activity | 10 |
| molecular function | glutamate formimidoyltransferase activity | 10 |
| molecular function | choline dehydrogenase activity | 10 |
| molecular function | kinesin binding | 10 |
| molecular function | phospholipase activity | 10 |
| molecular function | L-tryptophan transmembrane transporter activity | 10 |
| molecular function | dCMP deaminase activity | 10 |
| molecular function | sucrose phosphorylase activity | 10 |
| molecular function | malate dehydrogenase (decarboxylating) (NADP+) activity | 10 |
| molecular function | sulfate adenylyltransferase (ATP) activity | 10 |
| molecular function | protein-glutamate methylesterase activity | 10 |
| molecular function | ribonuclease R activity | 9 |
| molecular function | intramolecular oxidoreductase activity, interconverting keto- and enol-groups | 9 |
| molecular function | gluconate 2-dehydrogenase (acceptor) activity | 9 |
| molecular function | serine transmembrane transporter activity | 9 |
| molecular function | inosine nucleosidase activity | 9 |
| molecular function | arylsulfatase activity | 9 |
| molecular function | N-acetylglucosamine kinase activity | 9 |
| molecular function | 6,7-dihydropteridine reductase activity | 9 |
| molecular function | chondroitin AC lyase activity | 9 |
| molecular function | rRNA (cytosine-C5-)-methyltransferase activity | 9 |
| molecular function | pseudouridine kinase activity | 9 |
| molecular function | gamma-glutamyltransferase activity | 9 |
| molecular function | oxidoreductase activity, acting on the CH-NH group of donors | 9 |
| molecular function | oxidoreductase activity, acting on peroxide as acceptor | 9 |
| molecular function | ornithine decarboxylase inhibitor activity | 9 |
| molecular function | electron-transferring-flavoprotein dehydrogenase activity | 9 |
| molecular function | selenate reductase activity | 9 |
| molecular function | aldehyde dehydrogenase (NADP+) activity | 9 |
| molecular function | cytidine transmembrane transporter activity | 9 |
| molecular function | glycerol-3-phosphate O-acyltransferase activity | 9 |
| molecular function | GABA-A receptor activity | 9 |
| molecular function | siderophore transporter activity | 9 |
| molecular function | cyclic-nucleotide phosphodiesterase activity | 9 |
| molecular function | oxalate decarboxylase activity | 9 |
| molecular function | cellulose synthase activity | 9 |
| molecular function | GTP-dependent protein binding | 9 |
| molecular function | potassium ion symporter activity | 9 |
| molecular function | PDZ domain binding | 9 |
| molecular function | formate dehydrogenase (cytochrome-c-553) activity | 9 |
| molecular function | extracellular ligand-gated ion channel activity | 9 |
| molecular function | polysaccharide transmembrane transporter activity | 9 |
| molecular function | 2,3-dihydroxybenzoate-serine ligase activity | 9 |
| molecular function | creatininase activity | 9 |
| molecular function | GTPase activator activity | 9 |
| molecular function | ATPase activity, coupled to movement of substances | 9 |
| molecular function | copper-exporting ATPase activity | 9 |
| molecular function | ferredoxin-NADP+ reductase activity | 9 |
| molecular function | potassium uptake transmembrane transporter activity | 9 |
| molecular function | 1-phosphatidylinositol binding | 9 |
| molecular function | thiamine binding | 9 |
| molecular function | chondroitin B lyase activity | 9 |
| molecular function | RNA helicase activity | 9 |
| molecular function | single-stranded DNA 3'-5' exodeoxyribonuclease activity | 9 |
| molecular function | altronate dehydratase activity | 9 |
| molecular function | beta-fructofuranosidase activity | 9 |
| molecular function | 1,6-alpha-L-fucosidase activity | 9 |
| molecular function | malate synthase activity | 9 |
| molecular function | nitrogenase activity | 9 |
| molecular function | cellulose synthase (UDP-forming) activity | 9 |
| molecular function | chromate reductase activity | 9 |
| molecular function | oxidoreductase activity, acting on the aldehyde or oxo group of donors, iron-sulfur protein as acceptor | 9 |
| molecular function | trans-2-decenoyl-acyl-carrier-protein isomerase activity | 9 |
| molecular function | 4-hydroxy-3-methylbut-2-en-1-yl diphosphate reductase activity | 9 |
| molecular function | urease activity | 9 |
| molecular function | ADP-glyceromanno-heptose 6-epimerase activity | 9 |
| molecular function | 3',5'-cyclic-AMP phosphodiesterase activity | 9 |
| molecular function | 4-hydroxythreonine-4-phosphate dehydrogenase activity | 9 |
| molecular function | hyaluronate lyase activity | 9 |
| molecular function | neutral amino acid transmembrane transporter activity | 9 |
| molecular function | glycogen (starch) synthase activity | 9 |
| molecular function | uridine transmembrane transporter activity | 9 |
| molecular function | phenylacetate-CoA ligase activity | 9 |
| molecular function | CDP reductase activity | 9 |
| molecular function | protein tyrosine/serine/threonine phosphatase activity | 9 |
| molecular function | DNA photolyase activity | 9 |
| molecular function | protein N-acetylglucosaminyltransferase activity | 9 |
| molecular function | single-stranded DNA-dependent ATP-dependent DNA helicase activity | 9 |
| molecular function | isopentenyl-diphosphate delta-isomerase activity | 9 |
| molecular function | lipopolysaccharide heptosyltransferase activity | 9 |
| molecular function | UMP kinase activity | 9 |
| molecular function | ligand-gated channel activity | 9 |
| molecular function | undecaprenyl-diphosphatase activity | 9 |
| molecular function | iron-chelate-transporting ATPase activity | 9 |
| molecular function | pyrimidine-specific mismatch base pair DNA N-glycosylase activity | 9 |
| molecular function | FMN reductase (NADPH) activity | 9 |
| molecular function | lipopolysaccharide 3-alpha-galactosyltransferase activity | 9 |
| molecular function | alcohol dehydrogenase [NAD(P)+] activity | 9 |
| molecular function | acetyl-CoA C-acyltransferase activity | 8 |
| molecular function | endodeoxyribonuclease activity, producing 5'-phosphomonoesters | 8 |
| molecular function | pyrimidine-5'-nucleotide nucleosidase activity | 8 |
| molecular function | translation factor activity, RNA binding | 8 |
| molecular function | 5-amino-6-(5-phosphoribitylamino)uracil phosphatase activity | 8 |
| molecular function | alanine-glyoxylate transaminase activity | 8 |
| molecular function | malonate-semialdehyde dehydrogenase (acetylating) activity | 8 |
| molecular function | organic acid binding | 8 |
| molecular function | putrescine binding | 8 |
| molecular function | ADP-sugar diphosphatase activity | 8 |
| molecular function | CDP-diacylglycerol-serine O-phosphatidyltransferase activity | 8 |
| molecular function | 2-dehydro-3-deoxy-D-gluconate 5-dehydrogenase activity | 8 |
| molecular function | 2-deoxy-D-gluconate 3-dehydrogenase activity | 8 |
| molecular function | nucleocytoplasmic transporter activity | 8 |
| molecular function | steroid dehydrogenase activity, acting on the CH-OH group of donors, NAD or NADP as acceptor | 8 |
| molecular function | quaternary ammonium group:proton symporter activity | 8 |
| molecular function | sulfite reductase (NADPH) activity | 8 |
| molecular function | 4-amino-4-deoxychorismate lyase activity | 8 |
| molecular function | xyloglucan 1,6-alpha-xylosidase activity | 8 |
| molecular function | cytosine transmembrane transporter activity | 8 |
| molecular function | heparin-sulfate lyase activity | 8 |
| molecular function | channel activity | 8 |
| molecular function | dATP binding | 8 |
| molecular function | hydroxymethylglutaryl-CoA reductase (NADPH) activity | 8 |
| molecular function | DNA topoisomerase (ATP-hydrolyzing) inhibitor activity | 8 |
| molecular function | sirohydrochlorin cobaltochelatase activity | 8 |
| molecular function | ATP diphosphatase activity | 8 |
| molecular function | 5'-3' DNA helicase activity | 8 |
| molecular function | lysine 2,3-aminomutase activity | 8 |
| molecular function | 2-oxoglutarate decarboxylase activity | 8 |
| molecular function | CoA-ligase activity | 8 |
| molecular function | phosphotransferase activity, carboxyl group as acceptor | 8 |
| molecular function | rRNA (guanine-N1-)-methyltransferase activity | 8 |
| molecular function | protein histidine kinase binding | 8 |
| molecular function | tRNA m6t6A37 methyltransferase activity | 8 |
| molecular function | ureidoglycolate dehydrogenase activity | 8 |
| molecular function | lipid-A-disaccharide synthase activity | 8 |
| molecular function | fucosidase activity | 8 |
| molecular function | diphosphomevalonate decarboxylase activity | 8 |
| molecular function | secondary active sulfate transmembrane transporter activity | 8 |
| molecular function | glutamate-1-semialdehyde 2,1-aminomutase activity | 8 |
| molecular function | cellulose binding | 8 |
| molecular function | oxidoreductase activity, acting on the CH-NH2 group of donors, NAD or NADP as acceptor | 8 |
| molecular function | L-tyrosine transmembrane transporter activity | 8 |
| molecular function | exoribonuclease activity, producing 5'-phosphomonoesters | 8 |
| molecular function | arginine transmembrane transporter activity | 8 |
| molecular function | bile acid:sodium symporter activity | 8 |
| molecular function | mercury (II) reductase activity | 8 |
| molecular function | 7S RNA binding | 8 |
| molecular function | lipopolysaccharide binding | 8 |
| molecular function | histone deacetylase binding | 8 |
| molecular function | IAA-Ala conjugate hydrolase activity | 8 |
| molecular function | oxaloacetate tautomerase activity | 8 |
| molecular function | delayed rectifier potassium channel activity | 8 |
| molecular function | carnitine dehydratase activity | 8 |
| molecular function | ribonuclease inhibitor activity | 8 |
| molecular function | L-serine hydro-lyase (adding indole, L-tryptophan-forming) activity | 8 |
| molecular function | glutathione-regulated potassium exporter activity | 8 |
| molecular function | L-phenylalanine transmembrane transporter activity | 8 |
| molecular function | oxidoreductase activity, oxidizing metal ions | 8 |
| molecular function | D-xylose-importing ATPase activity | 8 |
| molecular function | homoserine O-acetyltransferase activity | 8 |
| molecular function | glutathione synthase activity | 8 |
| molecular function | receptor binding | 8 |
| molecular function | choline-sulfatase activity | 8 |
| molecular function | endopeptidase inhibitor activity | 8 |
| molecular function | amylo-alpha-1,6-glucosidase activity | 8 |
| molecular function | nitrite efflux transmembrane transporter activity | 8 |
| molecular function | alpha-D-xyloside xylohydrolase | 8 |
| molecular function | nucleoside:sodium symporter activity | 8 |
| molecular function | glyceraldehyde-3-phosphate dehydrogenase (NADP+) (phosphorylating) activity | 8 |
| molecular function | 2-oxoglutarate synthase activity | 8 |
| molecular function | cholate 7-alpha-dehydrogenase activity | 8 |
| molecular function | host cell surface receptor binding | 8 |
| molecular function | 6-deoxy-6-sulfofructose-1-phosphate aldolase activity | 8 |
| molecular function | neutral amino acid:sodium symporter activity | 8 |
| molecular function | arginase activity | 7 |
| molecular function | diamine N-acetyltransferase activity | 7 |
| molecular function | 8-oxo-dGDP phosphatase activity | 7 |
| molecular function | trisaccharide binding | 7 |
| molecular function | GMP binding | 7 |
| molecular function | 3-hydroxyacyl-[acyl-carrier-protein] dehydratase activity | 7 |
| molecular function | NAD(P)+ transhydrogenase activity | 7 |
| molecular function | raffinose alpha-galactosidase activity | 7 |
| molecular function | xylan binding | 7 |
| molecular function | IAA-Phe conjugate hydrolase activity | 7 |
| molecular function | glycerol transmembrane transporter activity | 7 |
| molecular function | nucleobase transmembrane transporter activity | 7 |
| molecular function | NADPHX epimerase activity | 7 |
| molecular function | recombination hotspot binding | 7 |
| molecular function | RNA strand annealing activity | 7 |
| molecular function | N4-(beta-N-acetylglucosaminyl)-L-asparaginase activity | 7 |
| molecular function | phosphate ion transmembrane transporter activity | 7 |
| molecular function | citrate CoA-transferase activity | 7 |
| molecular function | cGMP binding | 7 |
| molecular function | dihydroorotate oxidase activity | 7 |
| molecular function | oxygen transporter activity | 7 |
| molecular function | UDP-2,3-diacylglucosamine hydrolase activity | 7 |
| molecular function | zinc transporting ATPase activity | 7 |
| molecular function | protein-phosphocysteine-D-fructose-phosphotransferase system transporter activity | 7 |
| molecular function | 2-dehydro-3-deoxygalactonokinase activity | 7 |
| molecular function | Ran GTPase binding | 7 |
| molecular function | carbon-carbon lyase activity | 7 |
| molecular function | oxidoreductase activity, acting on the CH-OH group of donors, quinone or similar compound as acceptor | 7 |
| molecular function | alpha-N-acetylglucosaminidase activity | 7 |
| molecular function | antisigma factor binding | 7 |
| molecular function | pectin lyase activity | 7 |
| molecular function | keratin filament binding | 7 |
| molecular function | carbon-nitrogen lyase activity | 7 |
| molecular function | NADHX epimerase activity | 7 |
| molecular function | histone methyltransferase activity (H3-K4 specific) | 7 |
| molecular function | methyl-CpG binding | 7 |
| molecular function | tricarboxylic acid transmembrane transporter activity | 7 |
| molecular function | 3-phenylpropionate dioxygenase activity | 7 |
| molecular function | ion channel binding | 7 |
| molecular function | anti-sigma factor antagonist activity | 7 |
| molecular function | Z-farnesyl diphosphate synthase activity | 7 |
| molecular function | rRNA (uridine-2'-O-)-methyltransferase activity | 7 |
| molecular function | single-stranded DNA 5'-3' exodeoxyribonuclease activity | 7 |
| molecular function | protein-transmembrane transporting ATPase activity | 7 |
| molecular function | glycine betaine-transporting ATPase activity | 7 |
| molecular function | galactose uniporter activity | 7 |
| molecular function | isocitrate lyase activity | 7 |
| molecular function | cell adhesion molecule binding | 7 |
| molecular function | ribonuclease E activity | 7 |
| molecular function | RNA pyrophosphohydrolase activity | 7 |
| molecular function | acetoin dehydrogenase activity | 7 |
| molecular function | organic anion transmembrane transporter activity | 7 |
| molecular function | 3-demethylubiquinone-9 3-O-methyltransferase activity | 7 |
| molecular function | ethanolamine ammonia-lyase activity | 7 |
| molecular function | nicotinamidase activity | 7 |
| molecular function | AMP nucleosidase activity | 7 |
| molecular function | phosphatidylinositol binding | 7 |
| molecular function | hydroxypyruvate isomerase activity | 7 |
| molecular function | IAA-Leu conjugate hydrolase activity | 7 |
| molecular function | cysteine-transporting ATPase activity | 7 |
| molecular function | urate transmembrane transporter activity | 7 |
| molecular function | adenine transmembrane transporter activity | 7 |
| molecular function | protein-glutamate O-methyltransferase activity | 7 |
| molecular function | 2-iminoacetate synthase activity | 7 |
| molecular function | ADP-dependent NAD(P)H-hydrate dehydratase activity | 7 |
| molecular function | G-protein coupled receptor activity | 7 |
| molecular function | protein-N(PI)-phosphohistidine-mannitol phosphotransferase system transmembrane transporter activity | 7 |
| molecular function | serine-pyruvate transaminase activity | 7 |
| molecular function | neopullulanase activity | 7 |
| molecular function | carnitine transmembrane transporter activity | 7 |
| molecular function | hydroxyacylglutathione hydrolase activity | 7 |
| molecular function | 3-hydroxypropionate dehydrogenase (NADP+) activity | 7 |
| molecular function | molybdate ion transmembrane transporter activity | 7 |
| molecular function | spectrin binding | 7 |
| molecular function | methylisocitrate lyase activity | 7 |
| molecular function | xenobiotic-transporting ATPase activity | 7 |
| molecular function | GTPase binding | 7 |
| molecular function | succinyl-diaminopimelate desuccinylase activity | 7 |
| molecular function | histone deacetylase activity | 7 |
| molecular function | heme oxygenase (decyclizing) activity | 7 |
| molecular function | transcription cofactor activity | 7 |
| molecular function | galacturonan binding | 7 |
| molecular function | trimethylamine-N-oxide reductase activity | 6 |
| molecular function | protein kinase B binding | 6 |
| molecular function | mannose-phosphate guanylyltransferase activity | 6 |
| molecular function | dodecenoyl-CoA delta-isomerase activity | 6 |
| molecular function | amine-lyase activity | 6 |
| molecular function | sulfotransferase activity | 6 |
| molecular function | glucose-1-phosphatase activity | 6 |
| molecular function | S100 protein binding | 6 |
| molecular function | cytochrome bo3 ubiquinol oxidase activity | 6 |
| molecular function | acetylxylan esterase activity | 6 |
| molecular function | bis(5'-nucleosyl)-tetraphosphatase activity | 6 |
| molecular function | purine nucleotide binding | 6 |
| molecular function | lactaldehyde dehydrogenase activity | 6 |
| molecular function | oxidoreduction-driven active transmembrane transporter activity | 6 |
| molecular function | glycolaldehyde dehydrogenase activity | 6 |
| molecular function | olfactory receptor activity | 6 |
| molecular function | 2-keto-3-deoxygluconate:proton symporter activity | 6 |
| molecular function | L-valine transaminase activity | 6 |
| molecular function | amino-acid betaine transmembrane transporter activity | 6 |
| molecular function | phosphorylase kinase activity | 6 |
| molecular function | 5'-deoxyribose-5-phosphate lyase activity | 6 |
| molecular function | nicotinamide phosphoribosyltransferase activity | 6 |
| molecular function | S-(hydroxymethyl)glutathione dehydrogenase activity | 6 |
| molecular function | dynein binding | 6 |
| molecular function | oxidoreductase activity, acting on paired donors, with incorporation or reduction of molecular oxygen, NAD(P)H as one donor, and incorporation of two atoms of oxygen into one donor | 6 |
| molecular function | threonine aldolase activity | 6 |
| molecular function | 6-carboxy-5,6,7,8-tetrahydropterin synthase activity | 6 |
| molecular function | collagen binding | 6 |
| molecular function | translation initiation factor binding | 6 |
| molecular function | Cys-tRNA(Pro) hydrolase activity | 6 |
| molecular function | GDP-L-fucose synthase activity | 6 |
| molecular function | carnitine-CoA ligase activity | 6 |
| molecular function | structure-specific DNA binding | 6 |
| molecular function | S-adenosylmethionine-dependent tRNA (m5U54) methyltransferase activity | 6 |
| molecular function | bis(5'-nucleosyl)-tetraphosphatase (asymmetrical) activity | 6 |
| molecular function | tartronate-semialdehyde synthase activity | 6 |
| molecular function | lactose:proton symporter activity | 6 |
| molecular function | protein-arginine deiminase activity | 6 |
| molecular function | primary amine oxidase activity | 6 |
| molecular function | allantoinase activity | 6 |
| molecular function | UTP:galactose-1-phosphate uridylyltransferase activity | 6 |
| molecular function | inositol-3-phosphate synthase activity | 6 |
| molecular function | sulfathiazole transmembrane transporter activity | 6 |
| molecular function | odorant binding | 6 |
| molecular function | nucleoside:proton symporter activity | 6 |
| molecular function | 4-hydroxybenzoate octaprenyltransferase activity | 6 |
| molecular function | oxidoreductase activity, acting on single donors with incorporation of molecular oxygen, incorporation of two atoms of oxygen | 6 |
| molecular function | poly(3-hydroxybutyrate) depolymerase activity | 6 |
| molecular function | tRNA (adenine-N6-)-methyltransferase activity | 6 |
| molecular function | fructose transmembrane transporter activity | 6 |
| molecular function | glucan endo-1,6-beta-glucosidase activity | 6 |
| molecular function | diphosphotransferase activity | 6 |
| molecular function | protein-glutamine N-methyltransferase activity | 6 |
| molecular function | racemase and epimerase activity, acting on amino acids and derivatives | 6 |
| molecular function | carbon-nitrogen ligase activity, with glutamine as amido-N-donor | 6 |
| molecular function | phosphatidylcholine 1-acylhydrolase activity | 6 |
| molecular function | pyruvate dehydrogenase (quinone) activity | 6 |
| molecular function | citrate transmembrane transporter activity | 6 |
| molecular function | small protein activating enzyme activity | 6 |
| molecular function | ferroxidase activity | 6 |
| molecular function | cation channel activity | 6 |
| molecular function | GTPase activating protein binding | 6 |
| molecular function | protein self-association | 6 |
| molecular function | molybdopterin synthase activity | 6 |
| molecular function | receptor signaling protein serine/threonine kinase activity | 6 |
| molecular function | 6-carboxyhexanoate-CoA ligase activity | 6 |
| molecular function | oxidoreductase activity, acting on hydrogen as donor | 6 |
| molecular function | trans-2-enoyl-CoA reductase (NADPH) activity | 6 |
| molecular function | alkali metal ion binding | 6 |
| molecular function | glutamate-putrescine ligase activity | 6 |
| molecular function | thiol-dependent ubiquitin-specific protease activity | 6 |
| molecular function | L-methionine:thioredoxin-disulfide S-oxidoreductase activity | 6 |
| molecular function | crotonobetaine-CoA ligase activity | 6 |
| molecular function | 2-methylcitrate dehydratase activity | 6 |
| molecular function | blood-group-substance endo-1,4-beta-galactosidase activity | 6 |
| molecular function | 1,3-propanediol dehydrogenase activity | 6 |
| molecular function | serine 3-dehydrogenase activity | 6 |
| molecular function | L-isoleucine transaminase activity | 6 |
| molecular function | 8-oxo-7,8-dihydrodeoxyguanosine triphosphate pyrophosphatase activity | 6 |
| molecular function | alpha,alpha-trehalose-phosphate synthase (UDP-forming) activity | 6 |
| molecular function | aminobutyraldehyde dehydrogenase activity | 6 |
| molecular function | lactose binding | 6 |
| molecular function | L-iditol 2-dehydrogenase activity | 6 |
| molecular function | L-leucine transaminase activity | 6 |
| molecular function | [isocitrate dehydrogenase (NADP+)] kinase activity | 6 |
| molecular function | triglyceride lipase activity | 6 |
| molecular function | ferredoxin-NAD+ reductase activity | 6 |
| molecular function | endoribonuclease inhibitor activity | 6 |
| molecular function | ferric iron transmembrane transporter activity | 6 |
| molecular function | tRNA(Ile)-lysidine synthase activity | 6 |
| molecular function | rRNA (cytosine-2'-O-)-methyltransferase activity | 6 |
| molecular function | NADH binding | 6 |
| molecular function | phosphatidate phosphatase activity | 6 |
| molecular function | protease binding | 6 |
| molecular function | cyclopropane-fatty-acyl-phospholipid synthase activity | 6 |
| molecular function | ubiquitin-like protein transferase activity | 6 |
| molecular function | (2,3-dihydroxybenzoyl)adenylate synthase activity | 6 |
| molecular function | proteoglycan binding | 6 |
| molecular function | guanyl-nucleotide exchange factor activity | 6 |
| molecular function | tRNA cytidylyltransferase activity | 6 |
| molecular function | protein anchor | 6 |
| molecular function | D(-)-tartrate dehydratase activity | 6 |
| molecular function | 2-polyprenyl-6-methoxy-1,4-benzoquinone methyltransferase activity | 5 |
| molecular function | peptidase activity, acting on L-amino acid peptides | 5 |
| molecular function | isocitrate dehydrogenase activity | 5 |
| molecular function | 16S rRNA (guanine(966)-N(2))-methyltransferase activity | 5 |
| molecular function | cyclic nucleotide binding | 5 |
| molecular function | shikimate transmembrane transporter activity | 5 |
| molecular function | guanyl ribonucleotide binding | 5 |
| molecular function | ubiquitin protein ligase binding | 5 |
| molecular function | D-arabinose 1-dehydrogenase [NAD(P)+] activity | 5 |
| molecular function | L-ribulose-phosphate 4-epimerase activity | 5 |
| molecular function | protein-phosphocysteine-mannosylglycerate-phosphotransferase system transporter activity | 5 |
| molecular function | spermidine binding | 5 |
| molecular function | 3-dehydro-L-gulonate 2-dehydrogenase activity | 5 |
| molecular function | L-lysine efflux transmembrane transporter activity | 5 |
| molecular function | 3-hydroxyoctanoyl-[acyl-carrier-protein] dehydratase activity | 5 |
| molecular function | N-acetyl-anhydromuramoyl-L-alanine amidase activity | 5 |
| molecular function | fucosyltransferase activity | 5 |
| molecular function | poly(A) binding | 5 |
| molecular function | glycoprotein binding | 5 |
| molecular function | D-glutaminase activity | 5 |
| molecular function | long-chain fatty acid transporting porin activity | 5 |
| molecular function | ubiquitin-ubiquitin ligase activity | 5 |
| molecular function | rhamnulose-1-phosphate aldolase activity | 5 |
| molecular function | ribonucleoside binding | 5 |
| molecular function | 3-dehydro-L-gulonate-6-phosphate decarboxylase activity | 5 |
| molecular function | drug transporter activity | 5 |
| molecular function | long-chain-alcohol O-fatty-acyltransferase activity | 5 |
| molecular function | cytoskeletal adaptor activity | 5 |
| molecular function | titin binding | 5 |
| molecular function | nicotinamide-nucleotide amidase activity | 5 |
| molecular function | quercetin 2,3-dioxygenase activity | 5 |
| molecular function | O-acetylhomoserine aminocarboxypropyltransferase activity | 5 |
| molecular function | trimethylamine methyltransferase activity | 5 |
| molecular function | epoxyqueuosine reductase activity | 5 |
| molecular function | nitrite reductase [NAD(P)H] activity | 5 |
| molecular function | bacterial-type RNA polymerase transcription factor activity, metal ion regulated sequence-specific DNA binding | 5 |
| molecular function | heptose 7-phosphate kinase activity | 5 |
| molecular function | 14-3-3 protein binding | 5 |
| molecular function | non-membrane spanning protein tyrosine phosphatase activity | 5 |
| molecular function | diacylglycerol O-acyltransferase activity | 5 |
| molecular function | phospholipase A2 activity | 5 |
| molecular function | protein-phosphocysteine-mannitol phosphotransferase system transporter activity | 5 |
| molecular function | oxidoreductase activity, acting on other nitrogenous compounds as donors, with NAD or NADP as acceptor | 5 |
| molecular function | oxidoreductase activity, acting on single donors with incorporation of molecular oxygen | 5 |
| molecular function | hexosaminidase activity | 5 |
| molecular function | cystathionine gamma-synthase activity | 5 |
| molecular function | dihydrouracil dehydrogenase (NAD+) activity | 5 |
| molecular function | 3-hydroxydecanoyl-[acyl-carrier-protein] dehydratase activity | 5 |
| molecular function | butyryl-CoA dehydrogenase activity | 5 |
| molecular function | carboxymethylenebutenolidase activity | 5 |
| molecular function | citrate (Re)-synthase activity | 5 |
| molecular function | oxidoreductase activity, acting on phosphorus or arsenic in donors | 5 |
| molecular function | exoribonuclease activity | 5 |
| molecular function | ATPase activator activity | 5 |
| molecular function | solute:cation symporter activity | 5 |
| molecular function | myosin light chain kinase activity | 5 |
| molecular function | inorganic anion transmembrane transporter activity | 5 |
| molecular function | propionate-CoA ligase activity | 5 |
| molecular function | heptose-1-phosphate adenylyltransferase activity | 5 |
| molecular function | lipopolysaccharide core heptosyltransferase activity | 5 |
| molecular function | 23S rRNA (guanine(2445)-N(2))-methyltransferase activity | 5 |
| molecular function | inositol hexakisphosphate binding | 5 |
| molecular function | anthranilate phosphoribosyltransferase activity | 5 |
| molecular function | 2-oxopent-4-enoate hydratase activity | 5 |
| molecular function | sn-glycerol-3-phosphate:ubiquinone oxidoreductase activity | 5 |
| molecular function | peptidase inhibitor activity | 5 |
| molecular function | virion binding | 5 |
| molecular function | oxygen-dependent protoporphyrinogen oxidase activity | 5 |
| molecular function | glutathione binding | 5 |
| molecular function | tellurite uptake transmembrane transporter activity | 5 |
| molecular function | 6-phosphogluconolactonase activity | 5 |
| molecular function | ferric-chelate reductase (NADPH) activity | 5 |
| molecular function | UDP-N-acetylmuramoylalanyl-D-glutamyl-2,6-diaminopimelate-D-alanyl-D-alanine ligase activity | 5 |
| molecular function | heme-transporting ATPase activity | 5 |
| molecular function | cytochrome o ubiquinol oxidase activity | 5 |
| molecular function | ATP-dependent DNA/RNA helicase activity | 5 |
| molecular function | biotin-protein ligase activity | 5 |
| molecular function | tRNA (uracil) methyltransferase activity | 5 |
| molecular function | beta-ketoacyl-acyl-carrier-protein synthase II activity | 5 |
| molecular function | acyl binding | 5 |
| molecular function | CDP-glucose 4,6-dehydratase activity | 5 |
| molecular function | tRNA N-acetyltransferase activity | 5 |
| molecular function | protein-lysine N-methyltransferase activity | 5 |
| molecular function | calcium ion transmembrane transporter activity | 5 |
| molecular function | Hsp70 protein binding | 5 |
| molecular function | oxidoreductase activity, acting on a sulfur group of donors | 5 |
| molecular function | endo-1,4-beta-xylanase activity | 5 |
| molecular function | xanthosine transmembrane transporter activity | 5 |
| molecular function | RNA polymerase II transcription factor activity, sequence-specific DNA binding | 5 |
| molecular function | 4-hydroxybutyrate CoA-transferase activity | 5 |
| molecular function | cinnamoyl-CoA:phenyllactate CoA-transferase activity | 5 |
| molecular function | purine nucleoside binding | 5 |
| molecular function | lysozyme inhibitor activity | 5 |
| molecular function | phospholipid-hydroperoxide glutathione peroxidase activity | 5 |
| molecular function | kinase inhibitor activity | 5 |
| molecular function | phosphoenolpyruvate carboxykinase activity | 5 |
| molecular function | endoribonuclease activity, producing 5'-phosphomonoesters | 5 |
| molecular function | kappa-carrageenase activity | 5 |
| molecular function | UDP-N-acetylmuramoyl-L-alanyl-D-glutamyl-meso-2,6-diaminopimelyl-D-alanyl-D-alanine:undecaprenyl-phosphate transferase activity | 5 |
| molecular function | UDP-N-acetylglucosamine 4-epimerase activity | 5 |
| molecular function | pectinesterase activity | 5 |
| molecular function | TBP-class protein binding | 5 |
| molecular function | polyphosphate-glucose phosphotransferase activity | 5 |
| molecular function | glycerate dehydrogenase activity | 5 |
| molecular function | phosphatidylinositol alpha-mannosyltransferase activity | 5 |
| molecular function | oxidoreductase activity, acting on a heme group of donors | 5 |
| molecular function | adenosine nucleosidase activity | 5 |
| molecular function | glutathione hydrolase activity | 5 |
| molecular function | 2,5-didehydrogluconate reductase activity | 5 |
| molecular function | catechol 1,2-dioxygenase activity | 5 |
| molecular function | short-chain carboxylesterase activity | 5 |
| molecular function | 5-carboxymethyl-2-hydroxymuconate delta-isomerase activity | 5 |
| molecular function | acetylesterase activity | 5 |
| molecular function | 1,4-dihydroxy-2-naphthoate octaprenyltransferase activity | 5 |
| molecular function | 1,2-diacylglycerol 3-beta-galactosyltransferase activity | 5 |
| molecular function | N-acylglucosamine-6-phosphate 2-epimerase activity | 5 |
| molecular function | malate dehydrogenase (quinone) activity | 5 |
| molecular function | lysophospholipid transporter activity | 4 |
| molecular function | dimethylallyltranstransferase activity | 4 |
| molecular function | exodeoxyribonuclease I activity | 4 |
| molecular function | pyrroloquinoline quinone binding | 4 |
| molecular function | kinase binding | 4 |
| molecular function | 2-succinyl-6-hydroxy-2,4-cyclohexadiene-1-carboxylate synthase activity | 4 |
| molecular function | Type IV site-specific deoxyribonuclease activity | 4 |
| molecular function | N-acetylglucosamine-6-sulfatase activity | 4 |
| molecular function | putrescine:ornithine antiporter activity | 4 |
| molecular function | intracellular cAMP activated cation channel activity | 4 |
| molecular function | phosphohistidine phosphatase activity | 4 |
| molecular function | L,L-diaminopimelate aminotransferase activity | 4 |
| molecular function | phosphatidylethanolamine:Kdo2-lipid A phosphoethanolamine transferase activity | 4 |
| molecular function | aldehyde dehydrogenase [NAD(P)+] activity | 4 |
| molecular function | nutrient reservoir activity | 4 |
| molecular function | phosphoenolpyruvate-glycerone phosphotransferase activity | 4 |
| molecular function | acyl-CoA ligase activity | 4 |
| molecular function | quaternary ammonium group binding | 4 |
| molecular function | L-glutamate transmembrane transporter activity | 4 |
| molecular function | thiosulfate dehydrogenase activity | 4 |
| molecular function | adenylylsulfate kinase activity | 4 |
| molecular function | molybdenum cofactor guanylyltransferase activity | 4 |
| molecular function | mannosyl-glycoprotein endo-beta-N-acetylglucosaminidase activity | 4 |
| molecular function | ribosylnicotinamide kinase activity | 4 |
| molecular function | pantothenate:sodium symporter activity | 4 |
| molecular function | L-arabinose transmembrane transporter activity | 4 |
| molecular function | stearoyl-CoA 9-desaturase activity | 4 |
| molecular function | beta-amyloid binding | 4 |
| molecular function | hydrolase activity, acting on acid carbon-carbon bonds, in ketonic substances | 4 |
| molecular function | adenyl nucleotide binding | 4 |
| molecular function | arsenite secondary active transmembrane transporter activity | 4 |
| molecular function | Rho GTPase binding | 4 |
| molecular function | urea channel activity | 4 |
| molecular function | adenine/cytosine mispair binding | 4 |
| molecular function | NADPH:quinone reductase activity | 4 |
| molecular function | amine transmembrane transporter activity | 4 |
| molecular function | succinylglutamate-semialdehyde dehydrogenase activity | 4 |
| molecular function | galactarate dehydratase activity | 4 |
| molecular function | nitrite transmembrane transporter activity | 4 |
| molecular function | Hsp90 protein binding | 4 |
| molecular function | aralkylamine N-acetyltransferase activity | 4 |
| molecular function | nucleoside-diphosphatase activity | 4 |
| molecular function | L-ascorbic acid binding | 4 |
| molecular function | 3-hydroxybutyrate dehydrogenase activity | 4 |
| molecular function | deoxyribodipyrimidine photo-lyase activity | 4 |
| molecular function | translation repressor activity, nucleic acid binding | 4 |
| molecular function | polygalacturonase activity | 4 |
| molecular function | hydrolase activity, acting on carbon-nitrogen (but not peptide) bonds, in cyclic amidines | 4 |
| molecular function | CTP:3'-cytidine-tRNA cytidylyltransferase activity | 4 |
| molecular function | 2-dehydro-3-deoxy-D-pentonate aldolase activity | 4 |
| molecular function | ureidoglycine aminohydrolase activity | 4 |
| molecular function | pyridoxamine-phosphate oxidase activity | 4 |
| molecular function | oxidoreductase activity, acting on CH or CH2 groups, NAD or NADP as acceptor | 4 |
| molecular function | tubulin binding | 4 |
| molecular function | glycerol-3-phosphate dehydrogenase [NADP+] activity | 4 |
| molecular function | tagaturonate reductase activity | 4 |
| molecular function | oxidoreductase activity, acting on the CH-NH2 group of donors | 4 |
| molecular function | D-serine transmembrane transporter activity | 4 |
| molecular function | homocitrate synthase activity | 4 |
| molecular function | Rab GTPase binding | 4 |
| molecular function | protein-L-isoaspartate (D-aspartate) O-methyltransferase activity | 4 |
| molecular function | N-acylneuraminate-9-phosphate synthase activity | 4 |
| molecular function | ether hydrolase activity | 4 |
| molecular function | glycerophosphoinositol glycerophosphodiesterase activity | 4 |
| molecular function | allulose 6-phosphate 3-epimerase activity | 4 |
| molecular function | phosphatidylglycerol-phosphatidylethanolamine phosphatidyltransferase activity | 4 |
| molecular function | calcium:sodium antiporter activity | 4 |
| molecular function | antimonite transmembrane transporter activity | 4 |
| molecular function | L-fuculose-phosphate aldolase activity | 4 |
| molecular function | spermidine transmembrane transporter activity | 4 |
| molecular function | propionate kinase activity | 4 |
| molecular function | protein complex scaffold | 4 |
| molecular function | Roundabout binding | 4 |
| molecular function | maltose binding | 4 |
| molecular function | RNA-3'-phosphate cyclase activity | 4 |
| molecular function | carbon monoxide binding | 4 |
| molecular function | O-acyltransferase activity | 4 |
| molecular function | glucuronosyltransferase activity | 4 |
| molecular function | 3-(hydroxyamino)phenol mutase activity | 4 |
| molecular function | mechanically gated channel activity | 4 |
| molecular function | transcription antitermination factor activity, DNA binding | 4 |
| molecular function | aryldialkylphosphatase activity | 4 |
| molecular function | glycolipid 6-alpha-mannosyltransferase activity | 4 |
| molecular function | organic phosphonate transmembrane transporter activity | 4 |
| molecular function | translation repressor activity | 4 |
| molecular function | tartrate dehydrogenase activity | 4 |
| molecular function | energy transducer activity | 4 |
| molecular function | long-chain fatty acid transporter activity | 4 |
| molecular function | protein-phosphocysteine-glucose phosphotransferase system transporter activity | 4 |
| molecular function | 23S rRNA (adenine(2030)-N(6))-methyltransferase activity | 4 |
| molecular function | K63-linked polyubiquitin binding | 4 |
| molecular function | isovaleryl-CoA dehydrogenase activity | 4 |
| molecular function | 2-succinyl-5-enolpyruvyl-6-hydroxy-3-cyclohexene-1-carboxylic-acid synthase activity | 4 |
| molecular function | ATP:3'-cytidine-cytidine-tRNA adenylyltransferase activity | 4 |
| molecular function | S-formylglutathione hydrolase activity | 4 |
| molecular function | phosphofructokinase activity | 4 |
| molecular function | quinoprotein glucose dehydrogenase activity | 4 |
| molecular function | nitronate monooxygenase activity | 4 |
| molecular function | oxidoreductase activity, acting on CH or CH2 groups, with an iron-sulfur protein as acceptor | 4 |
| molecular function | RNA polymerase II core promoter proximal region sequence-specific DNA binding | 4 |
| molecular function | deoxyribonuclease I activity | 4 |
| molecular function | glucose-1-phosphate cytidylyltransferase activity | 4 |
| molecular function | mannose isomerase activity | 4 |
| molecular function | long-chain fatty acid [acyl-carrier-protein] ligase activity | 4 |
| molecular function | [acyl-carrier-protein] phosphodiesterase activity | 4 |
| molecular function | uniporter activity | 4 |
| molecular function | imidazolonepropionase activity | 4 |
| molecular function | guanylyltransferase activity | 4 |
| molecular function | DNA binding, bending | 4 |
| molecular function | 4-alpha-hydroxytetrahydrobiopterin dehydratase activity | 4 |
| molecular function | acyl-[acyl-carrier-protein]-phospholipid O-acyltransferase activity | 4 |
| molecular function | glycerophosphocholine phosphodiesterase activity | 4 |
| molecular function | cytochrome-c peroxidase activity | 4 |
| molecular function | diacylglycerol binding | 4 |
| molecular function | mannosidase activity | 4 |
| molecular function | CDP-glycerol glycerophosphotransferase activity | 4 |
| molecular function | L-allo-threonine aldolase activity | 4 |
| molecular function | choline binding | 4 |
| molecular function | oxidoreductase activity, acting on other nitrogenous compounds as donors | 4 |
| molecular function | sulfoquinovose isomerase activity | 4 |
| molecular function | trans-hexaprenyltranstransferase activity | 4 |
| molecular function | hydroxymethylglutaryl-CoA lyase activity | 4 |
| molecular function | formimidoylglutamase activity | 4 |
| molecular function | intracellular cGMP activated cation channel activity | 4 |
| molecular function | peptidoglycan peptide transporter activity | 4 |
| molecular function | ammonia-lyase activity | 4 |
| molecular function | 8-oxo-GDP phosphatase activity | 4 |
| molecular function | guanylate cyclase activity | 4 |
| molecular function | mannitol dehydrogenase activity | 4 |
| molecular function | protein complex binding | 4 |
| molecular function | N-succinylarginine dihydrolase activity | 4 |
| molecular function | CTP binding | 4 |
| molecular function | leucyltransferase activity | 4 |
| molecular function | nitrite reductase (NO-forming) activity | 4 |
| molecular function | D-erythro-sphingosine kinase activity | 4 |
| molecular function | ribonucleoprotein complex binding | 4 |
| molecular function | tryptophanase activity | 4 |
| molecular function | core promoter sequence-specific DNA binding | 4 |
| molecular function | fumarylacetoacetase activity | 4 |
| molecular function | UDP-sulfoquinovose:DAG sulfoquinovosyltransferase activity | 4 |
| molecular function | L-arabinose isomerase activity | 4 |
| molecular function | CTP:tRNA cytidylyltransferase activity | 4 |
| molecular function | muconate cycloisomerase activity | 4 |
| molecular function | protein kinase regulator activity | 4 |
| molecular function | 4-hydroxy-2-oxovalerate aldolase activity | 4 |
| molecular function | glutathione disulfide oxidoreductase activity | 4 |
| molecular function | calcium-dependent protein serine/threonine kinase activity | 4 |
| molecular function | RNA polymerase II core binding | 4 |
| molecular function | peptide-methionine (R)-S-oxide reductase activity | 4 |
| molecular function | exonuclease activity, active with either ribo- or deoxyribonucleic acids and producing 3'-phosphomonoesters | 4 |
| molecular function | tRNA (guanosine-2'-O-)-methyltransferase activity | 4 |
| molecular function | pyruvate, water dikinase activity | 4 |
| molecular function | maltose transporting porin activity | 4 |
| molecular function | NADPH-hemoprotein reductase activity | 4 |
| molecular function | bacterial-type RNA polymerase regulatory region DNA binding | 4 |
| molecular function | retroviral 3' processing activity | 4 |
| molecular function | N-hydroxyarylamine O-acetyltransferase activity | 4 |
| molecular function | angiostatin binding | 4 |
| molecular function | aromatic-amino-acid:2-oxoglutarate aminotransferase activity | 4 |
| molecular function | copper uptake transmembrane transporter activity | 4 |
| molecular function | ribonucleotide binding | 4 |
| molecular function | calmodulin-dependent protein kinase activity | 4 |
| molecular function | 3-carboxyethylcatechol 2,3-dioxygenase activity | 4 |
| molecular function | phosphogluconate dehydratase activity | 4 |
| molecular function | arylesterase activity | 4 |
| molecular function | preQ1 synthase activity | 3 |
| molecular function | pyrimidine nucleoside transmembrane transporter activity | 3 |
| molecular function | pectinesterase inhibitor activity | 3 |
| molecular function | phosphatidylglycerol phospholipase C activity | 3 |
| molecular function | stizolobate synthase activity | 3 |
| molecular function | spermine synthase activity | 3 |
| molecular function | arsenate reductase activity | 3 |
| molecular function | diphthine-ammonia ligase activity | 3 |
| molecular function | beta-1,4-mannosylglycoprotein 4-beta-N-acetylglucosaminyltransferase activity | 3 |
| molecular function | dCTP deaminase (dUMP-forming) activity | 3 |
| molecular function | trihydroxyferuloyl spermidine:S-adenosyl-L-methionine O-methyltransferase activity | 3 |
| molecular function | 3-oxoadipate enol-lactonase activity | 3 |
| molecular function | glycerol-1-phosphatase activity | 3 |
| molecular function | teichoic acid transmembrane transporter activity | 3 |
| molecular function | lysine:cadaverine antiporter activity | 3 |
| molecular function | uridylyltransferase activity | 3 |
| molecular function | O-palmitoyltransferase activity | 3 |
| molecular function | guanine transmembrane transporter activity | 3 |
| molecular function | 3-deoxy-manno-octulosonate-8-phosphatase activity | 3 |
| molecular function | palmitoyl-CoA hydrolase activity | 3 |
| molecular function | iron-responsive element binding | 3 |
| molecular function | L-histidine transmembrane transporter activity | 3 |
| molecular function | glucose-6-phosphate dehydrogenase activity | 3 |
| molecular function | 2-dehydro-3-deoxy-6-phosphogalactonate aldolase activity | 3 |
| molecular function | aspartate-prephenate aminotransferase activity | 3 |
| molecular function | guanosine-5'-triphosphate,3'-diphosphate diphosphatase activity | 3 |
| molecular function | lipopolysaccharide transmembrane transporter activity | 3 |
| molecular function | bacterial-type RNA polymerase transcriptional activator activity, sequence-specific DNA binding | 3 |
| molecular function | O antigen ligase activity | 3 |
| molecular function | very-long-chain-(S)-2-hydroxy-acid oxidase activity | 3 |
| molecular function | H3 histone acetyltransferase activity | 3 |
| molecular function | 3-hydroxypropionyl-CoA dehydratase activity | 3 |
| molecular function | neurexin family protein binding | 3 |
| molecular function | protein-succinyllysine desuccinylase activity | 3 |
| molecular function | N-carbamoyl-L-amino-acid hydrolase activity | 3 |
| molecular function | calcium-dependent phospholipid binding | 3 |
| molecular function | gluconokinase activity | 3 |
| molecular function | unmethylated CpG binding | 3 |
| molecular function | citryl-CoA lyase activity | 3 |
| molecular function | nicotinamide riboside hydrolase activity | 3 |
| molecular function | ureidoglycolate hydrolase activity | 3 |
| molecular function | trichloro-p-hydroquinone reductive dehalogenase activity | 3 |
| molecular function | L-idonate transmembrane transporter activity | 3 |
| molecular function | carnitine O-acetyltransferase activity | 3 |
| molecular function | chondroitin-sulfate-ABC exolyase activity | 3 |
| molecular function | oxidoreductase activity, acting on paired donors, with incorporation or reduction of molecular oxygen, 2-oxoglutarate as one donor, and incorporation of one atom each of oxygen into both donors | 3 |
| molecular function | caffeoyl-CoA O-methyltransferase activity | 3 |
| molecular function | allantoate deiminase activity | 3 |
| molecular function | rhodopsin kinase activity | 3 |
| molecular function | ureidoglycolate lyase activity | 3 |
| molecular function | glutamate-prephenate aminotransferase activity | 3 |
| molecular function | allantoin uptake transmembrane transporter activity | 3 |
| molecular function | cysteine-glucosaminylinositol ligase activity | 3 |
| molecular function | androstan-3-alpha,17-beta-diol dehydrogenase activity | 3 |
| molecular function | fatty acid synthase activity | 3 |
| molecular function | 1-pyrroline dehydrogenase activity | 3 |
| molecular function | amidinotransferase activity | 3 |
| molecular function | glutamate binding | 3 |
| molecular function | steryl-sulfatase activity | 3 |
| molecular function | outward rectifier potassium channel activity | 3 |
| molecular function | G-protein coupled receptor kinase activity | 3 |
| molecular function | 3-oxoacid CoA-transferase activity | 3 |
| molecular function | ribose-5-phosphate adenylyltransferase activity | 3 |
| molecular function | chitin deacetylase activity | 3 |
| molecular function | sarcosine oxidase activity | 3 |
| molecular function | intracellular cyclic nucleotide activated cation channel activity | 3 |
| molecular function | inositol monophosphate 4-phosphatase activity | 3 |
| molecular function | myosin binding | 3 |
| molecular function | urea transmembrane transporter activity | 3 |
| molecular function | D-xylulose reductase activity | 3 |
| molecular function | 2-hydroxyglutarate synthase activity | 3 |
| molecular function | phenylacetaldehyde dehydrogenase activity | 3 |
| molecular function | acylglycerol lipase activity | 3 |
| molecular function | L-rhamnose isomerase activity | 3 |
| molecular function | phosphatidylinositol-3-phosphate binding | 3 |
| molecular function | galactitol transmembrane transporter activity | 3 |
| molecular function | hexose transmembrane transporter activity | 3 |
| molecular function | 2-hydroxy-6-oxonona-2,4,7-trienedioate hydrolase activity | 3 |
| molecular function | ammeline aminohydrolase activity | 3 |
| molecular function | 2-octaprenyl-6-methoxyphenol hydroxylase activity | 3 |
| molecular function | 4-trimethylammoniobutyraldehyde dehydrogenase activity | 3 |
| molecular function | maltodextrin transmembrane transporter activity | 3 |
| molecular function | 3-oxoadipate CoA-transferase activity | 3 |
| molecular function | glutaryl-CoA dehydrogenase activity | 3 |
| molecular function | glycolipid transporter activity | 3 |
| molecular function | phenyllactate dehydrogenase activity | 3 |
| molecular function | cadaverine aminopropyltransferase activity | 3 |
| molecular function | DNA/RNA hybrid binding | 3 |
| molecular function | 6-deoxy-6-sulfofructose kinase activity | 3 |
| molecular function | ceramidase activity | 3 |
| molecular function | peptidyl-lysine N-acetyltransferase activity, acting on acetyl phosphate as donor | 3 |
| molecular function | lithium ion binding | 3 |
| molecular function | trehalose-phosphatase activity | 3 |
| molecular function | endochitinase activity | 3 |
| molecular function | URM1 activating enzyme activity | 3 |
| molecular function | macrolide binding | 3 |
| molecular function | fucose transmembrane transporter activity | 3 |
| molecular function | RNA strand-exchange activity | 3 |
| molecular function | aryl-alcohol dehydrogenase (NADP+) activity | 3 |
| molecular function | homoserine transmembrane transporter activity | 3 |
| molecular function | CTPase activity | 3 |
| molecular function | laminin binding | 3 |
| molecular function | organophosphate:inorganic phosphate antiporter activity | 3 |
| molecular function | thiopurine S-methyltransferase activity | 3 |
| molecular function | hippurate hydrolase activity | 3 |
| molecular function | nicotinic acid riboside hydrolase activity | 3 |
| molecular function | cyclic nucleotide-gated ion channel activity | 3 |
| molecular function | 2-keto-3-deoxygluconate aldolase activity | 3 |
| molecular function | formyl-CoA transferase activity | 3 |
| molecular function | lipopolysaccharide N-acetylmannosaminouronosyltransferase activity | 3 |
| molecular function | DNA/RNA hybrid annealing activity | 3 |
| molecular function | long-chain-(S)-2-hydroxy-long-chain-acid oxidase activity | 3 |
| molecular function | S-ribosylhomocysteine lyase activity | 3 |
| molecular function | sugar efflux transmembrane transporter activity | 3 |
| molecular function | 2-hydroxy-6-oxonona-2,4-dienedioate hydrolase activity | 3 |
| molecular function | alpha-L-rhamnosidase activity | 3 |
| molecular function | octanoyltransferase activity | 3 |
| molecular function | protein ADP-ribosylase activity | 3 |
| molecular function | succinyldiaminopimelate transaminase activity | 3 |
| molecular function | RNA polymerase II repressing transcription factor binding | 3 |
| molecular function | sorbitol-6-phosphate 2-dehydrogenase activity | 3 |
| molecular function | sulfate binding | 3 |
| molecular function | protein-malonyllysine demalonylase activity | 3 |
| molecular function | medium-chain-(S)-2-hydroxy-acid oxidase activity | 3 |
| molecular function | transferase activity, transferring selenium-containing groups | 3 |
| molecular function | phosphoric triester hydrolase activity | 3 |
| molecular function | kynurenine-oxoglutarate transaminase activity | 3 |
| molecular function | oxalyl-CoA decarboxylase activity | 3 |
| molecular function | erythrose-4-phosphate dehydrogenase activity | 3 |
| molecular function | arabinosyltransferase activity | 3 |
| molecular function | molybdenum ion transmembrane transporter activity | 3 |
| molecular function | methionine-oxo-acid transaminase activity | 3 |
| molecular function | hydrogen:quinone oxidoreductase activity | 3 |
| molecular function | alpha-ketoacid dehydrogenase activity | 3 |
| molecular function | Type II site-specific deoxyribonuclease activity | 3 |
| molecular function | nucleic acid binding transcription factor activity | 3 |
| molecular function | non-membrane spanning protein tyrosine kinase activity | 3 |
| molecular function | L-proline transmembrane transporter activity | 3 |
| molecular function | cobaltochelatase activity | 3 |
| molecular function | N-acylmannosamine kinase activity | 3 |
| molecular function | oxidoreductase activity, acting on iron-sulfur proteins as donors, NAD or NADP as acceptor | 3 |
| molecular function | thiamine-transporting ATPase activity | 3 |
| molecular function | myo-inosose-2 dehydratase activity | 3 |
| molecular function | high-affinity glutamate transmembrane transporter activity | 3 |
| molecular function | 2-ketobutyrate formate-lyase activity | 3 |
| molecular function | ribonuclease G activity | 3 |
| molecular function | serine binding | 3 |
| molecular function | trehalase activity | 3 |
| molecular function | voltage-gated ion channel activity | 3 |
| molecular function | 16S rRNA (guanine(1516)-N(2))-methyltransferase activity | 3 |
| molecular function | phenol 2-monooxygenase activity | 3 |
| molecular function | NAD+ nucleosidase activity | 3 |
| molecular function | rubredoxin-NAD+ reductase activity | 3 |
| molecular function | tropomyosin binding | 3 |
| molecular function | guanosine phosphorylase activity | 3 |
| molecular function | membrane insertase activity | 3 |
| molecular function | UDP-glucuronate decarboxylase activity | 3 |
| molecular function | L-leucine:2-oxoglutarate aminotransferase activity | 3 |
| molecular function | 4-hydroxyphenylpyruvate dioxygenase activity | 3 |
| molecular function | glycerol-2-phosphatase activity | 3 |
| molecular function | 4-hydroxyphenylacetate,NADH:oxygen oxidoreductase (3-hydroxylating) activity | 3 |
| molecular function | thiamine-pyrophosphatase activity | 3 |
| molecular function | lipopolysaccharide glucosyltransferase II activity | 3 |
| molecular function | mercury ion transmembrane transporter activity | 3 |
| molecular function | thermospermine synthase activity | 3 |
| molecular function | alpha,alpha-trehalose phosphorylase activity | 3 |
| molecular function | virus receptor activity | 3 |
| molecular function | 2-aminoethylphosphonate-pyruvate transaminase activity | 3 |
| molecular function | trihydroxyferuloyl spermidine O-methyltransferase activity | 3 |
| molecular function | L-ascorbate peroxidase activity | 3 |
| molecular function | phosphoadenylyl-sulfate reductase (thioredoxin) activity | 3 |
| molecular function | 23S rRNA (adenine(1618)-N(6))-methyltransferase activity | 3 |
| molecular function | fibronectin binding | 3 |
| molecular function | m7G(5')pppN diphosphatase activity | 3 |
| molecular function | dextransucrase activity | 3 |
| molecular function | UDP-N-acetylglucosamine-lysosomal-enzyme N-acetylglucosaminephosphotransferase activity | 3 |
| molecular function | acetyl-CoA:oxalate CoA-transferase | 3 |
| molecular function | starch alpha-glucosidase activity | 3 |
| molecular function | bacterial-type RNA polymerase regulatory region sequence-specific DNA binding | 3 |
| molecular function | 5-aminolevulinate synthase activity | 3 |
| molecular function | fatty-acyl-CoA synthase activity | 3 |
| molecular function | tRNA-(2-methylthio-N-6-(cis-hydroxy)isopentenyl adenosine)-hydroxylase activity | 3 |
| molecular function | glycolate dehydrogenase activity | 3 |
| molecular function | choline:sodium symporter activity | 3 |
| molecular function | alcohol oxidase activity | 3 |
| molecular function | high-affinity sulfate:proton symporter activity | 3 |
| molecular function | 2-aminoadipate transaminase activity | 3 |
| molecular function | 2'-phosphotransferase activity | 3 |
| molecular function | tRNA 2-selenouridine synthase activity | 3 |
| molecular function | tricaffeoyl spermidine:S-adenosyl-L-methionine O-methyltransferase activity | 3 |
| molecular function | protein serine/threonine/tyrosine kinase activity | 3 |
| molecular function | antimonite secondary active transmembrane transporter activity | 3 |
| molecular function | 2-octaprenyl-3-methyl-5-hydroxy-6-methoxy-1,4-benzoquinone methyltransferase activity | 3 |
| molecular function | galactonate dehydratase activity | 3 |
| molecular function | acharan sulfate lyase activity | 3 |
| molecular function | inositol monophosphate 3-phosphatase activity | 3 |
| molecular function | glucose-6-phosphatase activity | 3 |
| molecular function | transferase activity, transferring aldehyde or ketonic groups | 3 |
| molecular function | rRNA (adenine-N6-)-methyltransferase activity | 3 |
| molecular function | NAD+ ADP-ribosyltransferase activity | 3 |
| molecular function | 1-phosphatidylinositol-4-phosphate 5-kinase activity | 3 |
| molecular function | selenium binding | 3 |
| molecular function | RNA polymerase II regulatory region sequence-specific DNA binding | 3 |
| molecular function | hyaluronic acid binding | 3 |
| molecular function | ribose 1,5-bisphosphate phosphokinase activity | 3 |
| molecular function | S-nitrosoglutathione reductase activity | 3 |
| molecular function | cephalosporin-C deacetylase activity | 3 |
| molecular function | cytokine activity | 3 |
| molecular function | caffeoyl CoA:S-adenosyl-L-methionine O-methyltransferase activity | 3 |
| molecular function | pyridoxal 5'-phosphate synthase (glutamine hydrolysing) activity | 3 |
| molecular function | triokinase activity | 2 |
| molecular function | L-methionine-(S)-S-oxide reductase activity | 2 |
| molecular function | active transmembrane transporter activity | 2 |
| molecular function | intramolecular lyase activity | 2 |
| molecular function | fructuronate reductase activity | 2 |
| molecular function | dihydroneopterin triphosphate pyrophosphohydrolase activity | 2 |
| molecular function | high-affinity tryptophan transmembrane transporter activity | 2 |
| molecular function | lipopolysaccharide N-acetylglucosaminyltransferase activity | 2 |
| molecular function | organic phosphonate transmembrane-transporting ATPase activity | 2 |
| molecular function | aquacobalamin reductase activity | 2 |
| molecular function | gentisate 1,2-dioxygenase activity | 2 |
| molecular function | D-malate dehydrogenase (decarboxylating) activity | 2 |
| molecular function | taurine-transporting ATPase activity | 2 |
| molecular function | protoheme IX farnesyltransferase activity | 2 |
| molecular function | phenylalanine 4-monooxygenase activity | 2 |
| molecular function | IMP 5'-nucleotidase activity | 2 |
| molecular function | 4-hydroxybutyrate dehydrogenase activity | 2 |
| molecular function | GTPase regulator activity | 2 |
| molecular function | 2,3-dihydroxy-2,3-dihydro-phenylpropionate dehydrogenase activity | 2 |
| molecular function | (S)-2-hydroxy-acid oxidase activity | 2 |
| molecular function | 2-pyrone-4,6-dicarboxylate lactonase activity | 2 |
| molecular function | 2'-5'-RNA ligase activity | 2 |
| molecular function | arylamine N-acetyltransferase activity | 2 |
| molecular function | FAD transmembrane transporter activity | 2 |
| molecular function | beta-1,3-galactosyltransferase activity | 2 |
| molecular function | tripeptidase activity | 2 |
| molecular function | estradiol 17-beta-dehydrogenase activity | 2 |
| molecular function | coenzyme F390-G hydrolase activity | 2 |
| molecular function | carbon dioxide binding | 2 |
| molecular function | phosphotransferase activity, nitrogenous group as acceptor | 2 |
| molecular function | profilin binding | 2 |
| molecular function | ATP adenylyltransferase activity | 2 |
| molecular function | dihydroneopterin triphosphate 2'-epimerase activity | 2 |
| molecular function | pyridoxal phosphatase activity | 2 |
| molecular function | NADPH dehydrogenase (quinone) activity | 2 |
| molecular function | ligand-dependent nuclear receptor binding | 2 |
| molecular function | glycine transmembrane transporter activity | 2 |
| molecular function | thyroid hormone transmembrane transporter activity | 2 |
| molecular function | tetrachloro-p-hydroquinone reductive dehalogenase activity | 2 |
| molecular function | galactitol-1-phosphate 5-dehydrogenase activity | 2 |
| molecular function | protein-glutamine gamma-glutamyltransferase activity | 2 |
| molecular function | sterol-transporting ATPase activity | 2 |
| molecular function | 3-phytase activity | 2 |
| molecular function | protein phosphatase type 1 regulator activity | 2 |
| molecular function | histone-glutamine methyltransferase activity | 2 |
| molecular function | 1,4-dihydroxy-2-naphthoyl-CoA thioesterase activity | 2 |
| molecular function | diacylglycerol diphosphate phosphatase activity | 2 |
| molecular function | 3-sulfolactaldehyde reductase activity | 2 |
| molecular function | UDP-sugar diphosphatase activity | 2 |
| molecular function | calcium-dependent protein binding | 2 |
| molecular function | inosine kinase activity | 2 |
| molecular function | nucleoside-specific channel forming porin activity | 2 |
| molecular function | urea binding | 2 |
| molecular function | microtubule-severing ATPase activity | 2 |
| molecular function | fructose-6-phosphate binding | 2 |
| molecular function | antigen binding | 2 |
| molecular function | fructose 5-dehydrogenase activity | 2 |
| molecular function | exoribonuclease II activity | 2 |
| molecular function | indoleacetamide hydrolase activity | 2 |
| molecular function | guanine phosphoribosyltransferase activity | 2 |
| molecular function | hexokinase activity | 2 |
| molecular function | cation efflux transmembrane transporter activity | 2 |
| molecular function | acid-ammonia (or amide) ligase activity | 2 |
| molecular function | RNA ligase (ATP) activity | 2 |
| molecular function | molybdopterin-synthase adenylyltransferase activity | 2 |
| molecular function | 5-(carboxyamino)imidazole ribonucleotide mutase activity | 2 |
| molecular function | butane-1,4-diamine:2-oxoglutarate aminotransferase activity | 2 |
| molecular function | nucleotide phosphatase activity, acting on free nucleotides | 2 |
| molecular function | N-ethylmaleimide reductase activity | 2 |
| molecular function | 4-coumarate-CoA ligase activity | 2 |
| molecular function | acetylpyruvate hydrolase activity | 2 |
| molecular function | histone acetyltransferase binding | 2 |
| molecular function | tRNA-specific adenosine deaminase activity | 2 |
| molecular function | protein kinase inhibitor activity | 2 |
| molecular function | (S)-3-amino-2-methylpropionate transaminase activity | 2 |
| molecular function | 5-oxopent-3-ene-1,2,5-tricarboxylate decarboxylase activity | 2 |
| molecular function | abscisic acid binding | 2 |
| molecular function | aerobactin synthase activity | 2 |
| molecular function | galactosidase activity | 2 |
| molecular function | peptidyl-dipeptidase activity | 2 |
| molecular function | DNA (cytosine-5-)-methyltransferase activity, acting on CpG substrates | 2 |
| molecular function | lipoyl(octanoyl) transferase activity | 2 |
| molecular function | nucleoside triphosphate adenylate kinase activity | 2 |
| molecular function | 2-deoxyglucose-6-phosphatase activity | 2 |
| molecular function | D-xylose:proton symporter activity | 2 |
| molecular function | diaminopropionate ammonia-lyase activity | 2 |
| molecular function | oxidoreductase activity, acting on the CH-NH group of donors, oxygen as acceptor | 2 |
| molecular function | phosphoenolpyruvate mutase activity | 2 |
| molecular function | nucleobase-containing compound kinase activity | 2 |
| molecular function | rubidium ion transmembrane transporter activity | 2 |
| molecular function | cutinase activity | 2 |
| molecular function | galactinol-sucrose galactosyltransferase activity | 2 |
| molecular function | acetyl-CoA hydrolase activity | 2 |
| molecular function | proteasome binding | 2 |
| molecular function | aspartyl esterase activity | 2 |
| molecular function | mitogen-activated protein kinase binding | 2 |
| molecular function | tetracycline transporter activity | 2 |
| molecular function | peptidase activator activity | 2 |
| molecular function | GDP-Man:Man3GlcNAc2-PP-Dol alpha-1,2-mannosyltransferase activity | 2 |
| molecular function | acyl-phosphate glycerol-3-phosphate acyltransferase activity | 2 |
| molecular function | lauroyltransferase activity | 2 |
| molecular function | mycothione reductase activity | 2 |
| molecular function | threonine-type endopeptidase activity | 2 |
| molecular function | dATP pyrophosphohydrolase activity | 2 |
| molecular function | toxin-antitoxin pair type II binding | 2 |
| molecular function | maltooligosaccharide-importing ATPase activity | 2 |
| molecular function | adenosine kinase activity | 2 |
| molecular function | epoxide hydrolase activity | 2 |
| molecular function | chitin disaccharide deacetylase activity | 2 |
| molecular function | 4-hydroxy-4-methyl-2-oxoglutarate aldolase activity | 2 |
| molecular function | mRNA 3'-UTR AU-rich region binding | 2 |
| molecular function | flap endonuclease activity | 2 |
| molecular function | nuclear localization sequence binding | 2 |
| molecular function | 3',5'-nucleotide bisphosphate phosphatase activity | 2 |
| molecular function | estrogen receptor binding | 2 |
| molecular function | lipid phosphatase activity | 2 |
| molecular function | fructose binding | 2 |
| molecular function | tRNA (cytosine) methyltransferase activity | 2 |
| molecular function | L-2-hydroxyglutarate oxidase activity | 2 |
| molecular function | muconolactone delta-isomerase activity | 2 |
| molecular function | biphenyl-2,3-diol 1,2-dioxygenase activity | 2 |
| molecular function | lysine-acetylated histone binding | 2 |
| molecular function | chorismate lyase activity | 2 |
| molecular function | NAD(P)H nitroreductase activity | 2 |
| molecular function | L-idonate 5-dehydrogenase activity | 2 |
| molecular function | AMP-activated protein kinase activity | 2 |
| molecular function | 2-carboxy-D-arabinitol-1-phosphatase activity | 2 |
| molecular function | FMN transmembrane transporter activity | 2 |
| molecular function | cholesterol transporter activity | 2 |
| molecular function | galactosamine-6-phosphate isomerase activity | 2 |
| molecular function | acetoacetyl-CoA reductase activity | 2 |
| molecular function | arginine N-succinyltransferase activity | 2 |
| molecular function | L-methionine secondary active transmembrane transporter activity | 2 |
| molecular function | menaquinone-dependent protoporphyrinogen oxidase activity | 2 |
| molecular function | arginine:ornithine antiporter activity | 2 |
| molecular function | galactosyltransferase activity | 2 |
| molecular function | alpha-ribazole phosphatase activity | 2 |
| molecular function | 16S rRNA (guanine(1207)-N(2))-methyltransferase activity | 2 |
| molecular function | 2,3-dihydro-2,3-dihydroxybenzoate dehydrogenase activity | 2 |
| molecular function | prosthetic group binding | 2 |
| molecular function | S-carboxymethylcysteine synthase activity | 2 |
| molecular function | calcium-dependent ATPase activity | 2 |
| molecular function | activating transcription factor binding | 2 |
| molecular function | oxidoreductase activity, acting on diphenols and related substances as donors | 2 |
| molecular function | ATP-dependent 3'-5' RNA helicase activity | 2 |
| molecular function | mannokinase activity | 2 |
| molecular function | fucose-1-phosphate guanylyltransferase activity | 2 |
| molecular function | structural constituent of cuticle | 2 |
| molecular function | methionine-R-sulfoxide reductase activity | 2 |
| molecular function | D-alanine transmembrane transporter activity | 2 |
| molecular function | secondary active transmembrane transporter activity | 2 |
| molecular function | oxidoreductase activity, acting on NAD(P)H, oxygen as acceptor | 2 |
| molecular function | sialic acid transmembrane transporter activity | 2 |
| molecular function | toluene dioxygenase activity | 2 |
| molecular function | glucan 1,3-alpha-glucosidase activity | 2 |
| molecular function | exochitinase activity | 2 |
| molecular function | glutarate-semialdehyde dehydrogenase activity | 2 |
| molecular function | ATPase inhibitor activity | 2 |
| molecular function | S-methyl-5-thioadenosine phosphorylase activity | 2 |
| molecular function | glucose-6-phosphate 1-epimerase activity | 2 |
| molecular function | tRNA (cytosine-5-)-methyltransferase activity | 2 |
| molecular function | phenylserine aldolase activity | 2 |
| molecular function | 3-hydroxyacyl-CoA dehydratase activity | 2 |
| molecular function | coenzyme F390-A hydrolase activity | 2 |
| molecular function | lytic endotransglycosylase activity | 2 |
| molecular function | deoxyribonuclease V activity | 2 |
| molecular function | mannose-ethanolamine phosphotransferase activity | 2 |
| molecular function | fumarate reductase (NADH) activity | 2 |
| molecular function | vinylacetyl-CoA delta-isomerase activity | 2 |
| molecular function | fucokinase activity | 2 |
| molecular function | trans-2-enoyl-CoA reductase (NAD+) activity | 2 |
| molecular function | heteropolysaccharide binding | 2 |
| molecular function | oxygen sensor activity | 2 |
| molecular function | jasmonyl-Ile conjugate hydrolase activity | 2 |
| molecular function | sulfoacetaldehyde acetyltransferase activity | 2 |
| molecular function | phosphodiesterase I activity | 2 |
| molecular function | 3-demethylubiquinone-n 3-O-methyltransferase activity | 2 |
| molecular function | protein tag | 2 |
| molecular function | alkylglycerone-phosphate synthase activity | 2 |
| molecular function | rhamnulokinase activity | 2 |
| molecular function | bacterial-type RNA polymerase transcription factor activity, sequence-specific DNA binding | 2 |
| molecular function | myosin light chain binding | 2 |
| molecular function | cuprous ion binding | 2 |
| molecular function | CDP-diacylglycerol-inositol 3-phosphatidyltransferase activity | 2 |
| molecular function | racemase activity, acting on amino acids and derivatives | 2 |
| molecular function | peptide-serine-N-acetyltransferase activity | 2 |
| molecular function | troponin I binding | 2 |
| molecular function | pimelyl-[acyl-carrier protein] methyl ester esterase activity | 2 |
| molecular function | RNA polymerase II transcription corepressor activity | 2 |
| molecular function | troponin C binding | 2 |
| molecular function | alditol:NADP+ 1-oxidoreductase activity | 2 |
| molecular function | proteasome-activating ATPase activity | 2 |
| molecular function | acryloyl-CoA reductase (NADP+) activity | 2 |
| molecular function | D-cysteine desulfhydrase activity | 2 |
| molecular function | SUMO polymer binding | 2 |
| molecular function | feruloyl esterase activity | 2 |
| molecular function | oxidoreductase activity, acting on hydrogen as donor, NAD or NADP as acceptor | 2 |
| molecular function | D-amino-acid N-acetyltransferase activity | 2 |
| molecular function | beta-alanine transmembrane transporter activity | 2 |
| molecular function | nucleotide diphosphatase activity | 2 |
| molecular function | cardiolipin binding | 2 |
| molecular function | methylitaconate delta-isomerase activity | 2 |
| molecular function | L-cystine L-cysteine-lyase (deaminating) | 2 |
| molecular function | xylosyltransferase activity | 2 |
| molecular function | 1-phosphatidylinositol-3-kinase activity | 2 |
| molecular function | beta-N-acetylglucosaminylglycopeptide beta-1,4-galactosyltransferase activity | 2 |
| molecular function | isochorismatase activity | 2 |
| molecular function | GDP-glucosidase activity | 2 |
| molecular function | poly(ADP-ribose) glycohydrolase activity | 2 |
| molecular function | tryptophan 2,3-dioxygenase activity | 2 |
| molecular function | adenylyltransferase activity | 2 |
| molecular function | 3-methyl-2-oxobutanoate dehydrogenase (2-methylpropanoyl-transferring) activity | 2 |
| molecular function | proline-rich region binding | 2 |
| molecular function | alpha-glucosidase activity | 2 |
| molecular function | GDP-mannose mannosyl hydrolase activity | 2 |
| molecular function | 4-hydroxyproline epimerase activity | 2 |
| molecular function | glycerol-3-phosphatase activity | 2 |
| molecular function | riboflavin binding | 2 |
| molecular function | L(+)-tartrate dehydratase activity | 2 |
| molecular function | syntaxin binding | 2 |
| molecular function | neutral L-amino acid secondary active transmembrane transporter activity | 2 |
| molecular function | medium-chain fatty acid-CoA ligase activity | 2 |
| molecular function | hydroxyisourate hydrolase activity | 2 |
| molecular function | lipid kinase activity | 2 |
| molecular function | capsular-polysaccharide-transporting ATPase activity | 2 |
| molecular function | extracellular matrix structural constituent conferring tensile strength | 2 |
| molecular function | UDP-N-acetyl-D-glucosamine:N-acetylmuramoyl-L-alanyl-D-glutamyl-meso-2,6-diaminopimelyl-D-alanyl-D-alanine-diphosphoundecaprenol 4-beta-N-acetylglucosaminlytransferase activity | 2 |
| molecular function | nicotinamide riboside transmembrane transporter activity | 2 |
| molecular function | protein phosphatase inhibitor activity | 2 |
| molecular function | photoreceptor activity | 2 |
| molecular function | phosphatidylcholine synthase activity | 2 |
| molecular function | GPI-anchor transamidase activity | 2 |
| molecular function | agmatine deiminase activity | 2 |
| molecular function | tRNA (guanine(37)-N(1))-methyltransferase activity | 2 |
| molecular function | azobenzene reductase activity | 2 |
| molecular function | fumarylpyruvate hydrolase activity | 2 |
| molecular function | palmitoleoyl [acyl-carrier-protein]-dependent acyltransferase activity | 2 |
| molecular function | protein-lysine lysyltransferase activity | 2 |
| molecular function | tRNA-specific adenosine-34 deaminase activity | 2 |
| molecular function | L-fuculokinase activity | 2 |
| molecular function | polyamine transmembrane transporter activity | 2 |
| molecular function | methylaspartate mutase activity | 2 |
| molecular function | 3-mercaptopyruvate sulfurtransferase activity | 2 |
| molecular function | riboflavin reductase (NADH) activity | 2 |
| molecular function | inulinase activity | 2 |
| molecular function | ribonuclease D activity | 2 |
| molecular function | heparan sulfate proteoglycan binding | 2 |
| molecular function | bubble DNA binding | 2 |
| molecular function | nicotinate dehydrogenase activity | 2 |
| molecular function | testosterone dehydrogenase [NAD(P)] activity | 2 |
| molecular function | 2-octaprenyl-6-methoxy-1,4-benzoquinone methyltransferase activity | 2 |
| molecular function | cysteine-type endopeptidase inhibitor activity | 2 |
| molecular function | oligosaccharide transporting porin activity | 2 |
| molecular function | L-alanine transmembrane transporter activity | 2 |
| molecular function | 3,4-dihydroxyphenylacetate 2,3-dioxygenase activity | 2 |
| molecular function | carnitine racemase activity | 2 |
| molecular function | 3'-5' DNA helicase activity | 2 |
| molecular function | translation activator activity | 2 |
| molecular function | malate transmembrane transporter activity | 2 |
| molecular function | dTTP diphosphatase activity | 2 |
| molecular function | phenylacetyl-CoA 1,2-epoxidase activity | 2 |
| molecular function | 6-phospho-beta-galactosidase activity | 2 |
| molecular function | ubiquitin-like protein binding | 2 |
| molecular function | glycine betaine:proton symporter activity | 2 |
| molecular function | microtubule plus-end binding | 2 |
| molecular function | S-methylmethionine transmembrane transporter activity | 2 |
| molecular function | chlorophyllide a oxygenase [overall] activity | 2 |
| molecular function | 4-hydroxybenzoyl-CoA thioesterase activity | 2 |
| molecular function | GMP 5'-nucleotidase activity | 2 |
| molecular function | butyrate-CoA ligase activity | 2 |
| molecular function | 1-phosphatidylinositol-4-phosphate 3-kinase activity | 2 |
| molecular function | allophanate hydrolase activity | 1 |
| molecular function | ankyrin binding | 1 |
| molecular function | didemethylisoproturon amidohydrolase activity | 1 |
| molecular function | (3,5-dichlorophenylurea)acetate amidohydrolase activity | 1 |
| molecular function | mRNA (2'-O-methyladenosine-N6-)-methyltransferase activity | 1 |
| molecular function | 1,6-dihydroxycyclohexa-2,4-diene-1-carboxylate dehydrogenase activity | 1 |
| molecular function | 5' deoxyribonuclease (pyrimidine dimer) activity | 1 |
| molecular function | 2-hydroxydec-2,4-diene-1,10-dioate hydratase activity | 1 |
| molecular function | oligonucleotidase activity | 1 |
| molecular function | heme-copper terminal oxidase activity | 1 |
| molecular function | phosphatidylglycerol-membrane-oligosaccharide glycerophosphotransferase activity | 1 |
| molecular function | aminoacetone:oxygen oxidoreductase(deaminating) activity | 1 |
| molecular function | glucarate dehydratase activity | 1 |
| molecular function | S-methyl-5-thioribose kinase activity | 1 |
| molecular function | 2-methyl-6-phytyl-1,4-benzoquinone methyltransferase activity | 1 |
| molecular function | ferric-chelate reductase activity | 1 |
| molecular function | L-xylulokinase activity | 1 |
| molecular function | hexose-phosphate:inorganic phosphate antiporter activity | 1 |
| molecular function | transcriptional activator activity, RNA polymerase II transcription regulatory region sequence-specific binding | 1 |
| molecular function | ribonuclease T2 activity | 1 |
| molecular function | ARF guanyl-nucleotide exchange factor activity | 1 |
| molecular function | mannosyl-oligosaccharide 1,6-alpha-mannosidase activity | 1 |
| molecular function | transcription termination site sequence-specific DNA binding | 1 |
| molecular function | copper chaperone activity | 1 |
| molecular function | gluconate 2-dehydrogenase activity | 1 |
| molecular function | Enterobacter ribonuclease activity | 1 |
| molecular function | 3',5'-cyclic-GMP phosphodiesterase activity | 1 |
| molecular function | mediator complex binding | 1 |
| molecular function | ADP-ribosylation factor binding | 1 |
| molecular function | trans-2-methyl-5-isopropylhexa-2,5-dienoate-CoA ligase activity | 1 |
| molecular function | phosphoglycerate phosphatase activity | 1 |
| molecular function | N-acetylgalactosamine-6-sulfatase activity | 1 |
| molecular function | trans-feruloyl-CoA synthase activity | 1 |
| molecular function | hyalurononglucosaminidase activity | 1 |
| molecular function | oxidoreductase activity, acting on reduced flavodoxin as donor | 1 |
| molecular function | L-lysine transmembrane transporter activity | 1 |
| molecular function | lactonohydrolase activity | 1 |
| molecular function | tRNA (adenine-C2-)-methyltransferase activity | 1 |
| molecular function | cadmium ion sensor activity | 1 |
| molecular function | phosphoribosylglycinamide formyltransferase 2 activity | 1 |
| molecular function | retinyl-palmitate esterase activity | 1 |
| molecular function | 2,5-dioxovalerate dehydrogenase (NADP+) activity | 1 |
| molecular function | cerebroside-sulfatase activity | 1 |
| molecular function | guanine/thymine mispair binding | 1 |
| molecular function | perillic acid:CoA ligase (ADP-forming) activity | 1 |
| molecular function | ribulokinase activity | 1 |
| molecular function | polysialic-acid O-acetyltransferase activity | 1 |
| molecular function | core promoter binding | 1 |
| molecular function | protein-arginine N-methyltransferase activity | 1 |
| molecular function | decarboxylation-driven active transmembrane transporter activity | 1 |
| molecular function | trans-aconitate 2-methyltransferase activity | 1 |
| molecular function | UDP-glucosyltransferase activity | 1 |
| molecular function | precorrin-2 C20-methyltransferase activity | 1 |
| molecular function | coenzyme F420-0:L-glutamate ligase activity | 1 |
| molecular function | aryl-alcohol dehydrogenase (NAD+) activity | 1 |
| molecular function | transcription factor activity, RNA polymerase II core promoter sequence-specific binding involved in preinitiation complex assembly | 1 |
| molecular function | alpha-ketoglutarate:proton symporter activity | 1 |
| molecular function | calcium-dependent cysteine-type endopeptidase activity | 1 |
| molecular function | ortho-trichlorophenol reductive dehalogenase activity | 1 |
| molecular function | N-acetylcitrulline deacetylase activity | 1 |
| molecular function | octanol dehydrogenase activity | 1 |
| molecular function | fosmidomycin transporter activity | 1 |
| molecular function | lupeol synthase activity | 1 |
| molecular function | chromatin DNA binding | 1 |
| molecular function | threo-3-hydroxyaspartate ammonia-lyase activity | 1 |
| molecular function | poly(beta-D-mannuronate) lyase activity | 1 |
| molecular function | cysteine-type endopeptidase inhibitor activity involved in apoptotic process | 1 |
| molecular function | palmitoyl-CoA ligase activity | 1 |
| molecular function | MHC class II protein complex binding | 1 |
| molecular function | sialate O-acetylesterase activity | 1 |
| molecular function | MHC class II protein binding, via antigen binding groove | 1 |
| molecular function | enterochelin esterase activity | 1 |
| molecular function | chromo shadow domain binding | 1 |
| molecular function | phosphoserine:homoserine phosphotransferase activity | 1 |
| molecular function | [acyl-carrier-protein] S-acetyltransferase activity | 1 |
| molecular function | phosphoethanolamine N-methyltransferase activity | 1 |
| molecular function | N2-acetyl-L-lysine deacetylase activity | 1 |
| molecular function | macrophage migration inhibitory factor binding | 1 |
| molecular function | adenosylcobyric acid synthase (glutamine-hydrolyzing) activity | 1 |
| molecular function | phytochelatin transmembrane transporter ATPase activity | 1 |
| molecular function | ligand-dependent nuclear receptor transcription coactivator activity | 1 |
| molecular function | GDP-mannose hydrolase activity | 1 |
| molecular function | dynein complex binding | 1 |
| molecular function | protein-N-terminal asparagine amidohydrolase activity | 1 |
| molecular function | cupric reductase activity | 1 |
| molecular function | L-arabinitol 2-dehydrogenase activity | 1 |
| molecular function | peroxisome matrix targeting signal-1 binding | 1 |
| molecular function | succinyltransferase activity | 1 |
| molecular function | 3-methyl-5-hydroxy-6-(3-carboxy-3-oxopropenyl)-1H-2-pyridon hydratase-aldolase activity | 1 |
| molecular function | 3-(3-hydroxyphenyl)propionate hydroxylase activity | 1 |
| molecular function | N-cyclohexylformamide amidohydrolase activity | 1 |
| molecular function | oxidoreductase activity, acting on the CH-NH2 group of donors, oxygen as acceptor | 1 |
| molecular function | methylmalonyl-CoA decarboxylase activity | 1 |
| molecular function | DNA-3-methylguanine glycosylase activity | 1 |
| molecular function | sedoheptulokinase activity | 1 |
| molecular function | syndecan binding | 1 |
| molecular function | double-stranded DNA 5'-3' exodeoxyribonuclease activity | 1 |
| molecular function | zinc-exporting ATPase activity | 1 |
| molecular function | anthranilate 1,2-dioxygenase (deaminating, decarboxylating) activity | 1 |
| molecular function | cinnamyl-alcohol dehydrogenase activity | 1 |
| molecular function | testosterone 17-beta-dehydrogenase (NADP+) activity | 1 |
| molecular function | myosin phosphatase activity | 1 |
| molecular function | carboxyl-O-methyltransferase activity | 1 |
| molecular function | histone-arginine N-methyltransferase activity | 1 |
| molecular function | modified amino acid binding | 1 |
| molecular function | haptoglobin binding | 1 |
| molecular function | trans-4-(1'-hydroxynaphth-2'-yl)-2-oxobut-3-enoate hydratase-aldolase activity | 1 |
| molecular function | oxidoreductase activity, acting on paired donors, with incorporation or reduction of molecular oxygen, reduced flavin or flavoprotein as one donor, and incorporation of one atom of oxygen | 1 |
| molecular function | N-carbamoylputrescine amidase activity | 1 |
| molecular function | histone-lysine N-methyltransferase activity | 1 |
| molecular function | benzaldehyde dehydrogenase (NAD+) activity | 1 |
| molecular function | 2-hexaprenyl-6-methoxy-1,4-benzoquinone methyltransferase activity | 1 |
| molecular function | trans-octaprenyltranstransferase activity | 1 |
| molecular function | microtubule minus-end binding | 1 |
| molecular function | acetylenecarboxylate hydratase activity, producing 3-hydroxypropenoate | 1 |
| molecular function | mRNA (guanine-N7-)-methyltransferase activity | 1 |
| molecular function | eukaryotic translation initiation factor 2alpha kinase activity | 1 |
| molecular function | repressing transcription factor binding | 1 |
| molecular function | oleoyl-CoA ligase activity | 1 |
| molecular function | bis(5'-adenosyl)-triphosphatase activity | 1 |
| molecular function | cobyrinic acid a,c-diamide synthase activity | 1 |
| molecular function | Ser-tRNA(Thr) hydrolase activity | 1 |
| molecular function | annealing helicase activity | 1 |
| molecular function | serine C-palmitoyltransferase activity | 1 |
| molecular function | 2,4,4-trimethylpentanoate-CoA ligase activity | 1 |
| molecular function | sodium:sulfate symporter activity | 1 |
| molecular function | 6-aminohexanoate-dimer hydrolase activity | 1 |
| molecular function | G-protein coupled GABA receptor activity | 1 |
| molecular function | heparanase activity | 1 |
| molecular function | DEAD/H-box RNA helicase binding | 1 |
| molecular function | bromide peroxidase activity | 1 |
| molecular function | rRNA (adenine-C2-)-methyltransferase activity | 1 |
| molecular function | RNA polymerase II transcription factor binding | 1 |
| molecular function | ubiquitin conjugating enzyme binding | 1 |
| molecular function | (R,R)-butanediol dehydrogenase activity | 1 |
| molecular function | arabinogalactan endo-1,4-beta-galactosidase activity | 1 |
| molecular function | anaphase-promoting complex binding | 1 |
| molecular function | gamma-tubulin binding | 1 |
| molecular function | hydrogensulfite reductase activity | 1 |
| molecular function | aryl-CoA synthetase (ADP-forming) activity | 1 |
| molecular function | ferredoxin-nitrite reductase activity | 1 |
| molecular function | glutaminyl-peptide cyclotransferase activity | 1 |
| molecular function | protein channel activity | 1 |
| molecular function | sulfur compound transmembrane transporter activity | 1 |
| molecular function | 8-oxo-7,8-dihydroguanine DNA N-glycosylase activity | 1 |
| molecular function | tyrosine 2,3-aminomutase activity | 1 |
| molecular function | cis-1,2-dihydrobenzene-1,2-diol dehydrogenase activity | 1 |
| molecular function | UDPsulfoquinovose synthase activity | 1 |
| molecular function | mannosyl-3-phosphoglycerate phosphatase activity | 1 |
| molecular function | microcin uptake transporter activity | 1 |
| molecular function | RNA polymerase II activity | 1 |
| molecular function | 2-enoate reductase activity | 1 |
| molecular function | N-acyl-D-aspartate deacylase activity | 1 |
| molecular function | L-amino acid efflux transmembrane transporter activity | 1 |
| molecular function | indole-3-acetic acid amido synthetase activity | 1 |
| molecular function | malate:proton symporter activity | 1 |
| molecular function | pyridoxal 4-dehydrogenase activity | 1 |
| molecular function | ferritin receptor activity | 1 |
| molecular function | rhamnose transmembrane transporter activity | 1 |
| molecular function | RNA polymerase I core binding | 1 |
| molecular function | tetracycline:proton antiporter activity | 1 |
| molecular function | 23S rRNA (guanine(748)-N(1))-methyltransferase activity | 1 |
| molecular function | tryptamine:oxygen oxidoreductase (deaminating) activity | 1 |
| molecular function | mitogen-activated protein kinase kinase kinase binding | 1 |
| molecular function | SUMO-specific protease activity | 1 |
| molecular function | aconitate decarboxylase activity | 1 |
| molecular function | histone demethylase activity (H3-K36 specific) | 1 |
| molecular function | sulfur amino acid transmembrane transporter activity | 1 |
| molecular function | homoisocitrate dehydrogenase activity | 1 |
| molecular function | geranylgeranyl-diphosphate geranylgeranyltransferase activity | 1 |
| molecular function | lipopeptide binding | 1 |
| molecular function | butyrolactone hydrolase activity | 1 |
| molecular function | oxidoreductase activity, acting on CH or CH2 groups | 1 |
| molecular function | thiamine kinase activity | 1 |
| molecular function | inorganic anion exchanger activity | 1 |
| molecular function | mandelamide amidase activity | 1 |
| molecular function | troponin T binding | 1 |
| molecular function | succinylornithine transaminase activity | 1 |
| molecular function | methanol dehydrogenase activity | 1 |
| molecular function | single-stranded DNA-dependent ATP-dependent 3'-5' DNA helicase activity | 1 |
| molecular function | glutathionylspermidine amidase activity | 1 |
| molecular function | aminocarboxymuconate-semialdehyde decarboxylase activity | 1 |
| molecular function | nitric oxide binding | 1 |
| molecular function | succinate-CoA ligase activity | 1 |
| molecular function | glutamate synthase (NADH) activity | 1 |
| molecular function | N-isopropylacetanilide amidohydrolase activity | 1 |
| molecular function | type I interferon receptor binding | 1 |
| molecular function | ionotropic glutamate receptor binding | 1 |
| molecular function | oligosaccharide 4-alpha-D-glucosyltransferase activity | 1 |
| molecular function | arogenate dehydratase activity | 1 |
| molecular function | phosphatidylserine 1-acylhydrolase activity | 1 |
| molecular function | reelin receptor activity | 1 |
| molecular function | tyrosine phenol-lyase activity | 1 |
| molecular function | 3-dehydroshikimate dehydratase activity | 1 |
| molecular function | D-xylose transmembrane transporter activity | 1 |
| molecular function | serine-glyoxylate transaminase activity | 1 |
| molecular function | iduronate-2-sulfatase activity | 1 |
| molecular function | (3R)-3-isopropenyl-6-oxoheptanoate:CoA ligase (ADP-forming) activity | 1 |
| molecular function | oxidoreductase activity, acting on NAD(P)H, NAD(P) as acceptor | 1 |
| molecular function | cAMP deaminase activity | 1 |
| molecular function | peptidoglycan receptor activity | 1 |
| molecular function | 1,4-alpha-glucan 6-alpha-glucosyltransferase activity | 1 |
| molecular function | 2-keto-3-deoxygalactonate aldolase activity | 1 |
| molecular function | D-xylose 1-dehydrogenase (NADP+) activity | 1 |
| molecular function | 2-hydroxyhexa-2,4-dienoate hydratase activity | 1 |
| molecular function | malonyl-CoA synthetase activity | 1 |
| molecular function | endosulfan hemisulfate sulfatase activity | 1 |
| molecular function | erythrulose reductase activity | 1 |
| molecular function | nucleosome binding | 1 |
| molecular function | alkenylglycerophosphocholine hydrolase activity | 1 |
| molecular function | E-phenylitaconyl-CoA hydratase activity | 1 |
| molecular function | o-hydroxylaminobenzoate mutase activity | 1 |
| molecular function | branched-chain acyl-CoA synthetase (ADP-forming) activity | 1 |
| molecular function | 3-hydroxypropionyl-CoA synthetase activity | 1 |
| molecular function | mannosyl-oligosaccharide 1,2-alpha-mannosidase activity | 1 |
| molecular function | glycoprotein transporter activity | 1 |
| molecular function | mannan endo-1,4-beta-mannosidase activity | 1 |
| molecular function | ornithine-oxo-acid transaminase activity | 1 |
| molecular function | Ral GTPase binding | 1 |
| molecular function | receptor tyrosine kinase binding | 1 |
| molecular function | methyl jasmonate esterase activity | 1 |
| molecular function | serine hydrolase activity | 1 |
| molecular function | capsular polysaccharide transmembrane transporter activity | 1 |
| molecular function | beta-amyrin synthase activity | 1 |
| molecular function | 1,2-bis(4-hydroxyphenyl)-2-proponol dehydratase activity | 1 |
| molecular function | phosphatidylinositol phospholipase C activity | 1 |
| molecular function | pteridine reductase activity | 1 |
| molecular function | L-aminoadipate-semialdehyde dehydrogenase activity | 1 |
| molecular function | 3-hydroxy-2-methylbutyryl-CoA dehydrogenase activity | 1 |
| molecular function | protein phosphatase 1 binding | 1 |
| molecular function | gamma-aminobutyric acid:sodium symporter activity | 1 |
| molecular function | taurine dioxygenase activity | 1 |
| molecular function | sulfinoalanine decarboxylase activity | 1 |
| molecular function | phospholipase D activity | 1 |
| molecular function | FMN reductase (NADH) activity | 1 |
| molecular function | 3-isopropenyl-6-oxoheptanoyl-CoA synthetase activity | 1 |
| molecular function | protein kinase activator activity | 1 |
| molecular function | lactoyl-CoA dehydratase activity | 1 |
| molecular function | pivalyl-CoA mutase activity | 1 |
| molecular function | store-operated calcium channel activity | 1 |
| molecular function | SNAP receptor activity | 1 |
| molecular function | deoxyribonucleoside 5'-monophosphate N-glycosidase activity | 1 |
| molecular function | (3R)-hydroxymyristoyl-[acyl-carrier-protein] dehydratase activity | 1 |
| molecular function | neurotransmitter:sodium symporter activity | 1 |
| molecular function | fatty acid ligase activity | 1 |
| molecular function | 2-phosphosulfolactate phosphatase activity | 1 |
| molecular function | nucleotide kinase activity | 1 |
| molecular function | cis-2-methyl-5-isopropylhexa-2,5-dienoate-CoA ligase activity | 1 |
| molecular function | naringenin 3-dioxygenase activity | 1 |
| molecular function | fatty-acyl-CoA reductase (alcohol-forming) activity | 1 |
| molecular function | uracil oxygenase activity | 1 |
| molecular function | N-acylphosphatidylethanolamine-specific phospholipase D activity | 1 |
| molecular function | gamma-N-formylaminovinylacetate hydrolase activity | 1 |
| molecular function | structural constituent of chitin-based larval cuticle | 1 |
| molecular function | oligoribonucleotidase activity | 1 |
| molecular function | phosphatidylinositol N-acetylglucosaminyltransferase activity | 1 |
| molecular function | adipate-CoA ligase activity | 1 |
| molecular function | Ser(Gly)-tRNA(Ala) hydrolase activity | 1 |
| molecular function | prenylcysteine methylesterase activity | 1 |
| molecular function | acylpyruvate hydrolase activity | 1 |
| molecular function | potassium ion leak channel activity | 1 |
| molecular function | AP-2 adaptor complex binding | 1 |
| molecular function | RNA polymerase II transcription factor activity, TBP-class protein binding, involved in preinitiation complex assembly | 1 |
| molecular function | limonene 8-hydratase activity | 1 |
| molecular function | cadmium-exporting ATPase activity | 1 |
| molecular function | S-acyltransferase activity | 1 |
| molecular function | succinate-CoA ligase (GDP-forming) activity | 1 |
| molecular function | isoquinoline 1-oxidoreductase activity | 1 |
| molecular function | DNA polymerase processivity factor activity | 1 |
| molecular function | phosphocholine hydrolase activity | 1 |
| molecular function | dimethylargininase activity | 1 |
| molecular function | structural constituent of chromatin | 1 |
| molecular function | oxidative RNA demethylase activity | 1 |
| molecular function | xylan alpha-1,2-glucuronosidase activity | 1 |
| molecular function | murein tripeptide carboxypeptidase activity | 1 |
| molecular function | trans-1,2-dihydrobenzene-1,2-diol dehydrogenase activity | 1 |
| molecular function | extracellular matrix binding | 1 |
| molecular function | naphthyl-2-methylene-succinyl-CoA lyase activity | 1 |
| molecular function | urea carboxylase activity | 1 |
| molecular function | DNA polymerase binding | 1 |
| molecular function | 5-oxoprolinase (ATP-hydrolyzing) activity | 1 |
| molecular function | double-stranded RNA-specific ribonuclease activity | 1 |
| molecular function | oxidoreductase activity, acting on the aldehyde or oxo group of donors, disulfide as acceptor | 1 |
| molecular function | inositol monophosphate phosphatase activity | 1 |
| molecular function | transcription factor activity, core RNA polymerase binding | 1 |
| molecular function | insulin receptor binding | 1 |
| molecular function | blue light photoreceptor activity | 1 |
| molecular function | carotene 7,8-desaturase activity | 1 |
| molecular function | RNA-directed RNA polymerase activity | 1 |
| molecular function | oxoglutarate dehydrogenase (NAD+) activity | 1 |
| molecular function | phosphatase regulator activity | 1 |
| molecular function | 4-methyloct-2-enoyl-CoA hydratase activity | 1 |
| molecular function | 9,9'-dicis-carotene:quinone oxidoreductase activity | 1 |
| molecular function | carbon-oxygen lyase activity, acting on phosphates | 1 |
| molecular function | thiophene-2-carboxylate-CoA ligase activity | 1 |
| molecular function | U3 snoRNA binding | 1 |
| molecular function | dinucleotide insertion or deletion binding | 1 |
| molecular function | alpha-1,4-glucan synthase activity | 1 |
| molecular function | (S)-2-haloacid dehalogenase activity | 1 |
| molecular function | benzoylformate decarboxylase activity | 1 |
| molecular function | G-protein coupled peptide receptor activity | 1 |
| molecular function | ethanolamine kinase activity | 1 |
| molecular function | red or far-red light photoreceptor activity | 1 |
| molecular function | CDP-alcohol phosphatidyltransferase activity | 1 |
| molecular function | allose kinase activity | 1 |
| molecular function | ceramide glucosyltransferase activity | 1 |
| molecular function | holo-citrate lyase synthase activity | 1 |
| molecular function | cyclic-GMP-AMP synthase activity | 1 |
| molecular function | cyclohex-1-ene-1-carboxyl-CoA hydratase activity | 1 |
| molecular function | perillyl-CoA hydratase activity | 1 |
| molecular function | mannosyl-oligosaccharide 1,3-alpha-mannosidase activity | 1 |
| molecular function | preprotein binding | 1 |
| molecular function | zinc D-Ala-D-Ala carboxypeptidase activity | 1 |
| molecular function | gluconolactonase activity | 1 |
| molecular function | methyl indole-3-acetate esterase activity | 1 |
| molecular function | benzoate-CoA ligase activity | 1 |
| molecular function | growth factor activity | 1 |
| molecular function | rRNA (adenine) methyltransferase activity | 1 |
| molecular function | cyclin-dependent protein serine/threonine kinase activity | 1 |
| molecular function | protein-N-terminal glutamine amidohydrolase activity | 1 |
| molecular function | xylulokinase activity | 1 |
| molecular function | translation regulator activity | 1 |
| molecular function | O-acetyl-ADP-ribose deacetylase activity | 1 |
| molecular function | cardiolipin hydrolase activity | 1 |
| molecular function | O-succinylbenzoate synthase activity | 1 |
| molecular function | clathrin binding | 1 |
| molecular function | glutamine binding | 1 |
| molecular function | 3-chloro-D-alanine dehydrochlorinase activity | 1 |
| molecular function | peptide antigen binding | 1 |
| molecular function | molybdenum cofactor cytidylyltransferase activity | 1 |
| molecular function | 4'-(2-hydroxyisopropyl)phenylurea amidohydrolase activity | 1 |
| molecular function | transmembrane receptor protein kinase activity | 1 |
| molecular function | 6-hydroxycyclohex-1-ene-1-carboxyl-CoA hydratase activity | 1 |
| molecular function | DNA-7-methylguanine glycosylase activity | 1 |
| molecular function | protein serine/threonine phosphatase inhibitor activity | 1 |
| molecular function | endosulfan diol hydrolyase (cyclizing) activity | 1 |
| molecular function | farnesyl-diphosphate farnesyltransferase activity | 1 |
| molecular function | 3-oxo-2-(2'-pentenyl)cyclopentane-1-octanoic acid CoA ligase activity | 1 |
| molecular function | intramolecular transferase activity, transferring hydroxy groups | 1 |
| molecular function | xylonate dehydratase activity | 1 |
| molecular function | cytosine C-5 DNA demethylase activity | 1 |
| molecular function | DNA/RNA helicase activity | 1 |
| molecular function | extracellular matrix constituent conferring elasticity | 1 |
| molecular function | molybdopterin-synthase sulfurtransferase activity | 1 |
| molecular function | citronellyl-CoA ligase activity | 1 |
| molecular function | 7,9,9'-tricis-neurosporene:quinone oxidoreductase activity | 1 |
| molecular function | maltose alpha-D-glucosyltransferase activity | 1 |
| molecular function | formylmethionine deformylase activity | 1 |
| molecular function | phosphorus-oxygen lyase activity | 1 |
| molecular function | sulfite oxidase activity | 1 |
| molecular function | 4-hydroxybenzoate 3-monooxygenase activity | 1 |
| molecular function | glyceraldehyde-3-phosphate dehydrogenase (NADP+) (non-phosphorylating) activity | 1 |
| molecular function | vanillin dehydrogenase activity | 1 |
| molecular function | nucleoside deoxyribosyltransferase activity | 1 |
| molecular function | NADP-retinol dehydrogenase activity | 1 |
| molecular function | glucosylceramidase activity | 1 |
| molecular function | WW domain binding | 1 |
| molecular function | 2-methylhex-2-enoyl-CoA hydratase activity | 1 |
| molecular function | sterol 3-beta-glucosyltransferase activity | 1 |
| molecular function | myristoyltransferase activity | 1 |
| molecular function | rDNA binding | 1 |
| molecular function | 5-(carboxyamino)imidazole ribonucleotide synthase activity | 1 |
| molecular function | trehalose transmembrane transporter activity | 1 |
| molecular function | polyubiquitin binding | 1 |
| molecular function | cyclo-ligase activity | 1 |
| molecular function | 2-hydroxyisobutyrate dehydratase activity | 1 |
| molecular function | beta-amylase activity | 1 |
| molecular function | N-1-naphthylphthalamic acid binding | 1 |
| molecular function | iprodione amidohydrolase activity | 1 |
| molecular function | 2-octoprenyl-3-methyl-6-methoxy-1,4-benzoquinone hydroxylase activity | 1 |
| molecular function | maltodextrin phosphorylase activity | 1 |
| molecular function | methyl salicylate esterase activity | 1 |
| molecular function | nitric-oxide synthase binding | 1 |
| molecular function | ethanolamine transmembrane transporter activity | 1 |
| molecular function | 3-hydroxypalmitoyl-[acyl-carrier-protein] dehydratase activity | 1 |
| molecular function | ATP-dependent polydeoxyribonucleotide 5'-hydroxyl-kinase activity | 1 |
| molecular function | 1-acyl-2-lysophosphatidylserine acylhydrolase activity | 1 |
| molecular function | heteroduplex DNA loop binding | 1 |
| molecular function | GDP-Man:Man1GlcNAc2-PP-Dol alpha-1,3-mannosyltransferase activity | 1 |
| molecular function | apolipoprotein binding | 1 |
| molecular function | 4-hydroxyphenylacetaldehyde dehydrogenase activity | 1 |
| molecular function | hydroquinone:oxygen oxidoreductase activity | 1 |
| molecular function | protein kinase A regulatory subunit binding | 1 |
| molecular function | dihydroneopterin monophosphate phosphatase activity | 1 |
| molecular function | UTP diphosphatase activity | 1 |
| molecular function | nitrilase activity | 1 |
| molecular function | glycerol dehydrogenase [NADP+] activity | 1 |
| molecular function | 5'-3' exodeoxyribonuclease activity | 1 |
| molecular function | phosphatidylinositol trisphosphate phosphatase activity | 1 |
| molecular function | 23S rRNA (guanine(745)-N(1))-methyltransferase activity | 1 |
| molecular function | adenosine 5'-monophosphoramidase activity | 1 |
| molecular function | ornithine cyclodeaminase activity | 1 |
| molecular function | 2,4,4-trimethylpent-2-enoyl-CoA hydratase activity | 1 |
| molecular function | hydroxydechloroatrazine ethylaminohydrolase activity | 1 |
| molecular function | glucose-1,6-bisphosphate synthase activity | 1 |
| molecular function | lead ion transmembrane transporter activity | 1 |
| molecular function | N-methyl-L-amino-acid oxidase activity | 1 |
| molecular function | bile acid-exporting ATPase activity | 1 |
| molecular function | nucleosomal DNA binding | 1 |
| molecular function | pentaerythritol trinitrate reductase activity | 1 |
| molecular function | structural constituent of chitin-based cuticle | 1 |
| molecular function | aliphatic-amine oxidase activity | 1 |
| molecular function | pyruvate-flavodoxin oxidoreductase activity | 1 |
| molecular function | transferase activity, transferring nitrogenous groups | 1 |
| molecular function | D-alanine:2-oxoglutarate aminotransferase activity | 1 |
| molecular function | 2,4-dihydroxyhept-2-ene-1,7-dioate aldolase activity | 1 |
| molecular function | MAP kinase tyrosine/serine/threonine phosphatase activity | 1 |
| molecular function | GTP-dependent polyribonucleotide 5'-hydroxyl-kinase activity | 1 |
| molecular function | acetylene hydratase activity | 1 |
| molecular function | nucleoside kinase activity | 1 |
| molecular function | dehydrodolichyl diphosphate synthase activity | 1 |
| molecular function | hexuronate transmembrane transporter activity | 1 |
| molecular function | tRNA guanylyltransferase activity | 1 |
| molecular function | chitooligosaccharide deacetylase activity | 1 |
| molecular function | beta-tubulin binding | 1 |
| molecular function | 3,4-dihydrocoumarin hydrolase activity | 1 |
| molecular function | gated channel activity | 1 |
| molecular function | tumor necrosis factor receptor binding | 1 |
| molecular function | 2-oxo-delta3-4,5,5-trimethylcyclopentenylacetyl-CoA synthetase activity | 1 |
| molecular function | serine-type exopeptidase activity | 1 |
| molecular function | 2-oxo-hept-3-ene-1,7-dioate hydratase activity | 1 |
| molecular function | diacetylchitobiose deacetylase activity | 1 |
| molecular function | ubiquitin-protein transferase activator activity | 1 |
| molecular function | myristoyl-CoA ligase activity | 1 |
| molecular function | alkanesulfonate monooxygenase activity | 1 |
| molecular function | 16S rRNA (adenine(1518)-N(6)/adenine(1519)-N(6))-dimethyltransferase activity | 1 |
| molecular function | deoxyribonuclease inhibitor activity | 1 |
| molecular function | perillic acid:CoA ligase (AMP-forming) activity | 1 |
| molecular function | exopeptidase activity | 1 |
| molecular function | DNA-7-methyladenine glycosylase activity | 1 |
| molecular function | protein phosphatase regulator activity | 1 |
| molecular function | ubiquitin binding | 1 |
| molecular function | Rab guanyl-nucleotide exchange factor activity | 1 |
| molecular function | 1-oxa-2-oxocycloheptane lactonase activity | 1 |
| molecular function | tRNA (m5U54) methyltransferase activity | 1 |
| molecular function | sulfolactone hydrolase activity | 1 |
| molecular function | 8-hydroxy-dADP phosphatase activity | 1 |
| molecular function | (S,S)-butanediol dehydrogenase activity | 1 |
| molecular function | benzoate transporter activity | 1 |
| molecular function | 4-phytase activity | 1 |
| molecular function | site-specific endodeoxyribonuclease activity, specific for altered base | 1 |
| molecular function | acyl-[acyl-carrier-protein] hydrolase activity | 1 |
| molecular function | ATP-dependent 5'-3' RNA helicase activity | 1 |
| molecular function | very-low-density lipoprotein particle binding | 1 |
| molecular function | cholest-4-en-3-one 26-monooxygenase activity | 1 |
| molecular function | cyclopropanecarboxylate-CoA ligase activity | 1 |
| molecular function | lead ion binding | 1 |
| molecular function | complement component C3b binding | 1 |
| molecular function | autoinducer-2 kinase activity | 1 |
| molecular function | structural constituent of eye lens | 1 |
| molecular function | oleoyl-[acyl-carrier-protein] hydrolase activity | 1 |
| molecular function | L-threonine transmembrane transporter activity | 1 |
| molecular function | osmosensor activity | 1 |
| molecular function | cAMP-dependent protein kinase activity | 1 |
| molecular function | nitrate reductase (NADPH) activity | 1 |
| molecular function | trans-4-[2-(3-hydroxy)-thionaphthenyl]-2-oxo-3-butenoate hydratase activity | 1 |
| molecular function | ATP-dependent NAD(P)H-hydrate dehydratase activity | 1 |
| molecular function | gamma-glutamylcyclotransferase activity | 1 |
| molecular function | glycerol-phosphate:inorganic phosphate antiporter activity | 1 |
| molecular function | arsenate ion transmembrane transporter activity | 1 |
| molecular function | 23S rRNA (guanine(1835)-N(2))-methyltransferase activity | 1 |
| molecular function | second spliceosomal transesterification activity | 1 |
| molecular function | alpha-methylacyl-CoA racemase activity | 1 |
| molecular function | CTD phosphatase activity | 1 |
| molecular function | TAP binding | 1 |
| molecular function | cytochrome-b5 reductase activity, acting on NAD(P)H | 1 |
| molecular function | phenethylamine:oxygen oxidoreductase (deaminating) activity | 1 |
| molecular function | 5-chloro-2-oxopent-4-enoate hydratase activity | 1 |
| molecular function | exodeoxyribonuclease activity | 1 |
| molecular function | 2-hydroxychromene-2-carboxylate isomerase activity | 1 |
| molecular function | adenine binding | 1 |
| molecular function | NEDD8-specific protease activity | 1 |
| molecular function | tRNA nucleotidyltransferase activity | 1 |
| molecular function | cholesterol 26-hydroxylase activity | 1 |
| molecular function | CDP-diacylglycerol diphosphatase activity | 1 |
| molecular function | protein-arginine omega-N asymmetric methyltransferase activity | 1 |
| molecular function | actin lateral binding | 1 |
| molecular function | alkenylglycerophosphoethanolamine hydrolase activity | 1 |
| molecular function | dITP diphosphatase activity | 1 |
| molecular function | UDP-N-acetyl-D-mannosamine dehydrogenase activity | 1 |
| molecular function | hydrogen:proline symporter activity | 1 |
| molecular function | anion channel activity | 1 |
| molecular function | RNA polymerase I activity | 1 |
| molecular function | magnesium-importing ATPase activity | 1 |
| molecular function | telomeric DNA binding | 1 |
| molecular function | C-3 sterol dehydrogenase (C-4 sterol decarboxylase) activity | 1 |
| molecular function | baruol synthase activity | 1 |
| molecular function | thiol-dependent ubiquitinyl hydrolase activity | 1 |
| molecular function | pyrimidine nucleoside binding | 1 |
| molecular function | arginine-importing ATPase activity | 1 |
| molecular function | L-methionine-(R)-S-oxide reductase activity | 1 |
| molecular function | dolichyldiphosphatase activity | 1 |
| molecular function | (3R)-3-isopropenyl-6-oxoheptanoate:CoA ligase (AMP-forming) activity | 1 |
| molecular function | RNA polymerase II activating transcription factor binding | 1 |
| molecular function | purine nucleobase binding | 1 |
| molecular function | intramolecular oxidoreductase activity, transposing C=C bonds | 1 |
| molecular function | trans-o-hydroxybenzylidenepyruvate hydratase-aldolase activity | 1 |
| molecular function | aminoglycoside 6'-N-acetyltransferase activity | 1 |
| molecular function | 4-carboxy-2-hydroxymuconate semialdehyde hemiacetal dehydrogenase activity | 1 |
| molecular function | sinapyl alcohol dehydrogenase activity | 1 |
| molecular function | biuret amidohydrolase activity | 1 |
| molecular function | protein deacetylase activity | 1 |
| molecular function | sphingomyelin phosphodiesterase activity | 1 |
| molecular function | cyanate hydratase activity | 1 |
| molecular function | Rho-dependent protein serine/threonine kinase activity | 1 |
| molecular function | endosulfan lactone lactonase activity | 1 |
| molecular function | GPI anchor binding | 1 |
| molecular function | immunoglobulin receptor binding | 1 |
| molecular function | DNA-N1-methyladenine dioxygenase activity | 1 |
| molecular function | succinylglutamate desuccinylase activity | 1 |
| molecular function | ribosomal S6-glutamic acid ligase activity | 1 |
| molecular function | L-altrarate dehydratase activity | 1 |
| molecular function | dCTP diphosphatase activity | 1 |
| molecular function | guanosine tetraphosphate binding | 1 |
| molecular function | benzoyl acetate-CoA ligase activity | 1 |
| molecular function | misfolded RNA binding | 1 |
| molecular function | dihydromonapterin reductase activity | 1 |
| molecular function | glycosylphosphatidylinositol diacylglycerol-lyase activity | 1 |
| molecular function | arginyltransferase activity | 1 |
| molecular function | nicotianamine synthase activity | 1 |
| molecular function | UDP-N-acetylglucosamine-undecaprenyl-phosphate N-acetylglucosaminephosphotransferase activity | 1 |
| molecular function | (R)-carnitine:4-(trimethylammonio)butanoate antiporter activity | 1 |
| molecular function | glutathionylspermidine synthase activity | 1 |
| molecular function | pyrimidine dimer DNA N-glycosylase activity | 1 |
| molecular function | 3-hydroxyphenyl propionate:hydrogen ion symporter activity | 1 |
| molecular function | nitrate reductase (NADH) activity | 1 |
| molecular function | 2-dehydro-3-deoxyglucarate aldolase activity | 1 |
| molecular function | mentha-1,3-dione-CoA ligase activity | 1 |
| molecular function | rubredoxin-NADP reductase activity | 1 |
| molecular function | 2,4'-dihydroxyacetophenone dioxygenase activity | 1 |
| molecular function | glyceraldehyde-3-phosphate dehydrogenase (NAD(P)+) (phosphorylating) activity | 1 |
| molecular function | transcriptional activator activity, bacterial-type RNA polymerase core promoter proximal region sequence-specific binding | 1 |
| molecular function | TPR domain binding | 1 |
| molecular function | bacterial-type RNA polymerase core promoter sequence-specific DNA binding | 1 |
| molecular function | L-serine transmembrane transporter activity | 1 |
| molecular function | carbon-sulfur lyase activity | 1 |
| molecular function | protein-disulfide reductase (glutathione) activity | 1 |
| molecular function | aminoglycoside N-acetyltransferase activity | 1 |
| molecular function | rRNA (pseudouridine-N3-)-methyltransferase activity | 1 |
| molecular function | polynucleotide 3'-phosphatase activity | 1 |
| molecular function | N,N-dimethylaniline monooxygenase activity | 1 |
| molecular function | methylated histone binding | 1 |
| molecular function | XTP diphosphatase activity | 1 |
| molecular function | transferase activity, transferring one-carbon groups | 1 |
| molecular function | phosphoribulokinase activity | 1 |
| molecular function | beta-2-microglobulin binding | 1 |
| molecular function | branched-chain-2-oxoacid decarboxylase activity | 1 |
| molecular function | ethanolamine-phosphate cytidylyltransferase activity | 1 |
| molecular function | trans-L-3-hydroxyproline dehydratase activity | 1 |
| molecular function | isonicotinic acid hydrazide hydrolase activity | 1 |
| molecular function | T cell receptor binding | 1 |
| molecular function | 3-carboxymuconate cycloisomerase type II activity | 1 |
| molecular function | double-stranded methylated DNA binding | 1 |
| molecular function | transmembrane receptor histidine kinase activity | 1 |
| molecular function | pyrroline-2-carboxylate reductase activity | 1 |
| molecular function | versicolorin reductase activity | 1 |
| molecular function | precorrin-6A synthase (deacetylating) activity | 1 |
| molecular function | 3-oxoadipyl-CoA thiolase activity | 1 |
| molecular function | cytokine receptor activity | 1 |
| molecular function | kynureninase activity | 1 |
| molecular function | L-lysine 6-monooxygenase (NADPH) activity | 1 |
| molecular function | inorganic cation transmembrane transporter activity | 1 |
| molecular function | complement component C3a binding | 1 |
| molecular function | hydrolase activity, acting on acid carbon-phosphorus bonds | 1 |
| molecular function | translation termination factor activity | 1 |
| molecular function | p53 binding | 1 |
| molecular function | calcium-activated potassium channel activity | 1 |
| molecular function | enoyl-[acyl-carrier-protein] reductase activity | 1 |
| molecular function | protein phosphorylated amino acid binding | 1 |
| molecular function | 2-octaprenylphenol hydroxylase activity | 1 |
| molecular function | tetrahydroxynaphthalene reductase activity | 1 |
| molecular function | N-acetylgalactosamine-6-phosphate deacetylase activity | 1 |
| molecular function | pyruvate transmembrane transporter activity | 1 |
| molecular function | L-rhamnonate dehydratase activity | 1 |
| molecular function | cis-aconitamide amidase activity | 1 |
| molecular function | glycerol dehydrogenase [NAD(P)+] activity | 1 |
| molecular function | L-erythro-3-methylmalyl-CoA dehydratase activity | 1 |
| molecular function | hemi-methylated DNA-binding | 1 |
| molecular function | semaphorin receptor activity | 1 |
| molecular function | protein-DNA loading ATPase activity | 1 |
| molecular function | protein kinase A binding | 1 |
| molecular function | wide pore channel activity | 1 |
| molecular function | pivalate-CoA ligase activity | 1 |
| molecular function | retinal dehydrogenase activity | 1 |
| molecular function | proteinase activated receptor binding | 1 |
| molecular function | malate dehydrogenase (menaquinone) activity | 1 |
| molecular function | low-affinity glucose:sodium symporter activity | 1 |
| molecular function | pyrimidine nucleotide binding | 1 |
| molecular function | dextrin alpha-glucosidase activity | 1 |
| molecular function | pristanate-CoA ligase activity | 1 |
| molecular function | structural constituent of peritrophic membrane | 1 |
| molecular function | thyroid hormone receptor binding | 1 |
| molecular function | cytokine binding | 1 |
| molecular function | 3'-5' RNA helicase activity | 1 |
| molecular function | gamma-aminobutyric acid transmembrane transporter activity | 1 |
| molecular function | iron chaperone activity | 1 |
| molecular function | UDP-glucuronate 4-epimerase activity | 1 |
| molecular function | ferredoxin-nitrate reductase activity | 1 |
| molecular function | telomerase activity | 1 |
| molecular function | menth-2-enone hydratase activity | 1 |
| molecular function | acetate:proton symporter activity | 1 |
| molecular function | very-low-density lipoprotein particle receptor activity | 1 |
| molecular function | 2,4-dichlorobenzoate-CoA ligase activity | 1 |
| molecular function | misfolded protein binding | 1 |
| molecular function | MutSalpha complex binding | 1 |
| molecular function | mitochondrion targeting sequence binding | 1 |
| molecular function | MHC class II protein binding | 1 |
| molecular function | enterobactin transporter activity | 1 |
| molecular function | all-trans-retinyl-palmitate hydrolase, all-trans-retinol forming activity | 1 |
| molecular function | deoxycytidine deaminase activity | 1 |
| molecular function | transcription corepressor binding | 1 |
| molecular function | phosphatidylinositol-3-phosphatase activity | 1 |
| molecular function | steroid dehydrogenase activity, acting on the CH-CH group of donors | 1 |
| molecular function | endosulfan sulfate hydrolase activity | 1 |
| molecular function | IgE binding | 1 |
| molecular function | sialic acid:proton symporter activity | 1 |
| molecular function | testosterone dehydrogenase (NAD+) activity | 1 |
| molecular function | N6-hydroxylysine O-acetyltransferase activity | 1 |
| molecular function | hexose:proton symporter activity | 1 |
| NA | NA | 48 |
