# Supplementary figures and images for: Metagenomic analysis of captive Amur tiger faecal microbiome
Source: BMC Vet Res. 2018 Dec 4;14:379. doi: 10.1186/s12917-018-1696-5 (PMC6278063; doi:10.1186/s12917-018-1696-5)

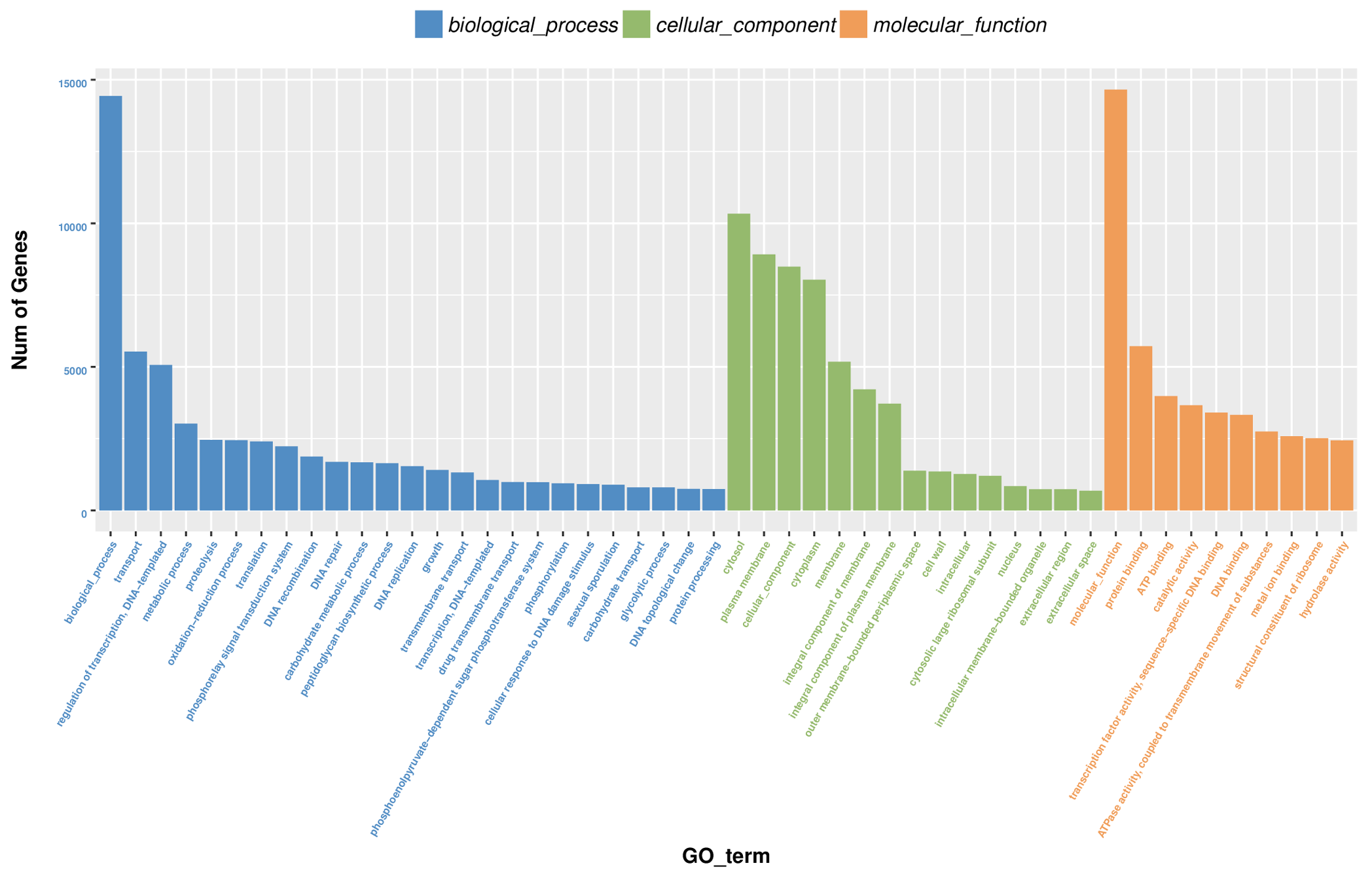

Supplement: Supplementary file 7 — GO annotations of the Amur tiger faecal metagenome. (TIF 9899 kb) [file 12917_2018_1696_MOESM7_ESM.tif]

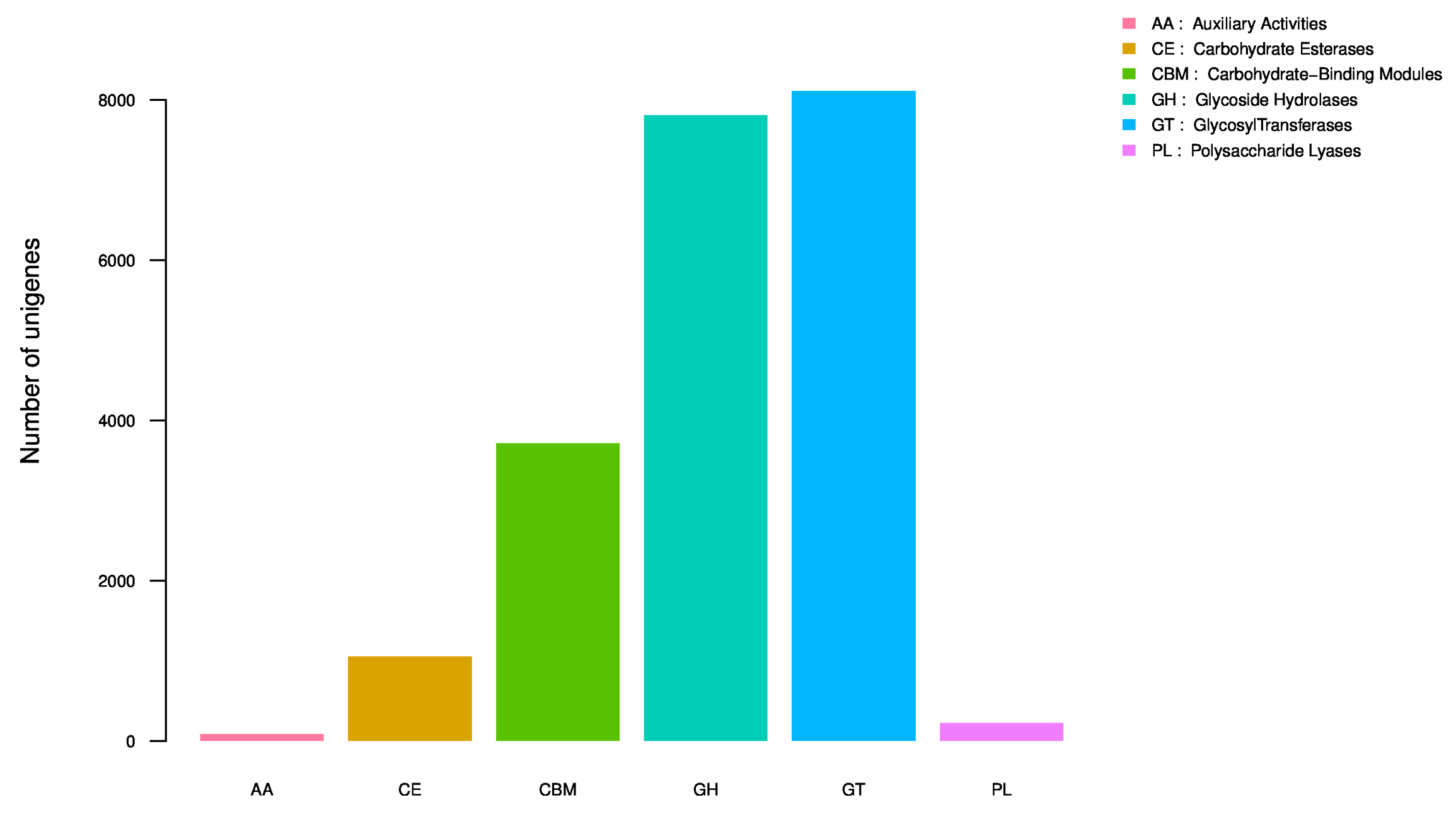

Supplement: Supplementary file 9 — CAZy classification of the Amur tiger faecal metagenome. (TIF 9289 kb) [file 12917_2018_1696_MOESM9_ESM.tif]
