# Supplementary material for: Metagenomic analysis of captive Amur tiger faecal microbiome
Source: BMC Vet Res. 2018 Dec 4;14:379. doi: 10.1186/s12917-018-1696-5 (PMC6278063; doi:10.1186/s12917-018-1696-5)
Supplement: Supplementary file 8 — CAZy annotations of the Amur tiger metagenome. (DOCX 48 kb) [file 12917_2018_1696_MOESM8_ESM.docx]

**Additional file 8: CAZy annotations of the Amur tiger metagenome.**

| Query | CAZy hit | NCBIAnno | CAZyLevel1 | CAZyLevel2 |
| --- | --- | --- | --- | --- |
| Unigene1 | AMQ12787.1 | X polypeptide (plasmid) [Escherichia coli] | Glycoside Hydrolases | GH23 |
| Unigene2 | CBL26300.1 | Type II secretory pathway, pullulanase PulA and related glycosidases [Ruminococcus torques L2-14] | Carbohydrate-Binding Modules | CBM48 |
| Unigene2 | CBL26300.1 | Type II secretory pathway, pullulanase PulA and related glycosidases [Ruminococcus torques L2-14] | Glycoside Hydrolases | GH13 |
| Unigene10 | ADH00730.1 | endo-beta-N-acetylglucosaminidase family protein [Bifidobacterium longum subsp. longum JDM301] | Glycoside Hydrolases | GH85 |
| Unigene50 | CBL24654.1 | penicillin-binding protein, 1A family [Blautia obeum A2-162] | GlycosylTransferases | GT51 |
| Unigene53 | ZP 02866877.1 | hypothetical protein CLOSPI 00679 [Clostridium spiroforme DSM 1552] | Carbohydrate-Binding Modules | CBM32 |
| Unigene53 | ZP 02866877.1 | hypothetical protein CLOSPI 00679 [Clostridium spiroforme DSM 1552] | Glycoside Hydrolases | GH36 |
| Unigene63 | AMD97704.1 | alpha-L-fucosidase [Streptococcus sp. oral taxon 431] | Glycoside Hydrolases | GH29 |
| Unigene66 | CCH61763.1 | hypothetical protein TBLA 0F02210 [Tetrapisispora blattae CBS 6284] | Carbohydrate-Binding Modules | CBM18 |
| Unigene66 | CCH61763.1 | hypothetical protein TBLA 0F02210 [Tetrapisispora blattae CBS 6284] | Glycoside Hydrolases | GH16 |
| Unigene77 | BAQ01109.1 | putative glycosyltransferase [Escherichia coli] | GlycosylTransferases | GT2 |
| Unigene92 | CCT65987.1 | related to mixed-linked glucanase precursor MLG1 [Fusarium fujikuroi IMI 58289] | Glycoside Hydrolases | GH16 |
| Unigene117 | AGT25470.1 | Sucrose-6-phosphate hydrolase [Klebsiella pneumoniae JM45] | Glycoside Hydrolases | GH32 |
| Unigene121 | AHA66737.1 | 6-phospho-beta-glucosidase [Shigella dysenteriae 1617] | Glycoside Hydrolases | GH1 |
| Unigene124 | EFN52293.1 | hypothetical protein CHLNCDRAFT 58899 [Chlorella variabilis] | GlycosylTransferases | GT96 |
| Unigene129 | AER83045.1 | hypothetical protein i02 0452 [Escherichia coli str. 'clone D i2'] | Carbohydrate Esterases | CE14 |
| Unigene155 | ABG84962.1 | discoidin domain protein [Clostridium perfringens ATCC 13124] | Carbohydrate-Binding Modules | CBM32 |
| Unigene155 | ABG84962.1 | discoidin domain protein [Clostridium perfringens ATCC 13124] | Carbohydrate-Binding Modules | CBM51 |
| Unigene196 | ACD23569.1 | starch binding domain protein [Clostridium botulinum B str. Eklund 17B (NRP)] | Carbohydrate-Binding Modules | CBM20 |
| Unigene209 | CAX41254.1 | conserved hypothetical protein [Candida dubliniensis CD36] | GlycosylTransferases | GT91 |
| Unigene212 | AFM00719.1 | putative peptidoglycan-binding domain-containing protein [Desulfitobacterium dehalogenans ATCC 51507] | Carbohydrate-Binding Modules | CBM50 |
| Unigene218 | ADE67533.1 | putative exported cell wall-binding protein [Bacillus megaterium QM B1551] | Carbohydrate-Binding Modules | CBM50 |
| Unigene251 | ADY57267.1 | glycoside hydrolase family 18 [Syntrophobotulus glycolicus DSM 8271] | Glycoside Hydrolases | GH18 |
| Unigene252 | AHO16406.1 | glycogen debranching protein [Salmonella enterica subsp. enterica serovar Enteritidis str. EC20121179] | Glycoside Hydrolases | GH13 |
| Unigene265 | AIS04007.1 | Teichoic acid ABC transporter, ATP-binding protein [Lactococcus lactis] | Carbohydrate-Binding Modules | CBM50 |
| Unigene279 | CAL53384.1 | DDX20 HUMAN Probable ATP-dependent RNA helicase DDX20 (ISS) [Ostreococcus tauri] | Carbohydrate Esterases | CE11 |
| Unigene306 | ZP 02868533.1 | hypothetical protein CLOSPI 02375 [Clostridium spiroforme DSM 1552] | Glycoside Hydrolases | GH73 |
| Unigene312 | AHZ46894.1 | beta-N-acetylhexosaminidase [Streptococcus sp. VT 162] | Glycoside Hydrolases | GH20 |
| Unigene324 | AMD89422.1 | ABC transporter [Desulfovibrio fairfieldensis] | GlycosylTransferases | GT4 |
| Unigene349 | AMD96221.1 | exopolysaccharide biosynthesis protein [Streptococcus sp. oral taxon 431] | GlycosylTransferases | GT8 |
| Unigene369 | AFD24470.1 | Glycoside hydrolase, family 3-like protein [Deinococcus gobiensis I-0] | Glycoside Hydrolases | GH3 |
| Unigene376 | ACD75811.1 | glycosy ltransferase [Escherichia coli] | GlycosylTransferases | GT4 |
| Unigene412 | AKK50560.1 | putative 6-phospho-beta-glucosidase [Escherichia coli PCN033] | Glycoside Hydrolases | GH4 |
| Unigene415 | ACR74768.1 | arabinogalactan endo-1,4-beta-galactosidase [[Eubacterium rectale] ATCC 33656] | Glycoside Hydrolases | GH53 |
| Unigene427 | ACP87452.1 | Sequence 352 from patent US 7504120 | Carbohydrate-Binding Modules | CBM22 |
| Unigene427 | ACP87452.1 | Sequence 352 from patent US 7504120 | Glycoside Hydrolases | GH10 |
| Unigene457 | ADK30641.1 | UDP-3-O-[3-hydroxymyristoyl] N-acetylglucosamine deacetylase [Brachyspira pilosicoli 95/1000] | Carbohydrate Esterases | CE11 |
| Unigene470 | BAI44118.1 | chitin binding protein 4 [Magnaporthe oryzae] | Carbohydrate-Binding Modules | CBM18 |
| Unigene491 | AEA20065.1 | aldose 1-epimerase [Prevotella denticola F0289] | Glycoside Hydrolases | GH43 |
| Unigene544 | CDS91732.1 | conserved exported hypothetical protein [Sphingobacterium sp. PM2-P1-29] | Carbohydrate-Binding Modules | CBM32 |
| Unigene552 | CBL01236.1 | Alpha-galactosidases/6-phospho-beta-glucosidases, family 4 of glycosyl hydrolases [Faecalibacterium prausnitzii SL3/3] | Glycoside Hydrolases | GH4 |
| Unigene562 | AMN32462.1 | discoidin domain protein [Clostridium perfringens] | Carbohydrate-Binding Modules | CBM32 |
| Unigene562 | AMN32462.1 | discoidin domain protein [Clostridium perfringens] | Carbohydrate-Binding Modules | CBM51 |
| Unigene569 | ALA45444.1 | hypothetical protein ADP64 00061 [Achromobacter phage phiAxp-2] | Glycoside Hydrolases | GH23 |
| Unigene586 | AEI12495.1 | UDP-N-acetylmuramate/alanine ligase [Cellulomonas gilvus ATCC 13127] | GlycosylTransferases | GT28 |
| Unigene593 | ALM27847.1 | phage tail protein [Bacillus pumilus] | Glycoside Hydrolases | GH23 |
| Unigene600 | BAN75646.1 | truncated putative alpha-mannosidase [Lactobacillus casei subsp. casei ATCC 393] | Glycoside Hydrolases | GH38 |
| Unigene617 | AKG73456.1 | hypothetical protein AAT16 04045 [Salinicoccus halodurans] | GlycosylTransferases | GT4 |
| Unigene647 | BAT81564.1 | hypothetical protein VIGAN 03131500 [Vigna angularis var. angularis] | Carbohydrate-Binding Modules | CBM20 |
| Unigene655 | ZP 03683877.1 | hypothetical protein CATMIT 02538 [Catenibacterium mitsuokai DSM 15897] | Carbohydrate-Binding Modules | CBM32 |
| Unigene655 | ZP 03683877.1 | hypothetical protein CATMIT 02538 [Catenibacterium mitsuokai DSM 15897] | Glycoside Hydrolases | GH20 |
| Unigene657 | AAO80135.1 | glycosyl hydrolase, family 1 [Enterococcus faecalis V583] | Glycoside Hydrolases | GH1 |
| Unigene687 | AMN32208.1 | cell wall-binding protein [Clostridium perfringens] | Glycoside Hydrolases | GH23 |
| Unigene694 | ABX41810.1 | Peptidoglycan-binding domain 1 protein [Lachnoclostridium phytofermentans ISDg] | Glycoside Hydrolases | GH19 |
| Unigene707 | AEE54321.1 | riboflavin biosynthesis protein RibF [Haliscomenobacter hydrossis DSM 1100] | GlycosylTransferases | GT2 |
| Unigene717 | AEF86905.1 | beta-1,4-xylosidase with transcriptional regulator of AraC/XylS family [Treponema primitia ZAS-2] | Glycoside Hydrolases | GH39 |
| Unigene738 | EGX51422.1 | hypothetical protein AOL s00054g121 [Arthrobotrys oligospora ATCC 24927] | Glycoside Hydrolases | GH55 |
| Unigene755 | ACK42700.1 | glycosyl transferase group 1 [Dictyoglomus turgidum DSM 6724] | GlycosylTransferases | GT4 |
| Unigene768 | CEJ75177.1 | putative alpha-amylase [[Clostridium] sordellii] | Glycoside Hydrolases | GH13 |
| Unigene807 | CEJ74586.1 | Glycosyltransferase [[Clostridium] sordellii] | GlycosylTransferases | GT2 |
| Unigene823 | ADL05597.1 | Peptidoglycan glycosyltransferase [[Clostridium] saccharolyticum WM1] | GlycosylTransferases | GT51 |
| Unigene829 | AFM26684.1 | UDP-3-O-(3-hydroxymyristoyl) N-acetylglucosamine deacetylase [Desulfomonile tiedjei DSM 6799] | Carbohydrate Esterases | CE11 |
| Unigene857 | AKA68908.1 | cwp66-like protein/N-acetylmuramoyl-L-alanine amidase [Clostridium scatologenes] | Carbohydrate-Binding Modules | CBM54 |
| Unigene861 | ACL59902.1 | hypothetical protein Mnod 5056 [Methylobacterium nodulans ORS 2060] | Glycoside Hydrolases | GH23 |
| Unigene869 | AJB53641.1 | glycosyl transferase [Escherichia coli] | GlycosylTransferases | GT2 |
| Unigene898 | CEJ75234.1 | putative phage cell wall hydrolase [[Clostridium] sordellii] | Carbohydrate-Binding Modules | CBM50 |
| Unigene899 | AIR70972.1 | hypothetical protein LH89 17775 [Cedecea neteri] | Carbohydrate-Binding Modules | CBM32 |
| Unigene910 | AKF31057.1 | peptidase M23 [Bacillus velezensis] | Glycoside Hydrolases | GH23 |
| Unigene919 | AMS11809.1 | pectate lyase [Erysipelothrix rhusiopathiae] | Glycoside Hydrolases | GH110 |
| Unigene941 | ADR25763.1 | beta-D-galactosidase [Escherichia coli O83:H1 str. NRG 857C] | Glycoside Hydrolases | GH2 |
| Unigene961 | AMN30576.1 | cell wall-binding protein TcpG (plasmid) [Clostridium perfringens] | Glycoside Hydrolases | GH73 |
| Unigene989 | AMC08994.1 | hypothetical protein AT726 08865 [Turicibacter sp. H121] | Glycoside Hydrolases | GH73 |
| Unigene1006 | AMM42870.1 | LRR-RLK, partial [Vernicia fordii] | Carbohydrate-Binding Modules | CBM57 |
| Unigene1033 | AMN35302.1 | pullulanase [Clostridium perfringens] | Carbohydrate-Binding Modules | CBM41 |
| Unigene1033 | AMN35302.1 | pullulanase [Clostridium perfringens] | Carbohydrate-Binding Modules | CBM48 |
| Unigene1033 | AMN35302.1 | pullulanase [Clostridium perfringens] | Glycoside Hydrolases | GH13 |
| Unigene1033 | AMN35302.1 | pullulanase [Clostridium perfringens] | Glycoside Hydrolases | GH13 |
| Unigene1040 | AAN17394.1 | Putataive InsB from Escherichia coli [Oryza sativa Japonica Group] | GlycosylTransferases | GT77 |
| Unigene1042 | CTQ97265.1 | Maltodextrin glucosidase (EC 3.2.1.20) [Kibdelosporangium sp. MJ126-NF4] | Carbohydrate-Binding Modules | CBM32 |
| Unigene1042 | CTQ97265.1 | Maltodextrin glucosidase (EC 3.2.1.20) [Kibdelosporangium sp. MJ126-NF4] | Glycoside Hydrolases | GH31 |
| Unigene1048 | AAQ72464.1 | alpha-fucosidase [Bifidobacterium bifidum JCM 1254] | Glycoside Hydrolases | GH95 |
| Unigene1055 | CBK91496.1 | Glycosyltransferase [[Eubacterium] rectale DSM 17629] | GlycosylTransferases | GT4 |
| Unigene1066 | CBL05434.1 | Lipopolysaccharide biosynthesis proteins, LPS:glycosyltransferases [Megamonas hypermegale ART12/1] | GlycosylTransferases | GT8 |
| Unigene1068 | AEY69616.1 | putative DNA methylase [Erwinia phage PEp14] | Glycoside Hydrolases | GH23 |
| Unigene1077 | AJA56769.1 | teichoic acid ABC transporter ATP-binding protein [Lactococcus lactis subsp. lactis] | Carbohydrate-Binding Modules | CBM50 |
| Unigene1079 | AJK44890.1 | glycosyltransferase [Burkholderia glumae PG1] | GlycosylTransferases | GT2 |
| Unigene1079 | AJK44890.1 | glycosyltransferase [Burkholderia glumae PG1] | GlycosylTransferases | GT4 |
| Unigene1102 | BAB47586.1 | alkaline pullulanase [Bacillus sp. KSM-1876] | Carbohydrate-Binding Modules | CBM41 |
| Unigene1102 | BAB47586.1 | alkaline pullulanase [Bacillus sp. KSM-1876] | Carbohydrate-Binding Modules | CBM48 |
| Unigene1102 | BAB47586.1 | alkaline pullulanase [Bacillus sp. KSM-1876] | Glycoside Hydrolases | GH13 |
| Unigene1123 | AEY69616.1 | putative DNA methylase [Erwinia phage PEp14] | Glycoside Hydrolases | GH23 |
| Unigene1155 | CBL26300.1 | Type II secretory pathway, pullulanase PulA and related glycosidases [Ruminococcus torques L2-14] | Carbohydrate-Binding Modules | CBM48 |
| Unigene1155 | CBL26300.1 | Type II secretory pathway, pullulanase PulA and related glycosidases [Ruminococcus torques L2-14] | Glycoside Hydrolases | GH13 |
| Unigene1170 | AII12421.1 | Teichoic acid ABC transporter [Lactococcus lactis subsp. lactis NCDO 2118] | Carbohydrate-Binding Modules | CBM50 |
| Unigene1193 | CAN65674.1 | hypothetical protein VITISV 002009 [Vitis vinifera] | Glycoside Hydrolases | GH28 |
| Unigene1259 | AJC25574.1 | Wall-associated protein [Bacillus sp. Pc3] | Carbohydrate-Binding Modules | CBM16 |
| Unigene1264 | AAO76732.1 | putative exported fucosidase [Bacteroides thetaiotaomicron VPI-5482] | Glycoside Hydrolases | GH29 |
| Unigene1288 | AKH70842.1 | lytic murein transglycosylase B [Spongiibacter sp. IMCC21906] | Glycoside Hydrolases | GH103 |
| Unigene1297 | CBL26134.1 | isoamylase [Ruminococcus torques L2-14] | Carbohydrate-Binding Modules | CBM48 |
| Unigene1297 | CBL26134.1 | isoamylase [Ruminococcus torques L2-14] | Glycoside Hydrolases | GH13 |
| Unigene1307 | AAF55402.1 | mucin related 89F [Drosophila melanogaster] | Carbohydrate-Binding Modules | CBM14 |
| Unigene1308 | CBL23266.1 | Putative glycosyl/glycerophosphate transferases involved in teichoic acid biosynthesis TagF/TagB/EpsJ/RodC [Blautia obeum A2-162] | GlycosylTransferases | GT4 |
| Unigene1310 | ADB31951.1 | cell division protein FtsW [Kribbella flavida DSM 17836] | GlycosylTransferases | GT28 |
| Unigene1312 | AFR30064.1 | beta-galactosidase LacZ [Arthrobacter sp. Rue61a] | Glycoside Hydrolases | GH2 |
| Unigene1333 | CBL18565.1 | glycogen/starch synthases, ADP-glucose type [Ruminococcus sp. SR1/5] | GlycosylTransferases | GT5 |
| Unigene1337 | ALC88354.1 | N-acetylglucosaminyltransferase [Bacillus sp. FJAT-22090] | GlycosylTransferases | GT2 |
| Unigene1356 | ADC46923.1 | glycosyl transferase GT2 family [Methanobrevibacter ruminantium M1] | GlycosylTransferases | GT2 |
| Unigene1360 | ALJ62047.1 | 3-oxoadipate enol-lactonase 2 [Bacteroides cellulosilyticus] | Glycoside Hydrolases | GH28 |
| Unigene1373 | AKG69460.1 | N-acetylmuramoyl-L-alanine amidase [Serratia fonticola] | Carbohydrate-Binding Modules | CBM50 |
| Unigene1386 | AGU86773.1 | Putative Murein transglycosylase-C [Serratia sp. ATCC 39006] | Glycoside Hydrolases | GH23 |
| Unigene1409 | BAN75402.1 | conserved hypothetical protein [Lactobacillus casei subsp. casei ATCC 393] | Glycoside Hydrolases | GH23 |
| Unigene1412 | AEJ53041.1 | exopolysaccharide biosynthesis protein, acetyltransferase [Streptococcus salivarius 57.I] | GlycosylTransferases | GT8 |
| Unigene1444 | AGN91810.1 | mannose-1-phosphate guanylyltransferase [Escherichia coli] | GlycosylTransferases | GT4 |
| Unigene1475 | ABS01822.1 | response regulator receiver protein [Kineococcus radiotolerans SRS30216 = ATCC BAA-149] | GlycosylTransferases | GT2 |
| Unigene1487 | BAQ02567.1 | hypothetical protein [Ralstonia phage RSL2] | Glycoside Hydrolases | GH19 |
| Unigene1495 | AJZ45867.1 | undecaprenyldiphospho-muramoylpentapeptide beta-N-acetylglucosaminyltransferase [Xanthomonas citri subsp. citri] | GlycosylTransferases | GT28 |
| Unigene1504 | AGK95561.1 | stage II sporulation protein P [Clostridium pasteurianum BC1] | Glycoside Hydrolases | GH73 |
| Unigene1518 | AGE56636.1 | chitin binding domain-containing protein [Acanthocystis turfacea Chlorella virus NE-JV-2] | Carbohydrate-Binding Modules | CBM14 |
| Unigene1527 | AAB18598.1 | alternate gene name rfa2 [Escherichia coli str. K-12 substr. MG1655] | GlycosylTransferases | GT9 |
| Unigene1540 | CEJ74586.1 | Glycosyltransferase [[Clostridium] sordellii] | GlycosylTransferases | GT2 |
| Unigene1546 | ADH00730.1 | endo-beta-N-acetylglucosaminidase family protein [Bifidobacterium longum subsp. longum JDM301] | Glycoside Hydrolases | GH85 |
| Unigene1552 | AFR09836.1 | carbohydrate binding domain protein [Nocardiopsis alba ATCC BAA-2165] | Carbohydrate-Binding Modules | CBM5 |
| Unigene1572 | CEK39427.1 | putative penicillin-binding protein [[Clostridium] sordellii] | GlycosylTransferases | GT51 |
| Unigene1573 | ACR72312.1 | Glycoside Hydrolase Family 25-like lysozyme/endolysin [[Eubacterium] eligens ATCC 27750] | Glycoside Hydrolases | GH25 |
| Unigene1586 | AJY10416.1 | transglycosylase SLT domain protein [Burkholderia dolosa AU0158] | Glycoside Hydrolases | GH23 |
| Unigene1589 | ALS26910.1 | N-acetylglucosamine-6-phosphate deacetylase [Paenibacillus sp. 32O-W] | Carbohydrate Esterases | CE9 |
| Unigene1598 | ACL49294.1 | ABC transporter related [Desulfovibrio desulfuricans subsp. desulfuricans str. ATCC 27774] | GlycosylTransferases | GT4 |
| Unigene1602 | AGF56149.1 | 6-phospho-beta-glucosidase AscB [Clostridium saccharoperbutylacetonicum N1-4(HMT)] | Glycoside Hydrolases | GH1 |
| Unigene1605 | CBK68010.1 | Heparinase II/III-like protein [Bacteroides xylanisolvens XB1A] | Polysaccharide Lyases | PL15 |
| Unigene1608 | AKK06402.1 | glycogen debranching enzyme GlgX [Corynebacterium mustelae] | Carbohydrate-Binding Modules | CBM48 |
| Unigene1608 | AKK06402.1 | glycogen debranching enzyme GlgX [Corynebacterium mustelae] | Glycoside Hydrolases | GH13 |
| Unigene1615 | AAM33211.1 | SEPTIN INTERACTING PROTEIN HOMOLOG [Dictyostelium discoideum] | GlycosylTransferases | GT49 |
| Unigene1618 | BAA25316.1 | sialyltransferase 0160 [Photobacterium damselae] | GlycosylTransferases | GT80 |
| Unigene1653 | ADX05725.1 | putative carbohydrate-active enzyme [uncultured organism] | Glycoside Hydrolases | GH5 |
| Unigene1675 | ACR71118.1 | Hypothetical protein EUBELI 00081 [[Eubacterium] eligens ATCC 27750] | GlycosylTransferases | GT2 |
| Unigene1684 | AIY82805.1 | lysM domain protein [Clostridium baratii str. Sullivan] | Carbohydrate-Binding Modules | CBM50 |
| Unigene1696 | ACF42936.1 | TPR repeat-containing protein [Pelodictyon phaeoclathratiforme BU-1] | GlycosylTransferases | GT41 |
| Unigene1706 | AAO41659.1 | CG33173 [Drosophila melanogaster] | Carbohydrate-Binding Modules | CBM14 |
| Unigene1710 | AMD89422.1 | ABC transporter [Desulfovibrio fairfieldensis] | GlycosylTransferases | GT4 |
| Unigene1717 | CCA60043.1 | Arylsulfatase [Streptomyces venezuelae ATCC 10712] | Carbohydrate-Binding Modules | CBM51 |
| Unigene1740 | AAK80956.1 | Glycosyltransferase [Clostridium acetobutylicum ATCC 824] | GlycosylTransferases | GT4 |
| Unigene1771 | BAC99989.1 | beta-N-acetylglucosaminidase [Clostridium paraputrificum] | Carbohydrate-Binding Modules | CBM32 |
| Unigene1771 | BAC99989.1 | beta-N-acetylglucosaminidase [Clostridium paraputrificum] | Glycoside Hydrolases | GH84 |
| Unigene1799 | AHM43529.1 | transglycosylase [Escherichia coli] | Glycoside Hydrolases | GH23 |
| Unigene1802 | ACL49294.1 | ABC transporter related [Desulfovibrio desulfuricans subsp. desulfuricans str. ATCC 27774] | GlycosylTransferases | GT4 |
| Unigene1804 | AEB06302.1 | polysaccharide deacetylase [Coriobacterium glomerans PW2] | Carbohydrate Esterases | CE4 |
| Unigene1822 | ALU14107.1 | glycoside hydrolase GH13 family [Eubacterium limosum] | Glycoside Hydrolases | GH13 |
| Unigene1855 | ADO82182.1 | glycosyl transferase family 2 [Ilyobacter polytropus DSM 2926] | GlycosylTransferases | GT2 |
| Unigene1902 | CCH61763.1 | hypothetical protein TBLA 0F02210 [Tetrapisispora blattae CBS 6284] | Carbohydrate-Binding Modules | CBM18 |
| Unigene1902 | CCH61763.1 | hypothetical protein TBLA 0F02210 [Tetrapisispora blattae CBS 6284] | Glycoside Hydrolases | GH16 |
| Unigene1928 | AEN97541.1 | N-acetylmuramidase/lysin [Roseburia hominis A2-183] | Glycoside Hydrolases | GH73 |
| Unigene1940 | CDL65439.1 | LysM domain/M23/M37 peptidase domain protein (plasmid) [wastewater metagenome] | Carbohydrate-Binding Modules | CBM50 |
| Unigene1941 | CEJ75234.1 | putative phage cell wall hydrolase [[Clostridium] sordellii] | Carbohydrate-Binding Modules | CBM50 |
| Unigene1950 | AMC08108.1 | hypothetical protein AT726 03505 [Turicibacter sp. H121] | Glycoside Hydrolases | GH73 |
| Unigene1965 | ACV05791.1 | hypothetical protein Ksed 07320 [Kytococcus sedentarius DSM 20547] | Glycoside Hydrolases | GH23 |
| Unigene1973 | ADF39834.1 | LysM domain protein [Bacillus megaterium DSM 319] | Carbohydrate-Binding Modules | CBM50 |
| Unigene1979 | AGV73396.1 | glycoside hydrolase GH85 family [Lactococcus lactis subsp. cremoris KW2] | Carbohydrate-Binding Modules | CBM32 |
| Unigene1980 | ALT69746.1 | glycosyl transferase GT2 family [Methanobrevibacter millerae] | GlycosylTransferases | GT2 |
| Unigene2012 | AKK37709.1 | choline dehydrogenase [Escherichia coli APEC O2] | Auxiliary Activities | AA3 |
| Unigene2023 | ABG83023.1 | cell wall binding repeat protein/mannosyl-glycoprotein endo-beta-N-acetylglucosamidase domain protein [Clostridium perfringens ATCC 13124] | Glycoside Hydrolases | GH73 |
| Unigene2036 | ACR62057.1 | beta-D-galactosidase [Escherichia coli BW2952] | Glycoside Hydrolases | GH2 |
| Unigene2037 | AEE54321.1 | riboflavin biosynthesis protein RibF [Haliscomenobacter hydrossis DSM 1100] | GlycosylTransferases | GT2 |
| Unigene2047 | AGG87862.1 | beta-phosphoglucomutase [Rhodanobacter denitrificans] | Glycoside Hydrolases | GH65 |
| Unigene2064 | AGX42211.1 | xylanase/chitin deacetylase [Clostridium saccharobutylicum DSM 13864] | Carbohydrate Esterases | CE4 |
| Unigene2074 | CBK81124.1 | Predicted xylanase/chitin deacetylase [Coprococcus catus GD/7] | Carbohydrate Esterases | CE4 |
| Unigene2075 | AAZ19599.1 | UDP-N-acetylglucosamine--N-acetylmuramyl-(pentapeptide) pyrophosphoryl-undecaprenol N-acetylglucosamine transferase [Psychrobacter arcticus 273-4] | GlycosylTransferases | GT28 |
| Unigene2092 | AFN84566.1 | beta-glucosidase-related glycosidase [uncultured bacterium scaffold00090] | Glycoside Hydrolases | GH3 |
| Unigene2100 | AJA56769.1 | teichoic acid ABC transporter ATP-binding protein [Lactococcus lactis subsp. lactis] | Carbohydrate-Binding Modules | CBM50 |
| Unigene2128 | ABG84084.1 | glycosyl hydrolase, family 31/fibronectin type III domain protein [Clostridium perfringens ATCC 13124] | Carbohydrate-Binding Modules | CBM32 |
| Unigene2128 | ABG84084.1 | glycosyl hydrolase, family 31/fibronectin type III domain protein [Clostridium perfringens ATCC 13124] | Carbohydrate-Binding Modules | CBM32 |
| Unigene2128 | ABG84084.1 | glycosyl hydrolase, family 31/fibronectin type III domain protein [Clostridium perfringens ATCC 13124] | Glycoside Hydrolases | GH31 |
| Unigene2141 | CEK39427.1 | putative penicillin-binding protein [[Clostridium] sordellii] | GlycosylTransferases | GT51 |
| Unigene2153 | AGK55699.1 | mannosyl-glycoprotein endo-beta-N-acetylglucosaminidase [Bacillus sp. 1NLA3E] | Glycoside Hydrolases | GH73 |
| Unigene2159 | AKA01905.1 | elongation factor Tu [Streptomyces albulus ZPM] | Carbohydrate-Binding Modules | CBM13 |
| Unigene2170 | ADC49105.1 | capsule anchoring protein CapD [Bacillus pseudofirmus OF4] | GlycosylTransferases | GT2 |
| Unigene2185 | ACV50538.1 | glycoside hydrolase family 2 sugar binding [Atopobium parvulum DSM 20469] | Carbohydrate-Binding Modules | CBM32 |
| Unigene2185 | ACV50538.1 | glycoside hydrolase family 2 sugar binding [Atopobium parvulum DSM 20469] | Glycoside Hydrolases | GH2 |
| Unigene2223 | AGY38647.1 | alpha-L-fucosidase [Streptococcus sp. I-P16] | Glycoside Hydrolases | GH95 |
| Unigene2273 | ADP35322.1 | 1,2-A-L-Fucosidase [Bifidobacterium bifidum PRL2010] | Glycoside Hydrolases | GH95 |
| Unigene2299 | AHF24012.1 | amylosucrase [uncultured bacterium Contig1756] | Glycoside Hydrolases | GH13 |
| Unigene2303 | AIY85063.1 | tetratricopeptide repeat family protein [Clostridium baratii str. Sullivan] | GlycosylTransferases | GT2 |
| Unigene2311 | AMO13190.1 | glycoside hydrolase [uncultured bacterium] | Glycoside Hydrolases | GH29 |
| Unigene2322 | AMC01573.1 | endopeptidase LytF [Aerococcus viridans] | Carbohydrate-Binding Modules | CBM50 |
| Unigene2331 | AME05014.1 | hypothetical protein AUL54 01095 [Bacillus sp. SDLI1] | Glycoside Hydrolases | GH23 |
| Unigene2333 | ABS01822.1 | response regulator receiver protein [Kineococcus radiotolerans SRS30216 = ATCC BAA-149] | GlycosylTransferases | GT2 |
| Unigene2345 | AHF10899.1 | cell wall-binding protein [Dehalobacter restrictus DSM 9455] | Glycoside Hydrolases | GH18 |
| Unigene2372 | ADH93004.1 | LPXTG-motif cell wall anchor domain protein [Arcanobacterium haemolyticum DSM 20595] | Glycoside Hydrolases | GH33 |
| Unigene2382 | AFU57543.1 | dolichyl-diphosphooligosaccharide--protein glycotransferase [Candidatus Nitrososphaera gargensis Ga9.2] | GlycosylTransferases | GT66 |
| Unigene2383 | CAD55485.1 | putative serine/threonine protein kinase (fragment) [Streptomyces coelicolor A3(2)] | Carbohydrate-Binding Modules | CBM13 |
| Unigene2396 | CDM67579.1 | Maltodextrin phosphorylase [Clostridium bornimense] | GlycosylTransferases | GT35 |
| Unigene2400 | AKP43468.1 | 6-phospho-alpha-glucosidase [Peptoclostridium difficile ATCC 9689 = DSM 1296] | Glycoside Hydrolases | GH4 |
| Unigene2403 | ACR74768.1 | arabinogalactan endo-1,4-beta-galactosidase [[Eubacterium rectale] ATCC 33656] | Glycoside Hydrolases | GH53 |
| Unigene2404 | AJT50020.1 | peptidoglycan-binding protein LysM [Lactobacillus mucosae LM1] | Carbohydrate-Binding Modules | CBM50 |
| Unigene2424 | AGF54571.1 | autolytic lysozyme Lyc [Clostridium saccharoperbutylacetonicum N1-4(HMT)] | Glycoside Hydrolases | GH25 |
| Unigene2463 | CBL25699.1 | Uncharacterised Sugar-binding Domain [Ruminococcus torques L2-14] | Glycoside Hydrolases | GH95 |
| Unigene2476 | AGZ24134.1 | penicillin-binding protein 2A [Streptococcus suis T15] | GlycosylTransferases | GT51 |
| Unigene2485 | ABR44858.1 | N-acetylglucosamine-6-phosphate deacetylase [Parabacteroides distasonis ATCC 8503] | Carbohydrate Esterases | CE9 |
| Unigene2490 | CBG69901.1 | putative 1,4-a-glucan branching enzyme [Streptomyces scabiei 87.22] | Carbohydrate-Binding Modules | CBM48 |
| Unigene2490 | CBG69901.1 | putative 1,4-a-glucan branching enzyme [Streptomyces scabiei 87.22] | Glycoside Hydrolases | GH13 |
